# LYZ Gene as a Novel Therapeutic Target and Diagnostic Biomarker in Glioblastoma: Insights from Multi-Omics Analysis and Functional Validation

**DOI:** 10.3390/biology15010009

**Published:** 2025-12-19

**Authors:** Nuoyan Zhu, Jiahui Wang, Liangliang Cai

**Affiliations:** 1 The First Clinical Medical College, Faculty of Medicine, Yangzhou University, Yangzhou 225000, China; 222205162@stu.yzu.edu.cn; 2 School of Basic Medical Sciences and School of Public Health, Faculty of Medicine, Yangzhou University, Yangzhou 225000, China

**Keywords:** LYZ gene, glioblastoma (GBM), therapeutic target, diagnostic biomarker, multi-omics analysis

## Abstract

Immune checkpoint blockade is one of the current treatments for glioblastoma (GBM), which is still a very aggressive and treatment-resistant tumor of the central nervous system. This study focused on the LYZ gene to find new therapeutic targets. We performed a thorough screening of differential gene expressions between GBM and normal samples using many databases (TCGA, GTEx, GEO, and CGGA). Because LYZ is significantly upregulated in GBM tissues and is associated with shorter patient survival periods, we identified it as a gene of interest. LYZ’s position on the exterior side of the plasma membrane and its participation in leukocyte-mediated immunity were identified by functional enrichment analysis, indicating a role in cell surface immune responses. Significant associations between LYZ expression and particular immune cell types were found using immune infiltration analysis, suggesting that LYZ may have an impact on the tumor microenvironment. Within GBM, single-cell research verified LYZ expression in macrophages and monocytes. LYZ was shown to express differently in GBM cell lines than in normal glial cells, according to cellular experimental verification. The LYZ gene’s functional importance in the pathophysiology of GBM was highlighted by the dramatic reduction in cell proliferation, motility, and invasion that resulted from its knockout. These results suggest that LYZ is a viable therapeutic target and possible GBM diagnostic biomarker, which calls for more research into its mechanisms of action and potential clinical use.

## Simple Summary

Glioblastoma (GBM) is a highly aggressive and treatment-resistant brain tumor with a poor prognosis for patients, who typically survive for around 15 months post-diagnosis. This study identifies the lysozyme (LYZ) gene as a promising new therapeutic target and diagnostic biomarker for GBM. Using multi-omics analysis across several databases (TCGA, GTEx, GEO, and CGGA), we found that LYZ is significantly upregulated in GBM tissues and associated with shorter patient survival times. Functional enrichment analysis revealed that LYZ is located on the cell surface and plays a role in immune responses, particularly involving leukocytes. Immune infiltration analysis indicated that LYZ expression is linked to specific immune cell types, suggesting its influence on the tumor microenvironment. Single-cell analysis confirmed LYZ expression in macrophages and monocytes within GBM. Cellular experiments showed that LYZ expression differs between GBM cell lines and normal glial cells, and knocking down the LYZ gene significantly reduced cell proliferation, motility, and invasion. These findings suggest that LYZ is a viable target for GBM therapy and a potential biomarker for diagnosis, warranting further research into its mechanisms and clinical applications.

## 1. Introduction

Glioblastoma, the most aggressive and malignant form of brain tumor, continues to pose a formidable challenge in the field of oncology, primarily due to its high mortality rate and limited treatment options [1,2]. Despite significant advancements in surgical techniques, radiation therapy, and chemotherapy, patients diagnosed with glioblastoma still face a dismal prognosis, with a median survival period of approximately 15 months [3,4]. The complexity and heterogeneity of glioblastoma, coupled with its propensity to infiltrate surrounding brain tissue, make complete surgical resection exceedingly difficult and recurrence inevitable [5,6]. Consequently, there is an urgent need to identify novel biomarkers and therapeutic targets to improve the diagnosis, prognosis, and treatment of this devastating disease.

The pathogenesis of glioblastoma is intricately linked to genetic alterations, including gene deletions, amplifications, and mutations [7]. These genetic aberrations disrupt normal cellular processes, leading to uncontrolled cell proliferation, invasion, and angiogenesis, which are hallmarks of cancer progression [8,9]. Over the past decade, high-throughput sequencing technologies have been extensively employed to identify genes that are differentially expressed in glioblastoma compared to normal brain tissue [10,11]. These differentially expressed genes provide valuable insights into the molecular mechanisms underlying glioblastoma pathogenesis, as they are involved in various biological processes such as cell cycle regulation, apoptosis, and immune response [12,13]. However, despite these advances, the precise genetic drivers of glioblastoma and their functional roles in tumor biology remain largely unknown, representing a critical gap in the literature that our study aims to address.

To bridge this gap, we employed a comprehensive screening approach utilizing multiple databases (Data sources can be accessed via the following links: https://portal.gdc.cancer.gov (accessed on 15 October 2025), https://www.gtexportal.org (accessed on 15 October 2025), http://www.ncbi.nlm.nih.gov/geo/ (accessed on 15 October 2025), http://www.cgga.org.cn (accessed on 15 October 2025)) to identify candidate genes associated with glioblastoma. Among the differentially expressed genes identified, the LYZ gene stood out due to its significant differential expression in glioblastoma tissues and its prognostic relevance. The LYZ gene, also known as lysozyme, is a well-characterized enzyme with antibacterial properties [14,15]. However, its role in cancer, particularly glioblastoma, has been relatively unexplored. The selection of the LYZ gene for further investigation was based on its potential to serve as a novel biomarker and therapeutic target, given its differential expression and prognostic significance in glioblastoma.

Our study aimed to investigate the expression pattern, clinical relevance, and molecular functions of the LYZ gene in glioblastoma. We conducted a series of analyses, including expression analysis across various databases, functional enrichment analysis, immune infiltration analysis, single-cell analysis, and experimental validation in glioblastoma cell lines. Additionally, to elucidate its functional involvement in tumor biology, we examined the effects of LYZ gene deletion on glioblastoma cell invasion, migration, and proliferation. By undertaking these comprehensive investigations, we aim to shed new light on the molecular pathways underlying glioblastoma pathogenesis and identify potential therapeutic targets for this aggressive cancer.

## 2. Materials and Methods

### 2.1. Data Collection and Preprocessing

We gathered information on normal tissue and glioblastoma (GBM) from multiple databases. For differential gene expression analysis, we initially screened clinical samples with available gene expression data from the Genotype-Tissue Expression (GTEx) and Cancer Genome Atlas (TCGA) databases. Specifically, the TCGA database contains nearly 1000 GBM cases; however, our analysis cohort was restricted to 153 clinical samples that met stringent inclusion criteria based on: (1) complete clinical annotation: survival time; and (2) availability of matched bio-specimen data: RNA sequencing quality metrics. This selection process ensured data homogeneity and analytical robustness. R software (version 4.2.1) was used to handle the raw sequencing data. For alignment, the reads were mapped to the GRCh38 (hg38) human reference genome built using STAR aligner (version 2.7.10a) with the following parameters: end-to-end alignment mode, allowing up to 2 mismatches per read, and a maximum intron length of 100,000 base pairs. After alignment, gene-level expression quantification was performed using featureCounts (version 2.0.3) with default settings. Differential expression analysis and normalization were conducted using the “limma” package (version 3.54.1), applying a threshold of log2 fold change > 1.5 and adjusted *p*-value < 0.05 (Benjamini–Hochberg correction). These detailed parameters ensure reproducibility and transparency of the analysis pipeline. To find genes with differential expressions, data from the Gene Expression Omnibus (GEO) database were also examined in a similar manner. The Chinese Glioma Genome Atlas (CGGA) was specifically chosen to confirm gene expression and clinical importance. It focuses on Chinese glioma patients, considering ethnic differences in glioma. Also, its multi-omics data enable in-depth analysis of glioma mechanisms, enhancing our study’s accuracy [16].

### 2.2. Gene Screening and Functional Enrichment

In the prognosis analysis, 890 genes were selected from TCGA. We downloaded clinical and gene expression data, divided patients into high/low-expression groups by median gene expression. Using Kaplan–Meier and log-rank tests, genes with *p* < 0.05 for survival differences were chosen. We used the intersection of differentially expressed genes from the TCGA and GEO datasets, taking into account their prognostic relevance, to find genes of interest. The “clusterProfiler” tool in R was used to pick the top 300 genes that had the highest correlation with the target gene for Gene Ontology (GO) enrichment analysis. To investigate possible biological pathways, signaling pathway analysis was carried out utilizing the Kyoto Encyclopedia of Genes and Genomes (KEGG) database.

### 2.3. Immune Infiltration Analysis

The CIBERSORT method was employed to quantify immune cell infiltration in glioblastoma multiforme (GBM). CIBERSORT is a computational approach that leverages gene expression signatures to deconvolute complex tissue samples into their constituent immune cell types and estimate their relative proportions. In our study, we first utilized this method to obtain the infiltration levels of various immune cell types within the GBM samples. Subsequently, we conducted a comprehensive examination of the relationships between the target gene and different immune cell types. This was achieved by correlating the expression levels of the target gene with the estimated proportions of each immune cell type. Moreover, to further investigate the potential impact of the target gene on immune cell distribution, we contrasted the immune cell enrichment scores across two distinct groups: one with high target gene expression and the other with low target gene expression [17,18].

### 2.4. Single-Cell Analysis

Seurat (version 4.0.4) in R was used to download and process single-cell RNA sequencing data (GSE163108_10x). This dataset (GSE163108_10x) was generated through, first, biological samples, such as tissues or cell suspensions, are collected from organisms. Then, these samples undergo cell dissociation processes to break down the tissue structure and obtain individual cells, often using enzymatic or mechanical methods. Next, single cells are isolated, which can be achieved through techniques like fluorescence-activated cell sorting (FACS) or microfluidic-based approaches to ensure each well or reaction chamber contains only one cell. After that, the genetic material (RNA or DNA) within each single cell is extracted and reverse-transcribed into cDNA (in the case of RNA sequencing). Subsequently, high-throughput sequencing technologies, such as next-generation sequencing (NGS), are employed to generate sequencing reads that represent the genetic information of each individual cell. Finally, these raw sequencing data are processed, including quality control, alignment to a reference genome, and quantification of gene expression levels, resulting in a comprehensive single-cell dataset. Cell populations were visualized using t-Distributed Stochastic Neighbor Embedding (tSNE) clustering, and the distribution of target gene expression across cells was examined [19].

### 2.5. Cell Culture

At 37 °C in an incubator with 5% CO_2_, human GBM cell lines (U87, A172) and normal glial cells were cultivated in Dulbecco’s Modified Eagle Medium (DMEM, manufactured by Gibco, Grand Island, NY, USA) supplemented with 10% fetal bovine serum, FBS (Umedium, Hefei, China), and 1% penicillin-streptomycin [20].

### 2.6. Gene Knockdown

Following the manufacturer’s instructions, Lipofectamine 3000 (Invitrogen, Carlsbad, CA, USA) was used to construct and transfect GBM cells with short hairpin RNA (shRNA) that targets the LYZ gene. Non-targeting shRNA-transfected cells were used as controls [21].

### 2.7. Quantitative Real-Time PCR (qRT-PCR)

The PrimeScript RT Reagent Kit (Takara, Otsu, Japan) was used to reverse transcribe the total RNA into cDNA after it had been isolated from cells using the TRIzol reagent (Invitrogen, Carlsbad, CA, USA). SYBR Green Master Mix (Takara, Otsu, Japan) was used for qRT-PCR using an ABI 7500 Real-Time PCR System (Applied Biosystems, Foster City, CA, USA). The 2^−ΔΔCt^ technique was utilized to determine relative gene expression, with β-actin serving as an internal reference gene [22].

### 2.8. Western Blot Analysis

RIPA lysis buffer (Beyotime, Shanghai, China) was used to extract the total protein from the cells, and the BCA Protein Assay Kit (Beyotime, Shanghai, China) was used to measure the amount of protein. SDS-PAGE was used to separate the proteins, which were then put onto PVDF membranes. Membranes were blocked using 5% non-fat milk, then incubated with primary antibodies against lyz (Proteintech, Rosemont, IL, USA, Catalog number: 84140-1-RR) and β-actin (Proteintech, Rosemont, IL, USA, Catalog number: 81115-1-RR) for a whole night at 4 °C. HRP-conjugated secondary antibodies (Proteintech, Rosemont, IL, USA, Catalog number: SA00001-4) were then incubated for an hour at room temperature. The ECL detection kit (Beyotime, Shanghai, China) was used to see the protein bands [23].

### 2.9. Cell Proliferation Assays

The CCK8 test, EdU staining, and colony formation assay were used to measure cell proliferation. Cells were seeded in 96-well plates for the CCK8 experiment, and they were incubated for two hours at the designated time points using the CCK8 reagent (Dojindo, Kumamoto, Japan). A microplate reader was used to measure absorbance at 450 nm. For the EdU staining, following the manufacturer’s instructions, cells were fixed, stained, and treated with EdU reagent (RiboBio, Guangzhou, China) at a concentration of 10 μM for two hours. The selection of this 10µM concentration was based on previous research, which demonstrated that this concentration provides optimal staining results with minimal cytotoxicity in similar cell types. Cells were cultivated for two weeks after being seeded in 6-well plates for the colony formation test. Methanol was used to fix the colonies, followed by crystal violet staining and counting [24].

### 2.10. Cell Migration and Invasion Assays

A scratch test was used to measure cell migration. In 6-well plates, cells were sown and allowed to grow until they reached confluence. A pipette tip was used to make a scratch, and at 0 and 24 h, cell migration was seen and captured on camera. A Transwell test was used to measure cell invasion. A Transwell insert covered with Matrigel (BD Biosciences, Franklin Lakes, NJ, USA) had its top chamber seeded with cells, and the lower chamber was filled with media containing 10% FBS. The Matrigel-invading cells were counted, stained, and fixed after a 24 h period [25].

### 2.11. Statistical Analysis

Data are shown as mean ± standard deviation (SD), and all experiments were run in triplicate. Prior to statistical analysis, the normality of the data was assessed using the Shapiro–Wilk test. Only after confirming that the data fit a normal distribution (or if the sample size was large enough to assume normality based on the central limit theorem, depending on your actual situation), GraphPad Prism 8.0 was used to conduct statistical analysis. Group differences were compared using either a one-way ANOVA or Student’s *t*-test, with *p* < 0.05 being statistically significant [26].

### 2.12. Experimental Design and Replication

To ensure the robustness and reproducibility of our findings, we carried out three independent experiments, each executed on different days using newly prepared reagents and materials to curtail batch-related variations. This rigorous experimental design, with multiple independent runs and technical repeats, not only minimized random errors but also bolstered the statistical power of our analyses, thereby facilitating the drawing of more reliable and widely applicable conclusions.

### 2.13. Imaging and Microscopy Details

In this study, all microscopic imaging was conducted using a Leica DM6 B microscope (Leica Microsystems, Wetzlar, Germany), renowned for its high-resolution and stable optical performance. Depending on the experimental needs and sample features, magnifications of 10× for general sample overviews, 40×, and 100× (oil immersion) for detailed cellular or sub-cellular structures were applied. For fluorescence imaging, an excitation filter with a wavelength range of 470–490 nm and an emission filter with a range of 510–550 nm were used to enhance contrast and reduce background noise. In bright-field imaging, the condenser and diaphragm were adjusted for optimal contrast and brightness, ensuring accurate and reliable microscopic data.

## 3. Results

### 3.1. Screening of the LYZ Gene in Glioblastoma

**Figure 1:**
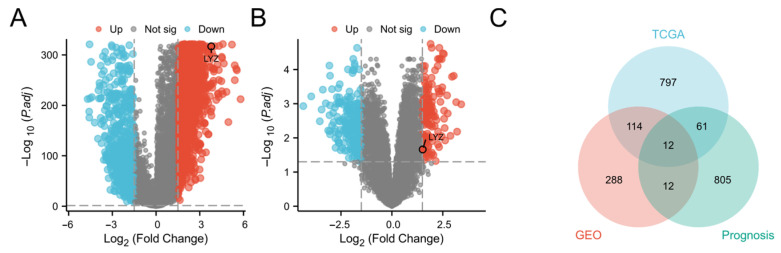
Screening of the LYZ gene in glioblastoma. (**A**) Differential expression genes between glioblastoma and normal samples in TCGA and GTEx databases. (**B**) Differential expression genes between glioblastoma and normal samples in GEO database. (**C**) The intersection of differentially expressed genes related to prognosis between TCGA and GEO databases.

We performed a thorough screening across many databases to find putative genes linked to glioblastoma (Log2 fold change > 1.5, *p* < 0.05). Using the TCGA and GTEx datasets, we first examined the genes that were expressed differently in glioblastoma and normal samples. When glioblastoma is compared to normal samples, red dots indicate up-regulated genes and blue points indicate down-regulated genes (Figure 1A). The complete list of all genes identified in Figure 1A is provided in Supplementary Table S1.

Furthermore, we used the GEO database to conduct a comparable study (Figure 1B). Similarly, the full list of all genes identified in Figure 1B can be found in Supplementary Table S2.

To select the LYZ gene as a gene of interest for additional research, we carried out the following steps. First, we calculated the intersection of the differential genes identified in the TCGA and GEO databases. Then, for the genes within this intersection set, we focused on their prognostic importance. Regarding the prognosis dataset in Figure 1C, we obtained patient survival information from the TCGA database, which includes detailed follow-up time and survival status (alive or deceased) for each glioblastoma patient. We then used established statistical methods, such as Kaplan–Meier survival analysis and Cox proportional hazards regression analysis, to evaluate the association between the expression levels of the genes in the intersection set and patient survival time. Genes that showed a significant correlation (*p*-value < 0.05 in the Cox regression analysis) with patient survival were considered to have prognostic importance. Based on these comprehensive analyses, we selected the LYZ gene for further study (Figure 1C).

### 3.2. Expression Level and Clinical Significance of the LYZ Gene in Glioblastoma

**Figure 2:**
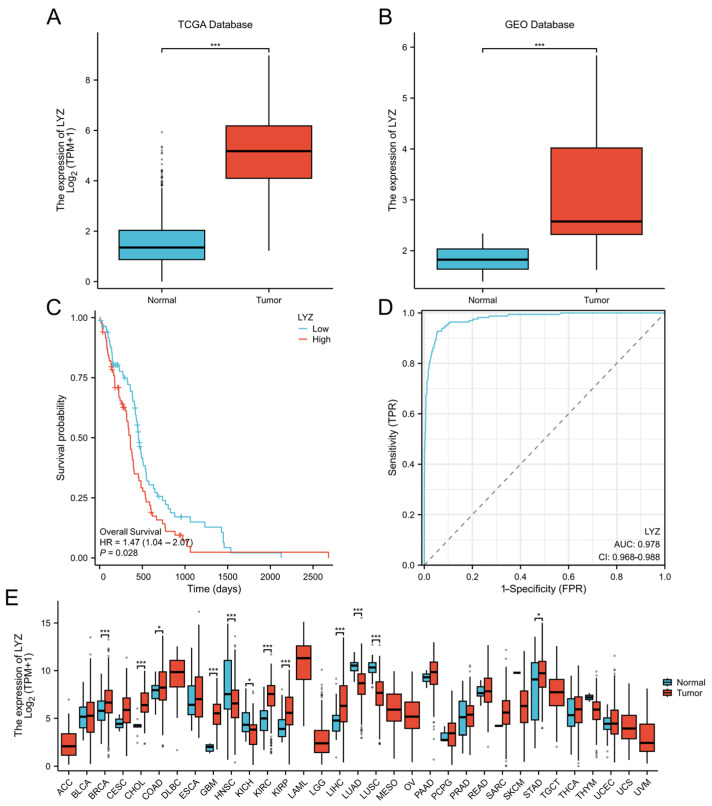
The expression level and clinical significance of LYZ gene in glioblastoma. (**A**) Differential expression of LYZ gene in tumor and normal tissues in TCGA database. (**B**) Differential expression of LYZ gene in tumor and normal tissues in GEO database. (**C**) Survival time differences between high and low LYZ gene expression groups. (**D**) Diagnostic efficacy of LYZ gene in patients with glioblastoma. (**E**) Differential expression of LYZ gene in pan cancer. * *p* < 0.05, *** *p* < 0.001.

We also investigated the LYZ gene’s expression pattern in glioblastoma. The LYZ gene was shown to be significantly expressed differently in tumor and normal tissues in the TCGA database, with greater expression levels in the tumor samples (Figure 2A). The GEO database study, which also revealed a significant overexpression of the LYZ gene in glioblastoma tissues relative to normal controls, further supported this finding (Figure 2B).

Based on the LYZ gene expression levels, we separated patients into groups with high and low expression to evaluate the clinical significance of the gene. Patients with high LYZ gene expression had a substantially shorter survival time than those with low expression (*p* < 0.05), according to survival analysis using the Kaplan–Meier technique and the Log-rank test (Figure 2C). Furthermore, receiver-operating characteristic (ROC) curve analysis was used to assess the LYZ gene’s diagnostic effectiveness in glioblastoma patients, suggesting that it may be used as a diagnostic biomarker (Figure 2D). The LYZ gene also showed differential expression in a number of other cancer types, according to a pan-cancer study, indicating that it contributes to the development and spread of malignancies (Figure 2E).

### 3.3. Functional Enrichment of the LYZ Gene in Glioblastoma

**Figure 3:**
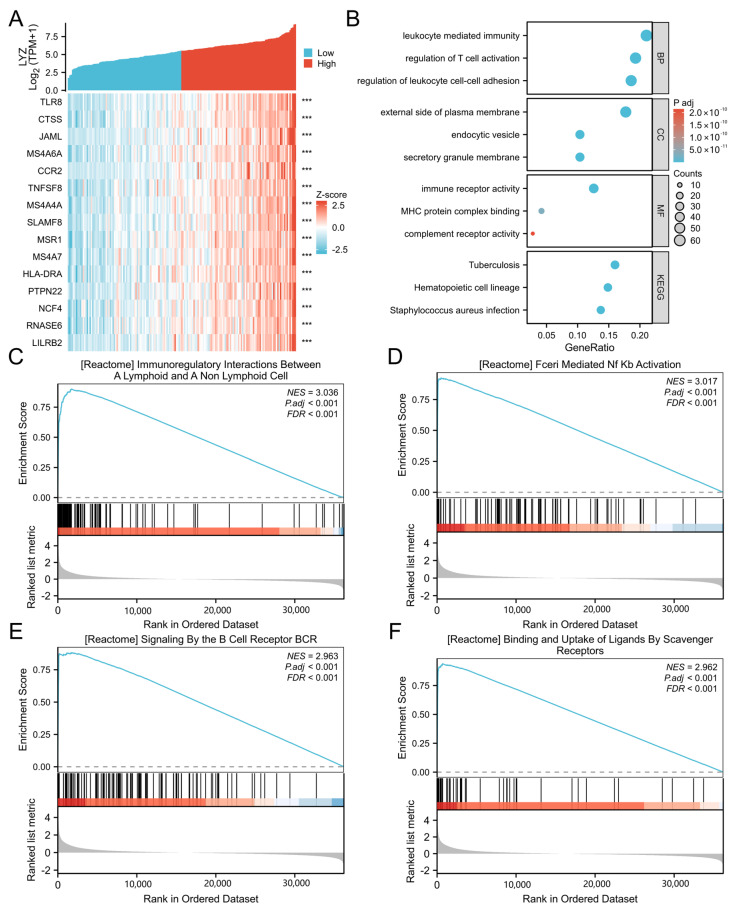
The functional enrichment of LYZ gene in glioblastoma. (**A**) The 15 genes with the highest correlation with differential expression of LYZ in glioblastoma. (**B**) The main biological processes and functions of LYZ gene in glioblastoma. (**C**–**F**) The related signaling pathway of LYZ gene in glioblastoma. *** *p* < 0.001.

We initially determined which 15 genes had the strongest link with the LYZ gene’s differential expression in order to comprehend the biological roles of the gene in glioblastoma (Figure 3A).

Regarding the cellular localization of LYZ, as depicted in Figure 3B, Gene Ontology enrichment analysis indicates that the LYZ gene is located on the outside of the plasma membrane (*p* < 0.05) and is implicated in leukocyte-mediated immunity. To further enhance the impact of this data, we have also included ICC images showing LYZ staining in the cell lines that are utilized later in the manuscript. These images provide direct visual evidence of LYZ’s localization on the cell surface (Supplementary Figure S1). This implies that via interacting with leukocytes on the cell surface, the LYZ gene may contribute to the immune response of glioblastoma, potentially affecting the tumor microenvironment and tumor-immune interactions.

Furthermore, the analysis of signaling pathways revealed that the LYZ gene is linked to several significant signaling pathways in glioblastoma, such as FceRI-mediated NF-kB activation and immunoregulatory interactions between lymphoid and non-lymphoid cells (*p* < 0.001) (Figure 3C–F).

### 3.4. Immune Infiltration Analysis of the LYZ Gene in Glioblastoma

**Figure 4:**
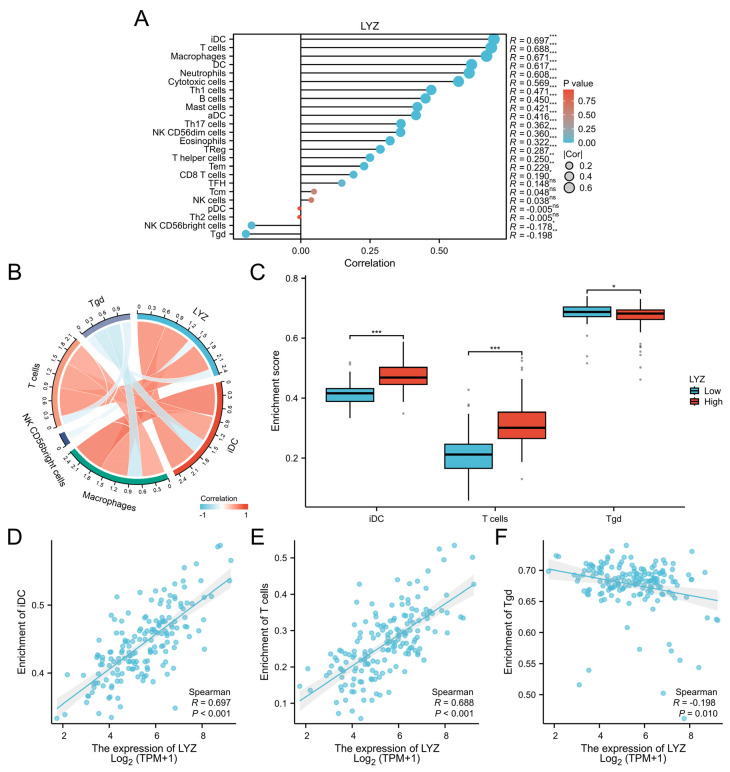
Immune infiltration analysis of LYZ gene in glioblastoma. (**A**) Immune cells associated with LYZ gene in glioblastoma. (**B**) Correlation analysis of major immune cells associated with LYZ gene in glioblastoma. (**C**) Enrichment scores of three immune cells between high and low expression groups of LYZ in glioblastoma. (**D**–**F**) The correlation between three types of immune cells and LYZ gene expression in glioblastoma. * *p* < 0.05, ** *p* < 0.01, *** *p* < 0.001. ns—not significant.

In the glioblastoma tumor microenvironment, immune infiltration is essential. In glioblastoma, we found immune cells linked to the LYZ gene (Figure 4A). Significant relationships between the LYZ gene and T cells, gamma delta T cells (Tgd), and immature dendritic cells (iDC) were revealed by correlation analysis (Figure 4B). Three immune cell types’ abundances varied between the groups with high and low LYZ gene expression, according to enrichment score analysis (Figure 4C). The strong link between these three immune cell types and the expression of the LYZ gene was verified by further correlation analysis (Figure 4D–F), indicating that the LYZ gene may have an impact on the immunological landscape of glioblastoma.

### 3.5. Verification of LYZ Gene Expression and Clinical Significance in the CGGA Database

**Figure 5:**
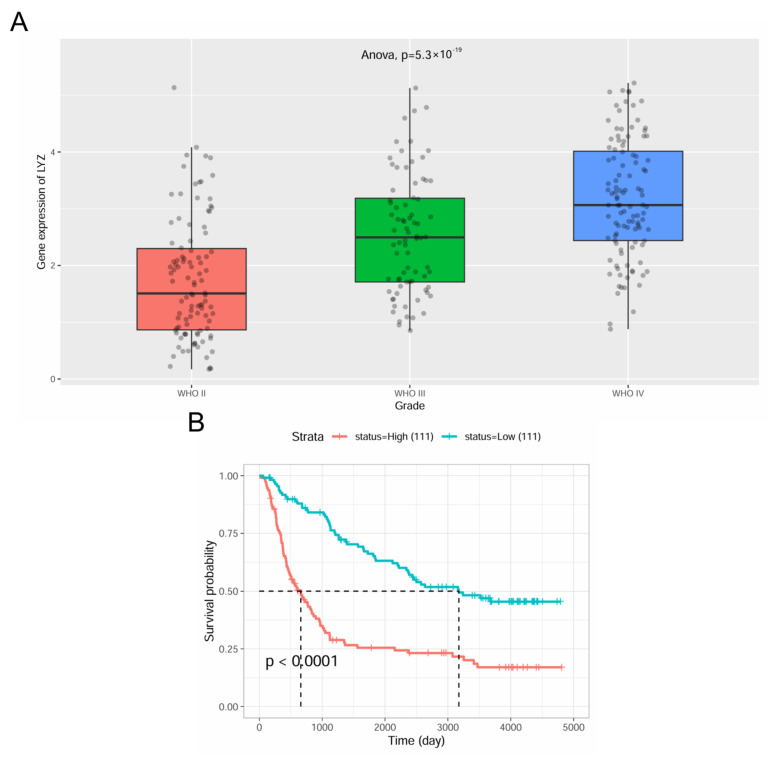
The verification of LYZ gene expression and clinical significance. (**A**) Differences in LYZ gene expression levels among different WHO stages. (**B**) Comparison of survival time with different expression levels of LYZ gene.

In the CGGA database, we also confirmed the LYZ gene’s expression and clinical relevance. Significant variations were seen in the levels of LYZ gene expression throughout the various WHO stages of glioblastoma, with greater expression in more advanced stages (Figure 5A). Consistent with our earlier findings, survival analysis based on varying levels of LYZ gene expression also shown that individuals with greater expression had a worse prognosis (Figure 5B).

### 3.6. Single-Cell Analysis of the LYZ Gene in Glioblastoma

**Figure 6:**
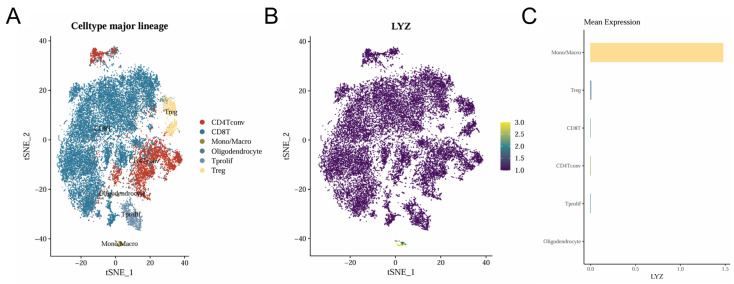
Single cell analysis of LYZ gene in glioblastoma. (**A**) The clustering of single-cell cells. (**B**) Distribution of LYZ gene expression in different cells. (**C**) Expression abundance of LYZ gene in different cells.

The location of the LYZ gene and the cellular heterogeneity of glioblastoma were both revealed by single-cell analysis. Different cell populations in glioblastoma (GSE163108_10x) were identified by tSNE clustering analysis of single-cell data (Figure 6A). Monocytes and macrophages were found to express the LYZ gene, according to the distribution of its expression across various cells (Figure 6B). The LYZ gene is mostly expressed in mononuclear macrophages, according to quantitative examination of its expression abundance in various cells (Figure 6C).

### 3.7. Experimental Verification of LYZ Gene Expression in Glioblastoma Cell Lines

We looked at the LYZ gene’s mRNA and protein levels in various glioblastoma cell lines to confirm its expression at the cellular level. For the normal astrocyte cell lines, we used the human normal astrocyte cell line HA1800, which was obtained from the American Type Culture Collection (ATCC, Manassas, VA, USA).

**Figure 7:**
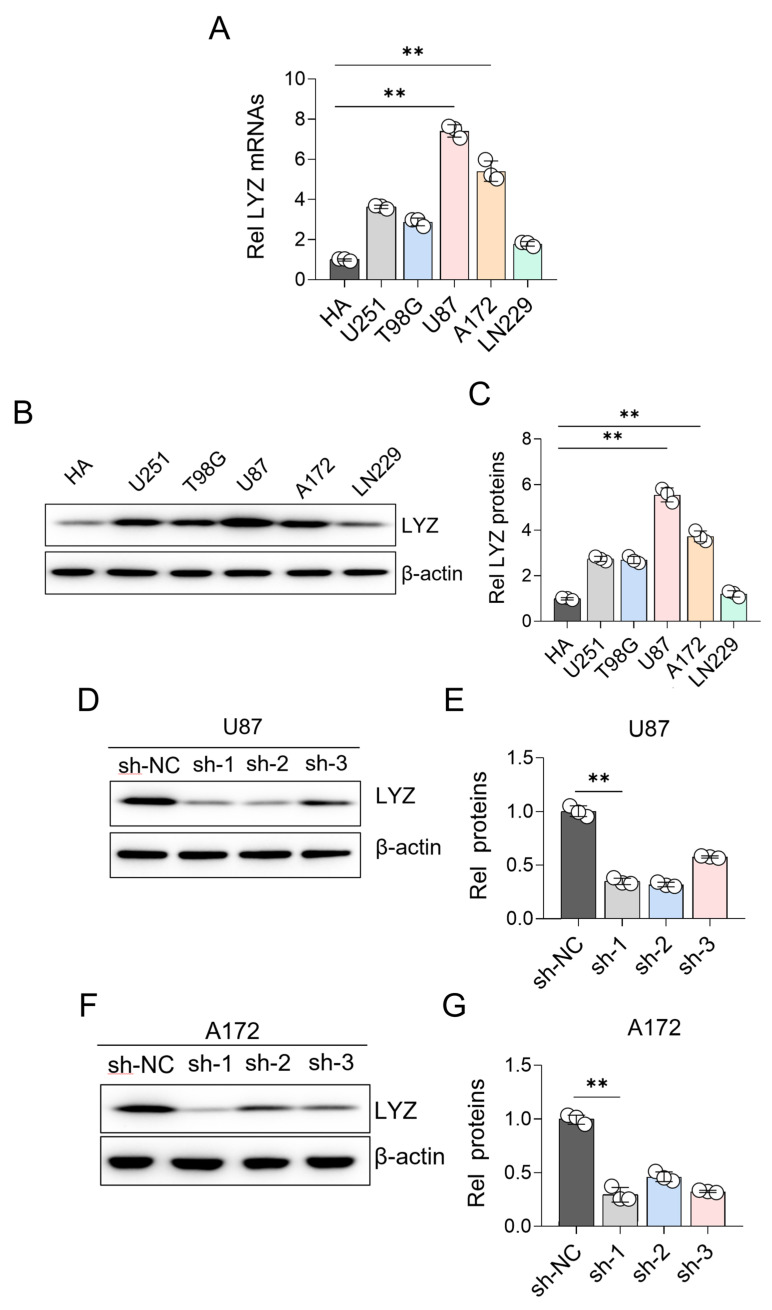
Further verification of LYZ gene expression in glioblastoma. (**A**) Differences in mRNA levels of LYZ gene in different glioblastoma cell lines. (**B**,**C**) Differences in protein levels of LYZ gene in different glioblastoma cell lines. (**D**,**E**) Differential protein expression of LYZ gene in U87 cell line. (**F**,**G**) Differential protein expression of LYZ gene in A172 cell line. The original protein images are shown in Supplementary Figure S2. ** *p* < 0.01.

For the qRT-PCR analysis, the PrimeScript RT Reagent Kit (Takara, Otsu, Japan) was used to reverse transcribe the total RNA, which had been isolated from cells using the TRIzol reagent (Invitrogen, Carlsbad, CA, USA), into cDNA. Subsequently, SYBR Green Master Mix (Takara, Otsu, Japan) was employed for qRT-PCR using an ABI 7500 Real-Time PCR System (Applied Biosystems, Foster City, CA, USA). The 2^−ΔΔCt^ technique was utilized to determine relative gene expression, with β-actin serving as an internal reference gene. This qRT-PCR analysis was carried out using three biological replicates for every cell line. The findings demonstrated that tumor cell lines and normal brain glial cells differed significantly in their LYZ gene mRNA levels (Figure 7A). These variations were validated at the protein level by Western blot analysis (Figure 7B,C).

After a preliminary screening of multiple GBM cell lines, we specifically chose to further examine the U87 and A172 cell lines. This selection was based on the fact that these two cell lines exhibited the most pronounced variation in LYZ gene expression among all the cell lines we tested. As shown in our results, there were clear differential expression patterns between U87 and A172 cell lines (Figure 7D–G), which made them ideal candidates for our in-depth study to explore the potential regulatory mechanisms and biological functions associated with the differential expression of the LYZ gene in GBM.

### 3.8. Effects of LYZ Gene Knockout on Glioblastoma Proliferation

**Figure 8:**
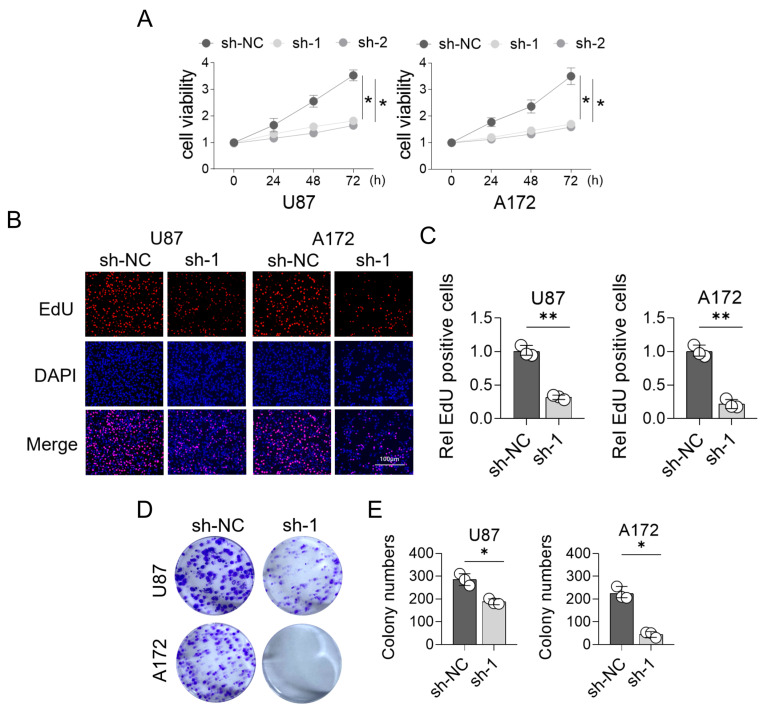
The proliferation of glioblastoma after knocking out the LYZ gene. (**A**) CCK8 experiment detects the proliferation ability of glioblastoma after knocking out the LYZ gene. (**B**,**C**) EdU method for detecting the proliferation ability of glioblastoma after knocking out the LYZ gene. (**D**,**E**) Cloning formation experiment to detect the proliferation ability of glioblastoma after knocking out the LYZ gene. * *p* < 0.05, ** *p* < 0.01.

In order to examine the LYZ gene’s functional significance in glioblastoma, we knocked it out using shRNA and evaluated how it affected cell growth. The CCK8 experiment showed that glioblastoma cell lines with LYZ gene knockout had significantly lower proliferation potential than glioblastoma cell lines without LYZ gene knockout (*p* < 0.05) (Figure 8A). Additionally, the LYZ-knockout group had a lower percentage of proliferating cells (*p* < 0.05) according to the EdU technique (Figure 8B,C). Additionally, the clone formation experiment showed that glioblastoma cells with LYZ gene deletion produced fewer and smaller colonies (*p* < 0.05) (Figure 8D,E), suggesting that the LYZ gene is necessary for glioblastoma cell proliferation.

### 3.9. Effects of LYZ Gene Knockout on Glioblastoma Migration and Invasion

**Figure 9:**
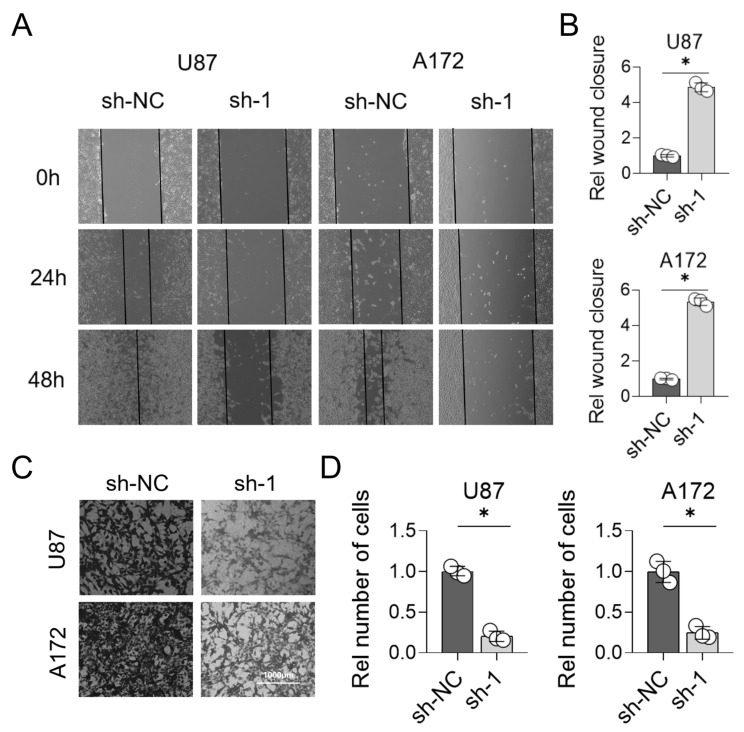
Detection of migration and invasion ability of glioblastoma after knocking out LYZ gene. (**A**,**B**) Scratch assay to detect the migration ability of glioblastoma after knocking out the LYZ gene. (**C**,**D**) Transwell method for detecting the invasive ability of glioblastoma after knocking out the LYZ gene. * *p* < 0.05.

We assessed how LYZ gene deletion affected glioblastoma cell migration and invasion in addition to proliferation. The scratch experiment revealed that LYZ gene deletion greatly reduced the capacity of glioblastoma cells to migrate (*p* < 0.05) (Figure 9A,B). To quantify cell infiltration, we defined the scratch boundaries as the cell-free area immediately adjacent to the initial wound edge (as shown in Figure 9A,B). Cells migrating into this boundary zone were counted as infiltrating cells. Exclusion criteria for data analysis included: (1) floating cells not firmly attached to the substrate; (2) abnormally shaped cells likely resulting from mechanical damage during scratch creation; and (3) cells overlapping with the original wound edge. As shown in the 24 h and 48 h time point panels, LYZ-knockout cells exhibited reduced scratch closure compared to controls, with fewer infiltrating cells within the defined boundaries. The LYZ gene may potentially contribute to glioblastoma cell migration and invasion, as evidenced by the Transwell technique, which showed a decreased invasive capacity of glioblastoma cells in the LYZ-knockout group (*p* < 0.05) (Figure 9C,D).

## 4. Discussion

According to our research, the LYZ gene is a potential diagnostic biomarker and therapeutic target for glioblastoma (GBM). The TCGA and GEO datasets show that LYZ is expressed differently in GBM tissues than in normal controls, which highlights its significance in the illness. The substantial overexpression of LYZ in GBM tissues and its correlation with shorter patient survival periods imply that LYZ is essential to the development of GBM. LYZ’s promise as a biomarker for early diagnosis and prognosis in GBM patients is further supported by its diagnostic effectiveness, as demonstrated by ROC curve analysis. This is consistent with earlier research that emphasized the significance of identifying certain gene expression patterns in cancer for both therapeutic and diagnostic purposes. A wider function for LYZ in carcinogenesis is suggested by the pan-cancer study showing differential expression of LYZ in several other cancer types, which calls for more research into its processes in diverse malignancies.

The LYZ gene, which is found on the outside of the plasma membrane and is implicated in leukocyte-mediated immunity, may play a part in immunological responses at the cell surface, according to functional enrichment analysis. This is especially important in GBM, because the tumor microenvironment is defined by intricate interactions between immune and tumor cells. In this context, monocytes/macrophages, as key components of the immune cell population within the GBM microenvironment, are of particular interest. Monocytes/macrophages can be polarized into different phenotypes, namely M1 (pro-inflammatory) and M2 (anti-inflammatory), which have distinct functions in tumor progression. Previous studies have shown that the polarization state of monocytes/macrophages in GBM is closely related to tumor growth, invasion, and immune evasion. Our immune infiltration analysis has revealed a strong association between LYZ expression and the presence of monocytes/macrophages in GBM tissues. It is plausible that LYZ may influence the polarization and function of monocytes/macrophages in the GBM microenvironment. For instance, LYZ might interact with specific receptors on the surface of monocytes/macrophages, triggering signaling pathways that lead to their polarization towards a particular phenotype. This, in turn, could affect the balance between pro- and anti-tumor immune responses, ultimately impacting the progression of GBM. Further research is needed to elucidate the precise mechanisms by which LYZ interacts with monocytes/macrophages and how this interaction contributes to the pathogenesis of GBM.

LYZ’s potential to alter the immunological landscape in GBM is further highlighted by its involvement with several immunoregulatory signaling pathways, including those involving interactions between lymphoid and non-lymphoid cells and NF-kB activation. Additional information was revealed by immune infiltration analysis, which revealed strong associations between LYZ expression and particular immune cell types, such as T cells and immature dendritic cells. Immune cell recruitment and activation within the GBM microenvironment may be influenced by LYZ, potentially impacting tumor-immune interactions and disease progression, as indicated by the variations in immune cell abundance between high and low LYZ expression groups. These results are in line with the increasing understanding of the significance of immune regulation in cancer treatment, especially in GBM, where conventional therapies have proven to be ineffective.

Recent studies have further enriched our understanding of GBM subtyping and immune microenvironment modulation, which are highly relevant to our findings on LYZ. They identified three integrative molecular subtypes (CS1, CS2, and CS3) of glioma through multi-omics data analysis. These subtypes exhibit distinct molecular and immunological features, with CS2 showing high immune infiltration and poor prognosis, while CS3 has an immunologically cold tumor microenvironment. This suggests that the impact of LYZ on immune cell polarization and function may vary across different glioma subtypes, potentially influencing their prognosis and treatment response [27]. Similarly, another group classified GBM patients into two disulfidptosis-related subtypes (DRGclusters) based on transcriptome data, revealing differences in tumor immune microenvironment and response to immunotherapy. This highlights the importance of considering novel cell death mechanisms and their associated subtypes when exploring the role of LYZ in GBM [28]. Moreover, the crucial role of tumor-associated macrophages (TAMs) has been emphasized, including microglia and bone marrow-derived macrophages, in GBM progression and treatment resistance. Their findings suggest that LYZ may interact with TAMs to modulate the immune microenvironment, which could be a key factor in determining the efficacy of immunotherapies in GBM [29]. Therefore, integrating our findings on LYZ with these recent studies could provide a more comprehensive understanding of GBM pathogenesis and guide the development of personalized treatment strategies.

The altered expression of LYZ in GBM cell lines as opposed to normal glial cells was verified experimentally at the cellular level. The substantial decrease in cell migration, invasion, and proliferation following LYZ gene deletion highlights the gene’s functional relevance in GBM pathogenesis. These findings imply that focusing on LYZ may be a practical tactic to stop the development and spread of GBM. Additionally, the experimental results relate LYZ expression to patient outcomes and offer a molecular explanation for the observed clinical relationships. The discovery of LYZ as a possible therapeutic target creates new opportunities for research and development given the poor efficacy of current GBM treatments. Future research should concentrate on clarifying the specific molecular pathways via which LYZ affects immune responses and GBM growth. To evaluate their effectiveness in preclinical and clinical contexts, the development of LYZ-targeted therapeutics, such as small molecule inhibitors or gene editing techniques, might also be investigated. By reprogramming myeloid cells and enhancing immune infiltration into the tumor microenvironment, the use of LYZ with current immunotherapeutic approaches, such as immune checkpoint inhibition, may also improve anticancer efficacy. All things considered, our research offers strong proof of LYZ’s involvement in GBM and lays the groundwork for future investigations into its potential for treatment.

## 5. Conclusions

This study presents compelling evidence that the LYZ gene represents a novel and promising therapeutic target and diagnostic biomarker for glioblastoma (GBM). Through comprehensive multi-omics analysis, we identified significant upregulation of LYZ in GBM tissues compared to normal controls, with a notable association with shorter patient survival periods. Functional enrichment analysis revealed LYZ’s localization on the plasma membrane and its involvement in leukocyte-mediated immunity, suggesting a role in cell surface immune responses. Immune infiltration analysis highlighted significant associations between LYZ expression and specific immune cell types, indicating its potential impact on the tumor microenvironment. Single-cell RNA sequencing confirmed LYZ expression in macrophages and monocytes within GBM, while cellular experiments demonstrated differential expression in GBM cell lines compared to normal glial cells. Notably, LYZ gene knockout resulted in a marked reduction in cell proliferation, motility, and invasion, underscoring its functional importance in GBM pathophysiology. These findings collectively support the potential of LYZ as a viable therapeutic target and diagnostic biomarker, warranting further research into its mechanisms of action and clinical applications to improve GBM management.

## Data Availability

Data can be requested from the corresponding author.

## Supplementary Materials

The following supporting information can be downloaded at https://www.mdpi.com/article/10.3390/biology15010009/s1: Table S1: Genes from TCGA and GTEx with differential expression in glioblastoma vs. normal samples. Lists all genes found to have different expression levels between glioblastoma and normal samples using TCGA and GTEx data. Corresponds to red (up-regulated) and blue (down-regulated) dots in Figure 1A. Table S2: Genes from GEO with differential expression in glioblastoma vs. normal samples. Shows all genes with differential expression between glioblastoma and normal samples based on GEO database analysis. Matches the red and blue dots in Figure 1B. Figure S1: Subcellular localization of LYZ protein in glioblastoma cells as determined by immunofluorescence staining. Figure S2: Original Western Blot images for the further verification of LYZ gene expression in glioblastoma.
