# Supplementary material for: LYZ Gene as a Novel Therapeutic Target and Diagnostic Biomarker in Glioblastoma: Insights from Multi-Omics Analysis and Functional Validation
Source: Biology (Basel). 2025 Dec 19;15(1):9. doi: 10.3390/biology15010009 (PMC12784846; doi:10.3390/biology15010009)
Supplement: Supplementary file 1 [file biology-15-00009-s001.zip › Supplementary Files/Table S1.pdf]

gene	logFC	padj
A1BG	-1.826768067	1.37E-220
A1CF	-0.00789936	0.000108569
A2M	3.169129241	8.46828516971897e-321
A2ML1	0.5490785	2.12E-31
A3GALT2	0.440795832	4.05E-72
A4GALT	0.742805035	9.93E-41
A4GNT	0.065993093	2.36E-06
AAAS	0.754969747	1.83E-31
AACS	-0.718145538	3.57E-17
AADAC	0.007569482	0.513757782
AADACL2	-0.007165886	0.023622868
AADACL3	0.003249366	0.002112399
AADACL4	-0.002565785	0.316923999
AADAT	1.13216535	1.79E-52
AAGAB	1.585777077	3.68E-123
AAK1	-0.542303317	5.02E-09
AAMDC	1.037859018	3.14E-107
AAMP	1.182532873	4.13E-70
AANAT	0.568451821	6.46E-74
AARD	-0.461714205	1.92E-24
AARS2	0.893757809	1.91E-33
AASDH	1.172655259	2.85E-67
AASDHPPT	-0.169724776	0.034285246
AASS	2.317765963	3.17E-205
AATF	0.949022739	2.84E-60
AATK	-3.033502803	7.03E-212
ABAT	0.262881395	0.000111191
ABCA1	2.925847346	3.97E-282
ABCA10	-0.76147115	4.60E-27
ABCA12	0.35212123	6.43E-274
ABCA13	0.537486376	3.81E-263
ABCA2	-1.357212544	9.64E-48
ABCA3	0.753172624	2.38E-20
ABCA4	0.135134053	1.10E-20
ABCA5	-0.970550036	1.71E-18
ABCA6	-0.331364104	6.45E-06
ABCA7	-0.733383397	4.21E-13
ABCA8	0.153299521	0.131950122
ABCA9	-1.175666365	3.03E-80
ABCB1	0.060510431	0.371880487
ABCB10	1.128161245	1.66E-51
ABCB11	0.126345759	7.75E-50
ABCB5	0.067883953	7.09E-135
ABCB7	1.60915268	3.16E-147
ABCB8	0.797593829	4.28E-27
ABCB9	-0.32583732	2.34E-06
ABCC1	1.39315173	5.98E-88

ABCC10	0.286401723	0.000872221
ABCC11	0.021395894	0.299965338
ABCC12	-0.315919477	2.84E-09
ABCC2	0.058460238	0.025747915
ABCC4	2.192577781	6.35314834093082e-314
ABCC5	-0.483677999	2.52E-06
ABCC6	0.569936648	6.09E-160
ABCC8	-2.550610848	7.24E-81
ABCC9	0.703978506	4.44E-38
ABCD2	0.704874183	1.22E-20
ABCD4	0.376400947	7.10E-08
ABCE1	1.169845009	1.51E-75
ABCF1	1.894605148	1.79E-160
ABCF2	0.235694576	0.00020853
ABCF3	0.397305326	6.31E-07
ABCG1	1.182658428	9.26E-81
ABCG2	1.002824648	3.49E-43
ABCG4	-2.089176285	1.26E-62
ABCG5	-0.024540205	0.067328541
ABCG8	-0.301131918	3.37E-32
ABHD1	-0.430304212	9.61E-34
ABHD10	0.936457326	7.34E-53
ABHD11	1.337168294	2.50E-112
ABHD12	0.610412446	1.35E-22
ABHD12B	-0.372124463	8.62E-08
ABHD13	1.52677776	5.21E-154
ABHD14A	-1.793891759	2.22E-98
ABHD14B	1.29779919	9.54E-98
ABHD17A	-1.133871933	2.32E-88
ABHD17B	0.787130264	9.41E-31
ABHD17C	1.601747076	1.38E-181
ABHD18	0.708220636	6.89E-30
ABHD2	1.466725651	1.18E-89
ABHD3	1.017050806	7.53E-56
ABHD5	1.841863469	2.33E-185
ABHD6	0.810097334	6.27E-28
ABHD8	-0.458865458	2.37E-07
ABI1	0.900273931	1.08E-56
ABI2	0.07222838	0.264631714
ABI3BP	0.980519564	1.11E-38
ABL1	1.619028173	1.10E-201
ABL2	0.344015862	6.84E-08
ABLIM1	-0.640435393	1.41E-07
ABLIM2	-1.573304832	4.67E-54
ABLIM3	-0.751795953	2.10E-14
ABO	-0.475054757	5.03E-40
ABR	-0.217111004	0.004095158
ABRA	-0.156440749	1.43E-07

ABRACL	2.381726589	5.20E-143
ABT1	2.027851117	4.54E-207
ABTB1	-1.405424914	1.45E-39
ABTB2	0.433064271	5.81E-09
ACAA1	-0.090368385	0.107941059
ACAA2	0.742435319	7.44E-34
ACACA	0.5975946	3.78E-20
ACACB	-0.538424867	5.65E-23
ACAD10	0.801909577	2.70E-63
ACAD8	0.834897662	6.76E-34
ACAD9	1.008613425	1.54E-55
ACADL	-0.049003162	0.348498462
ACADM	0.859970287	1.49E-61
ACADS	0.981604785	1.07E-82
ACADSB	-0.300507536	9.92E-05
ACADVL	0.156666797	0.013388921
ACAN	1.101686979	4.32E-181
ACAP1	0.173653884	0.000452434
ACAP2	0.858422963	2.59E-33
ACAP3	-1.267402395	1.79E-29
ACAT1	0.851667512	8.19E-70
ACAT2	1.18578311	8.62E-47
ACBD3	1.732111096	1.28E-164
ACBD4	0.062632146	0.339400672
ACBD5	-0.649967752	5.76E-44
ACBD6	1.19089376	1.19E-63
ACBD7	-0.700183719	2.59E-13
ACCS	0.6652155	2.43E-32
ACCSL	0.014940642	0.08509194
ACD	0.512535808	1.22E-08
ACE	1.185771228	2.48E-50
ACE2	-0.12252052	1.14E-16
ACER1	0.067624031	1.99E-07
ACER2	0.267300064	5.35E-07
ACER3	1.459955429	6.90E-126
ACHE	-0.432531626	0.000500171
ACIN1	0.63910752	9.45E-19
ACKR1	-0.623538303	3.61E-05
ACKR2	0.371277068	4.73E-81
ACKR4	0.403706414	7.94E-113
ACLY	2.378092262	1.70E-187
ACMSD	0.371652073	1.01E-157
ACO1	1.291848625	6.11E-102
ACO2	-1.37035599	3.37E-75
ACOD1	0.025235523	0.001386847
ACOT1	1.272531181	4.11E-85
ACOT11	0.238593583	5.80E-10
ACOT12	0.449205971	1.45E-163

ACOT13	0.989317147	4.38E-99
ACOT2	1.521901064	3.82E-164
ACOT4	0.027619136	0.611991703
ACOT6	-0.298617658	8.00E-21
ACOT7	-0.143015947	0.181052014
ACOT8	-0.063888087	0.30696888
ACOT9	2.05118611	7.06E-296
ACOX1	1.535344673	2.94E-214
ACOX2	1.276162685	7.49E-150
ACOX3	1.70413787	1.83E-273
ACP1	1.050238075	9.40E-57
ACP2	1.831256789	2.40E-143
ACP6	1.077691108	1.54E-138
ACP7	-1.226345136	1.34E-25
ACR	-0.286366743	9.77E-20
ACRBP	0.908919624	1.24E-32
ACRV1	0.338944401	1.23E-17
ACSBG1	-0.781423497	1.23E-18
ACSBG2	0.140127216	2.19E-06
ACSF2	0.550816636	5.91E-16
ACSF3	0.095482086	0.115841326
ACSL1	0.861196122	3.73E-26
ACSL3	1.053116192	1.49E-59
ACSL4	0.86062819	7.56E-31
ACSL5	0.462629639	2.36E-10
ACSL6	-2.203949646	1.72E-112
ACSM1	-0.210046428	3.74E-15
ACSM2A	-0.00205202	0.397824709
ACSM2B	-0.002425823	0.327322277
ACSM3	0.296489033	2.33E-191
ACSM4	0.226150408	2.60E-209
ACSM5	0.750541807	2.33E-33
ACSM6	0.020011398	6.32E-08
ACSS1	0.01867014	0.799770766
ACSS2	0.724679247	1.23E-27
ACSS3	1.031653025	4.45E-83
ACTA1	-1.139643717	1.13E-32
ACTA2	1.72251898	3.44E-85
ACTB	2.314348508	5.28E-255
ACTBL2	0.018849664	2.71E-18
ACTC1	0.172217877	0.001419328
ACTG2	1.464932097	1.78E-97
ACTL10	-0.318067507	3.49E-81
ACTL6B	-2.047446539	1.64E-43
ACTL7A	-0.023652994	0.000872567
ACTL7B	0.018774668	0.019572061
ACTL8	0.108574665	2.43E-31
ACTL9	-0.011634345	0.004751386

ACTN1	2.015180334	2.99E-97
ACTN2	-0.100926218	0.389248816
ACTN3	-0.037905008	0.091728142
ACTN4	1.611921652	3.68E-137
ACTR10	0.68125013	7.29E-21
ACTR1A	0.400528035	1.10E-10
ACTR1B	0.54286728	1.18E-11
ACTR2	1.624672526	2.13E-104
ACTR3	1.633630587	3.11E-156
ACTR3B	-1.139270751	5.95E-34
ACTR3C	-0.08954197	5.09E-05
ACTR5	1.239004409	7.81E-86
ACTR6	0.899886951	3.02E-51
ACTR8	1.776027891	5.48E-233
ACTRT1	-0.012653959	0.001909534
ACTRT2	-0.022595297	2.43E-05
ACTRT3	-0.077043073	0.195229948
ACVR1	1.855964469	3.09E-305
ACVR1B	0.887983169	9.20E-32
ACVR1C	-0.975037299	3.07E-41
ACVR2A	0.679487577	4.37E-19
ACVR2B	-0.063787316	0.366687808
ACVRL1	1.633307752	1.91E-141
ACY3	1.033521861	5.64E-29
ACYP1	0.136989198	0.040611078
ACYP2	0.893418015	3.14E-66
ADA	1.552023946	1.95E-140
ADAD1	-0.003833392	0.089171737
ADAD2	-0.392199197	6.27E-09
ADAL	0.93264714	5.56E-82
ADAM10	1.225729363	8.07E-69
ADAM11	-1.976206448	1.84E-31
ADAM15	0.762953623	2.75E-26
ADAM17	0.99722301	4.28E-44
ADAM18	0.00226938	0.054879974
ADAM19	1.61310095	1.15E-66
ADAM2	-0.017822846	0.020119948
ADAM20	-0.10412541	1.36E-05
ADAM21	0.409855785	7.26E-49
ADAM22	0.081882625	0.490868246
ADAM23	0.518544383	1.89E-06
ADAM28	1.802506284	5.26E-130
ADAM29	0.013073281	0.098138472
ADAM30	0.001560116	0.357934211
ADAM32	-0.203400207	3.72E-10
ADAM33	0.49362471	2.09E-13
ADAM7	0.011261906	2.71E-19
ADAM8	1.151033605	9.30E-75

ADAM9	1.801808162	5.23E-122
ADAMTS1	0.314190556	0.001057884
ADAMTS10	0.724731058	2.79E-20
ADAMTS12	1.334138757	3.25E-266
ADAMTS13	-0.431652498	9.49E-09
ADAMTS14	0.786704668	1.58E-22
ADAMTS16	-0.32817918	0.004672327
ADAMTS17	0.014283472	0.696919602
ADAMTS18	-0.281354125	0.04760046
ADAMTS19	-0.350942016	4.21E-12
ADAMTS2	0.957309421	1.93E-56
ADAMTS20	0.119625305	4.96E-58
ADAMTS3	1.382276691	2.83E-103
ADAMTS4	1.097793655	1.98E-24
ADAMTS5	0.852381631	2.10E-27
ADAMTS7	1.362956342	1.18E-266
ADAMTS8	-0.086991058	0.328479348
ADAMTS9	2.13178159	1.49E-116
ADAMTSL1	0.752041604	1.66E-62
ADAMTSL2	0.62474955	9.49E-21
ADAMTSL3	-0.047472602	0.209774551
ADAMTSL4	1.191082284	3.03E-95
ADAMTSL5	-0.402879026	4.02E-35
ADAR	1.045367305	4.66E-49
ADARB1	-0.817879617	7.30E-13
ADARB2	-1.955659821	2.23E-154
ADAT1	1.980912889	2.89E-275
ADAT2	0.393531684	9.29E-10
ADAT3	0.300657117	1.23E-05
ADCK1	0.248010067	8.50E-06
ADCK2	1.539778824	4.71E-120
ADCK5	0.236060886	0.003903725
ADCY1	-1.322765122	1.04E-21
ADCY10	-0.042835235	0.082749652
ADCY2	-0.488997992	7.51E-10
ADCY3	0.301823597	0.000204948
ADCY4	-0.35956152	1.57E-10
ADCY5	-1.821576803	4.56E-61
ADCY6	0.427088069	2.02E-06
ADCY7	0.970193287	1.51E-55
ADCY8	1.790631086	2.25E-157
ADCY9	0.347430371	1.69E-06
ADCYAP1	-0.747150411	6.31E-10
ADCYAP1R1	1.44041905	9.39E-83
ADD1	-0.221738776	0.000164696
ADD2	0.220685346	0.085703986
ADD3	0.24522786	4.15E-06
ADGB	-0.076826682	0.058144912

ADGRA1	0.087686567	0.364887466
ADGRA2	0.72583689	2.82E-29
ADGRA3	1.235135799	2.92E-78
ADGRB1	-0.368124041	7.44E-05
ADGRB2	0.441225339	2.95E-06
ADGRB3	-0.088916908	0.337309124
ADGRD1	-0.070917822	0.064101468
ADGRD2	0.162145706	0.000195827
ADGRE2	1.549807056	1.27E-212
ADGRE3	0.405900662	8.54E-213
ADGRF1	0.003381708	0.113887876
ADGRF2	-0.02881666	0.002169706
ADGRF3	0.03018521	0.378170486
ADGRF4	-0.104255962	0.002548642
ADGRF5	1.025945247	1.57E-59
ADGRG1	2.832131396	9.71E-246
ADGRG2	0.226530902	4.16E-20
ADGRG3	0.492151447	2.19E-35
ADGRG4	0.002335156	0.441307458
ADGRG5	0.666490566	1.10E-140
ADGRG6	0.77693991	4.71E-79
ADGRG7	0.51613778	1.00E-56
ADGRL1	-0.2201545	0.013485085
ADGRL2	0.452672387	2.83E-09
ADGRL3	1.035884323	1.73E-80
ADGRL4	1.75684978	5.68E-152
ADGRV1	-0.191845847	0.067312996
ADH1A	0.008346754	0.214634625
ADH1B	-0.913342083	1.10E-62
ADH1C	0.050061259	0.041478766
ADH4	0.114224715	1.20E-28
ADH5	1.925339966	4.79E-264
ADH7	0.101458499	5.78E-61
ADHFE1	-1.711004315	8.88E-191
ADI1	1.432944098	1.12E-180
ADIG	0.777124676	1.42E-227
ADIPOQ	-0.138354889	1.75E-13
ADIPOR1	1.283874514	1.73E-115
ADIPOR2	0.877561112	6.18E-37
ADIRF	-0.306296242	1.30E-17
ADK	1.189757404	9.70E-104
ADM	3.12487455	9.07E-160
ADM2	0.895249048	1.43E-306
ADM5	-0.331933674	7.37E-08
ADNP	1.51922774	2.00E-106
ADNP2	1.906603048	1.15E-239
ADO	1.049979829	6.12E-45
ADORA1	1.174865737	1.38E-77

ADORA2A	-3.002432096	8.97E-116
ADORA2B	0.720519359	2.97E-22
ADPGK	1.053179385	1.33E-62
ADPRHL1	-0.001092195	0.982623501
ADPRM	1.24057848	3.08E-135
ADRA1A	0.106217853	0.008983592
ADRA1B	-0.005286585	0.96291796
ADRA1D	0.969847952	3.30E-32
ADRA2A	-0.363531235	0.000420516
ADRA2B	-0.066924078	0.271605059
ADRA2C	-1.763143386	1.47E-65
ADRB1	-0.345716264	0.000194419
ADRB2	1.39877728	3.82E-220
ADRB3	-0.003167215	0.778911817
ADRM1	0.952335497	5.11E-40
ADSL	-1.041435375	2.57E-77
ADTRP	-0.020311323	0.714772931
AEBP1	3.96504728	1.11E-267
AEBP2	1.138173868	9.34E-113
AEN	2.124977442	2.04E-266
AFAP1	-0.821089219	7.85E-40
AFAP1L2	0.320338008	1.96E-05
AFDN	0.434061504	9.94E-11
AFF1	1.218963511	6.80E-132
AFF2	-0.714203231	1.67E-14
AFF3	-0.604942119	1.54E-10
AFF4	1.101294211	1.04E-51
AFG3L2	-0.071341542	0.38225626
AFM	-0.006535679	0.077773883
AFMID	0.287829662	0.00086162
AFP	0.402092595	1.55E-150
AFTPH	0.380378554	5.61E-07
AGA	2.201319508	5.51E-222
AGAP1	-0.069023418	0.284111253
AGAP2	-1.881959007	1.08E-38
AGAP3	-0.803326578	4.68E-23
AGAP4	-0.519998467	2.08E-10
AGAP5	-1.521564343	7.40E-137
AGAP6	-3.033646625	4.28E-222
AGAP9	-0.81535881	3.73E-20
AGBL1	-0.282162654	5.39E-12
AGBL2	0.457939699	1.83E-13
AGBL4	-0.027440062	0.634695939
AGBL5	1.533187765	2.39E-181
AGER	0.704062023	8.04E-16
AGFG1	1.583585236	7.06E-156
AGFG2	1.193361224	1.05E-86
AGGF1	0.497078213	1.01E-17

AGK	0.094918861	0.220500184
AGL	0.899149068	9.36E-44
AGMAT	0.864927144	2.17E-65
AGO1	0.754185616	1.38E-44
AGO2	0.691593751	1.05E-26
AGO3	0.402661358	2.52E-12
AGO4	0.544233343	2.97E-17
AGPAT1	0.319564459	6.64E-10
AGPAT2	1.47953102	1.78E-117
AGPAT3	0.267968488	7.16E-08
AGPAT4	-0.495194493	4.50E-09
AGPS	1.559693971	6.22E-144
AGR2	-0.007190007	0.585222889
AGR3	0.615613886	4.84E-57
AGRN	2.11478546	1.87E-136
AGRP	0.302956208	3.31E-06
AGT	2.170664545	3.62E-135
AGTPBP1	-0.007895341	0.923914975
AGTR1	0.558801785	9.65E-34
AGTR2	-0.028877733	0.10836514
AGXT	0.168331535	3.54E-25
AGXT2	0.015051766	8.15E-06
AHCTF1	0.940550997	3.26E-44
AHCY	2.28114341	5.98E-296
AHCYL1	1.082581635	3.02E-55
AHCYL2	1.255975715	4.81E-93
AHDC1	0.816994429	4.01E-18
AHI1	-0.495973863	8.69E-07
AHNAK	0.395925306	3.63E-10
AHNAK2	0.084666213	0.361014993
AHR	2.14313715	1.94E-293
AHSA1	0.718347739	6.59E-25
AHSG	0.225184325	1.97E-36
AHSP	0.187692597	4.71E-07
AICDA	0.019666924	6.44E-15
AIDA	1.700804491	1.44E-190
AIF1	3.403779829	5.79E-149
AIF1L	0.646259814	1.72E-13
AIFM1	-0.347674117	1.02E-09
AIFM2	0.295727804	2.10E-07
AIG1	1.079696662	4.61E-94
AIM2	1.201295949	1.26E-238
AIMP1	0.395274392	3.15E-14
AIP	1.021491123	2.81E-72
AIPL1	-0.014583342	0.091846762
AIRE	-0.0946311	0.130427968
AJAP1	-1.425587062	8.06E-77
AK2	1.57969312	2.23E-144

AK3	1.222621652	7.11E-120
AK4	1.26259653	5.15E-94
AK5	-2.006916157	7.19E-46
AK6	0.703678331	1.13E-40
AK7	-0.165926839	0.016931927
AK8	1.307911392	3.33E-149
AK9	0.453113366	1.59E-26
AKAIN1	-1.514190642	3.08E-20
AKAP1	0.570425091	6.76E-17
AKAP10	1.092442692	2.26E-76
AKAP11	0.031555947	0.695671705
AKAP12	0.635062719	1.07E-09
AKAP13	1.667938039	9.38E-160
AKAP14	0.293108826	2.63E-07
AKAP17A	0.803746827	4.57E-29
AKAP3	0.90243023	5.47E-111
AKAP4	-0.010709632	0.033564507
AKAP5	-0.835554101	1.12E-13
AKAP6	-0.304211612	7.73E-06
AKAP7	0.949202483	4.37E-72
AKAP8	1.210643614	2.81E-63
AKAP8L	-1.067574575	4.91E-36
AKAP9	0.15305217	0.06069469
AKIP1	1.61670197	3.61E-229
AKIRIN1	1.82475631	3.27E-187
AKIRIN2	1.826349596	3.83E-150
AKNA	1.310790203	1.51E-121
AKR1A1	2.396838824	3.84E-285
AKR1B1	1.454888978	1.66E-106
AKR1B10	0.629657139	1.45E-202
AKR1B15	0.396783414	3.72E-293
AKR1C1	-0.308913249	7.15E-09
AKR1C2	-0.633529284	3.79E-31
AKR1C3	0.206283577	0.001908437
AKR1C4	0.032584716	4.71E-11
AKR1C8P	0.00463074	0.005274094
AKR1D1	0.061622587	5.34E-136
AKR1E2	-0.145229887	7.49E-07
AKR7A2	1.606401923	7.37E-256
AKR7A3	0.14676926	0.001839782
AKT1	0.541418792	2.10E-21
AKT1S1	1.500029505	8.63E-226
AKT2	0.662673076	7.86E-14
AKT3	-0.212349648	0.005793788
AKTIP	-0.31265544	2.61E-09
ALAD	-0.117725149	0.038414543
ALAS1	1.63353249	1.77E-62
ALAS2	0.224268153	3.69E-05

ALB	-0.861487707	1.20E-43
ALCAM	0.885853516	1.85E-17
ALDH16A1	1.105492925	1.95E-65
ALDH18A1	2.007751714	1.16E-260
ALDH1A1	-0.830897959	8.81E-11
ALDH1A2	0.514943414	1.27E-32
ALDH1A3	-0.628890851	8.03E-22
ALDH1L1	-0.052030132	0.566485885
ALDH1L2	0.570615366	1.09E-12
ALDH2	-0.726752507	1.09E-31
ALDH3A1	0.710362339	3.90E-78
ALDH3A2	0.230369733	0.000370117
ALDH3B1	1.640101646	2.02E-153
ALDH3B2	-0.056707015	3.09E-08
ALDH4A1	1.209921089	1.60E-44
ALDH5A1	-0.559701139	1.22E-20
ALDH6A1	-0.244131566	0.000232279
ALDH7A1	0.184686487	0.00628671
ALDH8A1	-0.998598479	2.95E-46
ALDH9A1	1.710396283	2.67E-201
ALDOB	-0.401699382	1.03E-24
ALDOC	-0.729213259	9.58E-22
ALG1	1.194546871	1.76E-97
ALG10	0.918482172	8.91E-152
ALG10B	1.121516993	1.19E-161
ALG11	0.340697194	6.65E-30
ALG12	0.570050845	1.46E-22
ALG13	1.170182251	1.46E-100
ALG14	1.452223487	5.53353523342196e-322
ALG1L2	0.589797843	1.05E-158
ALG2	1.951226672	3.80E-199
ALG3	2.046593975	1.59E-286
ALG6	1.927930369	7.32E-254
ALG8	2.141776844	3.46E-306
ALG9	0.099778011	0.073859764
ALK	0.990015529	2.26E-95
ALKAL1	-0.039493104	0.000647669
ALKAL2	-0.215116129	0.011324163
ALKBH1	1.737488681	1.93E-200
ALKBH2	2.114821079	6.57020847480836e-318
ALKBH3	1.421544833	2.20E-110
ALKBH4	1.388642241	2.80E-86
ALKBH5	0.635542112	3.25E-25
ALKBH7	1.762504838	5.25E-186
ALKBH8	1.194777542	1.03E-120
ALLC	-0.164602027	1.38E-06
ALMS1	0.635838547	2.02E-28
ALOX12	0.96194268	1.92E-195

ALOX12B	-0.621178813	1.84E-48
ALOX15	-0.230261425	2.36E-09
ALOX15B	1.666812275	1.01E-138
ALOX5	2.046733253	1.12E-52
ALOXE3	-0.671503061	7.06E-22
ALPI	-0.005790319	0.350302999
ALPK1	0.821207033	4.53E-47
ALPK2	0.727287996	1.90E-279
ALPK3	1.520010233	3.19E-181
ALPL	0.797683093	2.95E-39
ALPP	-0.000764173	0.852654153
ALS2	0.158156833	0.254252023
ALS2CL	-1.310863547	4.09E-34
ALX1	0.30751877	3.29E-56
ALX3	0.483865866	7.42E-65
ALX4	0.515190229	1.85E-69
AMACR	-2.019469773	5.86E-214
AMBN	-0.315499354	7.37E-07
AMBP	-0.073489144	0.009881446
AMBRA1	1.215037701	1.43E-65
AMD1	-0.105459919	0.129824191
AMDHD1	0.381556439	5.18E-13
AMDHD2	-0.104710051	0.169928976
AMELX	0.078523759	4.63E-06
AMELY	0.004481189	0.233782923
AMER1	0.567857701	1.21E-22
AMER2	-0.914061767	2.16E-40
AMER3	-1.743497871	1.39E-51
AMFR	1.64644097	1.55E-167
AMH	0.683016852	1.98E-12
AMHR2	-0.012349975	0.361265566
AMIGO1	0.065932493	0.510717034
AMIGO2	1.215068654	3.59E-48
AMIGO3	0.0070657	6.42E-34
AMMECR1	1.340172928	5.77E-267
AMMECR1L	1.77820068	6.46E-186
AMN	-0.070225603	0.001768694
AMN1	-0.53115668	4.11E-16
AMOT	0.537089992	5.90E-13
AMOTL1	1.547493679	1.37E-179
AMOTL2	0.861305253	1.02E-34
AMPD1	-0.034591242	0.000549776
AMPD2	-0.589817916	1.01E-13
AMPD3	0.679753171	2.06E-23
AMPH	-0.454739912	0.000868819
AMT	-3.71008866	2.44E-262
AMTN	0.005881076	0.333015993
AMY1A	0.00722789	9.46E-14

AMY1B	0.003468536	0.561953227
AMY1C	-0.002343687	0.028558919
AMY2A	-0.787454669	2.48E-45
AMY2B	-1.821075168	2.32E-84
AMZ1	-0.09683952	0.04238132
AMZ2	0.74018358	1.02E-41
ANAPC1	0.526813576	1.14E-21
ANAPC10	-0.15552855	0.00028838
ANAPC11	1.58076682	1.39E-213
ANAPC13	1.220384287	5.54E-95
ANAPC15	-0.324523834	1.46E-11
ANAPC16	1.012749145	2.21E-62
ANAPC2	0.526072001	1.54E-11
ANAPC4	-0.325469051	1.28E-07
ANAPC5	-0.098164623	0.169041496
ANAPC7	0.989129365	2.11E-49
ANG	1.472171511	3.57E-147
ANGEL1	0.429995728	7.06E-14
ANGEL2	0.816369308	7.72E-46
ANGPT1	1.502184455	8.79E-133
ANGPT2	3.069507015	1.10E-294
ANGPT4	0.162815198	2.00E-16
ANGPTL1	1.738225518	1.94E-225
ANGPTL2	3.300936059	1.00E-211
ANGPTL3	-0.002691313	0.707717534
ANGPTL4	1.787432459	6.01E-42
ANGPTL5	0.060083102	4.23E-05
ANGPTL6	0.810703052	6.31E-115
ANGPTL7	-0.427286179	7.28E-07
ANGPTL8	0.126317363	2.15E-06
ANHX	-0.010711714	0.011611287
ANK1	-1.344383815	1.37E-16
ANK2	-0.339565136	0.000102988
ANK3	-2.210124027	1.78E-97
ANKAR	0.402728208	2.61E-26
ANKDD1A	-0.441404798	3.46E-12
ANKDD1B	-0.156649851	2.49E-05
ANKEF1	0.409382814	1.61E-18
ANKFN1	1.339089026	3.50E-256
ANKFY1	1.040878583	3.11E-52
ANKH	-0.137558684	0.068421388
ANKIB1	1.841816525	3.11E-223
ANKK1	-0.138458576	0.000622265
ANKLE1	0.025008073	0.777245579
ANKLE2	0.310994254	2.36E-06
ANKMY1	-0.004679883	0.946751584
ANKMY2	1.420980347	3.11E-85
ANKRA2	1.005920746	1.72E-43

ANKRD1	0.101468481	0.030296436
ANKRD10	1.376852907	1.06E-86
ANKRD11	0.154569993	0.023075039
ANKRD12	0.151217254	0.03362618
ANKRD13A	0.687430057	7.92E-29
ANKRD13B	-0.325806526	0.000253062
ANKRD13C	0.746597095	5.80E-24
ANKRD13D	-0.238745679	0.000178712
ANKRD16	0.385221417	4.76E-11
ANKRD17	0.996672209	1.22E-48
ANKRD18A	-0.80137993	3.67E-40
ANKRD18B	-0.847887108	4.21E-23
ANKRD2	-0.846607951	2.04E-40
ANKRD20A1	0.085907324	4.50E-17
ANKRD23	-1.76055952	5.32E-97
ANKRD24	-1.057070422	2.18E-26
ANKRD26	-0.660016168	3.30E-28
ANKRD27	0.518235101	6.24E-20
ANKRD28	0.040440247	0.639305098
ANKRD29	-1.194054076	7.20E-84
ANKRD30A	0.008514449	3.62E-08
ANKRD30B	-0.596140497	8.03E-31
ANKRD30BL	-0.648790507	4.83E-45
ANKRD31	-0.053336224	2.16E-06
ANKRD33	-0.026094405	0.025652366
ANKRD33B	-0.529536709	1.71E-07
ANKRD34A	-1.395579433	1.39E-36
ANKRD34B	-1.002129928	4.34E-16
ANKRD34C	-1.080336237	2.04E-39
ANKRD35	0.341858765	1.01E-09
ANKRD36	-0.769675083	3.79E-25
ANKRD36B	-0.206433219	1.91E-05
ANKRD36C	-0.647567652	1.21E-19
ANKRD37	-0.328684053	2.24E-07
ANKRD39	-1.265878834	8.93E-69
ANKRD40	-0.086006901	0.236415252
ANKRD42	0.410804198	4.11E-12
ANKRD44	1.165665229	1.24E-122
ANKRD45	-0.272715051	0.000168375
ANKRD46	0.576365201	2.46E-16
ANKRD49	1.175578904	9.21E-63
ANKRD50	1.541445425	1.88E-205
ANKRD52	1.001949595	1.65E-33
ANKRD53	1.437142317	1.07E-163
ANKRD54	0.72073762	9.93E-35
ANKRD55	-0.310423713	5.25E-08
ANKRD6	0.333195281	0.001868448
ANKRD60	0.015893565	0.00012486

ANKRD61	0.745695765	1.46E-170
ANKRD62	-0.006531844	0.060488782
ANKRD63	-0.365453176	9.29E-07
ANKRD65	-0.897332457	1.92E-40
ANKRD66	0.013050869	0.759486888
ANKRD7	0.556048511	2.38E-172
ANKRD9	-0.29215738	2.59E-06
ANKS1A	0.100839786	0.184250594
ANKS1B	-1.573468267	3.89E-89
ANKS3	-0.512951689	8.76E-15
ANKS4B	-0.226385104	2.74E-09
ANKS6	0.399855162	0.000137269
ANKUB1	0.355861957	1.83E-43
ANKZF1	-0.527188787	7.37E-07
ANLN	0.462100668	0.000508523
ANO1	0.530909093	2.97E-47
ANO10	1.432638018	1.15E-164
ANO2	-0.107788039	0.001290067
ANO3	-1.631591989	4.18E-20
ANO4	-1.00860093	7.78E-53
ANO5	-0.27801636	0.000258493
ANO7	0.291202928	8.75E-21
ANO8	0.322144674	0.000249349
ANO9	-0.018150503	0.47976214
ANOS1	2.559860144	2.93E-187
ANP32A	1.060787465	7.12E-55
ANP32B	1.549495563	9.34E-100
ANP32D	0.19647037	1.91E-87
ANP32E	0.901319315	9.83E-45
ANPEP	1.841222442	8.2279222330172e-319
ANTXR1	2.419715106	2.00E-292
ANTXRL	-0.001023284	0.92975181
ANXA10	0.059745883	1.40E-29
ANXA11	0.476499223	1.10E-10
ANXA13	0.042754427	0.515968525
ANXA2	3.685671251	7.44E-276
ANXA3	-2.002416565	8.89E-177
ANXA4	0.814390695	2.44E-43
ANXA6	1.337789745	3.38E-51
ANXA7	0.703335061	1.34E-39
ANXA8	-0.002388896	0.780490329
ANXA8L1	-0.190215908	3.04E-27
ANXA9	2.028185488	7.48E-205
AOAH	2.223945223	1.26E-213
AOC1	0.206936971	1.30E-265
AOC2	-0.478745278	1.85E-14
AOC3	0.349692475	9.38E-13
AOX1	1.147103207	5.92E-193

AP1AR	-0.239188773	0.000549126
AP1B1	1.089539216	2.31E-70
AP1G1	1.031345874	4.48E-46
AP1G2	-3.062405942	9.69E-98
AP1M1	1.577632658	2.43E-136
AP1M2	-0.037088346	0.079484343
AP1S1	1.81715012	1.14E-80
AP1S2	2.277033048	1.12E-213
AP1S3	0.268559702	2.73E-10
AP2A1	0.286761122	0.000745307
AP2A2	0.262060916	0.005865153
AP2B1	1.285334628	1.17E-85
AP2M1	0.832495089	5.56E-27
AP2S1	1.720661245	5.23E-126
AP3B2	-1.182868071	8.57E-24
AP3D1	0.640846441	3.44E-18
AP3M1	1.739068325	6.12E-184
AP3M2	0.306387492	1.23E-05
AP3S1	-0.420347636	4.87E-14
AP3S2	-0.169286385	0.001315917
AP4B1	0.33180411	1.60E-05
AP4E1	0.831261498	3.22E-50
AP4M1	1.641103254	1.64E-146
AP4S1	0.249975599	3.49E-05
AP5B1	1.275273273	1.61E-59
AP5M1	0.863118294	7.55E-48
AP5S1	1.520746516	1.21E-233
AP5Z1	0.482648506	2.38E-13
APAF1	0.639705454	1.59E-25
APBA1	-2.075787618	3.70E-99
APBA2	0.270397456	0.004683487
APBA3	0.556028504	7.64E-16
APBB1	-0.550917122	2.37E-10
APBB1IP	3.265504773	1.17E-254
APBB2	0.915067665	2.74E-61
APBB3	-0.442084778	5.96E-07
APC	0.410812591	8.11E-08
APC2	0.533188276	1.62E-13
APCDD1	1.844750476	7.31E-190
APCDD1L	0.979381233	8.91E-155
APCS	-0.057689016	1.10E-05
APEH	1.685552329	8.93E-169
APELA	0.12886512	1.93E-49
APEX1	2.42442031	5.70E-245
APH1B	1.408094053	9.85E-124
API5	1.146548268	8.33E-59
APIP	1.073389488	1.12E-86
APLF	0.656019098	8.37E-167

APLNR	3.094936103	1.23E-93
APLP1	-0.381428569	1.19E-05
APLP2	0.863450812	9.11E-28
APMAP	1.926042948	2.26E-85
APOA1	0.035971777	0.318390457
APOA2	-0.292440246	1.99E-18
APOA4	-0.034198658	0.007980802
APOA5	0.011898676	0.048554721
APOB	0.056599825	1.51E-12
APOBEC1	-0.001661119	0.424008807
APOBEC2	0.168141604	8.62E-19
APOBEC3A	0.324057058	1.73E-27
APOBEC3D	-0.083443677	0.000468177
APOBEC3G	1.884373759	5.20E-233
APOBEC3H	0.744787672	2.76E-105
APOBEC4	0.00048424	0.95967477
APOBR	2.086723443	4.28E-261
APOC1	2.696477499	1.46E-52
APOC2	-2.358832427	2.75E-91
APOC3	-0.221385852	7.52E-11
APOC4	-0.300265136	1.25E-31
APOD	-0.23937611	0.010747484
APOE	1.473799473	3.06E-67
APOF	0.05613676	4.93E-10
APOH	0.332572098	5.43E-35
APOL1	2.36830144	1.84E-203
APOL2	1.559999921	1.91E-59
APOL3	1.175156201	2.38E-117
APOL5	0.103264595	1.23E-26
APOL6	2.369249899	9.45E-301
APOLD1	0.444812185	7.38E-07
APOO	1.139308493	2.50E-79
APOOL	1.258000909	2.28E-130
APP	1.426321466	1.07E-74
APPBP2	1.323528719	5.31E-88
APPL1	1.152195266	2.89E-70
APPL2	1.434957463	1.65E-115
APRT	1.644961981	8.02E-195
APTX	-0.625643661	3.46E-37
AQP1	3.583917789	3.64E-143
AQP10	0.005329531	0.392324996
AQP11	0.819327595	2.39E-35
AQP12A	-0.010577708	0.024765447
AQP12B	-0.021678798	7.09E-06
AQP2	0.039897719	4.20E-23
AQP3	0.679364874	1.50E-16
AQP4	4.251577	8.55E-223
AQP5	1.100356369	7.86E-84

AQP6	0.396337684	4.81E-18
AQP7	-1.079130325	6.60E-21
AQP8	-0.437133156	2.36E-18
AQP9	1.696166754	3.73E-155
AQR	1.077567975	3.27E-74
ARAF	2.328783528	9.07E-291
ARAP1	0.628956108	7.29E-25
ARAP2	0.639278055	2.68E-23
ARAP3	2.286099062	8.23E-199
ARC	3.03093833	7.05E-123
ARCN1	1.815928058	1.65E-142
AREG	1.320773236	3.74E-172
AREL1	0.966112943	8.59E-50
ARF1	1.55414147	3.25E-129
ARF3	0.361915171	8.87E-07
ARF5	1.009933177	6.63E-40
ARF6	2.08108788	1.58E-259
ARFGAP1	0.68332673	2.38E-21
ARFGAP2	0.226257878	0.001109486
ARFGAP3	1.495018031	8.98E-123
ARFGEF1	1.008735826	1.48E-58
ARFGEF2	0.620990307	1.40E-18
ARFGEF3	-1.156927513	2.24E-34
ARFIP2	1.200713032	1.89E-68
ARFRP1	-0.066263514	0.229939023
ARG1	0.212722573	2.10E-14
ARG2	0.011594989	0.913948081
ARGFX	-0.015011594	0.000879524
ARGLU1	0.037758986	0.690232347
ARHGAP1	1.215143729	2.22E-105
ARHGAP10	0.303874191	2.26E-12
ARHGAP12	1.25710814	4.99E-83
ARHGAP15	1.779487805	2.25E-285
ARHGAP17	0.523706108	1.57E-19
ARHGAP19	0.891474902	1.55E-73
ARHGAP20	0.579777924	1.63E-13
ARHGAP21	-0.696426635	7.62E-18
ARHGAP22	0.087376794	0.239283807
ARHGAP23	-0.190116111	0.005963793
ARHGAP24	0.315954489	8.44E-07
ARHGAP26	-0.244366818	0.007321511
ARHGAP27	-0.102099176	0.170823238
ARHGAP28	0.311522085	4.30E-40
ARHGAP29	0.460443817	1.18E-08
ARHGAP30	2.485648727	9.47E-275
ARHGAP32	0.228625507	0.004606105
ARHGAP33	0.086373897	0.269776033
ARHGAP35	0.203529661	0.000506104

ARHGAP36	0.287350836	0.026914628
ARHGAP39	0.471160535	1.42E-09
ARHGAP4	0.756547383	1.58E-17
ARHGAP40	0.08368737	0.004453916
ARHGAP44	-3.060955617	1.15E-150
ARHGAP45	1.54419425	6.08E-147
ARHGAP5	-0.949052226	4.53E-72
ARHGAP6	0.936334858	2.59E-49
ARHGAP8	-0.740245435	5.88E-88
ARHGAP9	1.093055132	1.35E-39
ARHGDIA	0.962837361	1.15E-51
ARHGDIG	-3.778578507	1.84E-215
ARHGEF1	0.729399661	8.57E-29
ARHGEF10L	0.901986611	3.87E-14
ARHGEF11	0.479840598	5.34E-08
ARHGEF12	-0.560808488	1.67E-16
ARHGEF15	0.806172371	1.47E-62
ARHGEF16	-0.360575634	8.07E-13
ARHGEF17	-0.125798494	0.190303446
ARHGEF18	0.489803128	7.45E-18
ARHGEF19	0.933254129	1.48E-147
ARHGEF2	0.73050021	1.36E-17
ARHGEF25	-1.714888238	3.17E-43
ARHGEF26	2.051808868	1.02E-207
ARHGEF28	-0.112616955	0.025779915
ARHGEF3	1.835642015	2.02E-85
ARHGEF33	-1.145544421	5.86E-32
ARHGEF35	0.013019358	0.462451796
ARHGEF37	-0.651590153	1.17E-16
ARHGEF39	0.755164281	1.26E-35
ARHGEF4	-0.523021864	7.68E-11
ARHGEF40	1.737764688	3.23E-158
ARHGEF5	-0.092823907	0.002925038
ARHGEF7	0.601720387	3.13E-12
ARHGEF9	-0.351670961	9.88E-05
ARID1A	0.926257181	2.07E-42
ARID1B	0.115925593	0.07751985
ARID2	1.074520298	1.04E-60
ARID3A	0.814317179	9.93E-34
ARID3B	0.214658792	0.002433499
ARID3C	0.105107388	0.018116437
ARID4A	0.531741477	1.63E-16
ARID4B	0.050856475	0.483373404
ARID5A	2.1858303	1.74E-153
ARID5B	1.812427636	1.70E-161
ARIH1	0.672981832	3.88E-30
ARIH2	0.896742963	3.08E-35
ARL1	1.443215063	8.80E-111

ARL10	1.138068882	1.77E-81
ARL13B	1.798466495	5.69E-296
ARL14	0.017841784	0.00031162
ARL14EP	1.403657418	1.34E-115
ARL14EPL	0.34874889	5.40E-240
ARL15	1.134550897	2.54E-76
ARL16	0.688049547	7.07E-32
ARL17A	0.145363958	4.73E-05
ARL17B	-0.064250216	0.071934398
ARL2	-1.399525627	9.87E-106
ARL2BP	-1.409159628	3.38E-88
ARL3	0.544188408	6.84E-18
ARL4C	2.664935784	5.91E-130
ARL4D	1.542300318	5.11E-53
ARL5A	1.11542092	3.23E-81
ARL5B	1.095826701	4.17E-73
ARL5C	-0.091784918	4.00E-13
ARL6	0.651872148	2.30E-22
ARL6IP1	0.723127586	1.99E-24
ARL6IP5	1.797019162	9.30E-139
ARL8A	1.160458497	6.83E-69
ARL8B	1.101699546	9.83E-59
ARL9	0.977986293	2.72E-76
ARMC1	1.417447268	2.72E-110
ARMC10	1.278656505	1.69E-113
ARMC12	0.905790269	2.76E-93
ARMC2	0.319097972	4.70E-08
ARMC3	0.039454935	0.562292144
ARMC5	0.656617556	8.14E-26
ARMC6	1.822999221	1.20E-167
ARMC7	1.645010624	1.14E-98
ARMC8	0.641061478	8.25E-12
ARMC9	0.989181757	2.35E-57
ARMCX1	1.362015836	1.52E-94
ARMCX2	1.266213603	6.63E-67
ARMCX3	1.163273226	3.45E-80
ARMCX4	0.027250321	0.707000645
ARMCX5	0.958952354	3.95E-75
ARMCX5-GPRASP2	-0.748373881	1.48E-29
ARMS2	0.219224994	2.18E-37
ARMT1	1.059381559	1.42E-57
ARNT	1.018043373	4.77E-40
ARNT2	0.296933807	1.92E-05
ARNTL	0.67635634	5.41E-20
ARNTL2	1.534499073	3.23E-142
ARPC1A	-0.614776206	1.02E-25
ARPC1B	-2.579016425	5.13E-279
ARPC2	1.387953097	8.91E-121

ARPC3	0.853431447	3.53E-50
ARPC4	0.983776564	7.16E-75
ARPC5	2.246757391	2.42E-270
ARPC5L	0.626833441	7.47E-19
ARPIN	1.165292594	4.80E-131
ARPP19	0.523240846	3.09E-10
ARPP21	-2.613756436	7.43E-54
ARRB1	-1.857939508	1.38E-132
ARRB2	1.072624516	9.43E-36
ARRDC1	0.285504997	1.08E-05
ARRDC2	0.096468659	0.232971453
ARRDC3	2.294426673	1.08E-132
ARRDC4	1.977514793	2.77E-104
ARRDC5	0.409181995	1.26E-111
ARSA	0.651764979	1.07E-20
ARSB	1.664164333	7.92E-292
ARSF	1.563012476	1.67E-99
ARSG	0.563890681	3.79E-18
ARSH	0.011710796	0.010528017
ARSI	1.765375817	8.44E-168
ARSJ	2.269706947	2.69E-206
ARSK	1.678422679	1.17E-210
ART1	-0.037347359	0.000498816
ART3	-0.229854766	0.031141535
ART4	0.00500008	0.235196775
ART5	0.203036552	5.78E-31
ARTN	0.385092266	1.66E-09
ARV1	1.171175592	4.21E-95
ARVCF	0.118041993	0.371688862
ARX	0.631293649	2.23E-07
ASAH1	0.85974979	1.28E-45
ASAH2	0.055342393	0.016216827
ASAH2B	-0.196282621	0.000215618
ASAP1	1.793003765	6.02E-200
ASAP2	0.304676193	0.000162359
ASAP3	2.247318086	4.34E-238
ASB1	0.157549885	0.000687793
ASB10	0.013882624	0.001453016
ASB11	0.128778578	2.68E-99
ASB12	0.000490074	0.983597163
ASB13	0.852775364	6.73E-31
ASB14	0.083480662	0.017391271
ASB15	-0.00973196	0.001873591
ASB16	0.232927114	0.009386881
ASB17	0.008601204	0.155609756
ASB18	-0.050626316	2.27E-06
ASB2	-0.09668342	0.223448086
ASB3	1.357023262	1.37E-196

ASB4	-0.525838835	1.71E-27
ASB5	0.72742734	1.34E-66
ASB6	0.614588547	3.90E-20
ASB7	1.248726852	1.43E-120
ASB8	1.242691303	6.84E-84
ASCC1	0.969158731	7.86E-69
ASCC2	1.527632335	5.19E-202
ASCC3	1.922514263	4.29E-289
ASCL1	2.153069153	3.66E-216
ASCL2	1.051517362	1.05E-189
ASCL3	-0.020224423	0.09794901
ASCL4	-0.002464921	0.69817939
ASCL5	-0.052818416	0.119939
ASF1A	1.157158208	3.34E-113
ASGR1	0.119948339	0.209391378
ASGR2	0.490749704	9.59E-39
ASH1L	0.282756469	0.000204836
ASH2L	1.054228638	6.04E-58
ASIC1	0.565723046	1.05E-14
ASIC2	-2.015432	1.31E-116
ASIC3	-0.913148004	1.72E-18
ASIC4	1.050571975	1.83E-42
ASIC5	0.0781002	2.44E-85
ASL	-0.037387329	0.568171813
ASMT	-0.078576959	0.000667552
ASMTL	1.182628284	5.58E-77
ASNSD1	0.521714278	6.46E-14
ASPA	-1.215118042	5.64E-35
ASPDH	-2.365484998	1.33E-248
ASPG	-0.212624227	3.25E-25
ASPH	0.965144098	4.55E-49
ASPHD1	-2.105739716	2.57E-217
ASPHD2	-0.013728152	0.910347922
ASPN	1.847508962	1.15E-261
ASPRV1	0.154132239	0.00374209
ASPSCR1	-0.140124667	0.014821948
ASRGL1	-0.986533508	1.84E-66
ASS1	0.197739964	0.016910145
ASTE1	1.532326006	1.45E-170
ASTL	-0.192416667	7.85E-08
ASTN1	1.350993606	8.79E-62
ASTN2	1.62623426	2.34E-125
ASXL1	0.810808474	7.64E-29
ASXL2	1.132153754	1.21E-123
ASXL3	0.311865253	2.59E-11
ASZ1	0.000358876	0.802646011
ATAD1	0.578956369	1.65E-21
ATAD2B	0.597018397	2.83E-25

ATAD3A	1.605559817	1.38E-172
ATAD3B	-0.557936834	5.14E-13
ATAD3C	0.454064471	3.94E-15
ATAD5	1.06630793	3.73E-68
ATAT1	1.391478108	2.11E-136
ATCAY	-0.783621233	5.42E-13
ATE1	0.533518962	3.97E-16
ATF1	1.643384112	1.69E-110
ATF2	0.980652598	9.14E-43
ATF3	2.325161029	6.98E-91
ATF4	1.139445137	3.13E-65
ATF5	1.78680664	5.75E-189
ATF6	1.903042547	5.79E-209
ATF6B	0.905610672	1.01E-33
ATF7	0.4745185	3.21E-09
ATF7IP	1.706875383	1.82E-146
ATF7IP2	-0.586650386	4.17E-34
ATG10	1.204561663	1.27E-232
ATG101	0.952168958	2.80E-48
ATG12	1.137807966	6.81E-71
ATG13	0.369600547	1.75E-10
ATG14	0.794724928	2.91E-36
ATG16L1	0.226045514	0.017145146
ATG16L2	0.103947576	0.112648987
ATG2A	0.674916816	9.90E-21
ATG2B	-0.172644706	0.032982957
ATG3	0.798543941	1.27E-49
ATG4A	1.574699559	1.53E-162
ATG4B	0.348184288	1.29E-05
ATG4C	1.213684086	2.75E-84
ATG4D	0.819960102	1.88E-25
ATG5	1.630995893	3.19E-169
ATG7	1.308636826	1.31E-122
ATG9A	-0.189215374	0.015566152
ATG9B	-0.184729451	0.001785702
ATIC	1.281078006	1.75E-112
ATL1	-1.200728384	1.59E-32
ATL2	0.714714801	2.70E-21
ATL3	2.143272459	1.91E-301
ATM	0.547378472	5.04E-14
ATMIN	0.894572081	7.23E-50
ATN1	0.569636577	1.68E-11
ATOH1	0.034025885	4.06E-10
ATOH7	-0.825511238	1.08E-27
ATOH8	0.85456692	4.26E-40
ATOX1	1.682023352	4.86E-242
ATP10A	-0.5928699	6.61E-25
ATP10B	-0.104816627	0.167552219

ATP11A	0.985477682	1.11E-50
ATP11B	1.125901264	1.03E-59
ATP11C	1.201203635	4.99E-103
ATP12A	0.074667917	5.44E-06
ATP13A1	0.7647327	1.22E-25
ATP13A2	-0.124158191	0.154121008
ATP13A3	0.969779936	1.03E-48
ATP13A4	1.562510472	1.60E-85
ATP13A5	0.61067763	2.76E-75
ATP1A1	-0.857970363	2.30E-12
ATP1A2	-0.493283402	8.80E-08
ATP1A3	-4.580824784	2.21E-176
ATP1A4	0.100210313	3.92E-07
ATP1B1	-0.669474327	7.68E-09
ATP1B2	1.258879416	6.25E-43
ATP1B3	1.693544374	6.29E-98
ATP1B4	0.101604132	2.02E-37
ATP23	0.424617281	1.04E-09
ATP2A1	0.370349859	1.73E-18
ATP2A2	0.401252603	3.54E-06
ATP2A3	-0.307246681	0.01351002
ATP2B1	-0.200569143	0.090963796
ATP2B2	-1.588141334	5.15E-28
ATP2B3	-2.369660076	9.38E-74
ATP2B4	1.83434693	1.38E-126
ATP2C1	0.954583363	6.81E-60
ATP2C2	-0.882870179	1.67E-76
ATP4A	-0.758302397	1.14E-14
ATP4B	-0.054171968	0.193716786
ATP6AP1	1.370729594	1.83E-53
ATP6AP2	0.565609961	1.60E-15
ATP6V0A1	-1.07614373	8.83E-39
ATP6V0A2	0.82765725	4.39E-43
ATP6V0A4	0.080844431	0.002728365
ATP6V0B	1.621931383	1.88E-111
ATP6V0D1	-0.296505401	0.000369699
ATP6V0D2	0.430557991	2.05E-129
ATP6V0E2	-0.476803023	2.31E-10
ATP6V1A	0.595376891	3.53E-09
ATP6V1B1	-0.357532807	1.29E-07
ATP6V1B2	-0.075899104	0.468057991
ATP6V1C1	0.429540921	7.10E-07
ATP6V1C2	-0.253602808	2.48E-08
ATP6V1D	0.641676275	1.45E-19
ATP6V1E1	-0.162994574	0.044094147
ATP6V1E2	0.915019452	2.36E-111
ATP6V1F	1.394612304	1.35E-96
ATP6V1G1	0.584968222	5.16E-23

ATP6V1G2	-2.712570428	5.56E-112
ATP6V1G3	0.029012636	6.76E-09
ATP6V1H	-0.114283039	0.190566961
ATP7A	1.251011439	2.82E-240
ATP7B	-1.069631296	2.08E-42
ATP8A1	-1.564415521	1.97E-80
ATP8A2	-2.113178045	7.59E-65
ATP8B1	0.781302935	4.19E-86
ATP8B2	0.540652455	2.41E-07
ATP8B4	0.812539136	3.33E-65
ATP9A	0.112268021	0.174376325
ATP9B	0.290119333	6.09E-08
ATPAF1	0.279902874	4.77E-07
ATPAF2	0.876692909	1.98E-78
ATR	-0.234199975	0.000112221
ATRIP	1.111955527	2.39E-56
ATRN	1.243049062	4.51E-60
ATRNL1	-0.211847536	0.060562817
ATRX	0.249144264	0.000142639
ATXN1	0.4413921	1.15E-11
ATXN10	1.309976983	2.34E-74
ATXN1L	0.963584152	8.66E-83
ATXN2	-0.33141813	3.86E-07
ATXN2L	0.492183052	1.90E-06
ATXN3	1.122043513	1.30E-155
ATXN3L	-0.00029158	0.864905292
ATXN7	-0.114727152	0.086134201
ATXN7L1	0.778150492	3.37E-39
ATXN7L2	0.14547681	0.117001064
ATXN7L3	-0.060174066	0.479971403
ATXN7L3B	0.779683372	1.54E-25
AUH	0.144894433	0.046820149
AUNIP	1.309597575	2.16E-149
AUP1	1.333784446	3.40E-99
AURKAIP1	1.287530588	1.26E-138
AURKC	0.630894587	5.76E-20
AUTS2	-0.122190839	0.021512186
AVEN	0.487355535	9.05E-25
AVIL	1.314708679	6.18E-38
AVL9	1.040619928	2.88E-56
AVP	-0.919129364	3.24E-06
AVPI1	-0.363341746	1.12E-06
AVPR1A	0.836437621	9.53E-252
AVPR1B	-0.064848558	0.009752181
AVPR2	-0.287318121	2.55E-06
AWAT1	0.026868182	9.12E-22
AWAT2	0.064705468	8.79E-41
AXDND1	0.035780954	0.000810756

AXIN1	0.255047262	0.000131894
AXIN2	0.364805335	5.16E-10
AXL	1.911105234	6.33E-174
AZGP1	1.793560983	2.56E-77
AZI2	-0.221240791	0.00039156
AZIN1	1.693626777	1.59E-139
AZIN2	-0.265894942	8.44E-05
AZU1	-0.488677072	3.17E-23
B2M	2.648102035	3.94E-254
B3GALNT1	0.701729244	2.22E-19
B3GALNT2	1.268170865	4.96E-134
B3GALT1	0.542203273	1.57E-15
B3GALT2	0.110378536	0.258963206
B3GALT4	1.235172911	1.28E-134
B3GALT5	0.317043858	4.33E-07
B3GALT6	1.890585482	2.82E-201
B3GAT1	-0.552229939	1.93E-11
B3GAT2	0.032291024	0.686535952
B3GAT3	1.087772859	2.98E-67
B3GLCT	2.179387877	8.60E-140
B3GNT2	1.289668934	1.54E-60
B3GNT3	0.011388538	0.058602718
B3GNT4	-0.996758953	4.28E-41
B3GNT6	0.017374038	0.001260989
B3GNT7	1.264903404	2.27E-113
B3GNT8	0.502594844	2.91E-46
B3GNT9	2.631544323	4.80E-306
B3GNTL1	0.246846683	4.91E-07
B4GALNT1	-0.839715432	7.43E-11
B4GALNT2	-0.001981361	0.694760532
B4GALNT3	-0.258412211	0.000763137
B4GALNT4	-0.440786418	1.26E-08
B4GALT1	2.275201168	2.48E-143
B4GALT2	1.011199597	1.98E-78
B4GALT3	1.33269112	4.07E-88
B4GALT4	1.728774644	1.10663333384125e-310
B4GALT5	2.685742143	5.43E-211
B4GALT6	-0.236932951	0.031912809
B4GALT7	0.835344478	1.86E-56
B4GAT1	0.652768089	5.68E-12
B9D1	0.14832043	0.030136044
B9D2	1.831174087	1.72E-209
BAALC	1.435209786	6.89E-106
BAAT	-0.247666878	4.79E-13
BABAM1	-0.559277226	4.50E-16
BACE1	-0.096533722	0.2620736
BACE2	2.346106147	1.12E-221
BACH2	0.605862912	1.87E-26

BAD	-0.355584949	3.90E-16
BAG1	0.56247999	8.86E-35
BAG2	0.223154901	3.45E-06
BAG3	0.66142837	4.95E-10
BAG4	0.653982347	1.23E-15
BAG5	1.379995482	1.14E-93
BAG6	1.248762224	9.70E-75
BAHCC1	-0.050451072	0.636134838
BAHD1	0.815959148	1.11E-46
BAIAP2	-0.522808411	7.88E-09
BAIAP2L1	-0.13963897	1.73E-06
BAIAP2L2	-0.509076523	3.32E-13
BAIAP3	0.152931792	0.417470621
BAK1	1.898147501	1.14E-249
BAMBI	1.086191809	8.04E-55
BANF2	0.139963643	8.15E-109
BANK1	0.121009438	0.020033449
BANP	0.960102458	1.32E-55
BAP1	0.47269293	4.46E-11
BARHL1	-0.305854271	0.084287447
BARHL2	-1.065560678	5.24E-08
BARX1	0.988794169	3.21E-236
BARX2	-0.479201039	2.31E-15
BASP1	-0.734962517	1.18E-08
BATF	1.768770613	9.86E-109
BATF2	2.205136086	4.00E-304
BATF3	1.981920422	8.52E-158
BAX	2.3730853	3.69962107518174e-315
BAZ1A	1.774723034	1.60E-273
BAZ1B	1.628797604	5.81E-146
BAZ2A	0.5851599	5.48E-12
BAZ2B	-0.118570789	0.059432084
BBC3	0.730105703	6.33E-16
BBIP1	-0.410682141	5.73E-12
BBOF1	0.180106776	0.000110392
BBOX1	2.487683695	3.43E-199
BBS10	1.77280141	7.93E-177
BBS12	1.711472339	3.38E-196
BBS2	0.001594791	0.980820093
BBS4	1.52757483	2.25E-165
BBS5	-0.268472256	9.10E-07
BBS7	0.474330683	2.51E-09
BBS9	1.210802861	4.21E-143
BBX	1.334806043	6.59E-117
BCAM	1.436555749	8.18E-149
BCAP31	1.578235122	6.02E-187
BCAR1	0.730627409	2.25E-19
BCAR3	0.374013956	3.63E-12

BCAS1	-2.570442944	2.57E-86
BCAS2	1.547839451	1.04E-105
BCAS3	0.376090181	1.01E-10
BCAS4	-0.219177274	0.002676194
BCAT1	2.731823464	2.14E-232
BCAT2	0.357526092	3.25E-06
BCCIP	0.882619949	1.48E-73
BCDIN3D	1.238261095	3.54E-148
BCKDHB	1.716457373	1.87E-198
BCKDK	1.732823974	1.80E-155
BCL10	1.648695123	1.02E-273
BCL11A	-1.140057206	3.95E-26
BCL11B	-0.920993773	5.18E-15
BCL2	0.627482382	1.21E-44
BCL2A1	3.154470124	4.74E-239
BCL2L1	0.40239719	1.93E-11
BCL2L10	-0.69460054	1.14E-30
BCL2L13	1.530899655	5.38E-163
BCL2L14	0.181664154	9.87E-168
BCL2L15	-0.324075645	1.66E-09
BCL2L2	-1.651701384	3.21E-175
BCL2L2-PABPN1	-0.990931033	1.88E-48
BCL3	2.309822249	2.16E-190
BCL6	0.848651438	3.61E-16
BCL6B	1.795895827	2.33E-181
BCL7A	0.292412068	0.000343482
BCL7B	1.251595565	1.69E-113
BCL7C	1.69434036	3.1643849930838e-312
BCL9	1.40614287	2.53E-68
BCL9L	0.571834492	1.49E-08
BCLAF1	0.855930357	1.18E-29
BCO1	1.543859973	2.77E-230
BCO2	-1.048593112	1.99E-71
BCOR	1.067348576	1.56E-73
BCORL1	1.10697413	1.70E-70
BCR	6.83E-05	1
BCS1L	1.111797762	1.89E-80
BDH1	-1.241752621	3.05E-43
BDH2	-0.249198064	0.001185828
BDKRB1	-0.140368119	0.008147287
BDKRB2	1.130256215	1.22E-58
BDNF	-0.24256518	0.004847965
BDP1	0.190063099	0.013805724
BEAN1	0.271850763	6.85E-06
BECN1	0.040657429	0.577979395
BECN2	0.038854005	5.30E-22
BEGAIN	-2.356845923	3.52E-101
BEND2	0.018788919	7.92E-45

BEND3	1.34341357	1.80E-163
BEND4	-0.142368592	6.45E-05
BEND5	1.614627912	1.41E-98
BEND6	0.228684146	0.012003844
BEND7	0.34603988	2.53E-07
BEST1	-0.844743465	1.04E-21
BEST2	0.134884527	3.09E-28
BEST3	1.926671071	1.35E-148
BEST4	0.400933953	2.90E-07
BET1	1.934384326	1.22894382812198e-318
BET1L	0.920844616	7.57E-65
BEX1	0.08497356	0.419766558
BEX2	-0.774589523	1.27E-17
BEX3	1.14439142	9.47E-59
BEX4	-0.260823705	0.000340924
BEX5	-1.573207739	4.71E-46
BFAR	2.220008559	1.64E-292
BFSP1	-0.490447021	1.00E-22
BFSP2	0.108669013	2.14E-97
BHLHA15	0.187495071	1.70E-06
BHLHA9	-0.284014008	8.39E-14
BHLHB9	-2.916068338	3.23E-264
BHLHE22	-1.827816633	2.69E-41
BHLHE23	-0.000607233	0.924775387
BHLHE40	0.969737781	5.82E-36
BHLHE41	0.905007867	3.98E-31
BHMT	-0.224716346	1.73E-12
BHMT2	-0.868407197	9.23E-41
BICC1	1.188191561	1.41E-156
BICD1	1.520085519	2.51E-66
BICD2	0.964067214	4.16E-37
BICDL1	-2.360091197	9.41E-82
BICDL2	-0.118861943	9.12E-06
BID	0.66493136	2.18E-28
BIK	0.776702584	4.71E-230
BIN1	-1.190788041	3.97E-81
BIN2	2.105308891	8.25E-182
BIN3	0.312260049	6.00E-08
BIRC2	1.843929843	1.56E-185
BIRC3	1.513343269	4.31E-171
BIRC6	0.522255709	5.62E-13
BIRC7	0.808797055	2.82E-100
BIVM	-0.447466859	3.01E-09
BLCAP	0.275263047	0.000407103
BLID	0.021442572	0.01293448
BLK	0.066451509	9.82E-14
BLM	1.821388273	1.63E-236
BLMH	1.235344357	5.49E-78

BLNK	0.805708558	2.16E-25
BLOC1S1	-0.920728775	1.50E-82
BLOC1S2	0.88867023	5.41E-48
BLOC1S3	1.181806485	3.76E-113
BLOC1S6	1.290095479	1.04E-80
BLVRA	1.9075696	5.10E-189
BLVRB	2.001331206	1.12E-225
BLZF1	1.378970352	4.86E-151
BMF	2.046254077	4.81E-271
BMI1	0.385749607	4.03E-10
BMP1	1.993504668	5.82E-105
BMP10	-0.04296901	0.000128132
BMP15	0.023704471	1.11E-19
BMP2K	0.378044783	4.74E-12
BMP3	-0.234187493	6.25E-13
BMP4	1.20030111	6.14E-48
BMP5	0.57765505	4.45E-52
BMP6	-0.089054684	0.162861442
BMP7	1.937879437	1.10E-158
BMP8A	0.956348984	3.45E-87
BMP8B	1.113575802	1.59E-91
BMPER	0.766994065	3.22E-18
BMPR1A	1.749510267	1.47310739863848e-311
BMPR1B	1.162287306	1.46E-60
BMPR2	0.939578288	1.80E-55
BMS1	0.490112103	4.16E-13
BMT2	1.919488255	2.07E-207
BMX	0.106191528	2.52E-06
BNC1	0.098205616	2.65E-27
BNC2	0.850171708	2.60E-216
BNIP1	1.906997374	1.04E-283
BNIP2	1.335484555	3.09E-126
BNIP3	0.547177427	3.34E-18
BNIP3L	1.871464665	4.37E-166
BNIPL	0.234089397	7.59E-09
BOC	1.729631647	4.76E-147
BOD1	1.864894318	2.08E-216
BOD1L1	0.254460668	0.00063919
BOD1L2	-0.017811194	0.009214829
BOK	-1.166069443	2.11E-39
BOLA1	1.63424833	1.45E-217
BOLA2	-2.077660112	2.49E-205
BOLA2B	0.48565019	4.34E-49
BOLA3	-0.923468468	2.14E-56
BOLL	0.010163054	0.000686566
BOP1	1.080874332	1.08E-35
BORA	1.224649986	1.97E-132
BORCS5	0.498288478	1.45E-23

BORCS6	0.685715726	2.24E-51
BORCS7	0.11110025	0.043766603
BORCS8	-0.664232158	1.06E-29
BPGM	2.107011711	2.35E-261
BPHL	0.554020357	2.27E-23
BPI	0.199524324	5.24E-55
BPIFA1	0.01096197	0.006632391
BPIFA2	-0.011588779	0.121436516
BPIFA3	-0.005297364	0.013037527
BPIFB1	-0.004720305	0.347917324
BPIFB2	0.974092572	3.00E-241
BPIFB3	0.094279112	6.00E-143
BPIFB4	0.360718671	2.88E-140
BPIFB6	0.093762254	1.13E-137
BPIFC	0.129943704	6.11E-35
BPNT1	2.149041372	7.87E-275
BPTF	0.329256243	3.14E-05
BPY2	-1.34E-05	0.981129513
BPY2B	-2.55E-05	0.96238262
BPY2C	0	1
BRAF	-0.847789629	7.60E-27
BRAP	1.306803645	2.95E-129
BRAT1	1.007647295	1.11E-42
BRCC3	1.358111779	1.01E-77
BRD1	0.423693255	1.68E-05
BRD2	0.450410237	3.51E-09
BRD3	1.040906635	4.61E-74
BRD4	1.165462483	3.49E-64
BRD7	0.539083153	1.07E-27
BRD8	0.861688601	1.58E-32
BRD9	0.034626893	0.598444312
BRDT	-0.002002675	0.757739727
BRF1	-0.210889303	0.001884219
BRF2	0.877072676	3.57E-36
BRI3BP	0.044302614	0.49558063
BRICD5	-1.528361138	1.38E-32
BRINP1	-1.736984254	5.91E-69
BRINP2	1.350016226	2.06E-34
BRINP3	1.420852676	2.66E-99
BRIX1	0.231267966	8.57E-05
BRK1	2.095634076	1.94E-269
BRMS1	1.65870958	1.59E-207
BRMS1L	0.627301822	1.04E-22
BROX	1.609842798	3.62E-191
BRPF1	1.283323878	6.36E-60
BRPF3	0.780719226	6.18E-26
BRS3	-0.06543753	0.154388651
BRSK1	-1.442337979	1.15E-56

BRSK2	-1.680022419	9.08E-57
BRWD1	0.040174555	0.539576549
BRWD3	0.634968593	3.56E-23
BSDC1	0.529428562	4.52E-18
BSG	1.022665695	7.70E-104
BSN	-1.561398718	1.01E-31
BSND	-0.250663142	9.52E-11
BSPH1	-0.112696871	2.30E-07
BSPRY	-1.864447724	1.25E-53
BST2	3.110823645	5.11E-273
BSX	-0.027639252	0.374118854
BTAF1	0.374034853	4.43E-06
BTBD1	1.14517215	4.39E-90
BTBD10	0.904537169	1.70E-26
BTBD11	0.155509708	0.059959234
BTBD16	-0.221867869	0.000339145
BTBD17	2.421399517	3.95E-222
BTBD18	0.013951117	0.447502117
BTBD19	1.823422404	6.24E-265
BTBD2	-0.177225168	0.013214188
BTBD3	0.557210532	1.09E-07
BTBD6	0.172487647	0.012977778
BTBD7	0.946455986	8.86E-72
BTBD8	1.292462914	8.72E-176
BTBD9	-0.03740885	0.611352459
BTC	0.67356866	6.95E-45
BTD	0.432378869	2.17E-22
BTF3	0.084545622	0.121230134
BTF3L4	1.9982392	2.05E-198
BTG1	2.146398148	7.03E-88
BTG2	2.169996097	8.03E-139
BTG3	2.957545928	1.14E-217
BTG4	0.030865026	0.018060165
BTK	2.414423167	2.89E-263
BTLA	0.182965951	1.21E-91
BTN1A1	0.161603651	2.50E-16
BTN2A1	1.127158998	2.28E-33
BTN2A2	1.563668156	2.63E-60
BTN3A1	1.811225707	1.24E-125
BTN3A2	2.529434149	6.06E-275
BTN3A3	1.745233502	3.84E-168
BTNL2	0.238542021	1.22E-35
BTNL3	0.048100933	1.16E-07
BTNL8	0.127965284	2.78E-33
BTNL9	-1.132476433	3.61E-55
BTRC	-0.087510024	0.216437155
BUB3	1.478344758	1.36E-99
BUD13	1.881772704	1.77E-159

BUD31	1.411199275	1.34E-142
BVES	1.241745839	3.60E-61
BZW2	2.037940852	5.17E-134
C10orf105	-1.122430502	2.20E-22
C10orf120	-0.003174818	0.433051374
C10orf53	-0.044467033	2.13E-06
C10orf62	-0.132379255	0.00131318
C10orf67	-0.164016564	5.10E-14
C10orf71	-0.022955758	6.88E-05
C10orf82	-0.526945047	4.30E-24
C10orf88	0.510214353	1.47E-15
C10orf90	-0.140452131	0.067567606
C10orf95	-0.648777878	4.76E-44
C10orf99	-0.038479413	0.000141387
C11orf1	0.82375308	8.66E-57
C11orf16	-0.056574858	0.201900199
C11orf21	0.489205117	4.47E-88
C11orf24	1.973326116	1.03E-199
C11orf42	-0.07400932	0.16133851
C11orf45	0.793995225	7.81E-51
C11orf49	0.953144517	1.05E-57
C11orf52	-1.111085321	1.41E-31
C11orf53	-0.002785835	0.649064416
C11orf54	0.841502697	1.23E-64
C11orf58	0.405559036	4.63E-11
C11orf65	0.221351085	2.59E-28
C11orf68	1.054267016	3.03E-41
C11orf71	0.601329321	2.35E-30
C11orf80	-0.247566104	0.011129216
C11orf86	0.246745069	2.72E-67
C11orf87	-1.197901503	2.02E-22
C11orf91	0.069607834	0.029349847
C11orf94	0.627387152	1.37E-95
C11orf95	0.737287921	8.37E-22
C11orf96	1.421823837	2.40E-45
C11orf97	-1.023786567	1.95E-26
C12orf29	0.065312692	0.252109472
C12orf4	1.512170801	9.72E-102
C12orf40	0.012226676	7.12E-06
C12orf42	0.061364443	4.83E-27
C12orf43	0.378019294	1.83E-09
C12orf45	0.398877478	8.90E-30
C12orf50	0.02795819	0.000700618
C12orf54	-0.298674707	7.61E-31
C12orf56	-0.041347034	0.013119874
C12orf57	-0.056204743	0.308491297
C12orf60	0.96273111	5.18E-240
C12orf65	1.149031627	5.13E-93

C12orf66	1.309161789	2.95E-183
C12orf71	-0.025398435	0.3757179
C12orf73	0.491750487	7.74E-22
C12orf75	1.288175568	1.50E-82
C12orf76	0.477244661	6.12E-16
C14orf119	1.994655478	1.73E-283
C14orf132	-0.448950863	7.75E-07
C14orf180	-0.236047399	1.15E-17
C14orf28	0.115217757	0.003843517
C14orf39	0.151873259	7.04E-09
C14orf93	1.31113212	4.10E-284
C15orf40	0.656585786	3.60E-32
C15orf48	1.152542765	7.04E-216
C15orf61	-0.587550231	3.20E-23
C15orf62	-0.391790185	3.42E-06
C15orf65	1.045778023	2.22E-84
C16orf70	0.131269813	0.104393783
C16orf71	0.595059844	7.91E-44
C16orf72	0.640219692	2.31E-31
C16orf74	0.425295103	1.10E-16
C16orf78	0.005977525	0.149240337
C16orf82	-0.042859626	0.000218483
C16orf86	-0.413284509	0.000244472
C16orf87	1.067559373	2.18E-120
C16orf89	0.22029202	0.00216857
C16orf90	-0.006142889	0.514724837
C16orf91	1.845989904	9.41E-154
C16orf92	0.274537167	3.42E-10
C16orf95	-0.559147812	2.16E-58
C16orf96	0.46187127	3.86E-82
C17orf100	1.010015359	3.94E-63
C17orf107	-1.201462036	6.41E-62
C17orf49	-2.477279346	3.21E-299
C17orf50	-0.472789969	2.78E-27
C17orf58	1.696307631	4.45E-190
C17orf64	0.315819714	7.62E-100
C17orf67	-0.229483266	3.20E-08
C17orf75	0.710420347	4.15E-24
C17orf78	0.080157593	1.51E-33
C17orf80	1.495197747	5.26E-127
C17orf97	0.414748221	4.54E-12
C17orf98	0.171196977	5.83E-80
C17orf99	0.050391943	5.50E-28
C18orf21	0.957144735	1.45E-56
C18orf25	0.715817055	2.62E-28
C18orf54	1.687281283	2.30E-272
C18orf63	-0.00126137	0.340429226
C19orf12	0.987741744	1.70E-54

C19orf18	0.93419616	4.50E-61
C19orf25	1.125247339	6.93E-119
C19orf33	-1.152938112	6.76E-91
C19orf38	2.020762018	2.58E-226
C19orf44	0.870653152	1.23E-63
C19orf47	1.071256701	2.99E-137
C19orf53	1.907277584	5.30E-277
C19orf67	0.153241962	6.84E-42
C19orf71	0.116628103	0.331959026
C19orf73	0.418313418	2.75E-11
C19orf81	0.193079915	0.01339129
C19orf84	0.849349569	4.01E-295
C1D	1.302702906	3.82E-105
C1GALT1	1.419012922	9.54E-117
C1orf100	0.022973882	0.272588
C1orf105	0.114560055	4.04E-60
C1orf109	1.976178992	4.79E-229
C1orf112	1.461787644	1.44E-135
C1orf115	-1.085091143	1.68E-17
C1orf116	0.134050595	3.81E-40
C1orf122	0.838271928	4.35E-60
C1orf127	-0.24022061	0.000262726
C1orf131	1.562274757	1.31E-169
C1orf141	-0.077742109	0.003612779
C1orf146	0.23971984	1.43E-15
C1orf158	1.04229884	9.99E-80
C1orf159	-0.024851158	0.6714921
C1orf162	2.154190219	4.72E-114
C1orf167	-0.002643389	0.354578696
C1orf174	1.142951372	5.20E-80
C1orf185	0.090253528	1.04E-62
C1orf189	0.914291064	3.90E-89
C1orf194	0.860120172	1.87E-15
C1orf198	0.790888757	3.44E-20
C1orf21	0.858637391	2.77E-27
C1orf210	0.060402996	2.79E-08
C1orf216	0.834880147	7.16E-20
C1orf226	2.824287191	6.54E-242
C1orf35	0.899158864	7.10E-37
C1orf43	1.711138857	4.05E-181
C1orf50	-1.825584039	3.26E-273
C1orf52	1.155611929	1.06E-78
C1orf54	1.99349197	2.59E-241
C1orf56	1.665533833	1.91E-196
C1orf68	-0.124874924	1.52E-10
C1orf74	0.889165548	1.06E-50
C1orf87	-0.027758513	0.717113674
C1QA	5.39713385	3.71E-280

C1QB	5.403688034	1.21E-250
C1QBP	1.612584459	1.84E-141
C1QC	5.504640194	3.36E-273
C1QL1	2.946255281	3.32E-243
C1QL2	-1.738534111	3.36E-69
C1QL3	-0.82874406	1.00E-09
C1QTNF1	2.897080337	2.05355668332862e-317
C1QTNF12	0.093202148	0.058201898
C1QTNF2	1.226538656	3.79E-107
C1QTNF3	-0.393378126	8.08E-09
C1QTNF4	-2.721515073	2.87E-121
C1QTNF5	-0.196076079	4.09E-10
C1QTNF7	0.164187543	2.96E-16
C1QTNF8	-0.161951793	0.000166943
C1QTNF9	-0.023093669	0.406086596
C1QTNF9B	-0.309298337	3.55E-09
C1R	3.255848521	1.56E-250
C1RL	2.645404009	9.38E-295
C1S	3.154208832	2.18E-304
C2	1.380364267	1.98E-100
C20orf141	0.055623423	1.57E-23
C20orf144	0.615237113	3.99E-300
C20orf173	-0.02089042	0.003423093
C20orf194	-0.140936092	0.013532511
C20orf202	0.468676265	5.35E-35
C20orf203	-1.382280729	3.39E-97
C20orf27	1.657647244	1.97E-54
C20orf85	-0.236045879	2.28E-05
C20orf96	1.763884679	2.50E-194
C21orf58	1.121487964	5.68E-83
C21orf91	0.619961199	1.59E-11
C22orf15	0.018247063	0.755226635
C22orf23	-0.096956647	0.127944062
C22orf31	-0.361601459	1.05E-12
C22orf39	-0.265243572	2.62E-07
C22orf42	-0.2285278	0.004347683
C2CD2	0.274502609	0.000137048
C2CD2L	-1.262271153	2.02E-45
C2CD3	0.86193598	3.84E-50
C2CD4A	0.483593446	1.47E-36
C2CD4B	0.026125839	0.691237268
C2CD4C	-0.608193643	2.13E-07
C2CD4D	-0.284701796	1.23E-09
C2CD5	-0.198743021	0.055266006
C2orf15	-0.457318653	1.18E-14
C2orf16	0.505921256	2.08E-140
C2orf42	1.273203128	1.28E-108
C2orf49	1.429384967	4.20E-134

C2orf50	0.757160376	4.94E-54
C2orf66	1.693629376	1.41E-259
C2orf68	1.771521764	3.58E-119
C2orf69	0.591862608	8.55E-25
C2orf72	1.000557103	3.37E-39
C2orf73	0.232136088	6.83E-10
C2orf78	0.007392889	0.009124746
C2orf80	1.119211924	5.69E-13
C2orf88	1.729355367	2.07E-154
C3	4.575634861	4.29E-223
C3AR1	3.410523474	6.03E-216
C3orf14	-0.056124028	0.472605632
C3orf18	0.425196357	1.42E-06
C3orf20	0.238316853	3.56E-130
C3orf22	0.108453664	1.28E-44
C3orf33	-0.803178333	1.99E-56
C3orf49	0.176809212	1.54E-07
C3orf52	0.320288819	1.87E-80
C3orf56	-0.002465801	0.099524674
C3orf62	-0.006006085	0.92451559
C3orf70	1.236149596	1.08E-131
C3orf80	-0.644855836	1.29E-07
C3orf84	0.018655321	4.38E-08
C4A	1.989512442	4.72E-144
C4B	2.397901882	1.48E-205
C4BPA	0.270205143	2.89E-41
C4BPB	0.09915515	1.56E-16
C4orf17	0.022914091	1.82E-44
C4orf19	1.124579134	1.33E-143
C4orf3	1.249429875	3.03E-123
C4orf33	0.897547331	2.01E-39
C4orf36	-0.805755429	1.04E-123
C4orf45	0.039876694	0.006506144
C4orf47	1.714199265	6.34E-110
C4orf48	0.792763149	6.88E-24
C4orf50	-1.569062354	4.71E-57
C4orf51	0.126423767	8.94E-82
C5	1.041758428	9.78E-142
C5AR1	3.420431658	8.16E-243
C5AR2	1.006533348	5.37E-178
C5orf15	2.779949757	7.44097447232126e-319
C5orf22	1.268368425	1.99E-75
C5orf24	1.302690387	4.19E-69
C5orf34	1.297171721	2.91E-167
C5orf46	0.409658604	9.74E-24
C5orf47	0.09295779	3.42E-10
C5orf49	0.031006231	0.770556819
C5orf51	1.38080266	5.19E-113

C5orf52	-0.03718199	0.000299589
C5orf58	0.005752464	0.863552289
C5orf63	0.919785568	1.53E-164
C6	0.122311109	3.11E-07
C6orf118	1.468356417	1.67E-93
C6orf120	1.753094918	3.64E-181
C6orf132	-0.017744283	0.326951274
C6orf136	-0.227725257	0.002401257
C6orf141	0.769754241	8.94E-08
C6orf163	0.107386407	1.69E-06
C6orf226	1.77046663	2.49E-226
C6orf47	1.27758022	6.48E-109
C6orf52	1.700989937	1.10E-256
C6orf58	0.365031911	1.00E-93
C6orf89	1.380794871	2.24E-126
C7	0.710890946	8.23E-25
C7orf26	2.091576198	3.26E-223
C7orf31	1.478505783	3.01E-131
C7orf33	0.097027067	2.07E-122
C7orf50	0.905881689	4.38E-80
C7orf57	1.704336789	6.57E-118
C7orf61	0.975928473	1.34E-56
C8A	-0.00387957	0.285844761
C8B	-0.000203341	0.966929628
C8G	0.40598931	9.50E-35
C8orf33	1.518140714	1.22E-201
C8orf34	0.261782499	2.14E-07
C8orf37	1.694439532	9.33E-250
C8orf48	1.630751405	8.96E-228
C8orf58	1.139721794	3.19E-47
C8orf74	0.004875656	6.07E-06
C8orf76	-0.650635607	1.30E-30
C8orf82	1.274506515	4.90E-156
C8orf88	1.368057776	3.20E-135
C8orf89	0.289701029	8.55E-52
C9	0.0631453	1.27E-15
C9orf116	0.830319538	3.61E-39
C9orf131	-0.170499457	1.07E-17
C9orf135	0.448934634	1.21E-10
C9orf152	0.098130929	6.09E-21
C9orf153	0.150935377	6.99E-94
C9orf16	0.609918493	8.23E-17
C9orf24	-1.231535157	3.61E-45
C9orf40	1.085156498	4.40E-81
C9orf43	0.247974125	4.86E-21
C9orf50	0.6953053	5.48E-91
C9orf57	0.01674996	0.156502689
C9orf72	0.196395075	0.062638556

C9orf78	0.970253497	1.13E-88
C9orf85	1.046912692	8.91E-157
CA1	-0.044328665	0.075975864
CA10	-1.536582309	4.55E-24
CA11	-2.302930507	9.10E-109
CA12	2.687300521	1.68E-76
CA13	1.169057592	1.32E-117
CA14	1.351644988	3.26E-46
CA2	2.28629194	3.27E-121
CA4	-0.782582743	1.07E-07
CA5B	0.563469853	4.48E-36
CA6	0.116507826	1.66E-32
CA7	-1.680561912	5.76E-55
CA8	-0.06214253	0.550358973
CA9	2.987280577	1.57E-73
CAAP1	1.327564785	2.30E-137
CAB39	0.991594472	2.54E-47
CAB39L	0.44699517	4.33E-15
CABIN1	0.903873361	3.11E-24
CABLES1	-1.674158965	5.11E-67
CABLES2	1.089944455	5.32E-35
CABP1	-3.110183755	1.56E-86
CABP2	0.035050608	2.64E-26
CABP4	1.120445952	8.96E-280
CABP5	-0.002260417	0.862364594
CABP7	-0.271626572	0.015017228
CABS1	-0.010535286	0.018687979
CABYR	-0.139247153	0.021580536
CACFD1	1.708104823	1.56E-199
CACHD1	1.222687095	1.76E-59
CACNA1A	-3.116607672	1.71E-82
CACNA1B	-2.844225597	2.35E-121
CACNA1C	-0.726385892	4.01E-25
CACNA1D	-0.528349881	1.33E-15
CACNA1E	-1.657673022	1.62E-56
CACNA1F	-0.387080406	1.11E-19
CACNA1G	-0.05047356	0.69388088
CACNA1H	-0.52750582	3.37E-06
CACNA1I	-2.273187481	1.49E-104
CACNA1S	-0.097923448	7.80E-05
CACNA2D1	-0.595636629	8.06E-13
CACNA2D2	-1.669468535	2.09E-47
CACNA2D3	-1.616025228	1.05E-49
CACNA2D4	1.300990858	7.86E-264
CACNB1	-1.409550254	1.18E-50
CACNB2	-1.500753118	2.04E-49
CACNB3	-1.078623197	3.53E-20
CACNB4	-1.647789473	4.24E-51

CACNG1	0.804939676	3.33E-48
CACNG2	-1.665491865	3.02E-58
CACNG3	-2.189867695	1.38E-40
CACNG4	1.495715234	1.88E-56
CACNG5	0.780483178	9.54E-82
CACNG6	1.210006735	3.19E-172
CACNG7	0.330468455	2.16E-05
CACNG8	-1.183466865	1.49E-20
CACTIN	-0.049627372	0.442979793
CACUL1	0.680542349	5.30E-41
CACYBP	0.343072839	8.10E-06
CAD	1.674478855	1.72E-106
CADM1	1.339785897	7.24E-54
CADM2	-0.040130183	0.589372074
CADM3	-1.791196806	1.53E-42
CADM4	1.412855896	1.15E-132
CADPS	-0.98074187	3.75E-17
CADPS2	-0.098025185	0.574128427
CAGE1	0.118473296	1.46E-264
CALB1	-2.980286376	1.35E-61
CALB2	-2.797649222	1.13E-62
CALCA	-0.077723524	0.034654378
CALCB	-0.066514747	0.162861442
CALCOCO1	0.177395045	0.059619331
CALCOCO2	1.068940687	1.97E-91
CALCR	-0.047796327	0.426577666
CALD1	2.026258139	1.13E-252
CALHM1	-0.707977433	6.04E-24
CALHM2	1.922818238	1.56E-273
CALHM3	-0.122673145	1.74E-18
CALM1	-0.895346366	7.32E-26
CALM2	-1.336955298	2.94E-82
CALM3	-1.303731914	2.63E-33
CALML3	-0.235043682	6.82E-44
CALML4	-1.729746696	9.75E-151
CALML5	-0.534778585	8.59E-29
CALML6	-0.399952308	3.40E-14
CALN1	-2.535535047	2.35E-83
CALR	2.753957767	2.48921840032599e-314
CALR3	0.035940603	1.46E-07
CALY	-3.801179416	6.59E-176
CAMK1	-2.698371505	4.75E-220
CAMK1D	-0.563746849	1.27E-06
CAMK1G	-2.151133123	4.83E-44
CAMK2A	-2.852011808	2.79E-43
CAMK2B	-2.089262669	3.57E-60
CAMK2D	2.024399181	4.44E-72
CAMK2G	-0.421089854	9.92E-08

CAMK2N1	-0.316124321	0.003625995
CAMK2N2	0.668784817	9.66E-09
CAMK4	-2.087130891	1.27E-40
CAMKK1	-2.055642685	1.76E-51
CAMKK2	-0.963438391	2.63E-09
CAMKMT	0.491676152	1.29E-28
CAMKV	-2.093962723	1.19E-27
CAMLG	-0.479624354	2.44E-14
CAMP	0.715939533	1.08E-65
CAMSAP1	0.432231454	2.27E-07
CAMSAP2	0.806749986	3.39E-33
CAMSAP3	-1.597783512	9.53E-45
CAMTA1	0.162217613	0.043029919
CAMTA2	-1.309169673	1.42E-38
CAND1	1.486289649	3.98E-86
CAND2	0.536018511	9.25E-13
CANX	2.303940206	1.09E-268
CAP1	2.358461157	2.00E-294
CAP2	0.441507327	0.000346341
CAPN1	0.883337764	6.47E-33
CAPN10	-0.33153855	1.69E-06
CAPN11	0.165847848	1.05E-24
CAPN12	-0.002796622	0.874073583
CAPN13	-0.365740924	2.41E-27
CAPN14	0.194581789	1.34E-42
CAPN15	0.458354163	2.28E-06
CAPN2	1.752340049	2.62E-158
CAPN3	-3.620913258	1.60E-241
CAPN5	2.750064642	1.60E-248
CAPN6	0.788467188	8.39E-142
CAPN7	1.14274372	2.04E-55
CAPN8	0.07841491	1.50E-05
CAPN9	0.378111638	5.46E-19
CAPNS1	1.829901389	4.94E-203
CAPNS2	0.032414724	0.014992908
CAPRIN1	2.013768281	3.08E-194
CAPRIN2	-0.183569545	0.086061991
CAPS	0.951782679	6.09E-23
CAPS2	0.262729117	1.04E-05
CAPSL	0.059175099	0.5491653
CAPZA1	2.227596772	2.68E-286
CAPZA2	2.295388378	2.04E-295
CAPZA3	0.031796764	0.001525542
CAPZB	-0.45969388	2.42E-16
CARD10	0.805571901	7.85E-78
CARD11	1.474285229	3.96E-142
CARD14	-0.215580093	7.47E-10
CARD18	-0.035459265	0.001284133

CARD19	0.705220636	1.12E-15
CARD8	1.420448215	2.62E-192
CARD9	0.527285139	1.10E-18
CARF	0.295378578	1.79E-07
CARHSP1	1.371019216	7.99E-83
CARM1	0.775505796	2.68E-19
CARMIL1	0.283611645	0.000188721
CARMIL2	-3.030242984	1.30E-213
CARMIL3	-0.887399797	2.26E-12
CARNMT1	0.512203625	1.95E-10
CARNS1	-2.392921931	3.13E-63
CARS2	0.20205499	0.00111021
CARTPT	-2.245510123	8.66E-26
CASC3	0.436705193	4.21E-19
CASD1	0.259830782	0.000343726
CASK	1.194101242	3.35E-93
CASKIN1	-2.614021048	1.07E-131
CASKIN2	0.648741159	2.24E-44
CASP1	2.824167201	3.84E-288
CASP10	0.8817874	2.52E-102
CASP14	-0.109917804	1.98E-10
CASP2	1.659094754	4.83E-118
CASP3	2.645212405	3.48E-270
CASP4	2.150745043	1.51E-201
CASP7	1.664770912	4.46E-170
CASP8AP2	0.866730676	2.58E-44
CASP9	1.114921463	1.59E-95
CASQ1	1.500029414	7.20E-51
CASQ2	-0.486731963	1.01E-15
CASR	-0.005228509	0.520812608
CASS4	1.054263455	4.89E-216
CAST	0.978411072	3.73E-68
CASZ1	0.492906225	1.18E-70
CAT	2.213154662	3.64E-228
CATIP	-0.031111481	0.621591254
CATSPER1	0.781169669	1.00E-260
CATSPER2	-0.997994989	1.38E-25
CATSPER3	0.09738177	0.000526451
CATSPER4	-0.043741489	3.26E-06
CATSPERB	0.179925113	5.17E-173
CATSPERD	0.119542604	2.42E-07
CATSPERG	-0.192106802	0.025767543
CAV2	2.027883548	1.59E-197
CAV3	0.159222042	4.88E-24
CBARP	0.414615654	2.67E-06
CBFA2T2	0.529277608	2.06E-16
CBFA2T3	-2.024713729	1.40E-58
CBFB	2.107782186	1.47E-256

CBL	1.187169396	1.05E-97
CBLB	0.509913545	4.87E-07
CBLC	0.112671495	2.80E-17
CBLL1	1.765838341	1.04E-160
CBLN1	-2.474496615	3.71E-23
CBLN2	-1.184167382	2.00E-15
CBLN3	-1.360863273	1.36E-08
CBLN4	-0.193690202	0.213057476
CBR1	1.195246931	4.69E-72
CBR3	0.37017524	5.38E-09
CBR4	0.969809471	1.68E-64
CBS	0.319464198	3.52E-06
CBSL	0.426654158	5.41E-11
CBWD1	0.574756187	9.29E-48
CBWD2	-0.359802009	2.75E-08
CBWD3	-0.233453971	5.83E-10
CBWD5	-0.31761688	4.05E-09
CBX1	1.324893477	3.25E-109
CBX3	2.414764964	3.04E-302
CBX4	1.289140292	1.09E-91
CBX5	1.651466993	2.62E-159
CBX6	-0.81443768	1.48E-22
CBX7	-3.171745147	9.08E-237
CBY1	0.473167134	9.66E-33
CBY3	-0.489918436	6.11E-34
CC2D1A	0.096380193	0.230379598
CC2D1B	0.486691767	7.97E-15
CC2D2A	1.118810596	3.41E-159
CC2D2B	0.019868668	0.234080894
CCAR1	0.536764387	7.97E-17
CCAR2	0.662643079	4.97E-21
CCBE1	-0.286273991	8.89E-10
CCDC103	0.098930642	5.28E-11
CCDC105	-0.028822193	4.33E-09
CCDC106	0.434017677	8.69E-12
CCDC107	-0.572588491	3.04E-17
CCDC110	-0.167476495	0.000543474
CCDC112	0.987690772	6.28E-60
CCDC113	0.618286102	2.83E-24
CCDC115	-0.044274505	0.46878978
CCDC116	0.180332718	8.42E-19
CCDC117	1.009851302	4.16E-57
CCDC12	-0.136444889	0.021417611
CCDC120	0.010252999	0.918254724
CCDC121	1.12324656	2.47E-132
CCDC122	0.533061292	7.37E-71
CCDC124	1.006165385	2.27E-85
CCDC125	1.00407907	1.67E-104

CCDC126	1.608845715	9.92E-149
CCDC127	1.096496731	1.87E-91
CCDC13	-1.197297663	1.55E-123
CCDC130	1.134409406	2.45E-59
CCDC134	1.266373706	1.52E-78
CCDC136	-0.484856384	2.43E-05
CCDC137	1.798518042	2.84E-200
CCDC138	1.219452349	1.70E-141
CCDC14	1.47067759	1.36E-119
CCDC141	0.168407746	8.72E-50
CCDC142	0.921075804	3.57E-41
CCDC144A	-0.458153308	1.38E-13
CCDC146	0.659584603	9.62E-29
CCDC148	0.21040193	9.72E-11
CCDC149	-0.612623533	9.14E-25
CCDC150	0.565061625	5.63E-116
CCDC152	0.226959771	0.000626631
CCDC153	0.440932305	3.66E-13
CCDC154	-0.516247719	1.89E-12
CCDC157	-0.248850258	5.81E-06
CCDC158	0.184991339	5.60E-19
CCDC159	0.057499084	0.315542139
CCDC160	1.410991464	1.98E-162
CCDC163	1.175287865	1.52E-61
CCDC166	-0.07837865	1.08E-11
CCDC168	0.006654121	0.001204002
CCDC169	0.82662233	3.61E-288
CCDC17	-0.217124884	0.000129135
CCDC170	0.788434477	1.95E-41
CCDC171	-0.002537074	0.95583986
CCDC172	-0.018310948	0.000180764
CCDC173	0.944139171	3.43E-42
CCDC174	1.151059397	9.28E-78
CCDC175	-0.716715479	6.40E-10
CCDC177	-0.849213504	6.78E-20
CCDC178	0.431027145	9.66E-118
CCDC179	0.007971086	0.075513306
CCDC18	0.776578754	1.27E-102
CCDC180	-1.869032747	1.31E-124
CCDC181	0.497351068	1.26E-19
CCDC183	-0.939214296	1.85E-18
CCDC184	0.187601474	0.102806114
CCDC185	0.086778502	2.10E-19
CCDC186	-0.187822913	0.006750363
CCDC187	-0.039356459	0.027541215
CCDC188	0.415745766	6.64E-08
CCDC189	-0.892895605	3.09E-25
CCDC190	0.167373077	1.78E-14

CCDC191	0.310996364	1.38E-09
CCDC192	0.401347258	1.28E-172
CCDC22	1.3957846	1.21E-81
CCDC24	0.174837269	0.003054722
CCDC25	0.83676271	4.66E-50
CCDC27	0.071177979	7.68E-34
CCDC28A	0.367121806	5.82E-08
CCDC28B	1.458161489	3.04E-99
CCDC3	1.060888397	1.29E-27
CCDC30	-0.261162842	7.50E-09
CCDC33	0.02009323	0.721582892
CCDC34	1.900306654	1.09E-203
CCDC38	0.09983295	3.72E-52
CCDC40	0.742759296	5.02E-30
CCDC42	0.15338107	2.48E-108
CCDC43	0.710981445	1.09E-36
CCDC47	1.724705341	4.31E-135
CCDC50	1.856491735	5.63E-305
CCDC51	1.765804548	6.14E-246
CCDC54	-0.008823772	0.611273411
CCDC57	0.089485364	0.325216695
CCDC59	0.946053925	8.52E-69
CCDC6	0.207109383	0.005077742
CCDC60	-0.144860076	0.002491333
CCDC61	0.810165819	2.32E-38
CCDC62	-0.008534264	0.780721026
CCDC63	0.049274171	5.88E-08
CCDC65	0.842647415	1.52E-38
CCDC66	-0.269749231	0.000209386
CCDC68	-0.383912806	5.45E-17
CCDC69	0.207890005	6.75E-05
CCDC7	-0.137924158	0.001763034
CCDC70	0.128618457	5.33E-40
CCDC71L	1.779622583	1.38E-190
CCDC73	-0.096355356	5.99E-18
CCDC74A	0.927785137	9.18E-35
CCDC74B	0.650829194	9.78E-24
CCDC77	1.186751443	5.16E-109
CCDC78	-1.368673142	3.22E-43
CCDC8	1.711101947	1.01E-144
CCDC81	0.373620929	1.66E-12
CCDC82	-1.05631029	7.02E-49
CCDC83	0.054090734	2.63E-20
CCDC85A	-2.429155307	1.24E-139
CCDC85B	-0.090306378	0.232209064
CCDC85C	-0.536855654	2.53E-09
CCDC86	0.826905726	4.26E-47
CCDC87	0.588042134	1.26E-134

CCDC88A	-0.421382167	6.42E-14
CCDC88B	-0.234336912	0.119250499
CCDC88C	-0.105648798	0.299314745
CCDC89	1.413548879	2.39E-136
CCDC9	0.835512369	4.48E-53
CCDC90B	1.792601749	2.03E-208
CCDC91	0.57869228	6.45E-28
CCDC92	-0.279019266	9.37E-06
CCDC93	0.71584864	1.01E-21
CCDC96	0.392698246	1.41E-09
CCDC97	2.034971091	5.86E-278
CCER1	0.00130067	0.565320496
CCER2	1.230390399	1.46E-88
CCHCR1	0.833511766	3.27E-34
CCIN	0.772299311	6.07E-239
CCK	-1.754777153	1.73E-12
CCKAR	0.31734282	6.69E-22
CCKBR	-1.676795236	3.72E-34
CCL1	0.041914197	0.019657639
CCL11	0.156366752	2.20E-25
CCL13	0.648580828	3.98E-142
CCL14	-0.091339396	1.09E-09
CCL15	-0.020333908	0.028268307
CCL16	0.027583369	1.16E-18
CCL17	0.784251691	1.14E-199
CCL18	1.639358815	5.35E-234
CCL19	-0.056689862	0.575634889
CCL2	4.515053508	7.41E-229
CCL20	1.496979161	5.68E-119
CCL21	-0.14199442	2.42E-07
CCL22	0.571989821	2.45E-235
CCL23	0.579084081	1.69E-225
CCL24	0.314209968	1.93E-50
CCL25	0.790053187	6.38E-119
CCL27	-1.300693957	6.31E-137
CCL28	-0.13538487	0.000442864
CCL3	1.266297137	7.71E-36
CCL3L3	1.814190584	1.38E-97
CCL4	2.672822274	2.20E-167
CCL4L2	2.298441079	6.29E-168
CCL5	2.613594046	9.98E-229
CCL7	1.040967437	1.37E-178
CCM2	-0.53208954	6.04E-18
CCM2L	-0.374638958	1.52E-15
CCNA1	-0.301092371	0.004932096
CCNA2	2.982290714	7.50E-276
CCNB1IP1	1.436975813	4.43E-132
CCNB3	0.149514207	3.28E-06

CCNC	0.703417972	1.12E-22
CCND1	1.793400331	1.09E-145
CCND2	2.720864584	6.99E-241
CCND3	1.301401903	4.77E-145
CCNDBP1	-0.677600949	1.32E-18
CCNE1	1.194025717	1.63E-61
CCNE2	0.852807112	1.97E-32
CCNF	1.11813754	2.20E-44
CCNG1	1.692366883	1.65E-181
CCNG2	0.363902139	0.000277243
CCNH	0.386120502	4.41E-10
CCNI	1.168502108	9.87E-46
CCNI2	-0.846253736	1.59E-31
CCNJ	1.065406751	1.31E-71
CCNJL	0.08416716	0.552758396
CCNK	-0.308347122	2.05E-07
CCNL1	-0.208523116	0.015587579
CCNL2	-0.028794349	0.790691883
CCNO	-0.458525321	4.91E-06
CCNT1	1.504966652	3.57E-125
CCNT2	0.618722343	3.34E-14
CCNY	0.631607138	2.65E-31
CCNYL1	1.888463865	1.17E-246
CCP110	-0.797174905	8.36E-33
CCPG1	-1.808036028	3.71E-120
CCR1	3.470897342	9.43452935329069e-318
CCR10	0.097442273	0.185111248
CCR3	0.114758344	1.49E-112
CCR6	-0.469218858	2.85E-60
CCR7	0.62753702	2.64E-103
CCR8	0.094178085	3.67E-108
CCR9	0.025300193	0.004201356
CCS	0.892240268	4.94E-52
CCSAP	0.561758099	3.69E-10
CCSER1	-0.354661392	3.70E-39
CCSER2	0.265461594	2.96E-05
CCT2	1.697458021	3.66E-112
CCT3	1.984464278	1.81E-179
CCT4	1.617122504	3.56E-98
CCT5	1.228996083	4.79E-57
CCT6A	2.327116518	3.78E-197
CCT6B	0.366878482	9.16E-13
CCT7	1.713882126	4.26E-113
CCT8	1.526704017	2.02E-81
CCT8L2	-0.016145201	7.16E-05
CCZ1	0.297582842	1.02E-06
CCZ1B	0.762381684	1.68E-44
CD109	1.79444281	8.74E-255

CD14	4.531699277	2.29E-294
CD160	0.231480658	1.50E-17
CD163	4.507876396	4.71E-297
CD163L1	0.867337549	1.69E-191
CD164	1.529401984	8.90E-111
CD164L2	-0.285514648	1.30E-12
CD177	-0.260654704	1.49E-07
CD19	0.366237102	4.92E-153
CD1A	0.324697953	6.71E-126
CD1B	0.082417789	4.38E-57
CD1C	0.468786022	1.19E-307
CD1D	1.127344512	4.50E-220
CD1E	0.304257793	4.40E-273
CD200	-0.080246603	0.426146873
CD200R1	0.387972305	5.33E-93
CD200R1L	-0.000656718	0.852259061
CD209	0.397748808	4.14E-12
CD22	-2.352051385	2.02E-88
CD226	0.46032661	1.78E-77
CD24	3.174787614	1.62E-105
CD244	0.73367549	5.46E-216
CD247	0.250647658	2.14E-10
CD27	0.328737173	1.27E-17
CD274	1.419485228	5.15E-135
CD2AP	0.744183486	8.12E-24
CD2BP2	1.572876687	4.85E-131
CD300A	3.196307512	9.00E-284
CD300E	0.618960325	5.53E-28
CD300LD	0.007987211	0.004692246
CD300LF	2.080150655	2.19E-238
CD300LG	0.228339683	1.54E-71
CD302	0.008079878	0.889697385
CD33	1.762921902	1.55E-186
CD34	1.553498184	2.82E-120
CD36	1.064587035	1.57E-62
CD37	2.510716797	1.13E-154
CD38	1.461081333	1.19E-71
CD3E	1.352403193	2.06E-146
CD3G	0.503815243	1.15E-252
CD40	1.386891832	5.39E-96
CD40LG	0.366463775	4.28E-220
CD44	5.208493113	2.75194564733574e-321
CD46	0.621469892	1.41E-22
CD47	0.910537231	5.45E-27
CD5	0.739208993	2.11E-134
CD52	1.732230393	5.05E-141
CD53	3.930343972	1.81E-249
CD55	0.131585635	0.065136527

CD59	-0.94530783	2.47E-68
CD5L	0.589906022	7.20E-176
CD6	0.240263997	1.35E-07
CD68	-3.240930738	5.17E-208
CD69	1.54757562	6.25E-84
CD7	1.080089261	7.57E-176
CD70	1.346868245	1.61161125269062e-315
CD72	1.494732403	3.27E-278
CD74	3.801972796	2.38E-180
CD79A	0.772721646	7.51E-210
CD79B	0.235817618	6.49E-16
CD80	0.455630126	2.57E-196
CD81	1.209182822	9.38E-125
CD82	2.050005605	1.09E-180
CD83	1.257384135	4.70E-36
CD86	3.176279999	5.36E-270
CD8A	0.193649851	0.001186354
CD8B	0.124137607	0.004020631
CD9	2.50403967	3.41E-145
CD93	3.666344438	2.72E-303
CD96	0.541006856	4.52E-169
CD99L2	0.646717018	6.04E-18
CDA	1.734188821	3.10E-148
CDADC1	0.659887636	3.37E-31
CDAN1	0.418340652	8.87E-08
CDC123	0.52748138	2.16E-17
CDC14A	0.826705298	2.05E-68
CDC14B	0.985602352	6.90E-66
CDC14C	0.370106176	3.69E-168
CDC16	0.959440966	4.66E-77
CDC20B	0.066305083	3.51E-12
CDC23	1.691673609	2.68E-140
CDC25B	1.615907541	1.67E-58
CDC26	1.879124083	3.85364749629903e-312
CDC27	1.278668781	1.76E-82
CDC34	1.354849249	7.50E-98
CDC37	0.797960039	6.02E-28
CDC37L1	0.560710232	1.12E-18
CDC40	0.511919131	2.07E-12
CDC42	0.348445282	3.44E-07
CDC42BPA	-0.157810628	0.012097119
CDC42BPB	-0.498685327	2.37E-18
CDC42BPG	-0.688911495	0.000128626
CDC42EP1	2.199801386	2.05E-109
CDC42EP2	-3.018766468	8.61809822517918e-317
CDC42EP3	1.071904856	9.32E-76
CDC42EP4	2.087080521	1.50E-178
CDC42EP5	1.311106317	5.39E-127

CDC42SE1	1.495928789	1.77E-102
CDC42SE2	1.326406979	5.46E-95
CDC5L	1.664881635	1.80E-153
CDC7	1.612190958	2.78E-64
CDC73	1.322510211	1.46E-129
CDCA3	1.510873507	4.43E-60
CDCP2	-0.133238662	1.64E-35
CDH1	0.888882853	3.37E-75
CDH10	0.696927511	3.85E-11
CDH12	-0.511709364	7.54E-23
CDH13	0.083260589	0.454217835
CDH15	-0.334924136	0.123888542
CDH16	-0.002145088	0.773959214
CDH17	0.1694973	2.89E-128
CDH18	-1.260103303	4.88E-24
CDH19	0.020960945	0.791098032
CDH2	1.97101618	3.79E-213
CDH20	0.283036275	1.61E-05
CDH22	-1.456336829	4.54E-21
CDH23	-0.755606243	2.06E-12
CDH24	1.38779525	1.87E-61
CDH26	0.019693671	0.601083908
CDH3	0.370963669	4.58E-13
CDH4	1.35282215	1.64E-50
CDH5	1.593834508	3.13E-120
CDH6	1.609543525	3.26E-147
CDH7	-0.644115179	8.28E-18
CDH8	-1.046795053	3.27E-30
CDH9	-1.102816768	7.94E-26
CDHR1	-0.50089692	5.78E-07
CDHR2	0.284341978	4.24E-15
CDHR3	-0.274770334	0.000414684
CDHR4	0.113391725	0.026577667
CDHR5	-0.084508776	0.000169967
CDIP1	0.066351814	0.303437392
CDIPT	0.32247479	4.83E-05
CDK10	0.184161983	0.007983309
CDK11A	-2.278833584	9.31E-150
CDK11B	0.942521609	1.23E-61
CDK12	1.08274228	4.53E-61
CDK13	-0.101170241	0.158715721
CDK14	1.331610946	1.01E-44
CDK15	0.396092351	1.71E-143
CDK16	0.611744175	2.56E-11
CDK17	0.328941608	5.66E-06
CDK18	-0.534316155	2.13E-06
CDK19	0.20830914	0.000490089
CDK20	0.342312468	3.74E-05

CDK2AP1	2.318414517	1.00602318735473e-316
CDK2AP2	1.313791405	2.60E-138
CDK3	-2.670058642	6.63E-306
CDK5	0.594645713	9.54E-13
CDK5R1	-0.544768584	5.42E-06
CDK5R2	-1.659441754	2.99E-34
CDK5RAP1	0.366849831	2.91E-08
CDK5RAP2	0.607703717	2.61E-25
CDK5RAP3	-3.49304273	5.87E-307
CDK7	1.221795246	1.05E-91
CDK8	1.040055554	7.87E-49
CDK9	0.43170776	1.02E-09
CDKL1	1.339649805	6.95E-230
CDKL2	-1.132736292	3.72E-51
CDKL3	-0.469458879	5.08E-33
CDKL4	0.1160528	7.18E-13
CDKL5	-0.951242759	2.52E-33
CDKN1A	3.339494846	2.39E-165
CDKN1B	1.119813356	5.52E-48
CDKN1C	0.512549059	2.11E-05
CDKN2AIP	1.180401122	2.04E-76
CDKN2AIPNL	1.663782858	2.54E-111
CDKN2B	1.954372244	4.52E-228
CDKN2D	-1.07501469	1.69E-38
CDNF	0.3469679	4.30E-15
CDO1	1.985279446	7.72E-157
CDON	0.173297291	0.163296825
CDPF1	1.663717034	8.26E-188
CDR1	-2.860020727	1.32E-151
CDR2	0.475033022	8.08E-09
CDR2L	0.456566912	0.000190546
CDRT1	-0.040152806	0.020706502
CDRT15	0.279000671	9.17E-73
CDRT15L2	-0.078708032	0.007870647
CDS1	-1.178279661	3.37E-35
CDS2	0.841537499	5.56E-29
CDSN	-0.017749776	0.217134218
CDV3	1.72780322	9.11E-203
CDX1	0.168668423	4.88E-22
CDX2	0.01572975	0.011610679
CDX4	-0.000140232	0.935336784
CDY1	-2.13E-05	0.968311055
CDY1B	0.000212771	0.674403649
CDY2A	0.001346573	0.014656299
CDY2B	-5.06E-05	0.924534524
CDYL2	-0.748714359	1.49E-14
CEACAM1	0.361609332	4.09E-33
CEACAM16	0.078995606	6.18E-52

CEACAM18	0.001710957	0.249747104
CEACAM19	-0.781495871	1.29E-24
CEACAM20	0.023034186	1.18E-08
CEACAM21	1.478591707	2.34E-112
CEACAM3	0.157906536	1.20E-48
CEACAM4	0.993995274	9.13E-204
CEACAM5	-0.029916008	0.008373921
CEACAM6	-0.055061392	0.000505838
CEACAM7	-0.011904246	0.080275251
CEACAM8	0.0157748	0.127944062
CEBPA	1.243408191	7.33E-85
CEBPB	1.824505451	1.17E-113
CEBPD	3.294287029	1.34E-202
CEBPE	0.44524587	1.07E-153
CEBPG	1.68564996	2.74E-145
CEBPZ	1.321297968	2.36E-94
CEBPZOS	0.933979759	1.64E-56
CECR2	0.015749803	0.893955573
CEL	-0.281660003	0.001369646
CELA2A	-0.249771378	1.26E-06
CELA2B	0.203469461	7.44E-18
CELA3A	-0.823259673	3.89E-19
CELA3B	-0.418702067	4.77E-13
CELF1	0.515240028	7.35E-09
CELF2	0.556594953	4.85E-11
CELF3	-2.704222155	1.39E-82
CELF4	-3.081797479	1.75E-127
CELF5	-2.229172851	3.02E-73
CELSR1	1.440238757	8.45E-244
CELSR2	0.787558551	5.33E-23
CELSR3	-0.554068589	5.92E-06
CEMIP	0.974076565	1.93E-38
CEND1	-1.366022278	1.33E-44
CENPB	1.533460813	3.38E-154
CENPC	0.671726433	1.72E-32
CENPJ	0.482860135	2.35E-07
CENPO	1.024603225	2.28E-97
CENPP	0.455148367	4.07E-26
CENPS	-1.347081736	1.06E-108
CENPT	-0.335169232	0.001389694
CENPV	0.081978663	0.274618115
CENPVL1	0.012554028	4.03E-05
CENPVL3	0.269350158	1.52E-09
CENPX	1.54230376	3.62E-110
CEP104	0.05841329	0.282877756
CEP112	0.782872622	2.82E-107
CEP120	-0.131221968	0.079302695
CEP126	-1.073853387	2.25E-47

CEP128	0.600975383	1.22E-91
CEP131	0.807113816	4.40E-32
CEP135	1.292461942	6.90E-175
CEP152	0.788796263	5.54E-100
CEP162	0.596374571	5.59E-36
CEP164	-0.230768439	0.003557202
CEP170	0.875096249	1.46E-23
CEP170B	-1.31836892	1.29E-31
CEP19	1.127235687	4.41E-68
CEP192	0.87374601	3.11E-37
CEP250	0.375250877	5.05E-09
CEP290	-0.977645134	2.71E-63
CEP295	0.09990281	0.156278033
CEP295NL	0.077351715	9.39E-10
CEP350	0.708375941	4.20E-30
CEP41	1.526122416	3.05E-178
CEP44	0.850522701	7.23E-39
CEP57	0.337694078	5.13E-06
CEP57L1	0.746694814	1.78E-89
CEP63	0.31434583	1.91E-07
CEP68	0.632704083	1.82E-17
CEP70	1.324117898	3.03E-144
CEP72	0.628499393	2.58E-24
CEP76	0.945988414	6.88E-58
CEP78	0.879790993	7.73E-61
CEP83	0.908027363	3.24E-101
CEP85	0.865528556	3.01E-31
CEP85L	0.274365919	3.31E-05
CEP89	1.717147551	1.13E-218
CEP95	-0.115322228	0.155122348
CEP97	0.492175579	1.60E-16
CEPT1	1.346296256	1.48E-80
CER1	0.352111044	1.54E-23
CERCAM	-1.062110385	2.59E-18
CERK	0.936457948	3.88E-25
CERKL	-0.35490282	0.00080751
CERS1	-0.748606731	4.79E-39
CERS2	0.993570877	2.10E-36
CERS3	-0.000578683	0.97438146
CERS4	0.992031659	1.13E-69
CERS5	0.82116446	2.11E-18
CERS6	0.538032425	3.57E-10
CES1	0.6738048	3.90E-37
CES2	0.489780447	1.22E-12
CES3	-0.246703731	4.16E-05
CES4A	-2.773554379	1.10E-145
CES5A	-0.242273827	2.38E-23
CETN1	-0.005889896	0.175595229

CETN2	2.055365353	1.32E-221
CETN3	1.390429212	2.28E-200
CFAP100	0.02165309	0.797908065
CFAP157	0.126718273	0.30078461
CFAP161	0.545850409	1.53E-23
CFAP20	0.936521109	2.90E-40
CFAP206	-0.760028585	2.38E-44
CFAP221	0.262146699	5.08E-07
CFAP36	1.272484729	7.01E-83
CFAP43	0.072152422	0.334998053
CFAP44	-0.076372448	0.288690548
CFAP45	0.263269741	0.007061031
CFAP46	-0.270087067	6.24E-05
CFAP47	-0.071642359	0.043077023
CFAP52	0.856694376	2.54E-22
CFAP53	0.566211724	1.16E-11
CFAP54	0.552181953	2.80E-49
CFAP57	-0.116649578	0.067203552
CFAP58	0.007583903	0.881123601
CFAP61	0.934284948	9.91E-111
CFAP65	-0.096184525	0.09724338
CFAP69	-0.349083877	5.42E-10
CFAP70	-0.529547936	3.65E-15
CFAP73	0.444940019	4.11E-05
CFAP74	-1.178690037	5.25E-39
CFAP77	1.627234743	3.76E-87
CFAP97	2.080687563	2.29E-305
CFAP99	0.288045874	2.20E-09
CFB	-1.643326266	2.41E-124
CFC1	-0.744209395	9.04E-41
CFC1B	-0.023797685	0.000610177
CFD	2.114173492	2.08E-230
CFDP1	0.204204448	0.000355879
CFH	1.011222077	6.08E-40
CFHR1	0.064910393	3.40E-07
CFHR2	-0.003735906	0.422075196
CFHR3	0.02945111	1.26E-16
CFHR4	0.025392121	1.46E-07
CFHR5	0.041125422	3.74E-11
CFI	0.690027147	2.87E-20
CFL1	1.466521246	5.92E-116
CFL2	1.661823543	4.78E-137
CFLAR	0.189507798	0.045498766
CFP	-1.127295561	1.19E-76
CFTR	0.161396138	1.74E-08
CGA	-0.090212658	0.006257348
CGB1	-0.004689182	0.020352809
CGB2	-0.069786872	3.04E-06

CGB3	-0.002014835	0.652955467
CGB5	0.012472249	2.27E-06
CGB7	-0.018277927	0.146971746
CGB8	-0.000648769	0.786181336
CGGBP1	1.641347255	3.50E-124
CGN	-0.456329685	2.55E-05
CGNL1	0.325378913	1.64E-07
CGREF1	-0.79863739	4.89E-17
CGRRF1	0.758409038	3.24E-50
CH25H	2.011316423	4.86E-86
CHAC1	0.20078463	0.017393902
CHAD	-2.483512634	1.51E-109
CHADL	0.261203225	0.003656179
CHAF1A	2.037714264	1.49E-271
CHAF1B	1.793633	7.01E-129
CHAMP1	1.581000717	1.84E-99
CHAT	0.098418747	0.014237339
CHCHD1	1.345548887	8.62E-118
CHCHD10	0.347915751	1.09E-08
CHCHD3	2.377410325	8.20148972096469e-321
CHCHD4	0.97580028	2.57E-67
CHCHD6	1.128150012	6.62E-58
CHCHD7	-0.145624197	0.012567246
CHD1	0.992984869	1.08E-34
CHD2	0.17637972	0.008900113
CHD3	-0.321816089	0.000267847
CHD4	-2.020539705	1.84E-217
CHD5	-2.87527497	2.31E-83
CHD6	0.845406629	2.44E-39
CHD7	0.666109677	1.96E-08
CHD8	-0.031032487	0.702809358
CHD9	1.148180852	2.61E-59
CHDH	0.343530034	4.97E-08
CHERP	0.619054223	4.82E-14
CHFR	0.819466125	1.17E-50
CHGA	-1.928925233	1.46E-24
CHGB	-1.833332325	1.00E-19
CHI3L1	5.800292923	5.87E-213
CHIA	0.022742862	0.00017824
CHIC1	-0.323830777	3.01E-06
CHIT1	1.209788746	1.81E-197
CHKA	-0.227667451	4.45E-05
CHL1	1.470182125	2.99E-57
CHM	0.980481789	3.84E-49
CHML	0.812460739	1.04E-41
CHMP1A	0.113896933	0.031810539
CHMP1B	1.152186466	4.00E-71
CHMP2A	1.638625956	3.06E-198

CHMP2B	-0.053331059	0.437512587
CHMP3	0.46480459	1.49E-11
CHMP4B	1.369570612	9.05E-124
CHMP4C	0.261790907	1.40E-21
CHMP5	1.523672382	2.79E-129
CHMP6	1.635566785	4.49E-200
CHMP7	1.301857589	6.35E-85
CHN1	-1.860748145	2.24E-43
CHN2	-3.157318756	5.80E-95
CHODL	1.669793679	1.52E-93
CHORDC1	-0.196115545	0.004104744
CHP1	1.158372133	1.53E-61
CHP2	-0.529003707	1.15E-22
CHPF	2.537490282	3.49E-270
CHPF2	2.74845273	1.29E-277
CHPT1	1.489345169	1.01E-174
CHRAC1	1.915580095	1.18E-279
CHRD	-1.176743349	3.88E-12
CHRDL1	0.483013299	1.74E-05
CHRFAM7A	0.312193503	5.47E-72
CHRM1	-1.601829931	7.97E-18
CHRM2	-0.261485316	9.44E-12
CHRM3	0.221982374	0.02306146
CHRM4	-0.297563729	0.023352914
CHRM5	-0.473599517	9.13E-14
CHRNA1	1.818464978	1.49E-226
CHRNA10	0.155933423	0.002666769
CHRNA2	-0.684945734	3.01E-18
CHRNA3	-0.236581295	0.007858809
CHRNA4	-1.434158055	5.78E-77
CHRNA5	0.491760948	5.86E-88
CHRNA6	0.234478902	4.83E-06
CHRNA7	0.045643975	0.028488698
CHRNB1	1.221850563	1.82E-179
CHRNB2	-0.887180624	5.90E-16
CHRNB3	-0.358663753	9.92E-10
CHRNB4	0.22391383	2.65E-74
CHRND	0.224833642	3.65E-61
CHRNE	-0.160573811	0.001180675
CHRNG	-0.013787771	0.000782674
CHST1	-0.99505908	2.11E-17
CHST10	1.600084886	3.11E-150
CHST12	1.065383167	7.30E-85
CHST13	0.537429934	1.16E-89
CHST15	1.327107077	4.98E-50
CHST2	1.731135366	1.28E-87
CHST3	1.901676861	1.15E-138
CHST4	0.261165456	5.58E-163

CHST5	-1.416856532	2.92E-114
CHST6	1.458201665	2.52E-94
CHST7	1.771445225	1.13E-166
CHST8	0.195008035	0.094131788
CHSY1	2.130608605	2.17E-281
CHSY3	1.071923001	6.18E-60
CHTF18	0.417605316	9.78E-06
CHTOP	1.366732061	4.05E-95
CHUK	0.875077554	5.02E-56
CHURC1	1.178925271	3.76E-87
CIAO1	1.72339194	4.03E-127
CIAPIN1	1.734469028	2.64E-120
CIART	-0.120181858	0.225329212
CIB1	1.732133201	1.47E-183
CIB2	1.547261855	1.56E-179
CIB3	0.204797532	3.71E-135
CIB4	-0.062192726	0.001912624
CIC	0.562121089	9.56E-10
CIDEA	-0.773440375	9.58E-22
CIDEC	-0.088130106	0.000396553
CIITA	1.288079195	3.76E-108
CILP	0.489387715	2.05E-36
CILP2	0.178067108	8.09E-10
CINP	0.998165072	4.10E-82
CIPC	-0.226130479	0.000928259
CIR1	0.525464562	1.87E-23
CIRBP	0.59383585	3.23E-12
CISD1	0.657537483	9.70E-19
CISD2	-0.030743098	0.643687389
CISD3	-0.140596521	0.004944831
CISH	1.370878606	2.71E-107
CIT	-1.953505668	1.83E-102
CITED1	-0.980112193	1.13E-23
CITED2	2.178489912	1.20E-67
CITED4	0.179436867	0.005189105
CIZ1	0.862640808	1.17E-37
CKAP2	2.734487239	7.35E-224
CKAP5	1.07395582	1.46E-55
CKB	-0.012139402	0.883313731
CKM	-0.97392824	2.37E-21
CKMT1A	-1.968334971	1.05E-70
CKMT1B	-3.426981947	2.30E-92
CKMT2	-0.337081534	3.39E-08
CLASP1	0.529028486	1.84E-11
CLASP2	-0.369929466	1.30E-05
CLASRP	-0.277349214	0.000294887
CLC	0.111768248	0.000625691
CLCA1	-0.016913457	0.029018672

CLCA2	0.006476031	0.821524281
CLCA4	-1.09947006	1.90E-37
CLCC1	-0.19686435	0.000752354
CLCN1	0.209268034	6.96E-63
CLCN2	0.067287013	0.39438195
CLCN3	1.46790154	1.79E-121
CLCN4	-0.647176246	4.01E-13
CLCN5	0.783649599	2.55E-28
CLCN6	-0.007128057	0.927611455
CLCN7	0.566921403	1.87E-12
CLCNKA	-0.386353694	0.000111723
CLCNKB	-0.132732977	0.223568272
CLDN1	0.72292524	3.57E-50
CLDN10	-0.704689581	2.23E-14
CLDN11	0.392597708	0.003288291
CLDN12	1.636700089	2.56E-107
CLDN14	0.110299895	7.50E-90
CLDN15	1.21810272	2.67E-75
CLDN16	-0.199873934	9.26E-24
CLDN17	-0.002481054	0.522448902
CLDN18	0.037474643	0.088781071
CLDN19	0.000657597	0.980624818
CLDN2	0.065593221	1.70E-06
CLDN20	0.494010927	1.10E-57
CLDN23	1.444795237	9.43E-291
CLDN24	0.024010174	2.23E-07
CLDN25	0.004266109	0.240768317
CLDN3	-0.546006068	6.60E-07
CLDN34	0.066169177	6.17E-11
CLDN4	0.340341275	4.95E-16
CLDN5	1.082429949	1.62E-52
CLDN6	-0.169947962	2.11E-05
CLDN7	1.080999058	9.59E-248
CLDN8	-0.010255449	0.001044286
CLDN9	-1.577723046	9.31E-35
CLDND1	-0.123119869	0.239201646
CLDND2	0.289267667	3.43E-12
CLEC11A	1.313655346	2.71E-96
CLEC12B	-0.020190064	0.089419747
CLEC14A	1.756278058	2.67E-184
CLEC16A	0.126008068	0.059946281
CLEC17A	1.592148293	1.99E-287
CLEC18A	0.354563142	6.15E-53
CLEC18B	2.139072627	1.00E-268
CLEC18C	0.375132211	1.55E-119
CLEC19A	0.292607681	3.59E-44
CLEC1A	0.936132019	2.10E-81
CLEC1B	0.080460531	1.11E-117

CLEC2A	-0.089602658	1.50E-06
CLEC2B	1.410507424	4.09E-81
CLEC2D	0.998334612	3.20E-144
CLEC2L	-3.101875789	6.47E-132
CLEC3A	0.04351019	0.022373057
CLEC3B	-1.144316198	1.33E-50
CLEC4A	-0.871385937	2.87E-91
CLEC4C	0.069857512	6.07E-48
CLEC4D	0.218678214	2.28E-60
CLEC4E	1.613000311	4.62E-161
CLEC4F	0.517467662	7.55E-67
CLEC4G	-2.010359672	1.18E-62
CLEC4M	-0.175748934	6.80E-12
CLEC6A	0.085580888	2.98E-140
CLEC7A	1.905518748	3.95E-98
CLEC9A	0.278412551	2.28E-05
CLECL1	1.149968076	8.44308782178106e-320
CLGN	0.488728625	5.51E-07
CLHC1	1.309503649	7.22E-127
CLIC2	1.251321259	2.76E-147
CLIC3	0.408502433	2.23E-23
CLIC4	3.675428583	1.01E-288
CLIC5	-0.520355343	2.55E-36
CLIC6	0.319987379	5.46E-05
CLINT1	1.419835992	5.11E-130
CLIP1	-0.304008462	0.001389842
CLIP2	1.151562607	1.58E-96
CLIP3	0.180111667	0.045889759
CLIP4	-0.338717089	8.33E-08
CLK1	-0.774399444	2.04E-24
CLK2	1.186715555	2.29E-41
CLK3	0.390828054	1.21E-10
CLK4	-0.911825454	3.68E-12
CLMN	-0.54257299	7.53E-14
CLMP	0.222786441	0.038012242
CLN3	-2.066465812	9.98E-276
CLN5	0.333875288	4.79E-16
CLN6	-0.605878306	1.54E-25
CLN8	0.495192374	1.59E-17
CLNK	0.103281018	6.27E-92
CLNS1A	1.774338865	4.25E-292
CLOCK	0.671837394	4.10E-22
CLPB	1.472949603	5.32E-148
CLPP	0.968347108	2.25E-70
CLPS	-0.989110979	4.77E-23
CLPSL1	-0.255368209	1.14E-08
CLPSL2	0.153164431	7.15E-10
CLPTM1	1.102962357	1.66E-73

CLPTM1L	1.112892923	1.28E-88
CLPX	1.322538879	1.17E-141
CLRN1	0.095499324	3.88E-15
CLRN2	-0.045868363	5.26E-07
CLRN3	0.284053302	1.35E-32
CLSTN1	0.861537072	2.43E-18
CLSTN2	0.589415693	4.76E-08
CLSTN3	-0.326178623	0.029462969
CLTA	1.244098959	1.08E-78
CLTB	0.44627433	8.96E-11
CLTC	1.145574341	2.87E-46
CLTCL1	-0.009873061	0.883053164
CLU	2.194715575	4.28E-185
CLUAP1	0.546275597	7.90E-23
CLUH	0.583662643	1.33E-13
CLUL1	-0.385897627	5.25E-13
CLVS1	-0.905856956	1.26E-28
CLVS2	-1.378994019	2.04E-29
CLYBL	0.197812087	1.78E-06
CMA1	0.051365179	8.93E-24
CMAS	0.157617798	0.032976571
CMBL	1.012135486	1.81E-77
CMC1	0.378735261	9.98E-17
CMC2	0.085091372	0.0410095
CMIP	0.507562315	1.25E-11
CMPK1	1.247277934	1.61E-119
CMPK2	1.688549784	1.67E-114
CMSS1	1.389517915	6.36E-100
CMTM1	0.479453635	1.94E-19
CMTM2	0.300409965	1.95E-13
CMTM4	-0.799004741	4.97E-58
CMTM5	-0.272366767	0.010024216
CMTM7	1.733374661	2.21E-93
CMTM8	0.950912954	2.29E-74
CMTR1	1.475825924	2.16E-76
CMTR2	1.692186181	3.44E-242
CMYA5	0.423738018	2.80E-26
CNBD1	0.016173933	6.01E-23
CNBD2	0.313348548	9.00E-295
CNBP	0.780027621	5.21E-37
CNDP1	-1.485537394	5.91E-31
CNDP2	0.973925244	9.74E-73
CNEP1R1	1.479941234	3.99E-140
CNFN	-0.031459001	0.72962814
CNGA1	-0.031589264	0.04621603
CNGA2	0.017582807	8.84E-25
CNGA4	-0.342048436	1.28E-17
CNGB1	-0.817412777	6.53E-18

CNGB3	-0.006974561	0.521116654
CNIH1	2.041119215	5.23E-270
CNIH2	-0.462483317	1.70E-05
CNIH3	1.941432998	1.06E-171
CNKSR1	-1.000300481	3.63E-31
CNKSR2	-2.341441636	5.83E-66
CNKSR3	0.626205854	9.68E-59
CNMD	0.424303059	2.94E-08
CNN1	0.753096576	1.61E-27
CNNM1	-2.556594497	1.04E-117
CNNM2	-0.402408872	3.11E-13
CNNM3	0.900523935	4.38E-25
CNNM4	0.535076126	7.51E-08
CNOT1	1.186762545	1.96E-48
CNOT10	1.562910653	4.26E-152
CNOT11	1.774243612	8.07E-202
CNOT2	1.413012082	1.45E-64
CNOT3	0.945975698	3.50E-29
CNOT4	1.477464576	8.41E-150
CNOT6	1.142222266	2.03E-58
CNOT6L	0.924640602	6.52E-49
CNOT7	0.916229316	4.75E-35
CNOT8	1.590839005	1.34E-167
CNOT9	1.828138794	1.05E-161
CNP	0.260052481	0.009281984
CNPPD1	1.701425091	9.27E-144
CNPY1	-1.02571131	6.16E-13
CNPY2	-2.372635877	2.80360288078623e-313
CNR1	1.487004175	2.38E-32
CNR2	0.038479065	9.94E-65
CNRIP1	0.360530225	7.52E-06
CNST	0.339112004	0.000111831
CNTD1	0.096778097	0.001422164
CNTF	0.130262046	0.086519752
CNTFR	-0.149678632	0.088603751
CNTLN	1.056239361	3.34E-93
CNTN1	0.046070375	0.63685218
CNTN2	-2.022105725	9.95E-81
CNTN3	-1.04698357	2.40E-33
CNTN4	-1.265319037	4.27E-45
CNTN5	0.079296073	0.169924235
CNTN6	-0.80701199	2.34E-14
CNTNAP1	-0.386460034	0.00236786
CNTNAP2	-1.810967622	4.44E-79
CNTNAP3	0.015625408	0.725880657
CNTNAP3B	0.430615179	1.69E-101
CNTNAP4	-3.409221581	9.95E-200
CNTNAP5	0.150411016	2.73E-05

CNTRL	0.76287656	6.40E-32
CNTROB	0.952916849	9.87E-55
COA1	1.635776479	4.64E-185
COA3	0.812276446	7.69E-54
COA5	1.10594728	6.24E-97
COA6	0.141130482	0.040190128
COA7	1.641910879	2.71E-124
COASY	0.905496231	3.31E-49
COBL	-0.943246995	5.72E-64
COBLL1	0.288458759	6.46E-10
COCH	-1.192321502	5.87E-14
COG1	-0.241992119	0.012001177
COG2	0.032898093	0.676651126
COG3	0.77931826	3.99E-28
COG4	1.43542059	1.23E-114
COG5	1.623705748	6.25E-171
COG6	1.162685172	6.17E-78
COG7	0.642466773	8.95E-37
COG8	-0.507767167	6.88E-19
COIL	1.699166861	3.24E-129
COL10A1	0.202494064	7.68E-13
COL11A1	0.635481021	2.14E-16
COL11A2	-0.814402652	2.70E-16
COL12A1	1.196175811	2.11E-78
COL13A1	-0.73996367	5.59E-05
COL14A1	1.799687465	6.97E-251
COL15A1	1.40659651	1.75E-234
COL16A1	-0.056685104	0.462117774
COL17A1	-0.413789769	4.37E-20
COL18A1	1.172884799	9.82E-37
COL19A1	-0.161710814	0.002851804
COL20A1	1.487499118	1.16E-92
COL21A1	0.06891056	0.342847721
COL22A1	2.357128526	3.64E-203
COL23A1	0.385104718	6.92E-06
COL24A1	-0.542876927	3.95E-12
COL25A1	0.493805312	4.58E-19
COL26A1	-1.24439591	2.31E-28
COL27A1	-0.258603511	0.134804814
COL28A1	1.789052874	6.20E-140
COL2A1	0.518937255	4.97E-41
COL4A3	-0.20892856	3.21E-08
COL4A4	0.105909988	0.000575059
COL4A5	0.210755512	0.039953953
COL4A6	1.363841554	1.70E-294
COL5A1	2.561538096	7.91E-255
COL5A2	3.717463354	6.28E-239
COL5A3	0.599976775	6.97E-10

COL6A1	2.210903985	1.09E-73
COL6A5	0.009673517	0.376380783
COL6A6	-0.040263874	0.000531652
COL7A1	-0.97056717	4.13E-17
COL8A2	2.299712592	2.24E-107
COL9A1	-0.50970274	3.31E-17
COL9A2	0.023479435	0.815632545
COL9A3	-0.081994783	0.436062544
COLCA2	0.079047003	0.261912691
COLEC10	0.131503747	2.95E-33
COLEC11	0.608572334	3.87E-27
COLEC12	0.578803187	4.40E-22
COLGALT1	2.261509825	8.31E-147
COLGALT2	1.334392791	6.29E-75
COLQ	-0.961244533	7.78E-39
COMMD1	1.286505812	3.93E-131
COMMD2	2.133096602	6.88E-217
COMMD3	-0.905419382	2.34E-42
COMMD4	1.401710748	5.56E-163
COMMD5	1.93018962	3.97E-226
COMMD6	1.557603071	3.16E-201
COMMD7	1.467401322	1.94E-144
COMMD8	1.33684979	1.82E-138
COMMD9	1.665771509	1.12E-207
COMP	-0.24429712	0.008635595
COMT	1.185759412	4.81E-123
COMTD1	0.262179163	2.03E-05
COPA	1.214314283	4.13E-61
COPB1	2.253891572	3.14E-216
COPB2	0.611257524	2.67E-27
COPE	1.172107098	5.63E-131
COPG1	1.636770467	4.13E-99
COPG2	1.624929878	5.17E-120
COPRS	1.249676922	5.83E-83
COPS2	1.355453222	1.26E-96
COPS3	1.504602962	2.88E-90
COPS4	1.640196781	9.11E-110
COPS5	0.195016649	0.000979412
COPS6	1.20284639	2.07E-98
COPS7A	1.502592079	4.27E-100
COPS7B	0.446114197	4.49E-17
COPS8	1.615785332	1.82E-148
COPS9	1.050905616	7.88E-76
COPZ1	2.222194228	1.05E-290
COPZ2	2.451943883	5.18E-251
COQ10A	0.114143399	0.066177607
COQ10B	1.48029788	1.09E-146
COQ3	0.55677533	9.93E-16

COQ4	0.923157538	2.88E-49
COQ5	1.494352371	3.51E-172
COQ6	-0.917289156	2.34E-51
COQ7	1.417002762	9.16E-156
COQ8A	-3.229878009	3.31E-183
COQ8B	0.079011824	0.212274131
COQ9	0.345871016	6.47E-12
CORIN	-0.021846252	0.223937802
CORO1A	0.852493732	9.45E-23
CORO1B	1.69520971	6.03E-220
CORO1C	2.435209136	4.15E-292
CORO2A	-0.68313805	8.63E-11
CORO2B	0.780098114	1.52E-21
CORO6	-3.520118203	2.60E-191
CORO7	-1.023517767	2.28E-57
CORT	-2.359072701	1.04E-78
COX10	0.538561054	3.51E-21
COX11	0.303724589	7.19E-07
COX14	0.711123932	2.51E-31
COX15	0.385859918	3.46E-13
COX16	-1.189197338	9.52E-126
COX17	0.709676735	7.63E-30
COX18	1.435317951	1.59E-171
COX19	0.38382107	4.09E-09
COX4I1	0.013546166	0.80546407
COX4I2	1.361753711	4.20E-157
COX5A	1.370072619	8.78E-90
COX5B	0.450181983	9.61E-19
COX6A1	-1.819076121	4.74E-194
COX6A2	-1.076305592	5.89E-53
COX6B1	1.335443633	9.62E-125
COX6B2	-0.098028134	0.002508942
COX6C	0.363014336	7.51E-10
COX7A1	0.691762964	4.70E-21
COX7A2	0.856518567	2.26E-47
COX7A2L	0.922468334	1.07E-47
COX7B	1.584647373	2.05E-148
COX7B2	-0.004949758	0.532853468
COX7C	0.965005898	2.44E-75
COX8A	0.735879214	3.37E-40
COX8C	0.014709948	0.645720497
CP	1.805759307	6.81E-48
CPA1	-0.854188411	1.11E-16
CPA2	0.086374656	0.327704699
CPA3	0.38234901	5.45E-111
CPA4	1.03441201	2.55E-80
CPA5	0.466949506	2.29E-244
CPA6	0.428033284	3.90E-194

CPAMD8	-0.555983831	2.11E-12
CPB1	-0.41890554	5.72E-11
CPB2	-0.109071425	1.60E-20
CPD	1.534614288	1.05E-117
CPE	0.339192548	3.59E-05
CPEB1	-1.611304622	5.75E-95
CPEB2	0.419892188	4.38E-13
CPEB3	-1.451480631	1.81E-64
CPEB4	0.560622965	4.67E-17
CPED1	1.522968461	7.14E-263
CPLX1	-2.990544123	3.82E-114
CPLX2	-3.988773973	1.33E-134
CPLX3	-3.321520942	2.24E-73
CPLX4	-0.182317094	8.29E-05
CPM	2.408138206	1.15E-215
CPN1	0.009910565	0.000525783
CPN2	0.028867594	3.51E-12
CPNE1	1.438505726	1.05E-88
CPNE2	1.824487635	2.83E-163
CPNE3	2.28287046	1.00455487366185e-316
CPNE4	0.96227492	7.84E-15
CPNE5	0.206064518	0.145432971
CPNE6	-3.795430451	2.26E-279
CPNE7	-1.062429618	6.93E-16
CPNE8	0.407860024	1.23E-09
CPNE9	-1.993542388	1.18E-31
CPO	-0.456689135	7.08E-20
CPOX	0.390123783	3.53E-08
CPPED1	0.979141228	1.38E-49
CPQ	1.942169158	4.87E-216
CPS1	0.727583885	7.14E-26
CPSF1	0.54084747	1.22E-08
CPSF2	1.637207341	3.76E-297
CPSF4	1.308179262	5.36E-83
CPSF4L	0.381065714	3.09E-186
CPSF6	0.999431789	2.47E-27
CPSF7	0.884664658	1.06E-40
CPT1A	1.437506442	3.04E-118
CPT1B	-3.884747832	5.01E-245
CPT1C	0.143368342	0.092500951
CPT2	-0.012301357	0.7916256
CPTP	1.369131299	3.18E-134
CPVL	3.818907085	8.45E-195
CPXCR1	0.088783582	7.32E-36
CPXM2	-0.094962571	0.283332338
CPZ	0.010722642	0.449076999
CR1	0.444387343	3.20E-89
CR1L	0.097081813	2.30E-33

CR2	-0.04877167	6.02E-08
CRABP1	-2.564797059	5.70E-69
CRABP2	1.293761341	5.93E-48
CRACR2A	0.364210585	1.29E-227
CRACR2B	-0.364339094	2.54E-09
CRAMP1	-0.371892876	0.000520724
CRAT	1.517719043	7.79E-157
CRB1	1.364912409	1.32E-122
CRB2	1.945360458	2.04E-146
CRB3	-0.024459733	0.234452237
CRBN	-0.089439241	0.15150044
CRCP	1.834003065	1.29E-178
CRCT1	-0.179660856	1.01E-13
CREB1	1.223723188	1.15E-97
CREB3	1.510631149	8.21E-205
CREB3L1	1.7732252	1.30E-160
CREB3L2	2.267734886	2.19E-307
CREB3L3	-0.448003423	5.24E-27
CREB3L4	1.446827513	1.16E-87
CREB5	1.649571534	1.67E-109
CREBBP	0.746306965	1.46E-19
CREBL2	0.955730656	2.01E-51
CREBRF	0.129030673	0.019165395
CREBZF	0.338211865	0.000251352
CREG1	1.412522191	3.74E-111
CREG2	-0.942500261	4.82E-09
CRELD1	-3.452339513	1.23E-250
CRELD2	0.59846129	1.17E-24
CREM	1.164920504	1.32E-123
CRH	-1.231408114	4.27E-19
CRHBP	-0.508351836	6.91E-06
CRHR1	-2.80011568	8.86E-85
CRHR2	-0.619619881	1.73E-23
CRIM1	0.662643001	1.16E-28
CRIP1	-1.831019388	6.08E-143
CRIP2	1.5815139	8.95E-96
CRIP3	-1.357532824	1.12E-59
CRIPT	0.394001315	2.71E-14
CRISP1	0.002192416	0.025533895
CRISP2	-0.12351306	1.24E-09
CRISP3	-0.106736484	1.07E-07
CRISPLD2	0.652934143	4.82E-21
CRK	1.952144416	2.29E-247
CRKL	1.358069071	2.05E-100
CRLF1	-0.94911642	1.42E-12
CRLF2	0.068190779	6.57E-32
CRLF3	1.271625864	2.83E-86
CRMP1	1.599693131	9.66E-56

CRNKL1	1.948499489	6.99E-240
CRNN	-0.40600239	1.73E-18
CROCC	-0.363691051	0.000339514
CROCC2	-0.103826159	0.023363997
CRP	-0.232091532	3.50E-12
CRTAC1	-0.459394259	8.94E-08
CRTAM	-0.905073442	4.80E-05
CRTAP	1.83029466	7.74E-239
CRTC1	-0.631098201	1.37E-08
CRTC2	0.984042028	1.26E-38
CRTC3	0.865075183	1.03E-46
CRX	0.014575607	0.000216702
CRY1	1.687900717	1.63E-72
CRY2	-1.042381959	2.74E-33
CRYAA	0.017457038	3.61E-09
CRYAB	1.404478782	7.43E-42
CRYBA1	0.290799875	1.06E-73
CRYBA2	-0.103143764	0.017195956
CRYBA4	0.219026752	2.94E-16
CRYBB1	1.748691837	3.22E-206
CRYBB2	0.163204575	0.00060992
CRYBB3	0.092485055	3.95E-08
CRYBG3	0.597951848	6.82E-22
CRYGA	0.305842955	2.73E-28
CRYGB	-0.004215001	0.74827641
CRYGC	-0.011457797	0.167683014
CRYGD	0.132639598	0.047240644
CRYGN	-0.095430881	3.63E-05
CRYGS	0.212963203	0.013845842
CRYL1	0.293467094	9.09E-09
CRYM	-2.814586876	2.80E-51
CRYZ	2.465733484	2.36E-262
CRYZL1	0.553297014	1.50E-29
CS	0.594889997	4.30E-14
CSAD	-0.426839894	2.96E-07
CSAG1	1.26456995	1.65E-205
CSAG2	0.072230506	5.25E-16
CSAG3	0.237391239	3.31E-54
CSDC2	-1.318356922	1.43E-36
CSDE1	1.199077107	1.74E-103
CSE1L	2.010424348	3.03E-153
CSF1	2.784099294	6.47095068655885e-318
CSF1R	3.531263521	7.21E-193
CSF2	0.194232132	1.34E-102
CSF2RA	2.833867811	4.30E-258
CSF2RB	1.593024348	2.50E-212
CSF3	0.074855796	0.543330596
CSF3R	1.450281376	3.24E-61

CSGALNACT1	1.070276348	3.24E-36
CSGALNACT2	0.864297017	6.77E-43
CSH1	-0.002363304	0.386124609
CSH2	-0.040377045	0.000617542
CSHL1	-0.001094578	0.606396306
CSK	1.626128343	9.76E-151
CSMD1	-0.327234414	2.64E-08
CSMD2	0.828835468	3.66E-48
CSMD3	-0.820693256	4.55E-56
CSN1S1	-0.016456954	0.713796381
CSN2	-0.008948466	0.131007548
CSN3	0.0015159	0.643905033
CSNK1A1	0.257561008	3.19E-05
CSNK1A1L	0.267880811	1.14806082930356e-311
CSNK1D	0.270039336	8.88E-05
CSNK1E	0.073863242	0.273300855
CSNK1G1	0.990838687	1.02E-63
CSNK1G2	0.602401619	2.42E-16
CSNK1G3	1.446258842	3.36E-115
CSNK2A1	-0.390642028	1.29E-10
CSNK2A2	-0.278892175	7.89E-08
CSPG5	1.804842455	7.22E-118
CSPP1	-0.723143777	2.94E-33
CSRNP1	2.042567634	1.80E-149
CSRNP2	0.986779939	1.23E-37
CSRNP3	-0.024741973	0.755945774
CSRP1	-0.976819223	3.29E-45
CSRP2	1.830830792	7.95E-177
CSRP3	-0.030837685	0.280817657
CST1	0.297794352	5.36E-72
CST11	0.003208713	0.261912691
CST2	0.149513335	1.55E-65
CST3	1.596516318	1.69E-106
CST4	0.002999924	0.299708672
CST5	0.046207073	1.79E-24
CST6	0.514400673	1.20E-20
CST7	1.527360151	4.10E-134
CST8	-0.003503804	0.22028649
CST9	0.028470591	6.67E-38
CST9L	0.000812021	0.840209445
CSTB	1.212540552	6.26E-150
CSTF1	2.021141855	6.42E-221
CSTF2	1.857700287	5.07E-149
CSTF2T	1.256951582	1.30E-90
CSTF3	1.00982145	3.80E-63
CSTL1	0.019616881	0.000133129
CT45A1	0.006589204	0.651249335
CT45A10	0.00904533	0.710077625

CT45A2	-0.000131687	0.810755014
CT45A3	-0.001632798	0.276785263
CT45A5	0.008110107	0.000559085
CT45A6	0	1
CT45A7	-0.000219719	0.710512489
CT45A8	0	1
CT45A9	-5.19E-05	0.92370526
CT47A1	0	1
CT47A10	0	1
CT47A11	0	1
CT47A12	-2.13E-05	0.968693065
CT47A2	0	1
CT47A3	0	1
CT47A4	0	1
CT47A5	0	1
CT47A6	-2.50E-05	0.962986313
CT47A7	0	1
CT47A8	0	1
CT47A9	0	1
CT47B1	0.007053195	1.45E-06
CT55	-0.010417648	0.509714828
CT83	-0.002070735	0.451950329
CTAG1A	-0.006267911	0.081960803
CTAG1B	-0.00222027	0.18684424
CTAG2	-0.004578987	0.92451559
CTAGE1	0.063249717	8.53E-30
CTAGE15	0.009202045	2.49E-05
CTAGE4	0.010723678	0.006633044
CTAGE6	0.027626094	4.57E-26
CTAGE8	-0.001080916	0.709372575
CTAGE9	0.001976534	0.481872156
CTBP1	0.271036274	0.000133299
CTBP2	0.344950501	9.83E-07
CTBS	0.286829163	9.68E-10
CTC1	0.14765412	0.15571408
CTCF	0.261431882	9.49E-05
CTCFL	0.074810811	5.61E-75
CTDNEP1	0.038816857	0.430895743
CTDP1	1.627189132	4.76E-187
CTDSP2	1.644460711	2.64E-105
CTDSPL	0.681759661	3.06E-21
CTDSPL2	1.63711743	4.18E-110
CTF1	1.608088945	1.58E-181
CTH	1.47971961	1.00E-155
CTIF	-0.823006745	3.82E-32
CTLA4	0.583443007	3.28E-121
CTNNA1	1.778653531	1.53E-182
CTNNA2	0.444289874	1.59E-06

CTNNA3	-0.976002968	1.30E-24
CTNNB1	1.209910845	9.87E-59
CTNNBIP1	0.556908505	5.98E-14
CTNNBL1	1.990966446	5.28802167421444e-311
CTNND1	-2.190141229	2.06E-251
CTNND2	1.010988238	2.56E-68
CTNS	1.683819659	4.89E-249
CTPS1	1.050530116	4.17E-55
CTPS2	1.822303665	1.51E-166
CTR9	1.222935296	3.84E-96
CTRB1	-0.551977978	2.82E-14
CTRB2	-0.621721602	6.17E-15
CTRC	0.002112918	0.954829843
CTRL	-2.1836435	4.30E-200
CTSA	0.332070654	6.83E-07
CTSB	2.289438647	1.50E-216
CTSC	2.093915965	3.12E-175
CTSD	-0.355278053	1.34E-08
CTSE	-0.009284404	0.159444304
CTSF	0.071863177	0.379767887
CTSG	-0.047782563	0.052875168
CTSH	0.423732013	8.95E-10
CTSK	0.874195935	1.82E-30
CTSL	1.50172068	1.12E-115
CTSO	2.609855665	1.93E-294
CTSV	0.667756593	1.86E-45
CTSW	1.237274451	1.13E-180
CTSZ	1.962316957	1.28E-151
CTTN	0.011848056	0.871669283
CTTNBP2	0.347263908	1.28E-08
CTTNBP2NL	2.008886182	2.21521639543825e-316
CTU1	0.877115745	3.09E-49
CTU2	1.142833854	8.21E-80
CTXN1	0.358806401	0.115183938
CTXN2	-0.648591472	4.75E-54
CTXN3	-1.308268128	3.39E-23
CUBN	0.791873479	9.48E-102
CUEDC1	1.259532992	2.79E-110
CUEDC2	1.405695025	4.97E-127
CUL1	1.267863547	8.14E-73
CUL2	0.884860749	5.13E-43
CUL3	0.811639526	2.02E-35
CUL4A	1.423655381	1.07E-99
CUL4B	1.315543187	3.51E-98
CUL5	1.025669124	3.13E-62
CUL7	0.68456032	5.18E-13
CUL9	-0.244741964	0.000497519
CUTA	1.236675186	8.96E-88

CUTC	0.923892783	6.18E-74
CUX1	1.243218179	2.64E-59
CUX2	-0.853963782	4.59E-14
CUZD1	-1.116739702	5.00E-112
CWC15	0.689631468	1.39E-35
CWC22	1.217688842	3.68E-94
CWC25	1.156690174	5.88E-68
CWC27	1.111749773	2.24E-107
CWF19L1	1.03207955	1.41E-49
CWF19L2	1.014764994	2.64E-98
CWH43	0.156641174	2.96E-48
CX3CL1	1.203369493	6.19E-33
CX3CR1	3.701378897	1.13E-218
CXADR	2.148395496	6.45E-208
CXCL1	0.999515291	5.09E-24
CXCL10	4.307626406	8.91E-271
CXCL11	2.05955807	9.27E-118
CXCL12	1.377780192	3.42E-97
CXCL13	1.13566035	4.13E-63
CXCL14	1.368361718	1.69E-17
CXCL17	-0.060342617	0.022525221
CXCL2	1.691479875	1.99E-68
CXCL3	1.756595801	9.27E-213
CXCL5	0.957367181	7.23E-48
CXCL6	1.07539114	6.30E-228
CXCL8	3.912132657	2.61E-244
CXCL9	2.165369244	7.66295816699773e-321
CXCR1	0.570328184	4.15E-51
CXCR2	1.266371905	1.44E-284
CXCR4	3.855554028	1.14E-235
CXCR5	-0.032851868	1.58E-05
CXCR6	0.719588823	1.33E-285
CXorf38	1.497780408	5.96E-173
CXorf49	0.001080453	0.312246749
CXorf49B	0.003259227	0.000637134
CXorf51A	-0.000985499	0.424996027
CXorf51B	-0.000360161	0.631499975
CXorf58	0.193411126	1.38E-64
CXorf65	-0.096725689	9.04E-12
CXorf66	0.002029877	0.366291808
CXXC1	0.684084885	6.38E-15
CXXC4	0.92457241	8.36E-48
CXXC5	1.952162498	1.43E-67
CYB561	0.669434854	9.16E-12
CYB561A3	1.154219653	3.13E-107
CYB561D1	0.735627591	1.14E-18
CYB561D2	1.791699073	1.46E-218
CYB5A	0.425619811	1.01E-10

CYB5B	-0.042099708	0.475911614
CYB5D1	-1.790688784	2.12E-172
CYB5D2	1.521988761	9.83E-146
CYB5R1	1.924426812	1.14E-242
CYB5R2	-0.937990129	3.32E-23
CYB5R3	0.863546988	1.01E-71
CYB5R4	1.27263016	7.33E-80
CYB5RL	0.895480283	8.04E-104
CYBA	3.183786933	1.51E-245
CYBB	3.840342911	4.68E-305
CYBRD1	2.035917464	2.94E-138
CYC1	1.189270363	8.80E-72
CYCS	1.225068043	2.16E-72
CYFIP1	2.352949273	3.29E-300
CYFIP2	-1.034215439	1.68E-26
CYGB	-1.227348781	9.88E-36
CYHR1	-0.197755575	0.001682198
CYLC1	0.006599681	2.44E-05
CYLC2	-0.003704163	0.066257413
CYLD	-0.170327276	0.004221389
CYP11A1	-0.619770077	1.36E-31
CYP11B1	-0.205351562	7.78E-09
CYP11B2	-0.015603767	0.168596675
CYP17A1	-0.788995908	3.21E-71
CYP1A1	-0.229503785	1.53E-16
CYP1A2	0.005560397	0.184931831
CYP1B1	1.213370441	2.30E-56
CYP21A2	0.171562025	0.001257359
CYP24A1	0.151552687	3.02E-06
CYP26A1	-0.113287851	0.006520072
CYP26B1	-0.879091802	1.61E-09
CYP26C1	-0.005182514	0.654894229
CYP27A1	1.290090646	8.01E-64
CYP27C1	0.733520141	1.54E-87
CYP2A13	-0.027766397	6.81E-06
CYP2A6	-0.021188748	0.274575518
CYP2A7	-0.08517934	4.87E-16
CYP2B6	0.015061023	0.029567746
CYP2C18	-0.14741621	6.40E-16
CYP2C19	-0.032489156	3.30E-10
CYP2C8	-1.27236373	4.16E-82
CYP2C9	-0.018529109	0.004959945
CYP2D6	-0.566068272	1.76E-13
CYP2E1	-2.181667815	2.04E-120
CYP2F1	0.014928176	4.08E-08
CYP2J2	0.000292412	0.998913733
CYP2R1	1.019328377	4.27E-60
CYP2S1	1.623778069	7.76E-145

CYP2U1	1.726602653	3.54E-172
CYP2W1	0.102126777	6.58E-24
CYP39A1	0.542194287	6.18E-24
CYP3A4	0.007547575	0.701172684
CYP3A43	0.013955711	0.280448127
CYP3A7	-0.03460907	0.001594396
CYP46A1	-1.5161539	1.09E-40
CYP4A11	-0.271384397	1.40E-47
CYP4A22	-0.164720261	4.54E-51
CYP4B1	-0.122112655	2.48E-06
CYP4F11	0.725004148	3.22E-27
CYP4F12	0.355438988	8.99E-15
CYP4F2	0.071691398	9.96E-07
CYP4F22	0.074646043	2.97E-13
CYP4F3	0.657052087	1.22E-42
CYP4F8	-0.005510766	0.211224395
CYP4V2	-0.146669579	0.011928194
CYP4X1	-1.695971254	1.58E-75
CYP4Z1	-0.684496063	4.77E-62
CYP51A1	-0.46611472	2.73E-07
CYP7A1	0.049564994	3.21E-18
CYP7B1	0.675769173	1.52E-41
CYP8B1	0.019971154	0.000626631
CYS1	-1.145002255	1.33E-62
CYSLTR2	0.64423456	2.78E-48
CYSRT1	0.907968661	7.55E-82
CYSTM1	1.430319474	3.27E-161
CYTH1	-0.711627826	6.80E-26
CYTH2	0.805662968	1.97E-34
CYTH3	0.996867371	1.05E-56
CYTH4	2.307213908	1.35E-175
CYTIP	1.994903486	2.07E-236
CYYR1	1.382914249	1.96E-130
D2HGDH	0.211810769	0.000191807
DAAM1	0.196097955	0.004980243
DAAM2	-1.597259941	2.52E-58
DAB1	-0.606982962	3.43E-09
DAB2	2.014314621	1.88E-179
DAB2IP	-0.157300068	0.172664931
DACH1	0.083999962	0.371236916
DACH2	-0.961444081	6.17E-23
DACT1	0.704489861	1.10E-09
DACT2	-0.923035222	7.19E-17
DACT3	0.307275082	9.52E-05
DAGLA	0.725943009	5.95E-14
DAGLB	0.864215982	5.75E-33
DALRD3	0.880623874	1.54E-37
DAND5	0.618390958	5.67E-72

DAO	-0.920451653	3.14E-08
DAOA	-0.000174406	0.843321752
DAP3	1.847221029	3.45E-206
DAPK1	0.835537693	1.30E-17
DAPK2	0.025447976	0.5555461
DAPK3	1.447586335	2.62E-87
DAPL1	0.943807121	7.82E-33
DAPP1	1.09068486	9.36E-167
DARS2	1.452732303	6.95E-222
DAW1	-0.394694786	2.67E-06
DAXX	2.031451378	2.00E-253
DAZ1	0.021963692	3.86E-21
DAZ2	0.01857313	2.19E-37
DAZ3	0.017502667	7.50E-18
DAZ4	0.00919805	2.35E-14
DAZAP1	-0.493779807	3.15E-14
DAZAP2	1.409000866	2.71E-141
DAZL	0.034455566	3.21E-06
DBF4B	0.215292699	0.000104781
DBH	-0.100539488	0.05625247
DBI	2.53993543	3.22031987959324e-320
DBN1	1.616619056	1.47E-100
DBNDD1	0.462705088	6.29E-08
DBNL	1.655770987	8.03E-173
DBP	-1.757215089	7.03E-85
DBR1	2.050440636	3.59E-266
DBT	0.900216193	8.90E-86
DBX1	0.123190767	1.63E-75
DBX2	1.913374705	6.06E-208
DCAF1	0.986063316	5.67E-46
DCAF10	1.163643614	3.91E-86
DCAF11	0.043004985	0.501551964
DCAF12L1	-0.003417627	0.637285857
DCAF12L2	-0.781099972	5.89E-31
DCAF13	1.485923351	6.40E-233
DCAF15	1.441311292	3.49E-59
DCAF16	1.238771588	3.20E-75
DCAF17	0.753945271	1.51E-37
DCAF4	0.935946536	7.09E-68
DCAF4L1	0.174091456	3.08E-08
DCAF4L2	0.279128098	1.87E-161
DCAF5	1.107318637	1.13E-67
DCAF6	0.389638511	1.11E-05
DCAF7	1.969761882	1.93E-265
DCAF8	-1.014066883	2.33E-43
DCAF8L1	0.006119036	7.67E-09
DCAF8L2	0.006200239	1.39E-10
DCAKD	1.867605209	3.26E-248

DCANP1	0	1
DCBLD1	1.591473444	5.81E-117
DCBLD2	1.48914622	3.15E-92
DCC	0.371294804	7.99E-10
DCD	-0.273712608	1.21E-12
DCDC1	0.121393308	0.000216979
DCDC2	0.48638443	3.54E-22
DCDC2B	0.188742043	4.61E-09
DCDC2C	0.03860827	0.001380731
DCHS1	1.974118807	3.65E-202
DCHS2	0.057806507	0.231806229
DCK	1.673238867	5.02E-130
DCLK1	1.467456358	2.45E-46
DCLK2	2.292434853	1.57E-265
DCLK3	-0.381300263	2.68E-06
DCLRE1A	1.19060725	2.57E-106
DCLRE1B	2.368851186	3.22E-268
DCLRE1C	1.146948779	1.72E-149
DCN	2.325883082	2.44E-236
DCP1A	1.445705338	2.61E-80
DCP1B	1.540685792	1.42E-227
DCP2	1.047528632	2.90E-50
DCPS	0.471748984	9.69E-17
DCST1	0.257421054	4.61E-60
DCST2	-0.050454891	0.385383206
DCSTAMP	0.311327964	4.22E-13
DCT	0.473917926	5.41E-29
DCTD	1.881179534	4.64E-267
DCTN1	-1.135363648	1.14E-49
DCTN2	-0.109030274	0.173267228
DCTN3	1.554762078	1.83E-107
DCTN4	1.783547279	1.56E-177
DCTN5	0.727525715	1.47E-34
DCTN6	0.288537851	1.28E-08
DCTPP1	1.906152092	6.67E-154
DCUN1D1	1.146138013	8.74E-119
DCUN1D2	0.166748779	0.065688712
DCUN1D3	1.02376334	6.52E-97
DCUN1D4	0.753396906	1.30E-34
DCUN1D5	-0.38833513	8.25E-12
DCX	1.917899034	3.59E-160
DCXR	0.290740873	8.74E-09
DDA1	0.907903001	1.81E-69
DDAH1	0.565037153	1.65E-15
DDAH2	2.085414697	6.62E-250
DDB1	0.665574058	2.51E-23
DDB2	1.809175848	5.00E-132
DDC	-0.316078502	1.63E-05

DDHD1	-0.234575897	0.000597657
DDHD2	-0.653480932	1.16E-21
DDI1	-0.000646204	0.697414573
DDI2	0.129499371	0.047301289
DDIT3	0.71948804	6.48E-25
DDIT4	0.230242255	0.010709602
DDIT4L	1.526395482	3.75E-66
DDN	-3.139491988	4.54E-33
DDO	1.406661195	4.99E-82
DDR2	1.587049742	2.84E-121
DDRGK1	1.244275457	1.51E-66
DDTL	0.514926936	2.11E-28
DDX1	-0.109940238	0.154447043
DDX10	1.124791477	7.96E-73
DDX11	1.024591938	4.33E-29
DDX17	-0.133922546	0.108112385
DDX18	1.039182111	9.39E-62
DDX19A	-0.87205106	5.07E-44
DDX19B	1.395801638	1.14E-126
DDX20	1.848193418	5.67E-169
DDX21	1.824699026	4.81E-189
DDX23	1.654291072	4.70E-128
DDX24	-0.053313499	0.547428086
DDX25	-0.746102132	4.15E-23
DDX27	0.523856067	2.88E-11
DDX28	1.996871868	2.24E-191
DDX31	1.291590348	2.05E-60
DDX39A	2.384837409	1.33E-203
DDX39B	-1.225459255	7.87E-52
DDX3X	0.736384452	3.66E-26
DDX3Y	0.235843361	0.187488313
DDX4	-0.235816359	2.59E-51
DDX41	1.13799223	6.71E-65
DDX42	0.247803564	7.81E-05
DDX43	-0.14918831	1.17E-05
DDX46	0.676521542	2.63E-17
DDX47	0.661951865	5.81E-207
DDX49	1.447076489	7.05E-147
DDX5	0.755258287	1.89E-21
DDX50	1.362175312	6.71E-98
DDX51	0.078708216	0.358847793
DDX52	1.329439177	4.26E-123
DDX53	-0.003650595	0.434672805
DDX54	1.222891234	3.50E-82
DDX55	0.341772225	2.46E-06
DDX56	1.76015813	1.31E-87
DDX58	1.915576816	4.32E-270
DDX59	1.736063679	9.91E-242

DDX6	0.474347071	7.81E-13
DEAF1	0.005023086	0.950589278
DECR1	1.332397511	1.84E-160
DECR2	0.510833316	1.09E-16
DEDD	1.155981331	2.58E-94
DEDD2	0.827262366	1.33E-27
DEF6	1.90323366	1.81E-167
DEF8	0.317983235	7.10E-05
DEFA1	-0.002090291	0.268501811
DEFA1B	-0.002064601	0.264549559
DEFA3	-0.065312429	0.424812201
DEFA4	0.015189141	0.679434297
DEFA5	-0.242323743	8.49E-15
DEFA6	-0.049619536	0.012981027
DEFB1	-0.249330227	0.002008297
DEFB103A	-0.00045965	0.527331195
DEFB103B	0.00070689	0.464650587
DEFB104A	-0.000592932	0.562302537
DEFB104B	0.004460879	0.001872663
DEFB105A	0.002057709	0.033722782
DEFB105B	0.003848493	0.000340774
DEFB106A	-0.000371773	0.678420345
DEFB106B	-0.000544492	0.631500201
DEFB107A	0.001026576	0.241886341
DEFB107B	0.001424077	0.106400029
DEFB108B	0.017074212	0.000247328
DEFB110	0.012668046	5.65E-07
DEFB112	-0.005385678	0.164564033
DEFB113	0.005121929	1.59E-05
DEFB114	0.006676958	0.000257422
DEFB115	0.012425377	2.63E-05
DEFB116	0.014556479	1.41E-06
DEFB118	0.012754623	5.05E-22
DEFB119	0.034160474	2.02E-06
DEFB121	0.001411688	0.411489417
DEFB123	0.003099472	0.532862099
DEFB124	0.237546206	5.49E-34
DEFB125	0.006467211	0.004318022
DEFB126	0.023012974	2.30E-15
DEFB127	-0.001647157	0.266006556
DEFB128	0.001440378	0.436685172
DEFB129	0.003691062	0.024046213
DEFB132	0.000539609	0.671439118
DEFB134	-0.162794139	4.79E-14
DEFB135	0.044499129	2.51E-05
DEFB136	-0.023908692	0.011018716
DEFB4A	0.035766192	4.93E-08
DEFB4B	0.004422829	0.036391423

DEGS1	2.470492495	1.73E-304
DEGS2	-1.352784284	2.00E-35
DEK	1.374314204	6.30E-64
DENND1A	0.881725391	2.52E-51
DENND1B	0.734580852	1.13E-57
DENND1C	0.64872261	1.86E-29
DENND2C	0.155461326	6.90E-08
DENND3	0.556454826	7.32E-11
DENND4A	0.456773486	4.65E-11
DENND4B	-0.017981546	0.847353922
DENND4C	1.200590223	2.79E-62
DENND5A	0.616519826	8.98E-34
DENND5B	0.40634178	2.86E-07
DENND6A	1.359606196	1.27E-90
DENND6B	-0.489581018	8.52E-08
DENR	1.702214524	7.89E-137
DEPDC5	-0.714166261	7.64E-28
DEPDC7	0.629805554	1.08E-26
DEPTOR	0.382670741	4.35E-09
DERL1	0.944183819	3.51E-39
DERL2	1.665067224	5.18E-201
DERL3	-0.896849932	2.25E-45
DES	0.52548208	1.97E-08
DESI1	0.702272447	1.10E-44
DESI2	2.352490766	3.10E-191
DET1	-1.340671751	3.68E-179
DEUP1	-0.100389546	2.91E-06
DEXI	0.484970544	2.70E-16
DFFA	1.641034058	4.90E-252
DFFB	0.279587877	0.000892107
DGAT1	0.560658342	4.05E-13
DGAT2	-0.768024063	1.83E-13
DGAT2L6	0.062661231	6.53E-62
DGCR2	1.015957081	2.99E-55
DGCR6L	1.17291034	1.57E-138
DGCR8	0.206522729	0.048678712
DGKA	-0.198715752	0.020412317
DGKB	-0.007245576	0.955285322
DGKD	0.219851335	0.072542836
DGKE	-1.692812215	1.13E-90
DGKG	-0.135965008	0.232923353
DGKH	-0.749182021	1.54E-15
DGKI	-0.274478929	0.000342225
DGKK	-0.275543569	0.000150268
DGKQ	-0.286267865	0.002037802
DGKZ	-1.095714123	3.14E-26
DGUOK	1.50561565	6.75E-143
DHCR24	-0.337008048	0.011609682

DHCR7	1.557150143	4.55E-130
DHDDS	1.034503485	2.10E-19
DHDH	0.051342862	0.458527388
DHFR	2.012153049	1.92E-240
DHFR2	1.364251114	2.24E-155
DHH	0.30374464	6.64E-24
DHODH	1.47868859	1.26E-273
DHPS	-3.437052156	4.84E-293
DHRS1	0.293013649	1.24E-06
DHRS11	-0.583668437	8.76E-16
DHRS12	-0.398397385	1.39E-19
DHRS13	0.54591201	3.76E-08
DHRS2	-8.57E-05	1
DHRS3	2.136058874	8.04E-167
DHRS4	1.133959125	5.69E-139
DHRS4L2	1.31114148	9.89E-183
DHRS7	1.031612315	9.61E-68
DHRS7B	1.253770485	1.67E-142
DHRS7C	-0.127428308	0.000178931
DHRS9	1.046311677	2.87E-80
DHTKD1	1.188013913	5.21E-120
DHX15	1.821793362	1.56E-129
DHX16	1.445977396	1.56E-80
DHX29	1.069748094	1.09E-49
DHX30	1.127239659	8.55E-48
DHX32	1.371249528	2.56E-160
DHX33	1.439069531	1.04E-97
DHX34	0.77581679	2.07E-30
DHX35	1.126047352	3.30E-84
DHX36	1.038691522	9.40E-54
DHX37	1.516123784	1.32E-181
DHX38	0.736416255	2.12E-22
DHX40	1.932170703	2.35E-213
DHX57	1.165123383	1.89E-93
DHX58	1.308843206	1.56E-143
DHX8	1.418519006	6.00E-112
DHX9	1.882672612	8.52E-138
DIAPH1	1.359631506	5.25E-61
DIAPH2	1.318950527	1.42E-107
DICER1	0.296801315	1.36E-05
DIDO1	1.055808263	2.66E-44
DIMT1	-0.456192249	1.97E-21
DIO1	0.299875202	3.79E-124
DIO2	0.224384807	0.040551922
DIO3	-0.180601565	0.005787
DIP2A	-0.163774803	0.034402985
DIP2B	0.533251075	4.67E-10
DIP2C	0.188259542	0.004698894

DIRAS1	0.506749686	7.46E-05
DIRAS2	-0.958557764	1.53E-08
DIRAS3	2.39503542	8.30E-48
DIS3	1.099177378	8.90E-63
DIS3L	0.883427959	4.07E-65
DIS3L2	0.412925691	7.57E-15
DISC1	1.425153694	2.18E-271
DISP1	1.124283812	2.20E-158
DISP2	-0.961350343	5.73E-13
DISP3	0.796733054	5.95E-35
DIXDC1	-0.079128398	0.394422286
DKC1	1.792298917	2.38E-173
DKK1	1.115142828	3.89E-67
DKK2	0.308623219	3.76E-14
DKK3	-1.270548292	3.02E-46
DKK4	-0.323441774	3.59E-06
DLAT	1.158782879	2.39E-69
DLC1	0.813965797	2.10E-38
DLD	1.631935894	6.56E-134
DLEC1	0.591624936	2.06E-15
DLEU7	-0.270085975	2.30E-07
DLG1	-0.690211791	2.35E-26
DLG2	-2.010767743	7.06E-128
DLG3	-0.374477014	6.96E-05
DLG4	-2.053018813	9.44E-70
DLG5	1.02831347	2.08E-51
DLGAP1	-0.291051837	0.029364114
DLGAP2	-1.162817134	4.11E-39
DLGAP3	-2.555086523	4.69E-97
DLGAP4	0.774306013	3.06E-24
DLK1	0.328997514	0.004021707
DLK2	-0.508231175	5.24E-09
DLL1	1.179927018	2.77E-67
DLL3	2.899539786	7.38E-282
DLL4	0.466056638	4.05E-12
DLST	1.08442448	4.97E-59
DLX1	0.752039499	1.95E-09
DLX2	0.686292589	1.44E-26
DLX3	0.163868474	8.03E-16
DLX4	0.172043552	1.86E-13
DLX5	0.973567949	3.15E-19
DLX6	-0.226870368	0.042562044
DMAP1	0.850877825	1.95E-29
DMBT1	-0.142005797	0.000711588
DMBX1	0.658374622	1.18E-139
DMC1	-0.031275149	0.15561891
DMD	0.64054369	4.83E-47
DMGDH	0.173216781	1.64E-05

DMKN	-1.35897624	3.26E-63
DMP1	0.295013744	1.12E-278
DMPK	-0.257347626	5.48E-05
DMRT1	0.15466802	4.95E-61
DMRT2	0.290861513	8.17E-09
DMRT3	0.058639168	0.110143052
DMRTA1	0.29524881	1.27E-45
DMRTB1	0.015254503	4.89E-07
DMRTC1	0.044023498	2.64E-22
DMRTC1B	-0.034631296	0.032410838
DMRTC2	-0.001578231	0.415259979
DMTF1	-0.052937516	0.52152608
DMTN	-2.089775384	1.09E-74
DMWD	0.304174512	9.97E-07
DMXL1	0.443400238	4.48E-12
DMXL2	-0.147712038	0.08702119
DNA2	0.461644413	1.35E-12
DNAAF1	-0.735326519	1.68E-16
DNAAF2	1.837816225	2.35E-211
DNAAF3	0.646635995	1.45E-26
DNAAF5	2.029825707	2.66E-219
DNAH1	-0.525983593	7.33E-14
DNAH10	-0.210126921	3.63E-06
DNAH11	0.516411535	2.47E-48
DNAH12	0.063727053	0.093551363
DNAH14	-0.009455227	0.602118838
DNAH17	-0.276745327	0.000238644
DNAH2	-0.562048951	1.82E-22
DNAH3	-0.089728312	0.016646554
DNAH5	0.362703754	3.46E-23
DNAH6	-0.243863167	6.98E-06
DNAH7	-0.128747577	0.035730342
DNAH8	0.072837745	2.00E-09
DNAH9	1.460215505	2.01E-110
DNAI1	-0.270137765	0.000113104
DNAI2	0.118582591	0.081568312
DNAJA1	1.441362064	2.21E-81
DNAJA2	1.155206218	2.10E-72
DNAJA3	0.702772287	1.97E-24
DNAJA4	-2.354238299	2.21E-131
DNAJB1	0.391767303	0.00042893
DNAJB11	-0.164304156	0.006093044
DNAJB12	0.859155308	4.43E-44
DNAJB13	0.153370274	1.90E-05
DNAJB14	-0.128212445	0.065476204
DNAJB2	-0.618534471	1.73E-20
DNAJB4	0.728739791	4.37E-28
DNAJB5	1.189718242	1.16E-53

DNAJB6	0.984445107	1.86E-57
DNAJB7	-0.276877509	2.35E-12
DNAJB8	-0.005330918	0.083511649
DNAJB9	2.21417677	1.82E-221
DNAJC1	1.870947521	5.49E-280
DNAJC10	1.405464295	1.00E-140
DNAJC11	0.821778002	1.41E-28
DNAJC12	-0.955416344	2.93E-21
DNAJC13	1.008024756	9.50E-65
DNAJC14	0.006975819	0.917660768
DNAJC15	0.585697745	3.17E-30
DNAJC16	1.045096706	4.23E-78
DNAJC17	0.267395316	6.37E-05
DNAJC18	0.975174329	6.16E-57
DNAJC19	0.437571397	1.32E-15
DNAJC2	1.00411537	3.87E-76
DNAJC21	0.427968756	1.72E-20
DNAJC24	1.137943385	2.19E-101
DNAJC25	0.958102542	1.45E-43
DNAJC27	-0.32270953	7.79E-07
DNAJC28	0.508505381	8.89E-27
DNAJC3	1.947480043	4.63E-254
DNAJC30	1.131274234	5.00E-74
DNAJC4	-0.364198754	4.12E-16
DNAJC5	0.278754586	0.001144206
DNAJC5B	0.481234876	1.25E-250
DNAJC5G	-0.328293874	1.57E-07
DNAJC6	-0.973685217	1.85E-22
DNAJC7	-1.520875749	1.26E-110
DNAJC8	1.752103605	7.07E-198
DNAJC9	0.033844437	0.703806095
DNAL1	0.646121264	1.62E-40
DNAL4	0.748034289	1.29E-32
DNALI1	2.549909819	1.71E-294
DNASE1	-0.054677214	0.490280796
DNASE1L1	0.103967657	0.060670249
DNASE1L2	-1.247214252	5.47E-36
DNASE1L3	0.219435718	7.33E-33
DNASE2B	0.497971335	1.78E-48
DNER	1.714307485	3.11E-105
DNHD1	0.098348903	0.069628786
DNM1	-1.805221215	8.58E-31
DNM1L	-0.008868126	0.919124705
DNM2	0.589663134	4.32E-19
DNM3	-1.40521938	1.59E-47
DNMBP	1.285429652	1.80E-102
DNMT1	0.873778468	1.38E-19
DNMT3A	0.781551993	6.36E-26

DNMT3B	0.826556739	9.77E-37
DNMT3L	0.024657803	2.58E-23
DNPEP	1.519386114	3.82E-220
DNPH1	1.273677799	1.74E-100
DNTT	-0.015005089	0.007123384
DNTTIP1	2.233987635	3.01E-295
DNTTIP2	1.580185735	1.82E-171
DOC2A	-2.152369129	3.73E-46
DOC2B	-2.757266694	9.51E-105
DOCK1	0.063967494	0.314563047
DOCK10	0.82665567	8.09E-21
DOCK11	1.917202702	2.45E-190
DOCK2	1.849385468	2.08E-174
DOCK3	-1.169533888	9.50E-33
DOCK4	0.523586571	1.27E-14
DOCK5	-0.70888505	3.19E-19
DOCK6	0.787811676	1.15E-45
DOCK7	1.352520548	2.39E-155
DOCK8	1.835049706	2.86E-175
DOCK9	-0.833651982	3.03E-32
DOHH	0.198936732	0.002752827
DOK3	1.580393532	6.08E-139
DOK4	0.216096709	0.033825022
DOK5	2.893601349	3.78E-211
DOK6	-1.876624063	1.82E-70
DOK7	-0.44066343	0.000132862
DOLPP1	1.787989642	1.47E-188
DONSON	1.039281693	1.22E-44
DOT1L	0.911155539	3.28E-22
DPAGT1	1.140538259	1.83E-72
DPCD	1.089393666	5.45E-48
DPEP2	1.194704185	1.74E-181
DPEP3	0.383177705	7.54E-72
DPF1	0.361294134	3.44E-05
DPF2	0.278056139	7.70E-06
DPF3	1.286943105	3.27E-50
DPH1	-0.629541689	9.54E-10
DPH2	1.542189436	7.50E-146
DPH3	1.735438931	3.87E-228
DPH7	0.92824969	1.57E-37
DPM1	1.819476051	7.52E-169
DPM2	1.348830302	1.18E-160
DPP10	-0.879823384	1.94E-23
DPP3	2.019641872	2.47E-213
DPP4	1.1390511	7.53E-141
DPP6	0.360845175	0.000393823
DPP7	0.747922674	3.07E-38
DPP8	0.770256829	3.26E-34

DPP9	1.174762908	1.29E-88
DPPA2	0.011810399	5.60E-09
DPPA3	0.012097087	4.11E-06
DPPA4	0.20399762	2.36E-31
DPPA5	-0.000503266	0.937392508
DPRX	-0.063261789	3.74E-05
DPT	-0.302636566	8.59E-16
DPY19L1	2.960118752	6.08E-230
DPY19L2	-0.569946628	1.95E-12
DPY19L3	1.638211591	1.14E-174
DPY19L4	1.757187823	2.29E-247
DPY30	0.87089938	1.05E-58
DPYS	-0.206178418	5.24E-08
DPYSL2	1.114693667	5.19E-59
DPYSL3	3.256609102	4.04536430608219e-315
DPYSL4	0.740997308	3.81E-25
DPYSL5	0.71141028	5.79E-12
DQX1	0.189583071	2.03E-11
DR1	1.716067769	2.71E-161
DRAM2	1.821271276	5.68E-131
DRAP1	0.8386711	1.62E-44
DRC1	0.777751292	2.90E-22
DRC3	0.773519109	1.97E-26
DRC7	0.536273924	3.37E-20
DRD1	-1.499574072	1.76E-23
DRD2	-0.719433972	1.59E-05
DRD3	-0.169755547	0.004124762
DRD4	-0.141882417	0.025163035
DRD5	-0.722560222	1.00E-23
DRG1	1.600629139	4.62E-132
DRG2	-0.106815468	0.106394013
DRGX	-0.305981545	1.01E-07
DRICH1	-0.249065685	9.05E-05
DROSHA	0.82348586	1.59E-28
DRP2	-0.041506858	0.721955052
DSC1	-0.113008242	2.94E-13
DSC2	0.706283378	1.38E-111
DSC3	-0.032187658	0.042720964
DSCAM	0.876683959	2.81E-47
DSCAML1	-0.371687826	1.20E-08
DSCC1	2.242232988	1.38E-237
DSE	1.113661565	4.57E-157
DSEL	1.898059699	1.34E-234
DSG1	-0.18900941	4.57E-26
DSG2	1.271067597	4.61E-248
DSG3	-0.059822037	2.17E-07
DSG4	-0.001562808	0.276999486
DSN1	2.447136408	1.11E-304

DSP	0.067928158	0.178493799
DSPP	0.039716677	7.04E-50
DST	-0.711525577	2.06E-33
DSTN	1.485347762	5.56E-87
DSTYK	0.11133554	0.13228472
DTD1	-0.215853208	0.000328224
DTD2	-0.070072123	0.152100248
DTHD1	0.600594366	2.94E-26
DTNA	0.622202881	2.49E-26
DTNB	-1.208722594	6.17E-61
DTNBP1	0.576834985	2.42E-17
DTWD1	0.373469968	3.70E-12
DTWD2	0.913359224	8.27E-58
DTX1	0.606262052	7.18E-16
DTX2	1.411477202	4.30E-240
DTX3	0.925930438	6.71E-28
DTX4	-0.181689753	0.037327247
DUOX1	-0.112405572	0.135037387
DUOX2	-0.085146255	3.02E-05
DUOXA1	0.128007959	6.50E-06
DUOXA2	-0.006327375	0.451359468
DUS1L	-0.078222384	0.274240013
DUS2	1.044908961	6.18E-99
DUS3L	1.355510628	1.86E-90
DUS4L	-0.005508363	0.910219242
DUSP1	1.539660841	1.56E-67
DUSP10	2.046677203	8.47E-181
DUSP11	1.919531454	5.21E-222
DUSP12	1.92119306	2.61E-132
DUSP13	0.097783377	2.36E-13
DUSP14	1.90711192	2.22E-173
DUSP15	1.341959846	6.09E-144
DUSP16	1.085250966	2.75E-69
DUSP18	0.806739401	1.36E-91
DUSP19	0.83314693	9.59E-65
DUSP2	-0.381873126	6.21E-05
DUSP21	0.005692888	0.001445835
DUSP22	0.854039793	8.69E-55
DUSP23	1.305844369	3.31E-108
DUSP26	-1.435426377	1.06E-52
DUSP28	0.209231233	2.94E-05
DUSP3	0.77921041	6.24E-24
DUSP4	1.94189339	7.81E-87
DUSP5	0.972972488	2.55E-15
DUSP6	2.48239026	2.06E-206
DUSP7	0.303237487	2.57E-07
DUSP8	-1.674047681	2.64E-69
DUSP9	-0.347854007	5.01E-06

DUT	0.965691522	4.51E-57
DUX4	0.004052911	9.59E-06
DUXA	0.00559775	0.250350696
DVL1	-0.218152511	0.000260703
DVL2	1.031982304	6.70E-40
DVL3	1.568121728	4.14E-133
DXO	0.39043255	2.67E-06
DYDC1	-0.015897316	0.14538318
DYDC2	-0.419828241	1.15E-05
DYM	0.696687032	1.09E-29
DYNAP	0.001646603	0.506469159
DYNC1H1	-1.088400706	1.11E-40
DYNC1I1	-1.728579864	4.37E-47
DYNC1I2	0.776683002	5.82E-34
DYNC1LI1	0.263034381	0.000767386
DYNC1LI2	-0.716802771	8.20E-25
DYNC2H1	0.546585533	3.83E-29
DYNC2LI1	0.949946462	8.85E-62
DYNLL1	1.363936663	3.18E-89
DYNLL2	0.032542352	0.639598188
DYNLRB1	-2.323965435	1.39E-248
DYNLRB2	0.106575553	0.1049223
DYNLT3	0.595481999	3.49E-14
DYRK1A	-0.437644842	3.54E-11
DYRK1B	0.708626655	1.24E-25
DYRK2	1.365679727	5.94E-181
DYRK4	1.065839255	4.71E-126
DYSF	0.402783151	5.48E-09
DYTN	0.014435757	0.003589225
DZANK1	0.647162411	4.00E-38
DZIP1	-0.043541375	0.575969886
DZIP1L	1.082337859	2.67E-61
DZIP3	0.086811348	0.238121092
E2F1	2.436078904	5.63E-263
E2F3	2.013791764	6.24E-155
E2F4	0.921325436	6.77E-37
E2F5	1.556477638	2.21E-186
E2F6	1.742443244	6.66E-247
E4F1	-0.097511361	0.248695336
EAF1	0.88626302	1.29E-40
EAF2	1.055298358	5.69E-156
EAPP	1.514523462	2.76E-111
EARS2	0.957730953	9.82E-53
EBAG9	1.196438065	7.49E-100
EBF1	0.272939329	0.005704305
EBF2	0.551318634	1.71E-41
EBF3	0.161199348	0.019363799
EBF4	1.689076122	3.20E-182

EBI3	2.788827118	1.75E-238
EBLN1	0.043822156	0.001328258
EBLN2	0.331557929	5.10E-19
EBNA1BP2	1.487303672	1.28E-104
EBP	0.93872775	1.28E-31
EBPL	1.972098911	7.09E-267
ECD	0.934906798	1.64E-51
ECE1	2.413190788	1.44E-275
ECE2	-3.553360486	2.64E-226
ECEL1	-0.433111136	0.002237937
ECH1	0.199464956	5.33E-06
ECHDC1	1.28620735	1.28E-112
ECHDC2	-0.94907633	5.34E-25
ECHDC3	0.638006883	5.49E-25
ECHS1	1.149251735	3.27E-125
ECI1	1.355110722	4.95E-147
ECI2	0.075759777	0.122660148
ECM1	1.657965073	4.97E-91
ECSCR	-0.197281022	0.014600399
ECSIT	1.250099101	1.78E-71
ECT2L	0.057742216	0.044023763
EDA	-0.057914444	0.025897777
EDAR	0.151041853	5.28E-54
EDARADD	0.345527854	7.14E-143
EDC3	1.196982228	1.54E-82
EDC4	-0.399755359	2.23E-05
EDDM3A	0.153344241	1.38E-111
EDDM3B	-0.001356329	0.541446135
EDEM1	1.055884313	5.37E-64
EDEM2	2.80556817	1.07E-225
EDEM3	1.27413326	1.18E-83
EDF1	1.412436	1.76E-125
EDIL3	-0.830807216	7.39E-15
EDN1	0.908251493	9.36E-26
EDN2	0.370163528	2.42E-128
EDN3	0.114779209	0.113691547
EDNRB	2.286689073	1.78E-114
EDRF1	-0.48129358	2.63E-10
EEA1	1.382784777	2.42E-118
EED	0.681325025	8.83E-29
EEF1A1	1.793143623	9.67E-197
EEF1A2	-2.228087711	1.02E-56
EEF1AKMT1	1.682942257	6.36E-253
EEF1B2	1.212131909	1.17E-110
EEF1D	0.040295023	0.50704865
EEF1E1	-0.300606971	4.36E-06
EEF2	1.509529998	5.21E-101
EEF2K	0.965236177	1.73E-18

EEF2KMT	1.278598526	6.37E-152
EEFSEC	1.752817901	2.16E-165
EFCAB1	-0.184636987	0.031649649
EFCAB10	0.446119597	5.27E-27
EFCAB11	0.84846075	3.48E-147
EFCAB12	-0.671236008	2.30E-16
EFCAB13	0.112068359	0.000749887
EFCAB14	-0.178563407	0.000139971
EFCAB2	0.690883039	3.41E-62
EFCAB3	0.027232556	2.17E-11
EFCAB5	-0.124334685	3.72E-06
EFCAB6	-0.028518304	0.487308262
EFCAB7	0.827867542	6.23E-33
EFCAB8	0.065824069	2.27E-15
EFCAB9	0.03055838	0.243074669
EFCC1	0.160182457	0.012134668
EFEMP1	2.910382195	2.40E-142
EFEMP2	1.48546995	2.31E-209
EFHB	0.581111257	3.46E-25
EFHC1	1.020659911	6.59E-84
EFHC2	1.326819088	4.51E-103
EFHD1	0.300759882	0.003398088
EFHD2	1.517982988	1.53E-47
EFL1	1.868745521	1.44E-304
EFNA1	1.955143757	2.19E-137
EFNA2	1.675043721	3.88E-224
EFNA3	-0.747442557	2.71E-14
EFNA5	-0.23441238	0.004082952
EFNB2	0.692927906	3.91E-25
EFNB3	1.463927898	1.77E-24
EFR3A	0.577522179	7.73E-11
EFR3B	-0.420806988	5.62E-09
EFS	1.029780032	1.21E-42
EFTUD2	1.775920717	9.03E-157
EGF	1.079151658	1.59E-207
EGFL6	0.610023174	1.43E-51
EGFL7	-0.021298544	0.783406115
EGFLAM	1.516546076	2.58E-210
EGLN1	-1.919472646	2.16E-262
EGLN3	1.448000882	2.89E-118
EGR1	3.598515224	1.66E-143
EGR2	3.137311313	4.21E-243
EGR3	0.693367275	2.22E-06
EGR4	-2.074004806	7.34E-54
EHBP1	0.271868156	0.000145601
EHBP1L1	0.739215139	2.42E-33
EHD1	0.971662654	1.87E-23
EHD3	-0.261414468	0.004715693

EHD4	1.757649088	1.07E-96
EHF	0.025746481	0.530552535
EHHADH	1.054274935	2.67E-117
EHMT1	0.791638413	1.90E-48
EHMT2	-0.552156748	9.04E-17
EI24	1.728628497	1.64E-214
EID1	1.596532074	1.76E-115
EID2	1.381740566	1.20E-70
EID2B	-0.338930808	9.03E-05
EID3	0.576631513	1.46E-22
EIF1	1.119515364	1.70E-88
EIF1AD	1.698131346	2.91E-170
EIF1AX	1.603378562	3.30E-101
EIF1AY	0.815097524	1.08E-11
EIF1B	0.003829342	0.963696639
EIF2A	0.47861543	8.42E-09
EIF2AK1	1.898979765	1.74E-219
EIF2AK2	1.662956743	4.31E-184
EIF2AK3	1.578366824	8.29E-156
EIF2AK4	1.029096372	1.18E-76
EIF2B1	1.474863834	2.53E-96
EIF2B2	0.968204242	2.77E-73
EIF2B3	1.692685309	8.17E-128
EIF2B4	0.254068435	3.35E-05
EIF2B5	0.233676416	0.003695055
EIF2D	0.587613611	1.75E-38
EIF2S1	2.100299806	7.09E-259
EIF2S2	1.905555821	1.09E-218
EIF2S3	2.042005394	3.03E-186
EIF3A	0.830305486	3.55E-40
EIF3B	0.961506966	4.07E-57
EIF3C	1.149338718	1.48E-96
EIF3D	1.848342877	2.09E-167
EIF3E	-1.298421916	2.27E-74
EIF3F	0.866901367	1.49E-66
EIF3G	0.669184396	3.19E-36
EIF3H	1.328958197	6.11E-114
EIF3I	0.010116331	0.86422668
EIF3J	1.341860568	1.59E-133
EIF3K	1.308953083	1.39E-116
EIF3L	0.31975997	0.000229948
EIF3M	1.622184407	4.12E-159
EIF4A2	-1.111254629	8.30E-40
EIF4A3	1.168098763	1.19E-70
EIF4B	0.827900394	4.37E-31
EIF4E	0.93214581	9.03E-55
EIF4E1B	-1.327063531	3.70E-49
EIF4E2	1.06806212	1.44E-58

EIF4E3	-0.538268228	8.33E-19
EIF4EBP2	1.179307584	2.58E-101
EIF4ENIF1	0.245588625	7.75E-05
EIF4G1	1.348015485	3.31E-115
EIF4G2	1.409851918	8.73E-107
EIF4G3	0.806370218	4.61E-33
EIF4H	0.540396039	6.23E-16
EIF5	1.119342927	1.51E-72
EIF5A	1.833546707	1.45E-198
EIF5A2	0.8237991	1.13E-35
EIF5B	0.089495095	0.052998808
EIF6	1.494444692	7.98E-139
ELAC2	0.771785979	4.59E-36
ELANE	0.74006867	9.72E-69
ELAVL1	1.672862487	3.27E-141
ELAVL2	-1.072650705	1.83E-18
ELAVL3	-1.330069485	3.47E-42
ELAVL4	-0.282880102	0.030104176
ELF1	2.440944549	1.33E-291
ELF2	0.6108762	4.40E-21
ELF3	0.296843032	1.25E-44
ELF5	0.275337831	1.60E-166
ELFN1	1.421578053	1.55E-79
ELFN2	0.875379358	3.43E-31
ELK1	0.72163129	5.20E-13
ELK3	2.704922343	7.69E-267
ELK4	1.081454723	1.88E-71
ELL	0.815548686	3.06E-26
ELL2	1.169704991	5.49E-54
ELL3	-1.27283162	1.25E-44
ELMO1	-0.529542406	3.10E-10
ELMO2	0.72185645	1.58E-20
ELMO3	0.517631268	2.26E-09
ELMOD1	-0.338108082	0.003484229
ELMOD2	1.580385727	7.71E-165
ELMOD3	0.573017836	6.66E-24
ELN	3.006508889	1.01E-122
ELOA	1.685929164	1.10E-151
ELOA2	-0.004609091	0.490868246
ELOA3	4.66E-05	0.97354245
ELOA3B	-0.003770305	0.046726649
ELOA3D	-0.000641632	0.720820757
ELOB	1.545387933	2.53E-226
ELOC	1.531262832	9.31E-150
ELOF1	1.703368318	1.19E-181
ELOVL1	1.929935977	2.36E-82
ELOVL2	2.348496351	1.91E-158
ELOVL3	0.010090914	0.791070528

ELOVL4	-0.193871426	0.069213365
ELOVL5	2.172801132	3.86E-181
ELOVL6	1.003179169	1.08E-44
ELOVL7	-1.054533364	4.18E-45
ELP2	0.959277329	1.21E-48
ELP3	1.38362146	2.69E-112
ELP4	0.474700148	7.44E-24
ELP5	1.67494954	9.49E-179
ELP6	1.814994841	1.84E-220
ELSPBP1	0.424583513	3.57E-109
EMB	1.526709609	4.10E-116
EMC1	2.014251862	3.48E-183
EMC10	1.60688136	2.15E-208
EMC2	-0.825627243	1.48E-54
EMC3	1.442250047	4.66E-122
EMC4	-0.197581691	0.001293434
EMC7	2.102831365	2.47E-267
EMC8	1.436443455	2.12E-204
EMC9	0.127626314	0.118940964
EMCN	1.304745548	1.89E-146
EMD	1.214076037	5.33E-74
EME2	-1.258171996	1.41E-29
EMG1	0.877857643	9.75E-74
EMID1	0.56507523	6.54E-14
EMILIN2	2.461035556	4.16E-160
EMILIN3	2.66604891	4.94E-289
EML1	0.988679481	6.07E-36
EML2	-0.8117873	1.29E-33
EML3	1.098852609	3.61E-69
EML4	1.362732344	7.87E-108
EML5	-0.611296948	8.55E-19
EML6	-0.63339619	4.62E-13
EMP2	0.5021201	2.89E-22
EMSY	0.497476147	8.66E-12
EMX1	-0.503535406	1.25E-05
EMX2	-0.217523687	0.100861296
EN2	1.007270403	3.13E-08
ENAM	-0.002253581	0.379327394
ENC1	1.068643169	3.38E-08
ENDOD1	1.27973409	1.62E-78
ENDOG	-0.108020416	0.116588733
ENDOU	-0.335482243	9.28E-08
ENDOV	0.532794458	1.33E-21
ENG	2.389106863	4.22E-245
ENGASE	0.369624671	3.84E-09
ENHO	-1.817061136	1.16E-153
ENKD1	0.66793155	3.20E-20
ENKUR	1.837470575	1.67E-161

ENO1	1.139748482	3.80E-83
ENO2	-0.460529837	0.000516123
ENO3	0.56936134	4.34E-15
ENO4	0.093467364	0.026335533
ENOPH1	1.538027286	1.50E-129
ENOSF1	-0.422377876	4.13E-09
ENOX1	0.410017936	2.94E-09
ENOX2	1.371145445	1.87E-152
ENPP1	0.855572623	1.02E-142
ENPP2	-1.098903814	1.98E-16
ENPP3	-0.034658331	0.491129843
ENPP4	0.076456763	0.320180068
ENPP5	0.128393975	0.117555247
ENPP6	1.196204552	2.61E-29
ENPP7	0.073354676	9.30E-60
ENSA	1.152927774	3.48E-80
ENTHD1	0.189071071	3.98E-09
ENTPD1	0.390332768	6.33E-16
ENTPD2	0.886304675	8.40E-26
ENTPD3	-1.670192581	7.03E-47
ENTPD4	0.25462282	0.001455847
ENTPD5	0.579656298	9.98E-28
ENTPD6	-0.166130323	0.093551363
ENTPD7	1.276112363	1.41E-208
ENTPD8	0.206658362	2.29E-35
ENY2	0.662452183	1.70E-29
EOGT	0.867995544	3.22E-81
EOMES	-0.484699329	0.000952279
EP300	0.630125863	3.01E-14
EP400	0.272159522	0.000317406
EPAS1	1.235793134	2.16E-95
EPB41	-0.554269355	0.000325721
EPB41L1	-0.894052508	5.61E-30
EPB41L2	0.957536106	1.66E-31
EPB41L3	-1.10159613	1.22E-24
EPB41L4A	0.295930379	9.89E-18
EPB41L4B	-1.289902176	2.54E-35
EPB41L5	0.797319105	2.17E-25
EPB42	-0.064099383	1.51E-12
EPC1	-0.451463041	2.27E-09
EPC2	1.447771967	6.99E-86
EPCAM	-1.261576217	1.01E-41
EPDR1	1.23530205	6.64E-26
EPG5	0.617684754	3.96E-21
EPGN	0.073695802	1.51E-52
EPHA1	0.40201709	1.25E-09
EPHA10	-1.316816402	1.22E-55
EPHA2	2.032601071	1.67E-244

EPHA3	1.821991839	7.24E-275
EPHA4	0.806916278	3.17E-13
EPHA5	0.427427195	6.48E-08
EPHA6	-0.571513211	1.07E-44
EPHA7	-0.89337078	3.99E-21
EPHA8	-0.692232271	5.48E-53
EPHB1	0.920472497	1.15E-18
EPHB2	2.18923625	1.49E-287
EPHB3	2.116716653	1.40E-100
EPHB6	-2.039306249	7.11E-44
EPHX1	1.068041461	3.34E-68
EPHX2	0.35991138	9.54E-12
EPHX3	0.813947351	2.64E-225
EPHX4	0.242410809	0.015595409
EPM2A	-0.934762317	6.14E-81
EPM2AIP1	0.722073965	2.57E-18
EPN1	0.795582577	3.05E-32
EPN2	-0.203334117	4.50E-05
EPN3	-0.447194129	1.22E-12
EPO	0.4941109	1.51E-84
EPOP	0.838727756	9.81E-17
EPOR	0.796072768	9.83E-59
EPPIN	-0.045765921	2.28E-14
EPPK1	-0.024745416	0.262632549
EPS15	0.267211386	9.69E-06
EPS15L1	0.795077507	1.27E-27
EPS8	1.152017055	8.91E-127
EPS8L1	0.579171806	1.13E-172
EPS8L2	-0.174877988	0.159991841
EPS8L3	0.001061939	0.82719003
EPSTI1	1.968076885	1.70E-242
EPX	0.078898673	0.10212434
EPYC	0.189777266	1.50E-36
EQTN	0.036835494	0.040037455
ERAL1	1.337038132	1.05E-87
ERAP1	2.221470227	1.13E-293
ERAP2	2.450610104	8.83E-279
ERAS	-0.248894236	2.37E-05
ERBB2	1.458914207	4.59E-101
ERBB3	-0.421724121	4.82E-05
ERBB4	-0.644353364	1.92E-25
ERBIN	1.105197242	2.50E-46
ERC1	0.549451725	4.21E-12
ERC2	-0.527445601	1.37E-09
ERCC1	1.185602061	3.50E-96
ERCC2	0.947201505	3.78E-64
ERCC3	-0.065087649	0.446799143
ERCC4	1.119991229	1.78E-131

ERCC5	-0.227117758	1.14E-07
ERCC6	-0.050686243	0.363357249
ERCC6L2	1.010732507	3.55E-90
ERCC8	0.339038135	2.10E-19
EREG	0.414318971	4.26E-90
ERF	2.278345472	1.23E-243
ERFE	0.754055294	5.44E-12
ERG	0.808599719	2.90E-63
ERGIC1	1.845353534	1.25E-148
ERGIC2	1.476197273	3.89E-181
ERGIC3	1.810288332	2.88E-209
ERH	2.200276952	2.45307417228278e-316
ERI2	0.6713858	3.08E-44
ERI3	1.502204825	3.15E-174
ERICH1	0.691204347	2.74E-59
ERICH2	1.60212369	2.41E-188
ERICH3	-0.986383373	4.72E-21
ERICH4	0.44943592	3.42E-114
ERICH5	-0.686644771	2.31E-26
ERICH6	0.07829405	1.40E-12
ERICH6B	-0.194318485	4.43E-12
ERLEC1	1.850957285	2.59E-140
ERLIN1	2.388805156	6.81E-304
ERMAP	1.399033488	2.31E-144
ERMARD	0.459445356	4.33E-18
ERMN	-1.691321709	7.01E-35
ERMP1	0.329028222	4.06E-07
ERN1	0.892881626	1.77E-69
ERN2	0.056170073	2.16E-25
ERO1A	0.785542492	1.24E-38
ERO1B	0.396533531	2.63E-08
ERP29	1.273335936	9.08E-74
ERP44	2.123134369	2.02E-249
ERRFI1	1.270147149	5.47E-56
ERV3-1	1.169320918	3.91E-64
ERVFRD-1	0.006406505	0.379875427
ERVH48-1	-0.023784509	0.409939683
ERVK3-1	-2.106384639	1.28E-232
ERVMER34-1	0.484287498	5.19E-91
ERVV-1	-0.135572784	0.000881727
ERVV-2	0.044417527	1.10E-31
ERVW-1	-0.049057223	0.001549784
ESAM	1.666350054	1.12E-152
ESCO1	1.38437368	2.16E-115
ESD	1.745081476	2.44E-198
ESF1	1.080157888	1.43E-96
ESPN	-0.007059077	0.940352804
ESPNL	0.209311012	0.022842105

ESR1	0.025436311	0.504095798
ESR2	0.326339953	4.24E-41
ESRP1	-0.001339364	0.949673918
ESRP2	-0.12318405	9.61E-06
ESRRA	0.674825391	1.13E-26
ESRRB	0.403099143	1.05E-92
ESRRG	0.077955204	0.199428415
ESX1	0.064628683	4.91E-15
ESYT1	1.787472459	3.89E-109
ESYT2	0.901466397	9.37E-46
ESYT3	-1.260065765	4.52E-44
ETAA1	0.88845487	7.89E-51
ETF1	1.837554214	5.92E-251
ETFA	1.227201562	2.36E-135
ETFB	-1.308575338	2.04E-143
ETFBKMT	0.328746192	9.06E-20
ETFDH	0.29191035	2.96E-08
ETFRF1	1.272769246	4.50E-93
ETHE1	1.555171922	1.35E-196
ETNK1	0.573522887	1.12E-15
ETNK2	0.979537364	5.84E-49
ETNPPL	-2.643243092	8.24E-103
ETS2	0.470499578	9.34E-10
ETV1	2.579587678	2.58E-66
ETV2	1.167608338	3.18E-286
ETV3	1.436934025	1.07E-171
ETV3L	-0.029027057	0.317641048
ETV5	2.214668638	7.28E-205
ETV7	1.59842901	5.45E-285
EVA1C	1.326021192	2.07E-105
EVC	1.740075498	7.48E-275
EVI2A	-0.233453245	0.074162421
EVI2B	2.688738977	7.96E-174
EVI5	1.314029941	2.84E-147
EVI5L	-0.511872973	5.67E-18
EVL	-0.353201875	1.39E-05
EVPL	-0.361009173	3.80E-30
EVPLL	-0.018276695	0.000150268
EVX1	0.241989903	9.73E-105
EWSR1	-0.482956787	2.26E-11
EXD1	-0.077252196	1.69E-07
EXD2	1.195122846	2.59E-90
EXD3	0.137568906	0.031775439
EXO5	0.302500405	2.79E-06
EXOC1	0.468213646	8.39E-14
EXOC2	1.313127523	4.37E-121
EXOC3	0.53342776	2.69E-17
EXOC3L1	1.634581496	2.23E-230

EXOC3L4	0.044744545	1.24E-08
EXOC4	2.005676677	6.30E-227
EXOC5	1.104712258	1.10E-99
EXOC6	0.653731212	6.16E-26
EXOC6B	0.351043539	4.07E-12
EXOC7	-0.417516047	2.09E-09
EXOC8	0.969681683	5.17E-62
EXOG	1.090258138	7.10E-73
EXOSC1	-0.229884918	1.83E-05
EXOSC10	-0.158433669	0.065233391
EXOSC2	1.675741701	2.50E-108
EXOSC3	-0.197260384	0.00018212
EXOSC4	1.534538675	8.47E-169
EXOSC5	1.483218214	2.02E-124
EXOSC6	0.550502991	2.20E-14
EXOSC7	0.357740125	2.28E-08
EXOSC8	1.436340142	5.36E-119
EXOSC9	1.313553021	9.06E-68
EXPH5	-0.354054923	0.00723057
EXT1	1.91910585	7.29E-275
EXT2	1.466750033	1.22E-135
EXTL1	-0.690317298	2.86E-09
EXTL2	1.768083021	3.42E-118
EXTL3	1.202059635	4.17E-76
EYA1	-0.054534599	0.628504019
EYA2	2.20615397	6.89E-207
EYA3	0.918130903	4.00E-37
EYA4	1.516816931	8.15E-166
EYS	-0.018945081	0.083070186
EZH1	-0.738055784	3.27E-17
EZR	2.214758527	1.27E-89
F10	0.208635338	3.16E-09
F11	-0.005658546	0.542211355
F11R	1.080880708	3.07E-69
F12	0.817630216	1.21E-20
F13A1	3.537002469	1.22626828730585e-313
F13B	0.087316155	5.57E-93
F2	-0.349708263	7.15E-17
F2RL2	1.620098346	2.93E-173
F2RL3	0.985880319	1.49E-197
F3	2.474464729	1.87E-88
F5	0.427238669	8.52E-48
F7	-0.485653332	3.50E-28
F8	1.001161912	2.04E-79
F8A1	0.768274589	7.98E-35
F8A2	0.023827515	3.76E-11
F9	-0.009763884	0.002502849
FA2H	-1.223444126	6.44E-21

FAAH	-1.547556716	2.91E-83
FAAH2	-0.474931178	2.97E-10
FAAP100	1.662740729	2.84E-61
FAAP20	1.11656401	3.14E-116
FABP1	-0.219836389	1.34E-16
FABP12	0.128000267	2.93E-27
FABP2	0.003010043	0.426536291
FABP3	-0.270555529	0.011400993
FABP4	0.087133813	0.219990805
FABP5	1.284418985	2.52E-57
FABP6	-2.369927798	2.96E-127
FABP7	3.587644201	2.46E-205
FABP9	0.126296133	5.72E-85
FADD	1.106979343	5.90E-81
FADS1	1.165362883	1.28E-87
FADS2	1.523079946	3.67E-127
FADS3	-0.11219633	0.099052139
FADS6	-1.824549519	6.59E-62
FAF1	1.549777072	4.10E-149
FAF2	1.794538883	2.69E-161
FAH	1.128618603	1.73E-118
FAHD1	1.542151022	1.67E-116
FAHD2A	0.402336382	1.18E-07
FAHD2B	-0.33913828	3.82E-07
FAIM	0.476007084	2.90E-21
FAIM2	-1.452490539	4.11E-65
FAM102A	0.030470323	0.657559753
FAM102B	0.1576223	0.117327308
FAM104A	0.869675355	1.61E-52
FAM104B	1.544833264	3.33E-236
FAM107A	-0.470981577	4.73E-12
FAM107B	0.072881929	0.435073986
FAM110A	1.737458236	2.34E-209
FAM110B	1.289159918	2.11E-58
FAM110C	1.212840941	6.41E-65
FAM110D	0.311106496	1.15E-13
FAM117A	0.639171513	4.46E-17
FAM117B	0.494276703	1.56E-13
FAM118A	0.24912277	0.005134149
FAM118B	1.464694087	6.94E-164
FAM120A	1.698457857	1.98E-159
FAM120AOS	0.60753694	1.29E-42
FAM120B	1.066842155	1.17E-64
FAM120C	1.588639665	4.19E-262
FAM124A	-0.080159046	0.31475903
FAM124B	1.110911294	3.93E-207
FAM126B	-0.672791063	4.14E-14
FAM131A	0.073694972	0.44236962

FAM131B	0.462652165	2.04E-05
FAM131C	-1.847025575	1.62E-46
FAM133A	-0.953394023	4.46E-69
FAM133B	0.987099972	6.78E-52
FAM135A	0.667551426	1.98E-27
FAM135B	-0.046864446	0.564351338
FAM136A	1.248210633	3.05E-77
FAM13A	0.418918316	6.43E-07
FAM13B	0.345197476	6.52E-06
FAM13C	-0.527998312	1.07E-14
FAM149A	-0.010097178	0.877516954
FAM149B1	-0.241845271	5.47E-09
FAM151A	0.464105709	3.75E-153
FAM151B	0.938801701	6.22E-252
FAM153A	-1.94883066	1.57E-70
FAM153B	-2.680382519	8.67E-88
FAM155A	-0.885020997	3.07E-35
FAM155B	-0.408634904	7.25E-08
FAM156A	0.034163415	0.330194361
FAM160A1	0.041393065	0.429520696
FAM160A2	0.927518781	8.21E-36
FAM160B1	0.634017128	2.34E-23
FAM160B2	0.04619815	0.560314118
FAM161A	0.760222644	1.24E-55
FAM161B	-0.021817436	0.740413785
FAM162A	1.110009142	4.07E-79
FAM162B	1.014891551	3.37E-74
FAM163A	-0.610958449	1.80E-17
FAM163B	-1.112072437	1.82E-12
FAM166A	-0.105334792	0.016573289
FAM166B	0.585726689	8.60E-12
FAM167A	0.723974947	1.53E-25
FAM167B	0.859343003	9.56E-49
FAM168A	0.802752267	2.16E-37
FAM168B	0.573079267	4.97E-19
FAM169A	0.202295153	0.009179368
FAM170A	-0.0408432	1.31E-06
FAM170B	-0.003504457	0.593405651
FAM171A1	-0.734408118	6.98E-24
FAM171A2	0.23355233	0.028087341
FAM171B	0.628498265	2.17E-18
FAM172A	1.485213064	7.65E-120
FAM174A	0.542138385	8.88E-17
FAM174B	-0.268704316	1.28E-05
FAM177A1	0.206193743	0.000339971
FAM178B	0.049509871	0.456889482
FAM180B	0.346185393	5.97E-09
FAM181A	2.224708962	1.60E-190

FAM181B	2.390724948	1.98E-276
FAM183A	1.545306597	8.98E-41
FAM184A	-0.47011949	1.33E-15
FAM184B	0.269407634	2.03E-09
FAM185A	1.081392946	3.67E-103
FAM186A	0.01314271	0.406086596
FAM186B	0.155729607	6.46E-41
FAM187B	-0.047715114	9.02E-07
FAM189A1	-1.034805481	2.11E-27
FAM189A2	0.13934304	0.078390405
FAM189B	0.03054859	0.70321296
FAM193A	1.205252046	3.66E-74
FAM193B	0.188359349	0.021685791
FAM199X	0.942453099	1.21E-60
FAM200A	1.809028314	1.57E-203
FAM200B	-0.211804136	5.17E-05
FAM204A	0.43623923	1.29E-25
FAM205A	-0.006513229	0.285601553
FAM205C	-0.040180564	5.02E-06
FAM207A	1.34693925	1.42E-95
FAM209A	-0.011644991	0.636561301
FAM209B	0.188720835	8.19E-09
FAM20B	0.585379155	1.04E-18
FAM20C	2.538013426	2.58E-273
FAM210A	1.763898414	8.39E-283
FAM210B	1.652793488	3.21E-183
FAM214A	0.3942688	1.17E-07
FAM214B	0.882665397	1.37E-69
FAM216A	0.07262806	0.440439149
FAM216B	-0.036159083	0.644705555
FAM217A	0.009306035	0.096570598
FAM217B	0.519002647	1.33E-14
FAM219A	0.074051084	0.225996252
FAM219B	-0.559441746	2.55E-13
FAM220A	1.627640103	4.45E-92
FAM221A	0.542849937	1.74E-23
FAM221B	-0.085570885	2.80E-13
FAM222A	0.092771298	0.296625419
FAM222B	1.234735237	6.41E-76
FAM227A	0.422411375	6.94E-20
FAM227B	0.765047334	2.36E-97
FAM228A	-0.575622403	1.97E-61
FAM228B	-0.653467054	6.64E-42
FAM229A	-0.694058822	2.95E-19
FAM229B	1.279354413	4.93E-100
FAM234A	1.814477945	1.68E-131
FAM234B	0.111119469	0.253148997
FAM236A	0.005630383	0.505928069

FAM236B	0.016544013	7.61E-17
FAM236D	0.022300837	1.41E-06
FAM24A	-0.001237841	0.759671577
FAM24B	0.153469182	0.002719575
FAM25A	-0.073758005	3.79E-08
FAM25C	-0.006986568	0.099629006
FAM25G	0.002979516	0.244725025
FAM32A	1.868343332	2.17E-210
FAM3A	0.189943046	4.16E-05
FAM3B	0.646445122	5.48E-304
FAM3C	1.733832031	2.19E-88
FAM3D	0.279922479	1.57E-91
FAM43A	0.914776497	1.24E-41
FAM43B	0.413600666	4.58E-07
FAM47A	-0.004248153	0.012965889
FAM47B	-0.001574271	0.18508071
FAM47C	-0.018501541	2.55E-06
FAM47E	-0.657217709	6.72E-33
FAM50A	0.740804578	6.16E-31
FAM50B	-0.301838163	8.50E-05
FAM53A	-0.102388066	0.009451143
FAM53B	-0.020013894	0.768307127
FAM53C	0.573983739	1.95E-13
FAM71A	-0.030059563	0.011978555
FAM71B	-0.006069572	0.002114693
FAM71C	-0.052239808	1.01E-06
FAM71D	0.161743446	1.75E-48
FAM71E1	-1.007699432	8.08E-53
FAM71E2	-0.111391262	1.43E-08
FAM71F1	0.180627944	3.96E-11
FAM71F2	0.092330929	0.147007596
FAM72A	0.556581819	3.14E-82
FAM72B	0.964801676	2.69E-149
FAM72C	0.510215004	6.52E-90
FAM72D	0.6854598	5.56E-145
FAM76A	1.195044441	2.70E-82
FAM76B	0.996254552	2.84E-44
FAM78A	1.330029835	1.08E-115
FAM78B	0.520040907	8.21E-09
FAM81A	-0.341678272	0.003847691
FAM81B	0.385053344	1.95E-07
FAM83A	0.077067456	1.30E-41
FAM83B	-0.011480816	0.004237755
FAM83C	-0.01303059	0.054844542
FAM83E	0.137214256	1.81E-08
FAM83F	-0.042801535	1.65E-11
FAM83G	0.160676709	6.08E-06
FAM83H	-0.190058411	0.001736

FAM86B1	0.199724174	1.04E-05
FAM86B2	0.379692949	1.65E-35
FAM89A	0.712243373	2.73E-28
FAM89B	-1.447151122	3.23E-139
FAM8A1	1.260969811	7.46E-72
FAM90A1	0.32690654	5.40E-10
FAM90A26	0.038562419	7.10E-20
FAM91A1	1.668527505	8.24E-134
FAM98A	0.561073363	2.54E-20
FAM98B	1.026874419	4.75E-63
FAM98C	-0.39496299	5.20E-10
FAM9A	-0.006146499	0.559334565
FAM9B	-0.022052387	0.002136343
FAM9C	0.00849674	0.030262372
FAN1	-0.413200646	6.14E-09
FANCA	1.339387771	5.16E-94
FANCC	1.11937801	1.15E-32
FANCD2OS	0.124692023	9.37E-200
FANCE	2.047929539	6.08E-203
FANCG	-2.122961991	6.54E-141
FANCI	2.001474498	2.59E-301
FANCL	0.744155331	9.38E-29
FANCM	0.58848891	3.04E-51
FANK1	0.292009279	6.11E-06
FAP	1.158474009	1.12E-205
FAR1	0.580021719	7.43E-13
FAR2	1.419509465	4.35E-82
FARP1	0.45417505	7.70E-15
FARP2	0.844733682	3.70E-35
FARS2	1.376243439	1.85E-162
FARSA	1.462555901	9.25E-83
FARSB	0.863831591	1.86E-34
FAS	1.633467823	1.45E-117
FASLG	0.270538154	3.28E-83
FASN	0.22732281	0.024601234
FASTK	0.802026492	3.94E-32
FASTKD1	0.607898755	1.32E-24
FASTKD2	1.088784136	1.92E-69
FASTKD3	1.591921944	1.08E-185
FASTKD5	1.518622281	2.46E-94
FAT1	1.723438219	1.90E-144
FAT2	-1.500091437	2.24E-11
FAT3	0.726457401	6.88E-22
FAT4	0.360553593	4.33E-26
FATE1	0.10889029	0.005977511
FAU	1.351721734	1.04E-171
FAXC	-0.150504057	0.085001843
FAXDC2	0.072817726	0.346151116

FBF1	-1.052252512	1.07E-91
FBL	1.733953933	5.39E-216
FBLL1	-0.791698785	1.52E-15
FBLN1	1.909311754	3.68E-162
FBLN2	0.39701561	9.87E-12
FBLN5	2.358343915	3.07E-159
FBLN7	1.728766958	4.24E-98
FBN1	1.414525641	3.15E-131
FBN3	1.334747651	2.50E-251
FBP2	-0.024667113	0.322970411
FBRS	0.736042761	8.66E-30
FBRSL1	0.095148545	0.294195736
FBXL12	1.779545992	2.41E-158
FBXL13	0.724511502	2.69E-255
FBXL14	1.01151344	1.18E-49
FBXL15	-1.131830596	1.04E-54
FBXL16	-3.1283558	8.99E-96
FBXL17	0.970188406	5.39E-55
FBXL18	0.714167795	1.38E-23
FBXL19	-0.396290309	1.23E-06
FBXL2	-0.992198794	1.73E-31
FBXL20	0.519270658	1.08E-16
FBXL22	-0.155999414	0.001371384
FBXL3	0.153775384	0.014668608
FBXL4	0.996691176	1.80E-86
FBXL5	1.845994184	3.03E-204
FBXL6	-0.245744915	0.023729435
FBXL7	1.918898202	4.73E-188
FBXL8	-0.654604541	4.33E-14
FBXO10	0.341759465	2.52E-08
FBXO11	-0.047489564	0.633131163
FBXO15	0.195441489	3.44E-08
FBXO16	-0.633888833	1.06E-14
FBXO17	1.053555185	1.92E-35
FBXO21	1.241684968	7.28E-71
FBXO22	0.993791397	1.54E-36
FBXO24	-0.010395639	0.691160205
FBXO25	0.572428385	5.41E-19
FBXO27	-1.011941234	1.23E-33
FBXO28	1.362813645	4.98E-96
FBXO3	0.596185905	4.67E-19
FBXO30	1.637487011	4.59E-303
FBXO31	0.100338372	0.394328153
FBXO32	2.669200882	3.42E-155
FBXO33	0.599500063	7.26E-23
FBXO34	0.821134684	8.31E-23
FBXO36	1.430490262	8.29E-190
FBXO38	0.564504429	6.56E-18

FBXO40	-0.232413463	7.66E-10
FBXO41	-1.438776104	5.94E-32
FBXO42	1.306464978	2.45E-73
FBXO44	-0.740481629	1.49E-14
FBXO45	0.744624651	1.75E-34
FBXO46	1.262210603	3.99E-139
FBXO47	0.011966826	0.015528043
FBXO48	0.512301758	5.22E-60
FBXO5	2.358888649	1.76E-277
FBXO6	1.202313126	5.24E-116
FBXO7	-0.314767234	7.87E-09
FBXO9	0.362732754	2.84E-05
FBXW10	-0.100906203	0.01751195
FBXW11	0.983669749	3.97E-67
FBXW12	0.058549368	0.025901505
FBXW2	1.119453633	3.99E-59
FBXW4	-0.827288476	1.86E-64
FBXW5	0.825434721	4.82E-35
FBXW7	-0.92676648	1.67E-16
FBXW8	1.89253403	5.77E-219
FBXW9	1.995492184	6.16E-179
FCAMR	0.123504771	2.84E-49
FCAR	0.649643888	3.17E-95
FCER1A	1.364852708	1.16E-236
FCER1G	4.483061914	1.18E-248
FCER2	0.323031802	1.76E-50
FCF1	1.864527943	7.03E-272
FCGR1A	3.267874592	1.03E-209
FCGR1B	1.350759154	4.93E-90
FCGR2A	3.391267322	5.15E-209
FCGR2B	1.782726462	4.87E-190
FCGR3A	4.834227343	1.97E-227
FCGR3B	1.159174887	1.88E-94
FCHO1	-1.39552639	2.75E-89
FCHO2	1.285899933	7.31E-82
FCHSD1	0.44615172	2.02E-11
FCHSD2	0.872133324	8.05E-94
FCN1	0.350505482	5.75E-36
FCN2	0.016844585	0.373094198
FCN3	0.70541288	2.63E-55
FCRL1	0.027510175	6.25E-11
FCRL2	0.001366373	0.774966875
FCRL3	0.043127951	5.42E-16
FCRL4	0.001189265	0.623608393
FCRL5	0.035205091	1.85E-15
FCRL6	0.273078663	1.34E-22
FCRLA	0.953674521	1.44E-197
FCRLB	0.91678616	1.32E-72

FDCSP	0.042402737	0.128789472
FDFT1	1.18005479	2.42E-62
FDPS	0.077099094	0.268661898
FDX1	1.917734098	1.38E-286
FDXACB1	0.937213615	2.26E-170
FDXR	1.405863761	3.72E-63
FECH	0.60522293	1.01E-33
FEM1A	0.129423556	0.012661401
FEM1B	1.215653882	3.92E-60
FEM1C	2.157062993	1.77E-192
FEN1	2.392376678	6.74E-161
FER	1.114395982	8.56E-132
FER1L5	-0.224165731	6.93E-12
FER1L6	0.057082565	5.84E-07
FERD3L	-0.017526625	0.239178714
FERMT1	2.00947044	4.12E-263
FERMT2	1.320900717	1.78E-115
FERMT3	2.969606751	2.76676761671098e-322
FES	1.547829543	1.00E-150
FETUB	0.263244728	9.65E-92
FEV	0.434125138	3.45E-17
FEZ1	-0.418693917	7.97E-10
FEZ2	0.348909838	9.94E-10
FEZF1	0.045393922	0.616274126
FEZF2	-1.060031697	8.82E-14
FFAR1	-0.612535017	3.13E-29
FFAR2	0.289669342	7.16E-31
FFAR3	0.053078111	2.49E-06
FFAR4	0.985119671	9.04E-78
FGA	-0.169283568	3.54E-09
FGB	-0.212430671	9.92E-10
FGD1	0.525995305	7.28E-18
FGD2	0.725435414	1.85E-18
FGD3	1.79107779	5.30E-288
FGD4	0.504153248	2.34E-28
FGD5	0.810375901	1.56E-84
FGD6	0.040666539	0.371457203
FGF1	0.459788055	3.04E-08
FGF10	0.212021377	9.87E-15
FGF11	-1.728548443	2.16E-201
FGF12	-0.247565256	0.018622786
FGF13	-1.151332897	2.98E-23
FGF14	-0.682684762	8.45E-12
FGF16	-0.047790762	5.10E-05
FGF17	-2.728762548	2.44E-74
FGF18	-0.005481315	0.887997322
FGF19	0.080664547	0.000433372
FGF2	0.863268873	7.77E-35

FGF20	0.242081591	5.49E-05
FGF21	0.039031232	5.86E-05
FGF22	-1.678036525	2.18E-196
FGF23	0.006953018	0.000705865
FGF3	-0.671224842	4.35E-06
FGF4	-0.005202174	0.42263166
FGF5	-0.349740461	4.45E-05
FGF6	0.014027203	2.77E-07
FGF7	0.137701267	1.27E-14
FGF8	-0.116193883	4.07E-05
FGF9	-1.275245696	1.39E-27
FGFBP1	-0.05818892	8.33E-06
FGFBP2	2.385370772	9.71E-296
FGFBP3	0.967015819	3.90E-97
FGFR1	0.344162297	0.000936696
FGFR1OP2	0.077196652	0.227192614
FGFR2	-0.37096032	8.75E-06
FGFR3	-0.582366392	2.91E-09
FGFR4	0.081046061	0.342958861
FGFRL1	0.988988977	8.36E-31
FGG	-0.145674342	6.54E-06
FGGY	0.835185445	6.67E-73
FGL1	0.065420924	0.085801002
FGL2	2.003555495	5.62E-119
FGR	1.473131939	4.22E-113
FH	1.571460613	4.74E-131
FHAD1	0.406222839	2.58E-22
FHDC1	0.74481197	1.06E-23
FHIT	0.296406216	1.25E-10
FHL1	1.892211124	2.84E-160
FHL2	1.017210224	5.64E-44
FHL5	0.910406978	2.23E-48
FHOD1	0.993906938	6.78E-52
FHOD3	0.286682953	0.000656871
FIBCD1	1.05382506	1.62E-36
FIBP	0.812691526	2.40E-33
FICD	1.662354128	5.04E-266
FIG4	-1.629663558	4.27E-120
FIGLA	0.0763158	1.29E-57
FIGN	1.680636987	1.04E-267
FIGNL1	1.742562016	3.84E-151
FIGNL2	-0.282386404	1.35E-11
FILIP1	-0.205438364	1.05E-05
FIP1L1	-0.646770971	1.92E-28
FIS1	1.724934122	7.29E-185
FITM1	-0.173437181	9.60E-05
FITM2	1.448924604	4.44E-94
FIZ1	0.811839673	8.99E-53

FKBP11	0.45353726	6.76E-12
FKBP14	1.601752331	2.09E-112
FKBP15	1.319302326	2.36E-100
FKBP1A	0.375632958	1.06E-05
FKBP1B	-2.229610019	1.40E-122
FKBP3	-0.501803536	1.30E-13
FKBP4	0.489967372	3.66E-07
FKBP5	2.543164579	9.05E-139
FKBP6	-0.004868837	0.531225263
FKBP8	0.300286926	1.09E-07
FKBPL	2.150979828	5.62E-296
FKRP	1.083809451	6.69E-83
FKTN	0.090370086	0.132553835
FLCN	-0.744535048	3.14E-12
FLG	-0.116055664	6.40E-10
FLG2	-0.124093649	1.11E-12
FLI1	1.052105875	6.74E-69
FLII	1.211275208	8.40E-51
FLNA	2.975031491	4.120507486316e-321
FLNB	-0.350903984	9.85E-06
FLNC	3.330121107	3.84E-263
FLOT1	1.056456446	8.80E-66
FLOT2	1.68178055	1.53E-137
FLRT1	-0.244265934	0.000239951
FLRT2	-0.23120345	0.000401027
FLRT3	0.986419644	1.55E-24
FLT1	0.122431968	0.069880227
FLT3	-0.968423399	1.43E-13
FLT3LG	-1.165921569	5.46E-212
FLT4	1.177114399	6.19E-61
FLVCR1	0.864832	1.80E-35
FLVCR2	2.103543181	4.27E-283
FLYWCH1	0.001062424	0.992256926
FLYWCH2	1.429187469	8.76E-115
FMC1	-1.386714038	7.39E-188
FMN1	0.106984844	0.054814681
FMN2	0.787967795	6.99E-40
FMNL1	-0.811207217	1.72E-22
FMNL2	0.332518319	2.54E-06
FMNL3	1.503316777	5.55E-138
FMO1	0.609039535	2.78E-233
FMO2	-0.120163584	0.026639988
FMO3	0.525536007	3.13E-53
FMO4	1.547488038	4.52E-180
FMO5	0.713301973	5.38E-68
FMR1	0.363618082	2.83E-10
FMR1NB	-0.002534327	0.764273568
FN3K	-0.643072881	2.64E-22

FN3KRP	1.104215274	5.46E-93
FNBP1	-0.249693908	0.007011873
FNBP1L	1.374410141	3.49E-80
FNBP4	0.331826322	0.000597969
FNDC1	0.569665718	1.11E-32
FNDC10	-0.890039641	3.65E-33
FNDC11	0.597985394	2.99E-130
FNDC3A	1.740597495	6.71E-149
FNDC4	1.30687765	3.78E-68
FNDC5	-0.709403361	2.76E-10
FNDC7	0.646438743	1.26E-117
FNDC8	0.348966005	2.11E-98
FNDC9	-2.760151431	6.39E-103
FNIP1	1.050953554	4.43E-66
FNIP2	0.466386947	2.75E-16
FNTA	0.572120937	8.11E-22
FNTB	-0.205097361	0.025655976
FOCAD	0.121355885	0.16029803
FOLH1	-0.630232678	1.31E-07
FOLR1	2.235482557	4.55E-186
FOLR3	0.522169345	2.44E-154
FOS	2.445866795	2.68E-71
FOSB	1.022399514	1.60E-18
FOSL1	2.794951116	6.08E-206
FOSL2	1.761567492	3.84E-90
FOXA1	0.088527382	0.026852912
FOXA2	0.26907496	1.41E-14
FOXA3	0.537611597	4.63E-214
FOXB1	-0.049375549	0.469460967
FOXB2	0.066484769	4.58E-05
FOXC1	1.30167785	2.54E-112
FOXC2	0.510870975	4.51E-34
FOXD2	0.765285956	2.91E-202
FOXD4	-0.057156058	0.345705775
FOXD4L1	0.094607582	0.013053332
FOXD4L3	-0.043492885	0.003314481
FOXD4L4	-0.074527375	8.30E-06
FOXD4L5	0.011724748	0.021244088
FOXD4L6	-0.158446815	1.10E-14
FOXE1	0.060922574	5.09E-09
FOXE3	-0.083832849	0.005826102
FOXF1	0.510837725	1.01E-16
FOXF2	1.205791321	5.90E-138
FOXG1	2.702704927	1.42E-54
FOXH1	-0.039711636	0.057099033
FOXI1	0.028550605	7.84E-09
FOXI2	-0.001099744	0.885970853
FOXI3	0.054123516	4.71E-24

FOXJ1	3.300721725	2.50E-166
FOXJ2	1.130267733	5.99E-38
FOXJ3	1.145705166	5.97E-51
FOXK1	1.121092968	1.89E-73
FOXK2	0.974847034	7.74E-53
FOXL1	0.786860361	1.52E-175
FOXL2	0.697439713	1.38E-165
FOXL2NB	0.304180483	8.86E-34
FOXN1	-0.016202852	0.003920775
FOXN2	1.36993098	4.09E-62
FOXN3	0.789650644	4.51E-16
FOXN4	0.740889418	3.51E-200
FOXO1	1.654408565	7.69E-125
FOXO3	0.792903836	1.83E-21
FOXO4	-1.164721893	9.26E-96
FOXO6	1.145694681	8.37E-62
FOXP1	-1.208896684	9.64E-82
FOXP2	0.092871292	0.037755917
FOXP3	0.255412772	1.94E-15
FOXP4	0.5509922	9.07E-21
FOXQ1	-0.229142061	1.78E-05
FOXR1	0.113584641	1.88E-41
FOXR2	0.004298464	0.002987355
FOXRED1	-0.220467152	0.000621007
FOXRED2	1.679265964	1.92E-129
FOXS1	1.826828832	1.64E-243
FPGS	1.977115552	5.66E-210
FPR1	4.207014636	2.08E-281
FPR2	1.220246693	2.62769360523117e-314
FRA10AC1	-0.712445562	6.86E-48
FRAS1	-0.265892295	2.07E-05
FRAT1	0.798339023	6.99E-37
FRAT2	0.762706664	8.75E-40
FREM1	0.569024058	9.23E-09
FREM3	0.159085776	0.003843139
FRG1	1.839087221	1.47E-183
FRG2	-0.056561038	0.006857303
FRG2B	-0.067881757	4.58E-05
FRG2C	0.075497939	0.000193044
FRK	-0.058357067	0.044859097
FRMD1	-0.02585058	0.011164484
FRMD4A	-0.395797966	3.69E-16
FRMD4B	0.67910655	1.29E-16
FRMD5	0.812152207	1.16E-40
FRMD6	1.403228617	1.56E-73
FRMD7	0.03610104	3.11E-05
FRMD8	2.027914424	7.37E-207
FRMPD1	-0.172082595	7.69E-05

FRMPD2	-0.707815059	1.29E-35
FRMPD3	1.180707833	7.84E-39
FRMPD4	-1.547046878	5.12E-58
FRRS1L	-2.588827074	5.48E-116
FRS2	1.402455731	2.96E-128
FRS3	-0.485747198	2.13E-10
FRY	-0.561998897	3.78E-10
FRYL	0.482109688	3.92E-14
FRZB	2.495006634	1.79E-272
FSBP	1.291757975	9.22E-215
FSCB	-0.002831338	0.125134596
FSCN1	2.563531972	8.91E-204
FSCN2	-0.011021812	0.784429853
FSCN3	0.016251071	0.584037079
FSD1	-0.389114342	1.16E-05
FSD1L	-0.235188595	7.12E-06
FSD2	-0.742769549	1.11E-53
FSHB	-0.077785053	0.000316888
FSHR	-0.011364216	0.127318239
FSIP1	0.880399085	2.00E-128
FSIP2	0.029380735	0.000283655
FST	2.148035679	1.87E-306
FSTL3	1.011973127	4.11E-72
FSTL4	-1.137425014	2.14E-33
FSTL5	-2.306186847	3.35E-61
FTCD	-1.441517985	9.43E-79
FTCDNL1	1.362274814	1.96E-180
FTH1	-1.79554075	5.41E-125
FTHL17	-0.000247217	0.88739838
FTL	2.345161903	8.25E-227
FTMT	-0.000727403	0.787356301
FTO	1.0700458	1.17E-68
FTSJ1	1.879900641	5.62E-155
FTSJ3	1.444864172	2.24E-114
FUBP1	1.038692567	3.79E-33
FUBP3	1.367366958	7.88E-124
FUCA1	2.586377589	1.51389756900514e-311
FUCA2	2.220990224	2.33E-278
FUNDC1	1.238892867	5.05E-96
FUNDC2	1.306742202	5.26E-125
FURIN	1.322514258	1.67E-65
FUS	1.321334483	8.09E-60
FUT1	-0.481534307	2.64E-07
FUT10	1.0106759	5.30E-101
FUT11	1.160115915	3.54E-98
FUT2	-0.014118303	0.819266262
FUT3	0.158191546	1.75E-16
FUT4	0.790471685	2.22E-79

FUT5	-0.048474871	8.19E-06
FUT6	0.010439707	0.04542803
FUT7	-0.236609492	0.000434718
FUT8	0.944749261	3.16E-48
FUT9	-1.183692248	1.32E-44
FUZ	0.660266446	8.48E-35
FXN	-0.041672159	0.31970703
FXR1	0.843006636	1.01E-53
FXR2	-0.109657898	0.119699316
FXYD2	-0.065743278	0.032860177
FXYD3	-0.906846121	5.56E-98
FXYD4	-0.55639188	1.05E-21
FXYD5	2.932566629	3.91715006648774e-319
FXYD6	0.24611921	0.043023628
FXYD7	-3.763374175	1.27E-163
FYCO1	1.153409633	1.17E-84
FYN	1.613171201	3.09E-234
FYTTD1	1.784064909	7.20E-182
FZD1	2.249039685	1.86E-209
FZD10	0.199185298	3.07E-08
FZD2	2.436039959	2.16001341317186e-315
FZD3	1.453756473	1.06E-82
FZD4	0.752070024	2.20E-64
FZD5	1.770574233	4.42E-262
FZD6	1.373346582	2.06E-127
FZD7	2.627919268	2.70E-68
FZD8	1.211397426	1.40E-63
FZD9	-0.674261832	4.01E-58
FZR1	0.92405934	8.64E-30
G0S2	2.251032989	7.66E-149
G2E3	0.835253488	6.61E-48
G3BP1	1.280772941	1.06E-117
G3BP2	0.124242611	0.19322061
G6PC2	0.047519056	4.72E-06
G6PC3	1.328076234	3.74E-132
G6PD	1.331317732	1.92E-109
GAA	2.03731901	7.92E-185
GAB1	1.773476181	1.55E-140
GAB2	0.688639059	4.66E-20
GAB3	1.627748835	3.68E-236
GABARAPL1	-0.800038652	4.62E-17
GABARAPL2	1.127483983	3.17E-81
GABBR1	-1.72552205	4.02E-82
GABBR2	-0.546896896	0.000481788
GABPA	1.349426154	2.37E-107
GABPB1	1.138156345	3.91E-66
GABPB2	0.049149451	0.430527367
GABRA1	-2.48709343	1.73E-54

GABRA2	-1.437614722	1.65E-58
GABRA3	-0.159048393	0.178289932
GABRA4	-1.298292469	2.19E-38
GABRA5	-1.937854756	6.14E-26
GABRA6	-1.736768565	3.93E-13
GABRB1	0.118554264	0.058128912
GABRB2	-2.687142904	7.58E-69
GABRB3	-1.493300887	1.24E-44
GABRD	-3.255201516	3.28E-56
GABRE	-0.117233642	0.176392507
GABRG1	-2.750803036	9.72E-203
GABRG2	-1.994432229	3.39E-59
GABRG3	-0.650853197	3.60E-29
GABRP	0.035487059	6.27E-12
GABRQ	0.796497335	1.06E-12
GABRR1	0.224596745	1.56E-72
GABRR2	0.584339339	3.29E-131
GABRR3	0.072467142	4.66E-96
GAD1	-1.658057215	8.64E-35
GAD2	-2.485526377	1.50E-64
GADD45A	3.162046446	2.72E-201
GADD45B	1.319773314	1.33E-32
GADD45G	1.613686674	5.11E-63
GADD45GIP1	1.34916482	6.09E-136
GADL1	0.044065737	1.13E-05
GAGE1	0.007558306	8.85E-06
GAGE10	-0.04449837	0.164344748
GAGE12B	0	1
GAGE12C	0	1
GAGE12D	0	1
GAGE12E	0.001261122	0.0278599
GAGE12F	0	1
GAGE12G	0	1
GAGE12H	0.011900529	5.26E-05
GAGE12J	0.000457034	0.863634978
GAGE13	0.002347613	0.025679673
GAGE2A	0.082272352	2.54E-19
GAGE2E	0.013798229	4.35E-05
GAK	-0.583071402	2.74E-14
GAL	1.340875552	1.30E-31
GAL3ST1	-0.369891796	3.89E-05
GAL3ST2	0.35405462	4.98E-40
GAL3ST3	0.047576311	0.577736762
GALC	1.069135306	2.07E-74
GALE	1.534335078	2.33E-132
GALK2	1.004034696	3.93E-147
GALNS	0.825737009	1.38E-41
GALNT11	0.393549228	9.44E-09

GALNT12	-0.243325221	0.079106364
GALNT13	-0.100814751	0.291586454
GALNT14	0.209728324	0.00070107
GALNT15	1.978228924	1.55E-89
GALNT16	-0.404876228	1.02E-07
GALNT18	0.465814982	3.13E-11
GALNT3	0.777171064	1.10E-48
GALNT4	-1.092718724	4.60E-189
GALNT5	0.704499318	2.27E-17
GALNT6	0.391784432	1.08E-06
GALNT7	0.392401661	8.28E-05
GALNT8	0.314442579	0.000410939
GALNT9	-1.959428874	5.30E-29
GALNTL5	-0.344180859	3.52E-10
GALNTL6	-0.636184496	1.39E-36
GALP	0.013050318	0.35397565
GALR1	1.344644835	1.13E-238
GALR2	0.091075762	0.000140827
GALR3	-0.100856785	0.001463467
GALT	-1.25200996	1.83E-71
GAMT	1.020772909	2.85E-96
GAN	0.499917813	2.27E-28
GANAB	2.594250783	6.84E-274
GANC	0.860050482	5.36E-56
GAP43	2.41324947	3.46E-63
GAPDH	1.717862374	1.60E-165
GAPDHS	0.022896293	0.049935007
GAPVD1	1.211647198	1.79E-76
GAR1	1.642847778	3.42E-237
GAREM1	0.496723749	1.62E-15
GAREM2	0.809068904	5.14E-38
GARNL3	-1.500924237	4.12E-57
GART	2.106641806	6.35E-287
GAS2	0.314355465	1.78E-07
GAS2L1	0.846411066	7.07E-59
GAS2L2	-0.186390478	0.001015929
GAS6	-0.712702234	1.61E-31
GAS7	-0.568902037	7.95E-08
GAS8	-0.12857514	0.055298875
GAST	0.674199924	5.39E-106
GATA1	0.093465607	3.78E-09
GATA2	0.491072109	7.05E-23
GATA3	0.752993699	1.46E-105
GATA4	0.886140002	8.35E-274
GATA5	0.064905678	5.21E-22
GATA6	0.282766179	8.35E-51
GATAD1	0.906066823	2.09E-50
GATAD2A	1.773308916	7.85E-273

GATAD2B	1.129077682	3.53E-51
GATB	-0.59247991	6.34E-17
GATC	0.036752669	0.609538117
GATM	0.57876994	2.44E-20
GBA	2.517435811	1.18E-289
GBA2	0.389132316	4.03E-06
GBF1	-1.066303492	5.07E-38
GBGT1	0.826143952	2.76E-47
GBP2	3.107480534	2.60E-244
GBP3	2.76284478	7.81E-251
GBP4	1.717865898	8.03E-133
GBP5	1.293904926	2.17E-213
GBP6	0.325603212	1.15E-81
GBP7	0.221589515	1.04E-67
GBX1	0.121929722	2.79E-17
GBX2	1.25263589	5.73E-75
GC	-0.04144817	0.000202811
GCA	0.610559803	4.52E-18
GCAT	1.108977772	4.00E-89
GCC1	1.975158496	9.00E-238
GCC2	-0.110669155	0.071371427
GCDH	1.466502446	2.03E-167
GCFC2	0.921876942	2.08E-76
GCG	0.004821877	0.725328961
GCGR	0.067602113	0.000224276
GCH1	0.553154549	5.73E-16
GCHFR	-1.442016688	7.25E-84
GCK	0.351355448	1.31E-07
GCKR	0.435816046	9.18E-24
GCLC	0.446064935	1.02E-15
GCLM	1.480538057	1.55E-140
GCM1	0.142467014	7.12E-35
GCM2	0.033996825	3.85E-08
GCN1	1.463065022	3.10E-70
GCNA	-0.063400904	0.337085792
GCNT1	0.290248092	1.79E-05
GCNT2	1.140338883	4.71E-84
GCNT3	0.024562932	2.07E-05
GCNT4	-0.13017339	0.048289252
GCNT7	0.186843786	4.49E-36
GCSAM	0.184664675	1.16E-107
GCSAML	-0.382912337	7.22E-06
GCSH	-1.660604359	1.60E-232
GDA	-0.790109404	5.14E-06
GDAP1	-2.61475092	1.05E-184
GDAP1L1	-0.018957696	0.86971598
GDAP2	1.236633054	2.50E-254
GDE1	0.886441163	7.14E-53

GDF10	-1.401058741	5.94E-59
GDF11	0.56790229	2.49E-42
GDF2	-0.019244818	0.000377757
GDF3	0.222233518	1.68E-62
GDF5	0.644692924	8.32E-158
GDF6	0.054211683	0.026890278
GDF7	0.086265386	0.019753029
GDF9	0.523789845	2.25E-65
GDI1	0.320912376	2.21E-05
GDI2	1.988162494	8.41E-199
GDNF	-0.118810768	0.078410355
GDPD1	0.871163587	1.72E-28
GDPD2	2.434624207	1.42E-175
GDPD3	-0.147334317	0.139210785
GDPD4	0.130456608	1.97E-77
GDPD5	-0.638973102	1.03E-09
GDPGP1	0.379905809	6.27E-30
GEM	2.872973947	1.64E-188
GEMIN2	1.36250967	1.66E-117
GEMIN4	2.197769256	6.43E-228
GEMIN5	1.621445419	1.65E-179
GEMIN7	-1.83231965	2.14E-198
GEMIN8	1.339916801	2.13E-129
GEN1	1.220929756	6.88E-284
GET4	-1.025790632	5.14E-72
GFAP	1.656131872	4.01E-42
GFER	-0.016528195	0.811556153
GFI1	0.387545117	2.88E-153
GFI1B	0.225060942	1.65E-155
GFM1	0.044558579	0.566036325
GFM2	0.53593275	5.64E-16
GFOD1	-1.422131652	2.67E-45
GFOD2	0.488687293	1.52E-06
GFPT1	1.374356405	1.54E-101
GFPT2	1.777517103	1.65E-80
GFRA1	-1.083098319	2.42E-30
GFRA2	0.053985774	0.633275621
GFRA3	-0.033104541	0.760765189
GFRA4	-0.191126012	5.42E-16
GFRAL	-0.02223575	3.90E-06
GFY	-0.152008056	9.34E-05
GGA1	0.153136533	0.030553521
GGA2	0.523174488	5.41E-08
GGA3	0.609692915	2.57E-13
GGACT	1.145113557	1.89E-200
GGCT	0.65656316	8.97E-24
GGCX	1.64228965	1.69E-133
GGH	0.733924567	6.20E-17

GGN	0.796471209	1.08E-89
GGNBP2	0.00238121	0.975699922
GGPS1	0.594938308	1.24E-31
GGT1	-1.892161419	9.11E-188
GGT2	-0.045209516	1.48E-06
GGT5	1.097782147	1.98E-51
GGT6	-0.004677216	0.557737213
GGT7	0.747319859	2.86E-12
GGTLC1	0.008453781	0.025009767
GGTLC2	0.026524747	8.55E-06
GGTLC3	-0.03235869	0.049333939
GH1	-1.109371967	7.01E-20
GH2	-0.008043381	0.12338591
GHDC	1.496184204	3.87E-112
GHITM	0.050321691	0.484076698
GHR	0.699338579	2.35E-84
GHRH	-0.052249271	0.551863814
GHRHR	0.38383536	2.24E-94
GHRL	0.577647023	1.92E-82
GHSR	-0.080504959	0.000856487
GID4	0.74165345	3.25E-45
GID8	1.744547024	3.07E-170
GIGYF1	0.57954514	3.44E-09
GIGYF2	0.515026168	6.30E-19
GIMAP1	0.986372102	2.80E-99
GIMAP4	2.568612802	4.59E-239
GIMAP5	-2.328609349	1.16E-270
GIMAP6	2.331215182	2.67E-286
GIMAP7	1.282114433	4.38E-72
GIMAP8	1.632129965	7.16E-180
GIMD1	0.025374465	6.58E-37
GIN1	0.671343833	7.31E-38
GINM1	1.408442585	1.39E-138
GINS3	1.403054044	1.40E-78
GIP	0.04811609	4.12E-29
GIPC1	0.611376942	3.75E-32
GIPC2	0.079049996	0.000513728
GIPR	-0.579494928	1.08E-17
GIT1	-0.448650393	1.12E-12
GIT2	-1.641769266	1.75E-73
GJA1	1.653270236	7.52E-60
GJA10	-0.000841572	0.739505781
GJA3	0.774566181	1.11E-222
GJA4	1.589708348	1.13E-102
GJA5	0.333594915	1.07E-12
GJA8	0.039632448	2.59E-18
GJA9	-0.492662364	1.20E-60
GJB1	-0.882986718	1.53E-10

GJB2	2.074472864	5.42E-180
GJB3	-0.179836884	0.001197298
GJB4	0.054499772	4.89E-17
GJB5	0.212160061	2.69E-05
GJB6	-3.282892269	2.96E-133
GJB7	-0.000387101	0.967635951
GJC1	2.168885622	2.44E-213
GJC2	-0.318398026	0.002136806
GJC3	0.458231621	5.51E-32
GJD2	-0.569895529	6.59E-25
GJD4	-0.111992166	7.15E-09
GJE1	0.004791063	0.000900934
GK	1.488104392	1.79E-179
GK2	0.00499533	0.13850319
GK3P	0.102842408	3.06E-71
GK5	0.05462817	0.513405352
GKAP1	0.375811992	1.53E-09
GKN1	-0.196611228	9.04E-09
GKN2	-0.022260929	0.000586991
GLA	1.21921412	3.77E-88
GLB1L	1.852882877	1.33E-265
GLB1L2	1.139775724	2.35E-78
GLB1L3	-1.008335041	1.52E-33
GLCCI1	1.136682112	1.51E-30
GLCE	0.503260347	7.94E-05
GLDC	2.107191771	4.90E-217
GLDN	0.873950709	4.07E-15
GLE1	1.828058711	1.32E-116
GLG1	1.00146469	8.84E-66
GLI1	0.46761618	8.91E-11
GLI2	1.758313399	1.00E-277
GLI3	1.38212786	1.15E-104
GLI4	-0.384258379	3.44E-13
GLIPR1	2.481492762	2.25E-264
GLIPR1L1	0.712789523	8.32E-101
GLIPR1L2	0.413669921	5.48E-17
GLIS1	-0.402305817	3.25E-08
GLIS2	1.29035307	1.74E-73
GLMN	0.789046193	1.86E-25
GLO1	1.863801847	5.25E-212
GLOD4	0.77662007	1.71E-32
GLOD5	0.046359043	7.58E-10
GLP1R	0.043176269	0.292167203
GLP2R	-0.119527445	0.016904371
GLRA1	-0.244226854	5.37E-10
GLRA2	-0.647509855	1.70E-08
GLRA3	-0.341952004	5.49E-06
GLRB	0.196645061	0.046434743

GLRX	1.250293526	6.91E-35
GLRX2	0.707796723	4.20E-28
GLRX3	1.475599152	5.04E-137
GLRX5	1.104437406	2.11E-93
GLS	-0.817821286	2.06E-14
GLS2	-2.307149749	3.74E-68
GLT1D1	-1.906544996	2.55E-69
GLT6D1	-0.021656985	0.029460479
GLT8D1	1.872379769	5.97E-207
GLT8D2	0.48432278	9.20E-16
GLTP	1.468781531	5.57E-69
GLTPD2	0.349090041	7.00E-09
GLUD1	0.170128624	0.008417703
GLUD2	1.412956231	5.48E-241
GLUL	0.346841045	2.25E-06
GLYAT	0.080180008	1.48E-61
GLYATL1	0.15189307	5.62E-30
GLYATL2	0.861099549	2.00E-93
GLYATL3	0.005972507	0.000494325
GLYCTK	0.637558647	1.42E-36
GLYR1	0.832963216	1.31E-37
GM2A	2.203130129	8.36E-243
GMCL1	1.150364647	8.86E-93
GMDS	1.317064845	3.77E-165
GMEB1	1.361303531	1.45E-105
GMEB2	1.070547931	1.41E-57
GMFB	0.969040244	9.88E-62
GMIP	2.681223092	4.65758293001153e-316
GML	-0.029758484	0.000122869
GMNC	0.146356699	2.94E-06
GMNN	1.949155369	3.34E-154
GMPPA	1.123726192	8.87E-74
GMPPB	0.447476381	2.73E-16
GMPR	1.301070851	1.55E-37
GMPR2	1.18324741	1.02E-88
GMPS	1.646655051	3.33E-113
GNA11	-0.08934664	0.301202176
GNA12	2.067397958	2.75E-291
GNA13	2.034633344	8.11E-219
GNA14	0.044635761	0.657250453
GNA15	1.818935795	2.05E-122
GNAI1	-2.33522708	1.03E-149
GNAL	-2.085238093	3.74E-76
GNAO1	-1.33986446	1.96E-92
GNAQ	0.586865932	2.46E-27
GNAS	0.861855841	1.89E-25
GNAT1	0.055946375	7.35E-09
GNAT3	0.004992582	0.0187232

GNAZ	-0.316491507	1.09E-05
GNB1	0.530318625	1.16E-14
GNB1L	0.927084704	2.13E-125
GNB2	2.181069138	1.73E-284
GNB3	-1.04848545	1.07E-16
GNB5	-0.843785551	7.20E-18
GNE	1.071959281	4.62E-49
GNG11	1.808503923	1.44E-214
GNG13	-1.645237116	3.14E-17
GNG2	1.851288394	7.20E-48
GNG3	-4.406913218	5.12E-217
GNG4	0.941388063	7.52E-23
GNG5	-0.296310871	1.65E-06
GNG7	-0.873658715	7.67E-17
GNG8	0.571149193	6.60E-13
GNGT1	0.06154937	1.35E-14
GNL1	0.428100581	3.37E-11
GNL2	1.921313585	5.79E-257
GNL3	1.616765733	8.15E-107
GNLY	0.763854493	1.66E-29
GNMT	0.60515811	9.65E-28
GNPAT	1.783769854	4.12E-206
GNPDA1	2.262238018	5.42E-259
GNPDA2	0.880607777	1.63E-42
GNPNAT1	1.089779522	3.98E-66
GNPTAB	0.55980608	5.76E-26
GNPTG	0.063026029	0.205500184
GNRH1	-0.712084111	5.95E-15
GNRH2	0.341667143	3.56E-08
GNRHR	-0.021033479	0.29197637
GOLGA1	0.927430355	3.66E-69
GOLGA2	0.169243115	0.013140226
GOLGA3	0.629487619	9.79E-22
GOLGA4	0.372863431	2.47E-08
GOLGA5	1.494219252	1.07E-170
GOLGA6A	-0.002996777	0.692471339
GOLGA6B	0.000199654	0.983589583
GOLGA6C	0.007122103	0.005196192
GOLGA6D	-0.001774811	0.31943551
GOLGA6L1	-0.015375072	2.79E-06
GOLGA6L10	0.073853409	0.017891775
GOLGA6L2	0.131690436	1.46E-69
GOLGA6L22	-0.012811821	1.96E-05
GOLGA6L4	-0.073271147	7.57E-08
GOLGA6L6	-0.006371734	0.004038237
GOLGA6L9	0.108428599	0.091045304
GOLGA7	-0.503335385	3.26E-20
GOLGA7B	-2.159197933	6.77E-78

GOLGA8A	-1.575444663	2.67E-35
GOLGA8B	-1.397389992	2.86E-31
GOLGA8DP	0.003846808	0.012969101
GOLGA8F	-0.00114803	0.56166535
GOLGA8G	0.006156593	8.33E-14
GOLGA8H	0.295979075	3.80E-85
GOLGA8J	-0.016809773	0.000100051
GOLGA8K	-0.049095124	1.12E-07
GOLGA8M	0.064570441	0.001511405
GOLGA8N	-0.415099234	4.42E-29
GOLGA8O	-0.04938964	0.003009264
GOLGA8Q	-0.003835465	0.685514867
GOLGA8R	0.065867483	0.000191824
GOLGA8S	0.106365764	1.47E-77
GOLGA8T	-0.006405326	0.503554333
GOLGB1	0.591323053	2.56E-17
GOLIM4	2.447477331	1.98E-247
GOLM1	1.939819872	6.38E-175
GOLPH3	2.098213001	2.21E-295
GOLPH3L	1.855654546	1.37E-207
GOLT1A	-1.04933302	1.34E-20
GOLT1B	1.518932318	2.48E-81
GON4L	0.997095311	4.19E-52
GON7	2.170532116	1.61E-291
GOPC	0.660859273	1.14E-21
GORAB	1.816759901	5.71E-215
GORASP1	0.94258307	4.16E-48
GORASP2	1.690966991	6.47E-161
GOSR1	1.137589074	1.61E-76
GOSR2	0.934457319	1.72E-87
GOT1	-0.583600797	7.80E-08
GOT1L1	-0.003598626	0.099907252
GOT2	0.541168843	8.54E-11
GP1BA	0.18958034	1.32E-06
GP2	-0.48249942	4.30E-15
GP5	0.065629264	1.74E-10
GP6	-0.755004639	1.03E-33
GP9	0.138003772	3.81E-34
GPA33	0.279286037	3.59E-69
GPAA1	1.471489197	4.58E-114
GPALPP1	0.855716366	2.74E-42
GPAM	0.37340289	3.05E-10
GPANK1	1.795198063	3.45E-228
GPAT2	-0.60443779	9.25E-30
GPAT3	-0.08531893	0.332711404
GPAT4	0.723602123	4.82E-14
GPATCH1	0.725238027	8.48E-28
GPATCH11	1.451903762	1.11E-126

GPATCH2	1.371811	3.31E-252
GPATCH2L	0.484311381	7.80E-15
GPATCH3	1.342535294	4.85E-117
GPATCH4	0.492023075	2.52E-14
GPATCH8	0.625051956	6.40E-08
GPBP1	0.561840003	6.97E-18
GPBP1L1	1.077427012	1.57E-70
GPC1	1.725711012	1.80E-89
GPC3	1.360753816	1.51E-109
GPC5	0.008142617	0.935221627
GPCPD1	0.392410557	1.63E-06
GPD1	1.180737698	1.14E-22
GPD1L	0.511531323	7.58E-14
GPD2	1.056274907	1.98E-64
GPER1	0.813620679	5.66E-45
GPHA2	-0.256101511	2.01E-12
GPHB5	-0.020998973	0.102639614
GPHN	0.283064084	0.001084615
GPI	-0.407011527	3.35E-06
GPIHBP1	-0.468504704	5.63E-05
GPKOW	1.776437074	1.78E-175
GPLD1	-0.212111597	0.049188072
GPM6A	1.083522141	1.11E-27
GPM6B	1.624361572	9.64E-102
GPN1	1.440549433	4.50E-101
GPN2	1.116111103	4.42E-59
GPN3	1.633099485	1.30E-137
GPNMB	3.312076613	1.41E-216
GPR1	1.020592457	2.22E-107
GPR101	-0.424577824	9.37E-08
GPR107	1.45857358	7.04E-116
GPR108	1.643910741	6.98E-241
GPR119	0.009892233	1.37E-07
GPR12	-1.19156976	1.05E-25
GPR132	1.298526625	4.55E-279
GPR135	0.421883808	2.92E-16
GPR137	0.856017195	1.37E-56
GPR137B	1.725633558	5.64E-165
GPR137C	-0.612947503	7.65E-08
GPR139	0.461165496	3.73E-37
GPR141	0.749022255	3.30E-232
GPR142	-0.118608865	2.42E-05
GPR143	-0.838260088	1.38E-39
GPR146	-0.341496686	1.49E-11
GPR148	-0.271064713	2.92E-19
GPR149	-0.480765468	2.62E-14
GPR15	0.057415009	1.04E-07
GPR150	-0.212229123	0.000835862

GPR151	0.147053717	1.80E-10
GPR152	0.022547004	4.10E-05
GPR153	1.136552268	6.52E-40
GPR155	0.285867833	0.000194735
GPR158	-0.558034067	1.29E-05
GPR160	1.501230205	1.05E-280
GPR161	1.649526341	1.18E-234
GPR162	0.05534285	0.663937236
GPR17	0.143120961	0.121807954
GPR171	0.44250209	1.74E-124
GPR173	1.108900807	2.69E-51
GPR174	0.320293851	1.45E-83
GPR176	0.373806076	0.000299814
GPR179	-0.446515284	1.15E-14
GPR18	0.377308935	2.38E-188
GPR180	0.74663742	7.90E-33
GPR182	-0.044353798	8.31E-07
GPR183	2.89561353	7.56E-122
GPR19	1.022571721	1.45E-63
GPR20	0.034648383	0.104016199
GPR22	-1.4036974	1.11E-36
GPR25	-0.030457853	0.032758187
GPR26	-0.44217752	3.16E-10
GPR27	-2.088491518	4.73E-167
GPR3	1.418094106	5.08E-66
GPR32	0.018564506	0.000224485
GPR33	0.017882462	2.94E-23
GPR34	4.324490841	1.46487499598055e-318
GPR35	0.248427794	8.17E-07
GPR37	0.729636034	1.32E-08
GPR37L1	1.203923051	2.86E-65
GPR39	0.553179039	4.30E-110
GPR4	1.26248879	3.74E-45
GPR42	-0.002536055	0.61456292
GPR45	0.344745663	2.83E-14
GPR50	-0.011751599	0.742604764
GPR52	-0.717729046	9.94E-17
GPR55	0.063198805	0.261994273
GPR6	-1.558326711	6.66E-24
GPR61	-1.183408566	1.52E-54
GPR62	-0.759030455	8.03E-17
GPR63	-0.111183964	0.047350012
GPR68	-0.418026445	8.53E-06
GPR75	1.548526266	7.25E-115
GPR78	-0.079763947	4.25E-07
GPR83	-1.575758861	2.44E-37
GPR84	2.102138373	8.76E-302
GPR85	-0.024146361	0.673939085

GPR87	-0.063035998	1.43E-09
GPR88	-0.453648046	0.018460001
GPR89A	0.508275889	2.31E-22
GPR89B	-0.090122673	0.130116955
GPRASP1	-1.406951835	1.83E-33
GPRASP2	-0.053643395	0.612257954
GPRC5A	1.757569018	1.52E-223
GPRC5B	0.272550894	0.000100406
GPRC5C	0.009562075	0.9399706
GPRC5D	0.109406084	2.86E-13
GPRC6A	-0.000477032	0.750501121
GPRIN1	0.484743319	0.000201889
GPRIN2	-0.998605105	2.29E-20
GPRIN3	-0.078216555	0.496096847
GPS1	0.661486061	1.39E-23
GPSM1	0.884319283	4.58E-18
GPSM2	-0.20426277	0.000169386
GPT	-0.663889583	2.98E-21
GPT2	0.577972256	1.28E-21
GPX2	-0.26240857	1.09E-09
GPX3	1.545054037	2.47E-57
GPX4	1.129498283	1.82E-100
GPX5	-0.002789153	0.276760514
GPX6	-0.000786517	0.736117591
GRAMD1A	1.038791078	6.40E-56
GRAMD1B	-1.380997916	4.54E-27
GRAMD1C	0.034313004	0.606180829
GRAMD4	1.170307831	4.18E-85
GRAP	0.73739107	4.31E-164
GRAP2	0.207103919	6.91E-16
GRAPL	0.086339022	3.80E-152
GRB10	0.727571939	5.45E-20
GRB14	0.95317756	6.40E-40
GRB2	1.39213017	2.04E-94
GRB7	-0.697781861	6.65E-09
GREB1	-0.180965452	0.000705888
GREB1L	-0.222097752	3.81E-07
GREM1	-0.686066463	1.80E-24
GREM2	-0.79937264	1.87E-19
GRHL1	-0.571026407	5.96E-19
GRHL2	0.005837482	0.501418806
GRHL3	1.460718425	4.70E-178
GRHPR	1.109347018	3.68E-104
GRIA1	0.026176104	0.844332942
GRIA2	-1.015364157	5.34E-25
GRIA3	0.811193841	2.75E-16
GRIA4	0.34643034	0.002378788
GRID1	0.023001424	0.721093228

GRID2	0.568867136	3.71E-11
GRID2IP	0.262374587	1.09E-05
GRIFIN	0.001403258	0.948754959
GRIK1	1.01750375	1.09E-36
GRIK2	-0.425465038	0.001318057
GRIK3	2.605066582	9.41E-221
GRIK4	1.150161253	2.34E-87
GRIK5	1.135511783	1.71E-36
GRIN1	-4.695684985	3.02E-213
GRIN2A	-1.490919546	2.44E-41
GRIN2B	-1.174396811	2.44E-35
GRIN2C	-2.898853009	3.40E-81
GRIN2D	0.877018588	7.70E-43
GRIN3A	-0.222717843	0.001648495
GRIN3B	0.007775669	0.885926358
GRINA	1.030226533	7.84E-44
GRIP1	-0.487779334	1.38E-16
GRIP2	-1.182926499	1.10E-54
GRIPAP1	-0.943594194	1.73E-25
GRK1	0.242229284	5.46E-118
GRK2	-0.561069815	4.12E-10
GRK3	-0.217916023	0.005382746
GRK4	0.613514995	6.55E-20
GRK5	-0.6629139	9.99E-27
GRK6	1.201498054	7.27E-97
GRK7	0.044369967	7.79E-05
GRM1	-1.470753399	8.57E-31
GRM2	-1.052135462	3.86E-23
GRM3	-1.887898625	6.18E-113
GRM4	-2.578667869	5.85E-32
GRM5	-0.562349686	1.86E-17
GRM6	0.225852241	3.88E-17
GRM7	-0.906607649	4.40E-38
GRM8	0.488465357	1.64E-23
GRP	-0.336842104	0.00010711
GRPEL1	1.077168959	2.94E-71
GRPEL2	1.721343176	3.23E-132
GRPR	0.450509674	1.02E-205
GRSF1	1.326598698	3.25E-103
GRTP1	0.694895822	4.38E-74
GRWD1	1.984581726	5.74E-228
GRXCR1	-0.026140804	0.000358407
GRXCR2	-0.010509842	2.31E-05
GSAP	0.813626057	9.17E-47
GSC2	0.021232113	0.002754515
GSDMB	-1.601900983	1.81E-56
GSDMC	0.536463193	6.12E-77
GSDMD	1.865230163	1.28E-220

GSE1	0.643966482	1.90E-10
GSG1	-0.095936227	0.001660715
GSG1L	0.810364162	6.21E-22
GSG1L2	-0.013642441	0.269640984
GSK3A	-0.560380284	7.18E-14
GSK3B	0.034396919	0.663858446
GSKIP	0.788362772	1.09E-30
GSN	0.32377439	0.000115951
GSPT1	1.770419632	1.35E-213
GSPT2	2.016129502	2.45E-160
GSR	1.845622016	1.02E-155
GSS	0.328665737	7.71E-08
GSTA1	0.02156814	0.556415658
GSTA2	0.01265058	0.095977763
GSTA3	-0.04178556	0.033752114
GSTA4	1.096955335	3.28E-39
GSTA5	-0.001807729	0.366992498
GSTCD	1.819326972	6.18E-261
GSTM1	-0.014885582	0.952724542
GSTM2	0.587070769	1.81E-28
GSTM3	0.522570167	4.69E-14
GSTM4	1.086809279	9.05E-74
GSTM5	-1.19301357	1.28E-35
GSTO1	1.63605416	4.41E-147
GSTO2	-1.153226756	2.69E-86
GSTP1	1.589345381	3.56E-222
GSTT2	1.230286037	2.14E-252
GSTT2B	2.032531903	5.38E-113
GSTZ1	0.318537291	1.83E-08
GTDC1	0.275080305	9.24E-07
GTF2A1	1.045471665	9.79E-52
GTF2A1L	-0.209652735	9.12E-15
GTF2A2	1.381140985	1.41E-149
GTF2B	1.861079056	1.30E-198
GTF2E1	1.722537	1.80E-271
GTF2E2	1.522285199	4.73E-138
GTF2F1	1.397125928	2.89E-65
GTF2F2	1.620196729	6.39E-230
GTF2H1	1.160751962	4.70E-65
GTF2H2	0.840117648	1.77E-78
GTF2H3	1.50873793	3.40E-124
GTF2H4	-3.649733423	4.15E-298
GTF2H5	1.059695156	1.48E-96
GTF2I	0.598208832	4.37E-17
GTF2IRD1	1.765371088	5.12E-206
GTF2IRD2	0.767336843	2.57E-74
GTF2IRD2B	1.122604367	5.58E-130
GTF3A	1.590445396	4.57E-151

GTF3C1	0.802242133	1.27E-30
GTF3C2	1.256018043	6.74E-69
GTF3C3	1.433017658	1.72E-133
GTF3C4	1.652198297	1.84E-101
GTF3C5	1.234367041	1.04E-56
GTPBP1	0.529689446	2.84E-16
GTPBP10	1.305999913	9.84E-139
GTPBP2	1.519307834	1.16E-71
GTPBP3	0.83989418	7.84E-39
GTPBP4	0.980702966	6.20E-71
GTPBP6	0.709738228	1.64E-33
GTPBP8	1.497738437	1.36E-214
GTSF1	0.643123018	2.03E-143
GTSF1L	0.045651927	1.05E-09
GUCA1A	-1.773516892	1.66E-54
GUCA1B	0.149408334	0.041280257
GUCA1C	-0.117295817	2.43E-05
GUCA2A	0.3442212	9.74E-61
GUCA2B	-0.011211362	0.076333808
GUCD1	1.690961736	2.07E-265
GUCY1A2	0.525656486	3.94E-17
GUCY2C	0.02094114	0.038392008
GUCY2D	0.075242765	2.98E-11
GUCY2F	0.071105475	1.50E-90
GUF1	1.051118825	8.68E-74
GUK1	0.858467235	1.81E-61
GULP1	0.783346462	4.30E-46
GUSB	2.258870579	3.56E-193
GXYLT1	1.147214157	1.40E-134
GXYLT2	1.433758444	3.07E-198
GYG1	0.747254918	4.91E-36
GYG2	1.961667811	4.14E-102
GYPA	-0.088067344	3.53E-22
GYPB	-0.029404568	0.003297506
GYPE	0.111127882	1.72E-06
GYS1	1.919212222	9.50E-143
GYS2	-0.253354163	4.91E-50
GZF1	1.051914064	1.27E-84
GZMA	1.985607389	3.23E-298
GZMB	0.987760047	1.02E-108
GZMH	1.303326342	5.72E-173
GZMK	0.457453387	2.37E-57
GZMM	0.471542478	2.22E-09
H6PD	1.889105548	2.41E-205
HAAO	0.902987889	1.24E-67
HABP2	0.524914999	1.15E-92
HABP4	-0.847758182	2.21E-37
HACD1	-0.17878992	0.004361734

HACD2	1.286124106	1.56E-74
HACD3	1.418952795	3.17E-91
HACD4	0.995592126	1.25E-155
HACE1	-0.447045742	2.87E-09
HACL1	1.029441282	2.63E-43
HADH	0.992304016	2.40E-97
HADHA	1.459519249	7.99E-184
HADHB	1.171660209	3.73E-104
HAGH	-0.080239768	0.229212166
HAGHL	-1.275344555	2.72E-81
HAL	0.198359225	3.72E-77
HAMP	1.648323941	5.13E-35
HAND1	0.037736723	0.000228493
HAND2	1.150109725	2.71E-181
HAO1	0.125007845	1.00E-67
HAO2	0.143491466	1.35E-121
HAP1	0.975288225	1.74E-31
HAPLN2	-1.978820364	2.03E-55
HAPLN3	1.8556399	2.80E-217
HAPLN4	-3.248741779	9.35E-94
HARS2	0.21645473	0.003210887
HAS1	-0.957413105	2.90E-40
HAS3	1.088896598	2.98E-100
HAT1	1.454538247	1.75E-168
HAUS2	1.769588049	5.23E-190
HAUS3	0.414501312	1.74E-11
HAUS4	-0.32089368	6.61E-09
HAUS5	1.264789131	5.33E-45
HAUS6	1.493913319	8.18E-161
HAUS7	-0.111593882	0.011049585
HAVCR1	0.02198951	0.091820738
HAVCR2	3.45235908	4.52603732434293e-315
HAX1	1.260750106	5.72E-112
HBA1	0.674075469	3.16E-11
HBA2	0.714524411	5.30E-10
HBB	1.035548127	7.91E-17
HBD	-1.32486966	3.57E-19
HBE1	-0.372726028	3.97E-31
HBEGF	1.306439526	9.30E-65
HBG1	0.053332489	0.036315027
HBG2	-0.057823473	0.175423914
HBM	-0.151010954	2.67E-05
HBP1	1.428124303	8.44E-121
HBQ1	-1.154643555	1.27E-39
HBS1L	0.63173929	4.68E-26
HBZ	-0.027135368	0.252840615
HCAR1	0.676141886	3.88E-307
HCAR2	0.592488793	1.10E-109

HCAR3	0.140360982	9.95E-16
HCCS	1.838187261	2.86E-137
HCFC1	1.597481021	9.51E-96
HCFC1R1	-0.596694789	3.94E-29
HCFC2	1.612907495	7.51E-214
HCK	2.889210932	1.22E-221
HCLS1	3.390719282	1.33E-229
HCN1	-1.266789523	6.25E-42
HCN2	-1.741166806	2.67E-96
HCN3	-0.313899036	0.009229444
HCN4	-0.056400247	0.198736289
HCRT	-0.213875922	0.128519616
HCRTR1	-0.049776879	0.140903335
HCRTR2	-0.248244491	1.44E-14
HCST	0.901675984	4.68E-31
HDAC1	2.0732465	2.23E-244
HDAC10	-1.578174467	2.50E-96
HDAC11	-1.087113215	4.44E-92
HDAC2	1.207833738	2.43E-84
HDAC3	0.718064748	4.37E-20
HDAC4	0.227516691	0.000126046
HDAC5	-0.168759971	0.023199034
HDAC6	-0.000865714	0.994127121
HDAC7	0.475574607	2.16E-16
HDAC8	-1.386775755	3.60E-143
HDAC9	1.228386119	4.76E-94
HDC	-0.07396597	0.533539488
HDDC2	-0.761399116	5.51E-69
HDDC3	-0.609633572	2.23E-36
HDGF	2.029474579	2.41E-226
HDGFL1	0.009459378	0.241305638
HDHD3	0.825743122	9.11E-35
HDHD5	1.442491386	2.96E-114
HDLBP	1.344540773	1.66E-118
HDX	0.815224808	1.60E-64
HEATR1	1.543190596	3.03E-150
HEATR3	1.049401602	3.56E-45
HEATR4	-0.228472946	1.95E-06
HEATR5A	1.050239257	1.45E-117
HEATR5B	0.583240219	3.64E-15
HEATR6	1.189488905	1.42E-59
HEATR9	0.011970481	0.165527514
HEBP1	1.033770413	1.89E-77
HEBP2	1.329634227	4.32E-102
HECA	0.549623933	2.01E-27
HECTD1	0.333725402	5.91E-06
HECTD2	0.418573001	8.08E-11
HECTD3	1.498819423	3.56E-96

HECTD4	-0.827609474	1.93E-14
HECW1	-0.862451643	6.31E-23
HECW2	-0.880984376	1.08E-36
HEG1	1.772047704	1.76E-167
HELB	0.315459109	1.63E-34
HELLS	1.155342992	1.01E-49
HELQ	1.402329341	7.38E-131
HELT	0.01443308	0.100637136
HELZ	0.774324838	8.05E-38
HELZ2	2.165708023	5.58E-291
HEMGN	0.062286579	5.89E-07
HEMK1	0.929574106	2.53E-111
HENMT1	-0.446192585	2.29E-10
HEPACAM	1.225101853	3.39E-43
HEPACAM2	0.169793662	6.43E-40
HEPH	0.5130173	1.33E-14
HEPHL1	0.144803679	1.77E-106
HERC1	-0.443160036	1.04E-07
HERC2	-0.092092924	0.210037712
HERC3	-3.177510833	2.01E-219
HERC4	-0.030116628	0.585468092
HERC5	2.166754375	3.72E-273
HERC6	1.345214016	2.29E-69
HERPUD1	1.114918845	3.41E-69
HERPUD2	1.596343174	1.53E-179
HES1	1.717232084	3.68E-129
HES2	0.281138338	1.23E-112
HES3	0.087991252	4.20E-25
HES4	1.29393196	7.63E-52
HES5	0.439925692	1.00E-05
HES6	2.884064997	6.19364763976529e-313
HES7	-0.166215687	0.051658586
HESX1	1.305591698	5.14E-230
HEXA	-0.395094615	8.63E-10
HEXB	1.984062719	1.97E-184
HEXIM1	0.766726358	1.94E-45
HEXIM2	0.371622483	1.60E-13
HEY1	1.816978425	2.00E-162
HEY2	1.526017981	1.02E-160
HEYL	1.745341829	6.66E-114
HFM1	0.035817628	0.634643392
HGD	0.603602104	5.42E-55
HGF	1.39002407	9.98E-140
HGFAC	0.234299866	0.005553943
HGH1	1.818738041	3.44E-257
HGS	-0.743611914	2.42E-24
HGSNAT	0.767504695	1.60E-25
HHAT	1.013015049	1.70E-119

HHATL	-2.910204365	2.27E-142
HHEX	1.644767064	1.24E-186
HHIP	-0.633249688	8.30E-16
HHIPL1	-0.389559028	7.36E-13
HHIPL2	0.301541565	8.32E-118
HHLA1	-0.007705737	0.066563278
HHLA2	-0.077159854	2.39E-10
HIBADH	1.824658397	7.42E-285
HIBCH	0.507079472	4.33E-32
HIC1	0.318582484	5.32E-11
HIC2	-0.042545477	0.512329107
HID1	-1.96488968	3.96E-60
HIF1A	2.660998947	3.39E-271
HIF1AN	0.539217551	3.72E-26
HIF3A	-0.547771494	3.14E-11
HIGD1A	-0.666883936	4.78E-28
HIGD1B	0.368377076	1.62E-11
HIGD1C	0.19653232	6.04E-15
HIGD2A	0.777035079	2.49E-70
HIGD2B	0.110721108	7.07E-75
HIKESHI	1.13857374	1.15E-100
HILPDA	2.692662908	4.25E-124
HINFP	0.497006414	2.01E-10
HINT1	-0.212199252	0.00228668
HINT3	0.282705137	1.99E-07
HIP1	1.948736453	6.60E-181
HIP1R	-0.893929495	3.85E-46
HIPK1	1.04613376	6.67E-44
HIPK2	-0.521629352	1.78E-09
HIPK3	1.041936273	3.72E-66
HIPK4	-1.777761959	1.26E-59
HIRA	-1.084424986	3.55E-90
HIRIP3	0.788083411	6.19E-42
HIVEP1	0.783581771	2.04E-40
HIVEP2	-0.343778544	0.000148333
HIVEP3	0.802915279	1.02E-28
HK1	0.417914522	1.35E-05
HK3	2.030670802	2.64E-307
HKDC1	0.858009331	9.38E-61
HLA-B	2.361361548	1.54E-219
HLA-DMA	3.265058949	1.16E-222
HLA-DMB	2.668861363	2.12E-116
HLA-DOB	0.151441285	0.016317419
HLA-DPA1	3.344225253	9.02E-165
HLA-DPB1	2.9814593	1.52E-182
HLA-DQA1	3.554099181	5.36E-235
HLA-DQA2	3.206366196	9.26E-243
HLA-DQB1	2.822780134	1.40E-128

HLA-DQB2	2.369312847	6.83E-277
HLA-DRA	5.544265329	3.18E-270
HLA-DRB1	4.653504064	1.05E-283
HLA-DRB5	4.30379208	2.85E-217
HLA-E	2.419826703	5.80E-229
HLA-F	1.193151163	1.67E-74
HLA-G	0.991919749	1.90E-163
HLCS	0.990520718	5.12E-59
HLF	-1.528206107	1.72E-55
HLTF	-0.154061502	0.054182181
HLX	0.267695838	1.94E-10
HMBOX1	1.086448552	3.59E-136
HMBS	1.767159626	4.73E-221
HMCES	0.939779096	1.89E-47
HMCN2	0.278220985	3.63E-37
HMG20A	0.655901046	1.16E-19
HMG20B	1.93334039	2.51E-221
HMGA1	2.3039676	6.97E-163
HMGA2	0.679563717	9.88E-207
HMGB1	1.624390834	2.08E-179
HMGB2	2.770957289	1.01725009271704e-312
HMGB3	2.475608345	2.10E-283
HMGB4	-0.017648719	0.000994639
HMGCL	1.649850052	1.32E-231
HMGCLL1	-1.38351714	2.62E-86
HMGCR	0.810994244	2.35E-22
HMGCS1	0.087269267	0.35287867
HMGCS2	-0.088634869	1.81E-10
HMGN1	1.330310417	2.70E-102
HMGN2	2.408893215	1.48E-249
HMGN3	0.552525241	3.84E-24
HMGN4	2.691553859	1.41E-268
HMGN5	0.419621717	2.87E-10
HMGXB3	1.18823687	3.35E-62
HMGXB4	1.360018455	4.07E-111
HMHB1	0.368512851	2.94E-214
HMOX1	4.581898965	2.76676761671098e-322
HMOX2	1.057450416	3.80E-48
HMSD	0.090914568	0.26508829
HMX1	0.573767891	6.35E-32
HMX2	0.023445227	0.54261429
HMX3	0.118931539	0.004062868
HNF1A	0.045252283	3.64E-27
HNF1B	-0.012560548	0.193821457
HNF4A	-0.069407735	4.19E-08
HNRNPA0	2.121250042	1.73E-157
HNRNPA1	1.007081621	2.73E-48
HNRNPA1L2	1.315017178	1.95E-86

HNRNPA2B1	-0.236124586	0.000726456
HNRNPA3	0.895070211	1.53E-38
HNRNPAB	2.35924034	1.34E-253
HNRNPC	1.98577546	1.41E-246
HNRNPCL3	0.126805869	1.42E-237
HNRNPCL4	0.069833686	1.13E-102
HNRNPD	0.835021848	8.29E-37
HNRNPDL	0.819133964	1.07E-27
HNRNPF	2.412297037	1.50E-283
HNRNPH1	1.250030445	1.19E-50
HNRNPH2	0.952593477	1.49E-46
HNRNPH3	0.243212097	0.000245664
HNRNPK	1.413956961	1.50E-106
HNRNPL	-0.049607142	0.478337516
HNRNPLL	1.744842894	4.95E-147
HNRNPM	0.889591081	1.69E-37
HNRNPR	1.632685519	7.68E-146
HNRNPU	0.444984764	1.54E-10
HNRNPUL1	1.848276405	1.10E-206
HNRNPUL2	-0.933072561	1.60E-40
HOGA1	-0.120395752	0.093952933
HOMER1	0.437779528	2.95E-06
HOMER2	0.095056483	0.085703986
HOMER3	0.075487555	0.450361027
HOMEZ	1.192764319	9.97E-188
HOOK1	-1.814265748	3.23E-66
HOOK2	0.292549319	0.000471347
HOOK3	0.87386273	3.82E-48
HOPX	0.891888768	8.10E-20
HORMAD1	0.040188966	3.01E-08
HORMAD2	0.269130011	1.80E-53
HOXA2	1.954341161	9.03E-157
HOXA3	1.765792236	3.41E-267
HOXA4	0.824383274	7.73E-18
HOXA5	3.469626563	4.37E-237
HOXA6	1.363883727	2.23E-281
HOXA7	1.681075348	1.35E-178
HOXA9	1.053053792	1.16E-276
HOXB1	0.028353196	1.44E-09
HOXB2	2.433437637	8.74E-146
HOXB3	2.033290493	1.50E-197
HOXB4	2.148844688	6.23E-238
HOXB5	0.675783628	6.84E-20
HOXB6	0.311907813	3.60E-07
HOXB7	2.946933811	1.34E-220
HOXB8	0.708021063	1.44E-12
HOXB9	0.699568354	2.80E-79
HOXC12	0.098405959	8.88E-32

HOXC5	0.11774496	0.001497693
HOXD1	-0.38625526	2.38E-07
HOXD3	1.693193957	6.57E-192
HOXD4	2.185543823	6.93322320809021e-320
HOXD8	2.004798438	5.14E-211
HP	1.831465173	5.72E-168
HP1BP3	0.988769564	1.31E-56
HPCA	-2.643199778	1.31E-46
HPCAL1	-0.371595626	0.006638526
HPCAL4	-2.467266289	8.36E-76
HPD	1.720668006	4.15E-308
HPDL	-0.17919076	0.01274721
HPF1	1.485154056	1.47E-129
HPGD	0.34046684	1.19E-28
HPGDS	2.229065217	4.73E-240
HPN	-1.009192691	1.38E-48
HPR	0.115728206	0.235261744
HPRT1	0.123041011	0.244433227
HPS1	0.491579885	2.07E-17
HPS3	1.439535822	5.84E-135
HPS4	-0.306003815	0.000722501
HPS5	1.659678288	6.46E-189
HPS6	1.831584677	1.24E-180
HPSE2	-1.83350156	2.68E-62
HPX	0.190466033	0.009874512
HR	-0.613357063	1.66E-07
HRAS	0.423275971	1.00E-16
HRC	0.196864349	1.86E-06
HRCT1	1.475897551	5.83E-128
HRG	-0.045127062	6.06E-07
HRH2	0.013879229	0.915330297
HRH3	-3.501528027	6.40E-138
HRH4	0.090451175	1.66E-62
HRK	0.556169628	1.27E-12
HRNR	-0.031192153	2.65E-06
HS1BP3	1.273092709	7.09E-232
HS2ST1	2.088340069	5.78E-275
HS3ST1	1.797868994	7.53E-55
HS3ST2	0.590837615	1.31E-05
HS3ST4	-1.860179684	1.29E-57
HS3ST5	-0.908835363	1.06E-25
HS3ST6	-0.044338071	4.09E-05
HS6ST1	1.209117786	1.97E-56
HS6ST2	0.606269871	2.07E-11
HS6ST3	-1.371276479	7.63E-37
HSBP1	2.153367961	7.29E-285
HSBP1L1	-0.79147101	9.53E-28
HSCB	1.142313459	1.49E-158

HSD11B1	-0.015972632	0.894967578
HSD11B1L	-0.649645288	3.92E-18
HSD11B2	0.375919343	0.000539937
HSD17B1	-0.275059118	1.62E-07
HSD17B11	1.770896661	6.10E-117
HSD17B12	1.014451912	8.29E-51
HSD17B13	-0.020128165	0.77324812
HSD17B14	0.985696846	2.66E-69
HSD17B2	0.01775807	3.55E-11
HSD17B3	-1.691811881	5.20E-108
HSD17B4	-0.397972605	3.67E-15
HSD17B6	-0.439343227	2.49E-09
HSD17B7	0.707822203	3.58E-31
HSD17B8	2.016673728	2.28E-266
HSD3B1	-0.018343571	1.58E-06
HSD3B2	-0.059557015	0.008397396
HSD3B7	0.900477233	1.35E-40
HSDL1	0.826888553	1.49E-29
HSDL2	1.630675589	2.48E-143
HSF1	0.172827537	0.003777947
HSF2	1.151791658	2.87E-55
HSF4	-2.168308406	8.20E-84
HSF5	-0.011202875	0.22686901
HSFX1	-0.069537174	0.000761394
HSFX2	-0.002658378	0.873950492
HSFY1	0.000144794	0.824748902
HSFY2	-0.056681026	7.43E-13
HSH2D	0.41087503	1.72E-67
HSP90AA1	0.41322958	3.56E-08
HSP90AB1	1.049796248	3.86E-38
HSP90B1	1.319146825	6.79E-80
HSPA12A	-2.759717453	2.77E-211
HSPA12B	0.60605779	1.13E-24
HSPA13	2.330172005	1.34E-205
HSPA14	1.071541795	3.74E-51
HSPA1A	0.002966578	0.985363578
HSPA1B	0.102904689	0.452205705
HSPA1L	0.700177613	5.78E-38
HSPA2	0.232196861	0.047428376
HSPA4	1.535027401	3.10E-111
HSPA4L	0.340496241	8.83E-05
HSPA5	2.789129843	2.42E-219
HSPA6	2.244898826	7.27E-38
HSPA8	1.597302052	1.79E-70
HSPA9	0.281763453	7.65E-07
HSPB1	2.202503086	1.77E-88
HSPB11	1.727817216	1.72E-245
HSPB2	-2.653349619	1.04E-247

HSPB3	0.208993299	0.082143608
HSPB6	0.73410319	2.66E-22
HSPB7	0.86678077	1.70E-19
HSPB8	0.555878372	4.02E-07
HSPB9	0.536480254	4.21E-99
HSPBAP1	1.254702317	2.96E-191
HSPBP1	1.077915514	1.67E-54
HSPD1	0.2775147	1.26E-05
HSPE1	-0.696299412	7.84E-30
HSPH1	-0.155909474	0.174879694
HTATIP2	0.901020908	1.05E-44
HTATSF1	0.773802733	9.70E-39
HTN1	0.00163782	0.654489951
HTN3	0.002964475	0.446352083
HTR1A	-0.393368597	6.17E-08
HTR1B	-0.510927876	1.73E-15
HTR1D	0.181659884	0.056800033
HTR1E	-1.164179455	1.64E-58
HTR1F	0.208260498	5.95E-43
HTR2A	-0.437224625	6.14E-05
HTR2B	0.299460698	6.39E-63
HTR2C	-1.685473529	1.20E-29
HTR3A	-0.515699788	1.28E-16
HTR3B	-0.22356119	4.88E-05
HTR3C	0.046114787	2.35E-38
HTR3D	0.004771535	0.029787602
HTR3E	-0.007462197	0.011646123
HTR4	-0.202819623	1.85E-05
HTR5A	-2.489851939	1.28E-76
HTR6	-0.509638921	5.47E-10
HTR7	0.279965526	1.03E-07
HTRA1	1.725593936	2.04E-114
HTRA2	1.175150066	1.52E-90
HTRA4	0.524061329	3.09E-125
HTT	0.016767082	0.843145058
HUNK	0.967897909	2.27E-46
HUS1	1.763847058	2.24E-253
HUS1B	0.061559141	0.006100255
HUWE1	0.72138138	9.08E-26
HVCN1	1.160996017	9.36E-77
HYAL1	-0.138477463	0.00182519
HYAL2	2.478301921	5.53353523342196e-322
HYAL3	0.586622985	5.59E-13
HYAL4	0.048165874	3.69E-31
HYDIN	0.196896367	5.83E-07
HYKK	0.245722987	1.03E-17
HYLS1	0.662424328	2.77E-22
HYOU1	1.132397331	1.35E-47

HYPK	-1.220213824	1.35E-45
IAH1	0.537364471	3.02E-23
IAPP	0.008640901	0.249696348
IARS2	1.581904822	7.42E-146
IBA57	0.493743879	1.70E-24
IBTK	0.364430165	1.17E-07
ICA1	-0.523124067	1.29E-07
ICA1L	-0.064300147	0.451235568
ICAM1	2.745772178	3.18E-170
ICAM2	-0.884690335	8.21E-37
ICAM3	0.191984377	6.36E-13
ICAM5	-1.016871292	1.25E-07
ICE1	0.926460849	3.83E-32
ICE2	0.746316746	1.69E-54
ICMT	1.374906403	1.59E-83
ICOS	0.312880796	1.66E-184
ICOSLG	0.357710491	1.38E-19
ID1	1.812872458	4.81E-138
ID2	2.792360347	8.88E-246
ID4	2.340260098	3.54E-174
IDE	0.265334954	3.94E-07
IDH2	1.920652645	3.42E-209
IDH3A	-0.235923338	0.000965722
IDH3B	1.125336972	4.25E-45
IDH3G	1.088050724	6.18E-46
IDI1	-0.304796959	4.01E-05
IDI2	-0.132546571	9.08E-09
IDNK	0.231540823	0.000613271
IDO1	0.91629882	7.38E-145
IDO2	0.027478904	4.03E-22
IDUA	0.391750551	0.000305389
IER2	0.889241494	8.05E-39
IER3	0.60348307	2.69E-11
IER3IP1	1.255177034	1.71E-79
IER5	2.531418615	1.45E-161
IER5L	1.352748756	6.36E-71
IFFO1	0.93301703	3.26E-29
IFFO2	0.675510776	1.04E-11
IFI16	3.035301376	9.21E-281
IFI27	0.263892977	6.68E-05
IFI27L1	1.652654675	7.40E-181
IFI27L2	0.429485692	1.98E-17
IFI30	-2.473399962	5.03E-102
IFI44	2.662735815	5.96E-255
IFI44L	1.991399208	2.72E-114
IFI6	3.011173854	7.13E-283
IFIH1	2.199772434	1.76E-295
IFIT1	1.053739752	6.75E-41

IFIT1B	0.066146034	7.53E-14
IFIT2	1.643257435	3.43E-101
IFIT3	2.306981094	1.37E-199
IFIT5	1.34023963	5.86E-129
IFITM1	0.642987308	1.18E-14
IFITM10	-1.544025124	2.04E-54
IFITM2	1.589563971	1.27E-62
IFITM3	2.322902269	3.01E-132
IFITM5	0.218183566	2.53E-56
IFNA1	-0.03211838	0.015284738
IFNA10	0.001582657	0.581753422
IFNA13	-0.120130263	1.64E-23
IFNA14	0.010894831	0.001658377
IFNA16	-0.002649401	0.267048574
IFNA17	8.49E-05	0.981129513
IFNA2	-0.150382765	3.24E-22
IFNA21	-0.259763116	8.08E-23
IFNA4	0.005208091	0.121578683
IFNA5	0.005863101	0.603031518
IFNA6	-0.226226606	2.04E-28
IFNA7	-0.00387772	0.211510536
IFNA8	-0.088208986	6.21E-07
IFNAR1	1.934902856	1.55E-270
IFNAR2	-1.579536327	3.35E-218
IFNB1	0.076430178	3.16E-24
IFNE	-0.208077577	8.08E-06
IFNG	0.10300585	4.50E-07
IFNGR1	1.979491628	4.75E-172
IFNK	-0.011135736	0.182697572
IFNL1	0.013801918	0.05287295
IFNL2	-0.003555071	0.485187701
IFNL3	-0.084208065	1.29E-07
IFNLR1	0.772089262	4.79E-16
IFNW1	-0.048295103	1.08E-07
IFRD1	-0.382877761	1.23E-12
IFRD2	1.641945395	2.14E-134
IFT122	0.305696476	4.16E-05
IFT140	0.75798888	3.04E-23
IFT172	-0.569165418	2.04E-14
IFT20	0.007978799	0.924534524
IFT22	0.987845015	7.96E-55
IFT27	-0.520570184	5.04E-26
IFT43	1.039898696	9.22E-106
IFT46	1.424557562	5.07E-92
IFT57	1.942721388	6.99E-220
IFT74	-0.080915963	0.096158626
IFT80	-0.619175227	7.83E-20
IFT81	1.313008504	1.56E-94

IFT88	1.016132152	1.94E-98
IGBP1	1.251890376	1.80E-80
IGDCC3	0.914934645	7.10E-34
IGDCC4	2.554660073	1.88259052537058e-313
IGF1	-0.031719151	0.236709108
IGF1R	0.36252781	4.08E-08
IGF2	-1.375243851	2.59E-83
IGF2BP1	0.276668564	1.26E-74
IGF2BP2	2.048229037	3.81E-105
IGF2R	1.245698143	2.68E-110
IGFALS	-0.436265099	9.91E-18
IGFBP4	3.253790839	4.44293036025217e-310
IGFBP6	0.7379143	2.07E-22
IGFBPL1	1.91695944	2.19E-132
IGFL1	-0.041458152	0.000777617
IGFL2	-0.022190605	0.372219902
IGFL3	0.606309678	2.27E-289
IGFL4	-0.273330414	4.36E-05
IGFLR1	-0.538615811	2.45E-20
IGFN1	-0.038141826	0.516540048
IGHMBP2	-0.239984446	0.006225938
IGIP	-1.006775501	2.72E-70
IGLL1	-0.069563023	0.000119721
IGLON5	-0.050443293	0.726073981
IGSF1	0.621833256	1.35E-11
IGSF10	0.032905369	0.283958994
IGSF11	1.791610455	1.62E-98
IGSF21	-0.363815866	0.006684236
IGSF22	0.379651087	5.05E-20
IGSF23	0.045483843	4.17E-12
IGSF3	2.848762849	1.28E-303
IGSF5	1.24928612	8.65E-138
IGSF8	-0.160483038	0.062805561
IGSF9	0.954667939	9.25E-219
IGSF9B	1.456079219	3.96E-44
IHH	-0.028062562	0.087920799
IK	0.486061491	7.97E-14
IKBKB	0.836236592	3.01E-33
IKBKE	1.621997753	9.28E-143
IKBKG	0.916067046	7.03E-63
IKZF1	1.608892892	7.95E-210
IKZF2	0.512177593	6.62E-44
IKZF3	0.355417183	2.40E-96
IKZF4	0.590003761	3.80E-19
IKZF5	0.296669184	4.32E-06
IL10	0.83939243	3.77E-152
IL10RA	1.854425385	1.53E-112
IL10RB	-1.566966921	8.13E-214

IL11	0.237238901	0.008350786
IL11RA	-0.210610689	0.015284738
IL12A	0.72809073	6.91E-61
IL12B	0.005016565	0.341382607
IL12RB1	1.337468219	2.61E-284
IL12RB2	-0.044761312	0.387013629
IL13	0.050469897	3.64E-05
IL13RA1	2.838225738	6.55E-234
IL13RA2	3.775801997	1.30E-241
IL15	0.561992886	9.72E-95
IL15RA	1.281648672	1.34E-130
IL16	0.166478755	0.321049085
IL17A	-0.000572721	0.701572158
IL17B	0.629888145	1.10E-101
IL17C	-0.054431702	0.028822739
IL17D	-0.78671219	2.71E-45
IL17F	-0.02722381	0.010956951
IL17RA	1.363902235	4.14E-92
IL17RB	0.47634694	6.33E-12
IL17RC	0.71925596	1.51E-50
IL17RE	-0.349042586	0.000855861
IL17REL	-0.069663077	0.000314966
IL18	2.530826564	5.31E-138
IL18BP	1.692480292	3.54E-100
IL18R1	-0.133387639	0.026059858
IL18RAP	0.251215012	1.46E-16
IL19	0.055699695	8.66E-87
IL1A	1.53006507	8.86E-280
IL1B	2.612078951	1.10E-148
IL1F10	0.000417578	0.804754464
IL1R1	0.992574805	5.18E-38
IL1R2	1.275391712	1.06E-123
IL1RAPL1	-0.083990759	0.020641142
IL1RAPL2	-0.00426128	0.846280087
IL1RL1	-0.709085965	2.34E-08
IL1RL2	-0.215134604	6.30E-07
IL1RN	1.893446734	5.22E-297
IL2	0.022798948	6.89E-06
IL20	0.037557538	8.16E-18
IL20RA	-0.246831757	5.40E-06
IL20RB	0.742209212	8.27E-116
IL21	0.007628946	1.12E-07
IL21R	0.919345145	2.45E-144
IL22	-0.007845832	0.575381075
IL22RA1	0.13637026	2.54E-06
IL22RA2	0.036492299	1.68E-57
IL23A	1.088089412	4.43E-287
IL23R	0.028594937	1.00E-62

IL24	0.171593494	2.75E-41
IL25	0.038569768	0.001312961
IL26	0.054928545	4.57E-08
IL27	0.480516189	2.32E-93
IL27RA	1.457404523	9.47E-149
IL2RG	0.307148414	1.44E-08
IL3	0.008239052	0.04231742
IL31	0.011721448	0.185333075
IL31RA	0.198683964	7.49E-52
IL32	2.221158972	3.65E-200
IL33	2.190997322	2.19E-159
IL34	-0.363281665	4.74E-06
IL36A	-0.005482299	0.471900123
IL36B	0.433501432	6.17E-260
IL36G	0.187899186	5.48E-119
IL36RN	0.068080906	1.75E-31
IL37	0.179032228	1.48E-89
IL3RA	1.227723299	3.03E-67
IL4	0.293634645	1.44E-132
IL4R	1.259757215	4.18E-41
IL5	0.041243715	0.001488138
IL5RA	-0.020183374	0.654325465
IL6	0.930888095	1.02E-21
IL6R	1.308577785	9.87E-96
IL6ST	1.257122599	9.70E-130
IL7	0.929471341	3.13E-129
IL9	0.092821861	1.59E-08
IL9R	0.060153396	4.30E-26
ILDR1	0.16285962	4.64E-158
ILDR2	1.979297684	8.16E-203
ILF3	1.376683503	6.98E-69
ILKAP	0.120457169	0.092244841
ILVBL	1.135095877	3.29E-90
IMMP1L	-0.341654405	1.17E-10
IMMT	1.634129864	2.21E-118
IMP3	0.112558807	0.069488209
IMP4	1.234248774	2.76E-115
IMPA1	1.288078007	4.20E-92
IMPA2	1.193802482	3.80E-87
IMPACT	1.650936168	6.02E-149
IMPDH1	1.056437014	3.57E-59
IMPDH2	0.838314986	2.40E-46
IMPG1	-0.480001309	1.23E-17
IMPG2	0.258416872	3.36E-79
INA	-2.975357081	3.02E-89
INAFM1	2.47382834	2.61E-229
INAFM2	1.524471219	5.42E-112
INCA1	1.602144067	8.67E-234

INCENP	1.633164991	8.37E-67
INF2	-0.269712226	0.006638526
ING1	1.532795808	1.38E-173
ING2	0.800271588	1.09E-44
ING3	1.923377047	4.03E-151
ING4	1.312059104	1.33E-76
ING5	0.38096143	3.18E-08
INHA	-0.627008327	1.10E-14
INHBA	0.648287908	1.05E-40
INHBB	2.00238769	1.99E-195
INHBC	0.420194311	1.94E-37
INHBE	0.000359358	0.995111274
INIP	1.588814883	1.47E-149
INMT	1.327475628	8.59E-213
INO80	0.829302687	7.39E-30
INO80C	0.502521374	1.36E-20
INO80D	0.115625025	0.079137804
INO80E	0.630629135	9.60E-27
INPP1	0.148061547	0.010920959
INPP4A	0.137740024	0.154064142
INPP4B	0.325392754	2.44E-17
INPP5A	-0.732146436	4.50E-12
INPP5B	0.782686507	2.50E-24
INPP5D	1.731654912	1.52E-105
INPP5E	0.675760466	2.76E-19
INPP5F	-1.589720969	1.18E-49
INPP5J	-1.450514591	6.04E-23
INPP5K	0.928293394	6.21E-68
INPPL1	1.868924985	1.15E-213
INS	-0.22781385	2.23E-11
INSC	0.09889373	3.80E-08
INSIG1	0.691208607	5.52E-14
INSIG2	1.561735311	1.34E-125
INS-IGF2	-0.27263955	9.97E-34
INSL3	-0.360122861	5.88E-16
INSL4	0.012392977	3.66E-14
INSL5	0.252308772	2.53E-18
INSL6	0.127168046	2.47E-174
INSM1	1.697232121	5.54E-44
INSM2	-0.446225959	1.81E-15
INSR	0.629605678	9.72E-22
INSRR	0.344304553	6.53E-12
INTS1	0.909142263	1.17E-28
INTS10	0.734484944	1.67E-30
INTS11	0.343913759	0.000106666
INTS12	1.495430796	1.10E-206
INTS13	1.860422066	1.78E-179
INTS14	1.852781027	2.57E-238

INTS2	0.596661895	2.17E-21
INTS3	0.565284615	9.87E-12
INTS4	1.444665869	3.35E-109
INTS5	2.271898577	1.77E-236
INTS6	0.645362049	1.03E-62
INTS6L	-0.802950141	9.62E-15
INTS7	2.002135765	4.84E-280
INTS8	0.446118796	1.06E-10
INTS9	1.823591839	3.16E-289
INTU	-0.120414181	0.029924877
INVS	1.082470001	4.13E-102
IP6K1	0.960598045	2.90E-35
IP6K2	1.558242376	3.05E-63
IP6K3	0.587227555	4.39E-10
IPCEF1	-1.472612764	1.16E-33
IPMK	0.708279141	6.26E-46
IPO11	1.503591266	3.64E-201
IPO13	0.758571459	9.08E-33
IPO4	-2.163934613	1.21E-222
IPO5	1.545352968	8.19E-128
IPO7	1.734000628	2.67E-161
IPO8	1.572164157	2.60E-181
IPO9	1.87226077	8.70E-162
IPPK	-0.63742993	4.98E-16
IQCA1	-1.549419458	7.54E-74
IQCA1L	0.130388847	9.16E-78
IQCB1	1.348959141	7.89E-95
IQCC	0.70549285	6.05E-22
IQCD	1.191921221	1.69E-123
IQCE	0.75827519	6.21E-56
IQCF1	-0.030605393	0.235386621
IQCF2	-0.000649631	0.901550157
IQCF3	-0.089676292	3.12E-11
IQCF5	-0.001930865	0.572269142
IQCF6	-0.007495417	0.165489737
IQCG	1.140960952	9.11E-84
IQCH	0.763685071	2.03E-186
IQCJ	-0.047382568	1.15E-17
IQCK	0.214376551	2.02E-05
IQGAP1	2.039315797	7.72E-207
IQSEC1	-0.742604839	4.50E-18
IQSEC2	-0.956400109	2.13E-21
IQSEC3	-3.161072558	1.39E-118
IQUB	0.432463516	1.34E-48
IRAK1BP1	1.426074469	2.17E-295
IRAK2	1.634012478	1.68E-117
IRAK3	0.875242334	4.41E-68
IREB2	1.476173404	1.12E-101

IRF1	1.554782869	3.85E-96
IRF2BP1	1.305893724	3.61E-61
IRF2BP2	1.283647343	3.59E-84
IRF2BPL	1.635799376	3.38E-94
IRF3	0.87726245	4.94E-54
IRF4	0.215399692	6.26E-34
IRF5	1.931200593	3.24E-177
IRF6	-0.291780212	0.01810719
IRF7	1.803504822	2.45E-121
IRF8	2.475659468	7.18E-223
IRGC	-0.055561677	0.00047088
IRGM	0.211402066	1.71E-217
IRGQ	0.661116043	2.52E-17
IRS1	0.894864697	1.02E-34
IRS2	1.56520688	1.04E-110
IRS4	-0.120618758	0.005976382
IRX1	1.410671166	2.98E-45
IRX2	-0.098967644	0.241008298
IRX3	0.786588606	3.15E-17
IRX4	0.064384359	1.09E-12
IRX5	1.225276698	2.14E-68
IRX6	-0.122302408	0.001347452
ISCA1	0.940000955	7.33E-46
ISCA2	1.306387512	8.35E-162
ISCU	-0.340340687	9.27E-07
ISG15	3.161870927	1.17E-212
ISG20	1.123112908	2.88E-68
ISG20L2	1.871938871	3.00E-219
ISL1	-0.456195346	1.32E-08
ISLR	2.207684642	1.78E-130
ISLR2	-0.530493478	1.18E-06
ISM1	0.147134587	0.003091095
ISM2	-0.168446334	0.001642815
ISOC1	1.669673607	5.88E-88
ISOC2	0.720966132	3.38E-55
IST1	1.251143592	2.29E-109
ISX	0.001696407	0.292281892
ISY1	0.298194534	1.78E-09
ISYNA1	1.132965712	8.23E-89
ITCH	0.858536966	2.27E-43
ITFG1	0.638240472	9.58E-14
ITFG2	0.397853173	1.86E-07
ITGA1	1.367169402	3.94E-143
ITGA10	0.251891821	0.000208942
ITGA11	1.294805749	5.75E-273
ITGA2	1.929210101	5.33E-154
ITGA2B	-0.554436937	2.90E-12
ITGA3	1.432213334	1.07E-46

ITGA4	1.63618433	4.20E-170
ITGA5	3.154825168	3.81364380774964e-317
ITGA6	1.83969951	3.85E-169
ITGA7	2.066579435	1.36E-185
ITGA8	0.268273281	4.11E-08
ITGA9	0.522091824	1.01E-25
ITGAD	0.214301862	1.68E-56
ITGAE	1.042563579	3.22E-105
ITGAL	1.336108306	2.47E-121
ITGAM	2.109289048	8.77E-161
ITGAV	2.425439995	7.08E-251
ITGAX	1.669544162	5.76E-65
ITGB1	1.209204486	3.65E-64
ITGB1BP1	0.20877876	0.001685236
ITGB1BP2	0.214296123	3.95E-08
ITGB2	3.766869076	8.66E-244
ITGB3	1.898183696	4.39224359152868e-321
ITGB3BP	1.97362167	7.06415060423814e-320
ITGB4	2.007622051	6.90E-94
ITGB5	1.846518008	6.65E-256
ITGB6	-0.05944213	1.50E-21
ITGB7	0.209499094	5.68E-12
ITGB8	2.434750767	3.11E-196
ITGBL1	0.430096155	2.32E-18
ITIH1	0.321855409	9.46E-116
ITIH2	0.46206278	2.36E-14
ITIH3	-0.027359649	0.416493564
ITIH4	-2.170180225	1.90E-205
ITIH5	0.050792161	0.410618468
ITIH6	0.029596246	7.50E-55
ITK	0.582166748	4.68E-117
ITLN1	-0.031414172	0.038049651
ITLN2	0.156695988	5.49E-39
ITM2A	1.666787391	6.58E-106
ITM2B	1.509635281	1.86E-105
ITM2C	1.935804196	1.77E-85
ITPA	2.270727369	3.68E-307
ITPK1	-0.298697398	3.35E-10
ITPKA	-0.738319471	2.59E-06
ITPKB	1.016006751	4.67E-45
ITPKC	2.086328951	9.34E-159
ITPR1	-2.063012202	2.00E-54
ITPR2	1.531848664	4.55E-158
ITPR3	-0.3134387	4.92E-08
ITPRIP	2.18146062	6.67E-304
ITPRIPL1	2.568521132	5.81E-265
ITSN1	-0.109354943	0.074756004
ITSN2	0.077526112	0.165893683

IVD	0.806732966	1.46E-51
IVL	-0.097652469	4.36E-11
IVNS1ABP	1.408321242	1.79E-91
IWS1	1.087498866	7.44E-79
IYD	-0.038699936	0.001194357
IZUMO1	-0.19271991	0.000163846
IZUMO1R	0.032933869	1.88E-07
IZUMO2	0.003946186	0.638309229
IZUMO3	0.024649877	5.92E-06
IZUMO4	-0.906264427	2.29E-64
JADE1	0.79140388	4.20E-27
JADE2	-0.301100073	0.000116181
JADE3	1.561126769	3.58E-158
JAG2	-0.910611013	8.39E-35
JAK1	0.548988526	7.83E-16
JAK2	0.902306917	7.99E-50
JAK3	0.392130283	2.09E-08
JAKMIP1	-3.955030455	1.39E-230
JAKMIP2	0.742184218	2.90E-26
JAKMIP3	-2.36048897	2.59E-166
JAM3	0.162550678	0.028949137
JAML	0.013880725	0.898960936
JARID2	0.462560975	2.43E-07
JAZF1	0.968686071	1.46E-25
JCHAIN	1.554958508	4.22E-160
JDP2	0.836578377	6.41E-53
JKAMP	0.824747041	7.47E-33
JMJD1C	0.442602107	9.81E-06
JMJD4	-0.407321465	1.62E-15
JMJD6	0.775352799	2.47E-23
JMJD7	-3.863233686	6.43E-239
JMJD8	0.183284618	0.005026898
JMY	0.371277277	2.28E-09
JOSD1	0.941388188	5.36E-41
JOSD2	0.794591046	6.31E-51
JPH1	-1.363340607	1.91E-33
JPH2	1.001360511	2.83E-138
JPH3	-3.806365587	1.36E-214
JPH4	-2.478173485	1.86E-73
JRK	0.579932516	6.51E-16
JRKL	1.193529244	1.62E-121
JSRP1	-0.157517989	6.06E-10
JTB	-1.771538681	5.17E-193
JUN	2.231303041	4.04E-127
JUNB	1.652299871	1.33E-66
JUND	0.471548942	1.58E-13
JUP	0.913428101	3.31E-33
KAAG1	0.045999709	6.54E-07

KALRN	-0.227614136	0.028348178
KANK1	0.578684266	1.33E-24
KANK2	1.964811337	9.11E-281
KANK3	-0.495630642	1.13E-21
KANK4	-0.237245569	0.001156651
KANSL1	0.035798157	0.66874709
KANSL1L	0.447603976	2.95E-14
KANSL2	2.103917303	2.60E-150
KANSL3	0.600635751	1.46E-21
KAT14	0.627936562	1.13E-25
KAT2A	-0.060317888	0.470354138
KAT2B	0.880779646	1.51E-72
KAT5	-0.399753416	8.14E-09
KAT6A	0.137698499	0.043474388
KAT6B	-0.758221617	4.07E-46
KAT7	1.40594325	5.28E-75
KAT8	-0.119514097	0.135167687
KATNA1	1.769384658	7.55E-213
KATNAL1	0.863543239	5.45E-49
KATNAL2	0.379574592	6.92E-21
KATNB1	0.277252591	0.000702184
KATNBL1	0.851369525	1.89E-43
KAZALD1	1.172475561	1.51E-163
KAZN	-0.806714576	2.51E-15
KBTBD11	-0.631327808	5.03E-21
KBTBD11-OT1	-0.498102504	1.50E-23
KBTBD12	-0.261439993	6.47E-06
KBTBD13	0.067944831	8.41E-41
KBTBD2	1.783529671	7.75E-186
KBTBD3	0.779365219	1.42E-50
KBTBD4	0.459532346	2.31E-10
KBTBD6	0.762431083	1.37E-26
KBTBD7	0.76862644	4.40E-34
KBTBD8	1.180217731	1.55E-149
KCMF1	1.280584624	4.40E-104
KCNA1	-2.005644943	2.97E-44
KCNA10	-0.000564142	0.901494911
KCNA2	-0.887796859	2.01E-15
KCNA3	0.181352485	0.000160259
KCNA4	-1.039716281	5.51E-26
KCNA5	-0.85579554	3.68E-24
KCNA7	0.011059724	0.023734057
KCNAB1	-1.667706192	1.71E-46
KCNAB2	-2.477455306	1.00E-99
KCNAB3	-0.066870996	0.645185191
KCNB1	-1.094018148	7.44E-30
KCNB2	-0.52784989	7.07E-16
KCNC1	-1.324881581	1.49E-17

KCNC2	-1.371460777	1.26E-19
KCNC3	-0.955160481	3.58E-13
KCNC4	-0.889400932	2.77E-31
KCND1	1.029489392	1.51E-27
KCND2	0.558041024	1.23E-05
KCND3	0.036036569	0.753463269
KCNE1	0.167338612	2.20E-45
KCNE1B	0.062590953	4.93E-14
KCNE2	-0.666117395	1.62E-89
KCNE4	2.705581232	1.06E-240
KCNE5	0.715246081	2.31E-15
KCNF1	1.5365027	6.56E-26
KCNG1	0.082547141	0.331671983
KCNG2	0.091564486	0.093107737
KCNG3	-0.386104379	6.26E-16
KCNG4	0.046000738	0.018332323
KCNH1	-0.839235331	5.25E-22
KCNH2	0.885009271	1.01E-14
KCNH3	-2.075901114	6.03E-46
KCNH4	-0.503934783	3.48E-06
KCNH5	-0.361832513	1.56E-08
KCNH6	-0.426707599	2.30E-23
KCNH7	0.277120566	4.12E-22
KCNH8	-1.140875872	1.13E-30
KCNIP1	1.743819836	2.38E-116
KCNIP2	-2.804919424	4.00E-103
KCNIP3	-1.031851262	1.08E-32
KCNIP4	-2.525233166	7.41E-105
KCNJ1	-0.3333726	5.72E-10
KCNJ10	0.58894499	2.63E-12
KCNJ11	-0.83058416	7.26E-15
KCNJ12	-1.935206125	2.64E-34
KCNJ13	-0.211907316	7.20E-09
KCNJ14	0.130331337	0.010348996
KCNJ15	0.311952646	2.99E-158
KCNJ16	1.183227324	2.56E-37
KCNJ18	-0.022217308	0.000767051
KCNJ2	0.872565158	4.25E-31
KCNJ3	-1.717942047	8.86E-26
KCNJ4	-1.490466855	3.43E-13
KCNJ5	1.684696187	7.90E-156
KCNJ6	-1.023502506	3.50E-25
KCNJ8	2.088729295	7.35E-292
KCNJ9	-2.769861768	1.23E-158
KCNK1	-1.728263327	5.34E-36
KCNK10	-0.196255801	0.00354261
KCNK12	-2.182514599	5.45E-67
KCNK13	1.995793098	5.48E-265

KCNK15	0.262403177	8.35E-21
KCNK16	-0.014381377	0.000529067
KCNK17	-0.031109796	0.254724489
KCNK18	-0.069944535	2.06E-07
KCNK2	0.887591478	2.21E-21
KCNK3	-0.705166108	2.05E-10
KCNK4	-2.712135728	4.06E-161
KCNK5	0.65848584	5.75E-162
KCNK6	1.114298919	1.32E-198
KCNK7	0.303977117	5.68E-08
KCNK9	-1.363904211	1.23E-16
KCNMA1	-0.803167671	4.61E-27
KCNMB2	-0.406457142	6.47E-15
KCNMB3	0.166632449	1.64E-16
KCNMB4	0.164910114	0.017490228
KCNN1	-1.566497012	4.62E-37
KCNN2	0.515369809	1.68E-10
KCNN3	1.439286041	3.82E-70
KCNN4	1.043988956	1.38E-56
KCNQ2	-0.991557888	7.78E-13
KCNQ3	-0.738524195	1.31E-10
KCNQ4	-0.457199712	5.77E-08
KCNQ5	-0.564745853	6.33E-05
KCNRG	-0.262697016	4.37E-09
KCNS1	-0.301844013	0.053307178
KCNS2	-0.496240124	9.58E-11
KCNS3	0.681717864	1.38E-21
KCNT1	-3.195572392	2.60E-91
KCNT2	0.904830461	1.11E-57
KCNU1	0.09736349	1.10E-119
KCNV1	-0.999327946	7.49E-18
KCNV2	-0.133235583	6.07E-07
KCP	0.280299222	0.004421843
KCTD1	-0.773583991	4.10E-16
KCTD10	0.712458798	1.39E-44
KCTD11	1.932562136	1.99E-194
KCTD12	2.120842313	1.35E-155
KCTD13	0.085776232	0.229810322
KCTD14	0.44710662	1.85E-09
KCTD15	1.13959195	5.88E-70
KCTD16	-0.697946043	7.35E-17
KCTD17	0.308880651	0.00034563
KCTD18	0.704487178	2.77E-65
KCTD19	-0.027940005	0.286642162
KCTD2	0.157688635	0.119699316
KCTD21	0.739138727	4.42E-55
KCTD3	1.637195217	2.41E-113
KCTD4	-1.349252518	4.51E-24

KCTD6	0.844101066	3.43E-26
KCTD7	0.853121245	5.28E-39
KCTD8	-1.211419345	8.01E-32
KCTD9	1.087333511	2.02E-63
KDF1	-0.487654686	2.48E-15
KDM1A	1.431436097	3.02E-78
KDM1B	1.132304526	2.10E-112
KDM2A	0.548262555	2.77E-10
KDM2B	0.319640238	2.63E-05
KDM3A	1.054285456	8.42E-71
KDM3B	1.216289947	2.98E-79
KDM4A	0.561742793	1.58E-14
KDM4B	1.185096934	4.15E-53
KDM4C	0.071405235	0.49390087
KDM4D	1.348928979	1.53E-245
KDM4E	-0.023836749	0.005082335
KDM5A	1.619525162	6.41E-187
KDM5B	0.606437466	6.89E-20
KDM5C	1.081393324	8.33E-37
KDM5D	-0.462455409	0.006263273
KDM6A	-0.209126173	0.002506608
KDM6B	0.863532662	3.48E-19
KDM7A	0.769064978	1.59E-29
KDM8	0.195604701	0.000583195
KDR	1.958125304	9.97E-203
KDSR	0.688823903	7.46E-41
KEAP1	2.002284525	1.49E-253
KEL	-0.532502203	1.14E-16
KERA	0.168999239	2.43E-40
KHDC1	0.139773661	0.027264481
KHDC1L	-1.054544445	9.20E-49
KHDC3L	-0.382904265	2.52E-14
KHDRBS1	1.95487522	9.54E-144
KHDRBS2	-1.103156818	2.89E-34
KHDRBS3	0.84415286	8.96E-26
KHK	0.198996686	0.009823547
KHNYN	-0.041911722	0.632039513
KHSRP	1.794274123	2.27E-122
KIAA0100	0.68786191	1.16E-21
KIAA0232	0.418651121	3.73E-13
KIAA0319	-1.540813872	7.95E-67
KIAA0319L	0.911406293	2.98E-50
KIAA0513	-1.708989444	4.07E-39
KIAA0586	0.354081973	6.95E-10
KIAA0753	0.782376085	7.56E-36
KIAA0825	0.028014178	0.444490037
KIAA0895	0.798381379	3.93E-19
KIAA0895L	-0.58853788	6.16E-07

KIAA0930	-0.1390482	0.036321063
KIAA1109	-0.724001722	4.51E-25
KIAA1143	1.690898299	1.18E-201
KIAA1191	1.227561069	1.94E-88
KIAA1210	0.013615572	5.61E-06
KIAA1217	0.318739932	1.70E-06
KIAA1328	0.332145928	4.13E-16
KIAA1522	0.796721182	4.32E-19
KIAA1549	1.729591967	2.44E-151
KIAA1549L	-0.497074056	4.27E-05
KIAA1586	1.582669205	8.59E-121
KIAA1614	0.148413225	0.107995457
KIAA1671	-0.278557268	0.00012212
KIAA1755	0.110949159	0.115113999
KIAA1841	0.179651048	0.005492927
KIAA1958	1.075271776	3.31E-88
KIAA2012	-0.286678678	4.24E-07
KIAA2013	2.040669929	3.08E-258
KIAA2026	0.066725476	0.242814738
KIDINS220	0.595081083	9.56E-14
KIF12	-1.459634959	1.07E-74
KIF13A	0.492762289	1.93E-22
KIF13B	0.319263931	2.74E-05
KIF16B	0.574536431	7.48E-44
KIF17	-0.027145532	0.810436178
KIF19	-1.222231397	1.29E-33
KIF1A	-2.336806113	9.45E-216
KIF1B	-0.104916469	0.140451823
KIF1C	-0.609352485	1.81E-13
KIF20B	1.282914444	1.27E-152
KIF21A	0.405506241	2.83E-07
KIF21B	-0.114668664	0.207606399
KIF22	1.581177323	1.22E-150
KIF24	1.354778521	5.08E-222
KIF25	-1.231061724	6.94E-43
KIF26A	0.640928516	3.81E-44
KIF26B	0.65478806	2.92E-27
KIF27	0.826878196	1.75E-129
KIF2A	0.50535669	2.46E-18
KIF2B	-0.014173957	2.24E-06
KIF3A	-0.098552927	0.24877562
KIF3B	0.717089371	7.19E-35
KIF3C	-0.269146201	0.012242843
KIF4B	0.128133951	9.81E-120
KIF5A	-3.547037333	1.43E-159
KIF5B	0.048729548	0.44222061
KIF6	-0.082616641	0.08934449
KIF7	1.503088978	9.82E-226

KIF9	1.342418699	5.84E-168
KIFAP3	0.969190297	3.31E-33
KIFC2	-2.142196673	5.55E-55
KIFC3	0.830386222	2.44E-52
KIN	0.412572866	1.07E-12
KIR2DL1	0.035391247	1.94E-09
KIR2DL3	0.046670475	1.88E-22
KIR2DL4	0.273788708	2.46E-123
KIR2DS4	0.072023247	2.19E-20
KIR3DL1	0.054300108	5.80E-27
KIR3DL2	0.061074482	2.42E-37
KIR3DL3	0.024861347	1.68E-06
KIR3DX1	0.015615786	0.426524998
KIRREL2	1.203042324	2.52E-194
KIRREL3	-1.199364047	1.75E-48
KISS1	-0.183443101	0.007863787
KISS1R	0.58163371	9.06E-19
KIT	-0.26471042	0.021825666
KITLG	2.014625768	9.51E-152
KIZ	0.857399083	1.86E-36
KL	-0.173194224	0.006726336
KLB	-0.302969575	2.15E-23
KLC1	-3.07022965	7.56E-222
KLC2	-0.927036388	4.17E-16
KLC3	-0.176702132	4.39E-12
KLC4	0.133415707	0.060601353
KLF1	0.384136881	9.14E-130
KLF11	0.798661794	7.37E-24
KLF12	1.114596812	1.29E-89
KLF13	0.191252723	0.027057599
KLF14	0.049000679	5.48E-08
KLF15	1.056466316	2.47E-68
KLF16	0.261346828	0.001229862
KLF17	0.531969545	2.05E-286
KLF2	0.355539538	1.36E-06
KLF3	1.68313498	2.13E-229
KLF4	0.247253489	0.001411744
KLF5	0.306165783	0.000865524
KLF6	2.367423301	6.55E-208
KLF7	0.982132767	2.69E-41
KLF8	-0.662439691	1.22E-27
KLF9	0.969570081	2.61E-24
KLHDC1	-0.052432007	0.442108684
KLHDC10	1.284596331	2.36E-70
KLHDC2	0.163770005	0.044635933
KLHDC3	0.812463648	9.01E-26
KLHDC4	0.1739304	0.009177882
KLHDC7A	0.30333438	2.16E-25

KLHDC7B	0.724563794	7.15E-278
KLHDC8A	3.757727245	4.08E-188
KLHDC8B	1.536213157	1.11E-151
KLHDC9	0.029334384	0.689267812
KLHL1	-1.151627601	1.01E-39
KLHL10	-0.071703624	0.001186236
KLHL11	0.535991574	4.77E-18
KLHL12	1.646258707	4.66E-141
KLHL13	1.983689207	1.42E-134
KLHL14	0.173047778	7.04E-08
KLHL15	0.704043777	8.53E-41
KLHL17	0.435387091	1.30E-06
KLHL18	0.481345444	4.56E-10
KLHL2	-0.536145573	1.72E-17
KLHL20	1.686984354	1.23E-189
KLHL21	0.8395105	1.43E-45
KLHL22	0.520712172	1.41E-12
KLHL23	1.14051764	2.14E-100
KLHL24	0.991424089	7.11E-62
KLHL25	2.164576543	3.14147631384415e-311
KLHL26	0.074464819	0.261612406
KLHL28	1.041815855	1.92E-77
KLHL29	-0.299372347	0.000152704
KLHL3	-1.782791604	3.70E-51
KLHL30	-0.061658955	0.029661134
KLHL31	0.04168976	0.029701152
KLHL32	-0.84426335	1.31E-37
KLHL33	-0.105059266	0.011499876
KLHL34	-0.48570244	4.65E-22
KLHL35	-0.668743581	2.60E-26
KLHL36	1.221512206	1.03E-89
KLHL38	0.15710657	5.70E-13
KLHL4	2.578871245	1.37E-201
KLHL40	-0.065867493	5.36E-06
KLHL41	-0.764864208	6.15E-37
KLHL42	0.701487419	2.89E-24
KLHL5	1.086332349	4.29E-95
KLHL6	1.203350427	1.80E-135
KLHL7	2.040654275	1.20E-202
KLHL8	1.617186995	2.03E-206
KLHL9	1.202752997	2.76E-72
KLK1	0.025343211	0.268476492
KLK10	-0.49815331	1.92E-21
KLK11	-0.088636494	4.65E-13
KLK12	-0.01700548	0.001352819
KLK13	-0.001932696	0.868817927
KLK14	0.724141618	7.07E-35
KLK15	-0.009678989	0.004943589

KLK2	-0.069183613	4.54E-05
KLK3	-0.141465109	1.72E-07
KLK4	-0.054202492	0.013985941
KLK5	-0.718317343	1.57E-24
KLK6	-1.065166886	1.75E-12
KLK7	-1.366199075	1.16E-23
KLK8	-0.156266173	5.80E-10
KLK9	-0.018050022	7.65E-05
KLKB1	-0.928407472	1.02E-63
KLLN	0.140540368	3.64E-07
KLRB1	0.622722072	6.11E-91
KLRC1	0.055169998	0.072395423
KLRC2	0.556354116	1.23E-14
KLRC3	-0.925742249	1.33E-53
KLRC4	-0.408596596	5.82E-21
KLRD1	0.023620013	0.088781071
KLRF1	0.298466767	6.14E-28
KLRF2	0.074849366	2.06E-55
KLRG1	1.049792123	2.11E-109
KLRG2	0.131589467	1.66E-40
KLRK1	-0.934021391	3.32E-84
KMO	0.622611887	8.68E-105
KMT2A	-0.556944898	6.99E-12
KMT2B	0.699304792	2.15E-15
KMT2C	0.691567194	9.34E-22
KMT2D	0.203121059	0.01664433
KMT2E	0.710004053	3.01E-21
KMT5A	0.945272182	3.72E-73
KMT5B	0.805932235	5.01E-32
KMT5C	0.060231018	0.481872156
KNCN	-0.299453823	4.64E-08
KNDC1	-1.822391009	1.19E-69
KNG1	-0.051164853	0.000388595
KNOP1	0.897564338	2.30E-81
KNSTRN	1.446719413	1.06E-97
KNTC1	1.798699745	5.17E-288
KPNA1	1.028125386	3.37E-54
KPNA2	2.137844227	2.69E-181
KPNA3	1.7737545	5.69E-182
KPNA4	1.19064174	3.30E-102
KPNA5	0.251468529	0.000208875
KPNA6	0.989849077	3.22E-40
KPNA7	0.189932376	1.88E-119
KPNB1	1.362702878	7.09E-100
KPRP	-0.231375747	1.22E-12
KPTN	0.42176936	1.34E-07
KRAS	0.950810929	1.10E-40
KRBA1	1.064871798	1.15E-57

KRBA2	0.759641202	6.60E-61
KRBOX1	0.280854895	5.29E-24
KRBOX4	1.127777752	8.57E-72
KRCC1	1.87531019	3.88E-235
KREMEN1	0.302815062	9.96E-06
KREMEN2	0.763895966	4.85E-47
KRI1	0.879157554	3.02E-38
KRIT1	-0.807365663	3.79E-31
KRR1	0.643550558	1.76E-21
KRT1	-1.264815729	3.51E-47
KRT10	1.926386888	1.26E-227
KRT12	0.039405753	5.44E-19
KRT13	-1.077853926	2.89E-31
KRT14	-1.024164942	2.74E-45
KRT15	-0.159541111	6.34E-15
KRT16	0.303567974	9.29E-26
KRT17	-0.106803755	0.320829536
KRT18	-0.386609276	0.002908973
KRT19	-1.375524644	1.67E-26
KRT2	-0.645675126	1.57E-26
KRT20	0.042285107	1.18E-07
KRT222	-2.978897902	1.72E-140
KRT23	0.070337089	4.27E-06
KRT24	-0.088518427	1.77E-08
KRT25	-0.04999832	5.05E-05
KRT26	-0.005814575	0.001598208
KRT27	-0.044411702	3.62E-10
KRT28	-0.01290718	4.27E-05
KRT3	-0.029190988	0.001208603
KRT31	-1.376607302	1.09E-14
KRT32	0.081499377	1.24E-29
KRT33A	0.041034736	0.000217305
KRT33B	-0.110641347	3.84E-10
KRT34	-0.130972662	9.88E-06
KRT35	-0.01470927	0.002767176
KRT36	0.153998382	2.73E-145
KRT37	0.002598459	0.469948887
KRT38	-0.006311907	0.016076384
KRT39	-0.030204978	0.044755087
KRT4	-0.777600824	6.76E-25
KRT40	0.017858477	0.1053491
KRT5	-0.608250814	2.35E-17
KRT6A	-0.576168491	4.01E-24
KRT6B	-0.071733158	0.000692572
KRT6C	-0.077781404	9.84E-07
KRT7	0.485964133	3.36E-82
KRT71	-0.009880263	0.317466606
KRT72	-0.098034206	8.36E-07

KRT73	-0.016340049	0.000236225
KRT74	-0.033112728	0.042767598
KRT76	-0.002300515	0.080751832
KRT77	-0.067547276	8.11E-07
KRT78	-0.055037411	8.50E-05
KRT79	0.031385615	0.000224228
KRT8	0.03439175	0.473671725
KRT80	0.444071449	4.39E-104
KRT81	-0.323316222	3.82E-15
KRT82	0.001010896	0.837606752
KRT83	-0.146121744	0.002113949
KRT84	0.010151458	0.133044034
KRT85	0.001719047	0.847387296
KRT86	-0.162147673	2.30E-06
KRT9	0.029768055	4.31E-06
KRTAP10-1	-0.008710462	0.0599677
KRTAP10-10	-0.004906394	0.160420175
KRTAP10-11	-0.006146708	0.042299433
KRTAP10-12	-0.006440103	0.006441605
KRTAP10-2	-0.006884592	0.181010307
KRTAP10-3	-0.010115536	0.008202619
KRTAP10-4	-0.00483368	0.113587992
KRTAP10-5	-0.007910012	0.042499259
KRTAP10-6	-0.00012683	0.96291796
KRTAP10-7	-0.005207847	0.043969029
KRTAP10-8	-0.003708693	0.15476887
KRTAP10-9	-0.002808163	0.333710407
KRTAP1-1	-0.012432919	0.021163115
KRTAP11-1	-0.001942952	0.855518203
KRTAP12-1	-0.006135755	0.073403477
KRTAP12-2	-0.004935386	0.092244841
KRTAP12-3	-0.004668926	0.110772431
KRTAP12-4	0.000530032	0.852823784
KRTAP1-3	-0.016675518	0.001193781
KRTAP13-1	-0.001717139	0.488444025
KRTAP13-2	-0.002694392	0.578972985
KRTAP13-3	-0.000850378	0.381874307
KRTAP13-4	0.023672337	1.08E-23
KRTAP1-4	0.001797154	0.494812134
KRTAP1-5	0.026939102	1.07E-06
KRTAP15-1	-0.000503936	0.710315752
KRTAP16-1	0.022565993	0.001550803
KRTAP17-1	0.00257327	0.563196826
KRTAP19-1	0.007669522	0.009368407
KRTAP19-2	-0.000851877	0.586005744
KRTAP19-3	-0.004626494	0.124635452
KRTAP19-4	-0.000746074	0.834806923
KRTAP19-5	-0.008602917	0.166399954

KRTAP19-6	0.003603749	0.269762807
KRTAP19-7	0.001684837	0.296592104
KRTAP19-8	0.042621285	4.18E-08
KRTAP20-1	-0.000779323	0.641531053
KRTAP20-2	0.011832413	0.010924601
KRTAP20-3	0.002823225	0.022690625
KRTAP20-4	0.015840234	2.79E-05
KRTAP2-1	-0.005604285	0.035646485
KRTAP21-1	0.015121409	0.01052372
KRTAP21-2	0.110851791	4.45E-40
KRTAP21-3	0.00199489	0.15346052
KRTAP2-2	-0.005353634	0.062096472
KRTAP22-1	0.004106399	0.03912557
KRTAP22-2	0.006418343	0.024317118
KRTAP2-3	-0.0021669	0.84088509
KRTAP23-1	-0.000893784	0.622656387
KRTAP2-4	-0.013308842	0.225172074
KRTAP24-1	0.004209561	0.039292589
KRTAP25-1	-0.000647042	0.598444312
KRTAP26-1	0.003645171	0.180168062
KRTAP27-1	0.010747765	4.36E-07
KRTAP29-1	0.017143346	2.89E-10
KRTAP3-1	-0.02275192	0.005396407
KRTAP3-2	0.013473521	0.126175127
KRTAP3-3	-0.019036919	0.005087236
KRTAP4-1	0.005220479	0.274240013
KRTAP4-11	0.007266893	0.001229163
KRTAP4-12	-0.000268388	0.971281069
KRTAP4-2	0.00625164	0.045561898
KRTAP4-3	-0.000102628	0.966929628
KRTAP4-4	0.002161974	0.463229609
KRTAP4-5	-0.004655307	0.024128068
KRTAP4-6	-0.004265457	0.249337892
KRTAP4-7	-0.012753422	0.00215893
KRTAP4-8	0.012181349	1.74E-06
KRTAP4-9	0.003679274	0.405747436
KRTAP5-1	-1.100136661	5.58E-22
KRTAP5-10	0.054727523	0.076861019
KRTAP5-11	-0.32627196	1.91E-26
KRTAP5-2	-0.000926206	0.891907724
KRTAP5-3	-0.036655935	1.76E-07
KRTAP5-4	-0.076824581	1.26E-11
KRTAP5-5	-0.204538692	1.50E-06
KRTAP5-6	0.092404872	1.72E-17
KRTAP5-7	-0.625552512	6.39E-83
KRTAP5-8	-0.342011399	7.36E-38
KRTAP5-9	-0.272892919	8.12E-10
KRTAP6-1	-0.00465839	0.219703362

KRTAP6-2	0.003295467	0.095164318
KRTAP6-3	-0.00127586	0.436018691
KRTAP7-1	0.037273555	3.27E-08
KRTAP8-1	0.010388323	0.08725739
KRTAP9-1	0.003817463	0.014430648
KRTAP9-2	-0.002317883	0.08859152
KRTAP9-3	-0.006725109	0.072404633
KRTAP9-4	-0.007631825	0.04192247
KRTAP9-6	0.005637766	3.81E-07
KRTAP9-7	-0.001767577	0.1949037
KRTAP9-8	-0.003355881	0.06320975
KRTAP9-9	-0.008020214	0.007611914
KRTCAP3	0.283593606	9.21E-06
KRTDAP	-0.798084057	1.03E-49
KSR1	-0.263820456	7.61E-06
KSR2	-1.483376527	5.35E-58
KTI12	0.853344462	4.96E-41
KTN1	-0.664228085	9.52E-36
KXD1	0.701073072	1.39E-56
KY	-0.311921635	9.35E-11
KYAT3	1.457002929	5.76E-118
L1CAM	-2.208427897	3.23E-38
L1TD1	0.06794514	1.10E-08
L2HGDH	0.544243241	3.34E-19
L3HYPDH	1.319690964	6.93E-97
L3MBTL1	-2.247782469	4.75E-121
L3MBTL2	-0.028041664	0.700911187
L3MBTL3	0.712109133	1.48E-59
L3MBTL4	1.120832894	2.70E-124
LACC1	-0.609097619	1.47E-12
LACRT	0.004113687	0.059183078
LACTB	1.445741603	1.36E-215
LACTB2	1.387263839	2.48E-98
LACTBL1	0.005285331	0.000456135
LAD1	-0.051753942	0.00025686
LAG3	0.914862555	3.20E-68
LAGE3	1.338327637	1.38E-107
LAIR1	2.961598949	7.44E-238
LAIR2	0.316944944	1.20E-34
LALBA	0.055983975	1.69E-20
LAMA1	0.744543602	1.31E-38
LAMA2	1.589138107	1.03E-169
LAMA3	-0.464781113	7.91E-28
LAMA5	0.574667295	1.42E-05
LAMB1	1.062165381	1.29E-20
LAMB3	1.270433024	2.38E-106
LAMC2	-0.117557925	0.175970011
LAMP1	1.190747603	2.95E-83

LAMP2	1.653592129	1.24E-79
LAMP5	-0.390973497	0.015790097
LAMTOR3	1.811559626	2.68E-152
LAMTOR4	1.674679923	1.11E-229
LAMTOR5	0.781671398	3.36E-56
LANCL1	0.107112753	0.218856683
LANCL2	2.064961127	2.31E-173
LANCL3	0.293046398	2.26E-18
LAPTM5	4.129095067	2.32E-211
LARGE1	0.071197456	0.442305236
LARGE2	0.261021778	6.29E-30
LARP1	0.882462475	1.30E-35
LARP1B	0.610924995	5.94E-29
LARP4	1.497038572	6.88E-147
LARP4B	0.346936923	1.70E-08
LARP6	0.037815843	0.609538
LARP7	0.85351624	4.86E-74
LARS2	0.256628358	1.06E-06
LAS1L	-0.922048282	5.27E-27
LASP1	1.658957577	8.02E-157
LAT	-2.855386682	1.02E-214
LAT2	2.726646663	2.95E-227
LATS1	0.941089996	2.11E-44
LATS2	1.730354489	1.02E-206
LAX1	0.342333309	1.81E-246
LAYN	1.884084523	2.47E-135
LBH	2.668422892	2.55E-180
LBHD1	-0.136032911	0.017783207
LBP	0.993136168	1.57E-251
LBX1	0.262777554	0.000117835
LBX2	0.71791012	9.03E-178
LCA5	0.349333677	1.21E-11
LCA5L	0.702034466	8.46E-66
LCAT	1.344971003	2.70E-35
LCE1A	-0.075938171	0.000566735
LCE1B	-0.033952729	0.000868009
LCE1C	0.181619975	1.02E-12
LCE1D	0.222128553	3.67E-56
LCE1E	0.077323512	8.78E-20
LCE1F	-0.008387104	0.295229528
LCE2A	-0.026222795	0.000395745
LCE2B	-0.114383164	1.71E-09
LCE2C	-0.053325206	4.30E-06
LCE2D	-0.0321542	0.000513672
LCE3A	-0.002160565	0.664512356
LCE3B	-0.001533365	0.307440828
LCE3C	-0.001851731	0.398676285
LCE3D	-0.024036786	0.000388983

LCE3E	-0.006127825	0.061666031
LCE4A	-0.000648152	0.718613616
LCE5A	-0.077343817	4.92E-07
LCE6A	-0.031188948	0.0001188
LCK	0.962477934	4.74E-206
LCMT1	0.826385212	3.92E-31
LCMT2	1.749551026	4.84E-249
LCN1	0.015002595	0.735554398
LCN10	-0.904783268	1.32E-65
LCN12	-1.281293081	8.81E-94
LCN15	-0.490036903	9.49E-16
LCN2	0.029385738	0.52608158
LCN6	-0.612989394	6.03E-50
LCN8	-0.945173184	7.12E-10
LCN9	0.092527545	0.000287592
LCNL1	-2.451235552	7.38E-165
LCOR	-0.059473976	0.28225561
LCORL	0.902059865	8.76E-116
LCP1	3.025357808	4.77E-200
LCP2	2.385299434	2.00E-232
LCT	0.023603185	4.35E-06
LCTL	1.565401659	9.57E-240
LDB1	1.214007867	3.18E-50
LDB2	0.143022201	0.287020694
LDB3	-1.348464718	1.53E-39
LDHA	2.035478934	1.41E-122
LDHAL6A	-0.095250466	0.011935925
LDHAL6B	0.04617118	1.00E-05
LDHC	0.207341401	2.28E-09
LDHD	-0.676427996	1.27E-30
LDLR	0.896384483	7.69E-23
LDLRAD1	0.000411794	0.896289466
LDLRAD2	-0.065260809	0.106989693
LDLRAD3	2.686651715	3.22E-182
LDLRAD4	-0.650336312	3.95E-22
LDLRAP1	-0.21506154	0.167261391
LDOC1	-1.545873278	8.48E-75
LEAP2	0.74262145	7.17E-20
LECT2	-0.082593297	2.12E-15
LEF1	1.767731634	4.28E-224
LEFTY1	-0.894865456	6.70E-55
LEKR1	0.370752885	1.63E-81
LELP1	-0.072967967	2.22E-06
LEMD1	0.816588398	1.01E-20
LEMD2	0.250076691	0.00097712
LEMD3	0.793638889	1.15E-32
LENEP	0.224476739	5.82E-46
LENG1	1.224362337	2.24E-115

LENG8	-0.43177352	6.91E-05
LENG9	1.10316726	2.20E-124
LEO1	1.123731749	2.46E-57
LEP	0.057458326	3.48E-06
LEPR	0.331207497	1.99E-14
LEPROT	2.106312532	1.75E-307
LEPROTL1	1.681120493	6.56E-178
LETM1	0.810315312	4.61E-40
LETM2	0.139445492	0.112300969
LETMD1	0.16222353	0.00696714
LEUTX	9.89E-05	0.982623501
LEXM	-0.152363702	6.06E-08
LGALS1	2.827576493	6.16E-242
LGALS12	0.51561361	4.67E-243
LGALS13	-0.12163151	9.64E-09
LGALS14	0.038036545	3.47E-08
LGALS16	-0.209300764	3.46E-12
LGALS3	2.812254608	8.53E-274
LGALS3BP	3.512912816	5.41E-294
LGALS4	-0.08269587	0.078019501
LGALS7	0.03841362	0.021861175
LGALS7B	0.003197037	0.943051842
LGALS8	-0.354882355	3.79E-06
LGALS9	2.437489253	6.72E-189
LGALS9B	0.20364106	4.10E-174
LGALS9C	0.160687767	2.67E-66
LGALSL	1.099933298	8.30E-39
LGI1	-1.562054212	8.66E-75
LGI2	0.306692404	0.000474101
LGI3	-2.50104088	7.09E-153
LGI4	-1.666585764	6.44E-116
LGMN	2.430253581	8.04E-221
LGR4	1.006937371	1.89E-39
LGR5	-0.157484958	0.07172297
LGR6	1.319052875	1.60E-34
LGSN	0.013079685	0.314919754
LHB	0.182627121	3.75E-06
LHCGR	-0.027200069	0.001299859
LHFPL1	0.475190752	1.10E-17
LHFPL3	2.83936533	2.73E-190
LHFPL4	0.225205635	0.019409464
LHFPL5	-0.124710659	0.00773505
LHPP	-0.556511692	1.91E-12
LHX1	-0.886958622	3.84E-07
LHX2	1.235647276	7.37E-11
LHX3	0.21284174	7.50E-73
LHX4	-0.184593587	0.004476585
LHX5	-0.203429971	0.012974444

LHX6	-0.758131365	5.04E-14
LHX8	-0.171257497	0.001411744
LHX9	1.172770741	7.07E-281
LIAS	0.711576746	2.93E-68
LIF	3.195146027	1.80032587590683e-312
LIFR	1.550029181	6.07E-145
LIG1	1.297363262	7.89E-66
LIG3	0.509789637	5.08E-14
LIG4	0.305440306	2.98E-06
LILRA1	1.598529557	6.54E-220
LILRA4	1.090041639	3.39E-52
LILRA5	1.124401361	9.08E-110
LILRA6	1.183848513	1.16E-145
LILRB1	2.152743568	1.32E-174
LILRB2	1.898692308	2.56E-252
LILRB3	1.713279001	1.23E-176
LILRB5	0.14052686	5.93E-05
LIM2	0.010096789	0.000149098
LIMA1	2.59779162	1.92E-90
LIMCH1	0.092202936	0.108417287
LIMD1	1.487128787	1.68E-263
LIMD2	0.957136334	3.53E-32
LIME1	-2.520412839	2.57E-182
LIMK1	0.607257548	6.53E-10
LIMK2	0.702917486	4.24E-22
LIMS2	0.096797698	0.144727982
LIMS3	0.004190609	7.52E-07
LIMS4	0.002953413	0.117752481
LIN28A	0.05190115	1.18E-18
LIN28B	0.039159557	0.064167593
LIN37	-1.753019022	6.18E-138
LIN52	0.653628432	1.43E-23
LIN54	1.047440223	6.08E-90
LIN7A	0.206485222	0.057109174
LIN7B	-0.84391388	1.29E-30
LIN7C	1.071266582	5.93E-45
LIN9	1.715376392	5.94E-220
LINC00634	-0.58060416	1.46E-11
LINC00672	-0.699975722	2.94E-20
LINC02218	-0.029405386	0.005943461
LINGO1	0.458194598	1.55E-05
LINGO2	-0.641395141	1.29E-19
LINGO3	-0.658533502	4.02E-10
LINGO4	-0.420692848	3.67E-05
LINS1	0.780111353	1.08E-60
LIPA	2.177215626	1.82E-142
LIPC	0.595157794	2.91E-80
LIPE	-0.460666627	2.17E-07

LIPF	-0.620476454	1.26E-16
LIPG	1.708707668	3.79E-183
LIPH	0.381834474	2.25E-47
LIPI	0.111499309	5.38E-09
LIPJ	-0.21869178	8.34E-15
LIPK	0.000278737	0.923515819
LIPM	-0.002283242	0.774051869
LIPN	0.111329715	3.69E-32
LIPT1	1.024648986	4.15E-112
LIPT2	0.843238684	2.00E-112
LITAF	2.611514444	4.92E-201
LIX1	1.498245287	6.98E-71
LKAAEAR1	-0.310901233	7.98E-06
LLGL1	0.805566692	3.56E-36
LLGL2	-0.413465595	4.12E-06
LLPH	1.123369103	9.07E-180
LMAN1	2.012693202	3.43E-191
LMAN1L	-1.182483369	3.90E-12
LMAN2L	2.246934378	4.25E-285
LMBR1	1.564199564	1.27E-136
LMBR1L	0.694315759	2.03E-14
LMBRD1	-0.797998516	1.16E-37
LMBRD2	0.918714305	4.73E-50
LMCD1	1.702166613	1.96E-199
LMF1	0.540227417	1.12E-27
LMF2	1.400578235	5.74E-69
LMLN	0.064473761	0.294287101
LMNA	2.258501277	9.93E-213
LMNB1	3.710950811	4.40E-277
LMNB2	2.182972257	1.08E-168
LMNTD1	0.473201224	9.81E-72
LMNTD2	-0.564228653	1.57E-07
LMO1	2.488656549	3.97E-270
LMO2	2.331427399	7.41E-254
LMO3	-0.580017101	1.93E-06
LMO4	1.979878432	1.82E-80
LMO7	-0.491079818	2.25E-07
LMOD1	-0.024773931	0.715667057
LMOD2	-0.068848472	0.00051286
LMOD3	0.351088501	2.74E-53
LMTK2	0.161907086	0.091030678
LMTK3	-0.891602014	9.31E-23
LMX1A	-0.080004347	0.00317086
LMX1B	-0.028651918	0.604276443
LNP1	0.523162335	2.47E-12
LNPEP	0.350935603	1.93E-06
LNPK	1.87412789	5.33E-198
LNX1	0.296480797	0.00297739

LNX2	0.712514413	7.40E-25
LONP1	1.15089406	7.33E-83
LONP2	0.784594212	6.58E-35
LONRF1	1.093566466	2.22E-61
LONRF2	-1.054206469	7.83E-24
LONRF3	0.534503403	1.06E-11
LOXHD1	-0.258114157	1.07E-15
LOXL1	2.478999998	8.83202370917198e-317
LOXL4	1.097216106	5.26E-159
LPA	-0.025772381	0.049447798
LPAR1	-0.146332371	0.220756573
LPAR2	1.606817491	1.12E-138
LPAR3	-0.924216191	1.35E-31
LPAR4	1.269350914	1.19E-208
LPAR5	3.150287856	1.06020786079979e-316
LPAR6	2.424115002	1.29E-198
LPCAT1	2.308346605	8.18E-201
LPCAT2	1.769453975	2.46E-74
LPCAT3	0.828619625	1.39E-20
LPCAT4	-1.376368608	1.05E-28
LPGAT1	0.032114641	0.65833064
LPIN1	-0.725478346	2.29E-23
LPIN2	0.91969254	2.47E-42
LPIN3	0.160669715	0.010571877
LPL	2.697303541	4.81E-112
LPO	-0.069958109	0.013666668
LPP	1.308414493	5.84E-209
LPXN	2.023587398	2.22E-272
LRAT	0.669916199	1.11E-26
LRBA	0.807013507	5.83E-45
LRCH1	0.379274328	0.002519728
LRCH2	0.814619173	4.03E-55
LRCH3	0.720950517	5.22E-22
LRCH4	-2.119923272	1.81E-137
LRCOL1	0.53981023	9.45E-166
LRFN1	0.210612661	0.027863244
LRFN2	-0.693546371	1.04E-13
LRFN3	1.276357946	1.10E-90
LRFN4	0.599520624	3.99E-11
LRFN5	-1.054084709	1.68E-34
LRG1	0.741071149	4.14E-93
LRGUK	0.914597974	3.06E-189
LRIF1	0.982443418	1.07E-65
LRIG1	1.746179615	4.60E-131
LRIG2	0.74019747	1.88E-36
LRIG3	1.708345554	1.17E-230
LRIT1	-0.009992912	0.001544035
LRIT2	-0.357153367	1.83E-13

LRIT3	-0.14416409	0.000104514
LRP1	1.558742331	1.32E-79
LRP10	2.604730551	1.56E-291
LRP12	0.417686111	1.81E-11
LRP1B	0.273269358	5.90E-09
LRP2	-0.390411391	2.52E-05
LRP2BP	-0.341978836	8.60E-07
LRP3	-0.455349972	1.34E-08
LRP4	0.810158294	4.63E-27
LRP6	0.866468143	7.31E-58
LRP8	-0.895228854	1.95E-27
LRPAP1	0.89145757	1.19E-30
LRPPRC	0.735672852	6.94E-21
LRRC1	0.875983679	1.29E-59
LRRC10	0.143524716	4.19E-52
LRRC10B	-1.394001978	1.11E-17
LRRC14	0.454691972	8.45E-13
LRRC14B	-0.321725373	5.78E-17
LRRC15	0.702923006	3.85E-205
LRRC18	0.365808199	8.63E-32
LRRC19	0.046939167	3.20E-11
LRRC2	0.993805443	5.50E-31
LRRC20	0.279378776	0.000357642
LRRC23	0.859547466	2.11E-52
LRRC26	-0.601991443	1.33E-16
LRRC27	0.252438554	1.48E-08
LRRC28	0.754669872	1.27E-37
LRRC3	-0.010674932	0.75903448
LRRC30	-0.001736721	0.347989409
LRRC31	0.022954069	0.001640365
LRRC32	0.918905346	7.94E-32
LRRC34	0.680532126	2.39E-39
LRRC36	1.018432693	4.89E-142
LRRC37A	-0.247099935	3.92E-14
LRRC37A2	-0.507491672	1.80E-11
LRRC37A3	0.506758801	2.05E-18
LRRC37B	0.048714629	0.536457507
LRRC38	-0.628428786	8.50E-12
LRRC39	-0.118538816	0.043988505
LRRC3B	-1.199619997	6.72E-51
LRRC3C	0.288271429	6.67E-125
LRRC4	0.205324344	0.073159246
LRRC40	1.595609729	1.10E-120
LRRC41	1.629282476	5.60E-123
LRRC43	0.079567029	0.185098507
LRRC45	0.759529032	2.20E-14
LRRC46	0.959472778	3.35E-27
LRRC47	1.139877507	1.47E-52

LRRC49	0.191088438	0.006803672
LRRC4B	0.240633622	0.000356834
LRRC4C	0.933341993	5.19E-30
LRRC52	0.189982252	9.30E-61
LRRC53	-0.015327933	0.471760717
LRRC55	3.239672681	8.13E-282
LRRC56	-0.62664012	1.04E-14
LRRC57	0.705341803	1.34E-47
LRRC58	1.644142036	2.85E-170
LRRC59	2.443750548	3.89E-270
LRRC6	0.469684056	4.73E-13
LRRC61	0.050353157	0.491087969
LRRC63	-0.273502064	0.000208247
LRRC66	0.383218366	1.23E-18
LRRC69	0.392187614	8.03E-171
LRRC7	-1.70249974	1.47E-69
LRRC70	-0.191967778	6.59E-09
LRRC71	0.369731143	2.57E-07
LRRC72	-0.026343789	3.82E-06
LRRC73	-0.48015289	5.63E-07
LRRC74A	0.309849309	1.02205877958241e-317
LRRC74B	-0.346061168	2.11E-06
LRRC75A	0.924641699	8.40E-32
LRRC75B	0.037578238	0.587798354
LRRC8A	1.309951162	1.97E-77
LRRC8B	0.519758451	1.29E-08
LRRC8C	1.083980241	1.66E-111
LRRC8D	1.450168051	3.17E-90
LRRC8E	0.058731208	1.79E-26
LRRC9	0.214768343	2.40E-13
LRRCC1	1.719970864	8.18E-164
LRRD1	-0.126765804	4.55E-09
LRRFIP1	0.52332287	2.07E-17
LRRFIP2	0.015080818	0.817992677
LRRIQ1	-0.165832802	0.000849813
LRRIQ3	0.332742834	5.22E-130
LRRIQ4	0.174928554	1.03E-90
LRRK1	0.419629432	3.53E-11
LRRK2	0.775186239	4.68E-60
LRRN1	1.95524663	9.18E-152
LRRN2	1.497991349	1.24E-72
LRRN3	2.185074887	7.29E-143
LRRN4	-0.168057494	7.59E-07
LRRTM1	-0.401414686	0.000408949
LRRTM2	0.408831434	7.17E-07
LRRTM3	0.68731037	1.25E-18
LRRTM4	0.370018618	2.52E-06
LRSAM1	-0.361216956	1.48E-06

LRTM1	0.068411354	5.32E-10
LRTM2	-1.869496874	4.24E-45
LRTOMT	0.990469309	4.21E-118
LRWD1	0.951444876	1.30E-29
LSAMP	0.44187343	1.38E-10
LSG1	1.66135627	2.01E-163
LSM1	0.996780149	4.48E-88
LSM11	0.148108657	0.125693287
LSM12	1.809598816	6.28E-267
LSM14A	1.372185347	8.67E-125
LSM14B	1.226190112	8.86E-48
LSM3	2.059521328	2.16E-289
LSM4	1.769824569	4.51E-213
LSM6	1.588281736	2.80E-179
LSM7	1.89632236	1.84E-229
LSM8	1.551474966	6.01E-210
LSMEM1	-1.308257451	2.69E-68
LSMEM2	-0.244672662	8.03E-07
LSR	0.728177551	2.71E-39
LSS	0.102100495	0.158118719
LST1	2.696840789	1.79E-172
LTA	0.212459969	8.62E-07
LTA4H	0.167350874	0.026702681
LTB	0.604590718	2.67E-58
LTB4R	0.897146448	4.77E-44
LTB4R2	-0.325887798	4.31E-07
LTBP1	2.754840486	9.48E-306
LTBP2	1.684040026	1.73E-237
LTBP3	1.475292366	8.22E-101
LTBP4	1.775535971	2.97E-94
LTBR	0.886013506	3.85E-47
LTK	-0.035254312	0.660296115
LTN1	1.000025312	1.26E-50
LTV1	0.50530543	1.84E-16
LUC7L	-0.162826376	0.017567227
LUC7L2	-0.71524581	9.31E-29
LUC7L3	-0.580060511	4.15E-10
LURAP1	-0.288589161	0.000202841
LURAP1L	0.230181512	0.039429227
LUZP1	0.50843091	5.96E-12
LUZP2	0.643949435	1.16E-15
LUZP4	0.006168681	0.019993723
LVRN	0.16937221	6.76E-45
LXN	2.37956392	1.29E-208
LY6D	-0.50203311	7.87E-26
LY6E	0.627768933	4.49E-17
LY6G5B	-2.110405209	1.39E-78
LY6G5C	0.732111811	5.68E-21

LY6G6C	0.141156905	3.85E-05
LY6G6D	-0.19381628	2.27E-21
LY6G6F	-0.0203054	0.005166379
LY6H	-1.787209675	1.70E-20
LY6K	-0.808674397	7.32E-146
LY6L	-0.004047305	0.370444119
LY75	0.579404811	1.79E-55
LY86	3.959159311	8.08E-272
LY9	0.149276844	1.71E-61
LYAR	1.664979315	1.37E-242
LYG1	0.616669007	1.18E-21
LYG2	0.198592848	8.05E-13
LYL1	1.357546732	2.08E-105
LYN	2.744486481	1.38E-215
LYNX1	3.16713896	1.93E-263
LYPD1	0.678258343	1.11E-17
LYPD2	0.006271245	0.570537912
LYPD3	0.117706423	0.001021334
LYPD4	-0.00557066	0.121892448
LYPD5	-0.041542985	0.529454067
LYPD6	1.29028967	2.60E-114
LYPD6B	-0.195581381	3.22E-05
LYPD8	-0.782260522	1.95E-22
LYPLA2	1.561917625	5.86E-144
LYPLAL1	1.375216634	1.08E-135
LYRM1	1.337225329	5.59E-138
LYRM2	1.356956597	4.69E-161
LYRM7	1.175263761	7.54E-99
LYRM9	-1.799103862	4.12E-126
LYSMD1	1.485294755	1.97E-103
LYSMD2	0.352329082	2.79E-06
LYSMD3	1.412170127	8.78E-121
LYSMD4	-0.420078225	2.29E-09
LYST	0.767181944	5.68E-23
LYVE1	1.103663912	2.57E-47
LYZ	3.788890385	9.43452935329069e-318
LYZL1	-0.010926836	0.006322293
LYZL2	0.019626167	5.91E-09
LYZL4	-0.127930659	1.31E-05
LYZL6	-0.000988271	0.712194171
LZIC	2.007576061	3.05E-281
LZTFL1	0.922068269	2.98E-41
LZTR1	-1.289563158	9.04E-67
LZTS1	1.527650138	2.29E-33
LZTS2	0.163720129	0.015425189
LZTS3	-1.11527886	5.47E-19
M1AP	0.596530538	3.28E-49
M6PR	1.714855214	4.25E-115

MAB21L1	0.488022991	0.003781871
MAB21L2	0.093952234	0.111666162
MAB21L3	-0.114221254	7.52E-12
MACF1	0.14572025	0.010509093
MACROD1	1.050050804	1.39E-50
MACROD2	-0.905186394	6.38E-45
MAD2L1BP	2.124805171	4.50E-182
MADCAM1	-0.397421831	1.57E-10
MADD	-0.408845937	0.000364841
MAEA	0.356280328	1.49E-06
MAEL	1.340817858	2.67E-121
MAF	1.751597314	2.09E-130
MAF1	1.484788022	5.83E-163
MAFA	0.140243922	1.28E-05
MAFF	0.755802279	9.79E-14
MAFG	0.374815499	9.61E-10
MAFK	-0.271210187	4.35E-05
MAG	-1.172480299	7.46E-15
MAGEA1	0.101120289	4.69E-31
MAGEA10	0.006704985	0.000167363
MAGEA11	0.009228835	0.112672616
MAGEA12	0.835752154	4.71E-273
MAGEA2	0.003396769	1.20E-05
MAGEA2B	0.003825796	5.84E-07
MAGEA3	0.121221045	7.07E-47
MAGEA4	0.027219493	0.005664996
MAGEA6	0.110802368	9.21E-88
MAGEA8	-0.067653972	0.000182924
MAGEA9	0.013853112	3.66E-06
MAGEA9B	0.031246338	3.12E-05
MAGEB1	0.007409031	0.001427946
MAGEB10	0.001790634	0.070817614
MAGEB16	0.001295257	0.375542252
MAGEB17	0.389590022	2.85E-182
MAGEB18	0.002738738	0.001191297
MAGEB2	0.035858292	5.62E-12
MAGEB3	0.002092243	0.371294924
MAGEB4	-0.001801142	0.185819203
MAGEB5	0.00121019	0.258405954
MAGEB6	0.035377269	3.73E-49
MAGEC1	0.07306867	3.52E-30
MAGEC2	0.289882962	4.83E-68
MAGEC3	-0.772357776	3.04E-47
MAGED1	2.547785302	1.56E-192
MAGED2	2.079678914	4.75E-204
MAGEE1	-1.211255584	2.28E-26
MAGEE2	0.129105507	0.143345654
MAGEF1	1.636523053	3.37E-113

MAGEH1	1.229885847	9.68E-56
MAGEL2	0.141115278	0.1820212
MAGI1	0.54301755	4.84E-10
MAGI2	0.762812981	2.94E-71
MAGI3	0.603338513	5.19E-19
MAGIX	-0.371239901	1.72E-05
MAGOHB	1.558553753	5.96E-237
MAIP1	1.3265656	3.51E-107
MAJIN	-0.025790438	0.11672536
MAK	0.764310479	5.44E-85
MAK16	1.220671124	1.50E-107
MAL	-1.79162587	3.65E-63
MAL2	-2.853042879	2.08E-82
MALL	0.318036538	1.71E-65
MALRD1	0.110831734	1.01E-15
MALSU1	1.447973066	8.17E-136
MALT1	0.893819756	7.60E-39
MAMDC2	1.053978986	7.03E-157
MAMDC4	-0.097581438	0.515103047
MAML1	1.726922362	1.03E-87
MAML2	2.504677961	6.03E-279
MAML3	0.542674574	1.25E-08
MAMLD1	1.182296742	4.54E-70
MAMSTR	0.411706956	3.92E-08
MAN1A1	1.132699747	4.85E-36
MAN1A2	1.056792003	1.64E-79
MAN1B1	0.941343411	1.08E-43
MAN1C1	2.065391902	6.65E-99
MAN2A1	1.81749731	1.09E-102
MAN2A2	-0.212337182	0.025203035
MAN2B1	2.041122353	1.17E-207
MAN2B2	1.491597943	1.32E-98
MAN2C1	-0.803243296	1.22E-18
MANBA	0.417825616	8.50E-09
MANBAL	2.042424851	4.80E-291
MANEAL	1.412782385	1.43E-83
MANSC1	1.372954081	3.94E-131
MANSC4	-0.179509085	0.00013438
MAOA	0.493814559	1.59E-11
MAOB	1.911541096	3.51E-96
MAP10	1.726774419	5.59E-275
MAP1A	-0.852796069	6.44E-24
MAP1B	0.723290874	4.22E-13
MAP1LC3A	0.476119769	2.42E-10
MAP1LC3B	1.023882195	2.74E-48
MAP1LC3B2	0.993654272	2.97E-29
MAP1S	0.815197465	3.15E-25
MAP2	0.272714109	0.023134495

MAP2K1	0.698705184	8.03E-15
MAP2K2	0.409823635	5.21E-14
MAP2K3	1.485701873	3.27E-92
MAP2K4	-0.174796425	0.051465019
MAP2K5	1.057597498	1.08E-62
MAP2K6	0.511678883	8.29E-28
MAP2K7	-0.105590991	0.124715298
MAP3K1	1.914986227	1.22E-269
MAP3K10	-1.650069247	4.01E-78
MAP3K11	-0.175887962	0.001573741
MAP3K12	-0.439293821	0.000285198
MAP3K13	1.183012032	2.31E-136
MAP3K14	1.113556063	1.44E-54
MAP3K15	0.222963316	3.51E-20
MAP3K19	0.430096447	1.28E-13
MAP3K2	1.205423785	3.44E-80
MAP3K20	1.41069243	9.89E-182
MAP3K21	-0.873966671	2.20E-22
MAP3K3	0.427234708	9.53E-09
MAP3K4	0.351391556	5.71E-06
MAP3K5	0.290062159	1.88E-05
MAP3K6	0.904566354	1.95E-21
MAP3K7	1.157656793	1.85E-64
MAP3K8	1.470991109	1.63E-106
MAP3K9	-1.743339122	1.41E-48
MAP4	0.530630394	5.60E-17
MAP4K1	0.710770826	9.57E-60
MAP4K2	-0.16371878	0.055075027
MAP4K3	1.011437056	1.88E-40
MAP4K4	0.191188322	0.010482526
MAP4K5	-0.310048523	3.27E-06
MAP6	0.185438871	0.004606462
MAP6D1	-4.56527524	1.10670704668439e-321
MAP7	-1.363369198	4.19E-64
MAP7D1	0.156388911	0.03328949
MAP7D2	-2.480580019	4.87E-94
MAP9	0.35872873	1.11E-09
MAPK1	1.788377817	5.71E-120
MAPK10	-1.589127862	8.77E-101
MAPK11	-0.425604191	2.60E-06
MAPK12	-0.053869577	0.539117085
MAPK13	0.282867576	0.001858542
MAPK14	1.467499208	3.36E-85
MAPK15	-0.616342793	2.14E-07
MAPK1IP1L	1.983916491	1.53E-211
MAPK3	0.216295049	0.000142502
MAPK4	0.559883374	8.69E-12
MAPK6	1.414900216	7.43E-73

MAPK7	1.737027425	1.30E-171
MAPK8	0.056269212	0.509903531
MAPK8IP1	0.182840974	0.014253316
MAPK8IP2	-2.328866085	3.04E-112
MAPK8IP3	-1.622837211	3.61E-54
MAPK9	-0.512914149	6.70E-08
MAPKAP1	1.139935104	3.34E-117
MAPKAPK2	2.264462914	6.03E-290
MAPKAPK5	0.614851092	2.15E-23
MAPKBP1	-0.94834078	1.58E-15
MAPRE2	0.410698793	8.21E-08
MAPRE3	-1.044068419	1.21E-33
MAPT	-1.2213008	1.20E-41
MARCKS	2.761987672	3.90E-263
MARCKSL1	1.988912988	8.81E-149
MARCO	1.7621738	2.61E-146
MARK1	-0.53089521	5.24E-13
MARK2	-0.092736432	0.196357216
MARK3	-1.059404919	3.95E-43
MARK4	0.319506756	2.35E-06
MARS2	2.045721582	7.39616271824346e-321
MARVELD1	2.263149422	6.95E-279
MARVELD2	0.627803165	5.62E-106
MARVELD3	1.031510536	1.27E-296
MAS1	-0.357292543	4.64E-09
MAS1L	0.001479223	0.452535915
MASP2	-0.23511505	0.023490914
MAST1	-1.395764463	6.62E-19
MAST2	1.059694947	1.28E-51
MAST3	-0.645368773	8.14E-07
MAST4	0.454552012	3.33E-16
MASTL	1.132682278	6.92E-99
MAT1A	-0.062019344	0.074341886
MAT2A	1.221190398	2.76E-56
MAT2B	1.061702904	3.41E-53
MATK	-1.881144035	2.66E-57
MATN1	0.081443549	1.22E-05
MATN2	3.036604589	6.71701563487927e-317
MATN3	0.906324418	1.28E-35
MATN4	0.778748894	4.10E-215
MAU2	0.259078124	0.003744838
MAVS	1.562836234	2.28E-157
MAX	0.180891454	0.007564357
MAZ	0.000344419	0.998968964
MB	-0.41285551	1.26E-08
MB21D2	1.240046617	4.10E-60
MBD1	0.863577463	7.19E-36
MBD2	1.377360537	1.03E-124

MBD3	0.402055133	9.69E-14
MBD3L1	0.027076839	7.66E-19
MBD3L2	-0.000644268	0.849518559
MBD3L3	0.000202391	0.960458819
MBD3L4	-0.000450962	0.517444413
MBD3L5	0.000470941	0.683225248
MBD4	0.997230297	5.73E-53
MBD5	0.641372657	8.53E-42
MBD6	1.044037729	8.78E-38
MBIP	1.324038045	1.01E-119
MBL2	-0.002494821	0.15066432
MBLAC1	0.348928616	5.60E-07
MBLAC2	0.182983335	0.005593952
MBNL1	1.004476937	1.24E-73
MBNL2	-0.208721003	0.003484229
MBNL3	1.445413205	4.65955839167348e-311
MBOAT1	1.293420647	4.51E-60
MBOAT2	0.898073572	9.24E-74
MBOAT4	0.202137536	6.73E-70
MBOAT7	0.799492824	6.16E-27
MBP	-3.742848407	9.85E-126
MBTD1	0.934344613	5.51E-37
MBTPS1	0.268224557	0.000610317
MBTPS2	1.698245841	1.66E-136
MC1R	-0.926714122	2.24E-16
MC2R	0.007583373	0.254562168
MC3R	-0.035923623	0.326600423
MC4R	-0.634495648	4.15E-17
MC5R	0.706182839	9.62E-295
MCAM	1.427641978	3.72E-44
MCAT	1.324956217	2.23E-86
MCC	1.702444352	5.80E-196
MCCC1	1.218056135	7.01E-115
MCCC2	1.916122012	6.33E-245
MCCD1	-0.000150563	0.986962574
MCEE	1.545023599	1.15E-158
MCEMP1	0.519784658	1.06E-43
MCF2	-1.349120943	1.32E-77
MCF2L	-1.122823746	9.67E-23
MCF2L2	-3.049423019	3.71E-224
MCFD2	1.315916272	9.92E-78
MCHR1	2.054840882	1.68E-85
MCHR2	-0.398630641	1.24E-06
MCIDAS	0.107567771	4.30E-11
MCL1	1.829476791	8.60E-181
MCM2	2.859060113	3.75E-276
MCM3AP	-0.021768757	0.787970343
MCM4	2.451585925	6.77E-188

MCM6	2.765548986	3.42E-263
MCM7	1.921401093	5.57E-149
MCM8	0.835969077	7.26E-41
MCM9	0.568707361	3.38E-49
MCMBP	1.785906374	2.61E-187
MCMDC2	0.014191911	0.726354798
MCOLN1	0.71661197	1.68E-23
MCOLN2	0.931534702	8.34E-296
MCOLN3	0.085611927	0.008794277
MCPH1	1.255713191	2.76E-149
MCRIP1	1.568975318	2.08E-209
MCRIP2	-0.104887955	0.040146521
MCRS1	1.423235516	1.20E-138
MCTP1	-1.071391985	7.94E-28
MCTP2	0.035182941	0.382750811
MCTS1	0.318840712	4.11E-08
MCU	0.428754892	5.53E-05
MCUR1	1.381231532	2.44E-150
MDC1	1.279049642	4.33E-59
MDGA1	-0.28703114	0.128072148
MDGA2	0.068376897	0.073951998
MDH1	-0.440947612	9.89E-06
MDH1B	0.95055609	4.95E-55
MDH2	2.093171388	8.98E-160
MDM1	0.980820036	3.31E-79
MDM2	1.879392921	1.75E-184
MDM4	1.152861293	1.94E-63
MDN1	-0.379355148	5.43E-07
ME1	-0.698686037	2.40E-23
ME2	1.297293353	1.80E-146
ME3	0.236739227	0.005184645
MEA1	0.149879692	0.011428346
MEAF6	0.399767589	5.42E-09
MECOM	1.005310232	1.27E-101
MECP2	-0.019863279	0.808720018
MECR	1.082288656	3.38E-82
MED1	1.463425771	1.00E-111
MED10	0.792261048	7.92E-36
MED11	1.031830364	6.44E-106
MED12	1.463674949	5.19E-98
MED12L	0.106845438	0.020559844
MED13	1.338818146	1.01E-110
MED13L	-0.226367552	0.009826263
MED14	1.499094749	1.80E-114
MED15	-0.038254276	0.557542256
MED16	1.801987698	7.97E-193
MED17	-1.235284913	8.78E-85
MED19	1.68393751	5.53E-167

MED21	1.312940198	5.00E-100
MED22	1.340086687	1.39E-141
MED23	0.743812776	1.98E-18
MED24	1.085415309	7.99E-42
MED25	-0.282097837	4.70E-05
MED26	0.658840541	2.33E-33
MED27	1.360057812	2.40E-125
MED28	0.862329217	2.52E-49
MED29	1.19607557	5.31E-122
MED30	1.381526895	4.44E-145
MED31	-0.023202591	0.735436685
MED4	0.744945759	1.28E-42
MED6	0.417967811	1.16E-12
MED7	1.271253909	3.83E-156
MED8	0.953602924	1.06E-49
MED9	1.012548062	1.36E-67
MEDAG	1.119651014	5.58E-78
MEF2A	0.908017121	1.56E-28
MEF2C	1.015464383	2.91E-17
MEF2D	0.344401214	0.000511148
MEFV	0.481292049	1.24E-108
MEGF10	0.515512572	6.85E-14
MEGF11	0.234090493	0.037593571
MEGF6	0.215418652	7.42E-05
MEGF8	0.913083371	1.96E-31
MEGF9	0.680566704	5.48E-26
MEI1	0.289727144	5.86E-26
MEI4	-0.223253567	8.21E-25
MEIG1	0.445356149	9.50E-52
MEIKIN	-0.44636532	4.09E-102
MEIOB	0.249454514	1.05E-25
MEIOC	-0.11239942	0.002370024
MEIS1	0.729634285	5.28E-10
MEIS2	0.094573227	0.3333622
MEIS3	1.124081938	4.13E-15
MELTF	0.306766783	1.15E-06
MEMO1	0.173501749	1.68E-05
MEN1	1.295135139	4.87E-61
MEOX1	0.236762255	6.37E-35
MEP1A	0.209155263	1.38E-34
MEP1B	-0.009932167	0.575356332
MEPCE	1.902275562	3.05E-165
MEPE	0.206696548	0.0241663
MERTK	1.672684313	1.25E-98
MESP1	0.375249401	1.87E-08
MESP2	-0.256270516	3.58E-16
MET	1.673118945	1.10E-98
METAP1	1.439870328	3.62E-124

METAP1D	1.480782707	7.08E-242
METAP2	1.991566458	1.44E-269
METRN	1.73435013	1.63E-239
METRNL	2.004077573	4.97E-236
METTL11B	0.436601629	2.15E-175
METTL14	1.147394335	2.48E-86
METTL15	0.778510092	2.81E-61
METTL16	1.487716843	2.11E-199
METTL17	0.22035431	0.015032531
METTL18	1.743740841	7.59E-197
METTL21A	1.068606505	6.03E-162
METTL21C	-0.090204752	0.085764056
METTL22	0.899249338	1.48E-34
METTL23	1.330613852	1.76E-148
METTL24	0.046055787	0.204467741
METTL25	0.774175377	4.67E-54
METTL26	0.231430529	8.75E-05
METTL2A	1.845129873	1.56E-267
METTL2B	1.91661867	6.26E-257
METTL3	0.179921158	0.043858012
METTL4	1.133676033	4.38E-106
METTL5	0.537664376	7.74E-25
METTL6	1.354436555	1.64E-132
METTL7A	1.994487054	3.08E-181
METTL8	1.196095821	2.37E-168
METTL9	1.049554662	1.03E-76
MEX3B	1.02658726	6.11E-48
MEX3D	2.184467451	2.68503112549277e-316
MFAP1	1.744085315	2.83E-183
MFAP3	1.463601889	8.78E-119
MFAP3L	0.636934582	7.25E-31
MFAP5	0.527282565	8.96E-48
MFF	0.213614459	0.000156415
MFGE8	1.62586579	1.65E-144
MFHAS1	1.262437022	1.29E-84
MFN1	1.114640264	8.31E-46
MFN2	-0.173913643	0.00198213
MFRP	-0.041199628	0.04622903
MFSD1	2.056829672	1.65E-246
MFSD10	1.038006712	4.51E-40
MFSD11	1.189798211	8.73E-111
MFSD12	0.991798015	1.23E-44
MFSD13A	0.088681876	0.270445843
MFSD14B	1.628457353	3.08E-208
MFSD2A	2.398661403	1.17E-266
MFSD2B	0.12301063	1.87E-06
MFSD3	0.706504671	1.86E-20
MFSD4A	-1.760010923	4.66E-40

MFSD4B	0.485916789	7.52E-39
MFSD6	-0.256486351	0.002747698
MFSD6L	0.097518228	1.65E-36
MFSD8	0.232606654	0.001174521
MFSD9	0.992138068	3.44E-32
MGA	0.681835908	3.22E-23
MGAM	0.396768794	1.75E-121
MGAM2	0.122995962	5.28E-47
MGARP	0.120512315	0.208752452
MGAT1	1.990140594	9.03E-296
MGAT2	-1.175469808	2.69E-87
MGAT3	-1.107916271	6.81E-38
MGAT4A	0.930032818	6.86E-30
MGAT4B	1.351236966	8.78E-59
MGAT4C	-0.28279208	2.93E-17
MGAT4D	-0.011207838	0.011731047
MGAT5	0.694380164	6.54E-22
MGAT5B	-0.791065437	5.87E-10
MGLL	-0.184862318	0.015506221
MGMT	-0.423482861	1.18E-20
MGP	3.082013229	1.87E-222
MGRN1	0.532824417	2.78E-10
MGST1	1.259221074	2.54E-49
MGST2	1.740241855	1.53E-205
MGST3	0.436158597	1.13E-14
MIA	-0.631564438	1.03E-26
MIA3	1.034276676	7.75E-46
MIB1	1.016775766	9.08E-78
MIB2	-0.611438622	1.89E-16
MICA	0.548419192	1.25E-20
MICAL1	-0.081640963	0.375990887
MICAL2	-1.867491398	8.07E-25
MICAL3	-1.756609081	2.77E-74
MICALL1	1.668056044	1.70E-116
MICALL2	0.27865731	5.45E-06
MICB	1.741001898	6.94E-128
MICU1	0.261642629	0.002518092
MICU2	0.969833416	2.82E-47
MICU3	-1.183278857	9.54E-33
MID1	1.421207553	9.96E-78
MID1IP1	0.336673107	1.59E-05
MID2	0.850684172	1.99E-54
MIDN	2.427865166	4.72E-246
MIEF1	1.544593934	1.52E-140
MIEF2	1.152932225	2.33E-60
MIEN1	0.107512225	0.020574075
MIER1	1.473806394	1.39E-177
MIER2	0.712532282	8.71E-47

MIER3	1.434253696	4.88E-71
MIF	-2.498543637	8.12E-161
MIF4GD	1.650469128	3.76E-246
MIGA1	-0.304638312	1.35E-05
MIGA2	0.079628526	0.339400672
MIIP	1.703741477	2.07E-159
MILR1	2.816832568	2.61E-273
MINDY1	1.373186221	1.17E-80
MINDY2	0.095027909	0.210467457
MINDY3	0.410738257	3.29E-11
MINDY4	-1.371018494	5.64E-117
MINDY4B	-0.386547327	7.77E-11
MINK1	-0.328944721	1.73E-05
MINPP1	1.533732326	7.93E-162
MIOS	1.388900019	3.68E-102
MIOX	0.201620782	1.76E-96
MIP	-0.180040454	2.52E-43
MIPEP	2.029166	1.43263050762452e-314
MIPOL1	-0.059343393	0.000225892
MIS12	1.93768569	3.04E-290
MIS18A	2.203195446	1.08E-284
MISP	-0.113461917	2.07E-06
MISP3	-0.364104024	2.57E-07
MITD1	0.508408579	1.65E-22
MITF	1.161589221	5.77E-109
MIXL1	0.351597118	3.29E-13
MKKS	0.721697144	9.85E-29
MKLN1	1.304674786	1.58E-117
MKNK1	-0.159785155	0.033803351
MKNK2	0.616585501	3.67E-11
MKRN1	1.49015796	3.14E-98
MKRN2	-0.491213493	6.72E-12
MKRN2OS	-0.773921803	1.04E-12
MKRN3	1.20779328	5.47E-119
MKS1	0.259893962	3.78E-06
MKX	-0.143124767	0.026954087
MLANA	0.17074357	5.61E-34
MLC1	1.750243159	3.12E-94
MLEC	1.909864378	5.03E-271
MLF1	1.845458942	5.67E-163
MLF2	1.245063159	1.17E-72
MLH1	0.882394739	8.91E-55
MLH3	0.611701211	6.59E-30
MLIP	0.756847694	7.87E-24
MLKL	0.182618754	0.0178749
MLLT1	0.660856084	1.70E-17
MLLT10	0.173070358	0.014933441
MLLT11	1.154135339	1.17E-20

MLLT3	0.321890528	5.12E-10
MLLT6	-0.589446175	4.41E-16
MLN	0.229245564	1.22E-59
MLNR	0.088718481	3.79E-25
MLPH	0.299435269	2.64E-22
MLST8	0.805079768	6.32E-31
MLX	1.068154829	4.47E-57
MLXIP	0.719641285	3.35E-19
MLXIPL	-1.3454928	7.11E-26
MLYCD	0.207317932	5.60E-07
MMAA	0.888919319	3.18E-110
MMAB	0.808107047	2.68E-60
MMACHC	1.274241434	1.15E-190
MMADHC	1.899674042	1.92E-178
MMD	0.653571583	3.23E-09
MMD2	-0.435928361	8.55E-08
MME	-0.116631959	0.206830024
MMEL1	0.795173994	3.60E-83
MMGT1	1.942232256	9.91E-234
MMP10	0.50160401	2.14E-137
MMP11	1.905961991	1.984853495628e-316
MMP12	0.774129554	1.59E-182
MMP13	0.76159121	2.40E-153
MMP15	1.874737259	9.52E-171
MMP16	1.980932677	3.30E-249
MMP17	-1.012054427	2.59E-19
MMP20	0.010393259	6.30E-22
MMP21	0.080364513	0.000138713
MMP23B	0.113647178	8.97E-13
MMP24	-0.282744091	0.07646957
MMP25	1.439390606	1.80E-248
MMP26	-0.017808966	0.113680584
MMP27	0.013980587	5.42E-12
MMP28	1.200312164	5.59E-37
MMP3	0.685447306	8.20E-99
MMP8	0.167427383	1.41E-09
MMRN2	1.050392267	2.42E-70
MMS19	0.124493014	0.139793896
MMS22L	0.64981634	3.37E-94
MN1	-0.233191514	0.018273145
MNAT1	0.232697757	3.49E-06
MND1	-0.355745839	2.47E-27
MNS1	1.228039054	3.56E-42
MNT	-0.009637266	0.890511164
MNX1	0.643444896	3.37E-210
MOAP1	0.003388454	0.977002494
MOB1B	0.820019578	2.86E-51
MOB2	0.051003794	0.481719448

MOB3B	0.623357798	4.97E-13
MOB3C	1.006825261	1.37E-51
MOB4	1.062948154	9.86E-63
MOBP	-3.509207695	5.60E-108
MOCOS	0.879475544	5.57E-292
MOCS1	1.111240178	1.47E-149
MOCS2	0.758767641	2.80E-40
MOG	-1.566408029	7.14E-28
MOGAT1	0.075337216	4.21E-09
MOGAT2	0.016476953	0.001963949
MOGAT3	0.019597602	3.83E-08
MOGS	1.592344193	8.60E-93
MOK	-0.25447189	4.01E-05
MON1A	0.218898628	4.03E-05
MON1B	0.774426922	2.29E-57
MON2	0.365169046	1.28E-06
MORC1	0.248593077	7.47E-51
MORC2	0.151811824	0.013860718
MORC3	0.521228218	1.30E-13
MORC4	1.881506182	3.57E-281
MORF4L1	0.722689811	1.09E-26
MORF4L2	1.219432729	6.06E-54
MORN1	-0.124318019	0.215696022
MORN2	1.046573239	2.02E-58
MORN3	0.881480989	1.66E-36
MORN4	0.143210331	0.042297857
MORN5	1.338676185	2.35E-61
MOS	-0.03311281	0.005818841
MOSPD2	1.166534514	2.18E-53
MOV10	1.726244333	1.04E-175
MOV10L1	-0.174451945	1.36E-07
MOXD1	3.008116883	2.37E-125
MPC1	1.26183777	7.50E-108
MPC1L	0.054472763	1.42E-05
MPC2	0.677231096	1.59E-25
MPDU1	1.945311422	1.82E-273
MPDZ	0.135605815	0.039198876
MPEG1	2.330238966	3.21E-262
MPG	1.436999996	1.66E-126
MPHOSPH10	0.81190706	1.51E-43
MPHOSPH6	0.975226561	3.59E-62
MPHOSPH8	-0.046119852	0.481399984
MPHOSPH9	0.862921596	6.75E-30
MPI	0.570640807	8.73E-28
MPIG6B	0.367006042	2.54E-77
MPL	0.604059281	4.09E-153
MPLKIP	1.69092513	2.30E-247
MPND	0.23038497	0.000192104

MPO	-0.431505354	4.81E-28
MPP1	0.008529328	0.92370526
MPP2	0.025809858	0.752961823
MPP3	-0.541521648	0.000229692
MPP4	-0.189299419	0.001216474
MPP5	0.051012811	0.35801069
MPP6	0.851850101	3.67E-42
MPP7	-1.079545815	8.25E-36
MPPE1	0.718855132	3.50E-42
MPPED1	-1.495183346	1.58E-22
MPPED2	0.601485802	2.36E-21
MPRIP	-0.102552744	0.116895546
MPST	0.699775633	4.99E-39
MPV17	1.3490952	6.46E-137
MPV17L	1.203879221	9.20E-115
MPV17L2	1.522188551	1.97E-165
MPZ	0.783612919	1.24E-22
MPZL2	1.052271118	7.91E-40
MPZL3	0.410385971	2.29E-07
MRAP	0.119144105	3.24E-21
MRAP2	-0.313397714	0.009140445
MRAS	0.349007248	6.67E-09
MRC1	1.792976557	1.26E-202
MRE11	1.008714183	2.04E-62
MREG	1.98830448	4.66E-68
MRFAP1	0.842569552	1.24E-49
MRFAP1L1	0.658956143	3.53E-27
MRGBP	1.538068011	1.50E-140
MRGPRD	-0.155938793	1.20E-06
MRGPRE	0.151013855	5.17E-05
MRGPRF	0.436295994	5.38E-05
MRGPRG	-0.001063891	0.65663303
MRGPRX1	-0.001989469	0.276657429
MRGPRX2	0.001528886	0.280761407
MRGPRX3	0.172850639	1.66E-119
MRGPRX4	0.032456166	7.78E-08
MRI1	0.472248286	2.94E-09
MRLN	-0.797176391	7.34E-10
MRM1	1.166952806	4.21E-127
MRM3	1.167862391	1.04E-86
MRNIP	-0.395214455	0.000166244
MRO	-0.287540687	0.000370054
MROH1	-0.691770725	3.53E-14
MROH2A	-0.004233525	0.315294053
MROH2B	0.012972947	2.44E-14
MROH6	0.153331539	0.104531681
MROH7	-2.439729514	1.83E-221
MROH8	0.451535929	1.75E-17

MROH9	0.052838685	1.06E-106
MRPL1	1.630106785	1.42E-172
MRPL10	1.242664198	3.14E-89
MRPL12	-1.089787088	7.01E-85
MRPL13	1.397816252	7.58E-161
MRPL15	1.72960584	1.29E-164
MRPL16	0.925262488	2.83E-71
MRPL17	1.602548646	2.70E-163
MRPL18	1.505140127	8.30E-149
MRPL19	0.760333976	1.63E-67
MRPL2	-1.975723861	1.18E-199
MRPL20	0.719413734	3.70E-42
MRPL21	1.395071282	9.77E-104
MRPL22	1.037169042	1.96E-98
MRPL23	1.107501776	1.67E-83
MRPL27	1.411766345	4.76E-163
MRPL28	0.294009872	4.05E-07
MRPL3	1.736597793	5.10E-154
MRPL30	1.010062151	5.07E-56
MRPL32	1.814529202	5.36E-168
MRPL33	1.188961557	1.85E-81
MRPL34	1.24250688	1.60E-103
MRPL35	1.016230031	5.14E-64
MRPL37	2.085381548	1.89E-195
MRPL39	2.017843572	1.42E-231
MRPL4	0.60280672	3.50E-20
MRPL40	1.839339485	9.64E-288
MRPL41	0.438285993	1.30E-14
MRPL42	1.549000398	7.10E-178
MRPL43	0.343302386	1.40E-10
MRPL44	1.878189089	1.81E-182
MRPL45	1.975976227	5.44E-190
MRPL46	-2.279030682	4.26E-264
MRPL47	1.947545302	6.16E-250
MRPL48	1.068858458	9.32E-72
MRPL49	1.608900567	1.61E-156
MRPL50	1.531328574	1.14E-140
MRPL51	1.628116885	1.85E-199
MRPL52	0.658416161	4.69E-45
MRPL53	-2.307107531	1.70E-178
MRPL54	2.194108836	4.04E-306
MRPL55	1.462460743	6.35E-126
MRPL57	1.592415324	1.42E-178
MRPL58	1.944770427	4.25E-197
MRPL9	0.572937774	5.72E-18
MRPS10	1.867812887	1.16E-189
MRPS11	0.925635691	1.24E-68
MRPS12	2.001092564	1.04E-243

MRPS14	1.547718429	6.22E-221
MRPS15	1.610324142	4.49E-225
MRPS16	1.567607685	6.87E-148
MRPS17	1.12717964	3.94E-67
MRPS18A	0.794887417	2.07E-47
MRPS18B	1.813787727	3.40E-206
MRPS18C	-0.455175567	4.69E-18
MRPS2	1.072457315	2.17E-79
MRPS21	1.539252529	1.84E-139
MRPS22	0.384365146	2.19E-13
MRPS23	1.728945214	4.93E-166
MRPS25	-0.2967876	0.000123101
MRPS26	1.709839719	1.96E-189
MRPS27	1.22336479	6.67E-79
MRPS28	-0.483188617	6.62E-24
MRPS30	1.278636457	1.99E-85
MRPS31	1.142750934	2.30E-97
MRPS33	2.004225064	6.35E-260
MRPS34	1.374434084	4.81E-99
MRPS35	2.014334444	5.07E-198
MRPS36	1.223606979	6.59E-117
MRPS5	-1.095430031	5.59E-49
MRPS6	0.557461534	1.68E-22
MRPS7	1.638980424	2.29E-166
MRPS9	0.022550746	0.712194171
MRRF	0.965048465	1.47E-88
MRS2	1.134184657	4.73E-52
MRTO4	2.251739633	4.53E-267
MS4A1	0.026127251	0.065470132
MS4A10	0.034478969	2.50E-54
MS4A12	-0.001602155	0.528543171
MS4A13	0.004410411	0.000434718
MS4A14	0.781596894	4.92E-40
MS4A15	0.057336238	2.18E-13
MS4A18	0.004709027	0.005034471
MS4A2	0.035449323	2.16E-29
MS4A3	0.06717606	1.20E-11
MS4A5	-0.003601951	0.077634527
MS4A6A	3.877093969	2.55E-274
MS4A6E	0.354265231	1.74E-110
MS4A7	3.249267687	1.53E-234
MS4A8	-0.085082171	0.004331617
MSANTD1	-0.464198606	2.81E-27
MSANTD2	0.568326817	3.22E-18
MSANTD3	1.651440508	1.02E-209
MSANTD4	1.549582726	5.49E-166
MSC	0.858090827	5.98E-23
MSGN1	0.011162976	0.004003726

MSH2	0.090110143	0.237923975
MSH3	1.383925507	2.12E-166
MSH4	-0.253828121	6.38E-07
MSH5	-0.24657895	2.66E-07
MSH6	1.5622807	1.31E-104
MSI2	0.258207995	1.51E-05
MSL1	0.314933594	3.83E-05
MSL2	1.298874635	6.57E-95
MSL3	0.549316239	5.80E-22
MSLN	0.986239807	4.56E-86
MSMB	-0.170474643	1.53E-07
MSMO1	1.363590626	1.48E-58
MSRA	1.424480381	8.25E-72
MSRB2	0.506986537	1.35E-31
MSRB3	-0.012908637	0.772791786
MSS51	-0.343625323	5.38E-05
MST1	-0.223250986	0.000920153
MST1R	-0.130013546	0.000182222
MSTO1	1.194010623	1.40E-83
MSX1	2.631892096	1.40E-295
MSX2	0.193048352	0.035518649
MT1A	0.359214579	0.000617015
MT1B	-0.059544137	1.30E-05
MT1E	0.990979198	2.50E-33
MT1F	0.142476514	0.043723911
MT1G	-0.068646574	0.635374346
MT1H	0.530422096	3.81E-05
MT1HL1	0.38779602	2.38E-160
MT1M	1.620349704	2.04E-63
MT1X	-0.418834843	1.50E-05
MT2A	3.317605261	4.76E-240
MT3	0.370135167	5.73E-09
MT4	0.059611779	0.000682881
MTA1	-0.156429122	0.037406886
MTA2	1.566909587	3.94E-109
MTA3	0.076654476	0.247629991
MTAP	0.544901583	1.76E-44
MTBP	0.214275662	0.017354325
MTCH1	1.055393344	2.18E-50
MTCH2	2.045961685	4.04E-227
MTCL1	-0.53206111	0.004296548
MTCP1	-1.591878963	1.74E-302
MTDH	1.797651401	2.14E-152
MTERF1	1.736001873	1.24E-271
MTERF2	-0.10552371	0.173482854
MTERF3	1.749984893	2.11E-184
MTERF4	-0.166078392	0.039461492
MTF1	0.977049599	5.62E-73

MTF2	0.648085384	1.26E-19
MTFMT	1.288761668	4.96E-101
MTFP1	-1.476730962	4.83E-174
MTFR1L	-0.027696603	0.628323731
MTG1	-2.727997938	8.64E-225
MTG2	1.18295117	2.07E-55
MTHFD1	1.184350865	1.28E-85
MTHFD1L	1.766488657	1.75E-74
MTHFD2	1.538580355	3.25E-69
MTHFD2L	1.161527822	1.82E-204
MTHFR	0.218554134	0.000792148
MTHFSD	1.265929521	1.14E-177
MTIF2	1.495969114	3.42E-182
MTIF3	0.335192991	2.16E-11
MTM1	1.38547311	6.73E-154
MTMR1	0.795352305	5.19E-29
MTMR10	-0.117250969	0.104947097
MTMR11	1.802912822	3.44E-181
MTMR12	0.939659668	5.00E-47
MTMR14	0.996758819	1.95E-55
MTMR2	0.39201783	6.38E-10
MTMR3	-1.121197777	6.39E-108
MTMR4	0.620080145	4.90E-11
MTMR6	1.158733389	7.51E-70
MTMR7	-1.481722793	1.90E-69
MTMR8	0.183821893	2.71E-24
MTMR9	0.147994175	0.038172883
MTNR1A	-0.161419416	4.51E-05
MTNR1B	0.061263904	5.14E-08
MTO1	0.690611832	2.65E-38
MTOR	0.942147906	1.93E-39
MTPAP	0.524708851	1.19E-26
MTPN	2.382416892	3.23E-211
MTR	0.449924768	6.54E-11
MTRF1	0.956391538	2.74E-72
MTRF1L	0.148592471	0.003301429
MTRNR2L1	0.795969273	8.42E-44
MTRNR2L10	0.694901699	2.08E-99
MTRNR2L3	0.363507095	3.88E-209
MTRNR2L4	0.047100398	0.024046213
MTRNR2L5	0.032001812	1.62E-17
MTRNR2L6	0.374877391	8.54E-40
MTRNR2L7	0.012966289	5.57E-14
MTRNR2L8	0.474475526	2.97E-10
MTRR	0.931230178	5.40E-49
MTSS1	0.044336936	0.619293516
MTURN	-2.523764725	6.87E-158
MTUS1	-0.220101518	0.012664018

MTUS2	-1.702249564	7.54E-77
MTX1	1.516539011	3.18E-216
MTX2	0.880995029	9.63E-37
MTX3	0.691647066	4.04E-25
MUC12	0.171507929	3.21E-36
MUC13	0.128291933	6.55E-26
MUC15	-0.038156513	6.76E-07
MUC16	0.00088021	0.751279145
MUC17	-0.004825127	0.099087324
MUC19	0.003178906	0.316911149
MUC2	-0.02821654	0.020591028
MUC20	-0.10560601	0.116777157
MUC21	-0.094772811	6.53E-09
MUC22	0.027223134	2.96E-29
MUC3A	0.094480185	5.69E-05
MUC4	0.036975375	0.038210992
MUC5AC	-0.018620568	0.042594563
MUC5B	-0.043266401	2.29E-05
MUC6	-0.436495648	8.40E-17
MUC7	-0.472185587	7.12E-12
MUCL1	-0.202104152	2.09E-14
MUS81	0.59950843	2.29E-20
MUSK	-0.002913286	0.907545633
MUTYH	-2.761206813	1.33E-215
MVB12B	0.45899412	4.97E-10
MVD	-0.343120711	3.17E-06
MVK	-0.171789159	0.006949377
MVP	1.521073666	3.42E-97
MX1	1.121910058	1.52E-44
MX2	1.012595769	1.21E-60
MXD1	0.71537019	6.16E-20
MXD3	1.590473821	4.06E-184
MXD4	0.256036167	0.00785354
MXI1	-0.456456976	1.28E-18
MXRA7	0.99471878	2.25E-48
MXRA8	2.123062546	6.95E-290
MYADM	1.559151308	8.91E-97
MYADML2	-1.061338826	1.29E-49
MYB	0.330588007	5.85E-09
MYBBP1A	0.822850128	1.01E-23
MYBL1	1.321076459	4.91E-154
MYBPC1	-0.120212323	0.220830354
MYBPC2	0.015377171	0.69872977
MYBPC3	-0.086928245	0.001445835
MYBPH	2.299115898	1.25E-207
MYBPHL	0.611255175	2.00E-29
MYCBP	1.8204962	3.35E-227
MYCBP2	-0.575551685	4.23E-10

MYCBPAP	0.146258256	0.003321615
MYCN	1.888064448	2.17E-181
MYCT1	1.224861702	5.06E-108
MYD88	2.689169378	1.18E-280
MYEF2	0.403746488	1.19E-07
MYEOV	0.13264823	2.91E-11
MYF5	0.00222404	0.129019196
MYF6	-0.105914277	1.75E-05
MYH1	-0.301981422	2.95E-15
MYH10	0.803412045	6.11E-19
MYH11	-1.738997624	1.94E-79
MYH13	-0.010522742	0.037058995
MYH14	-1.335922408	4.10E-96
MYH15	-0.481461702	5.66E-41
MYH2	-0.246155162	8.98E-13
MYH3	-0.284900689	1.89E-06
MYH4	0.006306923	0.208226825
MYH6	-0.408541267	5.93E-23
MYH7	0.625148661	1.03E-16
MYH7B	-1.776646423	4.94E-163
MYH8	0.013135028	0.007693272
MYH9	1.721344034	1.42E-151
MYL1	-0.451623234	2.70E-20
MYL10	0.103530695	2.58E-40
MYL12A	2.066628167	1.47E-235
MYL12B	2.140024077	1.20E-250
MYL2	-1.604133785	8.34E-59
MYL3	-0.87903006	2.56E-54
MYL4	0.505141064	3.20E-70
MYL5	0.145314984	0.020012917
MYL6	2.078904659	8.15E-261
MYL6B	0.009393649	0.886753798
MYL7	-0.883797323	3.53E-46
MYL9	2.090840747	1.78E-174
MYLIP	0.658943401	6.00E-32
MYLK	0.109021798	0.259537167
MYLK2	1.554054014	1.42E-279
MYLK3	-0.184540005	0.009272832
MYLK4	0.091944567	0.025754117
MYLPF	0.103692167	0.019276406
MYNN	1.58839456	4.80E-177
MYO10	1.390250116	7.63E-78
MYO15A	-0.759131627	6.04E-27
MYO15B	-1.081559933	4.19E-52
MYO16	0.883762017	1.58E-61
MYO18A	-0.25064257	0.000457299
MYO18B	0.046370345	5.85E-06
MYO19	0.37395733	2.18E-07

MYO1A	-0.1467644	0.049829837
MYO1B	1.574768223	1.53E-137
MYO1C	1.5821533	4.04E-120
MYO1D	0.090419282	0.291778246
MYO1E	1.931119027	2.15E-125
MYO1F	2.19726829	1.48E-173
MYO1G	1.216913508	2.48E-92
MYO1H	0.063404573	4.55E-13
MYO3A	-0.237746748	2.53E-10
MYO3B	0.183893595	3.82E-25
MYO5A	-0.529317201	9.64E-08
MYO5B	1.034741081	5.60E-114
MYO5C	0.04608553	0.711124483
MYO6	0.310292771	4.69E-09
MYO7A	1.345219045	2.44E-124
MYO7B	0.623019865	1.43E-79
MYO9A	0.092820313	0.132299533
MYO9B	1.295014819	5.97E-52
MYOC	-0.081710774	7.92E-05
MYOCD	-0.138127292	6.65E-11
MYOD1	0.020906651	0.084503176
MYOF	2.572265696	1.59E-300
MYOG	0.167770374	3.03E-10
MYOM1	-0.993586199	3.31E-83
MYOM2	0.37557759	4.40E-08
MYOM3	0.01149415	0.348679374
MYOT	-1.696868708	2.86E-140
MYOZ1	0.822050288	4.10E-61
MYOZ2	0.459192008	1.32E-55
MYOZ3	0.157700711	0.013559166
MYPN	-0.009089426	0.420708367
MYPOP	0.001569821	0.986944609
MYRF	-1.573191159	8.72E-35
MYRFL	0.303517374	3.22E-12
MYRIP	-0.881831358	1.29E-18
MYSM1	-0.230969288	0.003908575
MYT1	-0.433048626	0.000517131
MYT1L	-2.845521856	3.33E-116
MYZAP	-0.313159574	0.012303417
MZB1	0.47422618	1.64E-79
MZF1	-1.026286732	1.57E-39
MZT1	0.581757374	9.96E-12
MZT2A	-0.83449832	6.55E-48
MZT2B	-1.29454813	8.79E-134
N4BP1	0.89947923	3.58E-39
N4BP2	1.613900779	2.4730406496019e-318
N4BP2L1	-0.496396274	1.95E-12
N4BP2L2	0.267450826	3.01E-07

N4BP3	-0.505515712	8.19E-09
N6AMT1	0.933841221	9.58E-70
NAA10	-0.654694481	8.14E-33
NAA11	0.129106971	1.59E-64
NAA15	1.564144299	9.46E-169
NAA16	0.695850161	2.24E-17
NAA20	1.869903938	1.90E-174
NAA25	0.142889816	0.056877417
NAA30	0.882040176	1.70E-50
NAA35	1.135756104	5.69E-94
NAA38	1.040297059	4.32E-86
NAA40	1.06216893	8.99E-33
NAA50	1.870241255	1.65E-182
NAA60	0.505914612	7.68E-19
NAAA	0.926373969	8.04E-50
NAALAD2	-0.962097735	4.39E-57
NAALADL1	0.724481026	2.40E-72
NAB1	0.929791912	1.41E-31
NAB2	1.252343053	1.39E-33
NABP1	0.837965943	2.03E-124
NABP2	1.615744634	3.77E-166
NACA	0.463729342	8.13E-16
NACAD	0.441959169	4.35E-08
NACC1	0.98014122	3.13E-68
NACC2	0.254218182	1.63E-06
NADK	1.787003579	2.21E-199
NADK2	1.174669572	5.41E-120
NADSYN1	0.249268333	0.000202162
NAE1	0.953113594	2.16E-42
NAF1	0.925207745	2.77E-68
NAGK	1.083161721	4.34E-67
NAGPA	-0.46958858	1.47E-08
NAGS	1.03359896	1.20E-94
NAIF1	1.560695747	1.18E-218
NAIP	0.403491735	4.16E-74
NALCN	-1.608133546	1.08E-106
NAMPT	3.120875373	4.73E-278
NANOG	0.014534076	5.08E-10
NANOGNB	0.0073636	4.41E-06
NANOGP8	0.019169647	0.095286237
NANOS1	0.328342389	9.37E-12
NANOS2	0.043234742	0.020369033
NANOS3	1.022678565	3.46E-40
NANP	2.080421744	9.78E-305
NANS	1.519879949	8.62E-164
NAP1L1	0.958296901	3.91E-74
NAP1L2	-1.901290921	1.11E-58
NAP1L3	-0.786136994	2.50E-12

NAP1L4	0.568714641	8.46E-33
NAP1L5	-0.003685428	0.975823524
NAPA	-0.549276259	3.40E-08
NAPB	-1.92140166	1.13E-53
NAPEPLD	0.358684299	1.26E-06
NAPG	-0.080847423	0.286762614
NAPRT	-0.290405611	7.71E-05
NAPSA	-0.568723872	3.32E-52
NARF	0.854567649	4.36E-30
NARS2	1.537543449	5.15E-141
NASP	1.042238844	2.72E-59
NAT10	1.520929034	2.85E-115
NAT14	1.155298144	3.52E-58
NAT16	1.056543594	6.02E-61
NAT2	0.816618561	9.28E-209
NAT8	-0.140702482	7.61E-10
NAT8L	-0.616761589	2.99E-13
NAT9	0.673894739	2.97E-23
NATD1	1.125241174	6.23E-62
NAV1	1.22695318	1.45E-111
NAV2	0.883640654	3.45E-33
NAV3	-0.757651934	9.67E-26
NAXD	0.561886188	4.28E-21
NBAS	1.140436677	4.63E-73
NBDY	1.120171779	5.50E-83
NBEA	-0.371597885	0.0006052
NBEAL1	0.205479613	7.34E-11
NBEAL2	0.07157513	0.361494903
NBL1	-0.494062501	4.34E-14
NBN	1.76239094	1.51E-192
NBPF1	0.904554535	3.80E-36
NBPF10	0.501428637	2.10E-238
NBPF11	0.964716202	2.86E-70
NBPF12	0.333109899	9.84E-08
NBPF14	0.800128965	9.99E-204
NBPF15	0.917414579	9.84E-17
NBPF19	-0.799880071	4.08E-130
NBPF20	-0.022559882	0.744656031
NBPF26	0.522583473	3.56E-20
NBPF3	1.06301804	2.01E-67
NBPF4	-0.014652704	0.091947136
NBPF6	-0.017042896	0.016923638
NBPF9	0.725336203	3.75E-16
NBR1	0.467927985	1.77E-17
NCALD	1.246963811	4.45E-19
NCAM1	0.57658336	1.74E-20
NCAM2	0.645126335	7.21E-18
NCAN	2.399289413	2.23E-96

NCAPD3	1.45510085	1.17E-121
NCAPH2	0.737343785	5.15E-22
NCBP1	1.649670659	2.40E-154
NCBP2	1.136081019	1.21E-107
NCBP2L	-0.06241626	2.38E-06
NCBP3	0.59628211	8.70E-22
NCCRP1	-0.036678296	0.146428059
NCDN	-1.890675494	2.23E-48
NCEH1	0.421385818	1.16E-05
NCF1	1.788722624	5.13E-240
NCF2	3.130965027	1.37844315189708e-321
NCF4	2.395256596	2.28E-194
NCK1	1.255221753	1.87E-113
NCK2	1.3054768	2.72E-67
NCKAP1	1.148607608	3.17E-58
NCKAP1L	2.731849402	9.93E-291
NCKAP5	1.066534457	2.22E-140
NCKAP5L	0.580690192	5.04E-20
NCKIPSD	0.38512092	3.02E-06
NCL	0.715797218	4.37E-32
NCLN	1.51139179	3.44E-87
NCMAP	0.946365879	1.20E-99
NCOA1	0.650692806	2.92E-26
NCOA2	0.65057714	3.66E-26
NCOA3	1.413003312	4.51E-104
NCOA4	1.724576441	5.62E-202
NCOA5	1.707448835	1.10E-99
NCOA6	1.043415961	2.44E-42
NCOA7	-0.366027876	4.87E-05
NCOR1	0.565981085	3.07E-19
NCOR2	1.029882874	1.99E-37
NCR1	0.077250797	4.78E-41
NCR2	0.011506885	2.70E-08
NCR3	0.281549734	9.14E-74
NCR3LG1	-0.492845659	8.81E-07
NCS1	-1.393763095	5.34E-38
NDE1	1.661769538	6.47E-97
NDEL1	-0.297549753	0.000388407
NDFIP1	1.183434585	8.34E-45
NDFIP2	0.971425454	4.86E-33
NDN	0.183010581	0.099180296
NDNF	1.444465666	7.62E-161
NDOR1	1.273207969	5.24E-71
NDP	1.392108831	5.36E-101
NDRG1	-0.319903511	0.001366386
NDRG2	-1.288304696	4.06E-123
NDRG3	0.023415048	0.832488297
NDRG4	-1.357814543	1.13E-34

NDST1	1.269081041	1.90E-98
NDST2	-3.315194962	8.24E-287
NDST3	-1.454329698	6.81E-48
NDST4	0.128856257	1.71E-14
NDUFA1	1.258611004	1.55E-106
NDUFA10	-0.795283576	1.04E-22
NDUFA11	-1.523308154	3.53E-144
NDUFA12	1.474761118	3.69E-152
NDUFA2	0.568756082	4.10E-30
NDUFA3	1.210883525	7.33E-111
NDUFA4	0.823205985	2.98E-31
NDUFA4L2	1.632708481	1.08E-187
NDUFA5	-0.279939318	1.04E-05
NDUFA6	1.673594804	8.42E-179
NDUFA8	1.15543306	3.40E-80
NDUFA9	-0.67898697	5.18E-28
NDUFAB1	1.474723048	9.01E-98
NDUFAF1	0.239621299	1.00E-05
NDUFAF2	-0.969742934	1.38E-64
NDUFAF3	0.933672731	8.10E-75
NDUFAF4	0.605425859	6.60E-16
NDUFAF5	0.779668803	2.60E-33
NDUFAF6	1.243992953	1.98E-145
NDUFAF7	0.362457509	3.00E-10
NDUFAF8	1.108600472	2.59E-104
NDUFB1	0.394700214	2.12E-11
NDUFB10	1.118230631	5.22E-95
NDUFB11	1.702388251	1.08E-178
NDUFB2	0.644943844	1.71E-32
NDUFB3	1.671098841	2.84E-183
NDUFB4	1.195796302	1.29E-86
NDUFB5	1.131060758	4.34E-78
NDUFB6	1.516446117	1.89E-137
NDUFB7	1.867497173	1.60E-239
NDUFC1	0.379160716	2.27E-13
NDUFC2	-0.011272774	0.873486082
NDUFS1	1.013046452	8.51E-59
NDUFS2	-0.148934232	0.060117339
NDUFS3	0.577439208	4.06E-22
NDUFS4	1.405249705	4.15E-116
NDUFS5	1.441983816	1.12E-116
NDUFS7	-2.737033461	2.16931266241067e-315
NDUFS8	0.34922819	2.04E-11
NDUFV1	-0.153923059	0.029794899
NDUFV3	1.425144759	1.07E-194
NEB	0.003218098	0.931608426
NEBL	-1.564813803	5.30E-134
NECAB1	-1.093641475	5.87E-16

NECAB2	-1.552128957	3.14E-22
NECAB3	0.1105608	0.139350857
NECAP1	-0.840285417	2.13E-16
NECAP2	1.875563706	3.47E-279
NECTIN1	-0.106898847	0.23113294
NECTIN3	1.22456857	1.98E-82
NECTIN4	0.352244739	1.30E-107
NEDD4L	-1.148861399	4.65E-39
NEDD8	-0.388409252	1.44E-10
NEFH	-2.44345585	6.82E-60
NEFL	-2.830903509	2.56E-62
NEFM	-4.591012723	2.84E-188
NEGR1	-1.596923724	9.31E-66
NEIL1	-1.051480379	3.97E-28
NEIL2	1.093262381	1.15E-95
NEK1	0.522928622	3.18E-22
NEK10	-0.228400182	9.94E-06
NEK11	1.473361607	3.68E-124
NEK3	0.319180235	1.44E-06
NEK4	0.821404885	2.66E-45
NEK5	0.162217572	0.003328517
NEK6	2.760902368	1.21E-264
NEK7	0.74789578	5.58E-29
NEK8	0.935745622	1.15E-101
NEK9	0.191746359	0.005667491
NELFA	1.21424768	2.18E-68
NELFB	1.602169413	3.00E-176
NELFCD	0.538527331	1.61E-17
NELFE	1.848743364	3.27E-271
NELL1	-0.465099398	1.33E-06
NELL2	0.218204307	0.133526712
NEMF	-0.406311046	2.18E-09
NEMP1	2.35998026	5.19E-262
NEMP2	0.647598415	1.64E-33
NENF	1.536541391	1.69E-150
NEO1	1.073152043	1.68E-85
NEPRO	1.151567273	4.61E-76
NET1	-0.470828298	6.24E-15
NETO1	-0.410133792	8.09E-05
NETO2	1.331487156	1.19E-48
NEU1	1.37233676	3.35E-73
NEU2	-0.00762101	0.024298722
NEU3	0.609263871	2.01E-21
NEU4	0.89194624	4.67E-54
NEURL1	-2.869391953	9.83E-122
NEURL1B	1.978531146	8.03E-105
NEURL2	-0.356761712	1.82E-10
NEURL4	-0.610366172	5.66E-12

NEUROD1	-0.057090136	0.816656545
NEUROD2	-2.159003309	4.36E-21
NEUROD4	0.447753349	8.21E-138
NEUROD6	-1.695051544	1.22E-21
NEUROG1	0.006627097	0.367201946
NEUROG2	-0.55338406	1.23E-13
NEUROG3	0.087718266	0.044386834
NEXN	1.29333822	1.34E-153
NF1	0.272191931	1.03E-05
NF2	0.437256267	1.17E-14
NFASC	0.071788484	0.357495694
NFAT5	0.156730544	0.035587996
NFATC1	1.369582319	4.31E-212
NFATC2	1.725944416	6.70E-204
NFATC2IP	0.766868428	1.00E-16
NFATC3	1.038206918	2.42E-78
NFATC4	0.496618771	2.20E-14
NFE2	0.6543764	1.71E-116
NFE2L1	1.073830115	3.54E-70
NFE2L2	1.593860022	4.63E-165
NFE2L3	0.747657321	1.70E-15
NFIA	-0.553304973	3.46E-15
NFIB	1.941568788	1.14E-208
NFIC	1.540857156	5.86E-96
NFIL3	2.34058245	3.87E-138
NFIX	1.792762145	5.16E-87
NFKB1	1.788477532	5.95E-163
NFKB2	1.63158082	1.56E-129
NFKBIA	1.849380242	1.75E-176
NFKBIB	1.257027875	3.45E-160
NFKBID	0.396098105	7.23E-07
NFKBIE	2.003121267	2.98E-158
NFKBIL1	0.955571232	3.17E-60
NFKBIZ	1.281954059	8.22E-90
NFRKB	1.198364973	7.71E-51
NFS1	-0.109705775	0.054073005
NFU1	1.920070257	8.04E-210
NFX1	0.727883434	6.31E-24
NFXL1	0.759503709	1.65E-35
NFYA	0.970001748	1.10E-70
NFYB	0.224696854	3.03E-06
NFYC	0.935386887	1.82E-43
NGB	-2.108040997	5.88E-54
NGDN	0.922613914	4.14E-42
NGEF	-0.970972443	1.33E-06
NGF	0.191844889	3.54E-05
NGFR	2.41644441	5.70E-75
NGLY1	0.44706532	6.00E-10

NGRN	-1.238960881	5.64E-86
NHEJ1	-0.819023539	7.99E-40
NHLH1	0.911295797	2.78E-49
NHLH2	-0.6741987	5.25E-15
NHLRC1	0.10389625	0.064190604
NHLRC2	0.614713951	2.16E-28
NHLRC3	0.472125559	1.48E-13
NHLRC4	0.13334782	0.03569611
NHP2	0.732538757	1.80E-36
NHS	0.192140776	6.45E-05
NHSL1	1.690795478	1.14E-225
NHSL2	-1.009591125	1.91E-23
NIF3L1	1.911743211	1.27E-152
NIFK	1.796859804	3.50E-227
NIM1K	1.517787575	1.43E-144
NIN	0.602646923	8.59E-22
NINJ2	0.408726818	5.70E-05
NINL	-0.504601031	3.57E-08
NIPA1	-0.63410414	9.01E-20
NIPA2	1.10916555	2.37E-59
NIPAL1	-0.154048136	5.37E-09
NIPAL2	-0.351266297	0.000228969
NIPAL3	-0.716753955	3.51E-17
NIPAL4	0.221243823	0.017864635
NIPBL	0.902975954	6.97E-47
NIPSNAP1	1.233200213	1.93E-51
NIPSNAP3A	1.647611398	2.34E-138
NIPSNAP3B	-0.877573763	2.04E-46
NISCH	-0.167158222	0.177501313
NIT1	1.244056494	4.51E-100
NIT2	1.216857164	1.18E-95
NKAIN1	-0.186452031	0.139721786
NKAIN2	-2.182695808	1.31E-120
NKAIN4	1.843843502	2.97E-106
NKAP	0.934726057	1.58E-90
NKAPL	-0.756881762	2.23E-62
NKD1	0.725315602	3.50E-40
NKD2	0.323074805	3.42E-06
NKG7	1.892905745	3.71E-201
NKIRAS1	-0.227229644	0.003201524
NKIRAS2	1.985349235	1.16E-272
NKPD1	-0.006333172	0.855146845
NKRF	-0.101175806	0.190462849
NKTR	-1.121394841	3.61E-31
NKX1-1	0.025931978	7.24E-05
NKX1-2	0.005779654	0.847688359
NKX2-1	-0.415063778	2.71E-06
NKX2-2	0.795114858	1.93E-24

NKX2-3	0.039138073	1.92E-10
NKX2-4	0.013647947	0.597184333
NKX2-6	0.020955048	3.39E-07
NKX2-8	-0.304705277	1.22E-05
NKX3-1	0.742557619	1.61E-80
NKX6-1	0.86668881	8.16E-87
NKX6-2	-1.683790063	1.26E-41
NKX6-3	-0.989141575	1.28E-19
NLE1	1.170727025	5.83E-73
NLGN1	1.946670444	8.03E-268
NLGN2	0.60091322	7.75E-13
NLGN3	1.976010355	8.60E-211
NLGN4X	2.023192225	2.09E-181
NLGN4Y	0.852624912	5.93E-19
NLK	0.594950626	1.88E-12
NLN	1.868007019	7.96E-229
NLRC3	0.356132346	3.85E-13
NLRC4	1.281570054	3.85E-228
NLRC5	1.867946794	2.20E-229
NLRP10	0.00487709	0.00684104
NLRP11	0.397444438	1.25E-207
NLRP12	0.342372483	2.70E-200
NLRP13	-0.000564805	0.676735903
NLRP14	0.146217631	1.02E-28
NLRP2	-0.298077616	2.77E-07
NLRP2B	-0.059470114	0.0006406
NLRP3	1.368943213	1.22E-135
NLRP4	0.301583209	3.75E-68
NLRP5	0.006218547	1.35E-05
NLRP6	0.222342517	2.41E-41
NLRP7	0.009937331	0.000129786
NLRP8	0.002345889	0.092277246
NLRP9	0.025113368	1.90E-06
NLRX1	1.449873093	6.81E-182
NMBR	0.168581061	1.65E-10
NME1	0.293515482	0.000103983
NME3	0.326107748	1.10E-07
NME4	1.445386802	6.41E-206
NME5	0.1816711	0.020964254
NME6	1.417690448	9.09E-133
NME7	0.648510301	4.95E-31
NME8	0.21793004	5.85E-15
NME9	-0.064065144	0.239489067
NMI	2.414128228	5.53353523342196e-322
NMNAT2	-1.746118653	4.34E-30
NMNAT3	-0.240073316	6.57E-10
NMRAL1	1.493012694	7.56E-144
NMRK1	0.315903411	9.58E-05

NMRK2	-0.330520617	6.89E-20
NMS	-0.058673399	0.055750744
NMT1	0.752074778	2.42E-28
NMT2	-0.180400486	0.012455048
NMU	1.921130837	8.37E-186
NMUR1	0.134707128	0.001511405
NMUR2	0.086240149	0.009348037
NNAT	-1.014025788	8.86E-10
NNT	0.753448681	2.31E-31
NOA1	2.095014802	7.98E-200
NOB1	1.195892101	5.42E-81
NOBOX	0.004710072	0.014067956
NOC2L	0.577802121	1.32E-12
NOC3L	1.624389138	2.31E-246
NOC4L	1.61491403	7.52E-121
NOCT	1.650369789	7.18E-188
NOD1	0.933066001	8.43E-102
NOD2	0.911684177	1.32E-155
NODAL	1.188961026	4.45E-95
NOG	0.525191759	1.55E-12
NOL10	1.537520449	2.54E-197
NOL11	1.638218621	9.62E-107
NOL12	-1.998067616	3.70E-139
NOL3	0.51561807	3.81E-22
NOL4	0.018910517	0.836374755
NOL4L	0.360572769	6.25E-06
NOL6	1.514915989	8.70E-86
NOL7	0.970488295	2.51E-73
NOL8	1.117874049	5.86E-100
NOL9	1.115463374	2.86E-74
NOLC1	0.981629421	3.00E-41
NOM1	1.355862534	1.99E-120
NOMO1	1.03050705	1.88E-43
NOMO2	1.184975984	8.43E-57
NOMO3	0.378225071	8.01E-11
NONO	1.460140447	2.58E-79
NOP14	1.25613665	4.20E-44
NOP16	1.248107576	1.91E-103
NOP56	1.665623821	2.20E-94
NOP58	0.403938463	3.55E-08
NOP9	0.417165351	9.87E-13
NOS1	-1.350403855	1.23E-85
NOS1AP	-0.957867009	1.14E-17
NOS2	1.758041154	2.71E-113
NOS3	0.147546914	0.029501239
NOSIP	0.82074733	1.87E-59
NOSTRIN	0.305736201	7.36E-09
NOTCH1	2.469772767	3.19448136918862e-312

NOTCH2	2.253147165	7.98E-207
NOTCH3	2.314692434	9.28E-247
NOTCH4	0.714668292	1.49E-45
NOTO	-0.001942373	0.747138308
NOTUM	-0.045538009	0.2476721
NOVA1	1.605352871	1.61E-75
NOVA2	0.644061694	1.67E-22
NOX1	0.939652833	2.16E-289
NOX3	0.17934472	2.00E-89
NOX5	-0.237614629	5.60E-26
NOXA1	-0.659600474	3.66E-16
NOXO1	-0.715235079	7.56E-23
NOXRED1	0.732483937	2.56E-146
NPAP1	-0.2394745	2.14E-21
NPAS1	0.312702023	0.001827504
NPAS2	-1.456739895	8.21E-75
NPAS3	1.660347461	2.69E-198
NPAS4	-0.819131793	1.15E-09
NPAT	1.359777115	9.16E-102
NPBWR1	-0.079331377	0.000106589
NPBWR2	-0.074847087	0.000445273
NPC1	1.156618221	6.65E-54
NPC1L1	0.085602827	0.025527904
NPC2	3.140847235	4.66E-275
NPDC1	-0.514340752	5.44E-09
NPEPL1	-2.747538155	1.74E-192
NPEPPS	-0.103436112	0.19322061
NPFF	-2.938375736	1.86E-180
NPFFR1	-1.292668438	1.44E-43
NPFFR2	-0.139700634	5.19E-08
NPHP1	0.010974687	0.844292942
NPHP3	-1.038464844	1.87E-51
NPHP4	-0.160606345	0.009643778
NPHS1	-0.336466054	2.25E-35
NPHS2	0.000414104	0.920493436
NPIPA1	-2.455853028	9.37E-112
NPIPA2	-1.037110951	1.90E-44
NPIPA3	-0.279745141	1.63E-19
NPIPA5	-0.614098676	1.65E-06
NPIPA7	-0.56409522	7.11E-27
NPIPA8	-0.293864054	2.24E-11
NPIPB11	-0.277310125	5.41E-05
NPIPB12	-0.10608146	0.033699007
NPIPB13	-0.225010069	5.52E-07
NPIPB15	-0.041711835	0.780014897
NPIPB2	0.557105403	3.45E-23
NPIPB3	-0.19061044	0.003409004
NPIPB4	-0.343884904	5.79E-08

NPIPB5	-1.277185056	3.32E-67
NPIPB6	-0.164481594	0.000241392
NPIPB8	0.006583595	0.222168993
NPIPB9	0.152993168	5.84E-26
NPL	2.052573873	2.75E-164
NPLOC4	1.521456332	1.06E-132
NPM1	0.599286257	1.02E-20
NPM2	-2.61228311	1.42E-95
NPM3	2.915954059	5.72E-300
NPNT	2.626116997	5.20E-178
NPPA	1.01631801	4.22E-29
NPPB	-0.162152818	0.06362428
NPPC	-0.488165881	2.80E-08
NPR1	1.178350407	7.02E-149
NPR2	0.747178522	4.19E-13
NPR3	0.451143837	1.02E-64
NPRL2	0.345583223	3.35E-06
NPRL3	0.530555207	2.47E-21
NPS	0.259105035	4.80E-57
NPSR1	0.271827071	4.87E-45
NPTN	0.042622702	0.69631993
NPTX1	-1.302442905	6.95E-09
NPTX2	1.412196006	5.26E-21
NPTXR	-0.014271982	0.925900253
NPVF	-0.0982145	0.04796063
NPY	-0.343877519	0.097130813
NPY1R	-1.301696395	1.87E-39
NPY2R	0.814589262	2.47E-31
NPY4R	-0.028739243	0.005290546
NPY5R	-0.57991842	5.75E-18
NQO1	0.984722655	5.58E-31
NQO2	1.171066693	1.07E-32
NR0B1	0.398241351	7.18E-20
NR0B2	-0.031613915	0.09086435
NR1D1	-0.530309429	1.03E-06
NR1D2	0.133145239	0.130179246
NR1H2	0.352566423	8.42E-13
NR1H3	1.57611406	9.20E-302
NR1H4	0.230775371	9.92E-89
NR1I2	0.064676286	0.001719827
NR1I3	-0.25708417	4.77E-06
NR2C1	0.180410908	0.040881166
NR2C2	0.313115041	0.00034731
NR2C2AP	2.083045692	4.33E-248
NR2E1	1.252700736	4.02E-19
NR2E3	-0.012096858	0.360952799
NR2F1	1.114390002	1.33E-40
NR2F2	1.513435737	4.71E-104

NR2F6	0.618007043	1.07E-22
NR3C1	1.39952966	1.46E-83
NR3C2	-0.186782233	0.001559266
NR4A1	-0.627974956	2.50E-07
NR4A2	-0.349111466	0.00614893
NR4A3	0.60760016	8.27E-13
NR5A1	0.048752411	0.138393942
NR6A1	0.188938907	0.000154002
NRAP	-0.127465004	0.000114812
NRARP	1.787676037	9.69E-154
NRBF2	1.641839579	6.57E-130
NRBP1	1.305061911	2.11E-64
NRBP2	-0.676322776	1.02E-16
NRCAM	2.093272246	2.54E-138
NRDC	0.985038818	2.64E-58
NRDE2	-0.200053527	0.002052536
NREP	-1.443746648	4.87E-22
NRF1	1.067736192	6.82E-56
NRG1	-0.138873834	0.008317644
NRG2	0.220431923	0.01423118
NRG3	-0.705362182	1.45E-17
NRG4	-1.205297145	2.93E-32
NRGN	-1.305046676	7.10E-06
NRIP1	1.750058177	3.20E-112
NRIP2	-1.319907991	9.84E-23
NRIP3	-1.738455459	4.19E-44
NRK	-0.294682074	6.89E-05
NRL	0.292908311	6.73E-25
NRN1	1.472189074	1.12E-19
NRN1L	-2.300366754	3.87E-192
NRP1	2.724335115	8.83E-300
NRP2	2.672888926	3.69E-266
NRROS	1.774463898	2.11E-237
NRSN1	-1.304990149	4.16E-29
NRSN2	1.009565111	6.82E-34
NRTN	0.132855282	9.77E-07
NRXN1	-0.336385172	0.000369937
NRXN2	-0.480911931	1.86E-05
NRXN3	-1.924497834	2.41E-50
NSA2	0.312918347	4.02E-08
NSD1	0.800116357	4.43E-27
NSD2	1.151669894	1.37E-68
NSD3	0.412138273	2.61E-08
NSDHL	2.057700119	1.04E-227
NSF	-0.311109847	0.009777886
NSFL1C	0.971174813	2.84E-74
NSG1	-0.453795448	0.001080903
NSL1	0.798290896	9.77E-59

NSMAF	1.064087769	1.08E-52
NSMCE1	1.625515873	3.93E-253
NSMCE2	2.083572665	1.21197750020869e-315
NSMCE3	1.702547174	3.58E-209
NSMCE4A	0.607841916	1.09E-26
NSMF	-0.222820383	0.031205347
NSRP1	0.819898366	4.69E-60
NSUN2	0.614838379	4.81E-18
NSUN3	0.911893622	2.12E-111
NSUN4	1.573554829	6.29E-199
NSUN5	1.09341671	9.58E-61
NSUN6	-0.455069545	2.68E-13
NSUN7	1.099386657	8.72E-132
NT5C	0.143675141	0.048214207
NT5C1A	-0.184991623	0.013119001
NT5C1B	-0.018937433	3.75E-05
NT5C2	-1.008571797	1.58E-43
NT5C3A	1.611558524	4.94E-157
NT5C3B	0.813597616	1.24E-33
NT5DC1	-0.539486811	5.73E-18
NT5DC2	2.119863776	9.60E-111
NT5DC3	0.850287237	3.66E-24
NT5DC4	-0.000439157	0.946805701
NT5E	2.704592936	7.60E-204
NT5M	0.812444388	3.52E-47
NTAN1	-0.827559116	8.60E-61
NTF3	-0.400680443	1.00E-06
NTF4	0.099481111	9.72E-34
NTHL1	1.027522111	1.06E-63
NTM	-0.35968952	1.22E-05
NTMT1	1.230023095	3.99E-93
NTN3	0.423837964	3.56E-77
NTN4	0.027114102	0.737143561
NTN5	-0.486328738	3.88E-23
NTNG1	0.443322397	1.73E-09
NTNG2	-0.366830136	2.15E-06
NTPCR	1.275851583	4.78E-156
NTRK1	0.207829763	0.001895962
NTRK2	0.731303069	3.93E-15
NTRK3	0.26034558	0.000368133
NTS	-0.883805389	4.63E-10
NTSR1	-0.011817134	0.865549704
NTSR2	-1.637811899	3.34E-45
NUAK1	-0.093646334	0.390667477
NUAK2	1.729816629	2.68E-69
NUB1	1.584491041	8.75E-131
NUBP1	1.128525685	3.21E-99
NUBP2	1.477960597	2.75E-131

NUBPL	0.637294873	3.62E-48
NUCB1	1.958833683	8.93E-219
NUCB2	1.26025805	4.06E-123
NUCKS1	1.145681979	6.44E-99
NUDC	0.928730962	8.91E-53
NUDCD1	0.712291627	1.24E-39
NUDCD2	1.615194023	3.26E-246
NUDCD3	1.139545273	1.85E-53
NUDT10	0.683941628	1.13E-13
NUDT11	1.400826359	9.43E-38
NUDT12	1.523012783	2.83E-163
NUDT13	0.373990222	1.43E-19
NUDT14	0.045972881	0.602490866
NUDT15	0.977699116	2.34E-61
NUDT16	0.248323751	2.96E-06
NUDT16L1	1.709193979	3.10E-168
NUDT17	0.845421166	3.03E-43
NUDT18	0.759077746	2.10E-25
NUDT2	1.335529642	3.53E-126
NUDT21	0.95709768	5.95E-41
NUDT22	0.213200866	0.000162894
NUDT4	1.941788746	2.65E-131
NUDT5	0.97552918	6.80E-60
NUDT6	-0.107472076	0.015655992
NUDT7	0.008340078	0.865666148
NUDT8	0.227900756	1.75E-05
NUDT9	1.19954664	6.70E-80
NUFIP1	1.432967603	1.01E-178
NUFIP2	1.320162479	5.54E-87
NUGGC	0.098630012	4.58E-66
NUMA1	0.020500438	0.811440819
NUMB	0.665869214	2.71E-23
NUMBL	0.792813194	2.44E-18
NUP107	1.908281649	5.06E-171
NUP133	1.527199023	5.94E-101
NUP153	1.496107185	7.45E-76
NUP155	1.945798246	3.75E-226
NUP160	2.123120387	3.21E-174
NUP188	1.681832157	2.39E-99
NUP205	2.355868989	4.01E-202
NUP210	1.342636955	3.21E-58
NUP210L	0.063968736	0.000658038
NUP214	0.975022317	5.51E-56
NUP35	1.573707133	5.95E-161
NUP43	1.875101884	1.11E-211
NUP50	1.084892577	1.76E-61
NUP54	1.641791508	6.10E-101
NUP58	1.001441565	1.32E-56

NUP62	1.900975919	3.17E-137
NUP62CL	0.265703579	1.50E-08
NUP85	1.665911982	1.34E-54
NUP88	1.170479066	8.76E-73
NUP93	1.458238252	2.42E-98
NUP98	1.587419295	1.87E-109
NUPR1	-1.300870519	1.88E-54
NUPR2	-1.694375454	3.46E-90
NUS1	1.373396903	1.91E-118
NUSAP1	-0.50591241	3.18E-43
NUTF2	1.26855455	1.45E-145
NUTM1	-0.016402082	0.153590523
NUTM2A	-0.056593418	0.01167149
NUTM2B	0.019624688	0.182607051
NUTM2D	-0.01360153	0.620905915
NUTM2E	0.008378672	0.638035111
NUTM2F	0.038649034	8.05E-22
NUTM2G	0.200664815	5.26E-98
NVL	1.138264343	2.62E-52
NWD1	-0.258666639	0.001783999
NWD2	-0.565401302	9.34E-17
NXF1	0.840662248	4.80E-23
NXF2	0.001233762	0.218532235
NXF2B	-0.009498086	2.40E-08
NXF3	0.290016623	1.34E-77
NXN	0.746658305	5.06E-48
NXNL1	-0.483427582	6.25E-79
NXNL2	-0.142507275	0.000471107
NXPE1	0.010459152	0.004302463
NXPE2	0.088317136	2.99E-28
NXPE3	0.031348011	0.609558737
NXPE4	-0.005215103	0.313091577
NXPH1	1.051082484	3.04E-32
NXPH2	0.520279961	1.07E-14
NXPH3	0.268270013	0.053558105
NXPH4	2.104763161	9.40E-57
NYAP1	-0.912312767	1.68E-18
NYAP2	-0.311688945	1.15E-19
NYNRIN	0.473131578	0.00054795
NYX	0.061550996	8.44E-55
OAF	1.234711805	7.14E-25
OARD1	0.751166993	2.36E-59
OAS2	2.724116387	4.66397969674137e-321
OAT	0.313814479	1.18E-06
OAZ1	-0.1002092	0.116386672
OAZ2	-0.287826363	1.12E-09
OAZ3	-0.226443137	7.88E-07
OBP2A	0.331746832	3.83E-65

OBP2B	0.080672508	7.06E-30
OBSCN	-1.684490575	5.76E-51
OBSL1	1.23780528	8.22E-43
OC90	-0.147047804	1.82E-13
OCA2	-0.711340138	1.52E-19
OCEL1	1.221872843	5.00E-88
OCIAD1	0.537337825	3.21E-15
OCIAD2	1.920218205	7.91E-102
OCLN	0.200571529	5.83E-10
OCM	-0.074253184	0.16386904
OCM2	0.001919371	0.710581564
OCRL	0.884005094	5.12E-32
OCSTAMP	0.12878295	4.10E-62
ODAM	-0.009558988	0.676942573
ODF1	-0.230418161	1.66E-15
ODF2	1.173713525	1.69E-39
ODF2L	-0.098351723	0.159466271
ODF3	-0.028113703	3.05E-05
ODF3B	0.649418633	5.55E-08
ODF3L1	0.539710488	2.44E-85
ODF3L2	0.151369022	1.42E-09
ODF4	0.107824444	6.60E-105
OFD1	0.549196031	4.02E-21
OGDH	1.38698312	4.18E-112
OGDHL	-2.446908026	5.22E-116
OGFOD1	1.508181232	3.15E-131
OGFOD2	-2.126305274	1.66E-168
OGFOD3	1.519723402	1.68E-106
OGFR	0.94686804	1.74E-42
OGFRL1	0.858211639	1.63E-37
OGG1	0.574185162	1.91E-33
OGN	0.367274127	1.35E-06
OGT	-0.178004683	0.070003074
OIT3	-0.018514936	0.557385721
OLA1	1.441331071	7.85E-72
OLAH	0.110270075	3.26E-07
OLFM1	-0.844411885	1.05E-11
OLFM2	1.886070649	3.35E-132
OLFM3	-1.634998253	4.99E-43
OLFM4	0.145102934	0.000457792
OLFML1	1.402164036	1.90E-148
OLIG1	1.173083175	2.72E-48
OLIG2	2.337734383	3.60E-171
OLIG3	0.101324608	5.93E-33
OLR1	3.041148758	6.17E-123
OMA1	0.771463301	5.22E-69
OMD	-0.489537178	2.40E-13
OMG	-0.616366503	2.84E-14

OMP	0.748646222	1.74E-285
ONECUT1	0.360910462	3.36E-208
ONECUT2	-0.26213108	0.000626503
ONECUT3	-0.007669876	0.674987898
OOEP	-0.006899072	0.565753746
OOSP2	0.004155441	0.064285356
OPA1	0.423039887	1.11E-10
OPA3	1.28576982	3.34E-152
OPALIN	-3.655750679	6.05E-170
OPCML	-0.872259261	2.62E-11
OPHN1	0.76876521	1.75E-41
OPLAH	0.132851971	0.08134629
OPN1LW	-0.003580425	0.035551201
OPN1MW	0.000723737	0.230612133
OPN1MW2	0	1
OPN1MW3	-3.48E-05	0.948277253
OPN1SW	-0.370822888	3.08E-22
OPN3	0.167122459	0.03153112
OPN4	-0.65291298	1.12E-18
OPN5	0.021856668	0.000235875
OPRD1	-0.170384704	0.015090117
OPRK1	-0.791031441	5.02E-27
OPRL1	-2.180158994	5.72E-121
OPRM1	-0.232004674	3.48E-10
OPRPN	-0.003456607	0.281496339
OPTC	0.294779577	2.24E-89
OPTN	-0.172883725	0.011489212
OR10A2	-0.015958422	0.004110515
OR10A3	0.007286817	0.000102393
OR10A4	-0.009635387	0.0062012
OR10A5	-0.008470346	0.007599157
OR10A6	-0.002702383	0.168600993
OR10A7	0.000398955	0.84088509
OR10AD1	-0.19351842	3.37E-10
OR10AG1	-0.000133912	0.928871463
OR10C1	0.001006696	0.567074414
OR10D3	-0.000480043	0.739666429
OR10G2	0.004556507	0.051514284
OR10G3	-0.00155803	0.426577666
OR10G4	0.002415661	0.07445876
OR10G6	-0.001179577	0.535296573
OR10G7	-0.006801413	0.014754218
OR10G8	-0.000691875	0.683370326
OR10G9	-0.001912796	0.194884346
OR10H1	0.011642396	5.08E-06
OR10H2	0.012857574	8.18E-07
OR10H3	0.00157525	0.448097412
OR10H4	-6.33E-06	1

OR10H5	-0.000130315	0.984678238
OR10J1	-0.001490073	0.481872156
OR10J5	-0.003525115	0.090548615
OR10K1	0.000538484	0.723803675
OR10K2	0.003613686	0.009678288
OR10P1	0.00134266	0.561378684
OR10Q1	0.081482898	1.12E-47
OR10R2	0	1
OR10S1	-0.001196318	0.468047486
OR10T2	-0.000767187	0.57221566
OR10V1	-0.005967202	0.016594606
OR10W1	0.000519134	0.645493529
OR10X1	0.001373689	0.520359459
OR10Z1	0.001499859	0.491096417
OR11A1	-0.004980261	0.241860636
OR11G2	0.005028401	0.025800374
OR11H1	0.004008844	0.020260672
OR11H12	0.00472663	0.018041225
OR11H2	0.002348976	0.14401371
OR11H4	-0.052840736	3.31E-07
OR11H6	-0.006043897	0.050712365
OR11L1	-0.000319268	0.887455809
OR12D2	-0.025680805	0.2530039
OR12D3	-0.002407489	0.252491456
OR13A1	-0.161036566	7.38E-07
OR13C2	-0.049625064	4.03E-09
OR13C3	-0.002572484	0.387686454
OR13C4	-0.002628089	0.247011549
OR13C5	-0.03525386	1.60E-05
OR13C8	-0.000144491	0.941974428
OR13C9	-0.011316026	0.009178794
OR13D1	-0.004495875	0.169163658
OR13F1	-0.004316601	0.132346037
OR13G1	-0.00316897	0.178502954
OR13H1	-0.000429485	0.828811359
OR13J1	-0.280720015	6.56E-28
OR14A16	-0.000419116	0.812333653
OR14A2	0.000786661	0.599704239
OR14C36	-0.020790829	9.37E-05
OR14I1	-0.536951038	2.87E-17
OR14J1	-0.001712044	0.550615406
OR14K1	0.000958936	0.737143561
OR1A1	0.001276366	0.554823423
OR1A2	-0.001358951	0.611536101
OR1B1	0.008070746	0.085703986
OR1C1	-0.007868714	0.005232956
OR1D2	-0.023858843	3.01E-06
OR1D5	-0.008130639	0.01203254

OR1E1	0.038701708	0.000228593
OR1E2	0.000363042	0.955425945
OR1F1	-0.639465503	3.57E-16
OR1G1	0.054480413	7.96E-25
OR1I1	0.010122383	1.72E-10
OR1J1	0.067011203	5.67E-37
OR1J2	0.012974495	1.28E-08
OR1J4	0.026866713	2.38E-23
OR1K1	0.017849497	0.00131627
OR1L1	-0.002709834	0.375694626
OR1L3	0.013841245	0.059826868
OR1L4	-0.00038633	0.911732581
OR1L6	0.002040481	0.482641915
OR1L8	0.19160789	7.88E-29
OR1M1	0.015285797	1.46E-13
OR1N1	0.017265498	0.015463261
OR1N2	0.005917019	0.029093443
OR1Q1	0.038519595	4.64E-16
OR1S1	0.001461143	0.405738805
OR1S2	-0.001000455	0.617242446
OR2A1	-0.016222492	0.023866917
OR2A12	0.007883778	1.32E-05
OR2A14	-0.005638826	0.073941928
OR2A2	0.001834155	0.362263204
OR2A25	0.000544843	0.84752729
OR2A4	-0.003103025	0.351482307
OR2A42	0.020357437	1.26E-06
OR2A5	0.000419593	0.852940565
OR2A7	-0.082231785	1.50E-09
OR2AE1	0.105251883	2.64E-32
OR2AG1	-0.012537352	0.004424768
OR2AG2	-0.041038871	4.62E-07
OR2AJ1	-0.046503788	7.55E-08
OR2AK2	-0.045482728	6.73E-11
OR2AP1	0.023260884	9.87E-33
OR2AT4	0.004544437	0.077306732
OR2B11	0.079403109	1.67E-65
OR2B2	0.009510366	4.25E-05
OR2B3	0.00671853	8.20E-05
OR2B6	0.757565757	1.79E-298
OR2C1	0.070494901	9.58E-06
OR2C3	-0.008759993	0.187825238
OR2D2	-0.024182902	8.72E-05
OR2D3	-0.00325974	0.235183334
OR2F1	0.009931896	0.001215964
OR2F2	-0.006436657	0.014857797
OR2G2	-0.007063314	0.042363233
OR2G3	-0.002355853	0.301999661

OR2G6	-0.002986311	0.190681219
OR2H1	-0.005558937	0.560866335
OR2H2	-0.588728085	1.19E-34
OR2J1	-0.001437653	0.335302185
OR2J2	-0.006584706	0.509468571
OR2J3	0.00772584	2.87E-05
OR2K2	-0.126116543	3.15E-06
OR2L13	-1.155326088	3.08E-43
OR2L2	-0.254366296	3.06E-13
OR2L3	-0.144429588	1.18E-13
OR2L5	-0.069123704	7.54E-14
OR2L8	-0.018911248	0.00019217
OR2M2	-0.004248228	0.148909006
OR2M3	-0.028843075	1.16E-06
OR2M4	-0.059984477	2.34E-07
OR2M5	-0.004050298	0.170020194
OR2M7	-0.00652365	0.032845152
OR2S2	-0.012389256	0.026993673
OR2T1	-0.000520234	0.781238789
OR2T10	-0.00474324	0.063709637
OR2T11	-0.00097019	0.502915446
OR2T12	-0.006273526	0.023320846
OR2T2	0.000740271	0.657078401
OR2T27	0.001039568	0.233683627
OR2T29	-0.00027665	0.771016252
OR2T3	-0.001440037	0.218415441
OR2T33	-0.087083569	6.32E-14
OR2T34	-0.001618203	0.222949946
OR2T35	-0.000656517	0.417326433
OR2T4	-0.023902995	0.001809614
OR2T5	0.00030302	0.734832645
OR2T6	-0.012088262	0.004118492
OR2T7	0.001413893	0.365628931
OR2T8	-0.157700184	6.22E-14
OR2V1	-0.012004673	0.019729333
OR2V2	-0.00786462	0.071623041
OR2W1	0.000262474	0.876601096
OR2W3	-0.374715228	1.46E-07
OR2Y1	-0.000978398	0.532873512
OR2Z1	0.000930673	0.606396306
OR3A1	0.098235943	5.10E-38
OR3A2	0.11187549	7.25E-08
OR3A3	-0.064821136	1.45E-06
OR4A15	-0.00139806	0.443980058
OR4A16	0.01209699	1.94E-07
OR4A47	-0.004994622	0.216770484
OR4A5	-0.000754182	0.526608459
OR4B1	0.001049963	0.496998535

OR4C11	-0.000242774	0.817704426
OR4C12	0.000483584	0.670608435
OR4C13	-0.001857748	0.357965504
OR4C15	8.48E-05	0.958534628
OR4C16	-0.000801052	0.722551824
OR4C3	-0.001335609	0.459257591
OR4C46	-0.001135604	0.498686841
OR4C5	0.000209992	0.857546987
OR4C6	0.01721091	3.56E-15
OR4D1	0.018716496	1.45E-07
OR4D10	-0.001718301	0.423228774
OR4D11	-0.001551336	0.327981034
OR4D2	0.000842908	0.707579137
OR4D5	-0.002477953	0.105538753
OR4D6	-0.001385054	0.393266443
OR4D9	0.002894633	0.050869627
OR4E1	-0.006867029	0.010576849
OR4E2	-0.000724772	0.773156211
OR4F15	0.000938507	0.716972232
OR4F17	-0.000836612	0.694380722
OR4F21	-0.001146438	0.771016252
OR4F3	-0.001283437	0.129608748
OR4F4	-0.000101857	0.966800204
OR4F5	-0.144793408	1.15E-13
OR4F6	0.005346947	0.236506072
OR4K1	0.01627082	2.05E-07
OR4K13	0.001128194	0.457366529
OR4K14	0.001129504	0.441584199
OR4K15	-0.001356216	0.519708813
OR4K17	0.001869138	0.336595979
OR4K2	0.062247806	4.10E-46
OR4K5	0.001332653	0.597568275
OR4L1	-0.000330624	0.873950492
OR4M1	0.00381511	0.102605221
OR4M2	0.001852863	0.00380144
OR4N2	0.637133773	6.81E-168
OR4N4	-0.005990789	0.033105846
OR4N5	-0.003317946	0.182213575
OR4P4	0.001566867	0.237994709
OR4Q3	-0.001654844	0.460412254
OR4S1	-2.07E-05	0.992745805
OR4S2	-0.000224605	0.930295054
OR4X1	-0.000405725	0.75171945
OR4X2	0.005255784	0.01282588
OR51A2	-0.000430036	0.53392816
OR51A4	-3.96E-06	0.999426236
OR51A7	0.00061345	0.71597457
OR51B2	0.008692902	0.001822495

OR51B4	0.070109097	9.49E-40
OR51B5	0.10579281	2.82E-76
OR51B6	-0.002387102	0.35387317
OR51D1	-0.000968486	0.561315879
OR51E2	0.191910485	5.15E-27
OR51F1	-0.001922303	0.283221318
OR51F2	-0.000640082	0.765627356
OR51G1	-0.002862155	0.118472161
OR51G2	-0.003082078	0.077770286
OR51I1	0.004380254	0.156438918
OR51I2	0.003425098	0.266377337
OR51L1	0.000231236	0.898266084
OR51M1	-0.00149623	0.707086522
OR51Q1	-0.036663511	3.69E-05
OR51S1	-0.002116099	0.258405954
OR51T1	-0.003077247	0.097387667
OR51V1	-0.003395989	0.133305146
OR52A1	-0.008336512	0.015486266
OR52A5	-0.012022022	0.017027005
OR52B2	-0.00577026	0.140014372
OR52B4	-0.005469347	0.025058708
OR52B6	0.013310197	0.000675257
OR52D1	0.014059504	5.26E-05
OR52E2	0.052292467	1.05E-63
OR52E4	-0.001079213	0.639047731
OR52E5	0.001790207	0.457247093
OR52E6	0.000185847	0.948981995
OR52E8	-0.000995301	0.554991763
OR52H1	0.016283217	2.92E-06
OR52I1	-0.009528359	0.268476492
OR52I2	0.003583365	0.337976625
OR52J3	-0.002923368	0.378574481
OR52K1	0.016614043	5.65E-10
OR52K2	0.017445529	2.15E-09
OR52L1	-0.000190871	0.948334376
OR52M1	0.003751455	0.086054442
OR52N1	0.000186298	0.962288715
OR52N2	0.000535891	0.955649276
OR52N4	0.061851801	0.006563161
OR52N5	-0.001634299	0.510717609
OR52R1	-0.003910413	0.023588819
OR52W1	-0.040126466	8.59E-07
OR56A1	-0.002254561	0.119245514
OR56A3	-0.003875825	0.098130949
OR56A4	-0.000192089	0.920672966
OR56A5	-0.006480895	0.030038684
OR56B1	-0.0048723	0.49177658
OR56B4	0.014631025	0.003297347

OR5A1	-0.001073945	0.578214336
OR5A2	-0.000826831	0.636864062
OR5AC2	0.020014139	5.39E-13
OR5AK2	-0.000383171	0.963924343
OR5AN1	-0.001005402	0.64831627
OR5AP2	8.73E-05	0.956983611
OR5AR1	-0.001747252	0.341704482
OR5AS1	0.000537223	0.71207503
OR5AU1	-0.007449246	0.197478099
OR5B12	-0.038269249	8.66E-07
OR5B17	0.001748093	0.224287686
OR5B2	-0.005344966	0.379152597
OR5B21	-0.005411897	0.153954542
OR5B3	0.008207252	1.36E-05
OR5C1	0.013511138	0.000701545
OR5D13	-0.001589691	0.383738822
OR5D14	-0.000355927	0.828713009
OR5D16	-0.000490296	0.73390003
OR5D18	-0.001713	0.432300444
OR5F1	-0.000765062	0.423628441
OR5H1	-0.000879217	0.696090083
OR5H14	0.000282737	0.868599262
OR5H15	-0.00081211	0.741468291
OR5H2	-0.001049564	0.547501382
OR5H6	-0.001250529	0.576776495
OR5I1	-7.14E-05	0.956997572
OR5J2	-0.009212874	0.134056858
OR5K1	7.38E-05	0.9818805
OR5K2	-0.198894464	9.13E-10
OR5K3	-0.003704237	0.102707098
OR5K4	-0.001748201	0.298458892
OR5L1	-0.000821928	0.627564074
OR5L2	-0.001702304	0.396033013
OR5M1	-0.000511954	0.728490517
OR5M10	-0.001206065	0.379512829
OR5M11	0.000746994	0.552544741
OR5M3	-0.000698602	0.600948614
OR5M8	0.000906033	0.444146102
OR5M9	-0.003633363	0.288754987
OR5P2	0.000185482	0.905881494
OR5P3	-0.000829135	0.546008031
OR5T1	-0.001448513	0.53673324
OR5T2	-0.001261047	0.528830788
OR5T3	-0.001703174	0.447906724
OR5V1	-0.013207672	0.047203307
OR5W2	-0.000215948	0.877333568
OR6A2	-0.021720408	1.13E-05
OR6B1	-0.002109749	0.405535483

OR6B2	0.006447264	0.269755364
OR6B3	-0.062661098	0.002021483
OR6C1	0.002994759	0.052808852
OR6C2	0.003901607	0.123513093
OR6C3	0.006735548	0.004201053
OR6C4	0.001364781	0.483128411
OR6C6	0.001706336	0.349103067
OR6C65	0.002296285	0.16445217
OR6C68	0.00607733	0.001978337
OR6C70	0.004730995	0.00731879
OR6C74	-0.000968536	0.51592843
OR6C75	0.00103401	0.628382876
OR6C76	0.000151513	0.937238411
OR6F1	-0.000295171	0.917799415
OR6J1	0.038431145	1.81E-22
OR6K2	0.000284983	0.854374727
OR6K3	0.011722757	9.59E-10
OR6K6	-0.001000265	0.440831273
OR6M1	-0.003809334	0.170489112
OR6N1	0.000624384	0.693711419
OR6N2	-0.001084769	0.50265252
OR6P1	0.000305367	0.772348434
OR6Q1	-0.002263435	0.183478374
OR6S1	-0.000450223	0.85306014
OR6T1	-0.001310982	0.555509603
OR6V1	-0.02232734	0.000174843
OR6X1	-0.009431619	0.003576305
OR6Y1	-6.95E-05	0.967635951
OR7A10	0.0021884	0.099789385
OR7A17	0.026162032	2.43E-77
OR7A5	0.406998699	8.60E-19
OR7C1	0.042205676	0.034507536
OR7C2	0.036725458	0.000246354
OR7D2	0.20411088	1.55E-09
OR7D4	0.008911393	0.000181181
OR7E24	0.007385068	2.23E-05
OR7G1	0.005102881	0.003170788
OR7G2	0.019345002	1.73E-37
OR7G3	-0.00577962	0.074979305
OR8A1	-0.00840933	0.003439581
OR8B12	-0.001914613	0.196636916
OR8B4	-0.000121296	0.938305394
OR8B8	-0.003174643	0.163084217
OR8D1	-0.002387246	0.203717563
OR8D2	-0.000958698	0.605996382
OR8D4	-0.008528958	0.010293412
OR8G1	0.000739392	0.52608158
OR8G5	0.006292938	0.006284797

OR8H1	0.000110356	0.952055458
OR8H2	-6.07E-05	0.980381159
OR8H3	-0.00093063	0.627592577
OR8I2	0.00034986	0.823872416
OR8J1	-0.000445593	0.824730879
OR8J3	-0.000871938	0.524021156
OR8K1	-0.000427378	0.835864112
OR8K3	-0.000218904	0.899583925
OR8K5	0.000688358	0.735735526
OR8U1	-0.003593938	0.156208827
OR9A2	-0.046616444	0.003935916
OR9A4	0.032524495	6.14E-13
OR9G1	-0.000417649	0.842838227
OR9G4	-0.000719522	0.7122398
OR9I1	-0.000546518	0.806211965
OR9K2	0.001759584	0.562433959
OR9Q1	0.005188373	0.000373006
OR9Q2	-0.001434752	0.268364728
ORAI2	1.468829041	1.02E-76
ORAI3	0.610881265	3.25E-44
ORC2	0.866197494	6.45E-34
ORC3	1.19109391	3.35E-77
ORC4	1.011342897	6.64E-44
ORC5	1.85250137	1.03E-137
ORC6	1.397684111	5.83E-104
ORM1	0.067971616	0.217942532
ORM2	0.365867299	1.73E-48
ORMDL1	1.190774119	1.11E-84
ORMDL2	0.813966472	7.74E-51
ORMDL3	1.477815725	1.29E-202
OS9	1.830838074	3.99E-126
OSBP	1.419371811	3.90E-111
OSBP2	0.084560565	0.307562255
OSBPL10	1.278054583	1.53E-147
OSBPL11	1.821794762	1.22E-209
OSBPL1A	0.077440668	0.368011309
OSBPL2	-0.18508712	0.011694132
OSBPL3	2.487481377	6.57E-215
OSBPL5	1.164921877	9.80E-100
OSBPL6	1.071959063	1.46E-59
OSBPL7	-0.021833124	0.765969056
OSBPL8	1.335415136	8.20E-83
OSBPL9	0.23279408	8.50E-05
OSCAR	2.291059587	1.57E-233
OSCP1	1.076612539	1.44E-52
OSER1	1.94043038	5.95E-181
OSGEP	0.437649388	2.54E-08
OSGEPL1	0.980848289	1.42E-58

OSGIN1	0.167287651	0.005778527
OSGIN2	1.690865725	9.94E-178
OSM	2.374251173	8.27E-269
OSMR	2.926947141	1.27E-247
OSR1	1.060310623	7.15E-103
OSTF1	1.439529882	3.49E-99
OSTM1	1.872694014	3.04E-200
OSTN	0.186311704	9.28E-05
OTC	0.140259204	7.34E-14
OTOA	0.181566395	2.34E-29
OTOF	-1.065858692	5.42E-20
OTOG	0.155956061	2.28E-44
OTOGL	0.054536081	0.167715199
OTOL1	0.049608035	2.71E-07
OTOP1	-0.009224891	0.07102812
OTOP2	-0.010364422	0.090882766
OTOP3	-0.018336634	0.002092136
OTOR	0.253940815	1.58E-58
OTOS	1.144350054	7.36E-37
OTP	1.884923693	3.71E-104
OTUB1	0.245233356	0.001574461
OTUB2	0.755482685	7.98E-82
OTUD1	0.777479754	6.31E-35
OTUD3	-0.065805254	0.452235276
OTUD4	0.752186691	7.80E-26
OTUD5	0.759110083	1.88E-28
OTUD6A	0.002515991	0.585524897
OTUD6B	1.575060542	3.60E-161
OTUD7A	-1.407558497	9.96E-79
OTUD7B	0.241552179	7.58E-05
OTULIN	1.255279168	7.25E-134
OTX1	1.320799149	5.34E-71
OTX2	-0.779164648	1.81E-08
OVCH1	0.058822598	1.39E-09
OVCH2	-0.015341612	0.007196973
OVGP1	-0.615719347	2.59E-13
OVOL1	-0.094792957	0.000116853
OVOL2	-0.226954017	4.40E-09
OVOL3	0.322445058	3.12E-44
OXA1L	1.425017271	2.48E-111
OXCT1	0.491758746	3.03E-08
OXCT2	-0.301475845	1.63E-15
OXER1	0.620282256	7.71E-63
OXGR1	-0.19187419	2.12E-07
OXLD1	1.348821476	1.78E-147
OXNAD1	1.304069645	6.26E-205
OXR1	0.859775693	3.05E-17
OXSM	1.611184583	1.15E-179

OXSR1	1.417789145	1.83E-102
OXT	-0.950730497	2.78E-07
OXTR	1.678360096	9.56E-83
P2RX1	0.126011911	0.00124997
P2RX2	-0.194817564	0.011804332
P2RX3	0.310402253	3.10E-232
P2RX4	0.149610983	0.192908063
P2RX5	-2.073643317	3.27E-110
P2RX6	-0.312447752	0.000176717
P2RX7	-0.137004966	0.1009212
P2RY1	2.388537219	1.45E-156
P2RY10	0.386224472	1.67E-220
P2RY11	-0.463108312	5.34E-13
P2RY12	2.384116268	2.01E-81
P2RY13	2.358481724	3.52E-179
P2RY14	0.171783328	0.000444136
P2RY2	-0.222244433	3.67E-09
P2RY4	0.109971421	2.95E-59
P3H1	1.272980098	4.81E-74
P3H2	2.067160732	9.50E-197
P3H3	-0.008830685	0.948171578
P3H4	2.314435846	1.57E-236
P4HA1	2.338954542	2.23E-243
P4HA2	1.414350669	9.91E-122
P4HA3	0.525686814	6.10E-27
P4HB	2.122470846	4.22E-211
P4HTM	0.104805425	0.201770233
PA2G4	1.005193413	2.13E-57
PAAF1	-0.008327601	0.861021649
PABPC1	1.431579	2.69E-89
PABPC1L	1.958288454	2.32E-169
PABPC1L2A	-1.720518642	5.12E-96
PABPC1L2B	-1.749711719	9.50E-97
PABPC4	0.296550587	7.02E-07
PABPC5	0.398974885	9.41E-22
PABPN1L	-0.580901299	1.37E-20
PACRG	1.60267636	3.55E-95
PACRGL	0.597793821	3.34E-40
PACS1	0.502845067	2.13E-11
PACS2	-0.875894408	8.70E-37
PACSIN1	-4.20938652	1.62E-166
PACSIN2	1.347608687	7.76E-78
PACSIN3	0.014175798	0.826822869
PADI1	-0.016853197	0.645438704
PADI2	0.919985292	2.64E-20
PADI3	0.048409491	6.68E-36
PADI4	-0.00301352	0.940739647
PADI6	0.03642459	7.05E-41

PAEP	0.208630847	2.26E-36
PAF1	1.188423222	3.66E-84
PAFAH1B1	-0.492675226	2.50E-09
PAFAH1B2	1.196027441	1.27E-67
PAFAH1B3	1.651847461	1.07E-89
PAG1	1.384498931	1.32E-42
PAGE1	0.032934864	3.38E-12
PAGE2	0.010924885	0.411390832
PAGE2B	0.104196359	4.56E-09
PAGE3	-0.006527926	0.091148687
PAGE4	-0.076953086	2.78E-08
PAGE5	0.157923391	9.15E-90
PAGR1	-2.837213585	8.41E-106
PAH	-0.437580593	6.50E-19
PAIP1	1.87870401	2.58E-219
PAIP2	0.336730456	5.42E-06
PAIP2B	-2.598777003	5.40E-145
PAK1	-0.525277369	0.000110673
PAK1IP1	2.297857198	1.68E-288
PAK2	2.020705456	6.37E-275
PAK3	-0.794401002	1.81E-11
PAK4	1.62440088	1.07E-261
PAK5	-1.425269416	3.22E-32
PAK6	-1.653578821	3.26E-48
PALB2	1.452392059	1.99E-120
PALD1	1.884221265	2.11E-166
PALM	-1.398033295	2.75E-98
PALM3	0.845197343	6.59E-29
PALMD	0.164049979	0.00666772
PAM	1.912116692	4.70E-103
PAM16	-1.834540873	2.43E-280
PAMR1	1.741630475	1.76E-64
PAN2	-0.272358079	0.008142945
PAN3	0.577373328	1.25E-09
PANK1	0.127877185	0.084766734
PANK2	0.990891347	8.93E-47
PANK3	0.620344847	6.29E-15
PANK4	0.85603522	4.32E-26
PANX1	2.304592038	4.03E-236
PANX2	-2.189884059	1.44E-83
PANX3	-0.101844031	8.55E-10
PAOX	-1.754481391	1.44E-220
PAPLN	0.080862278	0.362713243
PAPOLA	0.609519869	1.58E-25
PAPOLB	-0.039923004	5.82E-10
PAPOLG	0.964589758	5.37E-54
PAPPA	0.342255033	8.88E-61
PAPPA2	0.702181074	3.51E-88

PAPSS1	1.633844615	9.60E-120
PAPSS2	1.465700128	1.45E-143
PAQR3	0.281439503	9.70E-05
PAQR4	0.625937643	4.62E-13
PAQR5	-0.222211596	2.02E-10
PAQR6	-1.680489001	4.04E-52
PAQR7	1.210106035	1.39E-119
PAQR8	0.022776477	0.750501121
PAQR9	0.086287595	0.016291628
PARD3	1.260629402	2.46E-87
PARD3B	1.017756145	4.29E-87
PARD6A	0.016114135	0.86447294
PARD6B	0.742347052	5.80E-58
PARD6G	1.257990137	1.49E-225
PARG	0.77956584	7.92E-33
PARK7	1.100202501	8.75E-85
PARL	-0.965269317	2.04E-74
PARM1	-0.08512563	0.532466344
PARN	0.736495351	3.85E-35
PARP1	0.585807982	1.10E-25
PARP10	1.602767097	2.13E-183
PARP14	1.960410971	1.90E-206
PARP15	0.647236002	7.57E-146
PARP16	1.170209919	1.80E-64
PARP2	-0.190920263	0.032119504
PARP3	1.464808948	4.47E-170
PARP4	2.51059473	1.07E-277
PARP6	-0.305002697	0.000124848
PARP8	1.316978881	8.34E-126
PARPBP	1.726496126	1.78E-267
PARVA	0.817374677	9.30E-56
PARVB	0.497931042	2.11E-19
PARVG	1.459769078	1.80E-73
PASD1	0.004321627	0.218972506
PASK	-0.387025107	9.19E-05
PATE1	0.002061408	0.510012641
PATE2	-0.041533593	0.006541021
PATE3	-0.004449901	0.263307541
PATE4	-0.028873093	0.000195249
PATJ	0.21567645	0.115522743
PATL1	1.846099386	2.93E-159
PATL2	0.684586899	1.85E-206
PATZ1	1.400411619	8.36E-125
PAWR	0.581723959	2.96E-48
PAX1	0.330649496	5.02E-71
PAX2	-0.117775448	0.033559838
PAX3	0.566654383	3.12E-16
PAX4	0.024849354	5.15E-29

PAX5	0.119472898	1.61E-07
PAX6	-0.373067158	0.002194093
PAX7	-0.220251013	1.22E-06
PAX8	0.277012399	2.66E-09
PAX9	0.103918119	9.88E-19
PAXBP1	0.586244107	4.32E-12
PAXIP1	0.392911468	0.000716635
PBDC1	2.050593701	1.61E-285
PBLD	0.059863754	0.236252068
PBOV1	-0.07534133	1.30E-09
PBRM1	1.279982982	6.95E-86
PBX1	0.70709167	7.68E-32
PBX2	0.178145253	0.011576134
PBX3	1.59369609	3.70E-74
PBX4	0.498859611	3.77E-30
PBXIP1	1.331278819	8.83E-67
PC	-0.502009366	2.85E-18
PCBD1	1.01951424	4.02E-71
PCBP1	-2.931103474	5.89E-298
PCBP2	0.191053983	0.004560048
PCBP3	-1.188780609	1.95E-27
PCBP4	-0.179105661	0.086170072
PCCA	0.539591559	1.53E-17
PCCB	1.853444148	1.73E-222
PCDH1	1.429178118	1.02E-71
PCDH10	1.374853188	6.88E-41
PCDH11X	-0.188085392	0.002756464
PCDH11Y	-0.318661467	5.09E-10
PCDH12	2.440844336	3.38E-286
PCDH15	0.156852463	0.000720091
PCDH17	1.961074303	2.00E-152
PCDH19	1.46012819	2.31E-60
PCDH20	-1.702886133	3.36E-84
PCDH7	0.171166382	0.063731888
PCDH8	0.092438571	0.513576961
PCDH9	0.34044511	3.86E-10
PCDHA1	-1.118231998	2.51E-94
PCDHA10	0.034637957	0.524522444
PCDHA11	0.136286962	0.03165551
PCDHA12	-0.194717979	0.000197642
PCDHA13	-0.150403657	0.000529028
PCDHA2	-0.069640132	0.211208812
PCDHA3	-0.054958895	0.20759649
PCDHA4	0.281910051	5.98E-33
PCDHA5	-0.331577106	5.24E-13
PCDHA6	-0.360641561	1.20E-11
PCDHA7	0.020783114	0.619243589
PCDHA8	-0.114397026	0.007681178

PCDHA9	-0.059409224	0.017443319
PCDHAC1	0.083896266	0.002147451
PCDHAC2	-0.517228896	4.85E-08
PCDHB10	2.169980409	7.90E-297
PCDHB11	0.265241818	4.10E-15
PCDHB12	0.670698945	5.67E-49
PCDHB13	0.820612474	1.98E-122
PCDHB14	1.571102731	6.97E-126
PCDHB15	0.246615428	0.000150171
PCDHB2	1.304682573	3.71E-180
PCDHB3	1.585788007	2.18E-248
PCDHB4	0.051573403	0.412412117
PCDHB5	1.017922616	5.58E-79
PCDHB6	1.062785085	1.02E-102
PCDHGA1	0.439874693	2.67E-22
PCDHGA10	0.802645711	6.59E-51
PCDHGA11	-0.234830305	8.81E-07
PCDHGA12	-0.892808188	6.78E-31
PCDHGA2	-0.149302908	0.003046068
PCDHGA3	-0.931768995	3.91E-52
PCDHGA4	0.275870074	4.46E-07
PCDHGA5	-0.259407041	2.42E-06
PCDHGA6	0.209116834	4.89E-05
PCDHGA7	0.035175642	0.434824868
PCDHGA8	-0.014554263	0.342209838
PCDHGA9	0.38997351	6.50E-09
PCDHGB1	0.128356929	0.018004868
PCDHGB2	1.844651942	5.70E-160
PCDHGB3	0.500804202	4.80E-40
PCDHGB4	-0.46812215	3.59E-34
PCDHGB5	-0.325315891	1.54E-09
PCDHGB6	0.300298498	3.17E-09
PCDHGB7	1.110699268	1.36E-43
PCDHGC3	2.124479907	4.67E-124
PCDHGC4	1.133903502	1.18E-20
PCDHGC5	0.739719612	9.42E-11
PCED1A	1.377830486	1.22E-116
PCED1B	1.624877661	1.73E-184
PCF11	0.598524935	1.96E-16
PCGF1	1.924288822	1.04E-180
PCGF2	0.209067848	0.005567246
PCGF3	-0.209887607	0.015612385
PCGF5	0.723300099	3.24E-46
PCGF6	0.881370069	1.21E-49
PCID2	1.120831276	3.31E-43
PCIF1	1.693741946	1.45E-95
PCK1	-0.528567129	6.10E-22
PCK2	1.836459042	1.60E-222

PCLO	-1.839632087	1.57E-56
PCM1	0.244911631	0.000152103
PCMT1	0.637375757	1.84E-13
PCMTD1	0.269996561	1.49E-06
PCMTD2	1.522259766	2.50E-84
PCNP	1.223862948	1.34E-83
PCNT	0.100820747	0.202651532
PCNX1	0.495091914	5.98E-15
PCNX2	-0.30175463	0.002384407
PCNX3	1.414587228	3.51E-85
PCNX4	1.132650318	3.74E-98
PCOLCE	2.71504381	1.57E-128
PCP2	-0.83187892	6.19E-06
PCP4	-2.715680871	4.23E-49
PCP4L1	-2.37369022	1.55E-46
PCSK1	1.51109892	1.82E-26
PCSK1N	-1.114032437	8.26E-35
PCSK2	-1.124285714	6.12E-24
PCSK4	-0.002295427	0.967418707
PCSK6	-1.024549531	4.01E-24
PCSK7	-0.601599352	1.41E-17
PCSK9	-0.772787523	2.50E-08
PCTP	1.082982201	1.34E-170
PCYOX1	1.401225882	8.57E-92
PCYOX1L	0.319582947	0.000418817
PCYT1A	1.084884584	4.06E-94
PCYT1B	0.680862314	2.27E-31
PCYT2	-0.260668019	1.04E-06
PDAP1	2.006749836	1.23E-235
PDC	0.02988399	0.23822873
PDCD1	0.667230637	1.55E-102
PDCD10	1.574006135	1.97E-167
PDCD11	0.955728224	2.88E-45
PDCD2	1.654472257	4.49E-222
PDCD2L	0.56942583	1.23E-18
PDCD4	0.240314449	2.21E-05
PDCD5	1.126858928	4.92E-77
PDCD6IP	1.012528406	1.78E-53
PDCD7	1.026817189	1.73E-83
PDCL	1.911724117	1.02E-268
PDCL2	0.125258919	3.29E-21
PDCL3	2.067789226	4.94E-277
PDE10A	-1.198124267	8.10E-28
PDE11A	-0.35462252	3.19E-16
PDE12	1.541821521	4.02E-179
PDE1A	-1.095299978	4.98E-16
PDE1B	-2.70250491	4.68E-64
PDE1C	0.009099786	0.91100905

PDE2A	-1.482742413	1.11E-22
PDE3A	0.342454988	9.71E-08
PDE3B	0.130264711	0.153954542
PDE4A	-0.316053352	0.000476939
PDE4B	1.927361776	4.38E-195
PDE4C	-1.035573868	2.94E-68
PDE4D	0.915813826	6.44E-73
PDE4DIP	0.560289072	5.44E-15
PDE5A	0.847311644	1.95E-96
PDE6A	0.259980253	1.64E-89
PDE6B	0.125263084	0.180286815
PDE6C	-0.16565216	7.93E-05
PDE6D	1.167780023	3.16E-125
PDE6H	-0.279915168	7.60E-10
PDE7A	0.235790929	0.000350882
PDE7B	0.251363446	0.006187168
PDE8A	0.079864774	0.343025481
PDE8B	-0.779671489	1.26E-28
PDE9A	0.523840418	4.58E-17
PDF	-2.451641799	2.43E-278
PDGFA	1.640310689	1.58E-117
PDGFB	1.712258992	5.11E-190
PDGFD	2.487936649	1.46E-299
PDGFRA	1.867183706	1.91E-105
PDGFRB	1.63793969	3.50E-149
PDGFRL	1.30621865	1.86E-132
PDHA1	0.573028429	4.77E-18
PDHA2	0.004077086	0.092619807
PDHB	-0.29827774	2.06E-07
PDHX	0.61818252	2.59E-22
PDIA2	-3.12842782	1.26E-77
PDIA3	2.231930249	1.13E-235
PDIA6	2.873369776	2.62605772077539e-319
PDIK1L	1.429564208	4.61E-106
PDILT	0.001936775	0.617577805
PDK1	1.628537773	2.15E-249
PDK2	-0.804735693	2.97E-15
PDK3	1.914698875	2.43E-294
PDK4	-0.167747843	0.128554959
PDLIM1	3.332923301	3.15E-232
PDLIM2	-0.389302822	3.77E-07
PDLIM3	0.779861291	2.35E-33
PDLIM4	2.229719081	7.13E-143
PDLIM5	2.026099665	2.70E-259
PDLIM7	1.701170862	2.54E-97
PDP1	0.306310876	0.003782292
PDP2	1.037443519	4.95E-81
PDPK1	-0.521855609	9.43E-11

PDPR	0.508685661	1.68E-11
PDRG1	1.66503474	8.32E-105
PDS5A	0.860983495	1.26E-40
PDS5B	0.322760322	3.14E-06
PDSS1	1.346988367	1.40E-102
PDX1	0.305438635	3.64E-97
PDXDC1	0.34604094	2.20E-06
PDXK	0.233733627	0.000463139
PDXP	-2.398622732	8.16E-112
PDYN	-0.363521585	0.048271742
PDZD11	2.237201555	6.47E-298
PDZD2	-0.131632733	0.082860458
PDZD3	0.066388629	9.08E-12
PDZD4	-1.395094572	7.90E-35
PDZD7	-2.123030778	8.89E-87
PDZD8	0.101162364	0.043280262
PDZD9	-0.201732889	6.78E-05
PDZK1	-0.152420313	0.236709388
PDZK1IP1	1.515015071	4.15E-271
PDZRN3	1.7605357	5.27E-119
PDZRN4	0.424155778	5.97E-18
PEA15	1.194918067	2.97E-79
PEAK1	0.72599879	1.79E-50
PEAR1	0.597157709	2.64E-30
PEBP1	0.382012979	1.15E-10
PEBP4	-0.574766764	2.60E-11
PECAM1	2.310477172	1.32E-168
PECR	1.68085264	9.87E-233
PEF1	1.337503738	3.48E-113
PEG10	0.315342045	0.016598287
PEG3	-3.176279403	3.88E-177
PELI1	1.470649022	5.03E-90
PELI2	0.66938967	6.14E-19
PELI3	-1.078022028	3.83E-35
PELO	1.553419824	1.27E-188
PELP1	0.478410604	2.89E-16
PEMT	1.001083902	6.80E-112
PENK	-2.161649282	3.68E-22
PEPD	1.698986748	6.69E-195
PER1	1.199739112	9.75E-43
PER2	0.366941099	1.11E-06
PER3	-1.313595992	2.73E-41
PERM1	0.002340146	0.898931006
PERP	0.132917445	0.02882259
PES1	1.696474779	7.15E-198
PET100	-1.704509013	1.01E-209
PET117	-1.809493808	1.04E-228
PEX1	0.150674678	0.051417931

PEX10	1.114608069	6.91E-110
PEX11A	1.473353585	3.14E-195
PEX11B	1.240995821	4.01E-59
PEX11G	1.600008662	1.31E-194
PEX12	2.038020074	2.99E-300
PEX13	1.750215461	7.64E-275
PEX14	0.84478984	9.30E-39
PEX16	0.784453154	3.79E-53
PEX19	1.091638537	1.16E-85
PEX26	0.593853157	5.40E-29
PEX3	0.993606243	1.17E-68
PEX5	0.703584583	3.17E-40
PEX5L	-1.603693111	1.09E-49
PEX6	1.078421008	4.93E-50
PEX7	0.398715168	6.16E-16
PF4	0.755813979	5.40E-180
PF4V1	0.287930211	3.11E-70
PFAS	1.821812539	3.70E-139
PFDN1	0.918780254	1.44E-56
PFDN2	0.993796162	4.16E-68
PFDN4	1.134070063	6.15E-80
PFDN6	0.607711668	7.27E-31
PFKFB1	0.404856098	9.40E-120
PFKFB2	1.198515049	8.83E-55
PFKFB3	1.439239197	2.72E-93
PFKFB4	1.885677081	1.38E-194
PFKL	0.214163286	6.28E-05
PFKM	0.542406559	2.85E-10
PFKP	-0.33966275	0.000251713
PFN2	1.040695137	1.84E-36
PFN3	-0.019830191	0.072481852
PGA3	-0.097540814	9.76E-06
PGA4	0.002630555	0.076866871
PGA5	-0.027556299	0.424844073
PGAM1	1.248339623	4.13E-70
PGAM5	1.977747201	2.61E-168
PGAP1	1.329908037	1.32E-64
PGAP2	1.513080781	6.52E-169
PGAP3	0.956582574	2.42E-57
PGBD1	1.19276857	7.72E-58
PGBD2	1.137995712	1.10E-131
PGBD4	0.851841341	1.48E-86
PGBD5	-0.607902272	8.54E-11
PGC	-0.758034581	1.03E-19
PGD	2.170384142	1.10E-241
PGF	1.654637513	3.25E-137
PGGHG	0.818306782	3.58E-35
PGGT1B	1.470505137	5.50E-212

PGK1	2.665965841	1.48E-198
PGK2	0.109895444	1.08E-90
PGLS	1.053992296	1.19E-133
PGLYRP1	-0.013109543	0.763569801
PGLYRP2	0.023253202	1.50E-20
PGLYRP3	-0.023171052	4.80E-05
PGLYRP4	0.00589102	0.064160805
PGM1	2.068221784	5.33E-263
PGM2L1	1.087605898	1.64E-22
PGM3	1.21446274	4.42E-80
PGM5	1.008528142	2.45E-50
PGP	-0.564901355	1.67E-08
PGPEP1	1.633216394	2.17E-218
PGPEP1L	-0.291611602	2.37E-32
PGR	-0.380099991	3.74E-36
PGRMC1	1.338875891	1.95E-62
PGRMC2	1.544357876	3.47E-152
PGS1	0.565537791	1.19E-18
PHACTR1	-1.472553552	1.93E-44
PHACTR2	0.539347863	9.86E-46
PHACTR3	-2.244960018	3.55E-303
PHACTR4	1.7707755	2.72E-214
PHAX	1.512803498	1.16E-141
PHB	0.185363838	0.000292831
PHB2	1.287648375	1.15E-106
PHC1	0.656213404	2.20E-11
PHC2	2.39827531	3.44E-252
PHC3	1.317993328	2.22E-93
PHEX	1.59988748	2.30E-281
PHF1	0.058340029	0.333710407
PHF10	0.434639478	2.89E-19
PHF11	1.033032962	2.23E-81
PHF12	1.185038175	1.97E-72
PHF13	1.812411604	1.51E-213
PHF14	0.863448594	2.14E-82
PHF2	1.042274506	7.18E-42
PHF20	1.183055537	1.74E-70
PHF20L1	-0.048297899	0.468685252
PHF21A	0.415102137	1.76E-15
PHF21B	0.747640503	5.77E-48
PHF23	2.043680634	3.46E-233
PHF24	-2.128115485	1.86E-64
PHF3	-0.114299214	0.05812886
PHF5A	2.273918288	7.84E-292
PHF6	1.582136235	4.72E-132
PHF7	0.522791146	7.49E-23
PHF8	1.20592358	1.51E-66
PHGDH	0.859675408	9.10E-39

PHGR1	-0.173488773	3.10E-10
PHIP	0.376288691	1.35E-06
PHKA1	1.291764955	1.10E-90
PHKA2	0.28585168	5.77E-05
PHKB	1.32551395	2.60E-112
PHKG1	2.216113619	2.50E-173
PHKG2	0.077601277	0.233229606
PHLDA2	1.998804745	5.07E-135
PHLDA3	0.994717403	2.99E-50
PHLDB1	-0.826069774	2.50E-26
PHLDB2	0.717274074	9.82E-72
PHLDB3	1.026195141	1.39E-138
PHLPP1	1.105697725	1.63E-100
PHLPP2	-0.50425403	3.25E-10
PHOSPHO1	0.354197657	3.38E-09
PHOSPHO2	0.941079097	1.16E-86
PHOX2A	0.163638562	8.78E-37
PHOX2B	0.062205502	4.27E-09
PHPT1	1.640017018	2.28E-136
PHRF1	0.871591903	4.62E-29
PHTF1	0.766136226	3.07E-13
PHTF2	1.451305166	2.93E-110
PHYH	0.610509274	2.33E-24
PHYHD1	-0.116594796	0.091619678
PHYHIP	-3.411017611	6.77E-100
PHYHIPL	-0.130361996	0.055653142
PHYKPL	1.161903887	7.18E-101
PI15	0.584133442	5.22E-30
PI16	1.800765584	4.56E-63
PI4K2A	0.212452668	0.002158479
PI4KA	-0.589003955	1.53E-07
PI4KB	1.414986691	9.51E-88
PIANP	-0.659108093	1.09E-09
PIAS1	0.544238711	6.63E-15
PIAS2	0.726367335	2.86E-29
PIAS3	0.631803739	8.40E-15
PIAS4	0.807934076	1.61E-25
PIBF1	1.287680204	3.55E-153
PICALM	0.881282955	1.08E-48
PICK1	-1.035401433	3.56E-38
PID1	1.683379332	1.10E-101
PIDD1	0.183313943	0.035251887
PIEZO1	0.767643166	1.55E-30
PIEZO2	-0.06364177	0.416075724
PIF1	1.644989752	2.65E-278
PIFO	2.684990768	1.26E-191
PIGA	0.520523861	8.72E-24
PIGB	0.574635464	9.36E-38

PIGBOS1	1.358794634	2.68E-128
PIGC	2.02602989	6.40E-305
PIGF	1.184072964	6.81E-183
PIGG	1.193258872	1.07E-71
PIGH	0.879303181	1.80E-39
PIGK	1.561922223	2.03E-138
PIGL	0.378171137	2.59E-10
PIGM	1.770326075	1.91E-154
PIGN	1.460091016	3.89E-292
PIGO	1.365919944	9.62E-90
PIGP	1.208347247	5.75E-150
PIGQ	0.437172522	1.31E-12
PIGR	0.170974008	1.07E-14
PIGS	1.034986524	5.64E-45
PIGT	1.781940003	1.35E-158
PIGU	1.876852798	2.64E-214
PIGV	0.765324579	1.98E-72
PIGW	1.964422336	1.59E-282
PIGX	1.17054768	1.23E-96
PIGZ	-0.297270565	0.003144698
PIH1D1	0.939801519	1.25E-45
PIH1D2	0.996158151	6.61E-96
PIK3C2A	0.798904628	3.55E-34
PIK3C2B	0.37503669	2.03E-08
PIK3C2G	0.105077955	9.58E-25
PIK3C3	0.687139745	1.32E-26
PIK3CA	0.185164972	0.00195763
PIK3CB	0.318652546	4.96E-05
PIK3CD	0.80625301	1.08E-42
PIK3IP1	0.320979061	5.54E-07
PIK3R1	1.065930698	4.64E-56
PIK3R3	0.747881115	1.41E-12
PIK3R4	1.38472664	4.52E-109
PIK3R5	2.111064078	1.74E-300
PIK3R6	1.290938781	6.10E-152
PIKFYVE	0.777999833	6.09E-31
PILRA	1.341625515	2.25E-40
PILRB	-3.915566398	2.83E-214
PIM1	2.093025677	5.29E-112
PIM2	0.878764054	1.10E-26
PIM3	1.068671161	3.93E-57
PIN1	-0.203513239	0.006148592
PIN4	1.107746856	3.15E-126
PINK1	-0.661251886	3.01E-31
PINLYP	0.541150328	7.64E-27
PINX1	0.769610903	6.84E-46
PIP	-0.265125322	1.50E-09
PIP4K2A	-0.668973471	2.17E-11

PIP4K2B	0.313252239	5.70E-06
PIP4K2C	1.196383412	1.47E-42
PIP5K1A	1.774679461	9.44E-142
PIP5K1B	-0.911503708	1.89E-16
PIP5K1C	0.16836763	0.156969051
PIP5KL1	0.110129215	0.040994825
PIR	0.907114432	3.58E-43
PIRT	2.090737448	1.46E-85
PISD	-0.113032563	0.387597413
PITHD1	0.480892501	2.05E-09
PITPNA	0.876958147	6.02E-36
PITPNB	0.532292782	1.80E-18
PITPNC1	0.854201978	2.75E-48
PITPNM1	-0.632782752	8.76E-12
PITPNM2	-0.330069446	0.000432203
PITPNM3	-2.41679868	2.61E-64
PITRM1	-0.965993001	8.20E-45
PITX2	0.512240733	1.76E-37
PITX3	-0.007545657	0.860065787
PIWIL1	-0.010526043	0.137035025
PIWIL2	-0.041597371	0.348440938
PIWIL3	0.049095619	2.72E-33
PIWIL4	0.458492588	1.10E-19
PJA1	1.541794546	1.39E-80
PJA2	1.144449006	1.12E-46
PKD1	-1.508413825	1.68E-21
PKD1L1	0.191762485	2.02E-22
PKD1L3	0.10036268	3.51E-35
PKD2	1.102089142	2.90E-58
PKD2L1	0.410525166	8.91E-11
PKD2L2	0.016805214	0.058136408
PKDCC	0.389138676	9.85E-05
PKDREJ	0.070674766	1.00E-07
PKHD1	0.009604322	2.97E-10
PKHD1L1	-0.027757266	0.009421374
PKIA	0.396354991	8.36E-06
PKIB	1.524693282	1.02E-16
PKIG	1.666248746	2.08E-168
PKLR	-0.426815038	2.54E-15
PKM	0.876677555	6.05E-34
PKMYT1	1.936339632	2.81E-167
PKN1	1.625476077	1.27E-174
PKN2	1.672150981	5.26E-127
PKN3	1.190201229	9.60E-33
PKNOX1	0.680977135	7.06E-20
PKNOX2	0.459555154	6.53E-07
PKP1	-0.068046676	0.061419868
PKP2	-0.502500414	3.71E-16

PKP3	-0.155757486	6.69E-06
PKP4	-1.608269613	9.50E-78
PLA1A	-0.965243126	1.29E-23
PLA2G10	0.231063875	3.07E-30
PLA2G12A	0.315361675	3.57E-09
PLA2G12B	0.015767641	0.127344047
PLA2G15	1.258089306	2.98E-57
PLA2G1B	-0.06740487	0.332043745
PLA2G2C	0.63851367	8.49E-274
PLA2G2D	0.217887952	1.61E-190
PLA2G2E	0.009172644	0.244010354
PLA2G2F	0.001514494	0.662176648
PLA2G3	-0.165190953	1.56E-08
PLA2G4B	-4.533308677	2.00E-214
PLA2G4C	-0.451115205	1.75E-11
PLA2G4D	-0.152334264	2.16E-18
PLA2G4E	-0.076918768	4.59E-15
PLA2G4F	-0.155240448	4.09E-09
PLA2G5	2.002432315	5.42E-158
PLA2G6	-0.722815394	5.30E-37
PLA2G7	1.025749069	2.40E-55
PLA2R1	-0.11473477	0.213433593
PLAA	1.166365206	9.50E-79
PLAC1	0.481498789	4.80E-214
PLAC8	1.563313051	4.32E-271
PLAC8L1	-0.747103719	7.94E-46
PLAC9	0.52026313	6.71E-29
PLAG1	-0.004866814	0.938911931
PLAGL1	0.244878745	1.33E-05
PLAGL2	1.892958989	6.32E-211
PLAT	1.664587597	2.74E-148
PLAUR	3.005682867	2.36E-295
PLB1	1.255953417	2.52E-142
PLBD2	1.179697904	4.22E-55
PLCB1	-0.209119012	0.017399758
PLCB2	1.146504758	1.82E-35
PLCB3	1.544243534	3.04E-112
PLCB4	-0.982253439	4.64E-13
PLCD1	1.734440237	2.14E-169
PLCD3	1.419352065	4.85E-122
PLCD4	-0.79745813	1.67E-60
PLCE1	1.378521706	1.03E-233
PLCG1	1.159145107	1.95E-34
PLCG2	1.021831554	9.35E-96
PLCH1	-0.322418288	1.28E-05
PLCH2	-3.216927845	6.47E-115
PLCL1	-0.728287692	6.67E-19
PLCL2	0.285564436	0.005589413

PLCXD1	0.741343001	8.21E-15
PLCXD2	-0.352370241	1.47E-06
PLCXD3	-1.669127298	3.57E-38
PLCZ1	0.053981344	3.20E-35
PLD1	0.243786673	0.001364877
PLD2	0.879110195	3.59E-37
PLD3	0.147626039	0.089917788
PLD5	-0.058118448	0.49424059
PLD6	-0.813099914	7.31E-19
PLEC	0.713202611	1.52E-22
PLEK	2.686951961	1.17E-168
PLEKHA1	-0.900195857	1.99E-46
PLEKHA2	1.892472179	2.30E-119
PLEKHA3	0.748456376	9.80E-55
PLEKHA5	0.143204889	0.038823495
PLEKHA6	-1.499458214	1.43E-70
PLEKHA7	0.621090787	6.62E-32
PLEKHA8	0.930634292	1.18E-52
PLEKHB1	-0.973176347	1.96E-32
PLEKHB2	0.869805847	4.72E-33
PLEKHD1	-0.928805249	1.40E-14
PLEKHF1	1.615775688	1.51E-201
PLEKHG1	1.40230152	3.15E-87
PLEKHG3	-0.927418385	5.20E-37
PLEKHG4	-0.006089819	0.908739893
PLEKHG4B	0.405314381	8.04E-45
PLEKHG5	-1.268992176	1.01E-14
PLEKHG6	0.354049385	3.58E-257
PLEKHG7	0.054907224	2.05E-26
PLEKHH1	-2.103999463	9.33E-79
PLEKHH2	0.145481959	0.007804903
PLEKHH3	1.402678709	2.87E-67
PLEKHJ1	0.340819007	7.41E-12
PLEKHM1	0.627240634	1.75E-19
PLEKHM2	0.250085898	0.00103292
PLEKHM3	-0.025911107	0.725857148
PLEKHN1	0.129546108	1.38E-11
PLEKHO1	0.700614472	1.30E-24
PLEKHO2	1.737824201	3.40E-176
PLET1	0.032000664	1.97E-05
PLG	-0.044647912	8.46E-11
PLGLB1	-0.730277748	1.24E-21
PLGLB2	-0.045064744	0.027880611
PLIN1	-0.805699665	3.97E-29
PLIN2	2.932208042	2.78E-295
PLIN3	3.283314522	1.21E-259
PLIN4	-1.303063551	3.67E-91
PLIN5	-1.112194033	1.27E-63

PLK1	2.031408488	5.24E-151
PLK2	0.02411139	0.873082742
PLK3	2.137119413	5.17E-229
PLK5	-2.077092154	4.28E-55
PLLP	-0.09037095	0.310552939
PLN	0.453363144	5.15E-12
PLOD2	2.260400851	1.61E-273
PLOD3	2.488944088	1.80E-235
PLP1	-1.260861463	5.31E-18
PLP2	3.36571573	1.28E-265
PLPP1	0.891420568	9.27E-48
PLPP2	-1.647235019	1.35E-55
PLPP3	1.262731292	2.59E-48
PLPP4	-0.062355645	0.465259508
PLPP5	1.293361632	2.78E-82
PLPP6	0.738580733	2.27E-21
PLPP7	-0.132862003	0.081783283
PLPPR1	1.033353306	2.96E-29
PLPPR2	0.790568781	2.68E-29
PLPPR3	-1.650329738	4.09E-26
PLPPR4	1.042070504	3.82E-18
PLPPR5	0.768204222	1.66E-17
PLRG1	1.135012145	1.21E-83
PLS1	0.609563553	1.00E-50
PLS3	2.511312931	2.51E-222
PLSCR1	2.692946042	6.04E-240
PLSCR2	0.108421038	1.29E-56
PLSCR4	1.329042281	8.57E-94
PLSCR5	-0.049570565	2.93E-06
PLXDC1	0.708517064	1.03E-30
PLXDC2	2.070281271	8.36E-186
PLXNA1	0.896747962	1.06E-21
PLXNA2	0.29707956	0.002585113
PLXNA3	1.353866676	2.27E-36
PLXNA4	1.19413928	3.87E-43
PLXNB1	0.378390472	3.55E-10
PLXNB2	1.534752197	7.24E-39
PLXNB3	-0.002416241	0.982299793
PLXNC1	0.969260536	1.77E-24
PLXND1	1.93874561	2.86E-132
PM20D1	0.178348642	3.39E-22
PM20D2	1.313632088	2.66E-63
PMAIP1	1.336833301	1.75E-103
PMCH	-0.748981265	3.64E-06
PMEL	0.780152723	6.70E-159
PMEPA1	1.642543645	1.73E-75
PMF1	0.239500899	3.29E-06
PMFBP1	0.551926726	1.07E-84

PML	1.31764745	2.65E-132
PMM1	-1.025140851	2.60E-60
PMM2	0.558444518	1.06E-34
PMP2	2.37672282	2.91E-144
PMP22	-0.0456209	0.6313778
PMPCA	0.855182716	1.53E-47
PMPCB	1.064282417	3.27E-54
PMS1	1.13998705	4.42E-72
PMS2	0.64003276	8.06E-18
PMVK	1.569767197	2.63E-225
PNCK	-1.885576546	6.95E-66
PNISR	-0.54279348	2.15E-08
PNKD	0.493784052	1.30E-14
PNKP	0.68899095	2.46E-26
PNLDC1	-0.747927692	3.87E-24
PNLIP	-0.797769472	1.36E-16
PNLIPRP1	-0.147942036	6.58E-08
PNLIPRP3	0.160922119	1.60E-117
PNMA1	1.147248867	9.77E-48
PNMA2	0.872636335	4.21E-14
PNMA3	-2.349335961	3.34E-83
PNMA5	-1.444921946	5.55E-24
PNMA6A	-1.071828092	3.98E-31
PNMT	-1.656157421	3.97E-55
PNN	0.073133983	0.362879675
PNO1	2.146085668	1.26E-264
PNOC	0.098085551	0.301234255
PNP	1.159053416	2.04E-55
PNPLA1	0.070202647	2.67E-10
PNPLA2	-0.004443782	0.961907814
PNPLA3	0.574322655	1.63E-61
PNPLA4	0.740630275	2.73E-39
PNPLA5	-0.81430437	2.72E-30
PNPLA6	0.686468791	9.65E-28
PNPLA7	-0.831464216	6.43E-31
PNPLA8	1.722131511	1.04E-206
PNPO	0.47712586	2.04E-13
PNPT1	1.233866587	1.72E-88
PNRC1	1.569842116	1.11E-112
PNRC2	1.330217492	9.17E-127
POC1B	0.618442574	8.70E-29
POC5	1.11935933	3.32E-81
PODN	1.216276878	2.15E-96
PODNL1	0.687017106	1.66E-29
PODXL	1.812517502	7.65E-143
PODXL2	0.667424405	4.43E-14
POF1B	-0.02452945	0.013819979
POFUT2	1.6058468	2.44E-124

POGK	1.153579814	1.67E-103
POGLUT1	1.260972331	2.73E-101
POGZ	-0.189135061	0.043367978
POLA1	0.82777799	3.78E-70
POLA2	0.189805867	0.002332597
POLB	0.008172551	0.952724542
POLD1	1.809309284	4.60E-144
POLD2	2.275209144	5.53353523342196e-322
POLD3	1.968290841	5.83E-269
POLD4	-1.13674124	8.30E-122
POLDIP2	1.492150103	2.03E-146
POLDIP3	1.610057258	2.90E-139
POLE	0.303202165	0.041950108
POLE3	2.35607311	1.93E-179
POLE4	1.417327211	1.75E-96
POLG	0.405556631	1.17E-07
POLG2	1.150796632	1.89E-69
POLH	1.774365308	6.69E-173
POLI	0.568962054	2.21E-27
POLK	0.507202517	2.19E-18
POLL	0.507813301	8.14E-22
POLM	1.251317687	1.29E-58
POLN	-0.750440621	4.36E-58
POLR1A	1.667889159	7.18E-178
POLR1B	1.932942078	7.73E-265
POLR1C	0.379746254	5.19E-11
POLR1D	0.825202378	6.37E-30
POLR1E	1.938656916	2.79E-239
POLR2A	1.096937596	7.03E-36
POLR2B	1.222855799	3.08E-46
POLR2C	1.178260886	4.25E-62
POLR2D	1.987945871	2.99E-270
POLR2E	0.792617795	2.42E-43
POLR2F	1.700864058	5.47E-141
POLR2H	1.43396663	9.04E-110
POLR2I	-1.711767034	5.59E-233
POLR2J2	0.00716111	0.823758545
POLR2J3	0.351435388	2.02E-16
POLR2K	1.488763014	4.17E-127
POLR2M	0.537943106	4.41E-30
POLR3A	0.5557962	4.72E-11
POLR3B	1.450560722	2.49E-160
POLR3C	1.751780696	5.17E-120
POLR3D	1.983729597	6.63E-254
POLR3E	-0.017507321	0.814674816
POLR3F	1.194775259	2.85E-47
POLR3G	0.573308309	2.99E-21
POLR3GL	0.12346054	0.007521374

POLR3H	-0.630093891	1.34E-43
POLR3K	1.338133097	1.13E-92
POLRMT	0.859436503	2.99E-37
POM121	1.461730329	8.51E-69
POM121C	1.464609447	7.24E-81
POM121L12	-0.001860436	0.217816865
POM121L2	-0.016631587	0.035247985
POMC	-0.676374747	1.10E-14
POMGNT1	1.475113729	1.46E-128
POMGNT2	1.568716674	8.16E-67
POMK	2.207373344	4.97362225005249e-313
POMP	1.656964318	8.77E-226
POMT1	0.444307604	1.80E-07
POMT2	0.785231189	9.19E-39
POMZP3	1.012762113	5.88E-45
PON1	-0.239431436	2.35E-19
PON2	1.652906288	3.53E-127
PON3	-0.440575225	3.58E-20
POP1	1.154136388	9.94E-71
POP4	2.011253986	7.86E-294
POP5	1.737028096	3.19E-298
POP7	2.049849302	2.72E-148
POPDC2	1.141110299	5.91E-89
POPDC3	-0.606841144	3.60E-10
POR	2.0860904	2.53E-256
PORCN	0.450404486	6.65E-08
POT1	1.702751516	1.87E-194
POTEB	-0.000493343	0.423189503
POTEB2	-5.88E-05	0.919727334
POTEB3	0.001531849	0.022054588
POTEC	-0.000682364	0.481788574
POTED	0.001500841	0.017061408
POTEH	-0.000910187	0.530814149
POU1F1	-0.057551063	1.33E-05
POU2AF1	-0.103436296	0.002552512
POU2F1	0.268187127	0.000183087
POU2F2	0.590018531	7.86E-12
POU2F3	0.153305064	8.70E-44
POU3F1	-0.017838533	0.838043012
POU3F2	2.6886427	1.64E-295
POU3F3	1.282759894	1.23E-127
POU3F4	1.45202691	1.48E-38
POU4F1	0.786675245	3.77E-128
POU4F2	0.105624871	9.81E-10
POU4F3	0.28618185	3.58E-105
POU5F1	0.225314984	4.41E-15
POU5F1B	0.148440422	6.12E-85
POU5F2	-0.016489655	0.002595965

POU6F1	-0.075249309	0.390839961
POU6F2	-0.173102582	4.63E-05
PP2D1	0.231788916	8.20E-20
PPA1	-1.047190559	2.12E-41
PPA2	1.555605307	1.85E-206
PPAN	-1.259252787	5.07E-70
PPARA	0.376064582	6.42E-18
PPARD	1.310499186	6.74E-104
PPARG	0.509959141	4.98E-23
PPARGC1A	0.405912004	1.07E-10
PPARGC1B	-0.414692836	2.05E-07
PPAT	1.392356177	2.34E-122
PPBP	1.155770189	2.84E-218
PPCDC	1.522915684	1.74E-274
PPCS	0.919310425	4.48E-70
PPDPF	1.389368087	5.98E-114
PPEF1	0.29441793	0.000313482
PPEF2	-0.254080501	1.61E-12
PPFIA1	0.837403428	1.52E-51
PPFIA2	-1.608183588	7.00E-91
PPFIA3	-1.449282136	4.92E-40
PPFIA4	-1.953560045	1.93E-30
PPFIBP1	0.821293524	9.29E-29
PPFIBP2	0.288425372	1.29E-08
PPHLN1	0.891354206	4.54E-39
PPIA	1.127850791	1.15E-72
PPIAL4A	0.603360018	3.79E-286
PPIAL4C	0.292756781	2.64E-26
PPIAL4D	0.139444241	3.17E-21
PPIAL4E	0.124336617	5.52E-38
PPIB	1.262089133	2.65E-108
PPID	0.625800268	2.40E-19
PPIE	1.1147678	6.36E-97
PPIF	1.544200525	3.78E-131
PPIG	0.326047341	1.16E-07
PPIH	0.326946524	8.18E-08
PPIL1	2.639317897	1.97E-281
PPIL2	0.421961934	2.13E-08
PPIL3	1.235369469	3.25E-95
PPIL4	1.365791044	3.87E-137
PPIL6	1.009122128	4.43E-80
PPIP5K1	-0.666386924	3.54E-09
PPIP5K2	0.8325672	2.75E-35
PPL	0.174014023	0.121892448
PPM1A	0.46920582	3.56E-12
PPM1B	-0.918262847	4.66E-58
PPM1D	1.787361219	5.76E-84
PPM1E	0.705260437	2.98E-12

PPM1F	0.21039193	0.000959468
PPM1G	1.717148448	4.31E-165
PPM1H	-1.280303659	4.48E-34
PPM1J	-0.921545908	2.80E-20
PPM1K	-0.340115643	5.95E-07
PPM1L	0.139350758	0.079400261
PPM1M	1.501569834	3.21E-138
PPM1N	0.324483033	1.27E-13
PPME1	-0.418045576	4.58E-07
PPOX	0.348017753	5.70E-08
PPP1CA	1.404150182	4.90E-117
PPP1CB	2.277615182	3.27E-291
PPP1CC	1.540291561	7.20E-147
PPP1R10	1.519070465	1.28E-108
PPP1R11	1.44352932	2.93E-126
PPP1R12A	0.303844493	2.80E-07
PPP1R12B	-0.778564115	1.69E-29
PPP1R12C	-1.367562963	5.30E-96
PPP1R13B	-0.99621	2.59E-29
PPP1R13L	1.322952182	8.18E-141
PPP1R14A	0.083739965	0.501393737
PPP1R14C	0.669150478	1.38E-13
PPP1R14D	0.215410456	3.89E-29
PPP1R15A	1.185729611	3.73E-39
PPP1R15B	1.996600855	1.16E-188
PPP1R16A	-0.808462626	7.46E-37
PPP1R16B	-2.557349598	4.60E-160
PPP1R17	-0.259224651	0.051259306
PPP1R1A	-2.378613014	1.00E-93
PPP1R1B	-3.515575562	1.34E-140
PPP1R1C	1.760189773	1.59E-253
PPP1R2	0.865382182	6.40E-39
PPP1R21	0.54120516	1.98E-20
PPP1R26	0.683576201	2.10E-26
PPP1R27	0.00094785	0.962986313
PPP1R32	0.072176913	0.336178025
PPP1R35	0.776183103	1.04E-24
PPP1R36	0.517811122	3.46E-22
PPP1R37	-0.474028467	1.40E-08
PPP1R3A	-0.012415085	0.000822942
PPP1R3C	0.305310175	0.001409591
PPP1R3D	0.74453761	1.67E-55
PPP1R3E	-1.735523593	3.61E-86
PPP1R3F	-1.198209387	1.18E-52
PPP1R3G	0.777492793	1.62E-41
PPP1R42	0.988436338	1.53E-90
PPP1R7	-0.006059323	0.938694652
PPP1R8	2.057113099	2.71E-252

PPP1R9A	-0.384632342	1.21E-09
PPP1R9B	-0.282225512	0.000517198
PPP2CA	0.966007603	2.33E-41
PPP2CB	0.79890372	5.73E-61
PPP2R1A	0.771570095	1.17E-25
PPP2R1B	0.723154539	2.82E-29
PPP2R2A	0.895429242	1.65E-57
PPP2R2B	0.589977499	3.14E-16
PPP2R2D	0.16540848	0.026666836
PPP2R3A	0.698672961	3.94E-51
PPP2R3B	-0.016330543	0.777729414
PPP2R3C	0.658369864	5.86E-40
PPP2R5A	0.974637102	1.80E-96
PPP2R5B	-0.018608604	0.853889946
PPP2R5C	0.972878862	2.98E-80
PPP2R5D	-0.257242532	0.004697461
PPP2R5E	1.041671847	3.75E-55
PPP3CA	-0.512255151	1.30E-05
PPP3CB	-0.270867498	0.006758891
PPP3CC	1.300838746	1.05E-79
PPP3R1	-0.778435997	1.91E-15
PPP3R2	-0.012671918	0.001320465
PPP4C	2.382152975	6.03E-288
PPP4R1	1.134252525	4.13E-93
PPP4R2	1.411712986	9.22E-141
PPP4R3A	0.945415544	8.35E-42
PPP4R3B	1.540187104	2.32E-130
PPP4R4	-1.768626877	2.11E-80
PPP5C	0.298752173	8.26E-05
PPP6C	1.491126235	1.47E-146
PPP6R1	0.020577763	0.777944924
PPP6R2	0.247675662	0.002613145
PPP6R3	0.798575957	5.30E-30
PPRC1	1.283992584	4.03E-82
PPT1	2.38273693	3.52E-260
PPT2	-0.318121696	0.000206231
PPTC7	0.546500403	2.50E-15
PPWD1	0.242655327	0.000480696
PPY	0.422417332	1.41E-274
PQBP1	0.589204823	9.12E-18
PRAC1	0.226104502	1.29E-40
PRAC2	0.712968855	2.53E-214
PRADC1	1.582949901	4.69E-267
PRAF2	0.79554676	2.08E-43
PRAG1	1.118061511	1.39E-15
PRAM1	1.392903348	9.39E-81
PRAME	0.813930113	1.12E-121
PRAMEF1	0.010875739	0.109111127

PRAMEF10	-0.001185823	0.336974175
PRAMEF11	-0.000705664	0.764427463
PRAMEF12	0.029577718	2.68E-06
PRAMEF13	0.000119659	0.926396658
PRAMEF14	0.001744289	0.77250281
PRAMEF15	-0.000396645	0.882661005
PRAMEF17	-0.00112219	0.623196911
PRAMEF18	0.000181666	0.919848148
PRAMEF19	-0.000317027	0.929763183
PRAMEF2	0.002315435	0.817601175
PRAMEF20	-0.001198139	0.777920318
PRAMEF25	0.001781084	0.012238155
PRAMEF26	0.000161207	0.77261665
PRAMEF27	-0.000874168	0.697371913
PRAMEF33	-0.000290327	0.76041125
PRAMEF4	0.002139703	0.32604557
PRAMEF5	0.001622673	0.130100707
PRAMEF6	-0.000628805	0.619976703
PRAMEF7	0.002576544	0.670202652
PRAMEF8	0.002939599	0.674889485
PRAMEF9	0.001543746	0.289626295
PRAP1	-0.527775816	3.04E-42
PRB1	-0.256458117	1.48E-19
PRB2	-0.718844253	4.86E-37
PRB3	0.032478379	1.33E-13
PRB4	0.001344628	0.78738065
PRC1	-1.634105533	9.49E-78
PRCC	1.076566967	1.53E-55
PRCD	-1.027024705	6.15E-23
PRDM1	1.46356002	1.75E-143
PRDM10	0.487820711	6.98E-09
PRDM11	-0.019177388	0.757222909
PRDM12	-0.200585006	1.19E-09
PRDM13	0.552489043	6.59E-108
PRDM14	0.039989977	4.13E-73
PRDM15	0.227059076	1.77E-05
PRDM16	0.307755774	2.46E-05
PRDM2	-0.061918635	0.424333188
PRDM4	0.826274859	7.80E-34
PRDM5	1.305423132	5.40E-299
PRDM6	0.126065065	1.33E-09
PRDM7	0.012291619	0.002945858
PRDM8	-0.545113507	1.47E-06
PRDM9	0.002871759	0.009654356
PRDX1	1.153929147	5.02E-90
PRDX2	0.109703467	0.118757795
PRDX3	0.524402281	9.36E-22
PRDX5	1.367170664	1.04E-86

PRDX6	1.9369477	2.52E-249
PREB	1.352261732	1.19E-97
PRELID1	0.633433266	6.15E-35
PRELID2	0.692587569	1.52E-268
PRELID3A	0.461903348	4.69E-14
PRELP	0.620232539	1.31E-19
PREP	0.423406154	2.17E-08
PREPL	-0.679353534	8.20E-13
PREX1	1.543938098	3.22E-121
PREX2	0.161593177	0.003886187
PRG2	-0.103154374	5.18E-08
PRG3	0.002250937	0.743141115
PRG4	0.25889309	2.08E-11
PRH1	-0.918907993	1.07E-74
PRH2	0.104630424	7.57E-13
PRICKLE1	0.227642231	0.000259595
PRICKLE2	-0.163819414	0.037941197
PRICKLE3	1.43125248	1.78E-294
PRICKLE4	-0.777011546	1.04E-76
PRIM1	1.214243992	2.39E-59
PRIMA1	0.773837374	4.47E-12
PRIMPOL	1.481492543	7.04E-112
PRKAA1	1.259409535	1.27E-80
PRKAA2	0.092657719	0.193084205
PRKAB1	1.363637396	5.43E-109
PRKAB2	1.05709145	7.41E-39
PRKACA	0.201450373	0.007891926
PRKACB	0.279527756	0.000821954
PRKACG	0.036909349	8.76E-20
PRKAG1	1.371124614	4.58E-127
PRKAG2	-1.201606834	3.18E-56
PRKAG3	-0.080161703	0.000667781
PRKAR1A	0.534258919	9.79E-16
PRKAR1B	-0.482460489	2.10E-05
PRKAR2A	2.24473986	1.81E-255
PRKAR2B	-0.206530112	0.014577258
PRKCA	0.743020873	4.45E-30
PRKCB	-2.152032646	9.28E-60
PRKCD	1.116479898	2.93E-38
PRKCE	-0.51357744	6.35E-06
PRKCG	-3.257729646	9.62E-93
PRKCH	0.448703424	2.59E-11
PRKCI	0.859459101	5.08E-34
PRKCQ	-0.023905232	0.739666429
PRKCSH	1.545768962	2.03E-144
PRKCZ	-2.356041779	4.40E-98
PRKD2	2.099932895	4.29917150516511e-318
PRKDC	1.926681605	9.87E-159

PRKG1	-0.150132422	0.000412866
PRKG2	-0.509317445	1.14E-25
PRKRA	-1.147350975	1.46E-78
PRKRIP1	0.89817566	3.31E-35
PRKX	1.517227127	2.23E-106
PRL	-0.741395146	1.05E-12
PRLH	-0.028612336	0.373897504
PRLHR	-0.475490506	3.93E-17
PRLR	-0.219208331	2.85E-05
PRM1	-0.441316635	7.33E-15
PRM2	-0.488377115	2.29E-17
PRM3	-0.012889808	0.030329276
PRMT1	1.643588271	2.47E-124
PRMT2	1.128383794	5.43E-91
PRMT3	1.520759496	9.62E-192
PRMT5	1.00992004	3.11E-45
PRMT6	1.692794342	5.59E-148
PRMT7	0.41562767	1.95E-06
PRMT8	-2.326068436	1.32E-69
PRMT9	1.09854376	1.34E-88
PRND	0.376634868	5.44E-72
PRNP	0.435838718	8.05E-08
PROB1	1.041391461	1.83E-116
PROC	0.464642095	5.87E-42
PROCA1	0.060695092	0.310157321
PROCR	2.450995938	6.27E-279
PRODH	-2.707389639	1.97E-195
PRODH2	-0.07016023	2.64E-15
PROK1	0.576027806	2.48E-72
PROK2	0.44321917	5.19E-30
PROKR1	0.009262794	0.660716005
PROKR2	-0.035239397	0.175523709
PROM1	1.91644434	2.73E-280
PROM2	-0.505604465	2.04E-19
PROP1	-0.069967516	2.85E-06
PROSER1	1.04269366	2.45E-36
PROSER2	0.259409985	3.52E-24
PROSER3	1.297155938	2.09E-140
PROX1	0.993824942	1.36E-32
PROX2	-0.055666706	0.068223937
PROZ	-0.093804805	0.024310412
PRPF18	-0.686744235	2.11E-36
PRPF19	1.063907943	3.78E-34
PRPF3	1.038477208	7.18E-44
PRPF31	0.666843428	1.11E-29
PRPF38A	1.652745446	4.95E-176
PRPF38B	0.48908862	7.72E-08
PRPF39	-0.343538708	0.000191116

PRPF4	1.965903674	5.88E-192
PRPF40A	1.592388961	7.24E-153
PRPF40B	-0.81260154	3.03E-34
PRPF4B	0.906863833	3.80E-31
PRPF6	1.574078341	6.72E-78
PRPF8	1.033813174	1.04E-29
PRPH	1.268547495	2.08E-69
PRPH2	-0.601183819	1.70E-21
PRPS1	1.612242058	3.04E-88
PRPS1L1	0.159428653	1.87E-218
PRPS2	2.053115373	2.81E-128
PRPSAP1	1.24225515	9.26E-108
PRPSAP2	1.027499194	9.14E-46
PRR12	0.63793895	3.12E-11
PRR13	-0.155004559	0.006110534
PRR14	0.99769843	2.40E-36
PRR14L	0.61680818	1.02E-18
PRR15	0.941748411	1.46E-230
PRR15L	0.000904499	0.955909655
PRR16	0.595959266	6.87E-16
PRR18	-0.764367772	8.88E-14
PRR19	0.935702607	7.48E-69
PRR20A	0.000369827	0.463605033
PRR20B	0	1
PRR20C	0	1
PRR20D	0	1
PRR20E	-1.06E-05	0.985197762
PRR22	0.450121166	4.84E-20
PRR23A	0.001830583	0.298099991
PRR23B	0.003717992	0.000401864
PRR23C	0.003981006	0.000109676
PRR23D1	0.001970736	0.002305521
PRR23D2	0.000160073	0.82845684
PRR27	0.000433511	0.718941569
PRR29	-0.238722366	0.011317503
PRR3	0.783639115	1.16E-28
PRR30	-0.011674582	0.023000081
PRR35	-1.48390743	1.30E-09
PRR36	-0.816585834	2.46E-11
PRR4	-1.648443416	1.56E-201
PRR5	-0.335314678	2.78E-06
PRR5L	1.844075854	1.18E-120
PRR7	0.67709151	6.54E-20
PRR9	-0.006904322	0.196607292
PRRC1	2.041660194	2.49E-288
PRRC2A	0.936574156	1.67E-37
PRRC2B	0.583117158	3.07E-11
PRRC2C	0.460577016	1.42E-12

PRRG1	0.275810562	0.002952685
PRRG3	-0.964459413	2.51E-21
PRRG4	1.406855194	4.19E-244
PRRT1	-2.02442011	3.09E-72
PRRT2	-2.666205809	4.61E-67
PRRT3	-0.262994655	0.002492985
PRRT4	0.11541196	0.169822739
PRRX2	0.825983643	4.40E-85
PRSS1	-1.313114984	5.06E-23
PRSS12	0.304971271	1.57E-09
PRSS16	-0.385096516	8.58E-11
PRSS2	-1.446940722	2.64E-25
PRSS21	0.186537265	9.41E-19
PRSS22	-0.505904357	1.25E-11
PRSS23	2.145077298	1.67E-220
PRSS27	-0.132069684	0.066093334
PRSS3	-1.50828299	1.66E-33
PRSS33	0.578922106	6.01E-229
PRSS35	0.925789359	4.98E-17
PRSS36	0.714041343	5.43E-80
PRSS37	-0.067525211	0.000986559
PRSS38	-0.030440589	1.03E-05
PRSS41	-0.004133237	0.466112247
PRSS48	0.228070827	2.22E-48
PRSS53	-1.692151556	1.66E-66
PRSS54	0.253765722	1.22E-101
PRSS55	-0.016738569	0.654489951
PRSS56	-0.628324861	5.86E-30
PRSS57	0.014815905	0.106346672
PRSS58	-0.005482791	0.076323056
PRSS8	-0.362510728	4.75E-10
PRTFDC1	1.708629583	1.52E-184
PRTG	0.610277135	9.28E-104
PRTN3	0.054588198	0.456368229
PRUNE1	1.847325177	6.46E-157
PRUNE2	0.472660704	8.92E-07
PRX	-0.120961165	0.026357408
PRY	0.017316529	2.02E-27
PRY2	0.003946302	2.91E-05
PSAP	2.020115092	7.43E-166
PSAPL1	-0.044616645	1.03E-08
PSAT1	1.784353679	2.16E-109
PSCA	-0.082523004	0.005664463
PSD	-2.901649369	9.12E-102
PSD2	-0.189773669	0.010421492
PSD3	-0.799723105	2.37E-11
PSD4	1.023915519	3.78E-107
PSEN1	1.441928851	1.69E-100

PSEN2	-3.047450015	4.00588425648083e-320
PSENEN	0.328012168	6.37E-11
PSG1	0.021962671	5.31E-10
PSG11	0.050981691	1.06E-35
PSG2	-0.000457787	0.79655667
PSG3	0.000960139	0.749344884
PSG4	0.001042727	0.743502739
PSG5	-0.042949946	1.01E-07
PSG6	-0.002714418	0.282376516
PSG7	-0.005406903	0.02454774
PSG8	0.003628298	0.122726962
PSG9	-0.019858536	2.05E-05
PSIP1	0.702492272	7.50E-20
PSKH2	0.00174256	0.484234137
PSMA1	-1.08646789	4.61E-62
PSMA3	0.966082598	3.21E-66
PSMA4	1.647299641	9.40E-141
PSMA5	2.158763348	2.92E-244
PSMA7	2.544277036	1.41E-304
PSMA8	-0.001307293	0.8585229
PSMB1	2.222725323	5.10E-295
PSMB10	-0.855537632	2.26E-64
PSMB11	0.02655174	8.31E-11
PSMB2	2.370301562	2.17E-284
PSMB3	2.135986594	1.05E-255
PSMB4	1.161798298	2.30E-87
PSMB5	2.000350992	7.73E-231
PSMB6	1.393442915	1.63E-124
PSMB7	1.984948285	1.71E-204
PSMB8	2.163310914	2.45E-235
PSMB9	2.006025773	1.51E-154
PSMC1	-0.885908954	1.65E-31
PSMC2	1.031232153	1.65E-59
PSMC3	1.176190679	6.07E-100
PSMC3IP	0.633107679	9.42E-29
PSMC4	1.785821101	4.31E-150
PSMC5	-0.562712633	7.40E-22
PSMC6	0.991630369	7.45E-55
PSMD1	-0.590881013	2.66E-18
PSMD10	1.074240817	5.46E-75
PSMD11	0.867482119	1.97E-42
PSMD12	1.53815064	4.18E-130
PSMD13	1.010974814	3.93E-60
PSMD14	0.333633254	1.67E-08
PSMD2	1.842371249	3.32E-147
PSMD3	0.909215155	8.21E-32
PSMD4	0.783711499	2.29E-48
PSMD5	1.456785375	6.90E-84

PSMD6	-0.326742009	1.07E-08
PSMD7	0.404734009	1.12E-09
PSMD8	2.225117336	1.47E-210
PSMD9	-1.798452577	1.01E-268
PSME2	0.905234722	6.60E-71
PSME3	2.183510379	5.14E-271
PSME4	1.173868059	2.80E-88
PSMF1	1.171917794	7.03E-104
PSMG1	1.960165896	3.81E-175
PSMG2	1.565029355	8.75E-172
PSMG3	2.267137614	8.76E-217
PSMG4	0.363603098	2.14E-11
PSORS1C1	0.850898853	7.87E-179
PSORS1C2	0.2890956	2.22E-51
PSPC1	0.83405725	2.81E-29
PSPN	0.911154249	1.79E-50
PSRC1	2.510478872	1.53E-154
PSTK	-0.169946931	0.000231917
PSTPIP1	0.99100861	3.82E-130
PSTPIP2	1.14136358	9.95E-121
PTAR1	1.467391325	3.88E-157
PTBP1	2.074706931	1.91E-200
PTBP2	0.184228834	0.000932074
PTBP3	1.453842882	8.96E-115
PTCD1	-0.431643357	4.23E-14
PTCD2	0.860749381	3.02E-78
PTCD3	0.879586541	5.50E-52
PTCH1	0.052254712	0.625092236
PTCH2	0.203703382	0.030882415
PTCHD1	0.019241503	0.8711648
PTCHD3	0.018754919	3.50E-13
PTCHD4	0.769344702	4.73E-277
PTDSS1	1.81662628	3.53E-147
PTDSS2	0.514674368	2.74E-17
PTEN	0.398575138	3.10E-08
PTER	-0.925856758	1.09E-09
PTF1A	-0.249156953	2.09E-39
PTGDR	-0.099718072	0.000612416
PTGDR2	-0.664768957	7.51E-32
PTGER1	0.222789907	1.93E-10
PTGER2	0.460231812	1.57E-73
PTGER3	-0.280197141	1.10E-06
PTGER4	1.925650856	4.90E-216
PTGES	0.937861117	3.51E-43
PTGES2	-0.671746105	3.38E-21
PTGES3	1.879653109	8.20E-169
PTGES3L	-0.967266444	3.90E-46
PTGFR	0.018031198	0.711256277

PTGIR	0.953793849	4.35E-179
PTGIS	1.370796203	8.93E-141
PTGR1	1.602633836	8.63E-108
PTGS2	1.478348077	7.21E-83
PTH	-0.008075661	0.388693429
PTH1R	-0.525914021	3.98E-25
PTH2	-0.283085138	4.75E-07
PTH2R	-0.246587791	0.000618893
PTHLH	1.127117142	1.24E-58
PTK2	0.701755672	6.01E-37
PTK2B	-0.883730942	1.18E-09
PTK6	0.067310926	0.285026704
PTK7	2.819369249	9.86E-294
PTMA	1.121826924	3.97E-87
PTMS	2.117290043	6.80E-89
PTOV1	-0.827152013	8.40E-33
PTP4A2	0.600293064	6.10E-18
PTP4A3	1.991493391	3.57E-194
PTPA	0.915445612	9.04E-50
PTPDC1	-0.00986719	0.888124056
PTPMT1	-1.357067283	5.69E-185
PTPN1	1.858621427	2.76E-229
PTPN11	0.689718425	2.93E-37
PTPN12	1.620097841	1.28E-204
PTPN13	1.757415963	6.94E-151
PTPN14	1.347022328	2.88E-203
PTPN18	0.727759913	1.92E-16
PTPN2	0.847844462	9.00E-30
PTPN20	-1.206377938	2.34E-54
PTPN21	1.035904552	7.38E-111
PTPN22	0.871944733	8.90E-163
PTPN23	0.997516411	1.41E-39
PTPN3	-0.190634146	0.022804536
PTPN4	0.101156861	0.279174658
PTPN5	-2.305159005	2.97E-36
PTPN6	2.34832583	1.10E-210
PTPN7	1.284095872	6.21E-35
PTPN9	1.974357993	8.67E-262
PTPRA	0.994399929	2.12E-59
PTPRB	-0.361956903	8.21E-11
PTPRC	2.126653808	2.24E-126
PTPRD	-0.576312867	1.13E-09
PTPRE	1.066956168	1.24E-71
PTPRF	1.49296232	2.05E-105
PTPRG	1.362703314	8.11E-189
PTPRH	0.347853235	4.10E-05
PTPRJ	1.418449296	1.72E-148
PTPRK	0.886423161	1.72E-36

PTPRM	-0.364029266	5.48E-07
PTPRN	-2.095934564	5.41E-49
PTPRN2	0.383865722	0.000127799
PTPRO	0.653681938	2.48E-11
PTPRQ	-0.046611771	0.011469103
PTPRR	-1.13648706	1.73E-22
PTPRS	1.57929781	4.43E-52
PTPRT	-0.720117666	3.67E-11
PTPRU	-0.432051982	1.71E-06
PTRH1	-2.316614609	7.88E-243
PTRH2	1.595647544	2.83E-205
PTRHD1	1.269258699	1.22E-141
PTS	0.220720358	0.000367047
PTTG2	0.015285683	0.654324913
PTX4	0.04530892	0.020523924
PUDP	1.706162753	3.70E-234
PUF60	2.011165087	2.78E-258
PUM1	1.195595467	3.37E-70
PUM2	1.093605691	6.35E-49
PUM3	1.972583369	3.37E-279
PURA	0.537515154	2.62E-18
PURB	1.377286652	6.04E-62
PURG	0.288733195	2.68E-05
PUS1	0.655159826	2.60E-34
PUS10	0.774540019	3.81E-88
PUS3	1.963476805	7.46E-279
PUS7	1.716246936	1.25E-135
PUS7L	0.598632902	1.25E-19
PUSL1	0.491270745	1.82E-16
PVALB	-2.81514838	2.75E-30
PVR	0.379826191	1.81E-07
PVRIG	-1.740451425	2.24E-78
PWP1	2.129902478	4.32E-203
PWP2	0.365370012	2.51E-10
PWWP2A	0.728147786	9.31E-22
PWWP2B	-0.495785456	1.94E-11
PXDC1	2.390280768	2.23E-268
PXDN	2.761186239	8.45251953496017e-318
PXK	-0.113060884	0.20052856
PXMP2	-0.64742525	8.67E-43
PXN	0.816325942	1.90E-21
PXT1	-0.068196035	0.00493291
PYCARD	1.518032225	1.10E-67
PYCR2	-0.212133496	4.66E-05
PYDC1	-1.939220484	1.98E-56
PYDC2	0.012087577	0.000178829
PYGB	1.170424778	4.70E-50
PYGL	3.009435797	1.51E-293

PYGM	-1.193965548	1.02E-50
PYGO1	1.167460072	1.68E-72
PYGO2	1.119474681	7.16E-67
PYHIN1	0.19524762	6.61E-45
PYM1	1.776159065	5.88E-231
PYROXD1	0.257741302	2.39E-05
PYROXD2	-0.110543599	0.032234033
PYURF	-1.204577823	8.11E-90
PYY	0.134376612	2.23E-11
PZP	-0.090251494	0.058292007
QDPR	-0.625475003	5.84E-15
QKI	1.78849107	6.21E-121
QPCT	1.559670768	3.10E-47
QPCTL	1.314424755	1.75E-92
QPRT	1.391596448	5.24E-96
QRFP	0.554124508	3.59E-165
QRFPR	0.016240926	0.717808871
QRICH1	1.35831026	6.10E-57
QRICH2	-0.121179768	0.100606721
QRSL1	1.738951525	4.49E-239
QSER1	1.646659735	2.35E-147
QSOX1	1.847555665	4.70E-162
QSOX2	1.074863388	4.66E-24
QTRT1	0.143561069	0.045168063
QTRT2	1.056335274	3.33E-106
R3HCC1	1.521199903	6.09E-162
R3HCC1L	0.628345448	1.32E-25
R3HDM1	-0.072936379	0.508172394
R3HDM2	-0.710761749	2.33E-19
R3HDM4	1.171169439	1.50E-108
R3HDML	-0.018916735	0.268073237
RAB10	2.212370665	1.41E-282
RAB11A	1.417718142	2.00E-112
RAB11B	0.782231697	6.97E-45
RAB11FIP1	0.155335862	6.02E-05
RAB11FIP2	-0.295599743	1.51E-05
RAB11FIP3	0.338100772	6.92E-06
RAB11FIP5	0.476951118	4.17E-08
RAB12	0.878610535	2.39E-35
RAB13	0.717217727	2.89E-32
RAB14	1.601041436	8.75E-129
RAB15	-0.902882341	1.45E-11
RAB17	0.063266956	0.100759154
RAB18	0.743818461	9.61E-35
RAB19	0.172792778	9.16E-241
RAB1A	1.229410088	3.48E-75
RAB1B	0.773510729	6.85E-42
RAB21	0.859889892	1.07E-65

RAB22A	1.110457563	1.74E-103
RAB23	1.073220122	1.29E-64
RAB24	-2.70995802	9.64E-217
RAB25	-0.18832349	8.03E-07
RAB26	-2.213737977	8.12E-58
RAB27A	1.369788687	3.68E-156
RAB27B	0.391306665	8.52E-05
RAB28	1.178974531	3.33E-144
RAB29	2.195520245	1.54E-305
RAB2A	1.059109365	2.41E-56
RAB2B	1.195110576	4.78E-88
RAB30	0.645657411	6.57E-37
RAB31	2.534332355	4.25E-271
RAB33A	0.801224227	6.40E-18
RAB33B	1.222489682	1.86E-65
RAB34	2.055200229	1.57E-215
RAB35	1.465537516	7.12E-102
RAB36	1.235101137	4.14E-75
RAB37	-1.061596047	7.81E-10
RAB39A	1.707984304	3.43E-203
RAB39B	1.139016673	5.48E-52
RAB3A	-2.375030836	2.10E-64
RAB3B	-0.634139727	2.43E-11
RAB3C	-2.013032817	1.03E-61
RAB3D	0.49651654	2.23E-14
RAB3GAP1	0.891982133	2.53E-45
RAB3GAP2	1.318482571	7.22E-91
RAB3IL1	1.74136323	1.19E-182
RAB3IP	0.432939447	1.04E-06
RAB40A	0.168498161	0.000110501
RAB40AL	-0.098402323	9.36E-07
RAB40B	-1.747032467	1.81E-133
RAB40C	0.031407643	0.747216482
RAB43	-0.905385969	1.14E-65
RAB44	0.068972463	1.81E-52
RAB4A	0.821262667	5.21E-49
RAB5A	1.108852032	1.68E-97
RAB5B	0.576664613	2.49E-28
RAB5C	0.397386083	1.42E-16
RAB6A	0.132667233	0.061374469
RAB6B	-0.19753598	0.042684397
RAB6C	-0.506531833	4.56E-24
RAB7A	0.164993175	0.002822309
RAB7B	2.411943168	1.04E-211
RAB8A	0.697857641	5.92E-35
RAB8B	1.871497031	1.32E-228
RAB9B	-0.171039468	0.099087324
RABEP1	-0.381109091	2.30E-13

RABEP2	1.003985642	1.18E-106
RABEPK	1.895955894	2.37E-257
RABGAP1	0.458465484	1.16E-20
RABGAP1L	-0.034457693	0.635199684
RABGEF1	0.040335734	0.514724837
RABGGTA	0.559761487	1.97E-13
RABGGTB	0.837709714	2.55E-50
RABIF	1.274988253	3.23E-87
RABL2A	-0.448014274	3.12E-09
RABL2B	0.330015328	1.95E-05
RABL3	0.738689512	1.49E-48
RABL6	-0.880142035	1.84E-32
RAC1	1.658804001	1.28E-220
RAC2	2.963246319	3.40E-286
RAC3	1.105496624	6.23E-22
RACK1	2.383109204	4.120507486316e-321
RAD1	1.423615108	3.64E-118
RAD17	1.140597902	1.34E-76
RAD18	2.039790122	1.89E-245
RAD21	1.165038182	1.65E-82
RAD21L1	0.000829722	0.898253422
RAD23A	0.953457416	4.77E-42
RAD23B	1.769789668	8.34E-127
RAD50	-2.327660823	4.13E-252
RAD51AP2	-0.172843936	4.09E-13
RAD51C	1.322209086	5.20E-110
RAD51D	0.919573337	7.39E-108
RAD52	0.616409288	3.58E-16
RAD54B	0.96069347	1.94E-307
RAD54L	1.202781432	1.39E-88
RAD54L2	0.614580954	3.00E-19
RAD9A	0.687959003	1.82E-22
RAD9B	0.75456205	1.41E-180
RADIL	0.020389767	0.816870587
RAE1	1.294146689	1.14E-59
RAET1E	0.030021838	0.006592382
RAET1G	0.293161191	3.93E-31
RAET1L	0.158377847	2.50E-32
RAF1	-0.629366744	9.11E-16
RAG1	0.390079537	5.01E-294
RAG2	-0.008890124	0.011934316
RAI1	0.239330661	0.005068935
RAI14	1.679334252	7.67E-114
RAI2	-0.294326671	2.01E-06
RALB	1.173142821	1.50E-66
RALBP1	1.237473517	1.50E-103
RALGAPA1	-0.122736816	0.069713628
RALGAPA2	0.916007665	2.69E-38

RALGAPB	0.69096493	1.44E-19
RALGDS	-2.5314082	4.95E-197
RALGPS1	-0.482284746	5.62E-08
RALGPS2	0.457146757	3.58E-11
RALY	2.088533027	9.30E-300
RALYL	-2.134592494	3.15E-78
RAMP1	2.212575713	5.49E-255
RAMP2	1.522882967	1.45E-131
RAMP3	1.733642581	2.36E-53
RAN	1.777467292	6.50E-141
RANBP1	1.224043796	3.91E-73
RANBP10	0.697808337	4.38E-13
RANBP17	-0.468311102	3.46E-23
RANBP2	0.684817936	1.34E-20
RANBP3	0.601708204	3.26E-27
RANBP3L	0.311173178	0.007081705
RANBP6	1.16864243	1.76E-58
RANBP9	0.983269128	7.44E-52
RANGAP1	0.021620892	0.773257972
RANGRF	-2.028085235	1.05E-278
RAP1A	1.585947984	6.66E-161
RAP1GAP	-1.616702052	2.37E-39
RAP1GAP2	-1.160896485	4.90E-16
RAP1GDS1	0.528596353	9.38E-14
RAP2A	1.204070699	1.29E-76
RAP2B	2.349402108	1.49E-284
RAP2C	1.80094603	2.37E-264
RAPGEF1	0.940159983	4.74E-45
RAPGEF2	-0.296037123	1.76E-05
RAPGEF3	-1.434535681	3.11E-89
RAPGEF4	-2.3476184	4.17E-67
RAPGEF5	-1.029535825	1.82E-26
RAPGEF6	-0.577439012	1.10E-09
RAPGEFL1	-1.597393033	1.65E-56
RAPH1	-0.148113371	0.000552213
RAPSN	0.252992585	4.20E-32
RARA	1.971656315	5.41E-131
RARB	0.958328671	1.64E-23
RARG	0.596918827	2.97E-16
RARRES2	1.649737088	4.09E-119
RARS2	1.924353784	1.02E-188
RASA1	0.528965449	9.74E-13
RASA2	0.197649507	0.014732584
RASA3	1.599248338	3.61E-140
RASA4	0.015392115	0.667892368
RASA4B	0.671970683	2.26E-68
RASAL1	-1.595609792	1.68E-48
RASAL2	0.421420811	6.15E-13

RASAL3	1.620053783	5.20E-144
RASD1	1.578040734	2.57E-52
RASD2	-1.534636985	1.59E-24
RASEF	1.190207917	3.40E-302
RASGEF1A	-1.402989874	1.30E-60
RASGEF1B	0.576972453	2.29E-18
RASGEF1C	-0.033883154	0.811525928
RASGRF1	-2.452632381	6.17E-62
RASGRF2	-1.112196081	8.83E-26
RASGRP1	-0.0595311	0.682719453
RASGRP2	-0.458578569	2.39E-08
RASGRP3	0.341287319	0.001117983
RASGRP4	1.598047736	1.79E-278
RASIP1	0.417013313	2.76E-09
RASL10A	-0.584612029	1.49E-11
RASL10B	0.252459468	0.008322744
RASL11A	-0.061729034	0.488862432
RASL11B	-1.979584567	4.34E-49
RASL12	2.101282974	1.08E-111
RASSF1	1.063255642	2.36E-60
RASSF10	0.876847821	1.45E-96
RASSF2	1.522978721	2.20E-57
RASSF3	1.354206756	1.48E-111
RASSF4	0.509212945	9.32E-17
RASSF5	1.154522684	3.73E-68
RASSF6	-0.004994908	0.480275652
RASSF7	-0.53929505	4.94E-06
RASSF8	1.365781827	2.93E-136
RASSF9	0.43336355	3.63E-16
RAVER1	0.469565347	1.88E-15
RAVER2	0.798274419	2.91E-32
RAX	-0.005334779	0.780521755
RAX2	-0.071460488	2.37E-12
RB1	2.295342812	1.44E-265
RB1CC1	0.205472362	0.007128556
RBAK	1.122313242	4.50E-60
RBBP4	1.403966669	3.11E-109
RBBP5	1.478565225	2.64E-104
RBBP6	0.583623254	1.99E-13
RBBP7	1.064448237	3.59E-73
RBBP8NL	-0.1194717	1.25E-05
RBCK1	0.85252118	1.36E-26
RBFA	-0.896117587	1.28E-35
RBFOX1	-3.295967512	2.41E-121
RBFOX2	-0.456042796	0.000143287
RBFOX3	-3.584441017	1.69E-92
RBKS	0.62603646	2.62E-41
RBL1	2.130534444	1.66E-280

RBL2	0.39647616	2.17E-07
RBM10	1.214085743	4.59E-55
RBM11	-1.594105079	2.58E-80
RBM12	1.917880662	1.80E-166
RBM12B	0.453716136	9.46E-15
RBM14	0.5547743	2.26E-12
RBM15	1.569373065	7.70E-201
RBM15B	1.527501684	1.66E-140
RBM17	0.163293035	0.000443408
RBM18	1.53880341	1.47E-137
RBM19	0.61985327	1.39E-23
RBM20	0.076431331	0.064830867
RBM22	1.626514578	1.03E-141
RBM23	1.341046169	2.41E-96
RBM24	-0.212629029	0.007306175
RBM25	-0.177812117	0.04622903
RBM26	0.341796779	4.47E-07
RBM27	0.885086672	1.84E-49
RBM28	1.211621779	2.99E-98
RBM3	1.885198616	4.65E-115
RBM33	0.337383022	0.000146944
RBM34	-0.919629003	1.38E-120
RBM38	1.641470223	1.33E-114
RBM39	-0.137030511	0.048809102
RBM4	-1.64519451	9.07E-105
RBM41	0.667637349	1.51E-33
RBM42	1.620234232	2.62E-186
RBM44	0.239728783	7.09E-79
RBM45	1.04610898	1.42E-89
RBM46	0.209316095	2.35E-91
RBM48	0.413331685	2.67E-09
RBM4B	0.854296193	1.87E-31
RBM5	-0.643578861	1.53E-15
RBM6	-0.183712972	0.020304325
RBM7	1.722343592	4.98E-271
RBM8A	1.174463768	4.64E-98
RBMS1	1.104468511	6.62E-91
RBMS2	1.011085852	1.00E-99
RBMS3	0.332572809	1.88E-22
RBMX	2.114065375	1.18E-193
RBMX2	1.449001418	2.60E-208
RBMXL1	1.243445598	1.31E-177
RBMXL2	-0.020145038	0.050822959
RBMXL3	-0.00600422	0.016096613
RBMY1A1	0	1
RBMY1B	-6.51E-06	0.991807666
RBMY1D	0	1
RBMY1E	9.69E-05	0.872530941

RBMY1F	-0.000114344	0.85565474
RBMY1J	-0.000263045	0.643942854
RBP2	0.373167877	2.38E-192
RBP3	-0.17922181	3.64E-13
RBP4	-1.735282696	5.00E-25
RBP5	-0.214349014	0.000226055
RBP7	0.210741716	0.047876761
RBPJ	1.121638353	8.41E-94
RBPJL	0.012036921	0.398602188
RBPMS	0.870744753	2.58E-76
RBPMS2	1.487518491	4.21E-216
RBSN	0.891624299	1.96E-54
RBX1	0.209645174	7.95E-05
RC3H1	0.618858477	6.13E-17
RC3H2	1.024189557	4.04E-55
RCAN1	2.090458628	1.11E-212
RCAN2	-1.422288327	6.71E-39
RCAN3	-0.105183289	0.454428539
RCBTB1	0.881098006	3.59E-62
RCC1L	1.78366202	2.20E-295
RCC2	2.485647222	5.88E-265
RCCD1	0.185988391	0.030097708
RCE1	0.263035926	0.006359419
RCHY1	0.68785144	1.73E-31
RCL1	1.750500009	1.87E-289
RCN1	1.639848897	2.53E-110
RCN2	1.335562372	2.42E-89
RCOR1	1.267452263	1.04E-68
RCOR2	1.754287347	6.23E-79
RCOR3	0.467196014	6.17E-10
RCSD1	2.159713236	1.35E-275
RCVRN	-0.059975392	0.203665435
RD3	-0.07787235	0.001102887
RD3L	-0.033733577	4.57E-07
RDH11	0.526800282	2.23E-18
RDH12	-0.076550893	0.184112692
RDH13	-0.125898941	0.040461487
RDH14	0.695442826	8.25E-34
RDH16	0.181642664	2.82E-06
RDH5	-0.47357446	3.11E-15
RDH8	0.042916376	9.92E-15
RDM1	1.098012236	6.95E-306
RDX	2.199831116	1.06E-243
REC114	0.022677373	0.007512779
REC8	0.357870362	0.000686566
RECK	0.920835165	2.77E-59
RECQL	1.674538982	4.33E-154
RECQL4	1.143315394	1.18E-32

RECQL5	0.554485836	2.58E-24
REEP1	0.341861619	0.000464443
REEP2	0.105656172	0.240773265
REEP3	1.553265133	2.65E-66
REEP4	2.482181078	2.38E-288
REEP5	0.912074846	1.97E-34
REEP6	0.036364845	0.504737595
REG1A	-0.871430939	6.28E-15
REG1B	-0.519686407	8.07E-12
REG3A	-0.258677239	2.82E-08
REG3G	-0.103730939	5.18E-05
REG4	0.027472156	1.09E-09
REL	-0.00438612	0.940840021
RELA	1.740471067	1.32E-159
RELB	2.512047351	3.33995223701683e-312
RELL1	2.874539292	6.27E-255
RELL2	-1.417166462	1.74E-34
RELN	-2.015746445	1.17E-31
RELT	0.704989174	8.18E-34
REM1	0.457845653	2.99E-26
REM2	-0.357454974	1.15E-05
REN	-0.018187584	0.165726519
RENBP	1.742093907	1.51E-122
REP15	-0.397579015	5.24E-27
REPIN1	1.417747543	1.82E-121
REPS1	0.283016955	0.00090702
REPS2	-1.987761617	1.51E-101
RER1	1.439197427	9.61E-114
RERE	0.105463841	0.191372129
RERG	-0.871473348	9.76E-19
RERGL	0.046456515	0.57722866
RESP18	-2.091072553	2.13E-46
REST	1.972533432	6.03254153572162e-321
RET	0.284783558	0.00108147
RETN	0.983518694	1.01E-110
RETNLB	0.008913856	0.000284265
RETSAT	1.489700352	7.82E-142
REV1	0.200085006	0.045361775
REV3L	0.647123049	5.07E-12
REXO1	-0.247860253	0.000939812
REXO2	0.677958286	1.71E-40
REXO4	1.350549724	4.97E-109
RFC1	1.467923052	1.48E-148
RFC3	2.555080173	1.85E-234
RFC4	2.045891038	2.99E-144
RFC5	1.954702202	2.13E-209
RFESD	0.32806315	1.76E-26
RFFL	0.207325313	0.00580555

RFK	1.149507489	3.66E-43
RFLNA	-0.376757004	8.64E-09
RFLNB	1.913398694	2.15E-249
RFNG	0.029949466	0.637782252
RFPL1	-0.210571625	2.40E-22
RFPL2	-1.495222455	1.14E-68
RFPL3	-0.043599583	0.005726619
RFPL4A	0.075909858	2.81E-09
RFPL4AL1	0.018420137	0.054030082
RFPL4B	0.044363596	0.0002709
RFT1	1.647001169	2.09E-213
RFTN1	2.41483012	4.05E-161
RFTN2	1.589016306	1.28E-146
RFWD3	2.098629431	1.35E-202
RFX1	1.001995602	9.79E-33
RFX2	1.559446029	3.58E-181
RFX3	1.073519916	2.98E-65
RFX4	1.747961452	1.40E-104
RFX5	1.400443702	7.45E-102
RFX6	-0.042734508	2.69E-08
RFX7	1.187950699	3.71E-61
RFX8	0.122401605	0.002175401
RFXANK	1.536507923	3.09E-264
RFXAP	1.379601789	3.99E-97
RGCC	1.232545647	1.65E-88
RGL1	1.035664314	5.93E-64
RGL2	1.248065045	1.31E-66
RGL3	0.185849905	0.003987273
RGL4	-0.80209619	8.38E-41
RGMA	2.739408232	3.69E-287
RGMB	0.172722024	0.029017026
RGN	0.260946432	5.70E-05
RGP1	0.769472344	2.05E-38
RGPD1	-0.024405245	0.044339901
RGPD2	-0.025545455	0.157726831
RGPD3	-0.025492267	0.045408383
RGPD5	0.048605913	2.94E-155
RGPD6	0.036535112	1.94E-128
RGPD8	-0.137056384	0.000156807
RGR	-0.126663986	0.213419861
RGS1	3.040097242	1.60E-56
RGS10	3.354898023	1.29E-200
RGS11	-3.540359513	2.62E-172
RGS12	0.373665064	2.44E-07
RGS13	0.082270377	1.53E-28
RGS14	-1.313085169	1.38E-10
RGS16	2.601955365	6.52E-138
RGS17	0.683735663	5.26E-20

RGS18	1.819741262	1.50E-249
RGS2	1.787511119	7.38E-67
RGS20	-0.465627998	1.49E-06
RGS21	0.00294805	0.054388191
RGS22	0.879429972	5.98E-68
RGS3	0.939910625	1.08E-66
RGS4	-0.808968879	9.82E-06
RGS5	0.022673784	0.761262841
RGS6	1.018999914	1.25E-32
RGS7	-2.532837308	8.16E-100
RGS7BP	-0.86285328	5.04E-15
RGS8	-1.060431961	3.88E-18
RGS9	-0.033980893	0.787638938
RGS9BP	-0.025159908	0.272280626
RGSL1	-0.004031664	0.03577608
RHAG	0.1537121	1.39E-31
RHBDD1	1.575080516	4.63E-268
RHBDD2	1.487396378	5.86E-76
RHBDD3	0.930455892	5.43E-50
RHBDF1	1.30850209	6.06E-127
RHBDF2	2.18999266	2.99E-129
RHBDL1	-1.441808999	4.82E-38
RHBDL2	0.118198739	0.066887589
RHBDL3	0.131717896	0.088261598
RHBG	-0.452091294	0.009128709
RHCE	0.806604316	2.44E-67
RHCG	-0.240971913	3.03E-05
RHD	0.255265496	1.04E-20
RHEB	1.606647651	9.33E-170
RHEBL1	0.753099926	1.56E-16
RHO	-0.088268458	0.006117267
RHOA	1.498132313	5.02E-182
RHOB	1.67612702	1.63E-138
RHOBTB1	1.442253004	5.47E-104
RHOBTB2	-0.615143913	7.40E-10
RHOBTB3	1.547946501	2.50E-113
RHOC	2.105831368	7.51E-242
RHOD	0.990842084	1.52E-49
RHOF	-1.299557224	7.49E-67
RHOG	2.595140094	3.14E-193
RHOJ	2.054483677	4.65E-266
RHOQ	0.32220739	6.77E-09
RHOT1	0.951335609	3.45E-51
RHOT2	-0.262995781	0.006132937
RHOU	1.772599113	2.37E-74
RHOV	-0.766765079	5.23E-25
RHOXF1	-0.900803921	4.29E-16
RHOXF2	0.028173582	2.81E-09

RHOXF2B	0.031836641	1.00E-29
RHPN1	0.477985316	3.41E-08
RHPN2	1.886316917	3.91E-131
RIBC1	1.5896816	2.02E-175
RIBC2	0.843186635	8.29E-48
RIC1	0.576781732	4.72E-19
RIC3	-0.369767485	1.34E-05
RIC8A	1.399644578	5.52E-117
RIC8B	0.691888984	1.61E-19
RICTOR	-0.163150017	0.031490585
RIDA	0.272665752	1.91E-05
RIF1	0.765370474	4.19E-27
RIIAD1	-0.475804092	5.48E-12
RILP	0.481564552	1.72E-17
RILPL1	-0.093369377	0.064622442
RILPL2	1.468966401	1.74E-260
RIMBP2	-1.573625312	3.82E-45
RIMBP3	0.175746063	1.37E-91
RIMBP3B	0.050861829	1.33E-91
RIMBP3C	0.039545779	4.77E-43
RIMKLA	-0.558437328	2.77E-11
RIMKLB	0.485075874	5.98E-14
RIMS1	-2.713267349	1.08E-83
RIMS2	-2.406443094	5.82E-133
RIMS3	-1.696538451	2.39E-32
RIMS4	-0.595789234	1.04E-06
RIN1	-0.314813976	0.005462096
RIN2	1.620622332	1.32E-169
RING1	1.491212764	3.62E-104
RINL	0.787271479	2.02E-51
RINT1	1.596078762	4.13E-83
RIOK1	1.494198647	1.52E-120
RIOK2	1.52194407	2.69E-185
RIOK3	1.764241085	2.65E-125
RIOX1	0.716394691	8.31E-30
RIOX2	1.030649558	9.73E-95
RIPK1	0.803912365	5.88E-34
RIPK2	2.237782355	7.57E-268
RIPK3	0.853749975	6.46E-98
RIPK4	0.037777057	0.051760309
RIPPLY1	0.081865514	2.21E-12
RIPPLY2	-3.204140874	2.92E-256
RIPPLY3	0.337448029	5.25E-46
RIT2	-1.166489351	1.16E-16
RITA1	1.093664241	2.55E-55
RLBP1	0.930742293	2.23E-22
RLF	1.00475945	1.58E-36
RLIM	0.747109159	7.08E-32

RLN1	0.330163535	2.62E-105
RLN2	0.416821436	1.42E-113
RLN3	0.010421556	0.244670145
RMDN1	1.271100169	3.35E-157
RMDN2	0.642830111	1.83E-68
RMDN3	0.659030076	9.34E-38
RMI1	2.155225759	2.07E-213
RMI2	2.115197938	2.54E-230
RMND1	0.381470474	4.58E-09
RMND5A	0.270349075	0.005310882
RMND5B	0.377571977	2.89E-12
RNASE1	1.818845676	1.77E-62
RNASE10	0.418416235	4.41E-237
RNASE11	-0.008735367	5.80E-05
RNASE12	-0.000486321	0.799194239
RNASE13	0.014671839	0.149636519
RNASE2	4.027246275	4.48043430931134e-318
RNASE3	2.093843359	2.60E-276
RNASE4	-1.673975422	3.31E-168
RNASE7	0.075535149	2.67E-22
RNASE8	0.024413511	1.83E-06
RNASE9	0.001408737	0.138170226
RNASEH1	1.556933929	1.67E-125
RNASEH2B	-0.274896859	2.08E-07
RNASEH2C	0.503234483	1.98E-12
RNASEL	1.448729817	2.62E-220
RNASET2	-0.409185282	3.69E-07
RND1	-0.67100719	1.21E-11
RND2	1.955945785	7.25E-124
RNF10	0.769318433	1.77E-36
RNF103	-0.984639123	2.20E-40
RNF11	0.626009984	7.05E-23
RNF111	1.133454463	7.42E-59
RNF112	-0.741844919	1.28E-08
RNF113A	1.458875993	2.42E-99
RNF113B	0.023333786	0.004795556
RNF114	1.191473153	8.38E-105
RNF115	0.572291032	1.29E-11
RNF121	1.616542372	5.19E-154
RNF122	2.880613122	3.22E-114
RNF123	0.231837364	0.014578218
RNF125	0.352511485	5.58E-08
RNF126	0.288436966	9.27E-06
RNF128	-0.201185474	0.010336908
RNF13	1.894739738	1.91E-125
RNF130	0.630393344	1.07E-21
RNF133	-0.032599253	0.322473268
RNF138	1.953629192	5.46E-219

RNF139	2.105093811	8.87E-274
RNF14	1.357222051	1.96E-125
RNF141	0.382120364	1.14E-08
RNF144A	-0.277090433	0.004838108
RNF144B	1.220929904	1.70E-108
RNF145	1.942832429	6.86E-126
RNF146	0.300712682	0.000218947
RNF148	-0.010488315	0.767667457
RNF149	0.793613476	6.14E-37
RNF150	-0.026988809	0.76938381
RNF151	0.129841875	6.81E-15
RNF152	-0.228460565	0.041759858
RNF157	-0.792660359	4.33E-27
RNF165	-0.161339223	0.100948771
RNF166	1.065236613	5.82E-56
RNF167	1.289923461	1.54E-106
RNF168	1.036593053	1.29E-53
RNF169	0.745946343	1.62E-40
RNF17	-0.026140489	0.057519644
RNF170	0.519077625	9.50E-19
RNF175	-0.217142265	0.014561733
RNF181	1.962158364	1.60E-263
RNF182	1.140333113	6.00E-32
RNF183	0.062650075	1.15E-08
RNF185	1.259871543	8.93E-109
RNF186	-0.003574408	0.243029222
RNF187	1.021934715	7.26E-40
RNF19A	2.057987191	7.99E-209
RNF19B	1.923176411	1.77E-120
RNF2	2.423804241	1.49E-297
RNF20	1.231234615	3.81E-116
RNF207	-1.885739277	4.98E-66
RNF208	-0.491276767	3.68E-10
RNF212	-1.185643071	1.76E-52
RNF213	1.110698512	2.12E-83
RNF214	0.580505151	3.38E-23
RNF215	0.631530292	1.79E-24
RNF216	1.868766882	1.13E-201
RNF217	0.068590161	0.131511462
RNF220	-0.143494402	0.027828381
RNF222	0.01008322	0.196593848
RNF223	-0.04385167	7.19E-06
RNF224	0.211494822	5.95E-07
RNF225	-0.023397891	0.016591576
RNF24	1.398252065	1.52E-137
RNF25	1.592567807	5.29E-127
RNF26	1.673582257	7.41E-116
RNF32	-0.298685541	7.90E-23

RNF34	0.711013139	2.90E-31
RNF38	1.222899774	1.90E-63
RNF39	0.941931721	1.65E-79
RNF4	1.429670045	1.35E-99
RNF40	1.091990123	2.69E-46
RNF41	1.12671324	2.28E-44
RNF43	-1.194866935	8.33E-32
RNF44	0.539996612	1.07E-12
RNF5	1.694128875	2.62E-192
RNF6	0.524429002	4.84E-13
RNF7	1.269448971	1.78E-135
RNF8	0.818774874	1.67E-34
RNFT1	1.358645648	2.78E-81
RNFT2	0.498102629	4.24E-13
RNGTT	1.057097733	4.80E-57
RNH1	1.249920313	2.01E-90
RNLS	0.369839442	4.12E-16
RNMT	0.897468151	8.59E-35
RNPC3	-1.418007384	3.31E-42
RNPEP	1.689967674	2.72E-148
RNPEPL1	1.350058014	2.40E-154
RNPS1	0.210290715	0.006290698
ROBO1	1.788615921	3.24E-195
ROBO2	1.280322792	2.15E-53
ROBO3	1.019376687	5.46E-71
ROBO4	1.527521716	1.42E-132
ROCK1	1.063691271	7.52E-71
ROCK2	0.489692709	5.43E-10
ROGDI	-2.823767598	5.11E-242
ROM1	1.370196088	2.93E-112
ROPN1	0.064361739	4.45E-17
ROPN1B	-0.050503909	0.1375356
ROPN1L	0.511445585	1.01E-09
ROR1	1.29253207	7.17E-177
ROR2	0.766871165	1.07E-86
RORA	0.078806154	0.310552939
RORB	0.565507913	3.66E-09
RORC	-0.509788905	4.11E-08
ROS1	-0.029897242	0.416740062
RP1	0.056722141	2.52E-74
RP1L1	0.105503308	2.09E-104
RP9	1.349890284	2.75E-125
RPA1	2.242947432	3.97E-255
RPA2	2.289033585	2.98E-260
RPA3	2.011476734	4.38E-220
RPA4	0.106553175	7.68E-25
RPAIN	0.539978286	1.05E-15
RPAP1	1.221244086	1.41E-106

RPAP2	0.486576862	9.89E-10
RPAP3	1.24983285	1.64E-76
RPE	1.400462983	8.86E-119
RPEL1	0.289158448	9.74E-135
RPF1	1.768218803	4.46E-182
RPF2	1.597222866	2.07E-236
RPGR	0.395563384	7.31E-13
RPGRIP1	0.439016254	1.27E-162
RPGRIP1L	0.921197619	2.13E-76
RPH3A	-1.648178095	2.41E-30
RPH3AL	0.299357457	6.47E-08
RPIA	2.026209627	2.97E-259
RPL10	0.361770578	9.48E-14
RPL10A	2.111605534	6.40E-277
RPL11	1.391029397	1.10E-139
RPL12	2.199295084	1.50E-302
RPL13	1.857426062	7.02E-278
RPL13A	0.694449558	1.94E-42
RPL14	1.434813193	1.58E-142
RPL15	1.026435309	2.87E-69
RPL18	0.884847148	6.71E-85
RPL18A	1.850909376	7.47E-227
RPL19	0.424668314	1.01E-19
RPL21	0.416721258	1.37E-15
RPL22	-0.42590025	2.36E-13
RPL23	1.288056109	3.80E-126
RPL23A	0.801882207	3.92E-70
RPL24	0.409496531	7.11E-16
RPL26	-2.215426226	1.02E-295
RPL27	1.731366881	1.71E-268
RPL27A	1.987606803	3.01E-293
RPL3	0.600805826	8.21E-29
RPL30	0.653410159	1.48E-35
RPL31	1.365357454	6.90E-159
RPL32	1.737271632	9.54E-257
RPL34	-0.337732732	4.91E-12
RPL35	1.065412042	2.80E-125
RPL35A	1.439871298	3.50E-178
RPL36	1.34884731	3.01E-178
RPL36AL	1.382535073	1.21E-130
RPL37	1.067835817	1.41E-98
RPL37A	1.273873071	3.12E-145
RPL38	-0.348201035	4.57E-14
RPL39L	1.659797381	2.96E-143
RPL3L	0.200792553	9.52E-36
RPL4	0.561651023	1.72E-24
RPL5	0.691155778	1.04E-34
RPL6	0.490852724	4.47E-19

RPL7	1.748540309	4.06E-175
RPL7A	2.079769236	5.52E-289
RPL7L1	1.238531712	5.05E-80
RPL8	1.754552215	4.89E-246
RPL9	-0.572806714	1.37E-22
RPLP1	0.800459649	3.94E-70
RPLP2	1.529718417	3.10E-213
RPP14	-0.24934441	0.00011608
RPP25	0.035491273	0.66832526
RPP25L	1.416140573	3.07E-164
RPP30	1.041947842	1.01E-91
RPP38	0.37299156	5.78E-08
RPP40	1.759215814	1.16E-225
RPRD1A	0.946822076	4.93E-55
RPRD1B	1.687257547	8.91E-135
RPRD2	1.0206962	3.84E-43
RPRM	1.038837682	4.04E-19
RPRML	-1.864455701	2.28E-38
RPS11	1.591125298	2.68E-219
RPS12	1.685124964	1.50E-210
RPS13	0.576200084	6.22E-28
RPS14	1.191872699	2.65E-147
RPS15	1.147221255	1.73E-120
RPS15A	0.315736179	1.15E-10
RPS16	0.69214217	5.91E-48
RPS18	1.308837997	1.97E-151
RPS19BP1	-0.886859726	2.28E-71
RPS20	0.652918042	5.91E-46
RPS21	2.12377955	5.60E-262
RPS23	0.341925241	1.90E-10
RPS24	0.286893196	1.06E-08
RPS25	0.377246432	5.53E-16
RPS26	1.440566974	1.06E-86
RPS27	1.687951432	5.32E-211
RPS27A	0.311457164	1.28E-10
RPS28	0.572572633	3.07E-40
RPS29	1.891310744	3.06E-279
RPS3	2.202188164	1.53637404773931e-310
RPS3A	0.964347138	1.14E-57
RPS4X	2.184836837	2.62206320002877e-317
RPS4Y1	1.634047839	6.56E-13
RPS4Y2	0.257417231	2.51E-172
RPS5	1.965486434	1.18E-301
RPS6	1.660434193	7.28E-201
RPS6KA1	1.63041383	5.14E-54
RPS6KA2	0.017099317	0.798596357
RPS6KA3	0.944354275	1.26E-39
RPS6KA4	0.166346792	0.083691223

RPS6KA5	-0.670171369	5.73E-23
RPS6KA6	-0.892030401	6.44E-35
RPS6KB1	0.886231913	2.99E-45
RPS6KB2	-0.374460849	1.34E-06
RPS6KC1	0.910959921	1.93E-64
RPS6KL1	-0.895297557	1.13E-22
RPS7	-0.063513628	0.228034833
RPS8	1.926099383	2.04E-251
RPS9	2.033198902	3.43622656682587e-320
RPSA	1.15955553	1.56E-142
RPTN	-0.004806979	0.151903403
RPTOR	0.85963202	5.36E-47
RPUSD1	1.317457204	1.97E-93
RPUSD2	1.83753201	5.63E-259
RPUSD3	0.273376624	2.37E-05
RPUSD4	0.980101961	1.09E-46
RRAD	0.726343344	7.78E-14
RRAGA	1.496589172	6.15E-98
RRAGB	1.220294333	1.56E-68
RRAGC	0.596673121	2.34E-28
RRAGD	1.302554841	8.34E-67
RRAS	2.182839775	2.13E-304
RRAS2	1.787403776	1.27240296167048e-312
RRBP1	1.702891745	3.56E-149
RREB1	1.043321812	1.92E-164
RRH	-0.338971533	5.65E-18
RRM1	2.428953626	1.34E-221
RRM2B	1.814503991	9.08E-211
RRN3	1.376000937	6.40E-82
RRNAD1	1.402637909	1.79E-120
RRP1	1.022389958	4.46E-68
RRP12	0.652697698	2.92E-23
RRP15	1.681482567	6.50E-223
RRP1B	1.471292037	6.90E-120
RRP36	1.999248831	2.11E-265
RRP7A	0.145932095	0.017899172
RRP8	1.358087412	1.57E-181
RRP9	2.432648611	3.03E-293
RRS1	1.202206043	7.30E-106
RS1	-0.629000966	3.28E-68
RSAD1	0.394528076	6.94E-07
RSAD2	2.048101081	3.62E-150
RSBN1	1.242206594	8.02E-62
RSBN1L	1.495639477	2.68E-109
RSF1	0.7010815	1.33E-36
RSL1D1	1.97408845	3.90E-190
RSL24D1	0.828136725	1.32E-31
RSPH1	-0.905645181	3.42E-22

RSPH10B	-0.056600956	0.002235878
RSPH10B2	0.043234616	0.000495481
RSPH14	0.450775779	1.65E-23
RSPH3	1.231481803	1.07E-124
RSPH4A	1.130915648	2.06E-53
RSPH6A	0.015144919	0.008998832
RSPH9	0.52640471	2.67E-20
RSPO1	-0.076921947	0.000906831
RSPO2	-0.16453015	0.151809666
RSPO3	-0.686183106	3.16E-16
RSPO4	-0.838835839	2.03E-22
RSPRY1	1.780419208	6.58E-200
RSRC1	1.504859284	8.44E-107
RSRC2	-0.050881515	0.466584564
RSRP1	-0.21088151	0.006879331
RTBDN	-0.658051398	7.57E-18
RTCA	1.426378009	2.23E-96
RTCB	1.671192475	5.69E-140
RTEL1	-3.251283268	3.88E-185
RTF1	0.817728182	2.12E-29
RTKN	1.320664047	9.21E-52
RTL1	-0.169395984	0.001773513
RTN1	-1.641811522	1.84E-32
RTN2	-1.017801105	1.31E-25
RTN3	0.489691357	2.22E-08
RTN4	0.598616317	7.33E-15
RTN4IP1	0.681725928	1.87E-29
RTN4R	-1.053305944	2.63E-12
RTN4RL1	-1.312981924	1.78E-34
RTN4RL2	0.576458838	2.59E-05
RTP1	-0.170355417	0.027244164
RTP2	-0.002603707	0.589709413
RTP3	0.04704912	1.99E-24
RTP5	-1.764817015	2.03E-38
RTTN	0.24845253	0.000135202
RUBCN	-1.295897458	7.91E-75
RUBCNL	0.833881304	1.44E-65
RUFY1	1.132242442	1.93E-109
RUFY2	-0.404534739	9.01E-06
RUFY3	0.413115581	1.99E-09
RUFY4	0.342431075	2.52E-25
RUNDC1	0.764614153	5.06E-25
RUNDC3A	-3.329587479	9.12E-227
RUNDC3B	-0.98291535	5.74E-38
RUNX1T1	-1.210746301	3.69E-28
RUNX2	1.124763061	1.48E-146
RUSC1	0.005802717	0.962986313
RUSC2	-0.661560169	5.64E-14

RUVBL1	1.880907262	5.14E-238
RUVBL2	1.995523356	1.48E-224
RWDD1	1.278759702	1.25E-108
RWDD2A	1.016939781	3.95E-50
RWDD2B	1.747865917	3.34E-183
RWDD3	-1.411944141	1.26E-159
RWDD4	1.853242986	1.24E-294
RXFP1	-0.532094697	8.79E-08
RXFP2	-0.084272197	9.10E-05
RXFP3	-0.015396136	0.211775512
RXFP4	0.165011572	1.09E-15
RXRA	0.902786915	1.83E-66
RXRB	0.724808968	9.10E-22
RXRG	-0.979336642	9.92E-20
RYBP	0.495796768	4.18E-19
RYK	1.201306782	5.63E-178
RYR1	-0.971016373	2.77E-32
RYR2	-2.280425043	1.28E-79
RYR3	-0.706852109	2.63E-20
S100A1	-1.835458078	2.07E-90
S100A12	0.353227795	0.00045262
S100A13	-1.253650012	3.60E-92
S100A14	-0.468344332	1.89E-31
S100A5	0.448140769	2.59E-79
S100A7	-0.157425587	0.001642482
S100A7A	-0.001526169	0.208122099
S100A8	2.393799291	1.14E-77
S100A9	2.958704641	1.74E-111
S100B	1.040715923	1.39E-30
S100G	0.02088143	2.48E-10
S100P	1.318637702	2.17E-192
S100PBP	0.968702921	6.29E-41
S100Z	0.697160399	3.89E-193
S1PR1	-0.042384031	0.54036946
S1PR4	1.006388347	2.43E-236
S1PR5	-0.889803659	2.34E-15
SAA2	1.340102385	6.98E-177
SAA4	-0.083645913	0.000302487
SAAL1	1.743338098	1.43E-192
SAC3D1	1.423280846	1.45E-105
SACM1L	0.643495186	1.04E-22
SACS	0.903182559	9.40E-45
SAE1	1.922920587	6.14E-164
SAFB	-0.314805413	2.03E-05
SAFB2	0.126619715	0.103239951
SAG	-0.416952663	4.38E-11
SAGE1	0.115129025	4.45E-06
SALL1	2.876206771	1.15E-216

SALL2	1.030569464	6.44E-31
SALL3	2.349232219	6.78E-199
SALL4	0.641184485	1.08E-70
SAMD1	1.302913925	2.72E-197
SAMD10	0.270122634	0.014852599
SAMD11	0.187047866	0.002676273
SAMD12	-1.431659455	3.18E-82
SAMD13	1.20173055	1.61E-304
SAMD14	-1.538737278	1.99E-44
SAMD15	1.001495671	2.33E-94
SAMD3	-0.111562433	4.49E-06
SAMD4A	0.297172414	0.000309039
SAMD4B	0.991590475	3.22E-63
SAMD5	0.536988446	2.09E-34
SAMD7	0.026213676	1.67E-13
SAMD8	0.50637682	4.97E-20
SAMHD1	1.289092193	3.17E-90
SAMM50	0.152829564	0.006096016
SAMSN1	2.507742719	3.00E-229
SAP130	1.56490301	2.55E-121
SAP18	0.769988766	1.04E-49
SAP25	-3.575584577	3.58E-269
SAP30BP	0.862761618	5.37E-53
SAP30L	0.900412446	1.66E-65
SAPCD1	-0.76241584	8.52E-44
SAPCD2	1.12003001	1.02E-67
SAR1A	1.136775021	4.26E-103
SAR1B	1.124486327	1.80E-109
SARAF	1.040404101	2.94E-60
SARDH	0.648096814	5.81E-45
SARM1	-0.065427824	0.405171588
SARS2	-2.076649307	2.02E-188
SART1	0.904699198	8.07E-50
SART3	0.61571408	1.20E-15
SASH1	0.271712908	5.65E-05
SASS6	0.950999486	6.33E-48
SAT1	2.182401536	1.07E-232
SAT2	0.827895632	2.09E-47
SATB1	-0.121835088	0.124935887
SATB2	1.767857169	3.43E-69
SATL1	0.310332142	9.13E-37
SAV1	0.256251203	2.79E-07
SAXO1	0.079121611	1.44E-41
SAXO2	0.906282646	3.73E-80
SAYSD1	1.765994618	1.33E-297
SBDS	1.258003356	1.83E-102
SBF1	-0.430212416	3.12E-07
SBF2	-0.047391657	0.37757202

SBK1	0.507680281	2.94E-05
SBK2	0.107539289	2.66E-05
SBK3	-0.01271682	0.570897979
SBNO1	0.393333962	6.31E-08
SBNO2	1.696244099	1.08E-114
SBSN	-0.500148936	3.22E-10
SBSPON	0.707246275	3.53E-21
SC5D	-0.043893472	0.587178539
SCAF1	0.911980091	2.11E-39
SCAF11	1.259765051	4.43E-91
SCAF4	0.962805489	8.20E-46
SCAF8	0.901255795	3.81E-45
SCAI	-0.068824152	0.327234329
SCAMP1	0.80721135	2.43E-27
SCAMP2	1.861452741	1.62E-269
SCAMP3	2.11231535	9.64E-238
SCAMP4	1.021237075	2.19E-63
SCAMP5	-0.530849274	5.17E-07
SCAND1	1.178602704	7.21E-98
SCAP	1.211425207	1.00E-67
SCAPER	-0.505021968	3.98E-19
SCARA3	2.278842766	1.82E-161
SCARA5	0.118795131	0.004589861
SCARB1	0.364553109	1.35E-11
SCARB2	0.600160726	1.24E-19
SCARF1	0.675117227	3.13E-26
SCARF2	1.788653276	2.40E-226
SCART1	-0.534403941	3.84E-20
SCCPDH	1.966691787	6.40E-142
SCD	-0.09486646	0.316721232
SCD5	1.511731441	3.33E-139
SCEL	-0.046695815	0.007192114
SCFD1	-1.323177532	2.35E-108
SCFD2	2.304400795	7.75E-303
SCG2	1.329272687	4.25E-18
SCG3	0.564043818	4.04E-10
SCG5	-0.055574922	0.619425094
SCGB1A1	-0.177081167	1.69E-10
SCGB1C1	-0.000921421	0.92370526
SCGB1C2	-0.026616859	0.042365097
SCGB1D1	-0.00809601	0.195031504
SCGB1D2	0.861464013	1.08E-29
SCGB1D4	0.010673387	0.265022256
SCGB2A1	-0.018730682	0.593526804
SCGB2A2	-0.077918127	2.42E-08
SCGB2B2	0.362055429	4.68E-56
SCGB3A1	-0.465842429	4.64E-11
SCGB3A2	0.28542022	1.54E-68

SCGN	-0.899042213	1.11E-06
SCHIP1	-0.362369789	6.20E-11
SCIN	2.429859209	3.50E-163
SCLT1	0.271981984	5.03E-08
SCLY	-1.680711669	9.78E-128
SCMH1	1.050088363	1.62E-73
SCML1	0.288236813	6.31E-07
SCML2	0.976186955	3.15E-127
SCML4	0.224520716	1.82E-53
SCN10A	0.01912921	1.82E-06
SCN11A	-0.259483432	8.11E-36
SCN1A	-0.747320499	2.44E-18
SCN1B	-1.859698343	1.22E-45
SCN2A	-1.674335337	4.57E-34
SCN2B	-2.27196201	8.80E-68
SCN3A	0.220449857	0.006872381
SCN3B	-1.617803133	2.13E-33
SCN4A	0.100181475	3.84E-09
SCN4B	-0.527160452	0.000382406
SCN5A	-0.018853177	0.547977724
SCN7A	0.80209481	1.49E-36
SCN8A	-1.24291771	1.37E-27
SCN9A	-0.236450059	0.000767163
SCNM1	2.030874079	3.16E-268
SCNN1A	0.092318877	2.17E-07
SCNN1B	1.441575268	3.56E-111
SCNN1D	-0.502166375	1.52E-08
SCNN1G	0.106552471	0.385194415
SCO1	1.160952551	9.89E-117
SCO2	-2.254632298	2.38E-171
SCOC	0.601528008	4.62E-16
SCP2	1.58524845	5.79E-212
SCP2D1	-0.005344092	0.103167537
SCPEP1	1.155605995	2.36E-61
SCRG1	2.07542503	2.96E-167
SCRIB	1.069106377	1.46E-71
SCRN1	0.739117536	2.71E-21
SCRN2	0.599922129	2.07E-19
SCRN3	1.439525857	1.95E-137
SCRT1	-3.038251053	3.23E-100
SCRT2	0.359157835	0.00012517
SCT	0.135353051	0.001561207
SCTR	0.078071383	5.95E-05
SCUBE1	-0.157858772	2.69E-05
SCUBE2	1.754240053	2.98E-153
SCUBE3	0.550041821	7.78E-89
SCX	0.215684822	0.05791513
SCYL1	0.611067201	2.20E-19

SCYL2	1.621343327	2.43E-128
SCYL3	0.916386045	2.58E-61
SDAD1	1.380396962	1.89E-90
SDC1	3.21486445	2.06519439961641e-321
SDC2	1.553180103	3.27E-94
SDC3	2.477304577	1.65E-278
SDC4	1.162467522	4.25E-30
SDCBP	1.759735239	3.91E-173
SDCBP2	-0.335483545	4.96E-08
SDCCAG8	1.528587586	1.34E-223
SDE2	2.106908374	1.50025742561252e-312
SDF2	1.885472326	1.21E-243
SDF2L1	1.844569871	1.45E-171
SDF4	1.060723723	2.17E-42
SDHAF1	1.180415981	3.73E-53
SDHAF2	0.58626405	9.74E-33
SDHAF4	1.551240162	2.40E-119
SDHB	1.064840886	1.35E-67
SDHD	1.391114355	5.17E-159
SDK1	1.372531183	3.40E-168
SDK2	0.412434812	5.11E-15
SDR16C5	-0.766865763	4.31E-29
SDR39U1	-1.537256206	3.99E-89
SDR42E1	-0.02076327	0.012102901
SDR42E2	-0.057809693	0.293202619
SDR9C7	-0.242624584	7.11E-10
SDS	-0.054386691	0.628075849
SDSL	2.632807627	6.44E-255
SEBOX	-0.05829523	4.39E-08
SEC11A	1.456338581	1.16E-166
SEC11C	1.44788193	2.10E-120
SEC13	1.156883243	2.30E-93
SEC14L1	0.688833784	4.64E-24
SEC14L2	1.249880269	6.65E-108
SEC14L3	0.170752618	4.95E-170
SEC14L4	-0.03510215	0.020600375
SEC14L5	-2.161721326	3.08E-94
SEC14L6	-0.045479136	0.330769101
SEC16A	1.236661542	4.02E-52
SEC16B	-1.148214297	2.22E-74
SEC22A	1.447512557	6.07E-161
SEC22B	1.674185526	5.84E-179
SEC22C	1.118161818	5.50E-79
SEC23A	1.069835498	7.60E-45
SEC23B	2.238387529	2.52E-256
SEC23IP	1.175807267	1.37E-65
SEC24A	2.038527309	3.46E-223
SEC24B	1.561088543	6.63E-121

SEC24C	0.393174766	2.36E-05
SEC24D	2.149421846	8.94E-206
SEC31A	1.479345807	1.76E-99
SEC31B	-1.674691155	4.11E-53
SEC61A2	-0.171358186	0.027992646
SEC62	0.57236493	5.13E-35
SEC63	0.94294138	2.60E-71
SECISBP2	0.490013123	1.25E-12
SECISBP2L	-0.053059982	0.456889482
SEH1L	0.652272385	9.16E-25
SEL1L	1.306517676	3.94E-90
SEL1L2	0.04803368	1.70E-06
SEL1L3	1.033042554	1.54E-15
SELE	-0.261111378	0.002755248
SELENBP1	-0.563770481	5.95E-12
SELENOH	-1.132289746	3.39E-69
SELENOI	0.683419065	1.27E-17
SELENOK	1.474310781	1.01E-204
SELENOM	-1.711150054	9.54E-231
SELENOO	-0.072888122	0.492972612
SELENOP	-0.553766593	9.33E-07
SELENOS	1.756800861	3.47E-205
SELENOT	2.004499944	2.22E-222
SELENOV	0.963202593	9.94E-283
SELENOW	-0.673069117	3.23E-47
SELL	2.062481512	1.33E-150
SELP	0.138528585	3.60E-10
SELPLG	2.980988777	3.60E-177
SEM1	1.812466194	1.22E-283
SEMA3A	1.310323716	3.68E-93
SEMA3B	-0.597986679	9.70E-10
SEMA3C	0.991701663	4.11E-31
SEMA3D	0.272077834	3.55E-09
SEMA3E	1.175003932	2.30E-92
SEMA3F	1.229990745	1.89E-68
SEMA3G	-1.319384765	4.46E-42
SEMA4A	-1.282601659	1.19E-55
SEMA4B	0.888440472	9.50E-45
SEMA4C	0.479518023	1.31E-07
SEMA4D	-0.756195488	2.96E-17
SEMA4F	0.535474481	7.13E-11
SEMA4G	-0.756311953	6.62E-13
SEMA5A	2.566881534	1.51E-266
SEMA5B	2.084716629	1.60E-116
SEMA6A	1.649489044	7.22E-107
SEMA6B	-1.258955308	4.70E-38
SEMA6C	0.098282176	0.429080051
SEMA6D	1.871883609	4.96E-158

SEMA7A	0.855558449	1.34E-10
SEMG1	0.016122123	0.00050696
SEMG2	0.001151167	0.698830574
SENP1	1.416874159	5.72E-134
SENP2	0.723173596	9.64E-35
SENP3	-1.106657082	6.20E-63
SENP5	0.446247423	3.45E-12
SENP6	0.402571683	4.09E-11
SENP7	0.678292099	4.37E-21
SENP8	0.939063411	3.42E-147
SEPHS1	1.508461926	8.01E-191
SEPHS2	2.050517779	2.41E-169
SEPSECS	1.307705561	1.15E-112
SERAC1	0.931269881	8.24E-66
SERF1A	0.363113824	9.38E-79
SERF1B	0.813335412	3.29E-122
SERF2	-0.19453358	2.53E-06
SERGEF	0.018658302	0.785121586
SERHL2	-0.134987462	0.01182743
SERINC1	0.381819709	6.52E-08
SERINC3	1.10538991	5.42E-69
SERINC5	1.47134844	8.60E-96
SERP1	1.693928623	3.86E-229
SERP2	-0.3521241	1.26E-07
SERPINA1	3.979945013	5.44E-297
SERPINA10	-0.167726163	6.47E-28
SERPINA11	0.070272047	5.73E-12
SERPINA12	0.004846981	0.726086924
SERPINA2	0.002483687	0.274057891
SERPINA3	-0.974484932	1.51E-07
SERPINA4	-0.000468792	0.916780842
SERPINA5	2.219647487	8.99E-256
SERPINA6	0.033010959	6.95E-15
SERPINA7	-0.002474428	0.479367771
SERPINA9	0.012114529	3.10E-12
SERPINB1	2.206393831	5.19E-202
SERPINB10	0.051179633	3.13E-33
SERPINB11	-0.015837889	0.01991017
SERPINB12	-0.000525125	0.924598567
SERPINB13	-0.042186014	1.20E-10
SERPINB2	0.212279548	2.23E-40
SERPINB3	-0.054942852	5.74E-05
SERPINB4	0.035696638	2.89E-07
SERPINB5	-0.067014325	3.93E-08
SERPINB6	0.701254172	6.41E-49
SERPINB7	0.034087803	5.01E-05
SERPINB9	0.97707214	1.03E-55
SERPINC1	0.151910473	7.47E-12

SERPIND1	-0.156880632	0.008511247
SERPINE2	0.307952489	2.22E-05
SERPINE3	0.180446792	5.26E-12
SERPINF1	2.769811265	4.86E-118
SERPINF2	1.407449276	4.24E-201
SERPING1	2.021732143	2.51E-165
SERPINH1	3.496015415	9.25E-211
SERPINI1	-1.330864121	8.77E-26
SERPINI2	0.102621345	0.039204794
SERTAD2	1.695287464	2.85E-168
SERTAD4	-0.366236731	1.72E-07
SERTM1	-0.666851636	7.01E-08
SESN1	0.135686344	0.070063523
SESN2	1.981182041	1.65E-156
SESN3	1.192392423	3.96E-78
SESTD1	0.51792658	6.07E-21
SET	1.76223374	2.55E-201
SETBP1	0.823848917	3.86E-27
SETD1A	1.190797097	3.48E-51
SETD1B	0.836106039	1.30E-21
SETD2	0.839130817	3.28E-29
SETD3	0.424524011	6.05E-24
SETD4	0.558590633	2.62E-23
SETD5	0.950217052	3.99E-26
SETD6	-0.075980225	0.419969033
SETD7	1.27068354	2.18E-121
SETD9	1.817157861	5.63E-232
SETDB1	0.872860419	1.07E-32
SETDB2	0.48111112	9.45E-21
SETMAR	1.5039331	4.18E-207
SETX	1.056211879	7.12E-64
SEZ6	0.503999735	0.000342269
SEZ6L	0.566249348	1.03E-06
SEZ6L2	-0.039604425	0.764427463
SF1	0.941437338	1.63E-31
SF3A1	1.495718793	1.32E-94
SF3A2	1.177723312	1.42E-47
SF3A3	2.570156148	6.05E-295
SF3B1	0.804107054	1.11E-26
SF3B2	0.403578848	1.09E-09
SF3B3	1.948402497	2.18E-149
SF3B6	1.79260914	4.30E-269
SFI1	-0.774058841	8.30E-11
SFMBT1	1.217795495	1.94E-105
SFMBT2	0.598704696	3.09E-21
SFN	0.246729854	0.01069874
SFPQ	1.224215986	1.68E-51
SFR1	1.192898187	1.41E-100

SFRP1	-0.81745739	6.48E-18
SFRP2	1.097575024	8.18E-24
SFRP5	-0.344599877	0.000114144
SFSWAP	-0.332979977	7.99E-07
SFT2D1	1.092609587	2.64E-83
SFT2D2	1.982436487	9.10E-169
SFTA2	0.368479583	1.30E-56
SFTA3	-0.516107246	7.28E-17
SFTPA1	-0.312382484	7.62E-13
SFTPA2	-0.780202644	9.14E-26
SFTPB	-0.31483426	2.54E-11
SFTPC	-1.150189122	6.87E-79
SFTPD	-1.392825078	2.69E-118
SFXN1	1.277647112	3.83E-80
SFXN2	0.898662849	4.58E-103
SFXN3	0.429874788	8.24E-05
SFXN4	1.04968301	1.47E-90
SFXN5	0.503916653	1.05E-13
SGCA	0.113127338	0.236608087
SGCB	2.203423445	2.60E-278
SGCD	-0.167126332	0.003876036
SGCE	-1.080152273	1.62E-69
SGCG	0.316888212	5.55E-11
SGCZ	-0.183500903	1.63E-11
SGF29	0.559496514	3.86E-14
SGIP1	-1.27440783	3.21E-75
SGK1	1.526116634	1.88E-82
SGK2	-0.378368861	8.49E-07
SGK3	-0.524633459	4.07E-12
SGMS1	0.397236818	1.72E-15
SGMS2	0.221958664	5.14E-05
SGPL1	1.575015248	2.88E-220
SGPP1	1.122206217	1.06E-67
SGPP2	-0.041127864	0.672855538
SGSH	1.355536491	3.21E-105
SGSM1	-2.16947309	2.96E-97
SGSM2	-1.212932891	5.87E-54
SGTA	0.424266028	2.05E-16
SGTB	0.268942205	0.002211673
SH2B1	0.380996726	3.67E-07
SH2B2	1.727198347	1.59E-105
SH2B3	1.929212882	4.23E-257
SH2D1A	0.36735757	1.89E-71
SH2D1B	0.015515606	0.675912966
SH2D2A	1.012795895	2.93E-180
SH2D3A	0.148009297	1.67E-33
SH2D3C	0.801649445	2.66E-25
SH2D4B	0.079626009	8.04E-26

SH2D5	-1.82785188	2.78E-37
SH2D6	-0.063980085	0.141836191
SH2D7	0.409721425	2.57E-90
SH3BGR	-2.57433415	8.47E-272
SH3BGRL	2.237388015	1.18E-169
SH3BGRL2	-0.697253701	9.19E-25
SH3BP1	-0.029757174	0.723473578
SH3BP2	1.895572916	1.90E-261
SH3BP4	2.284913706	1.34E-261
SH3BP5	-1.079356532	1.47E-26
SH3BP5L	1.330359309	6.12E-65
SH3D19	0.995757632	1.27E-73
SH3D21	0.370707114	6.88E-06
SH3GL1	2.209089167	2.28E-224
SH3GL2	-2.034909661	1.49E-36
SH3GL3	-3.004343401	3.47E-290
SH3GLB2	-1.455164837	4.32E-89
SH3KBP1	1.644906809	1.69E-79
SH3PXD2A	-1.183683681	5.91E-69
SH3PXD2B	2.615237231	2.03E-220
SH3RF1	1.298520042	3.33E-73
SH3RF2	-0.672357614	1.85E-15
SH3RF3	1.898411584	5.58E-133
SH3TC1	1.488265166	4.93E-90
SH3TC2	-0.835790443	9.21E-26
SH3YL1	0.500684983	1.44E-21
SHANK1	-1.718480895	1.11E-34
SHANK2	-1.372113111	4.86E-57
SHANK3	-2.20290165	4.15E-83
SHARPIN	1.14416504	3.33E-120
SHB	-0.287721719	0.0001397
SHBG	0.642681887	6.94E-116
SHC1	1.982960623	1.91E-166
SHC2	0.405447092	1.85E-09
SHC3	0.226113317	0.071627198
SHC4	0.935181489	1.43E-42
SHCBP1	2.08948973	7.33E-283
SHCBP1L	0.002806297	0.466686641
SHD	2.54043234	1.24E-167
SHE	-0.350974427	3.13E-17
SHF	-0.33604219	0.020058011
SHH	0.197232821	7.53E-05
SHISA2	-0.241308486	0.000313482
SHISA3	0.532264958	9.42E-15
SHISA4	0.853099612	5.25E-76
SHISA6	-0.401985149	3.80E-05
SHISA7	-1.766790037	3.20E-99
SHISA8	-1.403087204	3.45E-13

SHISA9	0.154276831	0.106479948
SHKBP1	2.227486102	4.01E-283
SHMT1	0.909640483	4.95E-28
SHMT2	2.473638603	6.60E-206
SHOC2	0.707508546	1.88E-27
SHOX	0.019933691	0.000435425
SHPK	-0.238829992	7.23E-05
SHPRH	0.138321804	0.015587579
SHQ1	1.664643673	7.26E-259
SHROOM1	0.228720117	0.001553689
SHROOM2	1.509818322	1.93E-110
SHROOM3	0.88958728	9.55E-23
SHROOM4	0.692060586	7.59E-20
SHTN1	-1.599079741	5.00E-81
SI	-0.004292696	0.23433038
SIAE	-0.146469016	0.028398074
SIAH1	0.655234344	6.84E-45
SIAH2	1.555525888	4.00E-113
SIAH3	-0.802077135	8.67E-36
SIDT1	-0.575374601	2.89E-06
SIDT2	0.244567429	0.000519534
SIGIRR	-0.427141731	1.70E-12
SIGLEC10	2.941013836	1.82E-202
SIGLEC11	0.931273489	4.12E-201
SIGLEC12	0.560368512	1.07E-133
SIGLEC15	0.26183945	2.57E-228
SIGLEC5	-0.106617331	0.001447702
SIGLEC6	0.038082739	9.35E-32
SIGLEC8	2.966137472	1.99E-212
SIGLECL1	0.0082788	8.67E-05
SIGMAR1	1.989664983	3.83E-215
SIK1	0.249984079	1.20E-06
SIK2	0.744551367	2.35E-33
SIK3	-0.317142014	2.87E-06
SIKE1	1.849019811	1.00E-175
SIL1	2.115459176	3.42E-286
SIM1	0.017511794	0.544191144
SIM2	0.185841591	0.000896722
SIMC1	0.249864445	0.000427072
SIN3A	1.370952522	2.83E-114
SIN3B	0.375927276	2.21E-06
SIPA1	2.272134062	5.19E-292
SIPA1L1	0.19916376	0.024110787
SIPA1L2	2.232495206	7.23E-184
SIPA1L3	1.169760637	8.63E-70
SIRPA	0.51473849	2.36E-11
SIRPB1	0.343040031	2.15E-11
SIRPB2	1.743403199	8.06E-172

SIRPD	0.263576393	2.75E-166
SIRPG	0.49156806	1.81E-277
SIRT1	0.675948005	9.08E-27
SIRT2	0.395445997	3.07E-09
SIRT3	0.504167444	1.76E-16
SIRT4	0.809080631	2.18E-32
SIRT5	-0.061129205	0.295190353
SIRT6	1.197507686	5.69E-84
SIRT7	-0.1707658	0.03435149
SIVA1	1.023462595	9.41E-109
SIX2	0.863701989	9.52E-157
SIX3	-1.626358285	1.35E-20
SIX4	0.732895079	2.27E-67
SIX5	0.538221681	1.21E-29
SIX6	0.92450869	1.02E-59
SKA2	1.739026915	3.85E-136
SKAP1	0.395347933	7.81E-28
SKAP2	2.222317563	1.96E-132
SKI	1.133307947	2.19E-23
SKIDA1	0.300726109	9.73E-17
SKIL	1.745552302	4.14E-245
SKIV2L	1.214690531	8.82E-58
SKOR1	-0.222085108	0.018718582
SKOR2	0.355674619	1.06E-30
SKP1	0.643972826	1.71E-24
SKP2	0.205525281	0.00047157
SLA	2.704173972	2.13E-245
SLA2	0.451605097	1.88E-40
SLAIN1	0.918282027	4.28E-27
SLAIN2	1.711576693	4.46E-233
SLAMF1	0.402384653	1.05E-253
SLAMF7	0.828207095	5.76E-240
SLAMF8	0.372334495	5.74E-05
SLBP	1.794970014	6.93E-149
SLC10A1	-0.049307035	0.003578292
SLC10A2	0.030231044	1.65E-07
SLC10A3	1.990032454	2.21E-250
SLC10A4	1.800005089	1.50E-82
SLC10A5	-0.477165124	5.31E-15
SLC11A1	2.534515863	9.10E-101
SLC11A2	1.380083129	8.44E-97
SLC12A1	-0.324266669	6.53E-23
SLC12A2	-0.074270342	0.3355261
SLC12A3	0.007450884	0.360182665
SLC12A4	1.947181621	6.68E-207
SLC12A5	-3.425380464	5.10E-98
SLC12A6	0.335215108	1.54E-07
SLC12A7	1.316495803	1.19E-108

SLC12A8	0.852018054	5.72E-37
SLC12A9	1.047048265	3.33E-48
SLC13A1	0.004973847	0.002597704
SLC13A2	-0.018322944	1.97E-05
SLC13A3	-0.23071301	0.00073161
SLC13A4	0.26959641	0.000144128
SLC13A5	-1.334680943	3.64E-59
SLC14A1	-0.406591683	0.002834149
SLC14A2	0.210385451	2.38E-118
SLC15A1	0.084078455	2.14E-40
SLC15A2	1.663332405	9.20E-108
SLC15A3	1.513808925	2.36E-104
SLC15A4	1.102470258	2.75E-61
SLC15A5	-0.069363236	8.27E-07
SLC16A1	2.914741916	5.72E-252
SLC16A10	0.922160199	7.16E-74
SLC16A11	-0.057588485	0.278490769
SLC16A12	0.34127967	1.92E-29
SLC16A14	0.372495943	1.21E-06
SLC16A2	2.244947458	8.26E-270
SLC16A3	2.256592891	2.76E-175
SLC16A5	0.676914684	6.69E-48
SLC16A6	0.429898646	8.69E-14
SLC16A7	0.083720979	0.167903073
SLC16A8	-0.688015064	4.50E-29
SLC16A9	1.152292005	3.03E-30
SLC17A1	0.162707805	2.45E-79
SLC17A2	0.005213194	6.24E-06
SLC17A3	0.109451521	7.97E-97
SLC17A4	-0.062945106	1.41E-05
SLC17A5	1.657235662	3.48E-142
SLC17A6	-0.395486641	0.001138573
SLC17A7	-1.997470649	2.31E-10
SLC17A8	0.451712779	2.59E-19
SLC17A9	0.848759753	6.09E-69
SLC18A1	0.593364582	2.56E-114
SLC18A2	-0.153466971	0.035578211
SLC18A3	0.147824351	0.087170169
SLC18B1	1.065609984	7.70E-74
SLC19A1	0.246856587	3.07E-07
SLC19A2	1.219935032	3.84E-59
SLC19A3	0.200225961	2.54E-05
SLC1A1	0.084645483	0.215125016
SLC1A2	-1.741409691	1.09E-30
SLC1A3	1.416862785	2.61E-51
SLC1A4	0.535582278	2.34E-11
SLC1A5	3.253484835	1.45E-279
SLC1A6	-2.277038423	1.40E-58

SLC1A7	-0.176805405	0.004986056
SLC20A1	1.190883668	1.63E-48
SLC20A2	1.334825596	7.27E-95
SLC22A1	0.276051324	3.75E-74
SLC22A10	-0.214046272	2.14E-13
SLC22A11	0.070670767	3.18E-22
SLC22A12	0.012506545	0.098976139
SLC22A13	-0.093849871	6.55E-08
SLC22A14	-0.55285901	1.57E-24
SLC22A15	-0.756631851	3.60E-28
SLC22A16	0.121200313	3.13E-65
SLC22A17	-0.653897137	4.71E-12
SLC22A18	1.715574869	2.84E-131
SLC22A18AS	0.771377584	1.58E-82
SLC22A2	0.014262836	0.309283935
SLC22A23	0.279693819	6.83E-06
SLC22A24	-0.007835566	0.011177617
SLC22A25	-0.024284113	0.061882459
SLC22A3	-0.159150069	0.001141476
SLC22A31	-1.59367489	8.72E-11
SLC22A4	1.417331023	5.37E-152
SLC22A5	-0.781054038	5.20E-21
SLC22A6	-0.838373937	6.28E-33
SLC22A7	-0.195179865	2.94E-09
SLC22A8	-0.137624775	0.000418904
SLC22A9	-0.326062884	5.16E-15
SLC23A1	0.089357015	0.008155358
SLC23A2	0.601779643	4.53E-21
SLC23A3	-0.28244798	1.09E-19
SLC24A1	0.568119738	6.42E-44
SLC24A2	-1.524801177	1.67E-56
SLC24A3	0.607681696	3.88E-11
SLC24A4	-0.295994092	0.000102419
SLC24A5	0.022603572	0.004875572
SLC25A1	1.962661947	1.85E-257
SLC25A10	-1.063206572	1.55E-75
SLC25A11	0.920275091	2.42E-54
SLC25A12	-0.116532615	0.217679718
SLC25A13	2.439386029	1.69E-236
SLC25A14	0.701462778	1.40E-19
SLC25A16	0.585792388	2.87E-26
SLC25A17	1.762157478	2.31E-148
SLC25A18	-1.194541002	6.00E-45
SLC25A19	1.38792661	5.39E-125
SLC25A2	0.085917842	3.24E-17
SLC25A20	2.067787566	1.12E-241
SLC25A21	-0.01494655	0.429105603
SLC25A22	-0.813364738	9.02E-12

SLC25A23	-0.560257961	8.84E-12
SLC25A24	1.758619814	2.48020954212306e-321
SLC25A25	1.071930381	3.38E-38
SLC25A26	0.960417294	1.12E-70
SLC25A27	-1.975465601	3.13E-91
SLC25A28	-0.155350146	0.002323688
SLC25A29	-0.379290192	6.76E-07
SLC25A3	1.323439307	1.06E-89
SLC25A30	0.104512503	0.023503179
SLC25A31	-0.052241436	7.38E-09
SLC25A33	1.097338409	1.34E-71
SLC25A34	-0.47037609	6.07E-06
SLC25A35	0.942521283	4.75E-97
SLC25A36	0.773403732	1.20E-30
SLC25A37	0.529915941	9.29E-21
SLC25A38	1.269885147	7.19E-88
SLC25A39	1.399980239	5.31E-150
SLC25A4	0.516392542	1.83E-08
SLC25A40	1.181112373	5.39E-61
SLC25A41	-1.485324214	2.60E-52
SLC25A42	-0.308146643	1.99E-05
SLC25A43	2.000492967	3.48E-232
SLC25A44	1.20778725	8.61E-63
SLC25A45	0.164749602	0.001866961
SLC25A46	1.179227969	1.31E-54
SLC25A47	0.077432374	3.63E-16
SLC25A48	-0.677156253	3.37E-24
SLC25A5	1.994099829	2.06E-208
SLC25A51	1.041663702	2.88E-117
SLC25A52	0.089111558	1.73E-36
SLC25A6	2.005446288	3.88E-216
SLC26A1	-0.033304572	0.602084843
SLC26A11	0.879208738	2.20E-30
SLC26A2	1.688474445	1.71E-87
SLC26A3	0.004226914	0.685096151
SLC26A4	0.061420846	0.361783093
SLC26A5	-0.458780996	2.24E-14
SLC26A6	0.35137722	6.44E-07
SLC26A7	0.760035289	5.92E-222
SLC26A8	-1.602800358	1.91E-71
SLC26A9	-0.206743378	7.26E-06
SLC27A1	0.275097856	1.89E-05
SLC27A2	-0.833179122	2.52E-31
SLC27A3	1.480922541	4.93E-58
SLC27A4	1.182827707	3.95E-53
SLC27A5	-0.154171042	0.000214853
SLC27A6	0.411335241	1.51E-07
SLC28A2	-0.042466612	0.000248381

SLC28A3	0.121665061	3.42E-12
SLC29A1	1.653215518	9.32E-63
SLC29A2	0.255394097	0.017536137
SLC29A3	0.832387921	7.88E-72
SLC29A4	1.682892335	2.87E-98
SLC2A1	0.830339057	6.72E-42
SLC2A11	-1.521928889	3.66E-71
SLC2A12	-0.791314465	1.58E-35
SLC2A13	0.113600563	0.232253378
SLC2A14	0.118294829	5.74E-08
SLC2A2	0.040468122	3.46E-40
SLC2A3	0.182299737	0.043273189
SLC2A4	-0.305451997	3.84E-10
SLC2A4RG	1.367255851	5.62E-146
SLC2A5	2.915808361	5.11E-189
SLC2A6	0.249093775	0.001091543
SLC2A7	0.059712165	3.82E-70
SLC2A8	0.895977651	2.24E-49
SLC30A1	1.38806311	1.48E-176
SLC30A10	-0.029867252	0.542069652
SLC30A2	0.024389271	0.112385664
SLC30A3	-0.47498277	0.006698698
SLC30A4	0.322902633	7.07E-08
SLC30A5	1.718239026	4.58E-197
SLC30A7	1.906337661	1.67E-250
SLC30A8	-0.033393107	0.006626589
SLC30A9	1.018879626	3.64E-49
SLC31A2	-0.743048033	8.82E-22
SLC32A1	-1.830453862	5.09E-34
SLC33A1	0.808056841	1.87E-51
SLC34A1	-0.000893433	0.669194235
SLC34A2	0.991964147	1.24E-88
SLC34A3	0.340444253	2.21E-39
SLC35A1	-0.342516176	8.24E-07
SLC35A3	0.419036618	5.99E-18
SLC35A5	1.790691239	6.85E-164
SLC35B1	0.923492539	1.18E-53
SLC35B3	0.429553161	9.91E-14
SLC35B4	1.676491821	7.43E-93
SLC35C2	1.490956936	2.59E-172
SLC35D1	1.685937053	1.62E-166
SLC35D2	-0.017967147	0.8159392
SLC35D3	-0.652319822	1.01E-10
SLC35E1	1.392990304	3.31E-95
SLC35E2B	0.698666268	7.93E-16
SLC35E3	1.00596191	2.01E-100
SLC35E4	0.553933932	1.74E-29
SLC35F1	1.798696303	5.03E-89

SLC35F3	-2.266079708	1.32E-105
SLC35F4	-0.72508338	2.16E-13
SLC35F5	1.654045832	2.08E-216
SLC35G1	0.725735543	2.95E-85
SLC35G2	1.402881377	3.43E-87
SLC35G3	0.015687358	4.79E-08
SLC35G4	0.003070737	0.021366087
SLC35G5	-0.006838494	0.74078051
SLC35G6	0.144892211	1.82E-15
SLC36A1	0.563984905	3.27E-05
SLC36A2	-0.008012123	0.542738537
SLC36A3	0.006774932	0.002982574
SLC36A4	0.4597408	9.10E-11
SLC37A1	0.478346004	2.99E-13
SLC37A2	2.250976406	1.65511991356818e-321
SLC37A3	0.98690341	2.86E-42
SLC37A4	0.986726916	1.21E-43
SLC38A1	0.136692607	0.209747106
SLC38A10	1.013033974	5.82E-68
SLC38A11	-0.02052935	0.764715823
SLC38A2	1.437138057	2.82E-73
SLC38A3	1.653143977	2.24E-151
SLC38A4	0.422464354	5.45E-133
SLC38A5	2.283869004	2.02E-203
SLC38A6	0.7447104	6.11E-55
SLC38A7	1.224305663	7.49E-85
SLC38A8	-0.307873984	1.19E-10
SLC38A9	0.888665684	4.12E-83
SLC39A10	1.128739007	6.23E-50
SLC39A11	1.476864345	1.51E-176
SLC39A12	-0.384380768	0.002088757
SLC39A13	0.746413373	2.04E-23
SLC39A14	2.858768132	8.47E-193
SLC39A2	0.111925895	1.01E-05
SLC39A3	1.26670171	2.63E-101
SLC39A4	0.548889982	8.00E-15
SLC39A5	-0.00298871	0.907911946
SLC39A6	2.09390528	1.89E-176
SLC39A7	2.338153284	4.44E-229
SLC39A9	1.61841532	6.54E-188
SLC3A1	-0.355647817	9.79E-31
SLC3A2	1.810291663	4.27E-243
SLC41A1	1.088053391	9.47E-58
SLC41A2	1.418479764	4.65E-99
SLC41A3	0.166602612	0.025115015
SLC43A1	1.077544987	2.86E-126
SLC43A2	1.443935948	2.79E-78
SLC43A3	1.900413301	4.21E-307

SLC44A1	1.263198789	8.12E-47
SLC44A2	1.61032356	6.85E-161
SLC44A3	1.409520497	4.77E-80
SLC44A4	0.205951067	2.37E-28
SLC44A5	1.384393797	2.45E-108
SLC45A1	0.47965874	1.70E-09
SLC45A2	0.014544146	0.378460972
SLC45A3	-0.150246634	0.191025258
SLC45A4	-0.135916875	0.140384041
SLC46A1	-0.394810258	2.29E-10
SLC46A2	-0.164132676	0.000529403
SLC46A3	0.841283279	8.42E-44
SLC47A1	0.952322972	8.53E-47
SLC47A2	1.951114293	3.08E-76
SLC48A1	0.020999428	0.722060076
SLC4A1	-0.188372456	6.61E-07
SLC4A10	-0.988199102	1.88E-14
SLC4A11	0.583363972	3.80E-23
SLC4A1AP	0.066581551	0.410730447
SLC4A2	2.413262575	1.67E-286
SLC4A3	0.439406292	8.51E-06
SLC4A4	1.737021167	6.03E-85
SLC4A5	-0.806052107	1.35E-36
SLC4A7	1.774078392	1.27E-196
SLC4A8	0.138246775	0.11662988
SLC4A9	-0.268998562	6.00E-21
SLC50A1	1.671812447	1.77E-190
SLC51A	0.58893223	3.53E-117
SLC51B	1.888907418	2.24E-187
SLC52A1	0.423967949	2.34E-221
SLC52A2	1.263183215	6.62E-50
SLC52A3	0.297221991	7.05E-05
SLC5A1	0.00308841	0.725112256
SLC5A10	0.031730223	6.23E-05
SLC5A11	-1.091500681	4.80E-15
SLC5A12	-0.045511772	0.233866046
SLC5A2	-0.131098838	2.65E-05
SLC5A3	1.35376434	2.52E-109
SLC5A4	0.17409785	5.56E-10
SLC5A5	-0.343920433	2.60E-06
SLC5A6	0.670498431	2.13E-22
SLC5A7	-0.436834348	2.37E-11
SLC5A8	-0.159818074	1.67E-25
SLC5A9	0.805769657	2.01E-262
SLC6A1	-2.528625641	1.63E-223
SLC6A11	0.77199514	1.55E-11
SLC6A12	-1.522832639	6.59E-129
SLC6A13	-1.370960838	1.26E-116

SLC6A14	0.063476912	1.56E-39
SLC6A15	-1.715980153	3.07E-75
SLC6A16	0.355652359	4.13E-23
SLC6A17	-2.676257746	2.75E-74
SLC6A18	0.00967751	5.06E-06
SLC6A19	-0.002931693	0.296927491
SLC6A2	0.08090485	9.34E-38
SLC6A20	-0.164825683	2.46E-05
SLC6A3	-0.289807406	0.007396712
SLC6A4	0.019302172	0.244991774
SLC6A5	-0.202103129	2.12E-05
SLC6A6	0.269787591	0.001235909
SLC6A7	-2.432640196	2.44E-31
SLC6A8	1.193290795	1.31E-87
SLC6A9	2.370511236	2.41E-134
SLC7A1	1.603099968	4.81E-131
SLC7A10	-1.926695504	1.00E-91
SLC7A11	0.769369026	3.06E-28
SLC7A13	0.001564937	0.222276671
SLC7A14	-1.406208755	1.99E-47
SLC7A2	0.691163912	1.92E-21
SLC7A3	1.633774417	1.81E-126
SLC7A4	-1.395617914	3.20E-47
SLC7A5	1.595798264	4.35E-118
SLC7A6	-0.24521943	0.001897038
SLC7A6OS	-0.249860354	3.46E-06
SLC7A8	0.32606463	0.000744063
SLC7A9	-0.001088423	0.983589583
SLC8A1	0.496581849	2.16E-11
SLC8A2	-2.749399983	1.19E-60
SLC8A3	0.150153763	0.022895308
SLC8B1	1.373436806	5.17E-143
SLC9A1	0.804572969	8.48E-23
SLC9A2	-0.404602451	4.83E-39
SLC9A3	-0.425781438	5.57E-08
SLC9A3R1	0.003731498	0.962646436
SLC9A3R2	-0.03448902	0.491377402
SLC9A4	0.12711912	1.20E-112
SLC9A5	-1.476293671	6.85E-41
SLC9A6	-0.672163984	9.14E-18
SLC9A7	-0.163249155	0.106055003
SLC9A8	1.152220203	1.62E-76
SLC9A9	1.893642098	9.58E-135
SLC9B1	0.721349154	9.74E-190
SLC9B2	0.16750938	0.040301056
SLC9C1	0.071366656	1.53E-105
SLC9C2	-0.05822489	0.043048713
SLCO1A2	-2.263053584	5.24E-101

SLCO1B1	0.000952886	0.711476133
SLCO1B3	-0.010801195	0.020914644
SLCO1C1	0.782171553	1.42E-13
SLCO2A1	2.183723322	4.12E-226
SLCO2B1	1.548132408	6.83E-96
SLCO3A1	-0.181977494	0.010176895
SLCO4A1	-0.747207451	9.93E-13
SLCO4C1	-0.111709929	2.70E-07
SLCO5A1	0.704551355	2.00E-175
SLCO6A1	0.065190788	3.26E-62
SLF1	0.390642301	2.92E-06
SLF2	0.46903904	1.96E-13
SLFN11	1.836065156	7.46E-226
SLFN12L	-0.04448769	0.000830092
SLFN13	0.894523138	1.99E-161
SLFN14	0.02079279	6.85E-13
SLFN5	1.616500311	2.01E-206
SLFNL1	-0.039386254	0.304132551
SLIRP	0.402367163	3.22E-14
SLIT1	1.120653647	2.33E-17
SLIT2	-0.665852106	2.83E-12
SLIT3	-1.307195352	8.51E-36
SLITRK1	-0.528892469	9.21E-10
SLITRK2	0.182288878	0.00314366
SLITRK3	1.617516545	1.02E-89
SLITRK4	-1.682797396	4.51E-74
SLITRK5	-0.714314666	1.70E-21
SLITRK6	-0.567179437	1.62E-16
SLK	-0.066412263	0.314563047
SLMAP	0.591936086	4.95E-23
SLN	3.179052911	9.56E-168
SLTM	-0.260765319	3.23E-05
SLU7	0.864460024	2.00E-44
SLURP1	-0.192487422	8.69E-14
SLX1A	-0.847556386	2.05E-63
SLX1B	-0.115662296	1.82E-16
SLX4	0.219116522	0.033336456
SLX4IP	1.309980015	3.80E-186
SMAD1	2.616131776	2.59E-289
SMAD2	1.270276815	2.14E-248
SMAD3	0.986518246	1.64E-49
SMAD4	1.03065124	1.96E-45
SMAD5	1.924963878	1.10E-217
SMAD6	0.115342118	0.03252446
SMAD7	-0.464427614	1.49E-09
SMAD9	0.992336551	3.02E-39
SMAGP	1.258725404	1.91E-251
SMAP1	0.494467789	5.81E-14

SMAP2	1.087149389	2.39E-39
SMARCA1	1.519045275	3.51E-114
SMARCA2	0.110673442	0.100065081
SMARCA4	0.309328109	3.18E-05
SMARCA5	1.58282701	1.13E-168
SMARCAD1	1.598676894	2.28E-103
SMARCAL1	1.4833853	2.76E-198
SMARCB1	-0.68241688	1.31E-21
SMARCC1	1.743711139	2.90E-244
SMARCC2	-0.245248261	0.001529013
SMARCD1	1.682186895	2.80E-133
SMARCD2	0.965601749	1.56E-21
SMARCD3	0.486733112	1.21E-13
SMARCE1	-2.077861201	6.51E-299
SMC1A	1.670352971	1.17E-97
SMC1B	0.184175505	2.63E-74
SMC3	0.841148167	6.01E-33
SMC5	1.500122381	1.86E-71
SMC6	0.611496588	2.40E-25
SMCHD1	0.928241312	6.20E-35
SMCO1	-0.065182512	4.66E-07
SMCO2	0.191851557	8.29E-16
SMCO3	-0.080218157	2.31E-10
SMCO4	2.001754051	5.63E-154
SMCP	-0.168677166	0.001005902
SMCR8	1.143555005	8.04E-53
SMDT1	0.893226087	3.20E-66
SMG1	-0.172441209	0.081884399
SMG5	0.675602983	4.11E-26
SMG6	0.635581219	1.61E-25
SMG7	0.877436298	9.92E-33
SMG8	1.760612783	9.77E-172
SMG9	1.200587803	1.01E-77
SMIM1	0.280610833	1.43E-08
SMIM10	1.120718245	5.70E-67
SMIM10L1	0.788195037	3.31E-52
SMIM10L2A	-1.453614412	1.69E-62
SMIM10L2B	-2.029817437	1.92E-81
SMIM11A	0.089885034	1.71E-241
SMIM11B	0.0635462	3.82E-249
SMIM12	1.212192135	3.04E-109
SMIM13	0.433313212	6.78E-08
SMIM14	1.270992205	7.56E-93
SMIM15	1.807669031	1.92E-261
SMIM17	-0.275606994	0.003874237
SMIM18	-0.331537693	0.000252042
SMIM19	1.256224916	1.06E-100
SMIM2	-0.047571683	4.21E-05

SMIM20	1.803189888	4.80E-301
SMIM21	0.015446288	5.26E-16
SMIM22	-0.743596083	1.67E-32
SMIM23	0.095819298	7.20E-191
SMIM24	0.143095322	0.062679444
SMIM4	1.631934748	1.97E-170
SMIM5	0.552523143	1.08E-13
SMIM6	-0.405905506	1.68E-07
SMIM7	1.142713406	3.80E-135
SMIM8	-0.585431221	1.04E-29
SMIM9	0.034951932	9.21E-32
SMKR1	0.837189088	1.22E-23
SMLR1	0.007588253	0.209928364
SMN1	1.181302049	6.35E-127
SMN2	0.817277111	4.04E-35
SMNDC1	1.156599164	3.70E-77
SMOC1	0.626680316	5.52E-14
SMOC2	0.797651378	6.29E-33
SMOX	1.835830229	4.54E-210
SMPD1	1.815621247	9.56E-142
SMPD2	1.007833485	3.23E-78
SMPD3	-1.434725832	9.93E-47
SMPD4	0.611985811	1.55E-15
SMPDL3A	1.460487318	1.84E-75
SMPDL3B	-0.055822794	0.399242168
SMPX	-0.779015062	3.56E-20
SMR3A	0.002645199	0.363243479
SMR3B	-0.027366167	0.014784905
SMS	2.040751222	1.09E-150
SMTN	0.036884002	0.654205698
SMTNL1	1.093741269	1.14E-108
SMTNL2	-0.029712982	0.320928977
SMU1	1.617874514	2.01E-162
SMUG1	1.681543696	2.19E-265
SMURF1	0.888798024	1.21E-49
SMURF2	1.020505959	6.14E-85
SMYD1	0.028776578	0.352615406
SMYD2	1.669054971	5.81E-73
SMYD3	1.274535103	9.66E-75
SMYD4	1.149575703	2.07E-79
SMYD5	1.270850205	1.25E-95
SNAI1	1.049834386	2.07E-49
SNAI2	2.141876427	1.50E-127
SNAI3	0.38833007	1.07E-15
SNAP23	-0.032892972	0.528567494
SNAP25	-3.044937286	5.52E-69
SNAP29	1.157234785	5.78E-110
SNAP47	0.76938636	2.27E-26

SNAP91	-2.494145473	8.48E-76
SNAPC1	1.477799368	1.12E-162
SNAPC2	1.664228927	2.16E-118
SNAPC3	0.865358713	6.89E-37
SNAPC4	0.46036782	4.33E-07
SNAPC5	0.643689187	1.06E-21
SNCA	-2.817959283	2.10E-145
SNCAIP	1.676380821	6.55E-100
SNCB	-3.205339431	2.05E-88
SNCG	-3.066348393	2.10E-155
SND1	2.078563586	3.11E-169
SNED1	-0.531821647	2.92E-09
SNF8	0.33335004	2.04E-13
SNIP1	1.432427537	7.43E-138
SNN	0.556523331	1.40E-15
SNPH	-1.420864959	1.45E-51
SNRK	0.360831153	1.86E-05
SNRNP200	1.648616216	3.42E-85
SNRNP25	1.040511538	1.17E-72
SNRNP27	1.582116299	1.58E-171
SNRNP35	0.534068252	1.83E-29
SNRNP40	2.462533847	8.64E-244
SNRNP48	0.270772958	0.000998227
SNRNP70	-0.375876175	0.000146525
SNRPA	1.216386529	2.38E-99
SNRPA1	0.716703946	4.70E-24
SNRPB	2.572396362	2.40E-209
SNRPB2	2.254809487	3.15E-297
SNRPD1	2.036640969	2.16E-304
SNRPD3	-0.129656011	0.018207322
SNRPE	2.179882112	3.79E-264
SNRPF	1.693991688	3.06E-223
SNTA1	0.100672334	0.177825856
SNTB1	1.749179534	4.97E-216
SNTB2	1.261081258	2.24E-126
SNTG1	0.93554273	1.93E-57
SNTG2	-0.063142704	0.019719715
SNTN	-0.006537437	0.621884984
SNU13	0.841067747	7.91E-33
SNUPN	0.351724268	2.29E-12
SNURF	-0.368400244	1.21E-11
SNW1	1.257253206	2.08E-88
SNX1	1.200754126	2.03E-95
SNX10	0.876799146	3.55E-17
SNX11	1.381506187	4.66E-184
SNX12	1.659095215	5.86E-195
SNX13	1.330785018	2.90E-125
SNX14	0.874658082	3.06E-37

SNX16	0.736449793	4.07E-44
SNX17	1.707925841	5.30E-163
SNX18	1.696797395	1.75E-188
SNX19	0.14291619	0.005747159
SNX2	0.480859369	4.15E-15
SNX20	1.438148137	4.81E-178
SNX21	0.211196	0.000107895
SNX22	-0.720349542	1.69E-22
SNX24	1.346874656	2.81E-101
SNX25	-0.054621677	0.554675082
SNX27	-0.552177605	5.16E-19
SNX29	0.813189297	6.17E-64
SNX3	2.112795944	7.80E-277
SNX30	0.05846825	0.435057873
SNX31	0.92574077	9.13E-109
SNX32	-1.449249103	2.61E-53
SNX33	1.361171328	5.39E-169
SNX4	1.948136019	6.12E-191
SNX5	1.320901007	1.33E-199
SNX6	1.773865015	8.65E-175
SNX7	2.949026611	5.54E-293
SNX8	1.563988481	7.80E-161
SNX9	1.092830164	3.14E-96
SOAT2	0.085824147	3.06E-24
SOBP	0.034592526	0.581991548
SOCS1	1.746447048	1.17E-82
SOCS3	3.423191103	1.80E-107
SOCS4	1.645661181	2.12E-238
SOCS5	0.382676679	6.90E-06
SOCS6	2.072445021	6.80E-200
SOCS7	-0.09967253	0.368625843
SOD1	0.860081667	1.80E-44
SOD2	2.978130489	3.66E-259
SOD3	1.242017309	6.12E-54
SOGA1	0.932479063	1.28E-12
SOGA3	-2.356090281	7.64E-190
SOHLH1	-2.135904872	1.13E-50
SOHLH2	0.161850025	0.00013103
SON	1.210305424	3.17E-70
SORBS1	0.27962453	1.29E-05
SORBS2	-0.873598757	3.48E-31
SORBS3	0.912426904	4.49E-45
SORCS1	-0.560617798	2.75E-08
SORCS2	-0.334558852	5.38E-06
SORCS3	0.203438141	0.012938558
SORD	0.82431085	4.71E-48
SORL1	0.317458364	0.000149176
SORT1	1.171163857	3.22E-39

SOS1	0.899475951	2.19E-46
SOS2	0.96864038	1.45E-80
SOST	0.2236389	7.75E-42
SOSTDC1	0.129113661	0.09738863
SOWAHA	-3.22467852	9.80E-134
SOWAHB	-0.384116707	8.25E-06
SOWAHC	2.172276865	9.56E-284
SOWAHD	1.423096757	1.91273392483974e-311
SOX1	0.0183511	0.799664239
SOX12	1.878033786	9.78E-139
SOX13	1.706560087	5.12E-212
SOX14	-0.117787238	0.004780189
SOX15	0.91206038	3.37E-36
SOX17	0.303026103	1.74E-11
SOX18	0.865811839	1.60E-39
SOX21	2.25334949	2.19E-203
SOX3	1.279924628	4.28E-112
SOX30	0.142947068	2.39E-52
SOX5	1.155059889	2.04E-121
SOX6	1.493281479	9.50E-222
SOX7	-0.678697596	2.87E-34
SOX8	1.106425806	2.20E-41
SOX9	2.602691257	1.38E-197
SP1	1.605009926	4.65E-91
SP100	1.729388496	4.42E-154
SP110	1.642560267	1.84E-152
SP140	0.815137525	3.63E-148
SP140L	1.851178678	1.24045061701362e-318
SP2	1.51946665	1.07E-107
SP3	0.936261647	1.66E-59
SP4	1.54778304	4.69E-115
SP5	1.0210462	7.27E-98
SP6	1.180293291	1.28E-125
SP7	-0.096231907	6.14E-08
SP8	1.149726174	2.44E-112
SP9	-0.800070238	7.90E-12
SPA17	1.367541715	1.52E-146
SPAAR	0.685350145	5.17E-54
SPACA1	0.000451864	0.848465787
SPACA3	0.097681721	4.29E-42
SPACA4	0.138384554	9.08E-18
SPACA5	0.081307638	2.83E-71
SPACA5B	0.000236887	0.926798909
SPACA6	-1.236639411	1.63E-79
SPACA7	0.001256367	0.689772404
SPACA9	0.83563215	2.59E-65
SPAG1	1.225106017	5.84E-79
SPAG11A	0.001278406	0.251860907

SPAG11B	0.000773029	0.46548235
SPAG16	1.172855281	1.20E-134
SPAG17	0.00716729	0.920620528
SPAG4	1.781682181	1.11E-199
SPAG5	1.229083703	2.54E-61
SPAG6	-0.532970955	4.22E-08
SPAG7	1.242616838	7.60E-102
SPAG8	-0.098548723	0.242895593
SPAG9	0.610714215	8.59E-26
SPAM1	-0.000216913	0.886434349
SPANXA1	0.000774776	0.258174175
SPANXA2	-0.005907557	0.057460269
SPANXB1	0.040878318	2.70E-06
SPANXC	0.041017141	1.02E-16
SPANXD	0.001414681	0.661055191
SPANXN1	0.000124482	0.960173821
SPANXN2	-0.003182555	0.158538437
SPANXN3	-0.011855026	0.015792499
SPANXN4	-0.005739875	0.037426117
SPANXN5	0.000900411	0.770267678
SPARC	3.355869093	1.15E-188
SPARCL1	1.577543224	6.26E-55
SPAST	0.452411546	1.65E-14
SPATA1	0.079120804	0.000402074
SPATA13	0.937739897	1.07E-30
SPATA16	0.013076355	1.57E-06
SPATA17	1.140744962	3.04E-156
SPATA18	0.863765803	4.31E-30
SPATA19	-0.06759312	5.85E-06
SPATA2	0.416134137	4.12E-10
SPATA20	0.323189271	3.78E-05
SPATA21	-0.168815902	1.13E-06
SPATA22	0.290194293	3.10E-32
SPATA24	1.646848442	1.09288678552479e-314
SPATA25	0.961479881	2.29E-46
SPATA2L	1.155454478	2.93E-51
SPATA3	0.217997302	2.15E-34
SPATA31A1	0.000825129	0.694380722
SPATA31A3	0.000546336	0.430895743
SPATA31A5	-7.48E-05	0.893091834
SPATA31A6	-0.011464935	0.001715556
SPATA31A7	-0.001164478	0.592348819
SPATA31D1	-0.000371109	0.747357399
SPATA31D3	0.000519855	0.410624051
SPATA31D4	-8.10E-05	0.891091565
SPATA31E1	0.001337656	0.715906654
SPATA32	0.143007225	1.42E-67
SPATA33	1.317617838	1.78E-162

SPATA4	0.477590867	1.51E-59
SPATA45	0.502208695	1.76E-88
SPATA46	0.162755062	3.00E-06
SPATA5	1.205653422	2.28E-236
SPATA5L1	1.236388908	3.11E-66
SPATA6L	-0.218292099	2.70E-06
SPATA7	-0.062402648	0.388040137
SPATA9	0.364085922	3.52E-37
SPATC1	0.062207818	0.014922639
SPATC1L	1.123180081	5.75E-67
SPATS1	-0.246961572	3.21E-09
SPATS2	1.22456141	4.06E-44
SPATS2L	1.472681213	1.17E-160
SPCS1	0.292086223	6.04E-08
SPCS2	1.898358106	4.75695649266383e-315
SPCS3	1.400255655	2.11E-88
SPDEF	-0.645089434	3.90E-36
SPDL1	1.31614154	1.38E-84
SPDYA	0.188731347	7.51E-16
SPDYC	0.125048904	2.47E-42
SPDYE1	0.262701722	1.44E-54
SPDYE16	-0.001559229	0.831289411
SPDYE17	0.0235043	3.81E-25
SPDYE2B	0.094061488	3.88E-80
SPDYE3	-0.653675634	6.95E-98
SPDYE4	0.015426815	0.005443921
SPDYE5	0.398700869	6.15E-82
SPDYE6	0.365015347	8.08E-73
SPECC1	1.091202974	3.77E-98
SPECC1L	0.249967908	0.000590397
SPEF1	0.025875681	0.847182542
SPEF2	-0.615179477	1.05E-31
SPEG	0.239984288	0.008553107
SPEM1	0.014550067	0.080145685
SPEN	0.480211676	1.13E-09
SPESP1	0.021074244	0.707579137
SPG11	1.070896241	2.31E-76
SPG21	2.024773055	1.80E-306
SPG7	-0.973088937	1.11E-41
SPHK1	2.19103499	1.80E-179
SPHK2	0.14171903	0.044189132
SPHKAP	-1.803069484	2.16E-30
SPI1	3.588278118	1.50E-307
SPIB	0.141707619	3.17E-24
SPIC	0.035794441	5.95E-19
SPICE1	-0.795803269	2.06E-76
SPIDR	1.16540033	1.02E-51
SPIN1	0.809006128	3.48E-34

SPIN2A	-0.229663846	1.13E-15
SPIN2B	0.905024577	3.80E-57
SPIN3	0.077629146	0.312161015
SPIN4	1.443981378	7.37E-214
SPINK1	0.554317987	7.30E-41
SPINK13	0.19699978	4.75E-44
SPINK14	0.007990012	9.01E-07
SPINK2	0.302254519	2.98E-16
SPINK4	0.033134478	2.01E-05
SPINK5	-0.105931292	5.37E-07
SPINK6	-0.124262788	0.23026991
SPINK7	-0.034213965	0.015609035
SPINK9	-0.159488411	6.41E-12
SPINT1	0.914481521	3.42E-16
SPINT2	-3.222587583	1.03E-214
SPINT3	0.00258324	0.299838502
SPINT4	0.005995953	0.002147085
SPIRE1	0.798173129	2.55E-33
SPIRE2	-0.784924714	2.68E-16
SPN	1.403906838	6.95E-258
SPNS2	-0.694038976	1.21E-20
SPNS3	0.733939391	2.37E-77
SPO11	0.006113182	0.000280777
SPOCK1	-0.910708671	6.04E-16
SPOCK2	-0.163838922	0.087287132
SPOCK3	-1.869783603	7.79E-78
SPON1	0.992839333	2.75E-24
SPON2	1.09625768	9.81E-18
SPOP	0.982290587	2.14E-48
SPOPL	0.491065068	2.25E-22
SPP1	4.96309544	1.70E-167
SPP2	0.039835625	1.60E-06
SPPL2B	0.505526813	2.81E-09
SPPL2C	-0.453201175	2.33E-13
SPPL3	1.155901521	5.63E-99
SPR	2.020789388	5.62E-131
SPRED1	1.820558874	5.00E-235
SPRED2	1.983967174	5.84E-196
SPRED3	1.379481704	7.15E-84
SPRN	-3.024844026	7.89E-190
SPRR1A	-0.357141714	5.94E-15
SPRR1B	-0.325356086	5.82E-20
SPRR2A	-0.232505278	4.04E-10
SPRR2B	-0.012262032	0.013377224
SPRR2D	-0.062928687	0.002388468
SPRR2E	-0.2755059	3.31E-16
SPRR2F	-0.034183497	0.006378155
SPRR2G	-0.388234884	6.08E-29

SPRR3	-0.716484056	1.95E-25
SPRR4	-0.02848974	0.004393881
SPRY2	1.981269524	7.11E-126
SPRY3	-0.013744513	0.856232846
SPRY4	2.39990912	1.74E-260
SPRYD3	-0.508393125	7.14E-09
SPRYD4	0.232035518	0.001576161
SPRYD7	0.340734661	1.29E-07
SPSB1	2.600232631	3.38E-213
SPSB2	1.983855531	2.08E-307
SPTA1	0.040051096	4.58E-17
SPTAN1	-0.374603782	2.20E-05
SPTB	-2.116338643	2.46E-60
SPTBN1	-0.329842474	2.87E-13
SPTBN2	-2.034279085	3.18E-52
SPTBN4	-1.649134548	1.93E-44
SPTBN5	-0.55445988	4.66E-07
SPTLC1	1.943671137	9.02E-183
SPTLC2	0.886331414	1.76E-33
SPTLC3	1.153593492	1.02E-262
SPTSSB	0.008708425	0.943182232
SPTY2D1	1.440216667	5.26E-132
SPX	-2.665149228	1.45E-223
SPZ1	0.022907497	1.86E-20
SQLE	1.22661114	5.77E-44
SQSTM1	1.389900578	2.32E-82
SRA1	1.311149057	3.84E-72
SRC	1.354575249	2.69E-89
SRCAP	-2.881941203	6.14E-224
SRCIN1	-1.94332288	2.11E-79
SRD5A1	1.170041462	1.11E-63
SRD5A2	0.158305176	6.13E-39
SRD5A3	0.598436252	7.21E-25
SREBF1	0.30442991	2.37E-06
SREBF2	-0.110678021	0.217352065
SREK1	0.239391858	5.83E-05
SREK1IP1	1.155959519	2.34E-154
SRF	1.123762799	1.47E-66
SRGAP1	1.230666912	6.58E-196
SRGAP2	0.384054285	0.003718347
SRGAP2B	0.757413166	7.32E-18
SRGAP2C	0.672196516	1.79E-15
SRGAP3	-0.632781558	5.82E-10
SRGN	3.193547552	1.01E-181
SRI	1.761464677	3.55E-205
SRL	-0.002681443	0.933977
SRM	1.900461234	3.93E-274
SRMS	0.41976071	1.73E-28

SRP14	1.184255746	3.27E-110
SRP19	-1.111213443	4.33E-72
SRP54	0.005441166	0.934362235
SRP68	0.32947583	4.82E-07
SRP72	1.884620684	1.04E-149
SRP9	1.587205731	7.08E-180
SRPK1	0.110109479	0.186404311
SRPK2	0.905860048	2.39E-32
SRPK3	-0.411587828	1.34E-05
SRPRA	1.472212825	1.16E-85
SRPRB	2.087494833	1.15E-255
SRR	1.668894846	3.39E-247
SRRD	0.373862186	7.73E-09
SRRM1	0.228365807	0.010308508
SRRM2	0.439924114	1.87E-05
SRRM3	-2.238284168	3.41E-48
SRRM4	-2.247508049	2.21E-44
SRRM5	-0.248231226	3.14E-05
SRRT	1.160487971	4.14E-47
SRSF1	-1.106473083	2.46E-59
SRSF10	0.997741921	1.02E-45
SRSF11	0.772994172	1.41E-17
SRSF12	0.734777335	7.35E-30
SRSF2	0.763107347	4.67E-26
SRSF3	1.700317596	7.72E-155
SRSF4	-0.168449772	0.009589382
SRSF5	-0.281549275	0.001459162
SRSF6	1.170914471	2.99E-55
SRSF7	1.488773842	2.13E-95
SRSF8	1.10446711	3.03E-86
SRSF9	-0.10973931	0.055830576
SRY	0.693346602	4.19E-124
SS18	1.518250461	3.63E-204
SS18L1	0.135793653	0.180474012
SS18L2	-0.55832672	1.34E-19
SSB	0.809344992	2.37E-38
SSBP1	0.975658057	1.88E-70
SSBP2	1.281534559	1.13E-76
SSBP3	-0.20355893	0.024332938
SSBP4	0.438309754	7.08E-09
SSC5D	0.402917104	1.21E-07
SSH1	0.765609136	3.00E-22
SSH2	0.653831055	7.92E-26
SSH3	0.353175894	0.000822046
SSMEM1	0.050382646	2.67E-12
SSNA1	1.349132447	2.84E-116
SSPN	1.286820473	1.51E-113
SSR1	2.164825843	2.94E-308

SSR2	0.770875228	3.93E-50
SSRP1	1.164418969	9.22E-66
SST	-2.179602049	5.77E-19
SSTR1	-1.371205481	1.41E-43
SSTR2	-0.905488791	2.16E-14
SSTR3	-1.05142155	7.12E-28
SSTR4	-0.456143215	3.77E-23
SSTR5	0.048059549	0.032326161
SSU72	0.882726594	6.57E-51
SSUH2	0.111961768	9.03E-13
SSX1	0.162820261	2.96E-94
SSX2	0.005688356	3.64E-09
SSX2B	0.001713407	0.077831635
SSX2IP	0.351547037	0.000417024
SSX3	0.03363132	9.21E-39
SSX4	-0.000433252	0.68622599
SSX4B	0.006356109	2.59E-06
SSX5	0.016697771	0.005617842
SSX7	-0.000617183	0.555509603
ST13	1.462603564	2.64E-145
ST18	-1.740234195	8.44E-45
ST20	-0.586728038	1.41E-23
ST3GAL1	0.797979503	2.33E-42
ST3GAL2	1.034039289	2.46E-46
ST3GAL4	1.053088016	2.87E-58
ST3GAL5	0.225365976	0.000131894
ST3GAL6	0.976916626	1.29E-50
ST6GAL1	1.839698923	1.03E-126
ST6GAL2	0.784655375	5.33E-38
ST6GALNAC1	-0.282787057	2.80E-09
ST6GALNAC2	0.099615012	0.086591087
ST6GALNAC3	0.676461801	8.52E-29
ST6GALNAC4	1.792909213	5.69E-214
ST6GALNAC5	-0.57326207	1.27E-05
ST7	1.249553927	2.82E-62
ST7L	0.997877356	3.16E-94
ST8SIA1	0.90768464	4.74E-42
ST8SIA2	0.526426429	7.72E-29
ST8SIA3	-2.250864454	3.64E-69
ST8SIA5	-0.08426956	0.572871361
ST8SIA6	0.056506875	0.158666188
STAB1	2.603433452	8.75E-179
STAB2	0.155747949	1.33E-177
STAC	1.563360171	5.39E-50
STAC2	0.189692496	0.173990877
STAC3	1.794737553	1.51E-240
STAG1	1.363419692	3.19E-76
STAG2	1.926867851	4.21E-196

STAG3	0.404765854	7.57E-08
STAM	0.289541936	0.000144971
STAM2	1.574554707	1.04E-209
STAMBP	1.721541908	1.87E-174
STAMBPL1	0.0913619	0.178762202
STAP1	0.412332406	1.60E-64
STAP2	1.329971438	1.35E-187
STAR	-1.214386211	7.86E-46
STARD10	-0.417964514	5.48E-12
STARD13	0.75402449	1.10E-40
STARD3	0.533525843	1.55E-17
STARD3NL	1.706396079	1.34E-192
STARD4	-0.217382865	0.008387553
STARD5	-1.037283465	8.85E-31
STARD6	0.526721112	2.46E-140
STARD7	1.376090552	8.50E-127
STARD8	1.346536669	2.74E-109
STARD9	-0.777622328	6.79E-19
STAT1	1.885092338	2.48E-149
STAT2	1.026866922	8.35E-31
STAT3	1.691407069	1.80E-154
STAT4	-1.24067047	7.67E-30
STAT5A	1.46295896	8.55E-104
STAT5B	0.540168006	1.46E-11
STAT6	0.677465073	6.95E-20
STATH	-0.028347815	0.015189241
STAU1	1.753749175	6.76E-194
STAU2	0.561689609	3.82E-14
STBD1	-0.62725036	3.00E-21
STC1	2.331333963	2.65E-124
STEAP1B	0.121104155	0.01055387
STEAP2	-0.866919977	7.79E-22
STEAP4	0.134153747	1.79E-06
STH	-0.23633688	6.85E-07
STIM1	0.822470745	5.13E-28
STIM2	1.227748443	3.71E-87
STIP1	1.292998607	3.30E-61
STK10	1.509964487	2.06E-30
STK11	0.431226971	9.08E-10
STK11IP	0.687965374	8.76E-15
STK16	1.320724182	3.91E-161
STK19	0.285858195	7.94E-05
STK24	0.578627915	1.87E-21
STK25	0.323177875	8.84E-07
STK26	0.069170367	0.421576987
STK3	1.018578667	8.19E-107
STK31	-0.388374066	7.73E-16
STK32A	1.163372275	1.80E-47

STK32B	2.269467354	4.10E-303
STK32C	0.048230433	0.575000184
STK33	1.113351983	5.96E-95
STK35	0.851529706	3.20E-38
STK36	1.202619897	5.00E-47
STK38	1.547587806	3.42E-104
STK38L	1.662689429	8.55E-188
STK39	0.952516682	6.21E-43
STK4	1.443862052	1.69E-185
STK40	2.07303297	3.16E-220
STKLD1	0.247679034	1.83E-15
STMN1	0.869905639	1.22E-16
STMN2	-2.328139166	3.57E-45
STMN3	0.099265816	0.266810362
STMN4	-0.911999827	3.50E-21
STMND1	-0.008923862	0.793588289
STN1	-0.215271393	0.000163003
STOM	1.272909734	2.95E-62
STOML1	0.378005163	1.81E-08
STOML2	2.242567136	8.87E-230
STOML3	0.252067519	0.000847626
STON1	1.682957829	1.04E-73
STON1-GTF2A1L	-0.156281656	1.11E-07
STON2	0.210053062	0.039879973
STOX1	-0.729814136	4.57E-16
STOX2	-0.489251719	5.54E-11
STPG1	-0.3467071	9.65E-08
STPG2	0.042744789	3.24E-12
STPG3	0.109046349	1.04E-05
STRA8	-0.037998783	0.01273683
STRADA	-2.553635731	5.57E-263
STRADB	1.660222955	1.77E-212
STRAP	1.498999958	2.10E-125
STRBP	0.844855161	2.54E-36
STRC	-1.029929778	1.66E-21
STRIP1	0.44376935	6.64E-07
STRIP2	0.030683322	0.826223523
STRN	-0.048162314	0.508424283
STRN3	0.7774091	1.17E-46
STRN4	0.870100072	1.19E-53
STS	0.050331525	0.525042622
STT3A	2.413207056	2.32E-295
STT3B	1.844190249	1.07E-189
STUB1	-0.670843278	4.64E-26
STUM	-0.964222487	2.17E-16
STX10	1.868069717	4.77E-158
STX11	0.914707346	4.87E-64
STX12	1.574727532	1.66E-154

STX16	-0.042934439	0.589709413
STX17	1.124216956	2.88E-108
STX18	0.495597508	3.22E-19
STX19	0.042399698	0.054071962
STX1A	-1.314596612	5.97E-15
STX1B	-2.136547852	2.33E-55
STX2	1.313957867	7.65E-100
STX3	0.114354437	0.172786145
STX4	1.101630783	4.33E-80
STX5	1.471017343	4.02E-115
STX6	1.236874769	1.30E-74
STX7	0.634814457	8.39E-28
STX8	1.979490849	3.19619232241944e-310
STXBP1	-1.543826933	3.44E-29
STXBP2	0.651967389	4.09E-26
STXBP3	0.953661606	8.16E-52
STXBP5	-1.043569736	5.60E-31
STXBP5L	-1.414133627	5.57E-25
STXBP6	-1.435714822	2.78E-48
STYK1	-0.803609471	6.61E-17
STYX	1.717252788	3.67E-249
STYXL1	2.051138241	2.20E-303
SUB1	1.115308345	3.40E-56
SUCLA2	-0.286320681	0.000320271
SUCLG1	0.913731022	2.14E-54
SUCLG2	2.332993017	2.72E-267
SUCO	1.750881877	3.22E-200
SUDS3	1.011072907	3.37E-65
SUFU	0.94929689	1.42E-74
SUGP1	0.555751844	7.69E-19
SUGP2	0.307749866	0.001481957
SUGT1	0.313736436	8.51E-09
SULF1	2.039723642	1.51E-161
SULF2	2.032578447	3.73E-217
SULT1A1	-2.358156317	3.37E-189
SULT1A2	-1.317163206	2.42E-102
SULT1A3	-0.520334279	4.31E-50
SULT1A4	-0.529629898	1.70E-37
SULT1B1	0.335186797	2.58E-21
SULT1C2	0.109337845	0.001493335
SULT1C3	0.004484124	0.000974956
SULT1C4	2.166267687	2.21E-190
SULT1E1	0.038655468	0.001034244
SULT2A1	-0.037977713	0.000962815
SULT2B1	0.079854528	0.015284738
SULT4A1	-3.312031039	8.15E-106
SULT6B1	0.050546478	8.31E-41
SUMF2	2.44733777	1.99E-276

SUMO1	1.393090659	4.59E-104
SUMO2	1.702837716	3.22E-159
SUMO3	1.433238742	6.02E-90
SUMO4	0.144416844	5.47E-07
SUN1	0.156239584	0.111548368
SUN2	-0.191857776	0.011682454
SUN3	0.119032177	1.10E-18
SUN5	0.019102883	3.65E-09
SUOX	1.273148116	4.29E-151
SUPT16H	1.172613033	1.52E-47
SUPT20H	0.083545587	0.277411077
SUPT3H	0.172360758	0.000227439
SUPT5H	0.225155117	0.000548937
SUPT6H	0.576871335	8.77E-19
SUPT7L	0.566518435	2.67E-14
SUPV3L1	0.058844332	0.373897504
SURF1	1.645855149	2.77E-245
SURF2	0.928443643	1.48E-57
SURF6	0.890856541	3.56E-69
SUSD1	2.141702771	4.46E-158
SUSD2	1.470190085	5.37E-85
SUSD3	2.528584604	3.22E-206
SUSD4	0.084046374	0.410221417
SUSD5	-0.16642194	0.011200921
SUSD6	1.82959897	5.40E-60
SUV39H1	1.582584112	5.24E-166
SUV39H2	1.608138612	2.29E-203
SUZ12	1.218763271	8.34E-70
SV2A	0.331913711	0.004208994
SV2B	-2.270058072	1.54E-46
SV2C	-1.499473928	1.57E-29
SVBP	1.012048651	6.49E-66
SVEP1	0.108827462	0.272188256
SVIL	0.630559133	4.66E-40
SVIP	-0.651362016	4.25E-19
SVOP	-2.698174699	4.32E-81
SVOPL	0.177703585	2.28E-20
SWAP70	1.552438184	2.90E-141
SWI5	1.289709561	2.19E-160
SWSAP1	1.118149857	1.21E-238
SWT1	1.108806469	2.68E-149
SYAP1	1.781909965	6.61E-218
SYBU	-0.107270285	0.318137477
SYCE1	-2.899080856	3.61E-166
SYCE1L	0.439307324	2.96E-12
SYCE2	0.973253265	7.10E-62
SYCE3	1.165768886	4.05E-117
SYCN	-0.321630688	1.15E-11

SYCP1	-0.020251301	0.000239963
SYCP2	-0.225038337	6.55E-06
SYCP2L	-0.423465337	5.99E-15
SYCP3	-0.242349846	7.82E-08
SYDE2	0.176756731	1.63E-22
SYF2	1.137270192	7.53E-125
SYK	3.050618424	2.28E-295
SYMPK	0.503621904	4.80E-14
SYN1	-2.626525878	3.85E-72
SYN2	-2.199222949	2.77E-45
SYN3	-0.123616774	0.036027611
SYNC	0.936782505	8.11E-72
SYNCRIP	1.471575284	3.07E-172
SYNDIG1	0.82087867	4.70E-17
SYNDIG1L	-2.883028033	3.61E-31
SYNE1	-1.51173794	9.91E-55
SYNE2	0.7774431	5.83E-22
SYNE3	0.186366506	7.81E-05
SYNE4	-0.927818453	2.75E-11
SYNGAP1	0.192991832	0.035271825
SYNGR1	-1.554011532	3.62E-32
SYNGR2	1.918559264	1.77E-124
SYNGR3	-2.521872742	1.22E-79
SYNGR4	-0.099340476	0.2678788
SYNJ1	-0.147896447	0.134754991
SYNJ2	-0.717208997	1.24E-11
SYNJ2BP	0.717250139	4.85E-52
SYNM	0.446986855	2.85E-09
SYNPO	0.281430906	0.012416904
SYNPO2	-0.08507555	0.148129382
SYNPO2L	0.21207512	7.15E-40
SYNPR	-3.4450486	8.81E-103
SYNRG	0.749967524	6.39E-41
SYP	-1.996268154	1.62E-48
SYPL1	2.400889279	3.45E-160
SYPL2	0.340195865	1.12E-23
SYS1	1.912256382	3.89135537737391e-314
SYT1	-2.397106792	9.67E-43
SYT10	-0.243264824	5.09E-17
SYT11	1.544356736	1.46E-88
SYT12	-1.676744057	2.62E-37
SYT13	-2.008523482	4.06E-38
SYT14	-0.598426299	9.92E-17
SYT15	-0.369810682	5.18E-34
SYT16	-0.785504082	7.97E-17
SYT17	1.131352474	2.29E-36
SYT2	-1.693871923	3.36E-24
SYT3	-1.021065327	6.81E-21

SYT4	-2.593399823	5.77E-48
SYT5	-1.77056208	7.02E-32
SYT6	1.221617746	1.71E-37
SYT7	-1.680057082	2.73E-31
SYT8	0.083961582	4.69E-19
SYT9	-1.234778715	5.04E-54
SYTL1	-1.318610127	2.33E-13
SYTL2	1.072952321	1.46E-23
SYTL3	2.054561764	7.95E-234
SYTL4	1.519625492	6.10E-107
SYTL5	-0.672543489	1.03E-07
SYVN1	0.859863021	1.19E-32
SZT2	-0.380337725	1.18E-06
TAAR1	0.001295468	0.45918206
TAAR2	0.002860218	0.019637901
TAAR5	0.033215871	1.81E-24
TAAR6	0.00184395	0.750313115
TAAR8	0.008264295	0.021705067
TAAR9	-0.001358105	0.605592899
TAB1	0.968127623	3.59E-54
TAB2	1.808052316	4.28E-220
TAB3	0.680002249	6.53E-36
TAC1	-1.811219135	6.31E-18
TAC3	-0.510259631	0.000257852
TAC4	0.891943398	1.21E-277
TACC1	0.224734979	1.10E-05
TACC2	-0.15742701	0.034687118
TACO1	1.235917518	8.05E-102
TACR1	0.603207806	3.87E-15
TACR2	0.002597395	0.948003441
TACR3	-0.259334573	1.43E-10
TACSTD2	0.655421244	1.40E-71
TADA1	1.749851355	4.85E-129
TADA2A	1.276524437	6.17E-86
TADA2B	1.090503606	9.67E-78
TADA3	1.467121265	1.61E-244
TAF1	0.774790289	4.02E-32
TAF11	0.763901151	7.75E-35
TAF13	1.855276876	6.86E-203
TAF15	0.105057454	0.075301623
TAF1A	1.595580661	1.21E-243
TAF1B	1.770980411	6.03E-224
TAF1C	0.297492046	0.000195589
TAF1D	-0.375497866	2.69E-11
TAF1L	0.088544085	6.06E-140
TAF2	1.437312643	1.64E-97
TAF3	0.295730984	1.10E-09
TAF4	0.46552739	4.85E-10

TAF4B	0.043884665	0.502740554
TAF5	0.598764998	8.09E-19
TAF5L	1.533338466	4.07E-182
TAF6	0.778242317	1.98E-21
TAF6L	0.639662897	3.20E-33
TAF7	2.285620606	4.24E-196
TAF7L	0.094099508	0.047073243
TAF8	1.400949466	4.17E-283
TAF9	0.662823298	1.11E-19
TAF9B	1.872521787	3.69E-194
TAGAP	1.804690593	2.07E-184
TAGLN	0.86634223	4.43E-28
TAGLN3	-1.465222399	1.94E-30
TAL1	0.760912169	9.15E-40
TAL2	0.305152304	3.52E-36
TALDO1	0.935817843	3.92E-53
TAMM41	0.541780514	7.56E-23
TANC1	1.388042096	6.00E-83
TANC2	0.149223138	0.044632941
TANGO2	-0.40266222	2.06E-11
TANGO6	1.307607477	1.11E-156
TANK	1.794606218	2.65E-244
TAOK1	0.756495033	3.95E-42
TAOK2	0.480176969	2.32E-12
TAOK3	0.209518062	0.00081096
TAP1	2.348654419	5.96E-225
TAP2	0.847946965	7.43E-40
TAPBP	1.618023607	1.03E-149
TAPT1	0.458008401	2.59E-24
TARBP1	-0.521571521	9.11E-07
TARBP2	1.265202029	5.20E-35
TARDBP	0.250730189	0.000279084
TARM1	0.016307516	2.90E-05
TARS2	1.070562074	8.11E-61
TAS1R2	0.005834303	0.291679943
TAS1R3	0.095436939	0.024282621
TAS2R1	-0.069335343	3.58E-07
TAS2R10	0.201204725	1.74E-13
TAS2R13	-0.008585843	0.317207384
TAS2R14	-0.137492421	0.081208709
TAS2R16	-0.001746547	0.236078931
TAS2R19	0.310581203	5.26E-09
TAS2R20	-0.091451253	0.063333192
TAS2R3	-0.104177074	1.59E-05
TAS2R30	-0.000307245	0.979629742
TAS2R31	0.513340312	1.81E-64
TAS2R38	0.008422344	0.000130149
TAS2R39	-0.013184984	0.002481892

TAS2R4	-1.112815961	3.42E-48
TAS2R40	0.012120447	0.024959913
TAS2R41	-0.000651446	0.799664239
TAS2R42	-0.001780914	0.861080567
TAS2R43	-0.006598537	0.780521755
TAS2R46	-0.008212788	0.660578139
TAS2R5	0.21403706	0.00602882
TAS2R50	-0.00774082	0.679148865
TAS2R60	0.032471711	1.43E-10
TAS2R7	-0.008131145	0.068121893
TAS2R8	-0.04590757	8.26E-07
TAS2R9	-0.011297313	0.046157641
TASP1	1.206155176	1.97E-77
TAT	0.186633698	1.35E-45
TATDN1	0.078562823	0.192477768
TATDN2	0.014374301	0.818383715
TATDN3	1.055664529	1.82E-76
TAX1BP1	1.584659872	2.90E-159
TAZ	-0.403430656	1.17E-08
TBATA	0.037955558	5.33E-22
TBC1D1	1.520697827	1.41E-189
TBC1D10A	-0.569540215	4.36E-25
TBC1D10B	0.403347099	2.20E-09
TBC1D10C	0.26253214	7.00E-08
TBC1D12	0.234825502	0.002383325
TBC1D13	0.780134828	5.54E-48
TBC1D14	0.747701248	2.65E-35
TBC1D15	1.216904379	5.43E-103
TBC1D16	0.92450362	6.49E-30
TBC1D17	-0.33833056	1.76E-07
TBC1D19	1.227564449	2.35E-117
TBC1D2	0.272302073	0.000148956
TBC1D20	1.075850511	2.66E-58
TBC1D21	0.056970321	2.48E-16
TBC1D22A	1.024176606	4.07E-104
TBC1D22B	1.224534532	4.71E-81
TBC1D23	1.758594855	8.10E-167
TBC1D24	-0.073006909	0.430921836
TBC1D25	0.978120416	4.08E-35
TBC1D26	-1.305994268	1.78E-66
TBC1D28	-0.048440793	3.87E-07
TBC1D2B	0.750104363	3.65E-33
TBC1D3	0.008577996	0.237778201
TBC1D30	-1.363183423	2.46E-66
TBC1D31	0.20422126	0.000775468
TBC1D32	0.741507043	1.71E-64
TBC1D3B	0.030220419	0.060838346
TBC1D3C	-0.005510369	0.003041979

TBC1D3D	-0.153894511	0.00136681
TBC1D3E	0.033367723	0.356943263
TBC1D3F	0.058888563	0.000164544
TBC1D3G	0.039745583	2.18E-16
TBC1D3H	0.004600151	0.326559814
TBC1D3I	0.003839785	0.136233834
TBC1D3K	0.005777203	0.313011605
TBC1D3L	-0.528770202	3.12E-07
TBC1D4	0.229583986	5.30E-06
TBC1D5	1.235416066	1.44E-121
TBC1D7	1.471947477	2.24E-177
TBC1D8	0.647524044	1.61E-16
TBC1D8B	0.318186109	7.27E-24
TBC1D9	-0.065180948	0.604397795
TBC1D9B	0.112203791	0.074544438
TBCA	1.737917659	2.61E-204
TBCB	0.821863388	4.91E-48
TBCC	1.431987493	1.01E-88
TBCCD1	2.08071706	1.41E-240
TBCD	0.66346642	6.86E-18
TBCEL	0.885943021	3.82E-73
TBCK	0.934618941	5.83E-51
TBK1	-0.900571888	2.43E-44
TBKBP1	0.280864751	0.000935711
TBL1X	0.570424725	2.34E-28
TBL1XR1	0.879600757	4.68E-51
TBL1Y	-0.004620036	0.748766672
TBL3	0.989519362	1.15E-54
TBP	1.598801647	1.13E-115
TBPL1	-0.307521229	9.07E-06
TBPL2	0.003539569	0.273950881
TBR1	-0.873370602	1.13E-07
TBRG1	-0.790193809	2.51E-31
TBRG4	1.510210838	9.56E-113
TBX1	0.77160239	9.03E-105
TBX10	0.011421047	0.263133317
TBX18	0.175083932	1.94E-06
TBX19	0.982954318	1.12E-95
TBX2	1.463421993	5.27E-132
TBX20	0.105366466	1.29E-42
TBX21	0.499856089	3.90E-134
TBX22	-0.010117787	0.114962594
TBX3	0.262413604	2.34E-06
TBX4	0.280065236	5.52E-187
TBX5	1.070111554	4.29E-158
TBX6	0.762601575	2.12E-96
TBXAS1	2.883649226	1.79E-290
TC2N	1.456157954	1.67E-179

TCAF1	0.745835296	1.17E-19
TCAF2	1.607424232	1.01E-262
TCAIM	1.140118145	2.10E-100
TCAP	-0.170679941	0.015116706
TCEA2	0.780640852	4.04E-30
TCEA3	1.597175339	1.13E-197
TCEAL1	0.506600191	2.98E-11
TCEAL2	-2.058166126	1.03E-128
TCEAL3	-0.061251825	0.26565918
TCEAL4	0.618604457	6.84E-32
TCEAL5	-0.733640524	2.02E-16
TCEAL6	-2.544804479	8.42E-122
TCEAL7	0.724889571	2.21E-18
TCEAL8	1.605076768	2.93E-157
TCEAL9	1.374798088	1.25E-95
TCEANC	0.697307474	2.65E-51
TCEANC2	0.903534122	9.67E-109
TCERG1	-0.077728074	0.427195344
TCERG1L	-1.385076303	6.97E-37
TCF15	-0.157131887	1.88E-06
TCF19	2.978228334	9.75E-194
TCF20	0.356360852	2.40E-07
TCF21	-0.012444487	0.034217044
TCF24	0.350546565	4.18E-60
TCF25	0.113062972	0.129997195
TCF4	1.115793649	2.28E-52
TCF7	1.088586661	1.34E-115
TCF7L1	2.138357267	2.02E-306
TCF7L2	1.135953629	2.22E-106
TCFL5	-0.177338779	0.009701096
TCHH	-0.151935471	2.44E-08
TCHHL1	0.000258097	0.8585229
TCHP	-0.371557866	5.60E-08
TCIRG1	1.729862678	3.13E-136
TCL1A	0.058729263	2.36E-29
TCL1B	-0.003752324	0.091619678
TCN1	0.21571496	5.82E-43
TCN2	2.49124039	1.61902032534419e-315
TCOF1	-0.205619142	0.000820017
TCP1	0.879261336	5.45E-41
TCP10L	-1.472032994	3.20E-152
TCP10L2	-0.015627891	0.006813851
TCP11	0.142557229	1.77E-35
TCP11L1	0.772718006	1.85E-34
TCP11L2	1.141800378	1.57E-95
TCP11X1	-0.017580343	1.10E-05
TCP11X2	-0.018438814	4.09E-06
TCTA	1.584219332	1.88E-201

TCTE1	-0.792758507	2.90E-20
TCTN1	1.595388648	1.99E-183
TCTN2	1.09492401	1.12E-48
TCTN3	2.077629061	5.99E-282
TDG	1.55072351	7.99E-145
TDGF1	-0.323854211	3.15E-17
TDGF1P3	0.032973393	8.60E-05
TDO2	1.359853624	3.54E-171
TDP1	1.467838946	2.87E-142
TDP2	1.180567986	1.45E-76
TDRD1	0.046790079	5.17E-08
TDRD10	-0.512516654	1.74E-47
TDRD12	-0.118721746	0.00026203
TDRD15	0.003905737	0.04480965
TDRD3	0.044663903	0.339138183
TDRD5	-0.413007648	3.00E-16
TDRD6	-1.036414025	7.72E-27
TDRD7	1.389983505	3.29E-165
TDRD9	-0.787121436	1.21E-31
TDRKH	0.353160238	1.51E-05
TDRP	0.167558904	0.009914366
TEAD1	1.976687359	3.92E-275
TEAD3	2.275478358	9.90E-308
TEAD4	2.282875833	4.73E-223
TEC	0.335423227	3.82E-74
TECPR1	0.565027416	1.56E-14
TECPR2	0.214386276	3.97E-05
TECR	-0.634262634	2.48E-24
TECRL	-0.006539044	0.71207503
TECTA	0.133933937	0.005760665
TECTB	0.346533093	6.70E-73
TEDDM1	-0.077379872	8.13E-07
TEF	-1.299060899	2.46E-38
TEFM	1.428905206	5.59E-156
TEK	0.008497393	0.876858455
TEKT1	1.185448725	2.37E-46
TEKT2	1.184367464	9.88E-64
TEKT3	0.205148476	5.93E-05
TEKT4	-0.264156192	1.72E-05
TEKT5	-0.050325783	0.022437275
TELO2	1.001716311	1.08E-37
TENM1	0.279692642	0.033726598
TENM2	-0.47615117	7.11E-08
TENM3	-0.285370307	0.000131933
TENM4	1.93552251	1.01E-167
TEP1	1.204566266	2.75E-97
TEPP	-0.087561189	2.44E-05
TEPSIN	-0.469589401	5.93E-11

TERB1	0.021192563	0.000643463
TERB2	0.002761258	0.095988525
TERF1	-0.544749228	5.28E-16
TERF2	1.03983322	5.39E-60
TERF2IP	-0.349705955	5.51E-06
TERT	0.416131403	4.77E-50
TES	1.848999141	1.16E-297
TESC	-0.42490466	0.000328569
TESK1	0.878127887	1.32E-43
TESK2	-0.242795047	0.000369937
TESMIN	-0.036764424	0.249782167
TESPA1	-1.337444293	9.93E-11
TET1	0.268045162	2.93E-16
TET2	1.290230915	1.04E-232
TET3	0.952587604	9.86E-35
TEX10	1.464308089	1.51E-101
TEX101	-0.071273102	0.000118058
TEX11	0.107126166	2.27E-41
TEX12	0.04217182	0.004898442
TEX13A	6.22E-05	0.96540962
TEX13B	-0.010279369	0.005747159
TEX14	-0.002463865	0.95583986
TEX15	-0.064145825	0.005013522
TEX19	0.099498747	2.41E-60
TEX2	-0.184602206	0.000313257
TEX22	0.724877974	2.35E-132
TEX26	1.505250432	4.52E-93
TEX261	1.951999602	1.32E-230
TEX28	0.000556017	0.581126025
TEX29	-0.415023461	1.03E-06
TEX30	0.953895172	1.08E-56
TEX33	-0.010816196	0.024601956
TEX35	0.003603733	0.251828857
TEX36	0.026081024	5.33E-15
TEX37	-0.001611157	0.722974701
TEX38	0.238132197	1.35E-68
TEX43	0.173264993	2.17E-17
TEX44	0.017684858	0.061560385
TEX9	0.781520835	1.48E-60
TF	-3.225655651	2.38E-102
TFAM	1.472295064	5.91E-133
TFAP2A	1.136338736	1.54E-82
TFAP2B	0.090906712	0.328795658
TFAP2C	0.311570063	9.60E-19
TFAP2D	-0.034993369	0.111337219
TFAP2E	-1.456016905	5.90E-33
TFAP4	-0.512676658	3.90E-10
TFB1M	0.786662424	2.59E-60

TFB2M	2.135278449	2.31E-194
TFCP2	1.353953017	1.98E-124
TFCP2L1	1.240606039	1.17E-129
TFDP1	1.813027716	1.40E-193
TFDP2	1.359331637	1.18E-128
TFDP3	0.025550711	3.53E-26
TFE3	1.217943387	1.47E-88
TFEB	0.944593639	9.83E-44
TFEC	1.610949223	1.06E-278
TFF1	-0.076410949	3.38E-05
TFF2	-0.057328633	0.000205532
TFF3	0.550847358	1.37E-45
TFG	0.564876458	1.42E-20
TFIP11	0.94851999	2.43E-54
TFPI2	1.396822885	3.01E-109
TFPT	0.055697108	0.338037179
TFR2	0.220308085	0.029887476
TFRC	1.87134043	1.97E-135
TG	-0.148443547	0.007327035
TGDS	1.501945612	4.37E-169
TGFA	0.215451732	0.003994078
TGFB2	2.549509879	6.87E-212
TGFB3	0.901191579	2.54E-30
TGFBR1	2.80377279	3.21E-236
TGFBR2	1.779189356	4.23E-131
TGFBR3	0.81569027	2.40E-52
TGFBR3L	-1.178153866	6.07E-44
TGFBRAP1	1.564472772	1.80E-109
TGIF2LX	0.001275854	0.321934151
TGIF2LY	8.72E-05	0.953717891
TGM1	-0.439565921	2.91E-05
TGM2	0.378420388	4.73E-05
TGM3	-0.524532972	4.65E-38
TGM4	0.07398995	7.46E-14
TGM6	0.004560846	0.012201785
TGM7	0.032146861	3.14E-10
TGOLN2	1.255316746	2.16E-60
TGS1	1.672703946	3.53E-138
TH	-1.211083671	1.61E-15
THAP1	1.390655592	2.20E-140
THAP10	1.114626203	3.69E-64
THAP11	1.793939503	6.27E-187
THAP12	0.625025501	2.94E-40
THAP2	1.027085621	2.71E-63
THAP3	0.838220697	7.29E-45
THAP4	1.229102446	1.83E-109
THAP5	1.420606234	2.07E-117
THAP6	0.810034258	3.34E-52

THAP7	0.259855056	1.61E-06
THAP8	1.690020848	3.04E-242
THAP9	0.867521941	1.95E-83
THBD	2.032107972	1.36E-170
THBS1	2.774033071	1.37E-247
THBS2	1.096846208	6.12E-42
THBS3	0.907903793	2.01E-36
THBS4	1.233410326	7.88E-57
THEG	-0.066581503	0.001163512
THEGL	0.287378996	4.04E-132
THEM4	-0.460041893	5.63E-12
THEM5	0.361346888	1.36E-11
THEM6	0.458498731	3.76E-14
THEMIS	-0.007087625	0.90477539
THEMIS2	2.648493917	1.31E-165
THNSL1	0.961344963	9.55E-88
THNSL2	-0.254302848	0.001971585
THOC1	-0.492583607	1.00E-13
THOC2	0.849658046	1.42E-48
THOC3	0.913356979	3.26E-28
THOC5	1.126675746	8.99E-67
THOC6	1.599896618	2.75E-145
THOC7	0.844649011	2.96E-51
THOP1	0.72078872	2.76E-21
THPO	-0.515020192	1.43E-08
THRA	-0.090678798	0.180413003
THRAP3	1.455412094	6.73E-104
THRB	-0.206146435	0.046723006
THRSP	-0.117992907	0.108329794
THSD1	1.430240898	2.06E-133
THSD4	-0.197052466	6.99E-05
THSD7A	1.3200384	1.94E-163
THSD7B	0.03683316	0.215818883
THTPA	0.263908825	7.07E-07
THUMPD1	0.879719786	9.90E-43
THUMPD2	0.22104362	0.007374604
THUMPD3	1.498853006	2.37E-152
THY1	-0.153632453	0.215125016
THYN1	0.971647869	5.69E-44
TIA1	0.877501321	6.40E-25
TIAF1	-1.271448844	2.17E-46
TIAL1	0.706039572	1.84E-18
TIAM1	-0.33199239	0.024391067
TIAM2	0.02117565	0.823046644
TICAM1	1.742213265	6.40E-171
TICAM2	-1.394541827	3.75E-250
TIE1	1.356327582	3.65E-105
TIFA	1.541665331	4.41E-105

TIFAB	0.078209347	5.80E-37
TIGAR	0.514540766	1.94E-27
TIGD1	-0.079489821	0.139145082
TIGD2	1.551841596	2.62E-135
TIGD3	0.462316662	1.63E-08
TIGD5	1.378948179	3.85E-104
TIGD6	1.542346182	5.82859911005442e-314
TIGD7	-0.125512402	0.009452794
TIGIT	0.26094108	1.13E-47
TIMD4	0.672745429	1.79E-175
TIMM10	1.979303525	3.21E-237
TIMM10B	1.08129135	8.59E-81
TIMM13	1.755175922	2.04E-254
TIMM17A	1.588077468	9.06E-124
TIMM17B	1.177591036	2.82E-98
TIMM21	1.292166454	1.90E-145
TIMM22	1.614453062	1.76E-142
TIMM23	1.340924805	9.97E-108
TIMM23B	-0.354051504	5.98E-09
TIMM29	1.715338414	1.96E-191
TIMM44	0.857342956	1.55E-35
TIMM50	1.474638518	6.69E-134
TIMM8A	1.468879141	1.52E-202
TIMM9	1.214674537	3.24E-99
TIMMDC1	1.185717127	1.00E-125
TIMP2	2.526443307	1.70E-170
TIMP3	2.426326734	8.58E-234
TIMP4	4.249282009	1.78E-224
TINAG	-0.067808922	5.65E-07
TINAGL1	-0.329421738	6.25E-08
TINCR	-0.535381041	8.34E-14
TINF2	1.782715644	1.44E-172
TIPARP	2.626122441	2.76676761671098e-322
TIPIN	1.873108219	1.26E-306
TIPRL	2.031075851	5.06E-168
TIRAP	0.822814682	4.15E-73
TJAP1	0.576245889	4.03E-14
TJP1	0.934002077	1.32E-64
TJP3	0.071926457	0.000871585
TK2	0.03352537	0.479971403
TKFC	0.979487133	2.82E-73
TKT	0.296335864	9.73E-07
TKTL1	1.385098194	7.62E-283
TKTL2	0.090055957	1.42E-34
TLCD1	1.799967789	9.56E-139
TLCD2	1.134289502	1.70E-242
TLDC2	-0.311019703	1.84E-17
TLE1	1.160657524	2.48E-102

TLE2	0.126827487	0.468326425
TLE3	1.289972588	3.20E-115
TLE4	0.515255036	6.38E-11
TLE6	1.302899286	1.82E-132
TLK1	0.921796927	8.16E-58
TLK2	0.76020392	2.26E-42
TLL1	-0.674084141	2.62E-10
TLL2	-0.441515676	4.52E-16
TLN1	2.056098981	1.35E-252
TLN2	-0.125514159	0.112734091
TLR10	1.322025265	5.31E-149
TLR2	2.371614956	7.85E-156
TLR4	0.874470685	9.22E-42
TLR5	0.997880349	1.66E-83
TLR6	1.329286689	9.74E-214
TLR9	-1.017647357	5.59E-48
TLX1	1.027612178	1.6653197816342e-313
TLX2	0.204040365	3.41E-15
TLX3	-0.901453295	9.99E-08
TM2D1	1.352094133	4.73E-151
TM2D2	1.989561282	6.37E-202
TM2D3	0.224118766	0.003329944
TM4SF1	1.912421824	8.64E-89
TM4SF18	1.724197999	1.79E-167
TM4SF19	0.115998512	5.24E-34
TM4SF20	0.035044081	0.014549364
TM4SF4	0.013469389	0.005478183
TM4SF5	0.07433252	1.72E-34
TM6SF1	0.817340282	3.12E-25
TM6SF2	0.56227155	2.45E-44
TM7SF2	0.412261511	2.07E-13
TM7SF3	1.915443167	7.74E-236
TM9SF1	-1.376642826	1.85E-153
TM9SF2	1.645994945	2.87E-141
TM9SF3	1.078606059	3.57E-88
TM9SF4	1.572312894	1.27E-95
TMA16	1.462177845	3.72E-156
TMA7	-1.835718521	5.8502313124062e-320
TMBIM1	1.596830148	1.26E-80
TMBIM4	0.205889018	7.61E-05
TMC1	0.375736611	1.75E-79
TMC2	-1.00098587	1.11E-14
TMC3	-0.06172388	3.49E-05
TMC4	0.377861647	1.21E-21
TMC5	0.03218778	0.000500578
TMC6	0.323661586	0.000824926
TMC7	-0.064222936	0.440373411
TMC8	1.283976077	9.06E-90

TMCC1	1.063562221	2.43E-58
TMCC2	-2.044279553	4.65E-148
TMCC3	1.253229735	8.62E-52
TMCO2	0.028757479	0.012241695
TMCO3	1.351328283	2.06E-101
TMCO5A	-0.003590807	0.005604418
TMCO6	0.607460247	1.54E-16
TMED1	0.63344433	2.97E-46
TMED3	1.881804258	3.20E-215
TMED4	2.166928879	3.12E-188
TMED6	0.594434722	3.61E-101
TMED7	2.123608534	1.85E-248
TMED8	0.325179145	5.72E-08
TMED9	1.723413902	1.85E-224
TMEFF2	-0.436993961	5.50E-09
TMEM100	2.632647839	7.04E-216
TMEM102	1.268726531	9.72E-243
TMEM104	1.939941481	8.57E-240
TMEM106A	1.987098931	6.97543765906783e-317
TMEM106B	2.12964933	1.67E-243
TMEM106C	2.207459256	3.03E-165
TMEM107	1.632825241	1.39E-187
TMEM108	1.937248494	2.33E-179
TMEM109	2.185352991	3.93E-215
TMEM114	0.327999213	3.72E-14
TMEM115	2.046786219	6.62E-290
TMEM116	0.884944444	5.17E-68
TMEM117	1.693248067	1.11E-238
TMEM119	3.5667959	1.30E-303
TMEM120A	1.569289276	1.21E-211
TMEM120B	-0.994991635	1.99E-15
TMEM121	1.013689744	4.53E-61
TMEM123	2.484260861	5.69E-237
TMEM125	-0.875273522	7.22E-13
TMEM126A	1.501147773	1.12E-168
TMEM126B	1.244856683	1.39E-105
TMEM127	1.791463128	2.06E-165
TMEM128	2.099260207	2.00E-232
TMEM129	1.320212445	3.62E-138
TMEM130	-2.964789618	2.70E-95
TMEM131	0.865886255	4.31E-34
TMEM132A	0.967310779	8.44E-33
TMEM132B	0.245715351	0.014694697
TMEM132C	-0.909344449	2.56E-61
TMEM132D	-1.157316167	2.47E-40
TMEM132E	1.263978269	6.22E-29
TMEM134	1.038193473	8.98E-131
TMEM135	0.377360646	2.03E-16

TMEM138	1.314446918	1.45E-121
TMEM139	-0.349583364	4.54E-06
TMEM140	2.298060824	1.84E-190
TMEM141	-0.805714839	5.19E-45
TMEM143	0.060416188	0.325724735
TMEM144	-0.219468136	0.079454855
TMEM145	-0.439369156	0.0044307
TMEM147	1.217019376	4.04E-126
TMEM14A	0.93368565	3.46E-34
TMEM14B	1.277190834	1.27E-126
TMEM150A	1.689728925	8.27E-254
TMEM150B	1.163941019	1.25E-288
TMEM150C	0.532372581	3.33E-08
TMEM151A	-1.985184834	1.74E-104
TMEM151B	-2.501226224	1.31E-47
TMEM156	1.77047379	4.83E-221
TMEM158	2.587211025	3.74E-85
TMEM159	1.54372503	1.41E-32
TMEM160	0.238661158	0.003268339
TMEM161A	1.692764199	1.04E-152
TMEM161B	0.972517234	6.23E-52
TMEM163	-0.583020227	8.64E-07
TMEM164	1.867361943	1.26E-211
TMEM165	1.688309644	4.45E-106
TMEM167A	1.685641637	7.79E-187
TMEM167B	1.375858255	1.08E-133
TMEM169	1.597809988	1.84E-120
TMEM17	0.600752347	2.21E-17
TMEM170A	1.497007937	6.09E-214
TMEM170B	0.414538957	7.20E-12
TMEM171	0.134622234	0.002436538
TMEM174	0.099823773	1.17E-160
TMEM175	0.122807498	0.11279505
TMEM176A	1.69805146	1.23E-80
TMEM176B	3.277249844	1.32E-259
TMEM177	1.445042497	3.07E-110
TMEM178A	-0.759161327	1.17E-07
TMEM178B	1.23517894	3.45E-52
TMEM179	-1.387616125	1.81E-51
TMEM179B	1.543535754	4.28E-199
TMEM18	0.624759582	5.12E-28
TMEM181	1.272923802	2.22E-85
TMEM182	1.321221506	1.43E-216
TMEM183A	1.174099192	3.04E-45
TMEM184A	-0.053745126	0.001493474
TMEM184B	-0.166874596	0.010597322
TMEM184C	1.800198317	4.26E-177
TMEM185A	0.956949316	6.28E-67

TMEM185B	1.840087713	2.01E-219
TMEM186	1.226157793	2.28E-116
TMEM19	1.760406407	9.09E-214
TMEM190	-0.272461356	4.02E-06
TMEM191B	-0.76750164	1.80E-38
TMEM191C	-0.588345102	3.12E-51
TMEM192	1.12667931	6.14E-119
TMEM196	-0.370704044	5.66E-05
TMEM198	1.329662911	1.40E-64
TMEM199	0.201843473	0.000292088
TMEM200A	0.683433994	2.03E-47
TMEM200B	-0.274758998	0.00267082
TMEM200C	1.288997098	1.48E-129
TMEM201	1.463571112	1.16E-163
TMEM202	0.017729134	2.02E-08
TMEM203	1.857181016	9.52E-192
TMEM204	2.119555758	2.90E-255
TMEM205	-0.177799774	0.001411708
TMEM207	0.003098973	0.16932911
TMEM208	1.654894166	6.78E-219
TMEM209	2.242367548	1.05E-249
TMEM210	-0.0807701	2.78E-07
TMEM211	-0.163702096	4.22E-13
TMEM212	0.053379395	8.95E-06
TMEM213	0.020739957	0.005792178
TMEM214	1.27570567	1.40E-92
TMEM215	-0.005519574	0.847387296
TMEM216	1.634404011	7.13E-194
TMEM217	0.802018755	4.32E-276
TMEM218	1.757657781	9.83E-252
TMEM219	1.287898326	9.77E-242
TMEM220	1.042427133	9.26E-112
TMEM221	1.049466096	1.37E-51
TMEM222	0.779913231	2.56E-52
TMEM223	1.479074022	1.69E-163
TMEM225	-0.001608647	0.388560333
TMEM229A	-0.430977129	7.64E-11
TMEM229B	1.075420391	1.25E-29
TMEM230	2.155696084	1.76E-271
TMEM231	1.713286197	8.53E-183
TMEM232	0.136850232	0.000785249
TMEM233	0.767345038	2.10E-22
TMEM234	0.361801822	3.30E-12
TMEM235	-2.117957119	1.60E-77
TMEM236	0.261162145	6.72E-106
TMEM237	1.225342947	3.50E-85
TMEM238	1.105877089	8.36E-168
TMEM239	0.007334142	4.60E-06

TMEM240	-0.944001064	1.34E-22
TMEM241	1.275666273	7.66E-116
TMEM242	0.813061325	3.85E-35
TMEM243	0.848836927	2.64E-61
TMEM244	-0.023814151	0.347902378
TMEM245	0.689340355	3.34E-27
TMEM248	2.15589739	2.00E-218
TMEM249	-1.566582995	3.62E-53
TMEM25	0.020491311	0.84517881
TMEM251	0.577942001	1.68E-31
TMEM252	0.135113409	0.013791471
TMEM253	-0.050275325	0.100790591
TMEM254	0.621530984	2.66E-36
TMEM255A	3.539185449	2.86E-239
TMEM255B	1.235030534	3.27E-201
TMEM256	-0.454196298	7.50E-21
TMEM258	1.764868488	5.46E-257
TMEM259	0.247637468	0.001545913
TMEM26	0.76404465	1.69E-177
TMEM260	-0.169007242	0.008477702
TMEM262	0.040792162	0.00020244
TMEM263	2.195725788	3.07E-293
TMEM266	-1.173340977	6.58E-15
TMEM267	1.824098662	1.07E-246
TMEM268	0.389043787	2.34E-06
TMEM269	0.356906637	2.02E-37
TMEM30A	0.988251725	2.50E-51
TMEM30B	-0.023144635	0.559602771
TMEM31	-0.228300167	1.93E-09
TMEM33	1.82106608	2.60E-204
TMEM35A	-0.139547546	0.265556948
TMEM38A	0.50027245	3.33E-08
TMEM38B	1.96163793	5.68E-251
TMEM39A	1.61945069	9.52E-151
TMEM39B	1.760842791	5.84E-262
TMEM40	0.051933341	3.67E-13
TMEM41A	0.40930762	7.19E-20
TMEM41B	1.011489405	1.15E-60
TMEM42	-0.286388391	6.31E-09
TMEM43	1.988477059	6.96E-195
TMEM44	0.883997126	1.22E-45
TMEM45A	2.492879486	2.70E-211
TMEM45B	0.734362534	9.09E-105
TMEM47	1.571708133	6.31E-59
TMEM50A	1.84183653	1.41E-216
TMEM50B	1.040152502	1.15E-51
TMEM51	2.310671734	6.39E-133
TMEM52	-0.745658719	8.63E-26

TMEM52B	1.544939638	1.62E-216
TMEM53	1.135938907	1.24E-124
TMEM54	1.521714969	1.36E-136
TMEM59	1.923487333	2.22E-184
TMEM59L	-0.827552927	7.51E-13
TMEM61	-0.469407951	2.79E-07
TMEM62	0.605243165	2.34E-16
TMEM63A	0.023255138	0.844648377
TMEM63B	0.836314429	1.05E-24
TMEM63C	-1.311214124	1.60E-24
TMEM64	2.010407393	9.56E-228
TMEM65	1.061012819	1.68E-52
TMEM67	1.164430521	2.11E-73
TMEM68	1.703534743	5.01E-308
TMEM70	1.323391377	1.87E-73
TMEM72	-0.128079149	8.52E-07
TMEM74	-0.057953397	0.249471322
TMEM74B	-2.160484247	4.29E-116
TMEM79	1.280064618	1.59E-126
TMEM80	0.511558327	1.38E-17
TMEM81	0.380515192	6.44E-06
TMEM82	0.0230061	7.87E-14
TMEM86B	-1.573238791	1.12E-94
TMEM87A	1.612071884	1.78E-149
TMEM87B	1.299015347	4.50E-125
TMEM88	0.684427964	1.80E-38
TMEM88B	-1.740320211	4.90E-41
TMEM89	0.177425567	9.30E-50
TMEM8B	-0.74978056	2.36E-32
TMEM9	1.915937077	3.03E-236
TMEM91	-0.505637497	6.33E-09
TMEM92	0.258076449	3.03E-79
TMEM94	0.175175397	0.006469362
TMEM95	0.028644475	0.00740029
TMEM97	2.688060839	1.04E-299
TMEM98	1.289593411	3.35E-64
TMEM9B	1.943213681	1.56E-304
TMF1	0.738081234	2.02E-30
TMIE	0.640324035	2.36E-14
TMIGD1	0.014155223	0.00058844
TMIGD2	1.1395809	2.29E-105
TMIGD3	2.936678917	8.85E-164
TMLHE	1.279200499	4.44E-187
TMOD1	1.326629513	2.92E-41
TMOD2	-0.778895601	5.01E-27
TMOD3	1.457526813	1.79E-246
TMPO	2.594275945	5.35E-260
TMPPE	0.369378788	1.97E-54

TMPRSS11A	-0.001568501	0.627553074
TMPRSS11B	-0.047726146	8.62E-06
TMPRSS11D	0.01481925	0.001885992
TMPRSS11E	-0.001207912	0.887939975
TMPRSS11F	0.040449814	9.13E-15
TMPRSS12	-0.000468195	0.860601642
TMPRSS13	0.032984011	0.031914136
TMPRSS15	0.089032798	7.49E-81
TMPRSS2	0.306969756	7.51E-77
TMPRSS3	-0.322358505	5.44E-07
TMPRSS4	-0.038480332	0.001086751
TMPRSS5	0.831125917	5.06E-28
TMPRSS6	-0.248814346	0.001038726
TMPRSS7	0.535846656	5.38E-193
TMPRSS9	0.817550023	3.67E-148
TMSB10	3.039357742	6.73E-235
TMSB15B	0.34883748	6.79E-59
TMSB4X	2.691365608	2.03E-259
TMSB4Y	0.056411568	0.544858842
TMTC1	-0.261004577	0.003390552
TMTC2	0.744588626	1.51E-24
TMTC3	1.315126631	8.60E-104
TMTC4	0.593113643	1.05E-14
TMUB1	1.810724336	9.91E-268
TMUB2	1.306211303	8.53E-76
TMX2	1.612596705	4.02E-160
TMX3	1.302963857	2.41E-69
TMX4	1.531163077	9.64E-80
TNFAIP1	1.577208076	5.57E-161
TNFAIP2	1.617348677	1.81E-90
TNFAIP3	2.455964127	1.67E-241
TNFAIP8L1	1.011565156	2.60E-34
TNFAIP8L3	1.568714743	5.26E-54
TNFRSF10A	0.624017944	2.72E-45
TNFRSF10B	2.436598598	1.13E-252
TNFRSF10C	1.310689221	4.42E-255
TNFRSF10D	1.357337746	4.99E-87
TNFRSF11A	0.974622571	1.26E-96
TNFRSF11B	2.485096868	4.07E-242
TNFRSF13B	-0.044126438	1.89E-08
TNFRSF13C	0.580047704	2.03E-27
TNFRSF14	0.400907618	1.71E-10
TNFRSF17	0.114023903	7.10E-09
TNFRSF18	0.446323717	3.35E-07
TNFRSF1B	2.477096163	9.09E-165
TNFRSF21	2.381613569	5.10E-134
TNFRSF25	-2.292802306	1.39E-38
TNFRSF4	1.382029635	9.65E-208

TNFRSF8	0.34412194	8.33E-07
TNFRSF9	0.372863374	1.72E-185
TNFSF10	1.306724748	5.73E-57
TNFSF11	0.139629691	1.69E-52
TNFSF12	0.179834331	0.000162874
TNFSF13	-1.294892127	2.12E-84
TNFSF14	1.010907397	6.79E-199
TNFSF15	0.339403813	6.91E-252
TNFSF18	1.180127186	1.21E-286
TNFSF4	0.854945299	2.89E-70
TNFSF9	-0.437865158	4.89E-10
TNIK	0.179153899	0.00193084
TNIP1	1.640411893	3.91E-174
TNIP2	2.240200084	1.14E-271
TNIP3	0.033222967	0.014567777
TNK1	-0.919509656	2.63E-13
TNK2	-1.395504687	1.31E-86
TNKS	1.016465936	3.63E-47
TNKS1BP1	1.701376582	4.46E-207
TNKS2	0.835397615	8.61E-33
TNMD	0.754803093	2.95E-125
TNNC1	-0.69314939	7.57E-19
TNNC2	-0.717917087	1.35E-26
TNNI1	-0.072069178	0.000678297
TNNI2	1.056428634	5.61E-93
TNNI3	-0.800944713	3.10E-33
TNNI3K	-2.027631985	1.23E-123
TNNT1	-0.931799311	1.02E-27
TNNT2	-1.370496283	2.46E-25
TNNT3	-0.301163343	2.53E-11
TNP1	-0.235751452	1.56E-10
TNP2	-0.033736477	1.08E-05
TNPO1	2.219284412	4.53E-275
TNPO2	0.860776855	1.58E-35
TNPO3	1.966921989	3.53E-161
TNR	-0.321392422	1.33E-05
TNRC18	1.23706302	2.82E-47
TNRC6A	-0.104949993	0.224240174
TNRC6B	0.278687668	2.77E-05
TNRC6C	-0.312172625	1.36E-05
TNS1	0.750169342	4.40E-25
TNS2	-0.609370663	2.45E-34
TNS3	0.902998845	8.20E-61
TNS4	-0.055763023	1.26E-05
TNXB	0.121035681	0.248076485
TOB1	1.078494282	6.78E-56
TOB2	1.239678097	2.51E-45
TOE1	1.637494995	1.28E-145

TOGARAM1	-0.212240945	0.002738782
TOGARAM2	-0.25909882	6.82E-06
TOLLIP	0.172475043	0.038864906
TOM1	0.206870485	0.00092504
TOM1L1	0.934545771	9.98E-80
TOM1L2	-0.652700056	1.17E-26
TOMM20	1.234568213	1.74E-56
TOMM20L	0.795590713	2.84E-275
TOMM22	2.106185786	1.40E-245
TOMM34	1.266425374	2.50E-36
TOMM40	1.69377387	3.66E-137
TOMM40L	0.856951276	1.02E-33
TOMM7	0.836722966	1.17E-61
TOMM70	0.763729257	8.17E-18
TONSL	1.261789317	3.65E-54
TOP1	1.762102665	4.38E-132
TOP1MT	1.702703657	4.22E-278
TOP2B	1.062519135	4.10E-48
TOP3A	1.235024022	3.86E-67
TOP3B	-0.918232825	2.28E-52
TOPAZ1	-0.017394704	6.24E-05
TOPBP1	1.573989108	4.57E-111
TOPORS	1.12996249	6.46E-72
TOR1A	2.043726157	1.95E-205
TOR1AIP2	1.446382248	5.26E-223
TOR1B	2.039086064	3.59E-163
TOR2A	0.681127076	2.05E-18
TOX	1.354443929	5.75E-56
TOX2	0.042509846	0.689267812
TOX3	1.010198334	1.41E-42
TOX4	0.69418947	1.85E-27
TP53AIP1	0.062102164	0.154577167
TP53BP1	-0.025181371	0.76041125
TP53BP2	0.888861443	3.02E-42
TP53I11	1.140859853	1.19E-18
TP53I13	1.537124082	4.00E-160
TP53INP1	2.320858868	1.48E-300
TP53INP2	-0.505877652	3.24E-06
TP53TG3	-0.001023152	0.535082839
TP53TG3B	-0.000268654	0.621957229
TP53TG3C	-0.000197638	0.713289972
TP53TG3D	-0.195074351	2.22E-10
TP53TG3E	0	1
TP53TG3F	-0.000324785	0.55835614
TP53TG5	-0.432130197	5.19E-11
TP63	0.335034117	1.15E-58
TP73	0.996529105	4.10E-22
TPBG	1.009415361	4.52E-19

TPBGL	-0.453303515	3.23E-07
TPCN1	-0.218627757	0.023564322
TPCN2	-0.388808055	2.89E-06
TPD52	-0.190425748	0.018997384
TPD52L1	-1.782995186	1.11E-47
TPD52L2	0.480960554	9.15E-15
TPD52L3	0.007958709	0.001361606
TPGS1	-1.446547593	3.35E-103
TPGS2	1.252218541	5.72E-93
TPH1	0.443053253	1.19E-65
TPH2	-0.032297078	0.000332175
TPI1	1.923835618	2.14E-132
TPK1	0.385276732	7.77E-25
TPM1	0.176993424	0.011388482
TPM2	1.270836541	5.80E-50
TPM3	0.792612669	2.54E-40
TPM4	0.848883497	1.30E-22
TPMT	1.149452026	3.93E-71
TPO	-0.163481411	2.37E-11
TPP1	1.230481174	7.97E-93
TPP2	0.724018149	2.46E-23
TPPP	-2.765842009	7.65E-217
TPPP2	0.037479411	0.006656356
TPPP3	0.765271227	3.25E-13
TPR	0.998814684	1.17E-50
TPRA1	1.13596076	2.97E-74
TPRG1L	-0.288204505	0.000381403
TPRKB	1.450750153	2.35E-110
TPRN	-0.826403612	2.40E-17
TPRX1	-0.002177497	0.683252374
TPSAB1	0.547016364	3.67E-96
TPSB2	0.323704743	3.39E-21
TPSD1	-0.352070033	6.13E-17
TPSG1	0.044862182	0.144062093
TPST1	3.180118464	7.31380197508172e-319
TPST2	1.629755645	1.26E-210
TPT1	1.189416964	3.84E-100
TPTE	-0.009904898	0.000288273
TPTE2	-0.041779597	0.203024735
TRA2A	0.617101944	2.07E-21
TRA2B	0.270221465	1.19E-05
TRABD	0.508764748	5.34E-09
TRABD2A	-0.03840197	0.367106122
TRABD2B	0.078488553	0.089127931
TRADD	1.837266679	1.19E-242
TRAF1	0.966670045	1.15E-65
TRAF2	0.869246234	1.66E-37
TRAF3	0.338690716	9.84E-08

TRAF3IP1	0.325947507	2.51E-12
TRAF3IP2	1.841464242	4.61E-264
TRAF3IP3	0.635867828	3.91E-48
TRAF5	1.131302341	1.60E-100
TRAF6	1.500438346	1.60E-149
TRAF7	1.598214811	3.07E-97
TRAFD1	2.191729471	5.64E-228
TRAIP	1.705131105	1.39E-147
TRAK1	0.700805194	9.23E-23
TRAK2	-0.031763426	0.711559678
TRAM1L1	0.04126308	0.522210364
TRAM2	2.04599145	1.72E-306
TRANK1	-0.830840491	8.33E-09
TRAP1	0.703390197	3.72E-21
TRAPPC1	0.990374573	1.78E-71
TRAPPC10	0.303574446	4.16E-06
TRAPPC11	0.925738131	5.33E-36
TRAPPC12	0.634142207	5.67E-28
TRAPPC13	0.122333324	0.063152051
TRAPPC2	-0.215121657	0.002623423
TRAPPC2B	1.068633067	4.54E-94
TRAPPC2L	1.038988674	1.77E-75
TRAPPC3	1.929602369	3.14E-183
TRAPPC3L	-0.005646862	0.634127719
TRAPPC4	0.470370502	2.51E-15
TRAPPC5	-2.355308377	3.47493661168265e-310
TRAPPC6A	1.125960692	8.14E-103
TRAPPC6B	-2.816825178	1.52E-190
TRAPPC8	0.791479747	1.23E-28
TRAPPC9	0.78267881	1.76E-34
TRAT1	0.318898504	8.00E-294
TRDMT1	0.211231012	0.000106246
TRDN	-0.33925688	1.28E-09
TREH	0.167971547	1.45E-21
TREML1	1.734379659	4.06E-170
TREML2	0.340770058	3.15E-212
TREML4	0.235655419	6.05E-138
TRERF1	0.516330753	1.33E-13
TREX2	0.040129842	0.063814377
TRH	1.730626756	4.04E-53
TRHDE	-1.154694927	1.26E-35
TRHR	0.054006152	2.50E-11
TRIB1	1.765836494	1.53E-169
TRIB3	3.012242571	1.40E-258
TRIL	1.050799088	1.37E-34
TRIM10	-0.000871017	0.824286741
TRIM11	-0.075931382	0.49794549
TRIM13	0.81332953	2.20E-40

TRIM15	0.005030889	0.127454904
TRIM16	0.704225407	5.96E-45
TRIM17	-2.130622057	1.07E-54
TRIM2	-0.781222671	1.94E-29
TRIM22	2.592832926	5.86E-194
TRIM23	0.735419526	1.47E-23
TRIM25	1.116470182	1.32E-54
TRIM26	1.297085504	1.31E-79
TRIM27	1.134467765	3.13E-66
TRIM28	2.018000513	7.34E-140
TRIM29	0.126162911	0.0007419
TRIM3	-0.642052185	7.94E-08
TRIM31	0.020155449	0.02025305
TRIM32	1.55023802	8.32E-61
TRIM33	0.80845677	2.17E-33
TRIM34	-0.863233384	4.22E-91
TRIM35	1.527647017	4.83E-144
TRIM36	1.299085096	2.15E-32
TRIM37	0.42513326	1.86E-05
TRIM38	1.736966747	1.46E-305
TRIM39	0.346006916	1.33E-09
TRIM4	2.343073306	6.99E-294
TRIM40	0.002102566	0.591317105
TRIM41	-1.500045743	9.56E-141
TRIM42	0.000537684	0.867505444
TRIM43	-0.001604449	0.863702324
TRIM43B	0.016779785	0.02304527
TRIM44	0.169952929	0.024692431
TRIM45	1.289568425	2.12E-148
TRIM46	-0.302483903	0.002375275
TRIM47	1.968187294	4.21E-181
TRIM48	0.303854668	1.51E-64
TRIM49	0.001378481	0.843090892
TRIM49B	-0.002769515	0.76130278
TRIM49C	-0.000567761	0.941096586
TRIM49D1	8.69E-05	0.966833371
TRIM49D2	0.002357174	0.000828949
TRIM50	0.079952394	5.97E-15
TRIM51	-0.003984583	0.205367818
TRIM52	-1.041029119	3.91E-33
TRIM54	-0.371678609	0.000331321
TRIM55	-0.316530982	9.09E-08
TRIM56	1.866126053	5.45E-183
TRIM58	0.18073188	9.81E-06
TRIM59	0.298913721	5.96E-05
TRIM60	0.003712822	0.004295578
TRIM61	0.864741775	3.61E-148
TRIM62	0.705433635	7.15E-21

TRIM63	0.130541739	1.77E-05
TRIM64	0.001104607	0.159585628
TRIM64B	0.035436876	1.10E-21
TRIM64C	0.000234458	0.861671341
TRIM65	1.30294109	7.92E-69
TRIM66	-0.1788632	0.025533895
TRIM67	-0.553555233	9.72E-08
TRIM68	0.91834943	6.75E-48
TRIM69	1.040605987	1.33E-85
TRIM7	-0.920063238	7.47E-12
TRIM71	0.274296041	6.42E-19
TRIM72	-0.813993849	1.45E-14
TRIM73	-0.500962234	3.48E-20
TRIM74	-0.023987323	0.665238937
TRIM77	-0.002330633	0.207190376
TRIM8	-0.415391314	3.08E-18
TRIM9	1.14888031	3.71E-28
TRIML1	0.009819359	0.00130083
TRIML2	-0.098483572	1.04E-06
TRIO	1.461503687	9.37E-54
TRIP10	1.701426306	4.48E-169
TRIP11	0.977478985	1.98E-68
TRIP12	1.193393414	1.59E-94
TRIP4	1.351104333	9.30E-144
TRIQK	0.259383773	7.89E-05
TRIT1	-0.148939142	0.042317024
TRMO	1.693296952	2.25979005928132e-316
TRMT1	0.921333698	2.83E-45
TRMT10A	1.055246811	4.77E-135
TRMT10B	0.315943327	2.78E-08
TRMT10C	2.365794454	2.69E-244
TRMT11	-0.442988201	1.19E-08
TRMT112	2.163158911	2.69E-254
TRMT12	1.928830527	2.06E-209
TRMT13	0.901446921	2.08E-41
TRMT1L	0.868353016	1.21E-49
TRMT2A	0.677891718	1.27E-23
TRMT44	0.496322468	2.32E-14
TRMT5	1.674397247	1.05E-212
TRMT6	1.515271668	1.60E-92
TRMT61A	0.89686725	5.13E-60
TRMT61B	1.460477424	2.16E-85
TRMU	0.31932784	8.74E-08
TRNAU1AP	1.772099053	1.60E-172
TRNP1	-0.456580765	6.85E-06
TRNT1	0.987811046	9.66E-88
TRO	0.737241082	5.33E-20
TRPA1	0.03309198	7.38E-05

TRPC1	0.016288576	0.831503782
TRPC3	-0.660489076	1.14E-24
TRPC4	0.599778022	7.99E-59
TRPC4AP	1.712502116	1.94E-126
TRPC5	-0.13932849	1.35E-07
TRPC5OS	0.546108691	1.29E-285
TRPC6	0.95013902	5.31E-128
TRPC7	0.316658303	1.08E-37
TRPM1	-0.157483457	1.56E-08
TRPM2	-0.179589486	0.042186127
TRPM3	-1.247404185	1.75E-42
TRPM4	0.705294801	1.92E-36
TRPM5	0.017063983	0.007902883
TRPM6	-0.479191592	2.80E-22
TRPM7	0.770438538	2.95E-22
TRPS1	1.668925564	9.59E-233
TRPT1	0.612251087	9.10E-49
TRPV1	-0.67415427	4.56E-19
TRPV2	1.61447688	1.46E-108
TRPV3	-0.82393903	1.27E-58
TRPV5	-0.280022479	1.38E-15
TRPV6	-1.381465418	4.36E-65
TRRAP	0.871025751	3.92E-29
TRUB1	0.9925991	3.22E-45
TRUB2	1.260306146	2.40E-131
TSACC	0.723555948	1.63E-143
TSC1	-0.96036543	3.73E-29
TSC2	-0.57290356	7.79E-09
TSC22D1	0.68697132	1.05E-25
TSC22D2	0.77912114	1.48E-34
TSC22D3	1.224965189	1.57E-56
TSC22D4	1.334847021	1.50E-91
TSEN15	0.134199005	0.024520882
TSEN2	0.695435857	3.25E-33
TSEN54	0.65170757	3.04E-12
TSFM	-2.396315021	1.76E-278
TSG101	1.649276096	6.97E-182
TSGA10	-0.071720422	0.111274376
TSGA10IP	-0.10523531	0.03732697
TSGA13	0.022644331	1.11E-20
TSHB	-0.066891553	0.008972975
TSHR	0.532483934	5.92E-59
TSHZ1	0.235571444	0.001661032
TSHZ2	0.817038577	8.86E-52
TSHZ3	1.614652905	4.16E-138
TSKS	0.031367844	0.101036002
TSLP	0.579588322	3.32E-160
TSN	1.817564764	3.92E-215

TSNARE1	0.157382171	0.012014048
TSNAX	0.034915766	0.626180788
TSNAXIP1	-0.003809565	0.960639272
TSPAN1	0.203976346	0.038825221
TSPAN10	0.998093693	1.45E-266
TSPAN11	2.293506784	2.83E-163
TSPAN13	1.729281926	2.14E-57
TSPAN14	1.611196021	1.21E-177
TSPAN15	0.442323334	1.24E-05
TSPAN16	0.01335602	0.352644883
TSPAN17	1.116362122	1.87E-48
TSPAN18	1.18567031	4.38E-13
TSPAN19	-0.673256975	4.47E-54
TSPAN2	0.806438882	4.30E-42
TSPAN3	1.845732394	1.07E-216
TSPAN31	2.143310323	1.88E-215
TSPAN32	0.827659028	1.16E-236
TSPAN33	1.130791027	8.73E-78
TSPAN4	1.198086735	2.61E-54
TSPAN5	0.163356518	0.084176498
TSPAN7	-0.699678913	2.18E-14
TSPAN8	-0.435720858	5.43E-05
TSPAN9	0.828413302	1.50E-07
TSPEAR	-0.078229673	5.62E-05
TSPO	2.926303421	2.03E-296
TSPO2	0.106043049	4.78E-17
TSPOAP1	-2.393830891	5.92E-114
TSPY1	-0.000343286	0.602118838
TSPY10	0.000426234	0.474585743
TSPY2	0.006650085	0.527693715
TSPY3	-4.98E-05	0.925900253
TSPY4	-2.64E-05	0.961694594
TSPY8	0.00101825	0.047833226
TSPYL1	-1.679302879	2.23E-59
TSPYL2	-2.358918586	2.50E-96
TSPYL4	-0.063456096	0.522588396
TSPYL5	-0.866648449	1.45E-30
TSPYL6	-0.09790773	7.43E-17
TSR1	1.582184462	1.19E-157
TSR2	-1.105264688	1.40E-95
TSR3	1.06469184	1.35E-103
TSSC4	1.70829954	5.42E-231
TSSK1B	-0.002331463	0.448616518
TSSK3	-1.666433609	4.33E-58
TSSK4	0.513794146	1.43E-16
TSSK6	1.483407384	2.99E-275
TST	1.651157152	2.23E-172
TSTD1	0.292974053	0.004366185

TSTD2	0.997812397	3.94E-32
TSTD3	-0.624203474	1.47E-51
TTBK1	-0.508693429	1.31E-10
TTBK2	-0.868211218	1.10E-35
TTC1	1.080348268	2.15E-76
TTC12	0.559132473	9.84E-23
TTC13	1.011222744	8.50E-52
TTC14	0.032391138	0.639598188
TTC16	-0.269946764	7.78E-15
TTC17	0.522539462	8.56E-13
TTC19	0.499356464	6.78E-11
TTC21A	-0.086601341	0.126874747
TTC21B	0.406638634	1.78E-08
TTC22	-0.543387742	1.78E-23
TTC23L	0.371546898	3.99E-29
TTC24	0.478777756	7.45E-164
TTC28	1.429187246	3.90E-228
TTC29	0.361542891	6.45E-14
TTC3	0.007462268	0.929290015
TTC31	1.040587638	7.69E-44
TTC32	0.685589919	6.24E-22
TTC33	1.079140689	8.54E-70
TTC34	0.101095301	0.011573225
TTC36	-0.169612426	0.000821137
TTC37	0.355940297	7.83E-05
TTC38	1.980076088	3.70E-259
TTC39A	0.344871165	3.51E-11
TTC39B	0.098052784	0.270706174
TTC39C	1.258449732	1.44E-108
TTC5	0.857932341	3.35E-73
TTC6	-0.058702036	0.012413616
TTC7A	1.270788175	5.02E-94
TTC7B	-0.637749983	3.86E-15
TTC8	0.973943137	8.33E-57
TTC9	0.366198596	5.72E-05
TTC9B	-2.687907144	1.30E-91
TTC9C	1.606697567	8.17E-123
TTF1	1.160681228	1.10E-86
TTF2	1.58973828	1.71E-271
TTI1	1.924065827	2.19E-220
TTI2	1.74994543	1.69E-230
TTL	1.147693175	3.14E-124
TTLL1	0.993050828	1.15E-54
TTLL10	-0.135556752	7.29E-06
TTLL11	-0.118140908	0.088070054
TTLL12	0.517665977	1.41E-10
TTLL13P	-0.710345923	2.07E-111
TTLL2	0.085984255	3.22E-10

TTLL3	-1.109364895	8.34E-65
TTLL4	1.012773532	3.00E-73
TTLL5	0.531758364	8.89E-21
TTLL6	0.104104159	0.001639125
TTLL7	-0.795643371	3.32E-23
TTLL8	-0.00414243	0.216995001
TTLL9	0.698347457	5.32E-35
TTN	-0.105760826	1.51E-11
TTPA	-0.090924071	0.08932574
TTPAL	1.457005493	1.19E-124
TTR	-2.016640631	2.95E-26
TTYH1	0.772006719	6.19E-29
TTYH2	1.050148951	1.15E-26
TTYH3	2.594077801	1.48E-146
TUB	-0.44851554	6.07E-09
TUBA1A	2.858886083	5.33E-210
TUBA1B	0.27822716	0.00786297
TUBA3C	0.011883995	0.212104673
TUBA3D	0.12684811	0.002242634
TUBA3E	0.048445949	0.016098127
TUBA4A	-1.388072756	1.84E-30
TUBA4B	-0.259530808	0.000627741
TUBA8	-1.177451057	3.16E-36
TUBAL3	0.006171212	0.758173203
TUBB	2.558879834	2.27E-205
TUBB1	0.268405256	1.42E-22
TUBB2A	0.722995709	4.06E-13
TUBB2B	1.984606264	3.48E-133
TUBB3	-4.461657785	4.86E-287
TUBB4A	-2.082769131	5.92E-63
TUBB4B	1.313789372	6.02E-63
TUBB6	2.899455511	6.67E-286
TUBB8	-0.048670696	0.000662299
TUBD1	1.17421758	1.27E-96
TUBE1	-0.874112341	2.24E-19
TUBG1	1.45711393	1.48E-77
TUBG2	-0.727044255	6.17E-28
TUBGCP2	0.456281528	7.92E-11
TUBGCP3	1.224072701	1.99E-84
TUBGCP4	-0.487210141	2.06E-09
TUBGCP5	1.047022326	2.20E-58
TUBGCP6	-0.316179021	0.001778925
TUFM	1.919262428	3.08E-248
TUFT1	0.588151764	2.67E-10
TUG1	-0.042800001	0.536388605
TULP1	0.204602098	8.19E-17
TULP2	0.270402933	9.11E-26
TULP3	1.593957253	8.11E-79

TULP4	-0.368776855	1.86E-11
TUNAR	-2.696602697	1.34E-91
TUSC1	0.668321274	1.59E-28
TUSC2	0.745228717	4.93E-33
TUSC3	-0.320559494	0.003876235
TUT1	0.228772058	0.000103938
TVP23A	0.631160787	2.17E-31
TVP23B	1.042341396	1.02E-98
TVP23C	-0.518287106	8.02E-35
TWF2	-0.183173091	0.029456081
TWIST2	0.875535528	9.75E-117
TWNK	1.40980892	1.68E-165
TXK	0.133134601	1.52E-17
TXLNA	1.795405326	3.34E-140
TXLNG	0.849043958	3.17E-30
TXN	1.818289183	2.55E-201
TXNDC11	1.079326489	2.91E-44
TXNDC12	-0.930757524	1.86E-69
TXNDC15	1.725194544	5.09E-134
TXNDC16	1.0587862	3.08E-48
TXNDC17	1.543532196	4.20E-196
TXNDC2	0.437484576	3.13E-249
TXNDC8	0.008336281	3.04E-09
TXNDC9	1.656719542	2.72E-175
TXNIP	2.092240787	4.50E-133
TXNL1	1.293642053	9.99E-128
TXNL4A	1.282503921	6.99E-125
TXNL4B	1.332974084	8.97E-112
TXNRD1	1.989863397	2.27E-196
TXNRD2	0.375138284	5.02E-06
TYK2	1.330172078	8.32E-71
TYMP	1.734456825	2.03E-46
TYMS	2.307549269	3.66E-212
TYR	0.014365203	0.017736374
TYRO3	0.159248322	0.065050914
TYROBP	4.309720238	4.66E-251
TYRP1	-0.119519591	0.005177929
TYSND1	0.745856924	4.45E-23
TYW1	1.761178272	2.28E-207
TYW1B	1.002214361	6.34E-94
TYW3	0.497967757	1.04E-18
TYW5	-0.079762549	0.139005778
U2AF1	0.190361402	2.74E-15
U2AF1L4	-1.651576733	1.39E-133
U2AF1L5	0.163588207	3.14E-15
U2AF2	1.660771898	6.74E-104
U2SURP	0.370467731	5.60E-07
UACA	1.056267304	3.73E-66

UAP1	1.386237859	7.26E-80
UAP1L1	1.068912062	2.49E-59
UBA1	1.869224167	2.02E-129
UBA2	1.847502311	3.45E-166
UBA3	0.775706788	2.51E-36
UBA5	0.396873766	1.18E-10
UBA52	1.786398175	5.08E-284
UBA6	1.109456386	1.21E-66
UBA7	1.792457111	8.44E-171
UBAC1	1.121300691	4.34E-120
UBAC2	1.960613705	1.38338380835549e-322
UBALD1	0.278814259	0.000347162
UBAP1	1.13292039	3.54E-78
UBAP1L	-1.684090031	4.88E-75
UBAP2	0.031902092	0.617551933
UBAP2L	1.752888182	1.47E-145
UBASH3A	0.239123189	2.11E-83
UBASH3B	0.731066143	4.21E-08
UBB	0.970102468	2.46E-57
UBC	1.892827111	1.09E-135
UBD	-0.016148968	0.462480893
UBE2A	1.1557908	8.44E-97
UBE2B	1.033193258	1.18E-62
UBE2D1	0.790948398	8.67E-44
UBE2D2	1.626084697	1.40E-177
UBE2D3	0.962542032	5.67E-57
UBE2D4	1.156293517	3.11E-119
UBE2E1	0.411804348	6.35E-16
UBE2E2	1.004183164	1.28E-43
UBE2E3	1.565210314	1.64E-132
UBE2F	-0.419073319	2.43E-14
UBE2G1	1.597225351	1.57E-160
UBE2G2	0.450493565	1.27E-12
UBE2H	1.537037939	1.06E-145
UBE2I	-0.085057692	0.225682822
UBE2J1	1.845887804	7.41E-186
UBE2J2	1.462792145	8.43E-137
UBE2K	1.123570579	7.43E-49
UBE2L3	0.75170343	2.25E-49
UBE2M	0.845697688	8.21E-46
UBE2N	1.218089338	1.87E-68
UBE2O	0.453732477	1.16E-06
UBE2Q1	0.891693007	6.15E-43
UBE2Q2	1.738829813	1.22E-192
UBE2QL1	-1.576433613	7.71E-26
UBE2R2	1.420325516	1.11E-150
UBE2S	2.248464734	1.54E-226
UBE2T	2.148066416	7.62E-129

UBE2U	-0.016818656	0.082746025
UBE2V2	0.867815809	6.34E-36
UBE2W	0.878097485	1.08E-47
UBE2Z	1.120699966	6.60E-65
UBE3A	0.457440984	4.96E-14
UBE3B	0.586057766	1.69E-22
UBE3C	1.031753707	4.56E-44
UBE3D	0.491891012	4.99E-40
UBE4A	0.9074234	4.58E-21
UBE4B	0.269348561	0.000443408
UBFD1	0.92540648	1.99E-29
UBIAD1	2.06080328	3.33E-302
UBL3	1.130820275	2.56E-69
UBL4A	1.547181538	4.18E-128
UBL4B	1.016534838	8.24E-213
UBL5	0.241337535	8.16E-06
UBL7	1.213201208	3.91E-79
UBLCP1	1.539171099	1.51E-111
UBN1	1.007545659	2.84E-39
UBN2	0.083110149	0.296005833
UBOX5	0.856652159	6.48E-51
UBP1	0.048371956	0.577250268
UBQLN1	0.606087817	5.32E-15
UBQLN2	0.793053506	4.45E-25
UBQLN3	-0.012046816	0.010930903
UBQLN4	0.975381819	3.80E-55
UBQLNL	0.620914913	7.85E-181
UBR1	0.849409687	9.40E-35
UBR2	0.628155964	2.11E-18
UBR3	0.156514918	0.03076556
UBR4	0.303295502	9.63E-05
UBR5	0.451469612	5.74E-11
UBR7	1.81380138	7.98E-185
UBTD1	1.74510445	9.17E-286
UBTD2	2.06556421	1.44E-96
UBTF	1.16204665	4.50E-60
UBTFL1	0.001372131	0.54349323
UBXN1	0.473497877	2.38E-19
UBXN10	-0.060942099	0.376036743
UBXN11	0.70338753	3.66E-30
UBXN2B	1.43054168	4.87E-86
UBXN4	1.604313998	6.09E-125
UBXN6	-0.516646475	1.05E-15
UBXN7	0.672028593	7.83E-22
UBXN8	1.342253512	3.50E-155
UCHL1	-0.188626271	0.100853684
UCHL3	-1.787661984	2.04E-210
UCHL5	0.625773128	4.42E-20

UCK1	1.230750477	8.07E-101
UCK2	1.647900142	1.02E-133
UCKL1	0.941254166	3.48E-19
UCMA	-0.074641937	0.021685556
UCN	0.225125041	1.07E-05
UCN2	0.476451507	3.14E-44
UCN3	-0.09894047	0.000208875
UCP1	0.112617779	4.97E-22
UCP3	0.327762928	2.74E-07
UEVLD	1.790184649	3.68E-251
UFC1	1.380096671	1.39E-147
UFL1	1.197603872	1.12E-90
UFM1	0.937913092	9.65E-66
UFSP1	1.900314584	2.69496060981006e-317
UFSP2	1.074687828	5.89E-88
UGCG	2.15659148	2.15E-159
UGGT1	1.803713987	1.41E-186
UGGT2	1.135843412	9.75E-92
UGP2	-0.207270855	0.005187343
UGT1A1	0.002233676	0.263083469
UGT1A10	-0.001524221	0.605814036
UGT1A3	-0.00092806	0.459665324
UGT1A4	-0.00361149	0.130778411
UGT1A5	-0.000948813	0.568981593
UGT1A6	-0.000268814	0.825630464
UGT1A7	-0.000673894	0.600326007
UGT1A8	-0.001521865	0.577869888
UGT1A9	-0.002343401	0.305573692
UGT2A1	-0.003852711	0.038331593
UGT2A2	-0.000400644	0.843145058
UGT2A3	-0.005250809	0.011173098
UGT2B10	-0.006041392	0.038883939
UGT2B11	0.004708295	0.023982815
UGT2B15	0.016315543	6.52E-06
UGT2B17	0.182028056	1.37E-35
UGT2B28	0.008336961	1.86E-07
UGT2B4	0.00978821	0.081436749
UGT2B7	0.128998632	4.95E-31
UGT3A1	-0.002149344	0.142255637
UGT3A2	0.048583385	0.005226634
UGT8	0.121393338	0.390042421
UHMK1	0.795685884	4.60E-35
UHRF1BP1	1.13326252	1.78E-57
UHRF1BP1L	0.704566953	1.33E-29
UHRF2	0.706949417	2.87E-16
UIMC1	0.995406285	5.32E-56
ULBP1	0.677450581	4.42E-13
ULBP2	1.604669479	1.55E-96

ULK1	-0.664206566	3.72E-11
ULK2	-0.447352219	6.54E-12
ULK3	0.048718534	0.67270161
ULK4	1.191120509	1.60E-259
UMAD1	1.338604328	1.76E-110
UMOD	-0.006264322	0.309625862
UMODL1	0.093649293	7.03E-08
UNC119	0.713814018	9.84E-24
UNC119B	1.448322138	2.91E-134
UNC13A	-1.698984833	2.85E-34
UNC13B	0.543277987	1.53E-15
UNC13C	-2.672489582	2.22E-92
UNC13D	0.527299315	1.21E-11
UNC45A	0.858604315	1.16E-25
UNC45B	0.021498901	0.001023478
UNC50	2.041624592	4.41E-252
UNC5A	-1.761752494	9.45E-32
UNC5B	1.163571101	2.54E-31
UNC5C	-0.463760926	1.12E-14
UNC5CL	0.2167794	2.35E-05
UNC5D	-0.239510212	0.004190214
UNC79	-1.208446454	2.26E-34
UNC80	-1.446535388	4.41E-33
UNC93A	-0.004638869	0.104990811
UNC93B1	2.472358664	1.05E-298
UNCX	-0.899688929	8.08E-08
UNG	2.039498178	8.40E-294
UNK	1.537610029	1.43E-104
UNKL	0.035883071	0.700601902
UPB1	-0.219122367	6.30E-30
UPF1	0.776161072	9.18E-22
UPF2	0.008655568	0.88664149
UPF3A	-0.411843969	3.99E-08
UPF3B	0.579170578	5.31E-17
UPK1A	-0.025935052	0.447455522
UPK1B	-0.257722397	1.55E-07
UPK2	0.382793172	3.31E-22
UPK3A	0.883314496	2.66E-174
UPK3B	-0.053078589	0.027496965
UPP1	0.951238516	3.08E-25
UPP2	-0.467307357	4.43E-25
UPRT	1.457797728	1.25E-154
UQCC1	0.973584174	4.02E-44
UQCC2	0.242296949	3.27E-06
UQCC3	0.802393588	4.52E-57
UQCR10	0.250155814	2.04E-06
UQCR11	-0.624425283	4.35E-40
UQCRB	-0.800550609	5.81E-54

UQCRC1	1.256937982	1.02E-73
UQCRC2	0.772052522	1.06E-25
UQCRFS1	1.628340071	3.63E-146
UQCRH	0.502248474	3.04E-12
UQCRHL	1.601852828	2.27E-131
UQCRQ	0.178324505	0.000897545
URAD	-0.21237429	5.38E-09
URB1	1.520081216	2.95E-135
URGCP	0.976617847	3.57E-36
URI1	1.376328495	2.56E-167
URM1	1.848895611	3.49E-249
UROC1	0.13331276	7.52E-19
UROD	1.48813618	2.35E-142
UROS	-0.333900975	1.42E-07
USB1	1.122168486	1.39E-97
USE1	1.314576465	1.39E-153
USF1	1.521720666	2.83E-101
USF2	0.029477359	0.551783854
USF3	0.319357734	1.24E-07
USH1C	-0.243142003	0.03485734
USH1G	-0.129838509	0.000982705
USH2A	-0.003205566	0.725099509
USHBP1	-0.169859577	0.000144512
USO1	1.275743751	3.01E-81
USP1	2.247377642	1.58E-221
USP10	1.676099153	2.37E-126
USP11	0.452755749	1.31E-07
USP12	0.016714238	0.758961959
USP13	1.275067275	1.06E-101
USP14	1.045780566	1.23E-50
USP15	0.979441299	1.76E-52
USP16	0.239812599	0.000141453
USP17L1	-0.032057835	5.90E-06
USP17L10	0.00017503	0.803189059
USP17L11	0	1
USP17L12	0	1
USP17L13	-1.33E-05	0.981134128
USP17L15	0.000303494	0.555279757
USP17L17	0	1
USP17L18	0	1
USP17L19	0	1
USP17L2	-0.029033707	0.009246821
USP17L20	0	1
USP17L21	0	1
USP17L22	-0.000125757	0.822251212
USP17L24	0	1
USP17L25	0	1
USP17L26	0	1

USP17L27	0	1
USP17L28	0	1
USP17L29	0	1
USP17L3	0.001604193	0.304376143
USP17L30	0	1
USP17L4	0.00049228	0.717751436
USP17L5	0	1
USP17L7	-0.043003929	6.14E-05
USP17L8	-0.000903304	0.333198756
USP19	1.489575519	4.52E-79
USP2	0.326656928	0.00019892
USP20	0.471745228	5.36E-10
USP21	0.841918626	4.41E-38
USP22	0.612987124	7.94E-13
USP24	0.474809091	4.30E-09
USP25	0.141753752	0.016128678
USP26	0.005571652	0.000248994
USP27X	-0.281680996	0.010213194
USP28	1.080729177	4.08E-28
USP29	0.019712088	0.044446892
USP3	0.531615345	8.64E-06
USP30	0.984574469	5.27E-60
USP31	-0.368325081	9.85E-08
USP32	1.123697743	7.25E-61
USP33	0.607004194	6.63E-15
USP34	-0.230528107	0.001124666
USP35	0.148129212	0.017736374
USP36	0.604732131	3.44E-13
USP37	0.794826323	1.96E-48
USP38	2.039464435	5.37E-284
USP39	2.118346226	1.14E-295
USP4	1.275593425	4.18E-84
USP40	0.855485675	1.24E-65
USP41	0.384235785	3.25E-248
USP42	1.547453446	1.50E-103
USP43	-0.213996484	0.004996177
USP44	-0.466455648	6.88E-11
USP45	0.232658441	0.006993979
USP46	-0.128583208	0.017027005
USP47	-1.100463963	1.32E-58
USP48	0.413566548	2.95E-06
USP49	0.197059088	0.002150712
USP5	1.502584392	2.62E-90
USP50	0.334460566	6.43E-170
USP51	0.549335022	6.90E-36
USP53	0.136123861	0.014272362
USP54	-0.578265882	1.75E-16
USP6	0.0660765	0.028351245

USP6NL	1.317270605	1.69E-210
USP7	0.588586902	3.73E-17
USP8	0.901190257	2.80E-80
USP9X	1.219065794	5.59E-90
USP9Y	-0.223129391	0.092694406
USPL1	1.144870792	2.32E-63
UST	1.879115756	1.51E-129
UTF1	0.067090594	4.62E-06
UTP11	1.908537195	5.22E-250
UTP14A	1.715039845	6.25E-188
UTP15	1.660286749	2.93E-249
UTP18	1.681533912	8.24E-113
UTP20	1.515101188	1.18E-171
UTP23	1.450096154	2.95E-173
UTP3	1.748301483	1.17E-185
UTP4	2.00781484	2.81E-216
UTP6	1.238177741	4.06E-78
UTRN	0.933377984	4.61E-42
UTS2	-0.003749403	0.941852088
UTS2B	0.259236163	1.14E-33
UTS2R	-0.149848823	8.95E-05
UTY	0.447263465	3.25E-07
UVRAG	0.525741163	8.54E-16
UVSSA	0.639771919	4.70E-26
UXS1	0.905600971	1.10E-52
UXT	1.230762367	6.87E-138
VAC14	1.161164858	9.62E-92
VAMP1	-1.417789744	1.95E-42
VAMP2	-0.68744545	1.21E-14
VAMP3	1.870841191	6.07E-166
VAMP4	0.851182501	5.31E-35
VAMP5	2.883896416	1.61647520545752e-315
VAMP7	1.793827325	1.89E-191
VANGL2	2.58967809	2.87E-190
VAPA	0.809413461	2.42E-39
VAPB	0.451825552	5.37E-16
VASH1	0.78779448	3.37E-34
VASH2	0.836721591	3.66E-173
VASN	2.056642152	5.86E-122
VAT1	2.753307691	3.74E-238
VAT1L	-1.259933491	1.58E-17
VAV1	2.04927083	3.64E-158
VAV2	2.167847139	2.72E-234
VAV3	1.717430378	5.56E-154
VAX1	-0.248852889	8.87E-06
VAX2	0.962305964	8.95E-18
VBP1	1.796069591	3.83E-174
VCAM1	3.313382191	1.02E-276

VCL	1.855034689	9.78E-174
VCP	0.965351699	4.51E-45
VCPIP1	1.061784072	2.14E-103
VCPKMT	0.038865912	0.635753581
VCX	-0.013502869	0.421317484
VCX2	0.017667856	2.16E-07
VCX3A	-0.0450683	0.000239898
VCX3B	0.003441198	0.819065184
VCY	-0.000648368	0.434836846
VCY1B	0.004654592	0.000162499
VDAC1	1.396211179	3.57E-72
VDAC2	0.7088347	8.04E-24
VDAC3	0.34981973	1.21E-05
VDR	1.150995567	1.9178611584458e-316
VEGFA	1.835005431	5.00E-98
VEGFB	1.178281885	5.50E-166
VEGFC	0.330597887	5.46E-14
VEGFD	-0.870779137	2.44E-74
VENTX	1.09288807	5.83E-81
VEPH1	1.092459422	7.97E-150
VEZF1	2.215432824	1.16E-260
VEZT	1.087288356	3.76E-87
VGF	1.224857778	1.69E-13
VGLL1	0.015869528	2.22E-06
VGLL2	0.915470547	5.80E-136
VGLL3	0.102700017	0.000325545
VGLL4	1.973832374	8.43E-215
VHL	1.720047046	2.25E-120
VHLL	0.093287874	0.001119111
VIL1	0.003815436	0.548248843
VILL	-0.580787265	5.01E-27
VIM	3.986685322	2.13E-273
VIP	-1.70275216	2.45E-40
VIPAS39	1.048555225	7.35E-65
VIPR1	-1.964710578	2.66E-52
VIPR2	1.121638619	1.76E-84
VIT	0.117124989	0.033677252
VKORC1	-0.887315543	1.16E-67
VKORC1L1	1.810735957	1.30E-175
VLDLR	-0.057257631	0.43929408
VMA21	2.151933924	3.54E-276
VMAC	1.131361912	1.63E-98
VMP1	2.207056885	3.42E-225
VN1R1	-0.060944945	0.457894604
VN1R2	0.145405632	5.97E-58
VN1R4	0.003458678	0.03297414
VNN1	0.933391121	7.32E-265
VNN2	1.556780592	1.24E-208

VNN3	0.08909255	1.16E-25
VPREB1	0.04309904	4.48E-45
VPREB3	0.462118066	2.57E-24
VPS11	0.745368686	1.09E-26
VPS13A	0.121203396	0.119245514
VPS13B	0.963683071	1.59E-63
VPS13C	0.369116752	6.80E-07
VPS13D	0.312795851	1.29E-06
VPS16	1.282591263	5.30E-55
VPS18	1.668629887	5.52E-126
VPS25	2.126039766	2.89E-290
VPS26A	0.400601721	2.31E-09
VPS26B	0.707222098	8.76E-20
VPS28	1.220081873	1.21E-82
VPS29	1.378582915	1.93E-116
VPS33A	0.415507155	6.22E-12
VPS33B	-1.64070696	2.74E-92
VPS35	1.246363832	1.15E-67
VPS36	0.046583475	0.603188055
VPS37A	0.562692588	3.26E-18
VPS37B	1.317215598	4.35E-160
VPS37C	0.900554231	5.51E-63
VPS37D	1.541344562	1.01E-120
VPS39	0.483491749	1.57E-13
VPS41	1.40616905	2.39E-126
VPS45	0.494234368	3.19E-12
VPS4A	-0.298888851	7.19E-07
VPS4B	1.034073055	4.12E-60
VPS50	0.706646419	4.41E-22
VPS51	0.447572139	1.18E-08
VPS52	0.809398569	5.23E-31
VPS53	-0.16196525	0.064898848
VPS54	1.108767999	4.40E-70
VPS72	1.866629133	6.15E-205
VPS8	0.471650753	1.69E-14
VPS9D1	0.036849107	0.639713921
VRK1	1.377062586	1.84E-120
VRK2	0.806039198	2.48E-34
VRK3	0.743600173	1.40E-36
VRTN	0.011203983	0.000845548
VSIG1	0.027167134	0.46385622
VSIG10	1.149205287	2.77E-151
VSIG10L	0.19138659	0.001774213
VSIG2	0.143156005	0.002992919
VSIG8	-1.033973436	7.23E-21
VSIR	1.575464861	6.85E-104
VSNL1	-2.751420897	1.91E-40
VSTM1	0.868252229	1.84E-148

VSTM2A	-0.859195179	1.30E-09
VSTM2B	-2.567174323	5.74E-182
VSTM2L	-1.64320012	1.10E-38
VSTM4	0.676681439	2.17E-42
VSTM5	-0.735239498	1.64E-19
VSX1	-0.791177084	1.42E-14
VSX2	0.011956266	0.399857123
VTA1	1.674307461	3.82E-135
VTCN1	0.44167093	5.73E-294
VTI1A	0.783619058	3.75E-61
VTI1B	-0.561635407	7.09E-29
VTN	-1.16585434	8.10E-31
VWA1	1.937992241	4.86E-117
VWA2	0.585404319	1.18E-56
VWA3A	-0.108462091	0.319492059
VWA3B	0.739997947	4.07E-36
VWA5A	1.918165456	2.53E-94
VWA5B1	-0.028258916	0.598792615
VWA5B2	-3.391793463	2.08E-199
VWA7	-1.146372198	4.94E-27
VWA8	0.511954477	7.83E-18
VWC2	-0.761192865	3.07E-11
VWC2L	-0.012360796	0.86447294
VWCE	0.959383754	9.58E-53
VWDE	0.157564537	5.95E-15
VWF	2.384485338	2.00E-165
WAC	-0.541815728	8.54E-12
WAPL	1.07528341	1.58E-84
WAS	2.791688595	3.76E-297
WASF1	-0.447170482	3.05E-05
WASF2	2.268750105	1.19E-287
WASF3	-1.355005184	1.07E-101
WASHC1	0.191857224	0.013416306
WASHC2A	0.409301517	3.06E-10
WASHC2C	0.231139608	0.000856302
WASHC3	0.524188496	2.27E-25
WASHC4	1.48906263	3.53E-124
WASHC5	1.520657061	4.15E-132
WASL	1.509424496	2.91E-107
WBP11	1.475785101	3.05E-114
WBP1L	0.774074019	1.48E-72
WBP2	-0.476106264	1.59E-13
WBP2NL	-0.353564562	2.94E-35
WBP4	0.91793385	2.38E-61
WDCP	2.02321528	7.31E-204
WDFY1	0.97008737	4.73E-52
WDFY2	0.173762059	0.00010552
WDFY3	0.102370216	0.098401931

WDFY4	1.210604107	1.69E-150
WDHD1	1.805641773	5.21436882620852e-320
WDPCP	0.90835779	1.11E-143
WDR11	0.441934043	8.72E-10
WDR12	1.576675871	6.33E-159
WDR13	0.443944151	4.93E-18
WDR17	-0.180325896	0.023341787
WDR18	1.559496113	8.93E-87
WDR19	0.539906459	9.68E-11
WDR20	0.852745731	5.26E-58
WDR24	0.632207017	2.18E-18
WDR25	1.20890102	4.62E-127
WDR26	1.030543352	2.20E-56
WDR27	-0.242555047	0.000157978
WDR3	1.952486394	1.17E-279
WDR31	0.709799314	1.03E-55
WDR33	0.520161236	4.50E-12
WDR35	1.78496272	1.24E-278
WDR36	1.434956231	6.37E-117
WDR37	0.162467643	0.03130739
WDR38	1.499870334	3.85E-64
WDR4	1.750303438	2.94E-197
WDR41	1.481298404	4.84E-147
WDR43	2.068130194	1.11E-261
WDR44	1.042454181	1.74E-68
WDR45	-0.072177226	0.176352315
WDR45B	1.444835745	2.20E-169
WDR46	1.939764752	3.22E-169
WDR47	0.439205728	1.49E-07
WDR48	0.952258752	1.59E-61
WDR49	0.966829579	2.35E-57
WDR5	1.190582281	1.62E-56
WDR53	1.772783334	6.71E-234
WDR54	1.216353537	5.46E-50
WDR55	0.381454943	2.81E-08
WDR59	0.425687	5.98E-12
WDR6	1.401870314	3.64E-62
WDR61	0.272223906	1.33E-05
WDR64	0.045416572	2.66E-07
WDR7	0.851944378	9.02E-35
WDR70	0.972379315	2.13E-54
WDR72	0.198057316	1.35E-88
WDR73	-0.702799378	2.55E-39
WDR74	0.445832249	5.91E-11
WDR75	1.07785543	1.83E-56
WDR76	2.799471503	9.36E-290
WDR77	1.544848653	3.58E-179
WDR81	1.391056876	5.69E-111

WDR82	1.300695302	5.44E-61
WDR83	-1.350880868	2.48E-92
WDR86	-0.139088494	0.03711958
WDR87	-0.052583327	1.26E-13
WDR88	0.825825517	8.23E-191
WDR89	1.191931536	5.67E-121
WDR90	0.577939498	3.84E-10
WDR91	1.097393597	1.32E-49
WDR92	0.225871378	2.11E-05
WDR93	0.535595846	1.79E-35
WDR97	-1.015008118	3.88E-29
WDSUB1	1.016668506	2.59E-67
WDTC1	0.405001524	2.51E-11
WEE2	0.197660392	2.99E-67
WFDC1	-2.277140396	1.22E-204
WFDC10A	-0.035081673	0.000474839
WFDC10B	0.039660767	0.022988856
WFDC11	0.036367752	4.51E-06
WFDC12	0.045797092	2.68E-22
WFDC13	-0.022798953	0.023680182
WFDC2	0.258739741	0.000393508
WFDC3	0.522119015	9.69E-52
WFDC5	-0.008334691	0.588179913
WFDC6	-0.022803767	2.73E-05
WFDC8	0.013146709	6.80E-06
WFDC9	-0.001959383	0.517170153
WFIKKN1	-0.905237385	1.25E-19
WFIKKN2	0.144013712	0.02560397
WFS1	-0.183940529	0.023155369
WHAMM	0.660701364	7.84E-31
WHRN	-0.214744207	0.009466786
WIF1	-2.904272792	3.12E-63
WIPF1	2.02697262	2.93E-97
WIPF2	0.931303417	9.66E-57
WIPF3	-0.096438188	0.379908208
WIPI1	0.966852226	1.27E-47
WIPI2	0.895795132	1.54E-49
WIZ	0.708288139	1.11E-39
WLS	3.366347749	5.92E-259
WNK1	-0.127857075	0.047871766
WNK2	-1.941849836	2.15E-75
WNK3	1.242743805	1.67E-218
WNK4	0.819785211	2.30E-132
WNT1	-0.539554736	2.14E-12
WNT10A	0.244904514	6.72E-08
WNT10B	-2.086232632	1.11E-41
WNT11	-0.349307773	1.72E-11
WNT16	0.663221523	6.56E-59

WNT2	0.125104116	0.01029848
WNT2B	-0.404362594	2.57E-13
WNT3	0.94211566	7.96E-42
WNT3A	0.000238559	0.960610827
WNT4	0.207737106	1.37E-05
WNT5B	0.377766002	2.48E-15
WNT6	-0.18624106	0.066097654
WNT7A	0.795790322	4.59E-32
WNT7B	-0.159939286	0.020618604
WNT8A	-0.308492167	2.04E-21
WNT8B	-0.043442645	0.000808618
WNT9A	0.020910379	0.737143561
WNT9B	0.352040121	1.14E-51
WRAP53	1.837799846	1.12E-205
WRAP73	0.030013638	0.689352773
WRN	1.252788934	3.74E-123
WRNIP1	0.925344625	3.15E-42
WSB1	0.353652945	5.46E-06
WSB2	0.35065475	2.18E-07
WSCD1	2.58674521	2.52E-215
WSCD2	-2.76162452	2.89E-59
WT1	0.113540682	0.001384408
WTAP	1.827732181	6.46E-170
WTIP	0.513441381	2.38E-28
WWC1	0.267075388	3.84E-05
WWC2	0.197587804	0.000163293
WWC3	0.641990308	1.55E-24
WWOX	0.982205285	2.38E-90
WWP1	0.206495633	0.000168862
WWP2	0.766558311	3.55E-48
WWTR1	2.988672573	1.38E-306
XAB2	0.640373862	3.16E-19
XAF1	0.264342959	0.000114479
XAGE1A	0.01210579	1.43E-12
XAGE1B	0.008075022	3.77E-07
XAGE2	0.011953746	0.065821019
XAGE3	0.00053676	0.94122776
XAGE5	-0.002635716	0.667587002
XBP1	2.203057882	1.05E-173
XCL1	0.326367759	3.66E-62
XCL2	0.713041462	8.29E-201
XCR1	0.169139468	1.62E-181
XDH	0.024101603	0.000131224
XG	0.37005024	2.26E-94
XIAP	1.390683907	1.88E-129
XIRP1	0.375359247	2.39E-44
XIRP2	0.010172476	0.467353604
XK	-0.614260435	1.33E-12

XKR3	0.1406232	2.00E-108
XKR4	-0.067876155	0.178090222
XKR6	0.789232728	1.37E-119
XKR7	-0.912259167	6.53E-12
XKR8	0.553319322	1.11E-13
XKR9	0.318436701	1.38E-46
XKRX	0.252139442	9.89E-82
XPA	0.730025504	1.83E-45
XPC	0.222669661	0.000998959
XPNPEP1	0.613083305	2.53E-22
XPNPEP3	0.999127145	5.48E-152
XPO1	0.043984307	0.536418077
XPO4	0.961758988	3.41E-47
XPO5	1.549801443	6.51E-116
XPO6	1.02600523	2.33E-72
XPO7	0.964022698	7.91E-45
XPOT	1.649977158	2.50E-190
XPR1	2.193662178	4.36E-240
XRCC1	1.274502537	1.81E-74
XRCC3	-0.528509754	8.16E-14
XRCC5	1.7994515	4.20E-150
XRCC6	1.902109941	1.29E-152
XRN1	0.760002299	2.76E-26
XRN2	2.607799729	5.07E-283
XRRA1	0.038034434	0.499111268
XYLB	0.826863654	6.98E-75
XYLT1	0.623343226	2.16E-25
XYLT2	1.112719882	8.35E-58
YAF2	1.25351232	4.73E-135
YAP1	1.546699049	1.08E-122
YARS2	1.41046205	4.60E-123
YBEY	1.181025178	2.90E-103
YBX2	-0.231342727	0.000838735
YBX3	1.63506574	6.60E-66
YDJC	0.054899279	0.38187855
YEATS2	0.356068017	4.03E-06
YEATS4	2.385442806	3.37E-190
YES1	2.0843548	8.16E-286
YIF1B	0.777945608	1.15E-38
YIPF1	2.160866955	1.45E-276
YIPF2	1.579989065	1.01E-117
YIPF3	1.465129362	1.43E-143
YIPF4	1.220263837	3.72E-92
YIPF5	2.21852562	3.41E-213
YIPF6	1.571213025	7.24E-224
YIPF7	0.326522626	7.65E-22
YJEFN3	-3.088331538	9.74E-144
YKT6	1.029110004	3.25E-102

YLPM1	0.644917048	2.52E-24
YME1L1	1.217646961	4.50E-75
YOD1	0.769638244	4.74E-43
YPEL1	-0.854639056	2.15E-22
YPEL2	-1.046798207	6.91E-43
YPEL3	-0.539600835	4.98E-11
YPEL4	-1.856609212	7.50E-56
YPEL5	1.286049131	2.22E-74
YRDC	1.779149076	7.69E-154
YTHDC1	0.669880156	3.31E-25
YTHDC2	0.015241512	0.864563552
YTHDF1	1.881025857	1.59E-149
YTHDF2	2.326201446	2.07E-250
YTHDF3	1.837527056	2.52E-185
YWHAB	0.112510718	0.183224062
YWHAE	1.385939348	1.74E-156
YWHAG	0.760517416	1.08E-13
YWHAH	0.232482081	0.050413594
YWHAQ	1.956029473	2.44E-159
YWHAZ	0.88482341	3.70E-28
YY1	1.141807956	7.53E-78
YY1AP1	0.68045423	2.97E-20
YY2	0.783039548	6.97E-140
ZACN	-0.049738152	0.001652768
ZADH2	0.585763134	1.33E-17
ZAN	0.019769851	8.88E-10
ZAP70	0.241653228	1.66E-08
ZAR1	0.277445645	2.46E-27
ZAR1L	0.116166297	8.34E-75
ZBBX	-0.432142416	9.25E-06
ZBED1	1.930425729	1.47E-275
ZBED2	-0.14946657	0.000247794
ZBED3	1.577723443	3.30E-192
ZBED4	1.383944183	2.91E-85
ZBED5	1.323769354	1.92E-80
ZBED6	-0.583072831	4.88E-30
ZBED6CL	0.628808547	1.06E-50
ZBED8	1.307417809	1.02E-61
ZBED9	0.128012176	0.045366503
ZBP1	0.542528317	1.28E-68
ZBTB1	1.039548587	4.87E-89
ZBTB10	1.190211725	4.13E-79
ZBTB11	0.563088689	4.47E-15
ZBTB12	1.642853087	7.47E-202
ZBTB14	0.663926677	9.03E-45
ZBTB16	0.911198732	3.86E-27
ZBTB17	0.916874912	3.45E-58
ZBTB18	-0.1762039	0.207852021

ZBTB2	1.636258802	1.49E-190
ZBTB20	0.237776797	5.34E-15
ZBTB21	1.563740435	3.67E-194
ZBTB22	1.781058334	3.20E-167
ZBTB24	0.872711725	9.48E-52
ZBTB25	0.365541387	5.70E-12
ZBTB26	1.214143105	2.36E-88
ZBTB3	1.322418596	8.69E-245
ZBTB32	0.147367962	6.26E-14
ZBTB33	1.949493372	3.84E-200
ZBTB34	0.577428139	7.22E-20
ZBTB37	0.248389092	6.39E-09
ZBTB38	0.969333286	6.10E-43
ZBTB39	1.478467718	6.07E-205
ZBTB4	0.339860666	4.38E-07
ZBTB40	0.936969094	1.16E-41
ZBTB41	0.714707463	2.82E-21
ZBTB43	0.582639105	8.37E-23
ZBTB44	0.255672521	7.77E-06
ZBTB45	1.427790546	1.91E-123
ZBTB46	-0.442566434	3.29E-05
ZBTB47	-0.420730234	2.85E-18
ZBTB48	0.511620464	7.02E-18
ZBTB49	1.081711765	3.04E-76
ZBTB5	1.756123074	1.86E-179
ZBTB6	1.847313298	3.11E-225
ZBTB7A	-0.508712215	1.84E-09
ZBTB7B	1.169587219	2.46E-75
ZBTB7C	1.23851632	2.27E-103
ZBTB8A	0.886909074	6.10E-60
ZBTB8B	0.355165499	2.36E-38
ZBTB8OS	0.997177119	5.68E-86
ZC2HC1A	-0.080772743	0.308776663
ZC2HC1B	-0.054008488	1.58E-13
ZC2HC1C	-0.195527099	1.21E-06
ZC3H10	1.103129975	1.74E-183
ZC3H11A	-0.587197227	2.69E-55
ZC3H12A	1.493736416	2.14E-107
ZC3H12B	-1.028006597	5.16E-74
ZC3H12C	1.326107121	4.33E-137
ZC3H12D	0.547729167	2.39E-119
ZC3H13	0.404657168	1.52E-14
ZC3H14	0.607260762	1.96E-17
ZC3H15	1.276785237	2.97E-71
ZC3H18	0.105076626	0.172628878
ZC3H3	1.302101479	2.87E-87
ZC3H4	1.265588262	3.43E-78
ZC3H6	0.347669559	3.21E-09

ZC3H7A	0.996652002	2.19E-56
ZC3H7B	-0.363131638	4.42E-07
ZC3H8	0.265074502	4.07E-05
ZC4H2	1.237571055	1.90E-122
ZCCHC10	1.939703312	7.49E-249
ZCCHC12	-1.659501679	7.02E-26
ZCCHC13	0.002031865	0.319234597
ZCCHC14	0.439499189	4.38E-08
ZCCHC17	0.821915292	3.82E-42
ZCCHC18	0.503141244	4.95E-11
ZCCHC2	0.230789819	0.00010087
ZCCHC24	0.294043125	1.51E-05
ZCCHC3	1.719120361	3.12E-146
ZCCHC4	1.514212075	2.07E-166
ZCCHC7	1.7169741	2.49E-251
ZCCHC8	0.733455754	1.49E-24
ZCCHC9	1.440011578	1.24E-149
ZCRB1	-0.924538271	5.65E-69
ZCWPW1	0.247492972	5.74E-05
ZCWPW2	-0.099980503	7.99E-07
ZDBF2	-0.263523388	0.003172378
ZDHHC1	1.358160177	6.45E-126
ZDHHC11	-1.032581781	1.86E-28
ZDHHC11B	-2.538135095	2.50E-129
ZDHHC13	0.05000163	0.479256189
ZDHHC14	0.348428065	1.18E-06
ZDHHC16	1.104452462	8.66E-79
ZDHHC17	0.169567794	0.024889289
ZDHHC18	0.913188143	2.36E-49
ZDHHC19	-0.198965417	1.60E-08
ZDHHC2	0.906242392	1.18E-36
ZDHHC20	1.059366839	6.27E-36
ZDHHC21	0.276968152	0.000387413
ZDHHC22	0.029984116	0.753435869
ZDHHC23	0.356275864	5.69E-05
ZDHHC24	1.310348631	3.56E-172
ZDHHC3	0.908362347	1.89E-61
ZDHHC4	2.027849348	7.83E-264
ZDHHC5	1.565371226	1.11E-131
ZDHHC6	1.254179656	2.32E-125
ZDHHC7	1.339164229	1.62E-94
ZDHHC8	0.429625577	6.48E-10
ZDHHC9	1.846637216	1.11E-129
ZEB1	1.636903118	1.59E-244
ZEB2	0.070282024	0.436723552
ZER1	0.151147088	0.030999822
ZFAND1	0.229815756	0.003431521
ZFAND2A	1.073610891	2.31E-57

ZFAND2B	0.451599146	5.68E-10
ZFAND4	0.172483665	0.011557307
ZFAND5	0.850927861	3.77E-34
ZFAND6	1.424116938	3.80E-135
ZFAT	0.722319546	5.18E-57
ZFC3H1	-0.127738547	0.125530546
ZFHX2	-0.295227698	2.11E-06
ZFHX3	0.816826769	1.41E-61
ZFHX4	1.192905785	3.35E-106
ZFP1	1.252583396	1.05E-95
ZFP14	0.652916748	5.94E-31
ZFP2	0.476265292	1.41E-15
ZFP28	0.495990863	1.94E-19
ZFP3	1.564024862	2.76E-134
ZFP30	1.549220611	1.45E-132
ZFP36	2.470539376	2.09E-94
ZFP36L1	2.235333024	5.87E-161
ZFP36L2	2.472730962	1.27E-210
ZFP37	1.290192892	1.31E-118
ZFP41	0.274493005	4.45E-07
ZFP42	0.005146728	0.001397323
ZFP57	-1.004770014	1.79E-25
ZFP62	0.881212392	2.13E-58
ZFP64	1.788095193	3.08E-261
ZFP69	0.804791956	3.94E-70
ZFP69B	0.699625911	7.72E-44
ZFP82	0.39630657	3.16E-14
ZFP90	0.915115271	2.78E-61
ZFP91	0.701819237	2.12E-30
ZFP92	-0.413438333	3.77E-10
ZFPL1	-1.655757607	6.55E-221
ZFPM1	0.564680653	5.58E-35
ZFPM2	0.194483487	0.112410447
ZFR	0.844500603	2.08E-53
ZFR2	-1.605352379	3.10E-79
ZFX	1.495635282	1.09E-140
ZFY	0.820693522	1.73E-21
ZFYVE1	1.200133894	2.18E-103
ZFYVE16	-1.038753891	1.32E-48
ZFYVE19	0.498718302	1.43E-15
ZFYVE21	-2.073595636	3.47E-270
ZFYVE26	0.6628068	7.52E-21
ZFYVE27	-0.09362502	0.275641365
ZFYVE28	-0.821628226	1.01E-12
ZFYVE9	-0.104402468	0.20557589
ZG16	-0.019652483	0.005641496
ZG16B	-0.040356835	0.422569732
ZGLP1	-1.291964934	8.20E-51

ZGPAT	-1.965636542	8.79E-112
ZGRF1	0.561493956	4.27E-24
ZHX1	1.381698674	7.30E-167
ZHX2	1.570724925	3.79E-217
ZHX3	0.838025641	1.90E-47
ZIC1	1.024262815	3.85E-08
ZIC2	0.174072393	0.371584154
ZIC3	-0.561950097	7.53E-06
ZIC4	-0.766699102	2.89E-05
ZIC5	0.076073034	0.632796908
ZIK1	2.061579743	5.36E-185
ZIM2	-0.067221328	0.088070054
ZIM3	0.001773066	0.723635564
ZKSCAN1	0.883657625	2.19E-49
ZKSCAN2	1.008792931	2.75E-117
ZKSCAN3	1.279169025	1.33E-214
ZKSCAN4	1.170183394	1.45E-99
ZKSCAN5	1.916913501	2.83E-204
ZKSCAN7	1.211240517	1.32E-89
ZKSCAN8	1.396383281	2.49E-79
ZMAT1	-0.023752355	0.719395521
ZMAT2	1.24078504	3.59E-67
ZMAT3	1.406923955	2.61E-139
ZMAT4	-0.859656556	8.07E-13
ZMAT5	-0.691419093	5.61E-60
ZMIZ1	1.260750099	1.90E-73
ZMIZ2	0.393582568	5.97E-08
ZMPSTE24	2.021298964	9.92E-302
ZMYM1	1.730402162	4.98E-248
ZMYM2	0.49416868	2.21E-18
ZMYM3	1.201491554	9.64E-59
ZMYM4	1.548800456	8.51E-118
ZMYM5	1.02056149	4.63E-65
ZMYM6	0.186598369	0.0008784
ZMYND10	1.430629504	6.22E-58
ZMYND11	-0.065188444	0.244484185
ZMYND12	0.502700411	8.15E-17
ZMYND15	1.641329361	3.34E-186
ZMYND19	1.349901141	5.02E-97
ZMYND8	0.749657466	3.11E-35
ZNF10	0.705670678	2.89E-30
ZNF100	1.679969511	1.03E-155
ZNF101	1.073010135	1.66E-139
ZNF106	0.649832351	7.39E-36
ZNF112	1.022150281	1.96E-50
ZNF117	1.075507007	6.25E-83
ZNF12	1.774475951	1.03E-124
ZNF121	1.707093519	1.68E-207

ZNF131	1.137177894	5.90E-60
ZNF132	1.378603442	2.69E-89
ZNF133	0.639932665	1.50E-16
ZNF134	1.557244739	3.22E-90
ZNF135	-0.3993794	1.67E-12
ZNF136	1.42349014	7.24E-116
ZNF138	1.672090945	3.54E-165
ZNF14	1.473363074	7.58E-113
ZNF140	1.475883429	1.09E-123
ZNF141	0.202596399	0.000671435
ZNF142	0.135435126	0.054035816
ZNF143	1.211406216	1.08E-95
ZNF146	1.053438866	4.81E-66
ZNF148	1.578816415	1.58E-180
ZNF154	0.30702589	1.18E-05
ZNF155	0.602379616	1.09E-20
ZNF157	0.447799322	3.23E-22
ZNF16	0.751260046	2.21E-49
ZNF160	0.85351995	3.57E-42
ZNF165	0.451160406	1.14E-38
ZNF169	-0.129925294	0.023015647
ZNF17	0.613894057	2.41E-33
ZNF174	1.157311868	1.10E-78
ZNF175	0.660296788	3.23E-23
ZNF177	-1.57642475	5.24E-162
ZNF18	1.1698599	3.89E-147
ZNF180	1.440121356	3.96E-123
ZNF181	1.159417632	1.30E-81
ZNF182	0.230700613	0.002853998
ZNF184	1.624013386	2.09E-110
ZNF185	0.971923983	2.20E-85
ZNF189	0.852157907	1.03E-35
ZNF19	0.402359649	3.26E-35
ZNF195	-0.064123468	0.342165819
ZNF197	0.951492742	7.29E-66
ZNF2	1.513175825	1.57E-222
ZNF20	-0.880377068	7.95E-93
ZNF200	0.927203803	1.53E-62
ZNF202	1.020816204	2.12E-54
ZNF205	0.6474315	3.85E-33
ZNF207	1.307612783	3.98E-63
ZNF208	-0.408812234	6.26E-27
ZNF211	0.615188284	9.32E-18
ZNF212	1.860383892	2.28E-184
ZNF213	0.445540306	7.26E-13
ZNF214	0.369240809	5.01E-23
ZNF215	-0.081545622	0.001513668
ZNF219	0.456487361	2.89E-08

ZNF22	1.345496846	3.09E-176
ZNF221	0.452329501	2.27E-15
ZNF222	1.051577594	1.45E-58
ZNF223	-0.626463909	3.34E-27
ZNF224	-0.670625653	4.01E-18
ZNF225	0.846183788	5.54E-69
ZNF226	1.061239477	3.63E-83
ZNF227	1.033764499	1.44E-74
ZNF229	1.153355476	5.07E-112
ZNF230	0.908722322	1.23E-60
ZNF232	1.720263236	3.20E-177
ZNF233	-0.339006238	8.02E-09
ZNF234	1.438876071	4.00E-170
ZNF235	0.299178769	3.04E-08
ZNF236	0.042668431	0.458502195
ZNF239	1.230903984	3.51E-118
ZNF24	1.469919392	7.98E-151
ZNF248	-0.774254592	3.10E-17
ZNF25	-0.217522194	0.018550428
ZNF250	1.051466902	2.06E-56
ZNF251	-0.116306421	0.232340042
ZNF253	1.63012762	1.16E-124
ZNF254	0.735072484	2.51E-41
ZNF256	1.448232524	2.58E-124
ZNF257	0.302049063	3.54E-31
ZNF26	1.085446833	1.63E-183
ZNF260	2.062853539	1.90E-302
ZNF263	0.84996689	2.56E-37
ZNF264	1.14561846	9.49E-91
ZNF266	1.517599546	2.04E-68
ZNF267	1.173575815	4.72E-109
ZNF268	1.404270675	4.60E-249
ZNF273	-0.194393157	0.073871121
ZNF274	0.388989933	4.63E-07
ZNF275	1.480719267	1.45E-148
ZNF276	-0.937498054	1.36E-29
ZNF277	1.560604273	1.95E-215
ZNF28	1.898821025	4.71E-204
ZNF280A	0.110616463	5.36E-33
ZNF280B	0.111659342	0.051099588
ZNF280C	1.0233111	9.22E-124
ZNF280D	-0.037389127	0.531461148
ZNF281	1.329138546	1.58E-87
ZNF282	1.96921595	1.22E-142
ZNF283	0.763428293	1.33E-41
ZNF284	0.75874245	3.34E-44
ZNF285	0.386553497	1.01E-12
ZNF286A	0.697289442	3.99E-37

ZNF287	1.24820774	1.17E-154
ZNF292	0.575503311	6.98E-17
ZNF296	0.041193864	0.595128111
ZNF3	1.945812831	5.47E-209
ZNF30	0.659474241	8.17E-16
ZNF300	2.262176415	1.30E-191
ZNF302	0.582802818	1.94E-17
ZNF304	1.260320854	5.33E-56
ZNF316	0.743427767	3.32E-29
ZNF317	1.450641302	9.67E-103
ZNF318	0.959423129	5.12E-34
ZNF319	1.513546006	6.72E-158
ZNF32	0.241950474	6.03E-06
ZNF320	1.212535577	1.28E-74
ZNF322	1.401979996	3.53E-195
ZNF324	0.815721131	5.70E-31
ZNF324B	1.293510206	7.83E-151
ZNF326	0.334611414	6.61E-08
ZNF329	1.041862755	7.13E-53
ZNF330	1.192357277	1.10E-54
ZNF331	0.303893614	0.000226174
ZNF333	0.958526208	1.46E-33
ZNF334	0.743455233	1.53E-22
ZNF335	0.407650746	4.76E-07
ZNF337	-1.607038988	1.43E-39
ZNF33A	-0.051119678	0.556325313
ZNF33B	-0.098714558	0.279463604
ZNF34	0.212716454	0.007321511
ZNF341	-0.113191167	0.062256337
ZNF343	0.508685146	1.20E-14
ZNF345	1.179783972	5.72E-171
ZNF346	0.794086134	2.76E-36
ZNF347	1.033004986	6.99E-54
ZNF350	0.740840065	1.53E-41
ZNF354A	1.236293005	3.63E-120
ZNF354B	0.569357614	2.44E-15
ZNF354C	1.153394588	1.85E-61
ZNF358	-0.308505307	5.74E-09
ZNF362	1.48620449	6.87E-125
ZNF365	-0.374527482	1.16E-05
ZNF366	0.112659761	0.010621078
ZNF37A	-0.294752315	2.50E-05
ZNF382	-0.550452495	1.47E-07
ZNF383	0.818095685	5.06E-55
ZNF384	0.749072964	1.11E-17
ZNF385A	0.749634546	1.43E-45
ZNF385B	-1.473247651	3.79E-31
ZNF385C	-0.745183066	1.47E-13

ZNF385D	-0.152574767	0.10540705
ZNF391	0.885335321	4.65E-55
ZNF394	0.198799173	9.89E-05
ZNF395	1.159386693	3.20E-97
ZNF396	1.260745806	1.69E-293
ZNF397	0.695423602	4.51E-36
ZNF398	1.788769324	2.04E-149
ZNF404	0.138097835	0.040392172
ZNF407	0.457994029	1.24E-24
ZNF408	1.555343698	1.40E-216
ZNF41	1.110006648	2.04E-83
ZNF414	1.049559272	5.21E-68
ZNF415	0.672706679	3.12E-27
ZNF416	1.956647935	1.35E-206
ZNF417	1.079325691	8.55E-79
ZNF418	0.466778599	1.60E-10
ZNF419	0.128872153	0.043456883
ZNF420	1.384257532	4.35E-96
ZNF423	0.6265045	5.09E-35
ZNF425	1.30383123	7.16E-129
ZNF426	1.910243846	6.15E-275
ZNF428	0.387405677	2.79E-12
ZNF429	1.532433767	7.58E-191
ZNF43	1.867580867	4.18E-279
ZNF430	1.218028611	1.56E-122
ZNF431	1.028914046	3.57E-65
ZNF432	1.229805386	3.41E-82
ZNF433	1.258078634	3.46E-104
ZNF436	2.408637698	2.17E-288
ZNF438	1.089601918	1.14E-180
ZNF439	0.935973893	3.63E-30
ZNF44	0.993699648	1.31E-63
ZNF440	1.24059608	6.69E-83
ZNF441	0.601544098	1.55E-12
ZNF442	0.93730587	1.77E-143
ZNF443	1.115777806	2.73E-133
ZNF444	-0.359122829	2.20E-11
ZNF445	0.648609424	2.35E-22
ZNF446	-0.30681657	3.41E-06
ZNF449	0.756601505	1.49E-36
ZNF45	1.91973279	3.52E-201
ZNF451	0.524720189	1.19E-17
ZNF454	0.846797959	1.35E-65
ZNF460	0.341892299	5.56E-19
ZNF461	1.139554527	3.21E-54
ZNF462	1.433474338	3.83E-178
ZNF467	0.012558372	0.849592018
ZNF468	1.928868969	1.82E-177

ZNF469	0.601443631	8.07E-45
ZNF470	1.180623817	1.09E-127
ZNF471	0.166464497	0.011082835
ZNF473	1.307408684	7.11E-100
ZNF474	1.020844617	6.36E-130
ZNF479	0.011448836	5.33E-06
ZNF48	1.452390419	4.51E-86
ZNF480	1.91606052	1.09E-270
ZNF483	1.211447364	7.59E-119
ZNF484	0.612847849	2.18E-23
ZNF485	0.396859113	8.30E-24
ZNF487	0.42424479	1.38E-29
ZNF488	-0.656398606	9.29E-11
ZNF490	0.881950492	9.18E-88
ZNF491	1.250541966	3.09E-93
ZNF492	0.267612482	1.59E-84
ZNF493	-0.024850895	0.807354211
ZNF496	1.621573153	7.52E-130
ZNF497	-0.368522673	2.89E-08
ZNF500	0.963493753	5.54E-56
ZNF501	1.848221386	8.20148972096469e-321
ZNF502	1.888402315	2.98E-158
ZNF503	1.122334821	4.74E-49
ZNF506	0.349289959	2.35E-09
ZNF507	0.525438882	1.28E-20
ZNF510	1.050326902	5.25E-50
ZNF511	-0.929488183	4.67E-56
ZNF512	0.655392162	5.30E-25
ZNF512B	0.92133994	5.64E-28
ZNF513	1.045937032	1.11E-64
ZNF514	-0.464529551	2.14E-09
ZNF516	1.394477536	3.90E-132
ZNF517	0.493055946	2.93E-16
ZNF518A	0.109548599	0.09738863
ZNF518B	0.576655273	1.70E-26
ZNF519	-0.196231594	0.010069088
ZNF521	1.432872655	3.76E-28
ZNF524	0.952591691	2.42E-81
ZNF525	0.707207624	2.43E-36
ZNF526	1.368285193	5.86E-129
ZNF527	1.323210815	4.24E-192
ZNF528	0.757147809	7.31E-20
ZNF529	0.183730914	0.004367071
ZNF530	1.245142681	1.86E-70
ZNF532	0.826406463	9.97E-23
ZNF534	-0.310965484	8.65E-13
ZNF536	-1.2842045	1.86E-50
ZNF540	-1.758382862	3.39E-81

ZNF541	0.082785388	0.002192247
ZNF543	1.421130796	9.45E-193
ZNF544	0.882962176	2.75E-45
ZNF546	0.42965954	3.88E-20
ZNF547	0.53378307	8.05E-16
ZNF548	1.296387438	1.29E-87
ZNF549	0.759957706	8.65E-27
ZNF550	0.710458667	1.05E-25
ZNF551	1.103185397	2.53E-42
ZNF552	1.312643876	5.52E-209
ZNF554	-0.044704827	0.522991963
ZNF555	1.217957265	2.06E-136
ZNF556	-0.044777515	0.439046669
ZNF557	1.841443016	5.11E-282
ZNF558	1.87391758	2.26E-212
ZNF559	1.570896425	2.05E-163
ZNF559-ZNF177	-0.365845662	1.50E-16
ZNF560	0.686002068	2.92E-143
ZNF561	2.217502057	5.81278303326826e-313
ZNF562	1.444422133	2.63E-182
ZNF563	1.063546898	1.29E-199
ZNF564	-1.815399305	4.31E-268
ZNF565	0.659842957	4.45E-54
ZNF566	1.139060834	6.62E-73
ZNF567	1.388422959	4.05E-115
ZNF568	1.104341048	3.91E-119
ZNF569	1.197326286	1.24E-95
ZNF57	-1.47345787	5.08E-87
ZNF570	1.234816129	5.17E-151
ZNF571	0.834288285	1.61E-91
ZNF573	0.848812072	1.51E-138
ZNF574	1.58687716	4.10E-100
ZNF575	0.128074815	0.010757769
ZNF576	0.938220839	3.18E-52
ZNF577	0.269763024	9.80E-06
ZNF579	0.897242936	2.22E-27
ZNF580	0.590076187	1.67E-22
ZNF581	1.880546929	8.20E-168
ZNF582	0.79747027	7.21E-68
ZNF583	1.053689193	4.44E-141
ZNF584	1.51033964	2.62E-118
ZNF585A	0.349897149	1.07E-09
ZNF585B	1.04646477	2.30E-74
ZNF586	1.238467503	3.14E-112
ZNF587	0.267119485	9.87E-05
ZNF587B	1.116484571	4.18E-92
ZNF589	0.693732396	8.28E-17
ZNF592	1.15181937	2.77E-77

ZNF593	-0.030716513	0.014453077
ZNF594	0.454670015	1.58E-12
ZNF595	1.158739711	4.93E-81
ZNF596	0.546858751	6.69E-13
ZNF597	1.006377851	4.41E-115
ZNF598	0.627334047	3.45E-13
ZNF599	1.311008287	9.88E-171
ZNF600	0.109002382	0.321412208
ZNF605	-0.10669526	0.142211596
ZNF606	1.378048705	4.01E-115
ZNF607	0.980433148	3.01E-57
ZNF608	1.587843962	2.01E-123
ZNF609	0.773284794	3.26E-39
ZNF610	1.025169158	3.84E-117
ZNF611	0.762700762	3.10E-48
ZNF613	1.454270372	7.61E-268
ZNF614	1.879938863	5.37E-189
ZNF615	0.91609753	2.15E-51
ZNF616	1.735908962	1.41E-256
ZNF618	0.603742902	2.48E-16
ZNF619	1.202565548	6.40E-174
ZNF620	1.246696287	2.41E-117
ZNF621	1.335514571	6.33E-157
ZNF622	1.551204528	4.44E-123
ZNF623	1.300239124	3.87E-81
ZNF624	1.047004636	9.51E-182
ZNF625	-0.492973947	1.18E-21
ZNF626	1.366103152	1.00E-204
ZNF628	1.349488626	1.81E-146
ZNF629	1.60718322	1.19E-133
ZNF630	0.771534519	2.97E-52
ZNF638	-0.247253433	0.000475412
ZNF639	1.875382804	7.68E-276
ZNF641	1.13620107	1.00E-86
ZNF644	1.581677218	7.50E-150
ZNF646	1.010897426	1.14E-62
ZNF648	0.360939305	3.54E-237
ZNF649	1.792829967	7.49E-251
ZNF652	0.183579543	0.001065648
ZNF653	0.244340998	0.006035045
ZNF654	0.904064156	1.75E-57
ZNF655	0.840446141	2.61E-23
ZNF658	1.151250776	1.28E-160
ZNF66	0.454783485	1.68E-62
ZNF660	1.338259852	4.93E-137
ZNF662	0.576797712	1.75E-11
ZNF664	0.931925102	9.50E-60
ZNF665	0.977546962	1.32E-224

ZNF667	-0.166393324	0.011310547
ZNF668	0.460318947	1.85E-16
ZNF669	2.131958197	2.90E-257
ZNF670	1.336650854	1.18E-129
ZNF671	1.222335889	1.17E-41
ZNF672	1.500665962	3.01E-139
ZNF674	0.671399616	1.48E-60
ZNF675	0.892680361	1.85E-65
ZNF676	-0.057329319	0.019079158
ZNF678	1.321662841	3.21E-243
ZNF679	0.313457032	6.13E-87
ZNF680	1.37234646	2.13E-157
ZNF681	0.993463627	8.92E-74
ZNF682	0.837861329	2.79E-46
ZNF683	0.340127018	3.09E-36
ZNF684	1.381075675	4.88E-243
ZNF687	1.52268531	4.37E-113
ZNF688	1.061272758	9.32E-79
ZNF689	1.877245779	3.64E-219
ZNF69	1.462137171	2.50E-274
ZNF692	-0.406631766	5.38E-06
ZNF695	0.255612539	1.20E-78
ZNF696	1.059850978	4.19E-86
ZNF697	1.005145018	9.12E-82
ZNF699	1.75303713	4.12E-275
ZNF7	0.263415776	0.000158742
ZNF70	1.018564883	1.72E-108
ZNF700	1.0283975	1.31E-56
ZNF701	0.976781303	2.33E-139
ZNF703	1.293075068	9.33E-96
ZNF704	0.517937512	1.08E-10
ZNF705A	0.047229519	1.17E-51
ZNF705B	-0.000507727	0.504813421
ZNF705D	-0.000190274	0.831350806
ZNF705E	0.063335656	4.72E-07
ZNF705G	-0.004080793	0.069741803
ZNF706	0.721029016	7.41E-30
ZNF707	0.811650409	1.21E-65
ZNF708	1.058223003	9.70E-59
ZNF709	-0.946766183	6.95E-68
ZNF71	1.297326329	7.21E-148
ZNF710	0.740848039	8.37E-40
ZNF711	1.204160327	2.62E-70
ZNF713	1.278138338	3.09E-131
ZNF714	0.863085722	1.00E-39
ZNF716	0.031729333	2.87E-38
ZNF717	0.41873397	2.25E-37
ZNF718	1.021682852	1.73E-91

ZNF720	0.540183503	1.51E-17
ZNF721	0.04675977	0.540048086
ZNF724	0.881573987	1.02360915757906e-317
ZNF726	0.883306436	2.50E-135
ZNF727	0.364730313	1.22E-14
ZNF728	-0.093905419	0.000109242
ZNF729	0.087190254	6.83E-47
ZNF732	-0.429982857	1.46E-18
ZNF735	0.024217129	4.37E-14
ZNF736	1.364569856	1.58E-159
ZNF737	0.548300121	4.87E-21
ZNF738	1.422969612	8.26E-68
ZNF74	0.482585609	5.64E-15
ZNF740	0.750409963	3.63E-28
ZNF746	1.575333301	3.87E-140
ZNF747	0.372855344	5.91E-11
ZNF749	0.492008673	1.04E-17
ZNF750	-0.042883714	0.005942614
ZNF75A	0.240388887	0.00021366
ZNF75D	0.852454463	1.02E-55
ZNF76	0.636579303	2.12E-20
ZNF761	1.183302373	6.05E-84
ZNF763	0.03633414	0.447990403
ZNF764	1.370886046	2.18E-124
ZNF765	1.260597661	2.24E-287
ZNF766	1.291959827	1.20E-193
ZNF768	1.032808977	7.09E-114
ZNF77	1.639009583	4.19E-123
ZNF770	0.811818747	2.05E-23
ZNF771	0.260985835	4.19E-06
ZNF772	1.203837424	2.42E-93
ZNF773	0.914770693	2.00E-97
ZNF774	0.010436828	0.868691633
ZNF775	1.016086427	2.58E-104
ZNF776	1.428790184	5.27E-73
ZNF777	1.560754529	7.21E-138
ZNF778	0.587465848	9.20E-28
ZNF780A	0.82534363	3.22E-59
ZNF780B	0.298399829	0.00015771
ZNF781	0.016326949	0.847308827
ZNF782	0.598560076	8.18E-24
ZNF783	0.480244906	1.85E-11
ZNF784	1.430951465	2.61E-103
ZNF785	0.386267028	2.94E-09
ZNF786	1.430852189	4.97E-83
ZNF787	1.406566167	7.33E-180
ZNF789	0.601293308	3.54E-17
ZNF79	1.718740029	3.12E-204

ZNF790	1.200255327	1.29E-155
ZNF791	1.376455863	7.82E-110
ZNF792	1.264501321	2.67E-188
ZNF793	0.273780375	7.89E-05
ZNF799	0.568425107	3.94E-50
ZNF8	0.809569705	1.02E-35
ZNF80	0.054200481	4.69E-11
ZNF800	1.971597462	7.08E-283
ZNF804A	-0.2568352	0.002972709
ZNF804B	0.008150285	0.333397807
ZNF808	0.643436521	6.23E-49
ZNF81	1.242129294	1.16E-167
ZNF814	0.469653577	5.81E-14
ZNF816	1.250361347	1.07E-53
ZNF821	0.011227939	0.912244558
ZNF823	1.35176051	5.02E-103
ZNF827	0.134140544	0.073034175
ZNF829	1.089925014	2.72E-155
ZNF83	-0.021111278	0.84517881
ZNF830	1.623794198	5.16E-146
ZNF831	-0.362816615	2.73E-21
ZNF835	0.951387349	6.96E-59
ZNF836	0.757220546	1.37E-46
ZNF837	1.485529386	3.62E-278
ZNF839	0.316234487	2.22E-10
ZNF84	0.754217226	2.55E-36
ZNF841	0.242540985	0.007708637
ZNF843	1.157341017	3.48E-211
ZNF844	1.04375696	5.49E-71
ZNF845	1.524728331	3.10E-218
ZNF846	0.784827344	4.62E-69
ZNF85	1.685947809	4.64E-284
ZNF850	1.13042341	1.47E-285
ZNF852	0.348722151	6.65E-11
ZNF853	1.367439544	7.11E-163
ZNF860	0.152903718	3.79E-09
ZNF862	0.699491213	7.45E-20
ZNF865	1.170109861	3.30E-63
ZNF878	0.38055453	5.29E-29
ZNF879	1.144365625	1.71E-94
ZNF880	2.000900461	3.10E-250
ZNF888	1.032424904	1.03E-77
ZNF891	0.358460464	8.71E-14
ZNF90	0.836315198	4.16E-112
ZNF91	0.630614314	8.65E-19
ZNF92	2.192379566	1.32E-247
ZNF98	-0.258491088	9.93E-11
ZNF99	0.078524112	3.02E-27

ZNFX1	1.745028097	2.05E-239
ZNHIT2	1.212517521	6.79E-93
ZNHIT3	-0.181422838	0.004056268
ZNHIT6	1.156721902	2.82E-164
ZNRF1	-0.159570586	0.009027232
ZNRF2	1.389799621	2.61E-166
ZNRF3	0.598491235	2.00E-15
ZNRF4	-0.073541146	2.25E-08
ZP1	0.098201023	0.02919324
ZP2	-1.047840057	1.18E-08
ZP3	1.210309392	1.41E-98
ZP4	0.008729153	7.88E-14
ZPBP	-0.030982161	0.000224141
ZPBP2	-0.007582458	0.013059552
ZPLD1	0.055602108	8.85E-14
ZPR1	1.514954045	7.36E-157
ZRANB1	-1.243824598	2.52E-60
ZRANB2	0.378113418	5.25E-06
ZRANB3	0.882199808	1.04E-185
ZRSR2	0.3045438	4.11E-09
ZSCAN1	0.29048894	4.37E-07
ZSCAN10	-0.076999057	0.001281167
ZSCAN12	1.401332739	1.46E-126
ZSCAN16	1.378595797	5.60E-80
ZSCAN18	-0.558693906	7.02E-18
ZSCAN2	0.688800205	1.84E-77
ZSCAN20	0.741749841	3.49E-101
ZSCAN21	1.018098243	3.73E-70
ZSCAN22	1.11717658	3.45E-88
ZSCAN23	0.589675498	3.49E-58
ZSCAN25	1.507522263	4.73E-131
ZSCAN26	0.863249237	7.99E-35
ZSCAN29	0.957121984	4.95E-65
ZSCAN30	0.849598952	6.86E-30
ZSCAN31	0.62951116	3.62E-19
ZSCAN4	-0.077747858	0.000919634
ZSCAN5A	0.889880157	2.33E-178
ZSCAN5B	0.043155701	0.004415026
ZSCAN5C	0.107658243	1.70E-29
ZSCAN9	1.418246575	2.19E-84
ZSWIM1	1.612666582	1.24E-114
ZSWIM2	0.138135956	4.14E-21
ZSWIM3	1.446652294	1.38E-133
ZSWIM4	0.767025916	6.11E-65
ZSWIM5	0.847886608	1.17E-27
ZSWIM6	0.778283971	3.70E-45
ZSWIM7	1.491854593	4.42E-210
ZSWIM8	-0.275570476	0.0007263

ZW10	1.876906435	2.69E-181
ZWILCH	1.77362862	4.89E-198
ZXDA	1.241073056	2.05E-181
ZXDB	1.138441485	6.17E-74
ZXDC	1.052600768	2.74E-65
ZYG11A	0.067673813	0.00024256
ZYG11B	0.943327128	1.03E-30
ZYX	2.784315817	4.23E-187
ZZEF1	0.477547601	4.47E-11
ZZZ3	1.478266399	1.72E-201
