# Supplementary material for: LYZ Gene as a Novel Therapeutic Target and Diagnostic Biomarker in Glioblastoma: Insights from Multi-Omics Analysis and Functional Validation
Source: Biology (Basel). 2025 Dec 19;15(1):9. doi: 10.3390/biology15010009 (PMC12784846; doi:10.3390/biology15010009)
Supplement: Supplementary file 1 [file biology-15-00009-s001.zip › Supplementary Files/Table S2.pdf]

gene	logFC	padj
A1BG	0.096350397	0.540855396
A2M	1.674011214	0.011584437
A2ML1	-0.093037709	0.761697772
A4GALT	0.034404816	0.84159081
A4GNT	-0.049929336	0.699043016
AAAS	0.533156351	0.002419881
AACS	-0.657372417	0.015388688
AADAC	-0.105309772	0.217414475
AADACL2	-0.046448905	0.623712104
AADAT	0.089395733	0.735783159
AAGAB	-0.239380521	0.119909064
AAK1	-0.615002452	0.006130132
AAMDC	0.256447215	0.183878897
AAMP	-0.02442333	0.85369463
AARD	-0.045470497	0.724508351
AASDH	0.645653742	0.00140273
AASDHPPT	-0.076666865	0.774227942
AASS	1.296293266	0.001121126
AATF	0.147206458	0.58684035
ABAT	-0.071214975	0.919802651
ABCA1	2.259041783	4.88E-05
ABCA10	-0.487821468	0.383502248
ABCA12	0.129754769	0.401711068
ABCA4	-0.113293301	0.244535218
ABCA6	0.053186069	0.945950671
ABCA7	-0.037115713	0.770931382
ABCA8	-0.319117381	0.587451226
ABCA9	-0.144961164	0.777774563
ABCB1	-0.382435484	0.219212502
ABCB10	0.409575009	0.013636016
ABCB11	0.029845664	0.821868934
ABCB4	0.324131452	0.174741506
ABCB5	-0.071683764	0.455518383
ABCB7	0.81846293	0.002287614
ABCB9	-0.446390084	0.001597225
ABCC1	0.57305714	0.218780466
ABCC11	0.080478691	0.435380816
ABCC12	-0.071767462	0.329381327
ABCC2	0.156949546	0.346507164
ABCC3	2.876096132	0.008712348
ABCC4	0.402749732	0.170897774
ABCC8	-0.982774006	0.041889564
ABCC9	0.035668826	0.96051513
ABCD1	0.423848067	0.061198429
ABCD2	-0.223871209	0.747772576
ABCD4	0.835093481	0.000980529
ABCE1	0.209628094	0.549869358

ABCF2	0.159892101	0.571294279
ABCF3	0.120363232	0.382513211
ABCG1	0.31526392	0.542074826
ABCG2	0.111027967	0.869211288
ABCG4	-0.783054839	0.001072162
ABCG5	-0.098172127	0.247249456
ABCG8	-0.03910456	0.917640056
ABHD1	0.052781281	0.691358344
ABHD10	0.047190501	0.828352568
ABHD13	-0.004885224	0.991179488
ABHD14B	0.018777775	0.92654239
ABHD15	0.467273045	0.016124218
ABHD16B	0.088013364	0.592963535
ABHD17A	-0.019404725	0.951389636
ABHD17B	-0.822459593	0.012910317
ABHD18	0.043978061	0.845823862
ABHD2	0.229006515	0.449814104
ABHD3	0.891795266	0.000644185
ABHD5	0.193716166	0.327149371
ABHD6	-0.486717696	0.172583942
ABHD8	-0.968865316	0.002245834
ABI1	-0.337263183	0.195632868
ABI2	-0.053232648	0.868516953
ABI3	1.212891199	0.004734877
ABI3BP	0.796382214	0.413482691
ABITRAM	0.228093086	0.307608195
ABL1	0.28098642	0.412818927
ABL2	0.120113704	0.690801599
ABLIM1	-0.256337914	0.542131824
ABLIM3	-1.043094108	0.019234826
ABO	-0.066886525	0.673702005
ABR	-0.895241882	0.007676985
ABRA	-0.104726178	0.385155342
ABRAXAS1	0.320086728	0.258017379
ABRAXAS2	-0.269131913	0.127006537
ABT1	0.289889743	0.088557311
ABTB1	-0.137533867	0.759749878
ABTB2	-0.715120338	0.016048589
ACAA1	0.103284388	0.539346863
ACAA2	-0.801992189	0.100754919
ACACB	-0.064030093	0.900965117
ACAD10	-1.293302415	0.014933685
ACADL	-0.353675442	0.070843893
ACADM	-0.182314019	0.478055967
ACADS	-0.128150601	0.477276276
ACADSB	-1.038386656	0.005081616
ACADVL	0.201320483	0.598057051
ACAN	0.05663213	0.804686639

ACAP1	0.077294048	0.717706165
ACAP2	0.179119716	0.390361433
ACAP3	0.138631456	0.674436492
ACAT1	0.021577343	0.92170344
ACAT2	-0.292868918	0.277203467
ACBD3	0.202115432	0.319009971
ACBD5	-0.343795732	0.179409519
ACCS	0.896233061	0.009379327
ACE	0.359960469	0.086164987
ACE2	-0.097480506	0.20444268
ACER1	-0.058714735	0.61937456
ACER3	0.177240269	0.52082598
ACHE	-0.049199545	0.863600504
ACKR1	-1.197539118	0.139711058
ACKR3	0.784728503	0.023949051
ACKR4	-0.057479708	0.646762269
ACLY	0.466273758	0.214566503
ACMSD	-0.105802002	0.397286248
ACO1	-0.075513906	0.791787659
ACOT11	0.219769632	0.570269053
ACOT12	0.233992172	0.240533047
ACOT2	-0.241661777	0.480418384
ACOT4	-0.345454982	0.013337633
ACOT7	-0.073934556	0.674472706
ACOT8	-0.152280387	0.40680463
ACOT9	0.327390177	0.349485997
ACOX2	0.797810164	0.057770183
ACOX3	0.146461581	0.518807683
ACP2	0.156426121	0.442276137
ACP4	-0.175175721	0.245965752
ACP6	0.365702837	0.185113918
ACP7	-0.35839316	0.001338346
ACR	-0.099552187	0.602021763
ACRBP	0.3138317	0.122238399
ACRV1	-0.025726508	0.911063493
ACSBG1	-0.496269031	0.315226442
ACSBG2	-0.082009012	0.453509269
ACSF2	-0.000301139	0.998986205
ACSL1	0.241091199	0.661570092
ACSL3	-0.09339035	0.871438443
ACSL4	-0.38386321	0.242750377
ACSL5	-0.14802459	0.541226385
ACSM1	-0.11078211	0.33577345
ACSM2B	-0.094087828	0.52317169
ACSM3	-0.052684418	0.786914873
ACSM4	-0.088747735	0.479651966
ACSM5	0.614774351	0.078652069
ACSM6	-0.173888616	0.041889564

ACSS1	0.028783341	0.963152726
ACSS3	0.956312131	0.132468097
ACTA1	-0.333250715	0.049134091
ACTB	0.97414436	0.006944018
ACTC1	0.018229877	0.901611306
ACTG2	-0.223762559	0.469562447
ACTL6A	0.617707174	0.027403978
ACTL6B	-1.295723878	0.017293351
ACTL7A	-0.197318337	0.104998976
ACTL7B	-0.073984206	0.563836061
ACTL8	-0.142503539	0.403872871
ACTL9	-0.09449448	0.56648264
ACTN2	0.845675509	0.18647246
ACTN3	-0.361198451	0.017880032
ACTR10	-0.14834788	0.468633709
ACTR1A	-0.96174967	0.00168267
ACTR1B	-0.72080973	0.004659234
ACTR3	0.230087298	0.556390362
ACTR5	0.309521909	0.046796831
ACTR6	0.053532873	0.845786531
ACTR8	0.481210969	0.031966518
ACTRT1	0.02585623	0.77132529
ACTRT2	-0.153662693	0.166871868
ACTRT3	-0.304241772	0.154088396
ACVR1	0.123247824	0.677044272
ACVR1B	-0.061730939	0.816408453
ACVR1C	-0.706509897	0.021411228
ACVR2A	-0.007033692	0.984267688
ACVR2B	0.109280091	0.709237121
ACVRL1	0.215824405	0.180150463
ACY3	-0.68011417	0.107655525
ACYP1	0.352126505	0.078394754
ACYP2	0.172947923	0.542419383
ADA	0.065514698	0.84451194
ADA2	0.184470301	0.512058508
ADAD1	-0.065545087	0.448091491
ADAD2	-0.072104507	0.679091192
ADAM10	-0.148980388	0.515912866
ADAM11	-1.164972557	0.003806486
ADAM12	1.04873377	0.260514626
ADAM15	0.019316159	0.89921021
ADAM17	0.704008038	0.009598887
ADAM18	-0.061289299	0.601588011
ADAM19	0.132614898	0.872692977
ADAM2	0.049477124	0.522781348
ADAM20	0.367528342	0.263782233
ADAM21	-0.058865792	0.603713048
ADAM22	-0.252167317	0.675171286

ADAM23	-0.678475723	0.148529375
ADAM28	1.160430776	0.068213095
ADAM29	-0.072333427	0.453146081
ADAM30	-0.203228515	0.057828053
ADAM32	0.179380262	0.513684677
ADAM33	0.472546761	0.267750784
ADAM7	-0.003675805	0.975197365
ADAM8	0.054080766	0.843090108
ADAM9	0.820913047	0.098998399
ADAMTS1	-0.173780686	0.780791174
ADAMTS10	0.045904526	0.915817139
ADAMTS12	-0.12476491	0.897153565
ADAMTS13	0.018725986	0.919974005
ADAMTS14	0.312691027	0.532865057
ADAMTS15	0.043044675	0.939960521
ADAMTS16	-0.860626416	0.08194754
ADAMTS17	-0.348091342	0.133797118
ADAMTS18	0.092943592	0.837559998
ADAMTS19	-0.629214624	0.020744557
ADAMTS2	0.164406627	0.794786666
ADAMTS20	0.086478121	0.723956289
ADAMTS3	0.117383179	0.705319352
ADAMTS4	-0.206502669	0.752135041
ADAMTS5	0.227868758	0.456385946
ADAMTS6	1.17471292	0.009991617
ADAMTS7	-0.374172331	0.266586282
ADAMTS8	-0.721385213	0.00454605
ADAMTS9	2.952470178	0.000162776
ADAMTSL1	-0.123664762	0.806814268
ADAMTSL2	-0.253299129	0.085641646
ADAMTSL3	-0.491270033	0.010204925
ADAMTSL4	0.229239593	0.251206315
ADAMTSL5	-0.728579538	0.000783213
ADAP1	-1.092497781	0.001554747
ADAP2	1.731264175	0.001422408
ADARB1	-0.119028462	0.760420863
ADARB2	-1.015563669	0.001100481
ADAT1	0.379447117	0.020874654
ADAT2	0.759618879	0.000958583
ADAT3	0.409605716	0.022188125
ADCK1	-0.118695861	0.456985798
ADCK2	0.254680588	0.179104238
ADCK5	0.024916655	0.896168353
ADCY1	-1.52415823	0.070480847
ADCY10	-0.114488988	0.276265527
ADCY2	-0.781195379	0.228350481
ADCY3	0.026556679	0.911188953
ADCY4	0.097632956	0.428889748

ADCY5	-1.25824221	0.00637289
ADCY6	0.288111171	0.194609227
ADCY7	0.867670772	0.018191013
ADCY8	0.5757433	0.325895121
ADCY9	-0.164547873	0.46525882
ADCYAP1	-0.091100003	0.47142387
ADD1	-0.058753817	0.756015246
ADD2	-0.832547391	0.137059501
ADD3	-0.772568384	0.021925901
ADGRA2	0.024251101	0.962306545
ADGRB1	-0.109113437	0.876312032
ADGRB2	-0.233603538	0.576288716
ADGRB3	-0.615269206	0.494264726
ADGRD1	0.262188385	0.258131781
ADGRE2	1.269561395	0.010472913
ADGRE3	0.059042482	0.642315919
ADGRE5	1.260940542	0.038690557
ADGRF1	-0.112943384	0.391852408
ADGRF2	-0.139844784	0.120693464
ADGRF4	-0.055629692	0.581403145
ADGRF5	0.169950919	0.77121553
ADGRG3	-0.033467872	0.8146759
ADGRG4	-0.129826958	0.196667281
ADGRG5	-0.075194205	0.553712492
ADGRG6	0.297562986	0.57456578
ADGRG7	0.012883847	0.912619479
ADGRL2	-0.473884501	0.161711365
ADGRL3	0.328860741	0.657767191
ADGRV1	0.324833359	0.661615479
ADH1A	-0.547702629	0.013186951
ADH1B	0.06554989	0.608118412
ADH4	-0.001635365	0.992101096
ADH5	0.137181841	0.418584277
ADH7	-0.065199264	0.540515276
ADHFE1	0.023451259	0.9698923
ADI1	0.183233183	0.450867076
ADIPOR2	-0.397641536	0.133584504
ADIRF	-0.995539087	0.003170495
ADK	-0.502291666	0.059283877
ADM	1.037681523	0.178351374
ADM2	-0.050798551	0.757944468
ADM5	0.123260041	0.453919165
ADNP	0.458196529	0.025425025
ADNP2	0.224528829	0.339439747
ADO	-0.152157318	0.512415786
ADORA1	-0.083983606	0.856635804
ADORA2A	-0.053609858	0.760373199
ADORA2B	-0.359417588	0.290292168

ADORA3	0.602932757	0.100768054
ADPRH	0.14295229	0.332875404
ADPRHL1	-0.520257152	0.007280477
ADPRM	-0.034712743	0.873377072
ADRA1A	-0.411000792	0.132445969
ADRA1B	-0.614412273	0.006622958
ADRA1D	-0.227901099	0.480024633
ADRA2A	-0.338538765	0.184229981
ADRA2C	-0.22479389	0.279849035
ADRB1	-0.610479079	0.147759624
ADRB2	0.22107372	0.517739911
ADRB3	0.177569961	0.320054526
ADRM1	-0.187686032	0.37261928
ADSL	0.262909658	0.392538389
AEBP1	1.20277079	0.011514841
AEBP2	-0.094630338	0.396337371
AEN	0.015387759	0.958376062
AFAP1L2	0.02393157	0.943265544
AFF2	-1.350200718	0.013337633
AFF3	-1.095364293	0.002934498
AFF4	0.200648914	0.131673102
AFG1L	0.356070408	0.140000239
AFG3L2	0.120127821	0.405122436
AFM	-0.017625546	0.87676402
AFMID	0.432506512	0.3018865
AFP	-0.034155172	0.711061769
AFTPH	0.036553968	0.845640394
AGA	0.347952341	0.055109107
AGAP2	-1.659862168	0.032439335
AGAP3	0.015663862	0.951240846
AGAP4	0.101745315	0.777448942
AGBL1	0.040135885	0.769049389
AGBL2	0.59855807	0.073103353
AGBL3	0.361246191	0.134201228
AGBL4	-0.997192631	0.023282345
AGBL5	0.463108441	0.006626793
AGER	0.352856175	0.068403293
AGFG2	0.454019411	0.164120587
AGGF1	-0.020206743	0.917640056
AGK	0.475508923	0.014547991
AGL	0.211518464	0.478648665
AGMAT	0.093777283	0.828704205
AGO1	-0.023487656	0.896359297
AGO2	0.742501657	0.015257307
AGO3	0.503530896	0.018747154
AGO4	0.477878052	0.005869586
AGPAT1	-0.141643048	0.206794521
AGPAT2	-0.170298587	0.320910782

AGPAT3	-0.104257314	0.556316524
AGPAT4	0.10991733	0.780791174
AGPAT5	1.084939927	0.002172874
AGPS	0.14085688	0.65039546
AGR2	-0.055173311	0.589913524
AGR3	-0.014970145	0.901235662
AGRP	-0.025133914	0.824830805
AGT	0.114858581	0.838917532
AGTPBP1	-1.342399411	0.002032218
AGTR1	-0.275381424	0.329369557
AGTR2	-0.074475614	0.45779214
AGXT	-0.123745689	0.383453533
AGXT2	-0.142405812	0.322796921
AHCTF1	0.420576609	0.007810854
AHR	0.457378439	0.577380573
AHRR	0.008035076	0.973153649
AHSA1	-0.09825559	0.602782963
AHSP	0.04707422	0.621975967
AICDA	-0.123101635	0.254297669
AIF1	0.885299182	0.158572001
AIG1	-0.169444979	0.531569134
AIM2	0.291517559	0.290822404
AIMP2	0.220756255	0.152774879
AIP	-0.230894638	0.166614037
AIPL1	-0.037740851	0.745484819
AIRE	-0.194205651	0.106200492
AJAP1	-1.264276894	0.063600421
AJM1	-1.162630856	0.003602867
AJUBA	-0.070144297	0.872118673
AK3	-0.260230465	0.496993744
AK5	-3.245378453	0.000978538
AK7	-0.060882463	0.741269852
AK8	0.18282804	0.348905442
AKAIN1	-0.063888771	0.55590067
AKAP10	0.336609937	0.022188125
AKAP11	-0.816251358	0.032557927
AKAP12	-0.364675387	0.418633626
AKAP14	0.150175208	0.583914788
AKAP3	-0.00529539	0.969265127
AKAP4	-0.130297608	0.205994007
AKAP5	-0.865682303	0.008578399
AKAP6	-0.532568139	0.312995875
AKAP7	0.069711811	0.74931783
AKAP8L	0.385110288	0.067455431
AKAP9	0.17794736	0.383328795
AKIRIN1	0.146173834	0.509811843
AKIRIN2	0.040472214	0.882120044
AKNA	0.536083985	0.056755453

AKNAD1	0.431738889	0.206897977
AKR1A1	0.347502623	0.189223645
AKR1B1	0.283123759	0.121743152
AKR1B10	-0.090416932	0.447828731
AKR1B15	-0.05571575	0.735741986
AKR1C3	-0.196691995	0.161642263
AKR1C4	-0.007565731	0.943452168
AKR1D1	-0.11924188	0.222935337
AKR7A2	-0.198636896	0.436247993
AKR7A3	-0.041529509	0.878810579
AKT1	0.569251975	0.260068972
AKT1S1	0.356507248	0.056010851
AKT2	0.266456278	0.117943354
AKT3	-0.562026241	0.14030305
ALAD	-0.497190172	0.015713756
ALAS1	-0.160934134	0.348378671
ALAS2	-0.022951969	0.89921699
ALB	0.168932612	0.082563249
ALCAM	-0.611775045	0.218587045
ALDH16A1	-0.065894951	0.57448557
ALDH18A1	0.064932743	0.91445925
ALDH1A1	-1.417307706	0.028516008
ALDH1A3	-0.115993968	0.362847187
ALDH1B1	-0.746114221	0.11456237
ALDH2	-0.15676122	0.266895823
ALDH3B1	0.487086048	0.035852195
ALDH3B2	-0.058889009	0.734926031
ALDH4A1	-0.720803128	7.38E-05
ALDH5A1	-0.507756353	0.126596008
ALDH6A1	-1.075530811	0.025792332
ALDH7A1	0.025567284	0.953304329
ALDH8A1	-0.592782323	0.004734877
ALDH9A1	0.249677333	0.168681476
ALDOB	-0.130694024	0.244395196
ALDOC	-1.180115538	0.110456596
ALG1	0.279171702	0.380376396
ALG10	0.050266738	0.877887386
ALG11	-0.115836535	0.686864683
ALG12	-0.029879208	0.914554541
ALG14	0.140010795	0.532011599
ALG3	0.259539633	0.320910782
ALG5	0.350965002	0.061272135
ALG6	1.415752245	0.001899959
ALG8	0.571488245	0.013616856
ALG9	0.483244302	0.02664493
ALK	0.380455595	0.407647032
ALKAL1	-0.013449907	0.915607882
ALKAL2	-0.020711046	0.898588561

ALKBH1	0.156254669	0.521936247
ALKBH3	0.324538159	0.101166387
ALKBH4	-0.07026171	0.626037333
ALKBH5	0.156947958	0.198390135
ALKBH6	-0.017925643	0.943265544
ALKBH7	0.140670889	0.422691316
ALKBH8	0.207342246	0.281678876
ALLC	-0.189574047	0.101874794
ALMS1	0.386000578	0.106009241
ALOX12	-0.17801784	0.049033022
ALOX12B	-0.275606251	0.010011188
ALOX15	0.009973967	0.938914308
ALOX5	1.585672788	0.00166856
ALPG	-0.482900461	0.024618793
ALPI	-0.356719836	0.036219963
ALPK1	0.759124629	0.042762475
ALPK2	-0.76435388	0.290587504
ALPK3	0.927998898	0.020767647
ALPL	-0.597829923	0.138969461
ALS2	-0.015957956	0.936740169
ALX1	-0.094671117	0.250700001
ALX3	-0.018559263	0.942601244
ALX4	-0.159405828	0.331005671
ALYREF	0.562484257	0.051165623
AMBN	0.147779111	0.52317169
AMBP	-0.190477148	0.067203361
AMD1	-0.619031684	0.051585672
AMDHD1	-0.135039476	0.396542579
AMDHD2	-0.736426636	0.012927536
AMELX	-0.013631339	0.903945468
AMELY	-0.006645097	0.969065635
AMER1	0.129873938	0.526830681
AMER2	-0.910462553	0.084312483
AMFR	-0.139538519	0.506700199
AMH	0.490744443	0.134840884
AMHR2	-0.027029047	0.780791174
AMIGO1	-0.997964708	0.011978751
AMIGO3	0.233843705	0.220366706
AMMECR1L	0.493276562	0.057102086
AMN	-0.246616489	0.097030992
AMOT	-1.356783994	0.024017588
AMOTL1	0.412323892	0.160871323
AMOTL2	-0.458609663	0.170897774
AMPD1	-0.010402327	0.969720099
AMPD2	-0.247605784	0.275891463
AMPH	-1.36313279	0.040927029
AMTN	-0.501443472	0.083948157
AMY1A	0.026240956	0.951389636

AMY2A	-0.136111132	0.309990191
AMZ2	0.043331034	0.904553943
ANAPC1	0.331837435	0.07974385
ANAPC15	0.089713044	0.653942911
ANAPC16	0.309127463	0.414394125
ANAPC2	0.255989822	0.172583942
ANAPC4	0.419228287	0.017140888
ANAPC5	0.390694526	0.015428558
ANG	0.310396689	0.281151723
ANGEL1	0.596161723	0.041513123
ANGEL2	0.300850004	0.149621879
ANGPT1	0.212135983	0.681290959
ANGPT2	2.563150253	0.001607242
ANGPT4	-0.140703798	0.373969966
ANGPTL1	0.739863757	0.257228425
ANGPTL2	1.137645449	0.010684805
ANGPTL3	-0.080234732	0.536239224
ANGPTL4	-0.000245547	0.999827462
ANGPTL5	-0.024020303	0.779159502
ANGPTL6	0.170303526	0.355317656
ANGPTL7	-0.132364002	0.228630078
ANGPTL8	-0.157986807	0.443331542
ANHX	0.14654939	0.255690986
ANK2	-0.71403959	0.302557784
ANKDD1A	0.173161196	0.575581907
ANKDD1B	0.026162	0.893616805
ANKEF1	0.051836492	0.770820326
ANKFN1	1.712141822	0.000627774
ANKFY1	0.631352818	0.005139482
ANKH	-0.772461642	0.053556882
ANKHD1-EIF4EBP3	0.589057149	0.000722046
ANKK1	0.17739149	0.312373019
ANKLE1	0.264493968	0.071012042
ANKMY1	0.265036942	0.19285978
ANKMY2	-0.035085346	0.917640056
ANKRA2	0.742756881	0.000608526
ANKRD1	-1.475727459	0.125602685
ANKRD10	1.634171756	0.000165953
ANKRD12	-0.130722813	0.724508351
ANKRD13A	0.269973522	0.459874564
ANKRD13B	-0.256765717	0.3176019
ANKRD13C	-0.108753018	0.651630061
ANKRD17	0.164070476	0.246622033
ANKRD18A	-0.515429606	0.101835551
ANKRD18B	-0.215505575	0.26121274
ANKRD2	0.02878267	0.877091139
ANKRD20A1	0.762535816	0.281752955
ANKRD22	0.769818923	0.026749702

ANKRD23	-0.160319422	0.25431304
ANKRD27	-0.433341767	0.004981702
ANKRD28	0.281368475	0.380066483
ANKRD29	-0.855698454	0.045220534
ANKRD30BL	-0.126451428	0.463668515
ANKRD33	-0.011202989	0.935754612
ANKRD34B	0.053053519	0.790395797
ANKRD35	-0.232884078	0.23287476
ANKRD37	0.29623428	0.259077956
ANKRD39	0.477898699	0.025685469
ANKRD40	-0.406350485	0.1609015
ANKRD40CL	0.004477764	0.971953247
ANKRD42	-0.121708433	0.735783159
ANKRD44	0.678981859	0.019825246
ANKRD45	0.577294319	0.128740126
ANKRD49	0.239951774	0.1225386
ANKRD53	0.009632982	0.969720099
ANKRD54	0.265695513	0.11412816
ANKRD55	-0.30975552	0.01740717
ANKRD61	0.049572601	0.734987575
ANKRD62	-0.157120701	0.093631895
ANKRD65	0.136524584	0.409052598
ANKRD66	0.110838298	0.319817942
ANKRD7	0.100560234	0.629181947
ANKRD9	-0.353824026	0.219054412
ANKS1A	-0.135689819	0.573439645
ANKS1B	-2.032137107	0.006424462
ANKS4B	-0.042967793	0.760378549
ANKS6	0.072862956	0.77132529
ANKUB1	-0.075827425	0.6712973
ANKZF1	0.558934728	0.009154724
ANLN	-0.687434589	0.159943598
ANO1	0.230552719	0.357980789
ANO2	-0.102192399	0.620023788
ANO3	-0.887564334	0.081442916
ANO4	-1.825548705	7.88E-05
ANO5	-1.596056331	0.035157808
ANO7	-0.081846327	0.519665265
ANOS1	-0.034280611	0.930436366
ANP32A	0.555351319	0.00471239
ANP32B	0.127169439	0.672392651
ANP32D	-0.104237619	0.430567574
ANP32E	0.108002941	0.624336398
ANPEP	-0.049373132	0.944364579
ANTXR1	0.684284479	0.040668794
ANTXR2	0.374086192	0.392809192
ANXA1	2.449064983	0.008894029
ANXA10	-0.097086824	0.511049792

ANXA13	-0.13854588	0.172049116
ANXA2R	0.511047309	0.015450046
ANXA3	-1.829555673	6.64E-05
ANXA4	-0.182749302	0.566611412
ANXA5	1.303171055	0.008314096
ANXA6	-0.615417236	0.054488403
ANXA7	-0.723564041	0.00148582
ANXA9	-0.11446188	0.609173141
AOC1	-0.139932241	0.226415206
AOC2	0.159613589	0.279918882
AOC3	0.047657513	0.795866349
AOPEP	0.019821154	0.924928069
AOX1	-0.227152442	0.510790042
AP1AR	-0.167807215	0.348140956
AP1B1	-0.140376193	0.574451759
AP1G2	-1.187322322	0.011469016
AP1M2	-0.092094272	0.619223958
AP1S1	-0.094257912	0.711328813
AP1S2	0.724173855	0.098501871
AP2A1	-0.750596035	0.007782903
AP2M1	-0.46021611	0.015599671
AP2S1	-0.357647067	0.246828211
AP3B1	0.289759672	0.254105987
AP3B2	-0.787539769	0.137352678
AP3D1	0.37658438	0.093677832
AP3M2	0.214018344	0.430567574
AP3S1	-0.253241132	0.100754919
AP3S2	0.465607886	0.12708532
AP4E1	-0.059227969	0.868516953
AP4M1	0.423679803	0.082017642
AP4S1	0.168375222	0.414356468
AP5M1	-0.084708439	0.767258191
AP5S1	0.637440253	0.020490811
APAF1	0.85841479	0.005064013
APBA1	-1.156761881	0.017141009
APBA2	-0.209679673	0.536160749
APBA3	0.056952033	0.737435487
APBB1	-0.525171404	0.017222386
APBB1IP	0.961069375	0.109212762
APBB3	0.447346033	0.009646501
APC2	0.284503169	0.513684677
APCDD1	0.19467999	0.75582128
APCDD1L	-0.185649623	0.261850437
APCS	0.019161432	0.847725163
APEH	-0.138386223	0.51415788
APEX1	0.197900072	0.642440544
APEX2	0.313713561	0.290292168
APH1A	0.45917246	0.01673506

APH1B	0.444493689	0.030697987
APIP	0.206790753	0.371532601
APLF	-0.512832895	0.032425399
APLN	0.593375145	0.158088917
APLNR	0.981668261	0.131106757
APLP2	0.061397578	0.772614814
APMAP	0.162509863	0.587548898
APOA1	-0.197857215	0.202749448
APOA2	0.064313767	0.625457691
APOA4	-0.114240259	0.348517698
APOA5	0.011494404	0.950201693
APOB	-0.103215701	0.419875567
APOBEC1	0.029858629	0.760420863
APOBEC2	-0.093046095	0.319183834
APOBEC3A	-0.119221212	0.46925675
APOBEC3C	0.375753226	0.161711365
APOBEC4	-0.079550409	0.545645355
APOBR	0.182292606	0.250506267
APOC1	1.307322024	0.003962502
APOC3	-0.125078505	0.341341981
APOD	0.167998979	0.90078881
APOE	0.213990692	0.517739911
APOF	-0.033327791	0.789016468
APOH	-0.013444489	0.886535426
APOL1	0.443728545	0.040414934
APOL2	0.741409138	0.043579453
APOL4	0.36530189	0.107224994
APOL5	-0.122988087	0.233826324
APOL6	1.35614486	7.88E-05
APOLD1	0.095536717	0.746383043
APOM	0.223940986	0.049324838
APOO	-0.193933507	0.615227236
APP	-0.105677882	0.605871017
APPBP2	-0.064584158	0.815673957
APPL1	-0.033458758	0.871201192
APPL2	0.465708147	0.030260046
APRT	0.088847817	0.752177063
AQP1	1.601736389	0.002864507
AQP10	-0.050536254	0.626037333
AQP11	-0.364757481	0.033894695
AQP12A	0.238607335	0.09140524
AQP2	-0.064053313	0.62413692
AQP3	0.302241475	0.064015775
AQP4	0.851961683	0.417523719
AQP5	-0.023416038	0.941983489
AQP6	-0.001668952	0.994980306
AQP7	0.64770752	0.076949066
AQP8	0.058390553	0.695912218

AQP9	0.431301617	0.409857307
AQR	0.108673261	0.68917569
AR	1.018548103	0.001840246
ARAP2	0.033361429	0.963471701
ARAP3	1.181613762	0.000763664
ARC	0.546571303	0.294809012
ARCN1	0.12164507	0.567695781
AREG	0.272468112	0.420941497
ARF3	-0.411206374	0.019571398
ARF4	0.395642228	0.222935337
ARF5	0.260406398	0.280778748
ARF6	0.136098928	0.645165283
ARFGAP1	0.164246312	0.382513211
ARFGAP2	-0.099306239	0.573049395
ARFGAP3	0.42853192	0.119881707
ARFGEF1	0.408183261	0.011584437
ARFGEF2	-0.336571043	0.088639485
ARFGEF3	-1.248931953	0.075847919
ARFRP1	0.30417131	0.277330696
ARG1	-0.009282133	0.945980127
ARG2	-0.340561624	0.215247992
ARGFX	-0.197891439	0.243223519
ARGLU1	0.482055575	0.134065224
ARHGAP1	-0.219768569	0.374930374
ARHGAP10	-0.183125479	0.473325799
ARHGAP11A	0.908608637	0.087257026
ARHGAP12	0.022451942	0.934842248
ARHGAP15	0.39429676	0.120355934
ARHGAP18	1.155421476	0.056374918
ARHGAP20	-0.309928362	0.33514103
ARHGAP21	-0.564629915	0.089504455
ARHGAP26	-0.055617739	0.912752393
ARHGAP27	-0.139547413	0.579178647
ARHGAP29	-0.121183253	0.790568509
ARHGAP35	-0.739539796	0.004625182
ARHGAP36	-0.08811313	0.368114726
ARHGAP44	-1.326955415	0.001454069
ARHGAP45	0.564691892	0.026527015
ARHGAP6	0.512697494	0.110456596
ARHGAP9	0.176579681	0.245965752
ARHGDIB	0.566183921	0.005391307
ARHGDIG	-0.435627818	0.251493953
ARHGEF1	0.898155553	0.019703022
ARHGEF10	0.335091534	0.074275809
ARHGEF10L	0.645591289	0.020195597
ARHGEF11	0.20157011	0.405603417
ARHGEF12	-0.104915957	0.566611412
ARHGEF15	0.31055351	0.108001897

ARHGEF16	0.034195705	0.832319791
ARHGEF17	0.092105172	0.74874029
ARHGEF19	0.038552379	0.870307862
ARHGEF25	0.357744427	0.020517538
ARHGEF33	0.083550958	0.670415029
ARHGEF37	-0.342739777	0.110059166
ARHGEF39	0.051104154	0.799594134
ARHGEF4	-0.439592441	0.294586208
ARHGEF40	1.232235286	0.000430084
ARHGEF5	-0.053061964	0.691335841
ARHGEF6	1.90209412	1.78E-05
ARHGEF7	-0.166705614	0.518566194
ARID1A	0.326604366	0.065138652
ARID3A	0.189768226	0.378177004
ARID3B	0.358077186	0.080680741
ARID3C	-0.157954069	0.191008249
ARID4A	0.119748714	0.536215124
ARID4B	0.048594519	0.794683836
ARID5A	0.52658116	0.03141323
ARIH1	-0.13003741	0.427536098
ARIH2	0.546687398	0.019807497
ARL1	-0.001992742	0.992886227
ARL10	0.1819305	0.239878082
ARL11	-0.012739772	0.938195556
ARL14	-0.039797135	0.714970751
ARL14EP	0.040580881	0.866176566
ARL15	0.239601047	0.335906467
ARL2BP	0.276642419	0.257676696
ARL3	-0.70603467	0.006776741
ARL4A	0.807580747	0.015808134
ARL4C	0.784341179	0.274569019
ARL4D	-0.300808063	0.328587187
ARL5A	0.566564725	0.005714159
ARL5B	-0.184926721	0.663570856
ARL5C	0.841471268	0.073415686
ARL6	-0.234347994	0.506397029
ARL6IP1	-0.336683281	0.193824731
ARL6IP4	-0.67561044	0.009091777
ARL6IP5	-0.642433323	0.01915441
ARL6IP6	0.652564731	0.002725634
ARL8A	0.105926524	0.548307388
ARL8B	-0.470428374	0.04534134
ARL9	-0.072055623	0.651390935
ARMC1	-0.239074904	0.240957104
ARMC10	0.157130888	0.48715344
ARMC12	0.476695861	0.014785217
ARMC2	-0.040860653	0.926926533
ARMC3	0.176663762	0.67199317

ARMC5	-0.129020429	0.303461121
ARMC7	-0.142735547	0.380612457
ARMC8	0.408310369	0.015845112
ARMCX1	0.087192649	0.736206294
ARMCX2	0.31361856	0.233586232
ARMCX3	0.15603548	0.419688149
ARMCX6	0.570623009	0.019825246
ARMH1	0.319621052	0.073145543
ARMH3	-0.221120154	0.300495489
ARMT1	0.061417078	0.880787406
ARNT	0.399560928	0.021546998
ARNT2	-0.814855913	0.063240636
ARNTL	0.138020503	0.785140288
ARNTL2	-0.055278697	0.539908203
ARPC1A	0.462000545	0.057271757
ARPC2	0.042602476	0.861678768
ARPC3	0.70102221	0.000815172
ARPC5	0.198131313	0.542036039
ARPC5L	0.035339294	0.893241153
ARPIN	0.233607126	0.153156477
ARPP19	-0.651858397	0.017222386
ARPP21	-1.195131053	0.061559966
ARR3	0.035707199	0.75582128
ARRB1	-1.474251114	0.001455335
ARRB2	0.371835386	0.475703704
ARRDC1	-0.128335765	0.54615137
ARRDC3	1.473486651	0.002398463
ARRDC4	0.592932324	0.197095726
ARSA	0.02243915	0.93552248
ARSB	0.25765345	0.23287476
ARSD	0.723098458	0.06009801
ARSF	0.187972412	0.543228323
ARSG	-0.017747351	0.955471009
ARSH	-0.219032868	0.044520714
ARSJ	0.940853624	0.257447838
ARSK	0.092418941	0.773333319
ART1	0.119716749	0.380095352
ART4	-0.059497991	0.768066204
ARTN	-0.072307125	0.655803928
ARV1	-0.115769805	0.676095397
ARVCF	0.09846895	0.640818885
ARX	-0.415740934	0.248017555
ASAH1	0.379444227	0.036685772
ASAH2	0.126954974	0.355732302
ASAH2B	0.138620108	0.305375238
ASAP1	0.382516056	0.066166083
ASAP2	-0.170232039	0.584478839
ASAP3	1.092154081	0.000739219

ASB1	-0.329950669	0.061615095
ASB12	-0.095782806	0.327370792
ASB15	-0.13203767	0.090678601
ASB16	-0.180973814	0.281151723
ASB17	-0.023163777	0.820667679
ASB18	-0.146892267	0.270926222
ASB4	-0.183627537	0.093764507
ASB5	-0.043931599	0.732117756
ASB6	0.063697861	0.836438676
ASB7	0.416580196	0.067292748
ASB8	-0.179689662	0.531892735
ASB9	0.209988454	0.287977302
ASCC2	0.511993987	0.044473642
ASCC3	0.419183159	0.275982322
ASCL1	0.700309019	0.435455714
ASCL2	0.018629133	0.926890103
ASCL3	-0.078318606	0.395816592
ASCL4	-0.125160796	0.316483083
ASF1A	0.097595331	0.782828794
ASF1B	0.133576151	0.603610614
ASGR1	0.144421608	0.516421193
ASGR2	-0.070577712	0.698341604
ASH1L	-0.014173945	0.945534243
ASIC1	-0.027594896	0.957700639
ASIC2	-0.978425187	0.006289926
ASIC3	0.369405018	0.230743036
ASIC4	0.515581566	0.232654739
ASIC5	-0.093138701	0.234947653
ASIP	0.159651827	0.396629221
ASL	0.207894256	0.494835237
ASMT	0.153905663	0.47142387
ASMTL	0.043888208	0.845379815
ASNS	0.352469419	0.105858884
ASNSD1	0.264515419	0.234706512
ASPA	-1.380213274	0.06041
ASPH	-0.000373831	0.998986205
ASPHD1	-1.062650244	0.002363145
ASPHD2	-0.399273457	0.188151106
ASPM	1.179385559	0.054474658
ASPN	1.103932558	0.04438638
ASPRV1	0.26308485	0.155448076
ASS1	-0.672566931	0.199371809
ASTE1	0.125580516	0.501702656
ASTN1	-0.284484753	0.670085809
ASXL1	0.249908567	0.136957726
ASXL2	0.440602708	0.004830214
ASZ1	-0.089269908	0.174406672
ATAD1	-0.461896671	0.0169614

ATAD2	0.876124645	0.009150935
ATAD3A	0.020967942	0.948418763
ATAD5	1.10079717	0.007519601
ATCAY	-1.041586211	0.289876417
ATE1	-0.665749353	0.008396018
ATF4	0.101867331	0.645126174
ATF6	0.11843592	0.429379985
ATF6B	0.248785846	0.421738538
ATF7IP	0.748294208	0.007082753
ATF7IP2	-0.762573717	0.039616406
ATG10	0.355389236	0.035747902
ATG101	0.001763676	0.993945285
ATG16L1	0.258727521	0.163816455
ATG2B	-0.172400979	0.48504842
ATG3	0.28043499	0.1546531
ATG4A	0.068051761	0.883063921
ATG4B	0.46234983	0.034018658
ATG4C	0.278232527	0.499335429
ATG4D	-0.084637468	0.646395303
ATG5	0.189446594	0.392266258
ATG7	0.387706612	0.023262209
ATG9A	-0.508501449	0.003250746
ATIC	-0.038033251	0.855304945
ATL1	-1.190301973	0.010749576
ATL2	0.077288401	0.696468813
ATL3	0.514650717	0.063950041
ATM	0.792260312	0.000978538
ATOH1	-0.283893668	0.070843893
ATOH7	-0.330598668	0.007042821
ATOH8	-0.149690194	0.560024501
ATOX1	0.199454621	0.263766329
ATP10A	-0.778790344	0.007327295
ATP10D	0.822141897	0.023244711
ATP11A	0.398883397	0.193911699
ATP11C	0.624793474	0.132445969
ATP13A1	0.188118352	0.243804426
ATP13A2	-0.92206576	0.00400499
ATP13A3	0.441697776	0.173390717
ATP13A4	1.172418837	0.198897866
ATP13A5	0.58387933	0.140000239
ATP1A1	-0.317475316	0.248017555
ATP1A2	-0.859105272	0.382513211
ATP1A4	0.108490332	0.445186396
ATP1B1	-1.551256427	0.013504955
ATP1B2	0.401241986	0.678628983
ATP1B3	0.238796261	0.377647719
ATP23	0.12752542	0.592604121
ATP2A1	0.205684651	0.296280307

ATP2A2	-0.389953727	0.060908216
ATP2A3	0.174099043	0.320884214
ATP2B1	-0.885762137	0.075727858
ATP2B2	-1.765331957	0.025279593
ATP2B4	-0.06023413	0.879067476
ATP2C2	-0.319639278	0.030492101
ATP4A	-0.034913692	0.792979636
ATP4B	-0.211661356	0.139569359
ATP5F1A	-0.36274488	0.041553053
ATP5F1B	-0.672963294	0.025920214
ATP5F1C	-0.627914405	0.013465777
ATP5F1D	-0.805517978	0.011584437
ATP5F1E	0.285656704	0.184486834
ATP5IF1	-0.104941651	0.519070387
ATP5MC1	-0.484191873	0.039390494
ATP5MC2	0.145096834	0.494792618
ATP5ME	-0.302198205	0.136957726
ATP5MF	0.162173824	0.28898109
ATP5PB	-0.107490635	0.35811902
ATP5PD	-0.18684058	0.357808768
ATP5PF	-0.171935311	0.236528287
ATP5PO	-0.221143146	0.23210837
ATP6AP1	-0.114003193	0.517738935
ATP6AP2	0.202868723	0.318175113
ATP6V0A2	0.407045539	0.159226084
ATP6V0A4	-0.349925481	0.026485864
ATP6V0B	0.190074461	0.151947457
ATP6V0D1	-0.658814639	0.005093563
ATP6V0D2	0.075363865	0.568613411
ATP6V0E1	0.337828398	0.281291951
ATP6V0E2	0.029373143	0.924749002
ATP6V1A	-0.580260863	0.101983839
ATP6V1B1	0.500233266	0.059892155
ATP6V1B2	-0.519737599	0.100281092
ATP6V1C1	-0.708833145	0.066648169
ATP6V1D	-0.487643084	0.029475105
ATP6V1E1	-0.792703852	0.00126448
ATP6V1E2	0.129268781	0.427259491
ATP6V1G1	0.170956291	0.239029191
ATP6V1G2	0.861523201	0.000331368
ATP6V1G3	-0.057574695	0.582839574
ATP6V1H	-0.876088243	0.006281327
ATP7B	-0.237303989	0.394388931
ATP8A1	-1.564807382	0.049169446
ATP8A2	-2.603819389	0.00097933
ATP8B2	-0.001670838	0.995376964
ATP8B4	0.452461413	0.056016262
ATP9A	-1.07252808	0.015140509

ATPAF1	-0.560786222	0.036926773
ATPAF2	0.03909892	0.813769537
ATR	0.568643628	0.004369286
ATRAID	0.489587553	0.010607259
ATRIP	0.348468927	0.007391046
ATRN	-0.297787276	0.181459881
ATRNL1	-2.114382385	0.038599609
ATRX	0.09630215	0.539048753
ATXN1	-0.05907304	0.801083515
ATXN10	-0.077351483	0.63825878
ATXN2L	0.273195228	0.159863387
ATXN7L1	0.450272893	0.08303727
ATXN7L2	0.085224274	0.583914788
AUH	0.156171355	0.414462192
AUNIP	-0.084651378	0.481392472
AUP1	0.480021946	0.003814444
AURKA	0.265998443	0.312543787
AURKAIP1	-0.44259091	0.008236245
AURKB	0.816718592	0.120693464
AUTS2	0.498374328	0.149414778
AVEN	-0.153597893	0.668259657
AVIL	1.024534415	0.000123454
AVL9	0.379160276	0.056043915
AVP	-0.34170203	0.146280654
AVPI1	-0.853604268	0.000126605
AVPR1A	-0.013278802	0.954935141
AVPR1B	0.153773758	0.107546114
AWAT2	0.046619209	0.65734449
AXDND1	0.10767226	0.278913814
AXIN1	0.022805299	0.913475369
AXIN2	-0.215229247	0.34556292
AXL	-0.721572181	0.127365146
AZGP1	-0.101631341	0.688187591
AZI2	-0.056560296	0.752177063
AZIN1	-0.097972665	0.622639969
AZIN2	-0.06019942	0.873377072
AZU1	0.198760362	0.193712225
B2M	1.255766442	0.000389052
B3GALT1	-0.134862567	0.827153531
B3GALT2	-0.724186851	0.394842499
B3GALT4	-0.001537857	0.992886227
B3GALT5	0.378968201	0.102949081
B3GALT6	0.241387525	0.283753608
B3GAT1	-0.154374112	0.801253954
B3GAT2	-0.09260815	0.84159081
B3GAT3	0.271076595	0.293840704
B3GLCT	0.323191318	0.363326014
B3GNT2	0.091442557	0.774103978

B3GNT3	-0.038302959	0.7879778
B3GNT4	-0.426648637	0.047203268
B3GNT5	0.077279543	0.74315299
B3GNT6	-0.125227365	0.344525276
B3GNT7	-0.213224896	0.203783861
B3GNT8	-0.231500391	0.053209835
B3GNT9	0.597982594	0.076346416
B4GALNT1	-0.561928726	0.296827688
B4GALNT2	-0.136542147	0.28224197
B4GALNT3	-0.371827825	0.065392631
B4GALNT4	-0.064015491	0.861298403
B4GALT1	-0.051052572	0.866575918
B4GALT2	-0.336665409	0.021555184
B4GALT4	0.305896732	0.056271341
B4GALT5	0.70483725	0.081940615
B4GALT6	-1.322234021	0.076935303
B4GALT7	0.131051702	0.493772117
B4GAT1	-0.762475589	0.034098511
B9D2	-0.083276319	0.473389208
BAALC	0.735147354	0.059856299
BAAT	-0.426921582	0.016817883
BABAM2	0.151342357	0.288697544
BACE1	-0.31588926	0.262592402
BACE2	0.180193749	0.677826901
BACH1	0.269130054	0.160059954
BACH2	0.525247454	0.214039591
BAG1	-0.19636017	0.317915289
BAG2	-0.026529366	0.938370209
BAG3	-0.456552241	0.177287889
BAG4	-0.478919206	0.011850098
BAG6	-0.037440686	0.819704109
BAHCC1	0.023765417	0.931748888
BAHD1	-0.13268109	0.51552441
BAIAP2	-0.471339609	0.147149741
BAIAP2L1	-0.508789387	0.2400032
BAIAP2L2	-0.127718446	0.406099532
BAIAP3	-0.57500666	0.181766132
BAMBI	0.03152474	0.956242346
BANF1	0.069339583	0.736206294
BANF2	-0.164530729	0.116756442
BANK1	-0.150470468	0.240167593
BAP1	-0.454294371	0.052514998
BARD1	1.162714425	0.001392079
BARHL1	0.074649408	0.713113471
BARHL2	-0.135796342	0.22962459
BARX1	-0.174179652	0.257228425
BARX2	-0.26115482	0.07570597
BASP1	-2.282676354	0.004625182

BATF	0.077559707	0.538975565
BATF2	0.303470005	0.056043915
BATF3	0.538241419	0.121145569
BAX	0.418443313	0.331569694
BAZ1A	1.094262416	0.027036384
BAZ1B	0.630145239	0.018768941
BAZ2A	0.595922982	0.002621142
BAZ2B	0.30052839	0.365308672
BBOF1	0.137262629	0.42817647
BBOX1	1.222187893	0.0133765
BBS1	-0.072924324	0.75726135
BBS10	0.043524986	0.911351241
BBS2	0.448608913	0.259555334
BBS5	0.07945388	0.75455024
BCAN	1.479351383	0.05273051
BCAS1	-1.2700082	0.08103115
BCAS4	-0.6115978	0.005837491
BCAT1	0.72972347	0.207142254
BCCIP	-0.142704394	0.615580788
BCDIN3D	0.152876296	0.351918897
BCHE	1.139771927	0.008092617
BCKDHA	-0.327736755	0.142159077
BCKDHB	0.397824409	0.135445699
BCKDK	-0.047822052	0.853607455
BCL10	-0.060928763	0.821868934
BCL11A	-0.95634095	0.010685448
BCL11B	-0.175032257	0.316770683
BCL2	0.343071335	0.416336033
BCL2L1	-0.25463779	0.274104176
BCL2L10	-0.094554559	0.315884113
BCL2L12	0.018774837	0.943084607
BCL2L13	-0.236269961	0.382513211
BCL2L14	-0.024960551	0.824651719
BCL3	0.294894177	0.491010445
BCL6	0.992175134	0.007640845
BCL6B	0.713472582	0.05875883
BCL7A	-0.364845016	0.278161431
BCL7C	-0.08044135	0.751015759
BCL9	0.392460088	0.093377863
BCL9L	0.158368029	0.583881916
BCLAF1	0.221284644	0.090678601
BCLAF3	0.253247293	0.094233344
BCO1	0.073516655	0.74761503
BCO2	0.224357976	0.437000037
BCOR	-0.046687072	0.872097725
BCORL1	0.27943886	0.235863309
BCR	-0.243073813	0.408664247
BCS1L	0.684576328	0.003446888

BDH1	-0.583204828	0.103117374
BDH2	0.362219898	0.268388157
BDKRB1	-0.118803287	0.473389208
BDNF	-0.709961336	0.004996766
BDP1	-0.104761841	0.621989233
BECN1	0.154587635	0.327635999
BEND4	-0.415619303	0.159583742
BEND5	0.164861546	0.577372047
BEND6	-0.874281218	0.016465835
BEST1	-0.614512558	0.21462842
BEST2	-0.224384508	0.059949188
BEST3	1.423074776	0.035400588
BEST4	0.185665744	0.436014625
BET1	0.281552585	0.055086894
BET1L	-0.004085362	0.990996918
BEX1	-1.170614661	0.091598211
BEX3	-0.322815478	0.106075036
BEX5	-0.954896981	0.01622654
BFAR	0.259696518	0.46525882
BFSP2	-0.131904199	0.302708198
BGN	1.022833065	0.192216241
BHLHA15	0.092722152	0.626602907
BHLHA9	-0.382208098	0.019768736
BHLHE22	-0.097103876	0.583914788
BHLHE40	0.111206857	0.792979636
BHLHE41	0.2343233	0.700771547
BHMT	-0.211235859	0.058078298
BHMT2	-0.195451676	0.566497648
BICC1	-0.960701058	0.141254251
BICD1	0.977472155	0.001613537
BICD2	0.176618344	0.404937034
BICDL1	-0.56107712	0.034143362
BICRA	0.161391791	0.516421193
BID	-0.183849359	0.351099922
BIK	0.120022509	0.23670507
BIN1	-1.203847195	0.017128356
BIN2	1.010765148	0.032425399
BIN3	0.271406674	0.107507557
BIRC2	0.457759963	0.044156837
BIRC3	0.492334782	0.171793831
BIRC6	0.277312186	0.030168363
BIRC7	0.158550871	0.448578148
BLK	-0.005793199	0.956825161
BLM	1.552018674	0.000973474
BLMH	0.716592168	0.008044688
BLNK	0.990277371	0.045585085
BLOC1S3	-0.14169109	0.396108842
BLOC1S4	0.003339857	0.982765512

BLOC1S6	-0.128188496	0.597800651
BLVRA	0.636909072	0.007496002
BLVRB	-0.12229993	0.623438155
BLZF1	0.168759203	0.217419138
BMERB1	-0.474621565	0.137948025
BMF	0.209967446	0.145336887
BMP1	1.332584928	0.007676985
BMP10	-0.053985649	0.652822208
BMP15	-0.145390088	0.282459455
BMP2	0.509304013	0.38617935
BMP2K	0.286570969	0.271147368
BMP3	-0.086106914	0.447828731
BMP4	-0.422986173	0.3871543
BMP5	0.193886066	0.602738563
BMP6	-0.533534454	0.20687496
BMP7	0.565344117	0.114941533
BMP8A	-0.002562077	0.994849586
BMP8B	0.067465975	0.868600923
BMPER	-0.170846757	0.737832072
BMPR1A	0.031094714	0.932373222
BMPR1B	0.211310911	0.574759224
BMPR2	-0.389709236	0.185997113
BMS1	-0.067186392	0.82820301
BMT2	0.187421907	0.521703775
BMX	-0.156360239	0.041222772
BNC1	-0.13898904	0.190374885
BNC2	0.217041274	0.717706165
BNIP1	0.541353198	0.005755113
BNIP2	0.395802757	0.05613251
BNIP3	-0.396110393	0.323541329
BNIP3L	-0.111144044	0.552503918
BNIP5	-0.123251262	0.204128546
BOC	1.079470489	0.005920421
BOD1	0.638270915	0.000763434
BOD1L1	0.179154402	0.318175113
BOK	-1.927692273	0.00010541
BOLA1	0.677237323	0.026907792
BOLA3	-0.373185302	0.051632466
BOLL	-0.022575431	0.882145778
BORA	0.666075986	0.029826735
BORCS5	-0.237641122	0.287977302
BORCS6	-0.221768796	0.247249456
BPGM	0.130870816	0.523014367
BPI	0.040894005	0.755392658
BPIFA1	-0.067125922	0.420536191
BPIFA2	-0.143168341	0.31791568
BPIFA3	-0.122159877	0.299604752
BPIFB1	-0.091375872	0.257149272

BPIFB2	-0.092070795	0.4714811
BPIFB3	0.04275817	0.726883295
BPIFB4	-0.169989378	0.103255505
BPIFB6	-0.086194168	0.358816041
BPIFC	-0.056513549	0.581403145
BPNT1	0.175352294	0.385784865
BPTF	0.069457997	0.792324241
BPY2	-0.158339827	0.105046429
BRAP	0.198195161	0.243985433
BRAT1	0.504228468	0.002518283
BRCA1	0.963071231	0.002267206
BRCA2	1.130871774	0.017088581
BRCC3	-0.219713685	0.203596982
BRD1	0.196459074	0.268131236
BRD2	0.463340544	0.025596149
BRD3	0.351855528	0.078614655
BRD4	0.151502107	0.565244705
BRD8	0.393112228	0.034247788
BRDT	0.019123326	0.857392501
BRF2	-0.141586746	0.223823481
BRI3	0.117194173	0.534209384
BRI3BP	-0.520269166	0.006777787
BRICD5	-0.159326298	0.46591371
BRINP1	-1.739493956	0.014591133
BRINP2	-0.840991544	0.192559557
BRINP3	-0.317549597	0.811503426
BRIP1	1.125765067	0.091410569
BRIX1	0.334481077	0.12091543
BRK1	-0.177558686	0.449789427
BRMS1	0.330696834	0.068369928
BRMS1L	-0.075443244	0.770820326
BROX	0.357558985	0.051442184
BRPF1	0.145740374	0.522781348
BRS3	-0.012331473	0.933014136
BRSK1	-1.119700454	0.012715052
BRWD1	0.057136788	0.833863145
BRWD3	0.460682739	0.122433204
BSG	-0.352794381	0.032640805
BSN	-1.383506023	0.013273238
BSND	0.000802585	0.995953997
BSPRY	-0.05716891	0.726469436
BST1	-0.166472004	0.794214198
BST2	1.692189875	0.003414684
BSX	-0.138734866	0.409857307
BTAF1	0.174754554	0.401628644
BTBD1	-0.130208228	0.613196297
BTBD10	-0.885469217	0.000664132
BTBD16	-0.165783059	0.147253077

BTBD2	-0.637587101	0.008046009
BTBD3	-0.091487399	0.777461665
BTBD6	-0.431100455	0.05273051
BTBD8	-0.250583093	0.540839301
BTC	-0.215702206	0.053575397
BTD	-0.051042324	0.695647216
BTG1	0.781351317	0.001899959
BTG2	0.566682429	0.181129091
BTG4	-0.005595188	0.967748239
BTK	0.822963387	0.001454069
BTLA	0.022559996	0.86502926
BTN1A1	-0.114910493	0.362278328
BTN2A1	0.280329225	0.178134407
BTN3A1	0.819703653	0.006974901
BTN3A3	1.106582358	0.001781018
BTNL2	0.117932109	0.325516191
BTNL3	0.137526666	0.278481075
BTNL9	-0.054232533	0.780766159
BTRC	-0.596365241	0.047832669
BUB1	1.0884563	0.06539208
BUB1B	0.078019385	0.752870608
BUB3	0.174614735	0.560024501
BUD13	0.731127459	0.000690887
BUD31	0.907744892	0.00048008
BVES	-0.166232376	0.807710733
BYSL	0.453271897	0.082570416
C10ORF53	0.078436227	0.566611412
C10ORF67	-0.078037802	0.420462542
C10ORF82	-0.381199396	0.002245834
C10ORF88	-0.469385273	0.022935772
C10ORF95	-0.084888748	0.447783257
C10ORF99	0.111184332	0.394409696
C11ORF1	-0.228272052	0.308749255
C11ORF16	-0.002977338	0.988671261
C11ORF24	0.090716195	0.772614814
C11ORF42	-0.189063226	0.113921677
C11ORF45	0.037002029	0.762526857
C11ORF49	0.14874309	0.409056709
C11ORF53	-0.105959313	0.319064938
C11ORF54	0.489400115	0.06529065
C11ORF58	0.300801074	0.059797454
C11ORF65	0.133897696	0.268987728
C11ORF68	-0.474245528	0.008556158
C11ORF80	0.297939349	0.147176235
C11ORF86	-0.152596454	0.328241944
C11ORF87	-2.028171989	0.003357392
C11ORF94	0.054010694	0.556261656
C11ORF96	0.194745575	0.164313763

C12ORF29	-0.39217573	0.205716635
C12ORF4	0.476869896	0.026700523
C12ORF40	-0.050500428	0.581110545
C12ORF42	-0.179356104	0.016278382
C12ORF45	0.10152105	0.601656239
C12ORF50	-0.040268824	0.647755964
C12ORF57	0.304862214	0.221632639
C12ORF60	0.040826186	0.733454552
C12ORF66	0.043089716	0.883530891
C12ORF71	-0.003009437	0.986895085
C12ORF73	0.129327843	0.564742908
C12ORF76	0.029216874	0.922792657
C14ORF119	-0.129944183	0.668259657
C14ORF132	-0.908467531	0.056043915
C14ORF180	-0.147864323	0.40003876
C14ORF28	0.02353889	0.873311527
C14ORF39	0.180979495	0.663942355
C14ORF93	0.379170186	0.030822445
C15ORF39	0.433729643	0.032607802
C15ORF48	-0.048261162	0.639605565
C15ORF61	-0.338570484	0.109152341
C15ORF62	-0.133658429	0.300109754
C16ORF54	-0.128284472	0.327370792
C16ORF70	-0.389186382	0.05679073
C16ORF71	0.107935899	0.542314727
C16ORF72	0.07009114	0.803401414
C16ORF74	-0.06390145	0.670085809
C16ORF78	-0.140973596	0.22644373
C16ORF86	-0.122394612	0.510796592
C16ORF87	0.34451383	0.300029437
C16ORF89	-0.516818771	0.112654344
C16ORF90	0.20882004	0.110253544
C16ORF92	0.099820557	0.336886789
C16ORF95	-0.051624652	0.824610383
C16ORF96	-0.047582986	0.679671403
C17ORF107	-0.325716267	0.092843122
C17ORF58	0.165744887	0.435114027
C17ORF64	0.056162745	0.600215209
C17ORF75	-0.084446826	0.503473628
C17ORF80	0.394561094	0.040589423
C17ORF97	-0.158281089	0.669900902
C17ORF99	0.009545456	0.945534243
C18ORF54	1.229994014	0.009687733
C19ORF18	0.567699782	0.006398452
C19ORF25	0.13088389	0.588137206
C19ORF33	-1.116399792	0.000974395
C19ORF38	0.543253248	0.023128286
C19ORF44	0.603281453	0.002174066

C19ORF47	0.114383198	0.487841801
C19ORF53	0.136781283	0.407947913
C19ORF54	0.747254961	0.028932489
C19ORF67	-0.017698915	0.905804043
C19ORF71	-0.192677141	0.385767435
C19ORF73	-0.062494201	0.700015813
C1GALT1	-0.117099243	0.584768971
C1GALT1C1	0.26924898	0.124561843
C1ORF100	-0.182548142	0.039963264
C1ORF105	-0.012200854	0.945534243
C1ORF109	0.332911861	0.0240514
C1ORF115	-1.418376781	0.011495632
C1ORF116	0.04722283	0.763905761
C1ORF122	-0.404104292	0.017353105
C1ORF127	-0.057364515	0.587701111
C1ORF131	0.657269836	0.002341058
C1ORF141	-0.098951571	0.326060709
C1ORF146	-0.083365477	0.326633542
C1ORF159	0.113533644	0.374162971
C1ORF162	1.335198574	0.00340473
C1ORF167	-0.049943038	0.623080098
C1ORF174	0.042099185	0.874929281
C1ORF185	0.068819703	0.757117728
C1ORF194	0.698407629	0.099064074
C1ORF198	-0.998579954	0.005726112
C1ORF21	0.622049142	0.104500218
C1ORF210	-0.096298921	0.37308864
C1ORF216	0.02819032	0.906358055
C1ORF226	0.505108176	0.070488907
C1ORF35	0.03669672	0.761652338
C1ORF50	-0.007170377	0.971953247
C1ORF53	-0.120107733	0.514120748
C1ORF54	0.52741988	0.200132558
C1ORF56	-0.150720553	0.515432943
C1ORF74	0.04813108	0.691335841
C1ORF87	0.131284628	0.563322948
C1QA	1.3095348	0.003510116
C1QB	1.284063142	0.009598887
C1QBP	0.141455174	0.679193729
C1QC	1.668335182	0.009675087
C1QL1	0.3989826	0.350979442
C1QL2	-1.302789539	0.000992635
C1QTNF1	1.013429428	0.005461476
C1QTNF2	-0.127769037	0.320910782
C1QTNF4	-0.945087645	0.033319952
C1QTNF5	-0.033291418	0.820606478
C1QTNF6	0.215499663	0.21213973
C1QTNF8	-0.147478239	0.227889709

C1QTNF9	0.597134431	0.007298192
C1QTNF9B	-0.139152045	0.169782421
C1R	1.684244338	0.001858755
C1RL	0.859053436	0.013311097
C1S	1.722669606	0.001554747
C2	0.900038764	0.005616699
C20ORF141	-0.46886607	0.024436271
C20ORF144	0.181732064	0.489334542
C20ORF203	-0.048582549	0.726378259
C20ORF85	-0.092823266	0.373425037
C20ORF96	0.820262353	0.012129056
C21ORF58	0.241624486	0.234259446
C21ORF91	-0.438236007	0.110456596
C22ORF15	0.173276679	0.248754651
C22ORF23	-0.125034824	0.327121235
C22ORF31	-0.022964331	0.826878373
C22ORF39	0.141512535	0.37636203
C22ORF42	0.058714035	0.582904019
C2CD2	0.142043114	0.519213736
C2CD2L	-0.566186434	0.012594619
C2CD3	0.280273753	0.072637354
C2CD4A	-0.073850419	0.725073947
C2ORF15	-0.191723435	0.335797568
C2ORF16	0.530509253	0.015278861
C2ORF42	0.18131834	0.250506267
C2ORF49	0.101175617	0.481637738
C2ORF50	0.060978711	0.792479756
C2ORF66	0.480153656	0.066033801
C2ORF69	-0.295034043	0.04037969
C2ORF73	0.121354466	0.518818023
C2ORF74	0.148324159	0.639693527
C2ORF78	-0.147764286	0.158419904
C2ORF80	-0.087904827	0.90145729
C2ORF81	0.015995001	0.906262768
C3	2.04212503	0.031483651
C3AR1	0.323435165	0.210747035
C3ORF14	-0.576675788	0.075450299
C3ORF18	-0.0502355	0.795866349
C3ORF20	0.05944062	0.661783029
C3ORF22	0.010035657	0.954613647
C3ORF33	-0.086650009	0.548219322
C3ORF38	0.216621595	0.196360909
C3ORF49	-0.055759347	0.620696342
C3ORF52	-0.405762455	0.157509338
C3ORF80	-1.295968982	0.003563462
C3ORF84	-0.454049091	0.02100609
C4B	1.976353717	0.004726947
C4BPA	-0.018625259	0.86196737

C4BPB	-0.111708085	0.20003068
C4ORF17	0.040917101	0.726264211
C4ORF19	0.242315999	0.345104196
C4ORF33	0.020549677	0.904792329
C4ORF36	0.041825876	0.646942576
C4ORF45	-0.075862514	0.447940525
C4ORF46	0.155262754	0.572746938
C4ORF51	-0.118935454	0.217503543
C4ORF54	-0.091687165	0.588577878
C5	0.999983754	0.011372074
C5AR2	0.31242159	0.115779802
C5ORF15	0.578395666	0.023787801
C5ORF22	-0.378450659	0.125809145
C5ORF24	0.234781375	0.257298754
C5ORF34	0.380833477	0.079513979
C5ORF46	-0.585431067	0.114505221
C5ORF47	0.065187474	0.548610755
C5ORF49	-0.036345688	0.907973851
C5ORF51	-0.031386976	0.893831905
C5ORF52	-0.14088469	0.101936547
C5ORF58	-0.14231019	0.096275283
C5ORF63	0.593213757	0.049970996
C6ORF118	1.032230244	0.057106477
C6ORF141	0.410849106	0.36134822
C6ORF15	-0.088169291	0.730487879
C6ORF163	0.444869235	0.027165261
C6ORF226	-0.109230747	0.399340316
C6ORF62	0.435221851	0.012458369
C6ORF89	0.087924817	0.614139893
C7	0.45960523	0.256994183
C7ORF25	0.082494662	0.536160749
C7ORF26	0.403500597	0.011765389
C7ORF31	0.335168917	0.168506214
C7ORF33	-0.029166677	0.830359479
C7ORF50	-0.074291687	0.696930692
C7ORF57	0.536379164	0.235044258
C8A	-0.180106551	0.0883666
C8B	-0.091290112	0.281151723
C8G	-0.018197551	0.886144927
C8ORF33	0.330074373	0.014596147
C8ORF34	0.14644185	0.700121086
C8ORF37	0.349579418	0.175487696
C8ORF48	-0.016328977	0.962868049
C8ORF58	-0.000674234	0.998317175
C8ORF74	-0.094453704	0.547199227
C9	-0.064753425	0.650417176
C9ORF116	0.056357938	0.815040799
C9ORF131	-0.04460685	0.621871061

C9ORF135	0.029357799	0.773364838
C9ORF152	-0.040334371	0.772253466
C9ORF16	-0.387483017	0.083842292
C9ORF24	-0.367523173	0.042585017
C9ORF40	-0.151695009	0.303928246
C9ORF43	0.22352315	0.125729285
C9ORF50	0.205645893	0.041773903
C9ORF57	-0.103163952	0.271825342
C9ORF64	0.340691763	0.2572243
C9ORF78	0.141126938	0.299614407
C9ORF85	0.022030686	0.909233143
CA11	-2.336983233	0.00324239
CA12	0.93234285	0.114022013
CA13	0.979505988	0.01413174
CA14	-0.046703	0.927569725
CA2	0.313459776	0.609305808
CA4	-0.896822509	0.007810854
CA5A	0.091440804	0.590885658
CA5B	0.491817701	0.117891319
CA6	-0.029824044	0.776484731
CA8	0.215012078	0.497719676
CA9	0.521866954	0.155226851
CAB39L	-0.432067975	0.184777407
CABCOCO1	-0.306118896	0.360432501
CABLES1	-1.90254212	0.002150162
CABP1	-2.583632007	0.000653805
CABP2	-0.15807933	0.325980769
CABP4	0.08900678	0.663753666
CABP5	0.051610344	0.837559998
CABP7	0.040210339	0.90123799
CABS1	-0.104443978	0.303222355
CABYR	-0.784232441	0.008548221
CACHD1	0.46494516	0.115986257
CACNA1B	-2.184934364	0.001454069
CACNA1D	-0.216952351	0.719125255
CACNA1F	0.057105922	0.680402738
CACNA1G	-0.464669591	0.116353381
CACNA1H	-0.252556875	0.458902888
CACNA1S	-0.173865645	0.081898324
CACNA2D1	-1.584875908	0.005678092
CACNA2D3	-2.196244148	0.001192251
CACNA2D4	0.19020427	0.217900828
CACNB1	-0.912911487	0.046208779
CACNB3	-0.814869845	0.001768875
CACNG1	0.01116718	0.951240846
CACNG2	-2.060237075	0.013337633
CACNG3	-3.06332254	0.002175978
CACNG4	1.372744378	0.067342819

CACNG5	-0.17439178	0.340627648
CACNG6	-0.03436535	0.764723531
CACNG7	-0.765016216	0.208888586
CACNG8	-1.07853321	0.009085927
CACUL1	-0.311178143	0.071693499
CACYBP	-0.227327964	0.245965752
CAD	0.630124135	0.037871129
CADM1	0.196527863	0.578328949
CADM4	0.068383526	0.8746041
CADPS	-0.471549093	0.612180181
CADPS2	-1.068946663	0.102296257
CALB1	-1.20204588	0.100617837
CALB2	-0.649208977	0.396038142
CALCA	-0.046986331	0.861806384
CALCB	-0.167218395	0.161329673
CALCOCO1	-0.061741103	0.776457433
CALCRL	1.746003038	0.007553782
CALHM1	0.0115063	0.941168506
CALHM2	0.066719995	0.787690301
CALHM5	-0.206310551	0.749153568
CALM1	-0.206481604	0.583881916
CALM2	-0.067461016	0.782191352
CALM3	-0.875822662	0.002730929
CALML3	-0.331723482	0.032059339
CALML4	0.239910187	0.291933969
CALML5	0.01940857	0.891101631
CALML6	0.069967987	0.678164991
CALN1	-0.700624953	0.009688551
CALR	0.501171578	0.111193016
CALR3	-0.122121421	0.178414048
CALY	-0.742498351	0.008882389
CAMK1	-0.217630498	0.222869023
CAMK1D	-1.347702348	0.001174699
CAMK1G	-2.243592983	0.007419913
CAMK2A	-3.03640317	0.007894632
CAMK2B	-1.206218302	0.041708728
CAMK2G	-1.349536421	0.004019472
CAMK2N1	-1.093975897	0.023156918
CAMK2N2	-0.745577391	0.060489715
CAMK4	-1.793580177	0.006185744
CAMKK1	-1.200276092	0.006496089
CAMKK2	-1.314621181	0.002565396
CAMKMT	0.392645045	0.049314519
CAMKV	-1.851185587	0.009984808
CAMLG	-0.085623786	0.813178505
CAMP	0.005197023	0.963152726
CAMSAP1	0.067287323	0.764892467
CAMSAP2	-0.133497496	0.670750072

CAND1	0.086192626	0.693365203
CANT1	0.192631783	0.557208115
CANX	0.421344394	0.009360924
CAP1	-0.224335066	0.399038792
CAP2	-1.388786386	0.031141515
CAPG	0.35243858	0.154259025
CAPN1	-0.146805674	0.519463261
CAPN10	0.395277197	0.040433822
CAPN11	0.055957809	0.65968606
CAPN12	-0.180563065	0.163432372
CAPN13	-0.296185202	0.024894263
CAPN15	0.135097107	0.526787405
CAPN2	0.28795332	0.37636203
CAPN3	1.410267649	0.001369358
CAPN5	0.988304	0.000950848
CAPN6	0.06357485	0.666171783
CAPN7	0.64478737	0.006138731
CAPN9	-0.195720243	0.086478312
CAPNS1	-0.382032188	0.123801057
CAPRIN1	0.147707755	0.383328795
CAPRIN2	-0.094530628	0.752680135
CAPS2	-0.134368927	0.72028673
CAPSL	0.079609039	0.323217852
CAPZA1	0.208751896	0.28988499
CAPZA2	0.450958181	0.010454593
CAPZA3	-0.103041213	0.316898088
CAPZB	0.252026142	0.137134412
CARD10	0.00711683	0.963336619
CARD11	0.090189813	0.709670769
CARD14	-0.223972304	0.076453699
CARD18	0.01970851	0.876312032
CARD19	0.209179996	0.429798231
CARD6	0.269538152	0.394311101
CARD9	0.075213419	0.718701933
CARF	0.029332315	0.915143203
CARHSP1	-0.214200137	0.573966322
CARM1	-0.265774001	0.23805142
CARMIL3	-0.086019917	0.736206294
CARNMT1	-0.118136449	0.659647977
CARNS1	-0.972210008	0.009991617
CARS2	0.320088627	0.023787801
CARTPT	-0.831986388	0.014596147
CASC3	0.005881975	0.981452955
CASK	0.876985897	0.008051567
CASKIN1	-1.476845038	0.005514293
CASKIN2	0.063050896	0.830859796
CASP10	0.253858417	0.084103452
CASP14	0.049032745	0.740003676

CASP2	1.041511003	0.002725634
CASP3	0.998952214	0.105971251
CASP4	0.783291698	0.005980982
CASP5	0.10405698	0.364294487
CASP7	0.131578351	0.635671996
CASP8	0.337782045	0.137008538
CASP8AP2	0.388244573	0.007810854
CASP9	0.336479723	0.284849449
CASQ1	0.095809047	0.84451194
CASQ2	-0.184678434	0.153046427
CAT	-0.071880728	0.789069634
CATIP	0.135061492	0.43381273
CATSPER1	-0.326561618	0.031701597
CATSPER2	-0.394431556	0.304245061
CATSPER3	0.208306494	0.181766132
CATSPER4	-0.057679776	0.497563369
CATSPERB	0.073119983	0.918837703
CATSPERD	-0.234907277	0.027516253
CATSPERE	0.104668753	0.37549496
CATSPERG	-0.004472605	0.985975899
CAV3	-0.031670818	0.866152697
CAVIN1	0.166914626	0.857443332
CAVIN2	-1.095053792	0.016278382
CAVIN3	-0.085266845	0.800572098
CBARP	0.221754137	0.389908188
CBFA2T2	-0.018590024	0.939960521
CBFA2T3	-0.318639569	0.125933541
CBL	0.21942741	0.232901719
CBLB	0.577833931	0.040142545
CBLC	-0.229279587	0.089898613
CBLIF	-0.071833569	0.394842499
CBLL2	0.151561539	0.150559807
CBLN1	-0.131028623	0.485130721
CBLN2	-1.581077093	0.005859251
CBLN4	-0.60685005	0.101704242
CBR1	-0.230678434	0.5718214
CBR3	0.309254478	0.184556922
CBR4	0.690330898	0.055086894
CBWD1	0.123201457	0.665450164
CBWD2	0.594710085	0.035747902
CBWD3	0.23590744	0.333079874
CBX1	-0.09397909	0.762900028
CBX3	1.099398571	0.000204607
CBX4	0.28458193	0.172744121
CBX6	-1.101801149	0.001954005
CBX7	-0.213210515	0.356014927
CBX8	0.140004211	0.560642078
CBY1	0.046779528	0.873556302

CC2D1A	0.280548209	0.140000239
CCAR1	0.015450589	0.941983489
CCAR2	-0.154421723	0.489250951
CCBE1	-0.266820358	0.318547548
CCDC102A	0.000772467	0.997942116
CCDC103	2.442640846	0.000430084
CCDC105	-0.219255003	0.114270905
CCDC106	-0.048878137	0.79000918
CCDC107	0.222056545	0.392042779
CCDC110	-0.202041269	0.083448851
CCDC113	0.040119603	0.92602123
CCDC115	0.003518318	0.988788866
CCDC116	-0.033276271	0.777936515
CCDC117	0.293887162	0.136663136
CCDC12	0.128525417	0.323798441
CCDC122	0.204528815	0.537165089
CCDC124	-0.15755497	0.507148231
CCDC125	0.837295981	0.000653805
CCDC126	0.063408873	0.76589421
CCDC127	0.054239836	0.678705969
CCDC13	0.121735615	0.32786293
CCDC130	0.735736028	0.006731476
CCDC134	0.19187013	0.243105163
CCDC136	0.38894595	0.39175816
CCDC138	0.449230982	0.156921607
CCDC14	1.177282161	0.001409651
CCDC141	0.226945228	0.351099922
CCDC142	-0.045773737	0.846242672
CCDC148	-0.182156964	0.268208449
CCDC149	-0.134880204	0.443373422
CCDC15	0.936896237	0.008316901
CCDC153	0.194997623	0.332875404
CCDC160	0.104777139	0.486952504
CCDC170	0.132042918	0.595027084
CCDC171	-0.132642706	0.479545873
CCDC173	0.490276232	0.258835887
CCDC174	0.347997732	0.035405448
CCDC175	-0.079480234	0.780791174
CCDC178	0.274358291	0.6214455
CCDC18	1.140388364	0.005557192
CCDC181	0.116319785	0.455635494
CCDC182	-0.100412523	0.309608278
CCDC183	-0.217781707	0.191008249
CCDC184	-0.335930248	0.120281267
CCDC185	0.029173446	0.864891395
CCDC186	-0.73211615	0.002150168
CCDC188	0.075892236	0.694260995
CCDC190	-0.648680029	0.150554333

CCDC192	0.11358324	0.466000473
CCDC198	-0.062287005	0.546691005
CCDC22	0.198270155	0.164475288
CCDC24	0.070098493	0.63571801
CCDC25	0.099813408	0.589238898
CCDC27	-0.064928969	0.552352587
CCDC28A	-0.177144283	0.231877304
CCDC28B	0.03307839	0.82043669
CCDC30	0.413840705	0.263873634
CCDC32	0.050682182	0.847927611
CCDC34	0.143824374	0.494122093
CCDC38	-0.156135645	0.16792791
CCDC42	-0.147723548	0.25776106
CCDC43	-0.099576775	0.549312728
CCDC47	0.363378561	0.029045149
CCDC50	0.567008121	0.051020853
CCDC54	-0.083041061	0.380167689
CCDC57	0.371795285	0.239336125
CCDC59	0.211210698	0.151903986
CCDC6	-0.546808687	0.046525227
CCDC60	0.081799715	0.503836731
CCDC62	-0.028887843	0.879320595
CCDC63	-0.032674152	0.835141576
CCDC65	0.194899332	0.207142254
CCDC69	-0.11756667	0.459874564
CCDC7	-0.505049866	0.040494122
CCDC70	0.022015497	0.852044274
CCDC71	0.468084967	0.038645369
CCDC71L	0.404076026	0.287177172
CCDC74A	-0.295561303	0.090626249
CCDC74B	0.140015227	0.482742925
CCDC77	0.479440511	0.002165273
CCDC8	0.174188347	0.512056444
CCDC80	0.786618794	0.242253191
CCDC82	0.098971021	0.741269852
CCDC83	-0.159730237	0.142507722
CCDC85B	-0.357500758	0.034355668
CCDC86	0.034211775	0.897443359
CCDC87	-0.096461692	0.37598287
CCDC88B	0.11583211	0.368580248
CCDC89	0.264105913	0.169563691
CCDC9	0.323664844	0.380079378
CCDC90B	0.069310153	0.8229435
CCDC91	-0.068416057	0.723362559
CCDC92	-0.319522556	0.125375137
CCDC93	1.208382482	0.000331368
CCDC96	0.015894366	0.926926533
CCDC97	0.316823489	0.15449033

CCDC9B	0.356694988	0.23831229
CCER1	0.021806923	0.802667241
CCER2	0.287595656	0.404656768
CCHCR1	0.216473956	0.284137732
CCIN	-0.055121156	0.624315321
CCK	-0.896564502	0.005746243
CCKAR	0.001684139	0.99680095
CCKBR	-0.684035451	0.027018172
CCL1	-0.243763841	0.041419454
CCL11	-0.054872115	0.68189963
CCL13	-0.080634919	0.660597417
CCL14	0.127593878	0.213262526
CCL17	-0.113343287	0.596668065
CCL19	-0.053556989	0.810684451
CCL2	1.286707501	0.336105286
CCL20	0.122984834	0.532011599
CCL21	0.113823072	0.433764215
CCL22	-0.123788673	0.37965508
CCL24	-0.120904815	0.286555207
CCL25	0.007460903	0.956825161
CCL26	0.002179473	0.992175224
CCL27	-0.029772895	0.831791087
CCL28	-0.15146627	0.467595665
CCL3	0.224092552	0.575516391
CCL4	0.368516019	0.454080942
CCL8	0.174032964	0.55298525
CCM2	0.21374212	0.257587266
CCM2L	-0.152974493	0.303233859
CCN1	0.174987148	0.869171921
CCN2	0.324290572	0.749573888
CCN3	-0.28206113	0.506700199
CCN4	1.1097843	0.001776331
CCN5	0.021442399	0.901377776
CCN6	-0.024612025	0.826802803
CCNA1	-0.554160923	0.033803615
CCNA2	1.366199498	0.037288297
CCNB1	0.804714665	0.093804428
CCNB1IP1	0.270224718	0.482332494
CCNB2	0.620643343	0.1038076
CCNB3	0.02843125	0.839402086
CCNC	0.183840836	0.431715175
CCND1	0.392929562	0.50452383
CCND2	0.788064348	0.050110806
CCND3	0.030843792	0.884083488
CCNDBP1	-0.514991309	0.025976864
CCNE1	0.349354285	0.545059515
CCNE2	0.287181327	0.491388044
CCNF	0.209419163	0.434932199

CCNG1	-0.139253073	0.767258191
CCNG2	-0.25289454	0.373254452
CCNH	0.406999129	0.086780806
CCNI	-0.518492841	0.00525333
CCNJL	0.035794431	0.883723493
CCNK	0.005329972	0.984079638
CCNL1	0.861106966	0.002864507
CCNL2	1.206029467	0.001192251
CCNQ	-0.359760199	0.136317525
CCNT1	0.287914019	0.126004782
CCNT2	0.418096006	0.01535789
CCNY	-0.643583481	0.000269441
CCP110	-1.143820522	0.007591764
CCR1	0.604110547	0.050270949
CCR10	0.067455704	0.510426591
CCR3	0.011792535	0.939020857
CCR4	-0.050518193	0.707337139
CCR5	-0.00542128	0.965258071
CCR6	-0.122390362	0.318790499
CCR7	0.334906482	0.033803615
CCR8	-0.030481994	0.789016468
CCRL2	0.040945171	0.678361974
CCS	0.657636923	0.001024833
CCSAP	-0.081921264	0.854151388
CCSER2	-0.712139685	0.004919031
CCT4	-0.329527295	0.307586975
CCT5	0.329570002	0.196209899
CCT6A	0.478707676	0.061559966
CCT6B	-0.056631323	0.765619121
CCT8	0.24236683	0.340516155
CCT8L2	0.080928832	0.484795797
CCZ1	0.95962857	0.000289895
CCZ1B	0.171698088	0.509043396
CD101	0.686506367	0.023262209
CD14	1.679160221	0.001966114
CD151	0.3179546	0.448441679
CD163	3.15867292	0.000902126
CD163L1	0.17565929	0.558261496
CD164	0.669849632	0.021692485
CD164L2	-0.203620875	0.11793683
CD177	-0.214826446	0.076524118
CD180	1.237327851	0.00766069
CD1A	-0.019453009	0.859266603
CD1B	-0.073649478	0.456985798
CD1C	0.227648318	0.016932341
CD1D	-0.044386531	0.726649288
CD1E	-0.282894974	0.106511131
CD2	0.252740703	0.133809597

CD200	-1.295773814	0.011653303
CD200R1	-0.073607954	0.602304979
CD200R1L	-0.182053493	0.105539538
CD207	0.459151658	0.027926706
CD226	0.339344878	0.039205174
CD244	-0.045496137	0.654808174
CD247	-0.099235982	0.5208979
CD248	0.511686111	0.443817792
CD27	0.246648709	0.068391454
CD274	-0.133318662	0.766527221
CD28	0.090710772	0.593271905
CD2AP	0.172244337	0.68497309
CD2BP2	0.196659611	0.326832679
CD300A	0.534373768	0.015899204
CD300C	0.184369208	0.350979442
CD300E	-0.041948574	0.732432758
CD300LB	-0.056866241	0.601588011
CD300LD	-0.038265193	0.817111771
CD300LF	0.617274392	0.014375102
CD300LG	-0.092443012	0.543983376
CD320	0.216181246	0.26242907
CD33	-0.01693918	0.95239805
CD34	0.298245789	0.511253936
CD36	0.194012107	0.362976226
CD37	1.033408971	0.044140136
CD38	0.370858858	0.475725358
CD3D	0.007104919	0.952456416
CD3E	-0.044923806	0.781198768
CD3G	0.147033829	0.115683548
CD4	0.715297307	0.006735106
CD40	0.353531729	0.084213699
CD40LG	0.057335472	0.6062359
CD44	2.318613883	0.018591189
CD46	0.143939363	0.476416817
CD47	-0.074376664	0.827077846
CD48	0.064893421	0.72777771
CD5	0.054454516	0.702299889
CD52	-0.007109692	0.97653473
CD55	-1.052079387	0.005599703
CD58	0.717709668	0.015662415
CD5L	0.004942304	0.969663418
CD6	-0.283131072	0.045585085
CD68	1.775796587	0.003876591
CD69	0.894701643	0.039667929
CD7	-0.080555696	0.712617261
CD70	0.306718325	0.034227184
CD72	0.332869105	0.254132198
CD79A	-0.396657862	0.040673752

CD80	0.052628903	0.639818461
CD81	0.324952609	0.036219963
CD82	0.627264296	0.076338257
CD83	0.264146345	0.553475251
CD86	1.04883139	0.038599609
CD9	0.36258025	0.127118786
CD93	1.790815546	0.000331368
CD96	0.205760606	0.175391196
CD99	1.821421516	0.000739219
CD99L2	-0.884217692	0.000851026
CDA	-0.184880368	0.566978273
CDADC1	-0.039998057	0.855304945
CDAN1	0.138359044	0.402740226
CDC123	-0.194547351	0.344540219
CDC14A	0.163393448	0.583881916
CDC14B	0.282710326	0.369866211
CDC20	0.670463266	0.189638967
CDC20B	-0.140127543	0.113464855
CDC23	0.399795068	0.114971945
CDC25A	0.256659117	0.306104226
CDC26	0.328347632	0.078386588
CDC34	-0.026645341	0.935182135
CDC37	0.216709138	0.414686924
CDC37L1	-0.475570811	0.057716088
CDC40	-0.225320648	0.295121535
CDC42	0.214490515	0.154517401
CDC42BPA	-0.309753618	0.169371182
CDC42BPG	0.050835619	0.749573888
CDC42EP1	-0.435849709	0.117089749
CDC42EP3	-0.293576278	0.272725378
CDC42EP4	0.334376282	0.267906543
CDC42EP5	0.186363883	0.325148748
CDC42SE2	-0.302216792	0.114270905
CDC45	0.598624328	0.057216668
CDC5L	0.295225306	0.110603363
CDC6	0.35987367	0.323217852
CDC7	1.129819128	0.017155822
CDC73	0.575823819	0.001554747
CDCA2	0.451537074	0.313708077
CDCA3	0.193892357	0.444367648
CDCA4	-0.05621771	0.866152697
CDCA5	0.320531073	0.103814384
CDCA7	0.961993679	0.020555975
CDCP1	0.06024386	0.928374371
CDCP2	-0.133027011	0.223870308
CDH1	-0.004782295	0.992886227
CDH10	-0.649212119	0.37636203
CDH11	0.801447438	0.070281277

CDH15	0.026870177	0.933575125
CDH16	-0.018472076	0.884920673
CDH17	-0.10545082	0.238058584
CDH19	-0.58857312	0.147044206
CDH2	0.516088211	0.139745462
CDH20	-0.511620704	0.554645204
CDH22	-1.232442244	0.003619998
CDH23	-0.222248217	0.33775477
CDH24	0.193920947	0.568553857
CDH26	-0.142812473	0.436573225
CDH3	-0.159912131	0.781326457
CDH5	0.383815901	0.426612208
CDH6	0.23248721	0.651014997
CDH7	-0.369803505	0.247613471
CDH8	-3.037131358	0.000151704
CDH9	-1.713600728	0.009689545
CDHR1	-0.042902636	0.924838339
CDHR2	-0.111022037	0.297903216
CDHR3	0.329665767	0.315884113
CDHR4	-0.044275536	0.880038975
CDHR5	-0.029938789	0.822013734
CDIP1	-0.705031949	0.016817157
CDIPT	-0.256061692	0.269592402
CDK1	0.538991509	0.154815849
CDK10	0.253147704	0.306917517
CDK11A	0.15659571	0.518607925
CDK11B	-0.386071106	0.143899893
CDK13	0.237802462	0.1452049
CDK14	0.511187079	0.120693464
CDK15	-0.277184032	0.327635999
CDK16	-0.240989264	0.170476056
CDK18	-0.545898698	0.281773457
CDK19	-0.208847462	0.578622591
CDK2	0.717187379	0.052466203
CDK20	0.297419419	0.293228547
CDK2AP1	0.223419071	0.19438243
CDK2AP2	-0.259366812	0.247750029
CDK4	0.368904087	0.314740592
CDK5	-0.5770112	0.019547983
CDK5R1	-1.019957432	0.019580663
CDK5R2	-0.676899352	0.022930456
CDK5RAP1	0.201586173	0.329795252
CDK5RAP2	0.564991047	0.01845413
CDK5RAP3	0.699573401	0.011912584
CDK7	-0.116648637	0.730645777
CDK8	-0.216821734	0.323795214
CDKAL1	0.786686249	0.000627774
CDKL1	-0.137490459	0.62413692

CDKL2	-1.587318066	0.00186252
CDKL3	-0.104887414	0.544725411
CDKN1B	-0.007239634	0.97776514
CDKN1C	-0.56066649	0.013803795
CDKN2A	-0.61452422	0.279774949
CDKN2AIP	0.222046213	0.361019878
CDKN2AIPNL	-0.002330234	0.994980306
CDKN2C	0.638287345	0.050656469
CDKN2D	-0.512141915	0.033469262
CDKN3	0.704859476	0.032618429
CDNF	0.095411222	0.576567034
CDO1	0.014909791	0.983832633
CDPF1	0.047305468	0.785680379
CDR2L	-0.473880232	0.052870682
CDRT1	0.19862726	0.488963518
CDS1	-1.163183773	0.025596149
CDS2	-0.196249735	0.380079378
CDSN	-0.060054559	0.804091886
CDT1	0.243411112	0.318175113
CDX1	-0.328338406	0.063208828
CDX2	-0.002397401	0.990996918
CDX4	-0.070587752	0.489137041
CDY1	0.121931869	0.268573438
CDY2A	-0.083971707	0.598057051
CDY2B	-0.113403041	0.329413397
CDYL2	-1.243875632	0.001005193
CEACAM1	0.111235212	0.279908606
CEACAM3	-0.131558064	0.426612208
CEACAM4	-0.167174253	0.179602619
CEACAM5	-0.177910313	0.274252787
CEACAM6	-0.208800239	0.219970844
CEACAM7	-0.15241162	0.327556837
CEACAM8	-0.269007279	0.012301856
CEBPA	0.536472961	0.147608175
CEBPD	0.820674268	0.083132498
CEBPE	-0.213951517	0.058070792
CEBPZ	0.357611162	0.070281277
CEL	0.049837602	0.740178547
CELA1	-0.091321673	0.378881173
CELA2A	0.191308112	0.551807634
CELA3A	-0.167204733	0.376582561
CELF1	0.223948664	0.145538728
CELF3	0.112933014	0.348378671
CELF4	-2.30014682	0.003512268
CELF6	-0.289683786	0.346281317
CELSR1	0.220217304	0.598057051
CELSR2	-0.294353019	0.512709781
CELSR3	-0.194716339	0.515432943

CEMIP	-0.853840652	0.205446848
CEMIP2	0.24897595	0.547138402
CEND1	-1.672405102	0.003270886
CENPB	0.569383217	0.005869586
CENPC	-0.028668666	0.931511452
CENPE	1.299978138	0.020312883
CENPF	1.66049927	0.024785288
CENPH	0.369846565	0.107462271
CENPI	1.017481897	0.044369981
CENPM	0.429589547	0.110059166
CENPO	0.515835959	0.076935303
CENPQ	0.18918192	0.346507164
CENPT	-0.012198827	0.969215809
CENPU	1.127926084	0.011829886
CENPW	0.067297952	0.819909224
CENPX	-0.042108669	0.865529614
CEP112	0.463918191	0.187190382
CEP120	0.619525564	0.000783213
CEP128	0.566540821	0.01324414
CEP135	0.990778156	0.002492897
CEP162	1.035714946	0.000381607
CEP164	0.450163261	0.075609453
CEP170	0.105638412	0.55298525
CEP19	0.53191496	0.063240636
CEP250	0.401371412	0.129525902
CEP290	0.007402939	0.98042003
CEP350	0.367010866	0.100996839
CEP41	0.448384007	0.03315189
CEP55	0.30729215	0.452673547
CEP63	0.33433901	0.044170471
CEP68	-0.121769186	0.558261496
CEP70	0.226595581	0.389435147
CEP72	-0.055442338	0.78342719
CEP76	0.151975139	0.389435147
CEP83	1.169002694	0.001454069
CEP89	0.738403628	0.001056288
CEP95	0.710448692	0.031467109
CEP97	-0.088165855	0.782191352
CEPT1	0.561360421	0.020653563
CER1	-0.025848803	0.853715871
CERCAM	-1.286126217	0.004457905
CERK	-0.058428574	0.839163948
CERS2	-0.325022665	0.201466408
CERS3	0.052451708	0.642440544
CERS4	-0.187247209	0.309990191
CERS5	0.128298064	0.693033257
CERS6	-0.120769294	0.778244069
CES1	0.288183228	0.361878441

CETN1	0.052529787	0.651168817
CETN2	0.080745364	0.785071735
CETN3	0.408828314	0.047078994
CETP	-0.012170128	0.927569725
CFAP100	0.341626773	0.4457371
CFAP126	1.407857098	0.100174567
CFAP157	0.264464303	0.423226559
CFAP161	0.117698272	0.481412872
CFAP20	-0.141488229	0.525937835
CFAP206	0.363977108	0.298903943
CFAP298	0.350791748	0.067411593
CFAP300	0.302326988	0.134840884
CFAP36	-0.077766945	0.712015348
CFAP410	0.075465914	0.640752919
CFAP43	0.616647682	0.23713753
CFAP53	0.754520155	0.103598489
CFAP61	0.44027248	0.098985444
CFAP65	-0.002537139	0.990288759
CFAP69	0.58204846	0.073534026
CFAP70	0.554261623	0.173390717
CFAP77	0.058728605	0.773910522
CFAP97D1	-0.049312009	0.630467497
CFB	0.644778312	0.034988055
CFC1	-0.155497589	0.176802504
CFD	0.054766898	0.838917532
CFDP1	0.431415203	0.135638143
CFHR1	0.171612924	0.776457433
CFHR2	-0.532781484	0.292242641
CFHR5	-0.253961188	0.023282345
CFL1	0.291484153	0.567695781
CFL2	-0.786652618	0.004559667
CFLAR	0.559374722	0.009312966
CFP	0.066663822	0.730363414
CFTR	-0.184485466	0.075484765
CGAS	0.33155274	0.237421345
CGB1	0.203714663	0.425035431
CGB2	0.211161442	0.429306108
CGN	0.084293186	0.610476273
CGRRF1	-0.13215406	0.696930692
CH25H	0.60912545	0.26242907
CHAD	-0.869013181	0.006942795
CHAF1A	0.761119285	0.016753896
CHAF1B	0.272669747	0.449012487
CHAMP1	0.087397211	0.758326593
CHCHD1	-0.233690804	0.303821764
CHCHD10	-0.519648589	0.009946858
CHCHD2	0.131259476	0.468439363
CHCHD3	0.858411531	0.000589737

CHCHD4	-0.058549715	0.729035816
CHCHD5	0.348179897	0.068002914
CHCHD6	-0.062827143	0.606649052
CHCHD7	-0.123067669	0.49032626
CHD1	0.899995463	0.001210525
CHD1L	0.781286944	0.000974532
CHD3	-0.190037717	0.535399892
CHD6	0.304353549	0.095663309
CHD8	0.168192306	0.34866536
CHD9	0.52590803	0.024792405
CHDH	0.177608602	0.577168524
CHERP	0.207486475	0.274252787
CHGA	-2.053060924	0.001053722
CHGB	-0.613245168	0.291211053
CHI3L1	3.203751688	0.006589628
CHI3L2	1.397289567	0.026525449
CHIA	-0.085091885	0.311287029
CHIC2	1.109541278	0.102956472
CHKA	0.115692847	0.749523066
CHL1	0.58667001	0.508340807
CHM	0.065711724	0.75341415
CHML	-0.822770066	0.052792887
CHMP1A	0.075838533	0.762174089
CHMP1B	-0.200242442	0.385040423
CHMP2A	-0.127814548	0.629200991
CHMP2B	-0.308043957	0.180962503
CHMP4B	-0.16103708	0.460470474
CHMP4C	-0.31697727	0.267609207
CHMP5	-0.198643675	0.405193912
CHMP6	0.214632737	0.282459455
CHMP7	0.252215363	0.143516139
CHN1	-1.337999177	0.013403861
CHN2	-0.380683877	0.068147349
CHODL	0.294948387	0.414394125
CHORDC1	0.377668955	0.123447647
CHP1	-0.353753691	0.106370667
CHP2	-0.133850881	0.318281416
CHPF	0.237109789	0.566344542
CHPF2	0.057566011	0.873270613
CHPT1	0.275352394	0.389868572
CHRAC1	0.309018348	0.098693107
CHRD	-0.358817995	0.183472213
CHRDL2	0.246543641	0.2400032
CHRM1	-0.640691764	0.015093776
CHRM3	-0.7011685	0.076256414
CHRM4	-0.865785329	0.00239956
CHRM5	-0.605136118	0.076360903
CHRNA10	-0.124439613	0.481196182

CHRNA2	-0.329987099	0.030210499
CHRNA4	-0.181150265	0.254306297
CHRNA5	-0.099918803	0.776510832
CHRNA6	0.084183055	0.410637516
CHRNA7	0.001336933	0.9973031
CHRNA9	0.167671148	0.620584143
CHRNB1	0.015905197	0.966982584
CHRNB2	-0.856563464	0.022459642
CHRNB3	-0.130298465	0.240239771
CHRNB4	-0.070934313	0.451373362
CHRND	0.129461444	0.572629028
CHRNE	0.062346212	0.817881105
CHST1	-1.165607735	0.010638687
CHST10	0.028474072	0.866370176
CHST11	1.317611175	0.000814384
CHST13	-0.396067161	0.005557192
CHST14	0.611576402	0.076090731
CHST15	-0.209529898	0.553917583
CHST2	0.101280076	0.72028673
CHST6	0.436744442	0.295585953
CHST9	1.334700448	0.021160615
CHSY1	0.415971028	0.109119511
CHSY3	0.009414048	0.985057131
CHTF18	0.035743884	0.879901796
CHTOP	0.743270146	0.000151704
CIAO1	0.180579207	0.111086676
CIAO2A	0.344564031	0.169563691
CIAO3	0.116330007	0.53845611
CIAPIN1	0.27541807	0.24858739
CIART	-0.402779472	0.118578456
CIB1	0.268201419	0.169850016
CIB2	-0.184177712	0.223374357
CIB3	0.054516159	0.685402367
CIB4	-0.163645556	0.27793608
CIC	0.000914168	0.99800589
CIDEA	-0.010746299	0.94671625
CIITA	1.427574524	0.018774647
CILP	-0.007993399	0.960603739
CILP2	-0.281354996	0.113418848
CINP	0.172871215	0.566611412
CIP2A	0.695072967	0.04632088
CIPC	-0.064770302	0.834776267
CIR1	0.12707872	0.616944401
CISD1	-0.444956735	0.024817036
CITED2	-0.076771841	0.902832287
CITED4	-0.599866474	0.038556673
CKAP2	0.805787872	0.011327914
CKAP2L	1.1228125	0.030777055

CKAP4	-0.022803292	0.938370209
CKB	-0.701639216	0.173390271
CKLF	0.609253078	0.000690887
CKM	-0.223638479	0.152324857
CKMT1A	-1.111667576	0.012718786
CKMT1B	-2.517659643	0.0058159
CKMT2	-0.222810613	0.286243894
CKS2	1.278196279	0.032813584
CLASP1	0.114434949	0.513693168
CLASRP	0.190271594	0.618557122
CLBA1	0.384850889	0.087673335
CLC	-0.195212619	0.160298477
CLCA1	-0.091419124	0.326058883
CLCA2	-0.199231288	0.04142136
CLCA4	-0.992841058	0.013395748
CLCN1	-0.082191868	0.458398906
CLCN2	0.487842939	0.058354399
CLCN3	0.345846698	0.14504691
CLCN4	-1.563570532	0.002302815
CLCN6	0.011734419	0.971953247
CLCN7	0.144362711	0.569712378
CLCNKA	-0.050480152	0.78413419
CLCNKB	0.215103273	0.302520363
CLDN1	-0.453721891	0.331569694
CLDN10	-1.699126265	0.018573825
CLDN11	-0.935429262	0.012662973
CLDN12	-0.011958759	0.898595851
CLDN14	-0.03147747	0.801253954
CLDN16	-0.200399066	0.107411731
CLDN17	-0.009259422	0.923135849
CLDN18	0.157193639	0.13754129
CLDN20	0.005571078	0.96380606
CLDN23	-0.242768302	0.221757391
CLDN25	0.069635541	0.613196297
CLDN3	-0.139425265	0.665490054
CLDN4	0.134334744	0.37549496
CLDN6	-0.428623003	0.272519166
CLDN8	-0.310835245	0.070018393
CLEC10A	0.087371954	0.518422973
CLEC11A	0.140368768	0.457590223
CLEC12A	0.142396246	0.164313763
CLEC12B	-0.074049138	0.369103156
CLEC14A	0.102022908	0.519994015
CLEC18A	0.286859035	0.383582977
CLEC18C	0.516462478	0.020653563
CLEC19A	0.048499079	0.681191192
CLEC1A	0.173090855	0.252127612
CLEC1B	-0.078551478	0.384780797

CLEC20A	-0.163510327	0.41174686
CLEC2B	1.017441116	0.004500154
CLEC3A	0.007416539	0.953098268
CLEC3B	-0.11235342	0.583914788
CLEC4A	0.180930118	0.299443033
CLEC4C	-0.121442523	0.335647429
CLEC4D	-0.187533492	0.022283257
CLEC4E	0.166868356	0.295075692
CLEC4F	0.147681014	0.414686924
CLEC4G	-0.353662158	0.038372241
CLEC5A	1.025062368	0.007500217
CLEC6A	-0.008302928	0.954433066
CLEC7A	1.251137869	0.009063755
CLEC9A	-0.312179782	0.082058452
CLGN	0.018976644	0.936409681
CLHC1	0.890580288	0.006020258
CLIC1	1.128733432	0.045552482
CLIC2	0.285572194	0.231166126
CLIC3	-0.02868435	0.881976373
CLIC4	0.66804162	0.102780128
CLIC6	0.024902543	0.892641383
CLIP1	0.155635113	0.399340316
CLIP2	0.538199207	0.240167593
CLIP4	-0.377330124	0.100200988
CLK4	0.390517886	0.172016557
CLMN	-0.864669862	0.044522209
CLMP	-0.201109215	0.71038905
CLN5	0.388933408	0.151096255
CLN8	-0.038189339	0.847927611
CLNK	-0.090640135	0.230743036
CLNS1A	0.153869204	0.491158914
CLP1	-0.048802431	0.760767616
CLPP	0.084061713	0.720440528
CLPS	-0.112902482	0.210358003
CLPSL1	0.095507139	0.509043396
CLPSL2	-0.203246732	0.086120987
CLPTM1L	0.437908387	0.080768872
CLPX	0.257073146	0.17349014
CLRN3	-0.170244321	0.107274495
CLSTN2	-1.434958705	0.036794892
CLSTN3	-0.959902438	0.041960568
CLTA	-0.296317926	0.110354212
CLTC	-0.303678636	0.151947457
CLTRN	-0.053109013	0.716486195
CLU	0.25308477	0.573000336
CLUAP1	0.134666473	0.631524871
CLUH	-0.155368497	0.468156597
CLUL1	-0.302233787	0.046119664

CLVS1	-0.457636496	0.222107216
CLVS2	-1.074084661	0.212570884
CLYBL	0.386071278	0.248226123
CMA1	-0.104694367	0.267906543
CMAS	-0.330469429	0.355732302
CMBL	-0.22304601	0.461109885
CMC1	0.281530159	0.164496102
CMC2	-0.100617778	0.642889106
CMIP	-0.563897515	0.085425129
CMPK1	0.16652665	0.279415146
CMPK2	0.67052019	0.02952161
CMSS1	0.12790849	0.332668993
CMTM2	-0.024903352	0.859266603
CMTM3	1.649932393	0.000389052
CMTM4	-1.127747226	0.016615325
CMTM5	-0.044172454	0.944001602
CMTM6	0.651389915	0.032772464
CMTM7	0.493914841	0.128184123
CMTM8	0.056613263	0.929702416
CMTR1	0.146713248	0.271875473
CMTR2	0.344270434	0.063760341
CMYA5	1.091172073	0.065408457
CNBD1	-0.01093674	0.904256812
CNBD2	0.097504141	0.441706079
CNDP1	-2.642960905	0.011978751
CNDP2	0.04670342	0.847725163
CNEP1R1	0.457104782	0.099639765
CNFN	-0.131910371	0.461818232
CNGA1	-0.113787381	0.114071721
CNGA2	-0.122898487	0.415663144
CNGA3	0.73874212	0.054014736
CNGB1	-0.189253917	0.406530768
CNGB3	-0.097681423	0.287720729
CNIH1	0.200153768	0.375141671
CNIH2	-0.292162187	0.449141092
CNIH3	0.37643129	0.098501871
CNIH4	0.306886121	0.291211053
CNKSR1	0.10561929	0.536215124
CNKSR2	-0.85467739	0.27643327
CNMD	-0.045673472	0.817062158
CNN1	-1.209238876	0.103878201
CNN2	0.250923931	0.829985928
CNN3	1.071335762	0.001448285
CNNM1	-1.021714676	0.005386579
CNNM2	-0.660067091	0.002977765
CNNM3	-0.006439917	0.982945206
CNNM4	0.119657939	0.499541591
CNOT11	0.268886222	0.109696733

CNOT2	0.590968741	0.00305093
CNOT3	0.323306164	0.225724304
CNOT6	0.471221222	0.052762388
CNOT6L	0.191077707	0.351097651
CNOT7	0.111426029	0.523894917
CNOT8	0.187996896	0.329413397
CNOT9	0.544944626	0.002068027
CNP	-1.211818761	0.021742583
CNPPD1	0.096725834	0.688306212
CNPY1	0.003301947	0.982765512
CNPY2	0.273847302	0.2220945
CNPY4	0.715142311	0.0007686
CNR1	1.788939626	0.025948334
CNRIP1	-0.413324732	0.167502419
CNST	-0.434755397	0.111262938
CNTD1	0.047820983	0.736206294
CNTLN	0.916298146	0.008894029
CNTN1	-1.135186258	0.348237418
CNTN2	-1.886757789	0.010259208
CNTN5	-0.603763264	0.039616406
CNTN6	-0.18568702	0.210412136
CNTNAP1	-0.719711327	0.285728799
CNTNAP2	-2.935936339	0.004316834
CNTNAP3	0.264593175	0.657236467
CNTNAP4	-1.644678317	0.01119351
CNTNAP5	-1.69669338	0.021290241
CNTRL	1.063937818	0.001053722
CNTROB	0.663473281	0.015093776
COA1	0.806272103	0.000275651
COA4	0.460673171	0.06539208
COA6	-0.308163018	0.103226091
COA7	-0.089084795	0.683448753
COA8	0.315206486	0.133441614
COBL	-0.66939881	0.308277301
COBLL1	-0.087430141	0.776786373
COG1	-0.533982189	0.008981631
COG2	0.281183456	0.189348765
COG3	0.239842344	0.171793831
COG4	0.988710171	0.000511553
COG5	0.478734375	0.002567802
COG7	0.215157302	0.232267974
COG8	0.302127472	0.060482903
COIL	0.358186432	0.06539208
COL10A1	-0.069419452	0.86670478
COL11A1	-0.132160183	0.884560985
COL11A2	0.05510178	0.754802165
COL12A1	-0.014421997	0.990022357
COL15A1	0.49861095	0.058831789

COL16A1	0.908698422	0.050183209
COL17A1	-0.046027722	0.628067677
COL19A1	-0.13471114	0.398486049
COL1A1	0.782256093	0.512018484
COL1A2	1.400918908	0.238817137
COL20A1	1.159887419	0.030318565
COL21A1	0.090350882	0.700015813
COL22A1	0.794189541	0.062879145
COL24A1	-0.403309711	0.133331541
COL25A1	-0.085530266	0.709595462
COL26A1	-1.183877935	0.000126605
COL27A1	0.394829651	0.219662239
COL28A1	1.261341658	0.054240566
COL2A1	0.025278797	0.955864333
COL3A1	1.787011936	0.117967224
COL4A1	1.939366358	0.056632357
COL4A2	1.521577058	0.085748005
COL4A3	-0.12730455	0.3184098
COL4A5	-0.453965275	0.24372963
COL4A6	0.041287895	0.917193997
COL5A1	0.544935238	0.465188581
COL5A2	0.596218157	0.496608658
COL5A3	0.770849857	0.065878376
COL6A1	1.245797052	0.069431807
COL6A2	1.515544101	0.10399493
COL6A3	1.13666543	0.317655514
COL7A1	0.117038931	0.730363414
COL8A1	0.609745654	0.553475251
COL8A2	0.24626513	0.258017379
COL9A1	0.138532069	0.600309691
COL9A2	-0.10808795	0.603713048
COL9A3	0.634819655	0.166009707
COLEC10	-0.144501214	0.203243282
COLEC12	0.721533137	0.149257703
COLGALT2	0.370270498	0.448614769
COLQ	0.280608064	0.166026965
COMMD1	0.07807856	0.623425545
COMMD10	0.746847612	0.000537605
COMMD2	0.334387702	0.139569359
COMMD3	0.069274923	0.700479507
COMMD4	-0.05023933	0.855966277
COMMD5	0.204274164	0.196134898
COMMD6	-0.176238092	0.521278826
COMMD7	-0.428987328	0.089721575
COMMD8	0.086248484	0.737023599
COMMD9	0.051546755	0.792482806
COMP	-0.086340247	0.594000637
COMTD1	-0.002236245	0.990996918

COP1	0.448799625	0.011718351
COPB1	0.452353301	0.044369981
COPB2	0.31449875	0.181191921
COPE	-0.565626513	0.022985516
COPG1	0.149151379	0.547138402
COPG2	0.685842422	0.009409548
COPRS	-0.114003574	0.493948708
COPS2	-0.410246005	0.063729998
COPS3	-0.04442914	0.796566753
COPS4	-0.136455286	0.503157573
COPS5	0.359404186	0.046912077
COPS6	0.105847473	0.531227892
COPS7B	0.246487109	0.124427682
COPS8	0.066410689	0.801102444
COPS9	-0.444758665	0.006130132
COPZ2	0.197549232	0.638095427
COQ10B	-0.070662174	0.830359479
COQ2	1.099904667	0.00015288
COQ3	-0.012667591	0.941091514
COQ4	0.669930258	0.011141587
COQ5	0.231867945	0.073113834
COQ6	0.032265011	0.860002363
COQ7	0.230513057	0.197055799
COQ8A	0.148760293	0.518566194
COQ8B	0.591183177	0.009260952
COQ9	0.279539859	0.155522477
CORO1C	0.207600251	0.532744829
CORO2A	-0.486530679	0.056383937
CORO2B	-0.26440468	0.419236188
CORO6	-1.858475691	0.00010541
CORT	0.032030332	0.839170012
COTL1	0.537872359	0.221676849
COX10	0.335449035	0.134065224
COX11	-0.229161464	0.128237162
COX15	-0.221364619	0.174530912
COX17	0.456262477	0.012719369
COX18	0.458609801	0.001899052
COX20	0.465093887	0.098905266
COX4I1	-0.642350288	0.010751175
COX4I2	-0.0023178	0.990583302
COX5A	-0.45734074	0.017605167
COX5B	-0.352215928	0.024933462
COX6A1	-0.872064888	0.00097933
COX6B1	-0.30716644	0.065781539
COX6B2	-0.124228553	0.384216235
COX6C	0.166036647	0.263053899
COX7A1	-1.003721762	0.039095052
COX7A2	-0.242949016	0.164919812

COX7A2L	-0.083231278	0.655084643
COX7B	0.097763186	0.671295451
COX7B2	-0.00653013	0.953362739
COX7C	-0.113758785	0.592780583
COX8C	-0.071182684	0.652737384
CPA1	3.13E-05	0.999827462
CPA2	0.08713769	0.425097598
CPA3	-0.131170499	0.374228363
CPA4	-1.02102279	0.241422752
CPA5	-0.107505125	0.147601041
CPA6	0.064784062	0.836438676
CPAMD8	0.019976057	0.874712511
CPB1	-0.117529735	0.166614037
CPB2	-0.121428581	0.136621331
CPD	-0.186715638	0.640818885
CPE	-0.536733892	0.351052593
CPEB2	0.303970482	0.449570902
CPEB3	-1.096621686	0.066462881
CPEB4	-0.089159313	0.834076306
CPED1	0.272634588	0.532979116
CPLANE1	0.434411716	0.013943688
CPLANE2	-0.102190134	0.573793193
CPLX1	-0.810889955	0.025596149
CPLX2	-1.111982858	0.001899959
CPLX4	-0.049835755	0.572374594
CPM	0.439341987	0.298266643
CPN1	-0.258487839	0.043883128
CPNE2	0.452931474	0.249578491
CPNE3	0.145820313	0.638644577
CPNE4	-0.821798261	0.32786293
CPNE5	0.091333396	0.904792329
CPNE6	-1.053212071	0.007035913
CPNE7	-0.98411769	0.000263766
CPNE8	-0.64711788	0.065745695
CPNE9	-1.214798759	0.001056288
CPO	-0.132477496	0.4590178
CPOX	-0.369100038	0.20863383
CPPED1	-0.622776715	0.09026964
CPQ	0.068097878	0.835312849
CPSF1	0.246204182	0.223870308
CPSF2	0.44462579	0.126915641
CPSF3	0.48452229	0.057160472
CPSF4	0.883492354	0.005445074
CPSF6	0.150676762	0.534663977
CPT2	0.699333166	0.036926773
CPVL	0.955086288	0.001003819
CPXCR1	-0.164586167	0.089162742
CPXM1	1.813705868	0.008943538

CPXM2	-0.027128436	0.908578479
CPZ	-0.091608947	0.648110551
CR1	0.196100047	0.311113182
CR2	-0.142116897	0.203427605
CRABP1	-0.166929851	0.384570286
CRABP2	0.352460889	0.428751398
CRACR2B	-0.273280155	0.338119276
CRADD	0.732673542	0.005081616
CRB2	0.780252226	0.036600715
CRB3	-0.045262287	0.788380786
CRBN	-0.272598181	0.250939062
CRCP	0.166442064	0.323217852
CRCT1	-0.237010154	0.058987158
CREB3	-0.451398872	0.067292748
CREB3L1	-0.053674194	0.927473249
CREB3L2	0.533771801	0.152650935
CREB3L3	-0.130316427	0.278761589
CREB3L4	0.20229926	0.063836072
CREB5	0.78273946	0.037650934
CREBBP	0.190683312	0.310474997
CREBL2	-0.3336055	0.127373874
CREBRF	0.021489729	0.936703758
CREG1	-0.319916948	0.227318837
CREG2	-2.269442744	0.005391307
CRH	-0.220604684	0.038306095
CRHBP	-1.402903823	0.000910583
CRIM1	-0.880955673	0.080148455
CRIP1	0.181227314	0.446425189
CRIP2	-0.037545734	0.843764124
CRIP3	0.415370837	0.036219963
CRIPT	-0.092116648	0.799704143
CRISP2	-0.172186303	0.126968319
CRISP3	0.085882475	0.425368046
CRISPLD1	1.967462268	0.000888995
CRISPLD2	0.425965137	0.4436621
CRK	0.473598891	0.07570597
CRKL	0.010730995	0.968889368
CRLF1	-0.648294052	0.009879371
CRLF2	-0.09740237	0.361611858
CRLF3	0.403975355	0.015949182
CRLS1	0.598274134	0.004981702
CRMP1	-0.201563465	0.674472706
CRNKL1	0.077991989	0.725113118
CRNN	-0.031178545	0.83633567
CROCC	0.082004906	0.579975928
CRP	0.01155329	0.941091514
CRTAC1	-0.490467927	0.021342376
CRTAM	-0.042427745	0.640818885

CRTAP	-0.04544267	0.896454017
CRTC1	-0.539464692	0.157853617
CRTC3	-0.116380118	0.689933754
CRX	0.041357266	0.821001661
CRY1	0.588875107	0.012268421
CRY2	-0.503160837	0.068970312
CRYAA	0.041479815	0.738139199
CRYAB	0.050985817	0.912659903
CRYBA1	0.025919722	0.825626798
CRYBA2	-0.094353721	0.391833864
CRYBA4	-0.025291766	0.880038975
CRYBB2	-0.238809873	0.138219217
CRYBB3	-0.027234957	0.886755045
CRYGA	-0.064402561	0.72777771
CRYGB	0.031990305	0.850397328
CRYGC	-0.219677197	0.124886222
CRYGD	0.067458328	0.582636628
CRYGN	-0.112702462	0.229162614
CRYGS	0.768648666	0.000389052
CRYL1	-0.19119691	0.653942911
CRYM	-1.251490939	0.080768872
CRYZL1	0.590828799	0.054240566
CS	-0.065308662	0.813119406
CSAG1	-0.151323634	0.309003688
CSDC2	-0.443348978	0.059166821
CSE1L	-0.105042889	0.776786373
CSF1	0.737298139	0.013337633
CSF2	-0.079763015	0.3184098
CSF2RB	0.190414181	0.374191921
CSF3R	0.838822244	0.022459642
CSGALNACT2	-0.266436206	0.288918131
CSHL1	-0.304307253	0.186056479
CSK	0.344267367	0.14091306
CSKMT	-0.075276288	0.746533995
CSN1S1	0.255025237	0.669954425
CSN2	-0.209186024	0.104893376
CSN3	-0.300184613	0.01743244
CSNK1A1L	0.009405711	0.959726867
CSNK1D	0.159669556	0.544856765
CSNK1E	0.257135301	0.18535029
CSNK1G3	-0.193783797	0.232654739
CSNK2A1	0.23411123	0.295585953
CSNK2A2	0.118457464	0.547525935
CSPG4	0.730292297	0.172220439
CSPG5	0.141722026	0.791145992
CSPP1	0.452441977	0.15149962
CSRNP1	0.2424429	0.398392247
CSRP1	0.069509036	0.691193069

CSRP2	1.148165187	0.036497781
CSRP3	-0.034683263	0.727094424
CST1	-0.541874756	0.010751175
CST11	-0.137319168	0.179584139
CST2	0.196876645	0.348922502
CST3	-0.088996083	0.893828555
CST5	-0.207734835	0.072528403
CST6	-0.040942563	0.860727174
CST7	0.102227044	0.490985728
CST8	0.033983472	0.784418087
CST9	-0.131374129	0.254297669
CSTA	0.04099681	0.794214198
CSTB	0.186758113	0.438825668
CSTF1	0.285536301	0.111027902
CSTF2	0.168981732	0.452883153
CSTF2T	-0.167051947	0.488882599
CSTF3	0.385259992	0.16925862
CSTL1	0.001854183	0.986979433
CT55	-0.154630325	0.212570884
CT83	0.154192683	0.372815722
CTAG1A	-0.117822714	0.412315933
CTAG2	-0.043216816	0.858005023
CTAGE1	0.017960772	0.928243336
CTAGE8	0.252684612	0.265699994
CTBP2	0.020042011	0.915870551
CTCF	0.322351935	0.093677832
CTDP1	0.35988479	0.048385344
CTDSP1	0.368862073	0.058132615
CTDSPL2	0.573092881	0.010555103
CTF1	-0.405585656	0.091366606
CTH	0.560561772	0.139569652
CTHRC1	0.285650066	0.510790042
CTLA4	-0.148327079	0.138969461
CTNNA1	0.067066703	0.794214198
CTNNA2	-0.666617995	0.048880255
CTNNAL1	-0.409776566	0.395816592
CTNNB1	0.283723984	0.212980696
CTNNBIP1	-0.026547688	0.904689871
CTNNBL1	0.288113501	0.072245427
CTNS	0.451381399	0.029741798
CTPS1	0.308277708	0.548572116
CTPS2	0.656026022	0.013749517
CTR9	0.030644815	0.927976981
CTRB1	0.04187128	0.859802948
CTRC	0.081979954	0.608009487
CTSA	0.160623696	0.492496846
CTSB	0.6451194	0.016766817
CTSC	1.250498166	0.022050211

CTSD	-0.338291525	0.203901234
CTSE	-0.094716488	0.449012487
CTSF	-0.520676912	0.054231614
CTSG	-0.181652796	0.11910469
CTSH	-0.028828106	0.941983489
CTSL	0.364875342	0.266622752
CTSO	0.723482212	0.049688298
CTSS	1.638411989	0.001834847
CTSV	-0.142700531	0.373009992
CTSW	0.114976079	0.373498802
CTSZ	0.997641833	0.012204489
CTTN	0.181620259	0.478931255
CTTNBP2	0.402565101	0.576779548
CTTNBP2NL	0.167489856	0.560068078
CTU1	-0.021723512	0.904212194
CTXN1	-0.582969961	0.137102674
CTXN2	-0.207361411	0.117767257
CUBN	0.882445405	0.019789186
CUEDC1	0.618943688	0.030168363
CUEDC2	-0.457740002	0.02888318
CUL1	0.236684931	0.100094889
CUL3	0.203790285	0.233586232
CUL4A	0.358147445	0.052762388
CUL4B	0.02995504	0.912659903
CUL5	-0.033128293	0.861348324
CUL7	0.231053054	0.51880811
CUL9	0.391950053	0.098501871
CUTA	-0.120787381	0.454540665
CUTC	-0.057189873	0.709670769
CWC15	0.069080078	0.70495706
CWC25	0.202710742	0.250438598
CWC27	-0.168420814	0.295585953
CWF19L1	-0.131950906	0.615913073
CWF19L2	0.248510636	0.23564844
CWH43	-0.13509197	0.099126975
CX3CL1	-0.339165028	0.500451938
CXCL1	0.216727714	0.310482173
CXCL10	2.265426931	0.006525359
CXCL11	0.426788534	0.116878637
CXCL12	0.038367131	0.927581468
CXCL13	0.221514071	0.516197819
CXCL14	-0.674540198	0.498206112
CXCL17	-0.121737172	0.391833864
CXCL2	0.424695113	0.019993679
CXCL3	-0.050020198	0.808607914
CXCL5	0.229819001	0.741065302
CXCL6	0.193609165	0.574475876
CXCL8	0.557783264	0.310819867

CXCL9	0.396330695	0.072444373
CXCR1	-0.076376096	0.608979538
CXCR2	0.054860931	0.621609432
CXCR5	-0.086629934	0.553105314
CXCR6	0.07080714	0.572111593
CXORF38	0.275142352	0.1339868
CXORF58	-0.108487328	0.120281267
CXXC4	0.200533306	0.632111305
CXXC5	0.17872575	0.331569694
CYB561D2	0.058315922	0.775232596
CYB5A	0.095489354	0.678284057
CYB5B	-0.479109577	0.058188252
CYB5D2	-0.353594499	0.098691561
CYB5R1	-0.042784136	0.912079198
CYB5R2	-0.688483157	0.01599502
CYB5R4	0.103560632	0.563322948
CYBA	0.176209382	0.653843421
CYBB	2.386247051	0.000884493
CYBRD1	-0.275950823	0.274320551
CYC1	0.001690611	0.993903659
CYCS	0.145736995	0.420526737
CYFIP1	0.479599746	0.064849073
CYGB	-0.455505693	0.279385306
CYHR1	-0.043929073	0.735925036
CYLC1	-0.081808788	0.409675613
CYLC2	-0.138179847	0.19380966
CYLD	-0.370539056	0.082496364
CYP11A1	-0.119588923	0.330843428
CYP11B1	0.050641684	0.601426968
CYP11B2	-0.079915341	0.661188795
CYP17A1	-0.044596241	0.7879778
CYP19A1	-0.000914921	0.993945285
CYP1A1	-0.27733663	0.132252994
CYP1A2	-0.066124971	0.712525829
CYP1B1	0.092532816	0.855304945
CYP20A1	-0.213412167	0.292092737
CYP21A2	0.426299944	0.063747636
CYP24A1	-0.431697665	0.145025939
CYP26A1	-0.095145555	0.529207785
CYP26B1	-0.483538512	0.030713438
CYP26C1	-0.054170057	0.664232577
CYP27A1	0.414383399	0.323347141
CYP27B1	0.133765622	0.345354743
CYP27C1	0.148245354	0.382458915
CYP2A13	-0.114580131	0.623458448
CYP2A6	-0.25771495	0.312138794
CYP2B6	-0.351462258	0.02215249
CYP2C19	0.061506901	0.556803228

CYP2C8	0.080482501	0.849866923
CYP2C9	-0.111530428	0.3184098
CYP2D6	0.010143009	0.969414329
CYP2E1	-0.262864146	0.266915426
CYP2F1	-0.250518305	0.03171533
CYP2J2	-0.406712405	0.401730843
CYP2R1	0.209508312	0.366502877
CYP2S1	-0.177403986	0.34166678
CYP2U1	-0.128839496	0.712015348
CYP2W1	-0.09997504	0.441624749
CYP39A1	0.229327046	0.467379186
CYP3A4	-0.141982063	0.348378671
CYP3A43	-0.044993496	0.718751008
CYP46A1	-1.247344348	0.008431157
CYP4A11	0.22866301	0.167560462
CYP4A22	-0.065394719	0.762174089
CYP4F11	-0.089006079	0.845289305
CYP4F12	-0.134153113	0.534214966
CYP4F2	-0.106765641	0.393433755
CYP4F22	-0.020037553	0.893241153
CYP4F3	-0.117038083	0.530163682
CYP4V2	0.390289895	0.298903943
CYP4X1	-1.210238725	0.01322427
CYP4Z1	-0.201836835	0.20003068
CYP51A1	-0.321548613	0.218287399
CYP7A1	0.001225864	0.992633147
CYP7B1	-1.073621477	0.002329975
CYP8B1	-0.17726073	0.159583742
CYSLTR1	0.128444417	0.1627352
CYSLTR2	0.195237156	0.305720857
CYSRT1	0.17241659	0.29703521
CYSTM1	-0.331495296	0.098350432
CYTH1	0.019833815	0.954613647
CYTH3	0.210757494	0.226235767
CYTH4	0.865750522	0.011962872
CYTIP	0.331140366	0.066462881
CYTL1	0.185793088	0.743307749
CYYR1	0.09389785	0.794592949
CZIB	0.428930788	0.01690534
DAAM1	-0.018078189	0.96841268
DAAM2	-1.193006009	0.044213958
DAB1	-0.012835383	0.981263887
DAB2	0.859193972	0.201016628
DAB2IP	-0.240636933	0.228630078
DACH1	-0.567781488	0.261889537
DACT1	-0.018463725	0.953957295
DACT2	-0.016826012	0.930486251
DACT3	-0.710634589	0.011584437

DAD1	0.050930096	0.845275235
DAGLA	-0.322454619	0.344994288
DAND5	-0.048629796	0.823896006
DAO	-0.167408684	0.068025038
DAP	0.283870971	0.216122877
DAP3	0.370121959	0.035457643
DAPK1	-0.567782317	0.122433204
DAPK2	-0.16098202	0.295121535
DAPK3	-0.042150008	0.928120565
DAPP1	0.252817367	0.037569424
DARS2	0.285388686	0.334890212
DAW1	-0.445145378	0.074440514
DAZ2	0.215395725	0.516550683
DAZ4	0.236197971	0.487841801
DAZAP2	0.054432922	0.810771254
DBF4	0.895661696	0.016817157
DBF4B	0.403393169	0.04381043
DBI	1.008794335	0.001328451
DBN1	0.120956414	0.664890362
DBNDD1	-0.572781207	0.044017184
DBNL	0.65745337	0.0240514
DBP	-1.023638805	0.051595279
DBR1	0.374616893	0.015388688
DBT	0.05544238	0.844064696
DBX1	-0.080596854	0.589238898
DBX2	0.616749232	0.165431419
DCAF1	0.445543904	0.011608304
DCAF10	-0.083245123	0.743284488
DCAF12	0.382237027	0.215365311
DCAF12L1	-0.233136746	0.070281277
DCAF13	0.185043146	0.342865221
DCAF16	0.778158516	0.001306489
DCAF17	0.156315851	0.385170796
DCAF4	0.07101956	0.796558623
DCAF4L2	0.058912902	0.725073947
DCAF6	-1.099428431	0.001455335
DCAF7	0.065163896	0.817338701
DCAKD	0.047654333	0.886782715
DCANP1	-0.080197547	0.573439645
DCBLD1	0.136615061	0.732799122
DCBLD2	0.483751879	0.565587548
DCD	-0.143159369	0.221170849
DCDC1	0.548472297	0.05014765
DCDC2	0.072709817	0.789016468
DCDC2C	-0.043022051	0.661668618
DCHS1	-0.436641044	0.059593665
DCHS2	-0.276467196	0.231113261
DCK	-0.04814895	0.81684427

DCLK1	-0.782064	0.288380428
DCLRE1A	-0.098654959	0.71174887
DCLRE1B	0.607641614	0.018844974
DCN	0.537483653	0.205465654
DCP1A	0.276437108	0.067292748
DCP1B	0.07910785	0.746879258
DCP2	0.642414295	0.000612048
DCPS	0.671570126	0.005388978
DCST2	-0.054113835	0.620584143
DCSTAMP	0.104235934	0.265654233
DCTN2	-0.038572915	0.816579989
DCTN3	-0.238247708	0.262959324
DCTN4	0.492031289	7.88E-05
DCTN5	-0.146276341	0.520268919
DCTN6	-0.273443577	0.123902481
DCTPP1	-0.097372421	0.774084062
DCUN1D1	-0.247427869	0.222157832
DCUN1D2	0.242819209	0.215684792
DCUN1D5	-0.077181024	0.747730826
DCX	0.242981332	0.684194677
DCXR	-0.726449158	0.001784726
DDA1	-0.42589065	0.081772108
DDB1	0.035358143	0.781418662
DDB2	0.954515535	0.084442034
DDHD1	-0.026905953	0.952956034
DDHD2	-0.34430294	0.098691561
DDI2	0.397524872	0.006880123
DDIAS	0.412361192	0.063950041
DDIT4	0.898396685	0.047203268
DDIT4L	0.197619316	0.417523719
DDO	0.349631799	0.088130104
DDOST	0.407830169	0.072951624
DDR1	1.029082125	0.002001156
DDRGK1	0.208551978	0.513684677
DDX1	0.216551606	0.653942911
DDX10	-0.13875621	0.478333461
DDX11	0.393483635	0.058274197
DDX17	0.449645759	0.044768121
DDX18	0.501089264	0.016786009
DDX19A	0.231656608	0.340516155
DDX19B	0.511559132	0.005161235
DDX20	0.223498721	0.331756042
DDX21	0.022982107	0.957816002
DDX23	0.451855857	0.006545489
DDX24	-0.721981119	0.003015938
DDX25	-0.393296147	0.291211053
DDX27	0.5681755	0.009189763
DDX28	-0.266600547	0.107646206

DDX31	0.760029622	0.000507675
DDX3X	0.150019171	0.447321521
DDX3Y	0.803873682	0.245930877
DDX4	-0.059189598	0.486107177
DDX41	0.29072986	0.121791104
DDX43	-0.101777265	0.665450164
DDX46	0.532566299	0.00117745
DDX49	0.193725192	0.283716255
DDX5	0.627677657	0.003624066
DDX50	0.030935908	0.903242011
DDX51	0.019792779	0.902943459
DDX53	-0.175430923	0.088723575
DDX55	0.871434954	0.000173709
DDX56	0.74173355	0.002068027
DDX58	1.031422003	0.012001106
DDX60	1.623100744	7.88E-05
DEAF1	-0.40208512	0.024170104
DECR1	0.043071256	0.879255634
DEDD2	-0.274790073	0.168721983
DEF6	0.433416024	0.063006392
DEF8	-0.224649055	0.270838727
DEFA1	0.093583932	0.278760312
DEFA4	-0.041209839	0.731019998
DEFA5	0.070204547	0.6062359
DEFA6	-0.040463489	0.655976143
DEFB1	0.018476975	0.912632913
DEFB103B	0.151938139	0.215706597
DEFB104A	0.004127495	0.972416734
DEFB105B	0.068781616	0.521605163
DEFB106A	0.081559998	0.506700199
DEFB119	-0.01995566	0.936366256
DEFB123	0.068422903	0.61235819
DEFB125	-0.100547051	0.359588555
DEFB126	-0.00322778	0.98286235
DEFB127	-0.041308029	0.663494227
DEFB128	0.238245035	0.107462271
DEFB129	-0.144554417	0.173820085
DEFB132	-0.148405266	0.181118964
DEFB134	-0.021619263	0.879820479
DEFB4A	-0.063561051	0.622964251
DEFB4B	-0.019758866	0.878160468
DEGS1	0.150620815	0.608107078
DEGS2	-0.294137152	0.064433318
DEK	0.429889468	0.116274213
DELE1	0.202408738	0.186566312
DENND1C	0.634638286	0.052773912
DENND2A	1.416540102	0.003551012
DENND2C	-0.149250731	0.220045427

DENND2D	0.409630164	0.064172013
DENND3	0.253830323	0.55712095
DENND5A	0.116390282	0.625457691
DENND5B	-0.50537858	0.126477815
DENND6A	0.703429208	0.001009748
DENR	0.469739553	0.026629846
DEPDC1B	0.337273039	0.353410067
DEPDC4	0.060578448	0.583914788
DEPP1	0.789539963	0.093400021
DEPTOR	-0.681184809	0.012899394
DERA	0.469238549	0.003702362
DERL1	0.407038313	0.169563691
DERL2	0.449814785	0.220586083
DERL3	0.156444885	0.350472952
DES	-0.477807895	0.44673969
DESI2	1.012746125	0.004919031
DET1	-0.08410855	0.338892631
DEUP1	-0.071330809	0.54759672
DEXI	-0.208931207	0.417948411
DFFA	0.026639232	0.927976981
DFFB	-0.057491515	0.780791174
DGAT1	0.084409701	0.56677898
DGAT2	-0.218305097	0.053637776
DGAT2L6	-0.16143224	0.179519907
DGCR2	-0.01762207	0.956825161
DGCR6	-0.045088101	0.78220314
DGCR6L	-0.40394105	0.016287042
DGKA	-0.157385616	0.573791214
DGKB	-0.852004938	0.307041607
DGKD	0.495788337	0.055621986
DGKE	-1.601269447	0.002172874
DGKG	1.168156537	0.145193906
DGKH	0.178337224	0.602524331
DGKI	-0.871536728	0.059004868
DGKZ	-0.951542217	0.010927461
DGUOK	0.141460045	0.584768971
DHCR24	-2.062205215	0.001056288
DHCR7	-0.789602927	0.0017866
DHDDS	-0.01976502	0.957816002
DHDH	-0.262358433	0.187031577
DHFR	0.182223645	0.368199679
DHFR2	0.04682201	0.730938595
DHH	-0.18924705	0.246828211
DHODH	0.724696965	0.002165273
DHPS	0.289567666	0.205657208
DHRS1	0.291197382	0.188020133
DHRS11	-0.553028807	0.048587206
DHRS12	0.133629243	0.44476973

DHRS3	-0.02579057	0.966910049
DHRS4	-0.14427256	0.5208979
DHRS7	0.05020519	0.873009495
DHRS7B	-0.016210466	0.940268759
DHRS9	-0.201712216	0.321620139
DHRSX	0.354191775	0.105885379
DHTKD1	0.005888584	0.985639169
DHX15	0.696646935	0.001454069
DHX16	0.316511129	0.063208828
DHX29	-0.040528975	0.861236145
DHX30	0.035817042	0.786569291
DHX32	-0.019561986	0.959862565
DHX34	0.257578358	0.244224823
DHX35	0.667490613	0.001618895
DHX36	0.403300304	0.010555103
DHX37	0.362577249	0.018397292
DHX38	0.436981948	0.014439706
DHX57	0.504650238	0.03471813
DHX58	0.484641213	0.014591133
DHX8	0.233762621	0.193042841
DIABLO	0.35789489	0.056755453
DIAPH1	0.338321191	0.212133643
DICER1	0.278775744	0.196976608
DIMT1	0.225046799	0.422425331
DIO1	-0.090064057	0.508981429
DIP2A	0.52580418	0.013522194
DIP2C	-0.32339641	0.288509325
DIPK1B	-0.528134541	0.135123268
DIPK2A	-0.021117484	0.931560935
DIRAS1	-0.652990905	0.123801057
DIRAS2	-1.536007284	0.021293717
DIRAS3	0.850249288	0.027165261
DIS3	-0.049173501	0.872118673
DIS3L	0.204172944	0.403288078
DISP1	1.164010293	0.000187608
DISP2	-0.715559814	0.009955338
DKC1	0.642261763	0.015441473
DKK1	-0.026161943	0.971953702
DKK2	-0.387395511	0.018844974
DKK3	-0.906039609	0.017272826
DKK4	-0.122484076	0.290665805
DKKL1	-0.194459918	0.114470693
DLAT	0.091980693	0.668794145
DLC1	-0.159776045	0.725705946
DLD	0.362313297	0.067292748
DLEC1	0.379229693	0.057451491
DLG3	-0.516417194	0.194105223
DLG4	-0.979594187	0.021293717

DLG5	0.717524441	0.024149987
DLGAP1	-0.742566155	0.115683548
DLGAP2	-1.556992351	0.001776331
DLGAP3	-1.482896378	0.000511553
DLGAP5	0.64927661	0.184990188
DLK1	-0.362961737	0.512168114
DLK2	0.02498014	0.912070891
DLL1	0.575850494	0.369020321
DLL3	0.932747748	0.380462395
DLL4	0.158287177	0.62217349
DLST	0.104937063	0.581618911
DLX1	0.006679511	0.99037514
DLX2	-0.316312646	0.214706104
DLX3	-0.197217372	0.049245411
DLX4	0.012002932	0.936404425
DLX6	0.072929018	0.878058817
DMAC1	-0.280300977	0.079836405
DMAC2	-0.106986713	0.657659243
DMAC2L	0.285769654	0.215704613
DMAP1	-0.095746428	0.502848118
DMBT1	-0.002338085	0.992016034
DMBX1	0.065460317	0.737992416
DMC1	-0.083976286	0.429798231
DMGDH	0.083970161	0.674472706
DMP1	-0.018285973	0.856424995
DMRT1	-0.184711552	0.080355719
DMRT2	-0.06560192	0.827442753
DMRT3	-0.096751893	0.497470334
DMRTA1	-0.238880737	0.313708077
DMRTA2	0.158452001	0.582804387
DMRTB1	-0.106626427	0.335387051
DMTN	-1.869379908	0.003658552
DMXL1	-0.166909878	0.408664247
DMXL2	0.225039004	0.519234888
DNA2	0.192082068	0.47739712
DNAAF1	0.42512679	0.545059515
DNAAF2	0.132270977	0.394423915
DNAAF3	-0.161939224	0.513207922
DNAH10	0.102579039	0.546004746
DNAH12	0.277793902	0.409654189
DNAH3	0.139916124	0.607712692
DNAH5	0.514098116	0.06969506
DNAH7	0.437341695	0.39963402
DNAH9	0.95522724	0.029698504
DNAI1	0.659711298	0.210364638
DNAI2	0.09494385	0.582026712
DNAJA1	-0.461000154	0.055012311
DNAJA2	-0.179698963	0.23852602

DNAJA3	0.162193785	0.292461941
DNAJA4	-0.779608202	0.060056966
DNAJB1	0.575544527	0.124119955
DNAJB11	0.674679785	0.018554166
DNAJB12	-0.275190258	0.075945188
DNAJB13	0.080520035	0.592768909
DNAJB14	-0.165640062	0.41174686
DNAJB2	-0.102033923	0.721002028
DNAJB4	-0.207897077	0.449235883
DNAJB5	0.315971732	0.317915289
DNAJB6	0.369962963	0.008222173
DNAJB7	-0.058559274	0.552640677
DNAJB8	0.097774633	0.400453703
DNAJB9	0.135469063	0.208450437
DNAJC1	0.177399597	0.723824018
DNAJC10	0.315734875	0.093764507
DNAJC11	-0.179473939	0.35695735
DNAJC12	-0.140676211	0.739807844
DNAJC13	0.376102012	0.025425025
DNAJC14	0.001358495	0.994980306
DNAJC15	-0.728808019	0.14269909
DNAJC17	0.026008217	0.87184609
DNAJC18	-0.034724973	0.904207344
DNAJC19	0.239646036	0.36134822
DNAJC21	-0.188582723	0.272643265
DNAJC22	0.068028663	0.68886919
DNAJC24	0.264433171	0.15915853
DNAJC27	-0.174161647	0.523304981
DNAJC28	-0.115411433	0.422023769
DNAJC3	0.20200466	0.491069497
DNAJC30	-0.107630382	0.691624557
DNAJC5	-0.160420558	0.624020772
DNAJC5B	0.220401136	0.037405671
DNAJC5G	-0.438433475	0.006497385
DNAJC7	-0.224181187	0.118872519
DNAJC8	0.163407082	0.334308509
DNAJC9	0.042585708	0.879395967
DNAL1	-0.09968126	0.423060214
DNAL4	-0.326798204	0.066889346
DNALI1	1.561267875	0.000739219
DNASE1	0.431295908	0.017826742
DNASE1L1	-0.062006046	0.849374219
DNASE1L2	0.048540489	0.794592949
DNASE2	0.614818322	0.024436271
DNASE2B	0.116891976	0.373969966
DND1	0.12072485	0.480832489
DNER	0.054311295	0.956825161
DNHD1	0.502816327	0.025937403

DNM1	-1.34943448	0.082058452
DNM1L	-0.44552037	0.043406974
DNM3	-0.758202107	0.345154663
DNMBP	0.374847902	0.335906467
DNMT3A	0.370529266	0.043996213
DNMT3B	-0.035412373	0.821763617
DNMT3L	-0.131984956	0.234889728
DNPEP	0.375831797	0.059750851
DNPH1	-0.171448447	0.34945583
DNTT	-0.027531475	0.790780478
DNTTIP1	0.149016967	0.475052564
DNTTIP2	-0.126841432	0.430432133
DOC2A	-1.57880988	0.038331746
DOC2B	-0.865237244	0.209189667
DOCK1	-0.570379978	0.029094312
DOCK11	1.484280927	0.00010541
DOCK2	1.151019152	0.006571229
DOCK3	-1.345739214	0.066441462
DOCK4	0.487844175	0.267549543
DOCK5	-1.722967462	0.002957531
DOCK6	0.842272323	0.025596149
DOCK7	0.806503577	0.00390644
DOCK8	1.045498718	0.021047024
DOK1	0.02114764	0.928651342
DOK2	0.300851095	0.145193906
DOK3	0.363013831	0.093971499
DOK4	-0.48971331	0.039168674
DOK5	0.412848951	0.307178611
DOK6	-1.660478894	0.011596407
DOK7	-0.074642492	0.559486406
DOLPP1	0.056141086	0.843055497
DOP1B	-0.013900385	0.949549469
DOT1L	1.118858711	0.002623259
DPAGT1	0.443231621	0.06143058
DPCD	-0.116246559	0.394334383
DPEP3	-0.294835503	0.038275075
DPF3	0.71604767	0.082720016
DPH2	0.191439708	0.465576961
DPH3	0.10193255	0.650065932
DPH6	0.773264741	0.009991617
DPH7	0.544762064	0.008878668
DPM1	0.14899908	0.478648665
DPM2	0.105992433	0.670750072
DPM3	-0.069570182	0.699486223
DPP10	-1.601538218	0.017141009
DPP4	0.330685648	0.659450812
DPP7	-0.09533197	0.794214198
DPP8	-0.245569362	0.258718816

DPPA2	0.048617055	0.726874788
DPPA3	0.008330732	0.946788797
DPT	0.053361084	0.912618675
DPY19L2	0.021632138	0.941983489
DPY19L4	0.464523034	0.011198625
DPYD	0.635261505	0.127006537
DPYS	-0.079631301	0.660708583
DPYSL3	1.421367126	0.009403536
DPYSL4	-0.883525363	0.014547991
DR1	0.20908393	0.22497975
DRAM1	-0.095626051	0.847063203
DRAM2	0.677957497	0.00184649
DRAP1	-0.225011227	0.19285978
DRAXIN	0.811856528	0.22187924
DRC1	0.228187674	0.351662486
DRC7	0.247570615	0.329413397
DRD1	-0.464247577	0.04256124
DRD2	0.345634055	0.262201825
DRD3	-0.01470518	0.911351241
DRD4	0.041515472	0.897855492
DRD5	-0.388374574	0.001401204
DRG1	-0.080162327	0.780791174
DRICH1	-0.054188255	0.68189963
DROSHA	0.463761494	0.001198093
DRP2	-0.991609544	0.040414934
DSC1	-0.117946469	0.270838727
DSC2	0.367830247	0.213267269
DSC3	0.015407353	0.963194792
DSCAM	0.301045098	0.67915756
DSCAML1	-0.371900247	0.557086564
DSCC1	0.441562433	0.122238554
DSE	0.264948307	0.054716207
DSEL	1.169627454	0.020883126
DSG1	-0.033880191	0.655976143
DSG2	-0.274432539	0.705363982
DSG3	-0.001438855	0.990310157
DSG4	-0.18500339	0.183211794
DSP	-0.944540885	0.085181906
DSPP	-0.222620457	0.053470148
DST	-0.008424678	0.972003957
DSTYK	-0.267343601	0.441769769
DTD1	0.039614329	0.892604256
DTD2	-0.030046876	0.926910554
DTHD1	0.78723421	0.115025433
DTL	0.96086577	0.149383727
DTNA	0.602942594	0.235608451
DTWD1	0.434394156	0.108681693
DTWD2	-0.276213679	0.160578797

DTX1	-0.316866705	0.439088098
DTX2	0.612027449	0.005083883
DTX3	0.512058657	0.070281277
DTX3L	1.360841277	0.000204607
DTYMK	0.846087957	0.013250814
DUOX1	-0.079689831	0.396543306
DUOX2	-0.198574506	0.101927177
DUOXA1	-0.000491135	0.996803176
DUOXA2	-0.24206792	0.008271496
DUS1L	0.284317701	0.276265527
DUS2	0.156431702	0.429572074
DUS3L	0.522648639	0.048048058
DUSP10	0.543141402	0.1038076
DUSP11	0.21922526	0.10216072
DUSP12	0.600322104	0.000991006
DUSP15	0.123805306	0.390444458
DUSP16	0.354056016	0.265654233
DUSP18	0.503358425	0.069055408
DUSP19	0.040538996	0.910283756
DUSP2	0.121651325	0.558239715
DUSP21	-0.088993046	0.384852931
DUSP22	0.31533626	0.228350481
DUSP23	-0.027666988	0.922139037
DUSP26	-0.887960964	0.105918599
DUSP28	-0.099754223	0.486154507
DUSP3	-0.827148138	0.001227346
DUSP4	0.409592705	0.259243554
DUSP5	-0.040293114	0.94332102
DUSP6	1.439257248	0.007391046
DUSP7	-0.870052059	0.001009748
DUSP8	-1.355801459	0.000962015
DUSP9	-0.195649251	0.246846601
DVL1	-0.311412829	0.203243282
DVL2	0.402091302	0.070198976
DVL3	0.179857352	0.286627449
DYDC1	0.025894184	0.803596079
DYM	-0.276520007	0.076256414
DYNAP	-0.046572272	0.692614306
DYNC1H1	-0.624315531	0.001739808
DYNC1I1	-1.460697689	0.010316231
DYNC1I2	-0.261605997	0.087781129
DYNC1LI1	-0.395472702	0.034169158
DYNC1LI2	0.150072622	0.584587654
DYNC2LI1	0.496358628	0.112078161
DYNLL1	-0.200921518	0.126915641
DYNLL2	-0.371064703	0.368464473
DYNLRB1	-0.155054506	0.290822404
DYNLRB2	0.069483548	0.720000862

DYNLT1	0.981976179	0.007821977
DYNLT3	-0.536887369	0.188670982
DYRK1A	0.109327146	0.501120842
DYRK1B	-0.240552275	0.351536825
DYRK2	0.271078966	0.116341498
DYRK3	0.196337082	0.570872368
DYRK4	0.373454445	0.002856643
DZIP1	-0.031504633	0.921612349
DZIP1L	0.464499533	0.037875226
DZIP3	-0.271822452	0.52317169
E2F1	0.26139404	0.364266034
E2F2	0.216656719	0.193636372
E2F3	0.465197475	0.064680316
E2F4	0.286562155	0.172222557
E2F5	0.575427459	0.016238619
E2F7	0.200465838	0.741269852
EAF1	0.421656419	0.067292748
EAF2	0.543539676	0.105227452
EAPP	0.251334484	0.223732065
EBAG9	0.025809694	0.911948444
EBF1	0.395007883	0.351645396
EBF2	0.225739746	0.628067677
EBI3	-0.003867836	0.992016034
EBP	0.155269882	0.423953702
EBPL	0.315030658	0.078312945
ECD	-0.433202922	0.051052528
ECE2	-0.131791856	0.413190317
ECEL1	0.222756794	0.515173789
ECH1	-0.467053039	0.161642263
ECHDC1	0.490858032	0.009766183
ECHDC3	-0.066901079	0.788380786
ECHS1	-0.972061903	9.92E-05
ECI1	0.309189871	0.06529065
ECI2	0.332341886	0.218647611
ECM1	-0.034312106	0.910536149
ECM2	1.962200167	0.00183367
ECRG4	-0.089227515	0.709670769
EDA	0.030981844	0.826101558
EDA2R	0.357314473	0.660708583
EDAR	0.172098388	0.259979076
EDARADD	-0.039732596	0.746528402
EDDM3A	-0.102416533	0.426916031
EDDM3B	-0.02792741	0.869211288
EDEM1	0.328682943	0.109696733
EDEM3	0.46400171	0.00839662
EDF1	0.145576981	0.432364083
EDIL3	-3.067452304	7.67E-05
EDN2	-0.074707114	0.617800417

EDN3	-0.139606765	0.529762228
EDNRA	1.260751629	0.039899513
EDNRB	0.700610776	0.234706512
EEA1	0.345815559	0.173390271
EED	0.379610243	0.041222772
EEF1A1	0.148895827	0.319064938
EEF1A2	-1.955823611	0.006182552
EEF1AKMT1	0.007869704	0.979840621
EEF1AKNMT	0.318226527	0.132037295
EEF1B2	-0.035092432	0.878160468
EEF1D	0.563206711	0.017118008
EEF2	-0.264386581	0.207194825
EEF2K	0.644553994	0.061672093
EEF2KMT	0.098690841	0.741065302
EEFSEC	0.147323067	0.469757823
EEPD1	1.039065518	0.024157203
EFCAB1	0.112648441	0.763718293
EFCAB11	-0.101876396	0.531227892
EFCAB12	0.391498731	0.403872871
EFCAB13	0.250129579	0.17349014
EFCAB5	0.060323164	0.733642627
EFCAB6	0.004597181	0.991101527
EFCAB8	0.0067459	0.956825161
EFCC1	0.272659133	0.206294371
EFHC2	0.188892134	0.582941588
EFHD1	-1.241034729	0.000978538
EFHD2	-0.147869878	0.65000111
EFNA1	0.195617116	0.477248291
EFNA2	0.204876033	0.318895907
EFNA3	-0.484615634	0.255407492
EFNA4	-0.136878287	0.660250094
EFNA5	-1.044116777	0.023797504
EFNB1	0.291236912	0.51415788
EFNB2	-0.860871914	0.142733654
EFNB3	-0.049418833	0.904792329
EFS	0.767468129	0.054300635
EFTUD2	0.661082815	0.026331477
EGF	0.237724502	0.334004274
EGFL6	0.21646176	0.624020772
EGFL7	-0.120774364	0.580309198
EGFLAM	0.437317972	0.044128583
EGFR	1.521774873	0.071387892
EGLN1	0.226328551	0.380564954
EGLN3	-0.126988357	0.370192134
EGR1	1.06033741	0.026220292
EGR2	-0.062084348	0.819093264
EGR3	-0.57352164	0.108773057
EGR4	-0.362039069	0.339412764

EHBP1	-0.23028514	0.250939062
EHD1	-0.148742811	0.274456375
EHD2	0.293848046	0.659552619
EHD3	-1.618913726	0.008098686
EHD4	0.793555134	0.122433204
EHMT2	-0.068776471	0.727903728
EI24	0.046755798	0.872374833
EID1	-0.269330356	0.173093618
EID2	-0.351409382	0.056009706
EID2B	-0.01325244	0.946782009
EIF1	-0.180105942	0.196995482
EIF1AX	-0.199980534	0.295585953
EIF1AY	0.191861435	0.731136744
EIF1B	-0.128519929	0.499195822
EIF2A	0.320923667	0.226306722
EIF2AK2	0.611773252	0.009889747
EIF2AK3	0.622226984	0.055494413
EIF2B1	0.427179097	0.010673905
EIF2B2	0.31632015	0.095531284
EIF2B3	0.334743407	0.033747886
EIF2B4	0.457271327	0.00898722
EIF2B5	0.241819265	0.116072246
EIF2D	0.48436126	0.012784284
EIF2S1	0.209664722	0.356070019
EIF2S2	0.103277029	0.710236734
EIF2S3	0.063880265	0.739670021
EIF2S3B	-0.054830105	0.57428374
EIF3A	-0.239324803	0.132468097
EIF3CL	-0.177373813	0.477434776
EIF3D	0.238984524	0.329795252
EIF3F	0.072609381	0.68280633
EIF3G	0.167230271	0.583914788
EIF3H	0.094009648	0.537165089
EIF3I	0.211684437	0.418760753
EIF3J	-0.249688331	0.268197687
EIF3K	-0.292942775	0.078955881
EIF3M	0.154835435	0.562821065
EIF4A2	0.041658399	0.839105888
EIF4A3	0.664094195	0.000980529
EIF4E1B	-0.144953361	0.245189575
EIF4E2	0.194026242	0.163401884
EIF4EBP1	0.811073665	0.048726015
EIF4EBP2	-0.189171794	0.295361674
EIF4ENIF1	-0.094699827	0.518288881
EIF4G1	0.368411674	0.024305591
EIF4G2	-0.145279191	0.277618337
EIF4H	0.225361866	0.288325171
EIF5	0.037073491	0.869806475

EIF5A2	-0.915449472	0.038266069
EIF5B	-0.230588654	0.207180851
EIF6	-0.014840589	0.959862565
EIPR1	0.057627275	0.699632125
ELAC2	0.262875216	0.187919562
ELANE	-0.068667316	0.627227825
ELAVL1	0.411345527	0.024618793
ELAVL3	-0.936934494	0.242695127
ELAVL4	-0.754348862	0.177287889
ELF1	0.637335416	0.022445836
ELF2	0.377130397	0.023487605
ELF3	0.08486488	0.46665328
ELF5	-0.071817203	0.425828964
ELFN1	0.157803435	0.74773532
ELK3	0.611143319	0.100714877
ELL	0.495259049	0.101785225
ELL2	0.093946571	0.838917532
ELL3	-0.0794069	0.653101302
ELMO2	-0.193733619	0.317475293
ELMO3	0.042095836	0.741065302
ELMOD1	-0.176438971	0.873454177
ELMOD2	0.548812069	0.062277687
ELMOD3	0.327147879	0.027639103
ELN	0.657052177	0.074870519
ELOA	0.1586398	0.453656837
ELOA2	-0.090747361	0.473826225
ELOA3	-0.129462799	0.558236109
ELOB	-0.103703601	0.593914303
ELOC	-0.209803405	0.198583413
ELOF1	0.309577961	0.269682994
ELOVL2	1.208571416	0.183662823
ELOVL3	-0.147330249	0.196134898
ELOVL4	-0.7872418	0.147623721
ELOVL6	-0.105389575	0.717407272
ELP1	0.346865139	0.071584801
ELP3	0.050059499	0.794680068
ELP4	0.311279688	0.261850437
ELP5	-0.032433111	0.921943398
ELP6	0.345454597	0.040364084
ELSPBP1	-0.102736601	0.37548222
EMB	0.048781904	0.894416316
EMC1	0.035176158	0.868433712
EMC10	-0.346319457	0.011930446
EMC2	-0.059920915	0.782828938
EMC3	0.055900551	0.815673957
EMC4	-0.601260727	0.029094312
EMC7	-0.335956051	0.23287476
EMC8	0.162539117	0.41529825

EMC9	0.130120836	0.457590223
EMCN	0.397805068	0.106075036
EMD	0.093362305	0.732977979
EMG1	0.585678079	0.001039348
EMID1	0.247470304	0.455789497
EMILIN1	0.49780806	0.232049464
EMILIN2	0.447766007	0.350402797
EMILIN3	0.435404022	0.122247398
EML4	0.995576487	0.00343336
EML5	-0.353892208	0.181545073
EML6	-0.444630186	0.415499507
EMP1	2.189127447	0.003011379
EMP2	0.177275057	0.67780975
EMP3	1.518640372	0.059425494
EMSY	0.273937919	0.236795966
EMX1	-0.103938014	0.546091808
EN1	0.359855375	0.172992444
EN2	0.137486983	0.414462192
ENAM	-0.236068026	0.060370505
ENDOG	-0.226569894	0.271825342
ENDOV	0.145584496	0.350077294
ENHO	-1.357459158	0.00183367
ENKUR	1.498597927	0.000589737
ENO1	0.480780153	0.032546902
ENO2	-0.371965584	0.405907035
ENO3	0.209137035	0.225020885
ENOPH1	0.262247737	0.23287476
ENOSF1	-0.058842293	0.905495936
ENOX1	-0.450162167	0.151108631
ENOX2	0.141567545	0.524908613
ENPEP	1.137468779	0.026003628
ENPP1	0.595067123	0.295121535
ENPP2	-1.785846067	0.004142884
ENPP3	-0.032805486	0.888190176
ENPP5	-0.782376615	0.250588662
ENPP6	-0.217672119	0.671089464
ENPP7	-0.115280843	0.343433863
ENSA	-0.147039863	0.515432943
ENTHD1	-0.145770622	0.073808099
ENTPD2	-0.005901387	0.987717747
ENTPD3	-0.893629785	0.000557559
ENTPD4	0.555398995	0.319997474
ENTPD5	0.254184071	0.302467936
ENTPD6	-0.304878941	0.223143157
ENTPD7	0.139794041	0.63605684
ENY2	0.423682276	0.026003628
EOGT	-0.267312251	0.412776329
EOMES	0.199853873	0.23214561

EP300	-0.108070659	0.636740808
EP400	0.498330788	0.011584437
EPAS1	-0.029363269	0.96451093
EPB41	-0.138924361	0.739670021
EPB41L1	-1.340206739	0.001939871
EPB41L2	0.092211007	0.724508351
EPB41L3	-1.113808499	0.130017478
EPB41L4A	0.867189583	0.006301603
EPB41L4B	-1.928373712	0.005624542
EPB41L5	-0.077547736	0.846092035
EPB42	-0.143981065	0.503991656
EPC1	0.208417811	0.374191921
EPC2	0.254624284	0.165398162
EPCAM	-0.129518353	0.314686643
EPDR1	-0.118531656	0.739759003
EPG5	0.054151151	0.849863963
EPHA1	-0.007744898	0.948142628
EPHA2	-0.094360124	0.878185547
EPHA3	0.583645275	0.444756716
EPHA4	-0.097940829	0.857879493
EPHA5	-1.644105059	0.001392079
EPHA7	-0.543146325	0.164496102
EPHA8	-0.059818466	0.686036634
EPHB1	-0.194529852	0.730363414
EPHB2	-0.199289871	0.650417176
EPHB3	0.734022608	0.008845735
EPHB4	0.701713948	0.09677388
EPHB6	-2.072183576	0.000690887
EPHX1	-0.692129619	0.005161235
EPHX2	-0.045134323	0.886755045
EPHX3	-0.012678	0.927976981
EPHX4	-0.950258718	0.009612941
EPM2A	0.196252154	0.361019878
EPN2	0.321014788	0.449734088
EPN3	0.013513124	0.967049328
EPO	0.002732634	0.986387457
EPS15	-0.530620962	0.052433755
EPS8	0.614647639	0.051448573
EPS8L1	-0.078761139	0.593412031
EPS8L2	-0.098689249	0.718996095
EPS8L3	-0.277656608	0.067570002
EPSTI1	0.701037597	0.024864763
EPX	-0.088500168	0.453795581
EPYC	0.120231652	0.420941497
EQTN	-0.047344827	0.774355077
ERAL1	0.04504067	0.903468454
ERAP2	0.200344453	0.679713931
ERAS	0.068728218	0.568456067

ERBB2	0.447022523	0.281430558
ERBB3	-0.098217887	0.894865376
ERBB4	-0.719243263	0.312995875
ERC1	0.195322531	0.357866979
ERC2	-1.710677545	0.006188209
ERCC1	-0.12383921	0.622639969
ERCC2	-0.338172676	0.326932275
ERCC3	0.32157151	0.043326827
ERCC4	0.590175035	0.005088041
ERCC6	-0.274621716	0.30366548
ERCC6L	-0.158795586	0.132832354
EREG	0.220876928	0.557806519
ERG28	-0.515035864	0.133080948
ERGIC2	-0.366356567	0.200694292
ERGIC3	0.308515979	0.168038754
ERH	0.636473478	0.007845807
ERI1	0.408892186	0.037650934
ERI2	-0.084404551	0.70915631
ERICH1	0.403623954	0.033854934
ERICH3	-0.675122428	0.114155544
ERICH4	-0.035155121	0.764778035
ERICH5	-0.065834891	0.625902395
ERICH6	-0.073655869	0.520194039
ERLEC1	-0.124942598	0.58264073
ERLIN1	0.365687162	0.297135787
ERLIN2	0.476129487	0.017646437
ERMAP	0.414051938	0.038596937
ERMARD	0.866239093	0.000389052
ERN1	0.316799097	0.055487058
ERO1A	0.067499791	0.856424995
ERO1B	0.131118541	0.401711068
ERP27	0.175730747	0.326058883
ERP29	0.187792811	0.198053836
ERP44	0.423681363	0.01120723
ERRFI1	0.011112905	0.986614651
ERV3-1	1.262086524	0.001927913
ERVFRD-1	-0.159495346	0.20948538
ERVMER34-1	0.035661297	0.830717269
ERVV-1	-0.107683804	0.215892279
ERVW-1	-0.273055689	0.070059059
ESAM	0.318522621	0.320683845
ESCO1	0.351025171	0.028452842
ESCO2	0.24675742	0.593336567
ESD	-0.09693386	0.780438484
ESF1	0.171126638	0.425384977
ESM1	0.559128005	0.159863387
ESPL1	0.462318994	0.016124829
ESPNL	-0.115898039	0.512915077

ESR1	-0.003666257	0.982318638
ESR2	0.243782222	0.173314313
ESRP1	-0.077880658	0.329674449
ESRP2	0.084693336	0.539746
ESRRA	0.075245902	0.701241054
ESRRB	0.010557296	0.939960521
ESS2	-0.056144305	0.847725163
ESX1	-0.224287816	0.039200246
ESYT2	0.110853849	0.645711398
ESYT3	-0.675700022	0.016817157
ETAA1	0.333520238	0.107274495
ETDA	-0.029428336	0.809020028
ETDB	0.029487401	0.863600504
ETFA	-0.038276996	0.807436219
ETFB	-0.006774357	0.970647222
ETFBKMT	0.055670467	0.711091662
ETFDH	-0.089844237	0.728011946
ETFRF1	0.031814426	0.884394993
ETHE1	-0.304622106	0.345154663
ETNK1	0.344143118	0.052182985
ETNK2	-0.302514655	0.053314874
ETNPPL	-2.795396287	0.005405116
ETV1	1.677168544	0.00064817
ETV2	0.017589007	0.872447647
ETV3L	-0.149877472	0.080202281
ETV4	0.175799437	0.6712973
ETV5	0.318223162	0.286021893
ETV6	1.069663445	0.005162429
EVA1A	-0.306910384	0.17102908
EVA1B	0.081848852	0.799493951
EVA1C	0.269920023	0.515150095
EVC2	0.439207951	0.130017478
EVI2A	0.379128935	0.436247993
EVL	0.057621383	0.826802803
EVPL	-0.127913808	0.430786831
EVX1	-0.156963334	0.14799748
EVX2	-0.119575949	0.500451938
EXD1	-0.02686034	0.821868934
EXD3	0.074885909	0.650427205
EXO1	0.881636443	0.067292748
EXO5	0.105459133	0.655380323
EXOC1	0.247813964	0.128669959
EXOC2	0.077673879	0.70248201
EXOC3	0.15207343	0.440160896
EXOC3L1	-0.03599508	0.878631732
EXOC3L2	-0.070277957	0.731984105
EXOC4	0.84566084	0.000309879
EXOC5	-0.105371175	0.653942911

EXOG	0.429857568	0.072637113
EXOSC1	-0.185192633	0.4689651
EXOSC10	0.578718791	0.00183367
EXOSC2	0.528191133	0.002712845
EXOSC3	0.147088405	0.631258654
EXOSC4	0.135833628	0.422691316
EXOSC5	-0.333002883	0.263766329
EXOSC6	-0.067807757	0.649684927
EXOSC7	0.381471255	0.026701201
EXOSC8	0.821605361	0.003687745
EXPH5	-0.212187225	0.443670516
EXT1	-0.437721051	0.436014625
EXT2	0.178652472	0.41922487
EXTL1	-1.30690538	0.004240643
EXTL2	0.291665892	0.26242907
EXTL3	0.092952864	0.693365203
EYA1	-0.17434377	0.846242672
EYA2	0.779411705	0.131673102
EYA3	0.151634627	0.460608508
EYA4	1.373137598	0.044310474
EZH1	0.00548955	0.989997727
EZH2	1.972646516	0.001258004
EZHIP	0.346341706	0.172026648
EZR	-0.446294166	0.140823867
F10	-0.139587981	0.355140928
F11R	0.275660228	0.646942576
F12	0.005013066	0.973630919
F13A1	1.316132055	0.079742987
F13B	-0.076763496	0.374228363
F2	-0.23134861	0.016226911
F2R	0.96202757	0.057834887
F2RL1	-0.247190886	0.573387796
F2RL2	0.346192029	0.693365203
F2RL3	0.028860562	0.893231278
F3	0.218677491	0.759850228
F5	0.018506807	0.923498499
F7	0.056103763	0.662753428
F8	0.186368476	0.327357676
F8A2	-0.116130311	0.524810007
F9	-0.130305743	0.195488245
FA2H	-1.744594148	0.011678018
FAAH2	0.07044072	0.70551044
FAAP24	-0.11922289	0.33581858
FABP1	-0.109573628	0.324631071
FABP12	-0.05262346	0.506429489
FABP2	-0.094528142	0.25676815
FABP3	-1.453055885	0.006281327
FABP4	-0.067252692	0.632430202

FABP5	0.285541164	0.447940525
FABP6	-0.226235814	0.313353465
FABP7	1.152265471	0.084597529
FABP9	-0.012336017	0.915561258
FADD	0.282735049	0.227110312
FADS1	-0.305832164	0.125857027
FADS2	0.188265835	0.507144787
FADS3	-0.091307022	0.740003676
FADS6	-0.340609236	0.019064046
FAF1	-0.084296766	0.839105888
FAF2	0.289633221	0.107626615
FAH	0.129318077	0.625902395
FAHD1	-0.082015608	0.691358344
FAHD2A	-0.339894767	0.081092156
FAHD2B	-0.038199012	0.887269342
FAIM2	-1.338386875	0.037689346
FAM102B	0.051359552	0.924763137
FAM107A	-0.579228692	0.140000239
FAM110A	0.193772229	0.207205603
FAM110B	0.367302671	0.173690738
FAM110D	-0.03297528	0.780202244
FAM111A	2.333021057	2.32E-05
FAM111B	0.769223701	0.081109342
FAM114A1	0.569541182	0.368580248
FAM114A2	0.384331542	0.045879041
FAM117A	-0.066521382	0.624029608
FAM117B	-0.349456238	0.31754923
FAM118A	0.304987188	0.441582171
FAM118B	0.133248978	0.579913621
FAM120A	0.43124838	0.013611479
FAM120AOS	-0.090213431	0.714731176
FAM120B	0.388976375	0.010026957
FAM120C	0.669209475	0.00227739
FAM124A	-0.378871211	0.060199973
FAM124B	0.266023395	0.091239708
FAM126A	0.626489303	0.153311985
FAM126B	-0.130414172	0.620047289
FAM131A	-0.344276916	0.250197691
FAM131B	-0.455778509	0.301570429
FAM135A	0.868692649	0.001192251
FAM135B	-0.111729266	0.811305444
FAM136A	0.222512786	0.232643514
FAM13B	-0.276004041	0.123229751
FAM151A	-0.244432702	0.10943235
FAM151B	0.152210795	0.432216082
FAM153A	-1.96896666	0.02100609
FAM155B	-0.313642915	0.109575774
FAM156B	0.537933573	0.016899346

FAM160A2	0.221835971	0.416394904
FAM160B2	0.45654394	0.020517538
FAM161B	-0.181564126	0.189348765
FAM162A	0.038221543	0.904256812
FAM163A	-0.287372322	0.044170471
FAM167A	0.029927708	0.892641383
FAM167B	-0.213418011	0.252786332
FAM170A	-0.134662654	0.323258085
FAM171A2	-0.969668999	0.012967984
FAM171B	-1.154723172	0.07197268
FAM172A	0.149431002	0.276841337
FAM174A	-0.071309216	0.760420863
FAM174B	-0.383644274	0.040414934
FAM177A1	-0.201149851	0.223362507
FAM177B	0.092697823	0.620741982
FAM180A	0.352592677	0.426612208
FAM181A	0.0611246	0.592400311
FAM181B	0.457168687	0.035192281
FAM184A	0.038696527	0.93451723
FAM184B	0.41472868	0.250647627
FAM187B	-0.043921112	0.756015246
FAM189A2	0.60684807	0.280274355
FAM189B	-0.222140062	0.21403625
FAM200A	0.435263315	0.016644122
FAM204A	-0.18484368	0.318130405
FAM207A	0.107033373	0.575581907
FAM209A	0.059318047	0.72777771
FAM20B	-0.558487348	0.015405659
FAM20C	1.343162773	0.028452842
FAM210B	-0.106986378	0.756811437
FAM214B	-0.217141559	0.406099532
FAM216A	-0.629415383	0.00525333
FAM216B	-0.080720426	0.670187191
FAM217A	-0.193988215	0.028921846
FAM217B	-0.707687889	0.093636651
FAM219B	0.000857429	0.996803176
FAM221A	0.349802667	0.254297669
FAM222A	-0.180337712	0.632786331
FAM222B	0.441760238	0.032595502
FAM227B	-0.036996403	0.722807353
FAM229A	0.047167321	0.847927611
FAM234A	0.360855516	0.106838727
FAM234B	-0.844232916	0.057494446
FAM236D	0.115003153	0.540536449
FAM241A	-0.199620325	0.360124848
FAM241B	-0.241431082	0.040927029
FAM243A	-0.03150068	0.784418087
FAM32A	0.355250154	0.149257703

FAM3B	-0.064049742	0.659647977
FAM3C	0.44060975	0.144254129
FAM3D	-0.085915424	0.413793226
FAM43A	-0.32446567	0.517107433
FAM43B	-0.096986965	0.657224321
FAM47A	-0.181599132	0.262617666
FAM47B	-0.167078733	0.471742439
FAM50A	0.247620236	0.235163956
FAM50B	-0.729479877	0.003505554
FAM53B	-0.339132318	0.062359498
FAM53C	-0.098976223	0.331569694
FAM71A	0.217267235	0.302106724
FAM71B	-0.048873979	0.754207571
FAM71C	0.010346678	0.931748888
FAM71D	-0.0380117	0.642315919
FAM71F1	0.245141869	0.149621879
FAM72C	0.28333265	0.151903986
FAM76B	0.646238194	0.000635034
FAM78A	0.113700387	0.583914788
FAM78B	0.025289048	0.960583721
FAM81A	-0.919710834	0.001056288
FAM81B	-0.075422577	0.622639969
FAM83A	-0.020896216	0.833439613
FAM83C	0.068309477	0.610901519
FAM83E	0.05666261	0.764678612
FAM83F	0.003432957	0.987148725
FAM83G	-0.198846017	0.399753106
FAM83H	-0.426095528	0.006185744
FAM89A	0.033335792	0.931777778
FAM8A1	-0.059436104	0.874048428
FAM90A1	-0.066796836	0.830365605
FAM91A1	0.665092093	0.007364098
FAM98A	-0.461868305	0.064735077
FAM98B	-0.006308302	0.982318638
FAM98C	-0.177720392	0.497672332
FAM9B	-0.105820462	0.247648009
FANCA	1.010448404	0.013194164
FANCD2	0.847998354	0.040348424
FANCD2OS	-0.072873132	0.33581858
FANCE	0.108994133	0.510565481
FANCG	0.37829189	0.078394754
FANK1	0.196178363	0.422691316
FAP	0.317798432	0.708608221
FAR1	-0.150094583	0.4022945
FAR2	-0.058989483	0.90380542
FARP1	-0.02005944	0.942522673
FARS2	0.249171844	0.06244516
FARSA	-0.021709715	0.916630472

FARSB	-0.043807511	0.782419818
FAS	0.431310806	0.269592402
FASLG	0.069693508	0.511285816
FASN	-0.686707497	0.001148355
FASTK	0.149713644	0.462801577
FASTKD1	0.455929378	0.093443295
FASTKD3	0.23882478	0.106008246
FAT1	0.304881082	0.510592076
FAT2	-0.215774732	0.191623519
FAT4	-0.255362074	0.514120748
FATE1	0.070613068	0.506263613
FAU	-0.207060095	0.261936453
FAXC	-0.675683155	0.165431419
FAXDC2	-0.470288591	0.186566312
FBH1	0.066893835	0.795866349
FBLIM1	-0.080077392	0.85360537
FBLN5	0.070772749	0.920850924
FBLN7	-0.088169286	0.772253466
FBN1	-0.283241391	0.731977162
FBN2	-0.0192936	0.989421806
FBN3	0.509518108	0.12804237
FBP1	0.637269041	0.003820513
FBP2	-0.019855794	0.897050949
FBRS	-0.010087279	0.970477208
FBRSL1	-0.201744581	0.348982569
FBXL12	-0.077894459	0.820792146
FBXL14	-0.063598115	0.844347734
FBXL18	-0.15955971	0.393143635
FBXL2	-0.791840313	0.004500154
FBXL20	-0.455319242	0.033678519
FBXL22	-0.113823727	0.290587504
FBXL3	-0.237321667	0.250700001
FBXL4	0.177420838	0.338352185
FBXL6	-0.038226044	0.865012559
FBXL7	0.123980446	0.712611767
FBXO17	0.450972339	0.25431304
FBXO2	-0.810896669	0.010397685
FBXO21	-0.025825443	0.930110116
FBXO24	0.034445609	0.730363414
FBXO25	-0.392470536	0.091863175
FBXO27	-0.904220074	0.001181813
FBXO28	0.109713024	0.640068791
FBXO3	-0.135273237	0.605883997
FBXO30	0.43014929	0.160076093
FBXO32	0.635971087	0.267609207
FBXO33	-0.574708674	0.043883036
FBXO34	0.174281378	0.544236806
FBXO38	0.141228643	0.34163193

FBXO39	0.247042135	0.192559557
FBXO4	0.240071714	0.291652566
FBXO40	-0.251682191	0.084913957
FBXO41	-1.142057179	0.002287614
FBXO42	0.675868882	0.009194554
FBXO44	-0.746748691	0.019251941
FBXO5	0.57608233	0.058095758
FBXO6	-0.031044581	0.866703923
FBXO7	-0.315188959	0.322585459
FBXO8	0.362750557	0.137948025
FBXO9	-0.061938008	0.784105431
FBXW11	-0.261191731	0.310556884
FBXW12	-0.056828292	0.681406422
FBXW2	0.416028372	0.029502776
FBXW4	-0.127109186	0.665622234
FBXW5	-0.147613427	0.576779548
FBXW7	-1.285529826	0.019659172
FBXW8	0.706438918	0.006574157
FBXW9	0.327128699	0.071166412
FCAR	0.148311246	0.355183166
FCER1A	-0.134796257	0.308749255
FCER1G	1.818077468	0.002899844
FCER2	0.07098194	0.702957543
FCF1	0.282508265	0.291228984
FCGBP	2.474242835	0.000331368
FCGR1A	0.391006656	0.457779061
FCGR3A	0.292244458	0.266895823
FCGRT	0.776460805	0.007315104
FCHSD1	0.374752474	0.092545137
FCHSD2	-0.126656462	0.736476972
FCMR	0.223581978	0.038331746
FCN1	-0.066604768	0.479544747
FCN2	-0.082808571	0.743883616
FCN3	-0.147071649	0.394342731
FCRL1	-0.129333731	0.25191183
FCRL2	-0.06320383	0.680402738
FCRL3	-0.018252737	0.861734293
FCRL4	-0.078245794	0.345154663
FCRL5	0.004248017	0.980526403
FCRLB	0.023007068	0.895078219
FCSK	0.098963238	0.570358311
FDCSP	-0.126685749	0.183435993
FDFT1	-0.300530788	0.165779238
FDPS	0.205187682	0.233246575
FDX1	0.241310491	0.154319751
FDX2	-0.427340126	0.027411858
FEM1B	-0.444879766	0.009403536
FEM1C	0.782138052	0.012784284

FEN1	0.114020023	0.642073436
FER	0.166554557	0.298854812
FERD3L	-0.198428654	0.154668207
FERMT1	0.7732644	0.344495657
FERMT2	-0.12754662	0.668755927
FERMT3	0.365227697	0.097582498
FES	0.340293936	0.023815176
FETUB	-0.123025502	0.274018269
FEV	-0.329794184	0.003563462
FEZ1	-0.49853591	0.176007134
FEZ2	0.127799187	0.581641615
FEZF2	-1.707545773	2.32E-05
FFAR1	-0.227571944	0.1225386
FFAR2	-0.032329362	0.754465661
FFAR3	-0.094062996	0.646187802
FFAR4	0.223329359	0.468893197
FGA	-0.061188558	0.486301245
FGB	-0.125063968	0.139113867
FGD1	0.123602988	0.59692935
FGD2	0.4886449	0.111085479
FGD4	0.126618495	0.701353149
FGD5	0.328705474	0.024792405
FGF10	-0.038759772	0.861636252
FGF11	1.22911067	0.00764822
FGF12	-1.139114712	0.14269909
FGF14	-0.430352622	0.624418177
FGF16	-0.181426275	0.059191332
FGF17	-0.32575043	0.373437343
FGF18	-0.300433997	0.074282574
FGF19	-0.11342902	0.407537778
FGF20	0.184295849	0.25431304
FGF21	-0.234358768	0.049074918
FGF22	-0.146805739	0.253676848
FGF23	-0.005067473	0.97286117
FGF3	-0.009743573	0.939044278
FGF4	-0.364647321	0.011765584
FGF5	-0.439123589	0.329369557
FGF6	-0.05924424	0.563638129
FGF7	0.053640162	0.78917966
FGF8	0.007075328	0.967317802
FGF9	-0.465899929	0.053470148
FGFBP1	-0.12518665	0.211600856
FGFBP2	0.384050656	0.516953978
FGFBP3	0.190233167	0.34556292
FGFR1	-0.077109859	0.730363414
FGFR1OP2	0.410564612	0.029454101
FGFR2	-1.326146337	0.010201971
FGFR3	-0.620247861	0.373388265

FGFR4	0.077870032	0.587103195
FGFRL1	-0.280284398	0.3422411
FGG	-0.011669015	0.910373242
FGL1	-0.081859029	0.416681284
FGL2	1.646713798	0.00114661
FH	0.039720809	0.873085293
FHDC1	0.230835651	0.601588011
FHIT	0.118831935	0.515618724
FHL2	-0.956636327	0.051351295
FHL3	0.981212718	0.035695554
FHL5	-0.174853975	0.631638721
FHOD1	0.078154322	0.821835029
FIBIN	1.204009482	0.007625398
FIBP	0.084296932	0.601656239
FICD	0.224622446	0.166149474
FIG4	-0.050391067	0.827469652
FIGLA	0.000691594	0.996189598
FIGN	0.974053946	0.00297406
FIGNL1	0.565111708	0.012000691
FILIP1	0.104142922	0.723376553
FILIP1L	0.021856139	0.948142628
FIP1L1	0.140529844	0.752202506
FIS1	0.019583565	0.928866131
FITM1	-0.17060215	0.298915854
FITM2	-0.116672684	0.640752919
FIZ1	-0.136257895	0.46925675
FJX1	1.292995418	0.023342763
FKBP10	1.227200885	0.038900113
FKBP11	-0.283556922	0.298266643
FKBP1C	0.025797776	0.872956667
FKBP2	-0.411820229	0.098350432
FKBP3	-0.489921577	0.006497556
FKBP4	-0.186727349	0.225020885
FKBP7	0.195810669	0.548270461
FKBP8	0.546048475	0.048302042
FKBP9	0.649022001	0.076022756
FKBPL	-0.085846111	0.570047614
FKRP	-0.260449995	0.248627195
FLACC1	0.435362353	0.148616265
FLCN	0.148354799	0.617883109
FLI1	0.345240118	0.151903986
FLII	0.443051238	0.132230039
FLNC	-0.165757752	0.862276842
FLOT1	0.161883023	0.582941588
FLOT2	0.529219582	0.024719845
FLRT1	-0.066908287	0.897867328
FLRT2	-1.232001581	0.008424217
FLRT3	-0.105474501	0.809211438

FLT1	-0.345788303	0.523555752
FLT3	-0.292841517	0.022239
FLT4	0.314285033	0.08658043
FLVCR1	0.236121317	0.320347182
FLVCR2	-0.173136919	0.062214697
FLYWCH1	0.091493714	0.632748122
FMNL1	-0.122432503	0.777420731
FMNL2	-0.269567976	0.47059038
FMNL3	0.615821552	0.05679073
FMO1	-0.108161476	0.433725746
FMO2	-0.120664447	0.574759224
FMO3	-0.027640905	0.903766356
FMO4	0.389667718	0.099273698
FMO5	0.132830265	0.568985288
FMOD	1.415440822	0.008737673
FMR1	-0.071708289	0.781131164
FMR1NB	-0.021955954	0.824382088
FN1	1.252608942	0.140119197
FN3K	-0.491708954	0.062347184
FNBP1	-0.145093257	0.736206294
FNBP4	0.697391486	0.005365281
FNDC10	-0.605775761	0.025834777
FNDC11	0.097515794	0.261777605
FNDC3B	0.930490005	0.080024958
FNDC4	-0.266249903	0.380095352
FNDC7	0.38448102	0.198073975
FNDC8	-0.249380646	0.069500937
FNDC9	-0.885344621	0.054057069
FNTA	0.183135301	0.306928724
FOCAD	-1.075964883	0.011635622
FOLR1	0.228414766	0.295774062
FOLR2	0.486312487	0.023534883
FOLR3	-0.18313081	0.114153909
FOSL1	-0.591482983	0.364266034
FOSL2	0.241444777	0.696913847
FOXA1	-0.054843229	0.707824035
FOXA2	-0.067704426	0.595889777
FOXA3	-0.494346741	0.001544789
FOXB1	-0.058975051	0.685949422
FOXC1	0.276466682	0.433974236
FOXC2	-0.712539661	0.186670547
FOXD1	0.272626042	0.507246453
FOXD2	0.014871499	0.926890103
FOXD3	0.001478967	0.993945285
FOXD4	0.075162898	0.840636705
FOXD4L1	-0.06130246	0.785361295
FOXD4L3	-0.109341439	0.693033257
FOXE1	-0.111562582	0.521837798

FOXE3	0.04700855	0.825954884
FOXF1	0.308832976	0.120199333
FOXF2	0.98292689	0.006729601
FOXH1	0.056735291	0.632855398
FOXI1	0.045950033	0.726307053
FOXI2	-0.081075282	0.479498653
FOXJ1	0.621139076	0.181063637
FOXJ2	-0.059984996	0.752135041
FOXK2	-0.120710088	0.452308799
FOXL1	-0.715101271	0.152650935
FOXL2	-0.259087361	0.180154024
FOXL2NB	-0.028272628	0.897484194
FOXM1	0.778977792	0.151142131
FOXN1	-0.193286715	0.098656545
FOXN2	0.149799062	0.741183923
FOXN4	-0.022908749	0.910153865
FOXO1	0.529481183	0.057699866
FOXO3	0.608323589	0.009169897
FOXO4	-0.585185919	0.144769636
FOXO6	0.333165696	0.181758916
FOXP1	-0.369945085	0.119624812
FOXP3	0.031650816	0.821666052
FOXQ1	0.041712258	0.826878373
FOXR1	-0.226080306	0.023266695
FOXR2	0.145345672	0.167786992
FOXRED1	0.263412704	0.140684406
FOXS1	-0.163800582	0.511594042
FPGS	0.016547727	0.938370209
FPR2	-0.046041211	0.66207657
FPR3	1.499538166	0.003551012
FRA10AC1	-0.50626295	0.033823837
FRAS1	-0.371635703	0.379999971
FRAT1	0.065945729	0.59150484
FRAT2	0.00668977	0.985962589
FREM1	0.644660771	0.066166083
FREM2	1.437817396	0.060011766
FREM3	-0.643222276	0.293719062
FRG1	0.238290863	0.061198429
FRK	-0.189237357	0.189506063
FRMD1	-0.189245319	0.110456596
FRMD4A	-0.408206554	0.206755147
FRMD5	-0.598123797	0.171793831
FRMD7	0.026586395	0.798602742
FRMD8	0.424877862	0.070480847
FRMPD1	-0.065563584	0.567695781
FRMPD3	0.714594807	0.023596613
FRRS1	0.291819337	0.307195901
FRS2	0.167049027	0.583220485

FRS3	-0.34665461	0.039200246
FRY	-0.233670918	0.706303011
FRZB	1.020896267	0.173820085
FSCB	-0.095030018	0.339356809
FSCN1	-0.227579114	0.575647993
FSCN2	-0.337002272	0.119298934
FSCN3	0.061402935	0.590067083
FSD1	-0.738061326	0.011311993
FSD2	0.061168091	0.673940677
FSHB	-0.237623475	0.045937888
FSHR	-0.343147047	0.01535789
FSIP1	-0.574208172	0.038556673
FST	0.001164448	0.998811869
FSTL1	0.694910003	0.350573936
FSTL3	-0.487008248	0.239588827
FTCD	-0.257151074	0.037650934
FTH1	0.967968672	0.016013049
FTHL17	-0.288521435	0.067633212
FTL	-0.177621876	0.674828435
FTMT	-0.114268882	0.25431304
FTO	-0.218453257	0.068124574
FTSJ1	0.057487783	0.885192487
FTSJ3	0.727933937	0.008556158
FUBP1	0.795946187	0.00044063
FUCA1	0.336425176	0.395751097
FUCA2	0.302808273	0.512007635
FUNDC1	-0.230122624	0.120584598
FUNDC2	0.022558324	0.91915956
FUOM	-0.049461548	0.893241153
FURIN	-0.311126205	0.28242945
FUS	0.568008589	0.005962991
FUT1	-0.153440211	0.29113245
FUT10	0.003484679	0.992016034
FUT11	-0.128352923	0.559687792
FUT2	0.096899157	0.372787239
FUT3	0.256954742	0.111086676
FUT5	0.089004696	0.649335092
FUT7	0.245911864	0.018371667
FUT8	-0.79479506	0.018435704
FUT9	-0.973261424	0.142826069
FUZ	-0.070827004	0.717346538
FXN	-0.186306148	0.305375238
FXR2	-0.662255982	0.001038868
FXYD1	-1.77655478	0.017791812
FXYD4	-0.12836335	0.402775257
FYB1	1.276134636	0.019789186
FYB2	-0.128272513	0.193810368
FYCO1	0.396229559	0.119298934

FYN	1.069351723	0.000839788
FZD1	0.356160093	0.153468999
FZD10	-0.012368804	0.94493417
FZD2	-0.111137268	0.768354761
FZD3	0.908101619	0.098615853
FZD4	0.061989784	0.833194058
FZD5	0.308922034	0.344996338
FZD6	-0.210963282	0.726551291
FZD7	0.422995174	0.445033163
FZD8	-0.392180053	0.102055123
FZD9	-0.233609379	0.037188291
G0S2	0.358294883	0.390808971
G2E3	0.390785467	0.153312271
G3BP1	0.399213126	0.006691815
G3BP2	-0.414627771	0.016362396
G6PC2	-0.174339222	0.196976608
G6PD	-0.399403018	0.095051152
GAA	0.54149343	0.058993008
GAB1	0.414859923	0.320716701
GAB2	0.34309128	0.329732103
GABARAP	-0.262318364	0.158088917
GABARAPL1	-0.275239043	0.268542643
GABARAPL2	-0.2825014	0.316898088
GABBR2	-1.460522324	0.172784937
GABPA	0.707617297	0.001660138
GABPB2	0.180078878	0.473735539
GABRA1	-1.82752172	0.005780236
GABRA3	-1.610387243	0.027018172
GABRA4	-2.213572283	0.004122462
GABRA6	-0.129362597	0.135332976
GABRB1	-1.419618578	0.060836467
GABRB2	-2.111405367	0.006880123
GABRB3	-0.736700742	0.061016234
GABRD	-1.600017006	0.001788518
GABRE	-0.385295724	0.433577409
GABRG1	-3.389555606	0.000389052
GABRG2	-2.437659118	0.019659172
GABRG3	-1.236855077	0.000760638
GABRP	0.056611962	0.583914788
GABRR2	0.23652045	0.157140014
GAD1	0.128416281	0.872956667
GAD2	-1.241902673	0.006576658
GADD45A	0.8395785	0.07393545
GADD45G	0.373662366	0.358491591
GADD45GIP1	-0.280046034	0.246636816
GADL1	-0.0658179	0.547307448
GAK	0.202219667	0.288877482
GAL	0.077097314	0.789882925

GAL3ST1	-0.251580203	0.229933371
GAL3ST2	-0.211315806	0.200496037
GAL3ST3	-0.091488573	0.679023431
GAL3ST4	1.021070747	0.000314598
GALC	-0.162048483	0.609035847
GALE	0.171021989	0.22280711
GALK1	0.152977233	0.340720019
GALM	0.520494002	0.032640805
GALNT1	0.270199542	0.582945807
GALNT11	0.047451621	0.863600504
GALNT12	0.063682435	0.766009292
GALNT13	0.518954118	0.418052228
GALNT15	0.425276097	0.259077956
GALNT17	-2.079892874	0.001320049
GALNT18	-0.667186846	0.012866514
GALNT3	-0.080102777	0.83809315
GALNT5	-0.183440148	0.789965157
GALNT6	-0.498572951	0.010259208
GALNT7	0.107826418	0.752870608
GALNT8	-0.558978442	0.218897625
GALNTL6	-0.384312195	0.031179777
GALP	0.073334083	0.587088938
GALR1	0.877899486	0.057329499
GALR2	-0.031788768	0.835359151
GALR3	-0.056608709	0.815978446
GAMT	-0.254736563	0.041222772
GAN	-0.306716816	0.240533047
GANAB	0.503296984	0.138539258
GANC	-0.286204035	0.577387987
GAPDH	-0.074029932	0.669970192
GAPDHS	-0.134602614	0.227303532
GAPT	0.60131163	0.102956472
GAPVD1	0.352091128	0.007676985
GAR1	0.166161392	0.399038792
GARNL3	-0.779061612	0.005965654
GAS1	0.534784528	0.095988755
GAS2	-0.227931973	0.355403974
GAS2L1	0.490841922	0.014126133
GAS2L2	0.033767455	0.827347756
GAS2L3	1.202950974	0.017720052
GAS7	-0.147857359	0.866703923
GAST	-0.06737889	0.700015813
GATA3	-0.063350239	0.736206294
GATA4	-0.079721702	0.610213815
GATA5	-0.178672008	0.105046429
GATA6	-0.831162623	0.116972907
GATAD1	0.405335089	0.133619905
GATAD2A	0.527051948	0.020285134

GATAD2B	0.078848317	0.722085968
GATB	0.030267476	0.915446555
GATD1	0.138468012	0.622964251
GATD3A	0.328656583	0.074473662
GATM	0.137358186	0.787173374
GBA2	0.104664327	0.673087029
GBE1	1.192096661	0.014988328
GBF1	-0.511894075	0.036926773
GBGT1	0.31089326	0.209189667
GBP1	1.747736232	0.002267206
GBP2	2.119479474	0.00010541
GBP3	0.981780388	0.06009886
GBP4	0.657492922	0.201701899
GBP5	0.362030615	0.122327439
GBP6	0.142017178	0.200496037
GBP7	2.272352424	0.000366288
GBX2	0.319439596	0.137568345
GC	0.138463566	0.201671817
GCA	-0.082543326	0.696471369
GCC1	0.110190617	0.414394125
GCC2	-0.43929208	0.073083677
GCDH	0.223308901	0.229823961
GCFC2	0.272043718	0.151947457
GCG	-0.078386897	0.375141671
GCGR	-0.071939643	0.513684677
GCH1	-0.180811406	0.434487402
GCHFR	-0.013536977	0.956825161
GCK	-0.027530402	0.84046247
GCKR	-0.110692946	0.486154507
GCLC	-0.183440938	0.298993003
GCLM	-0.145477041	0.630540343
GCM1	-0.066244316	0.529207785
GCM2	-0.246556401	0.15553997
GCN1	0.446963151	0.027490144
GCNA	0.215129671	0.271652673
GCNT1	-0.200091623	0.588301474
GCNT2	-0.01277755	0.953293342
GCNT3	-0.091064597	0.363424132
GCNT4	-0.924351788	0.010927461
GCSAM	-0.043805088	0.729361605
GCSAML	-0.017871758	0.915817139
GCSH	0.105172587	0.6062359
GDA	-2.077899735	0.00863371
GDAP1	-0.422635575	0.347845274
GDAP2	-0.115937391	0.291228984
GDE1	-0.731533334	0.036926773
GDF1	-0.029799729	0.94629917
GDF10	-0.437413851	0.015414012

GDF15	0.234156915	0.714262147
GDF2	-0.237193043	0.065789373
GDF3	-0.080214192	0.368114726
GDF5	-0.241319369	0.224170519
GDF7	-0.157583913	0.2491227
GDF9	0.076682703	0.658552671
GDI1	-0.256346996	0.104811761
GDI2	-0.258765102	0.266765546
GDNF	0.009214386	0.973662488
GDPD1	-0.445987261	0.398558553
GDPD3	0.360489499	0.14161703
GDPD4	0.01782611	0.942981424
GDPD5	-0.098216915	0.479580596
GDPGP1	-0.183815037	0.153046427
GEM	0.719248319	0.120281267
GEMIN2	0.293069899	0.025589823
GEMIN4	0.216258704	0.421610984
GEMIN5	0.228131884	0.335608162
GEMIN6	0.028282475	0.884083488
GEMIN7	-0.075676373	0.758628178
GEMIN8	0.746617557	0.006055699
GET3	-0.097291951	0.685402367
GET4	0.233840898	0.243517574
GFER	-0.016623504	0.939579338
GFI1	-0.04518691	0.670085809
GFI1B	0.073296354	0.568327491
GFM1	0.122608559	0.537662679
GFM2	-0.189971327	0.375141671
GFOD2	0.14844303	0.55266491
GFPT2	0.483608049	0.375141671
GFRA1	-0.756097527	0.139173829
GFRA2	-0.514290555	0.17102908
GFRA3	0.054198223	0.700541866
GFRA4	-0.180721472	0.283439618
GFRAL	-0.150030953	0.063760341
GGA1	0.156921276	0.486640129
GGA2	0.068683581	0.743927746
GGCX	0.290919103	0.196742655
GGH	0.366921442	0.316349868
GGN	-0.000718971	0.997942116
GGNBP2	0.119058872	0.380612457
GGT1	0.148778443	0.429824416
GGT2	0.42442934	0.051261944
GGT7	-0.172371612	0.430432133
GGTLC1	-0.22624943	0.182834325
GH1	-0.080811449	0.529207785
GHITM	-1.13927135	0.002419881
GHR	0.12069915	0.516380268

GHRH	0.128513606	0.324365155
GHRHR	-0.117840773	0.302520363
GHSR	-0.041854905	0.789310457
GID4	-0.328369351	0.056305323
GID8	0.385277336	0.016372941
GIGYF1	0.830914795	0.011850098
GIMAP1	0.232810777	0.214121064
GIMAP4	0.782788096	0.020624238
GIMAP7	0.239257054	0.372787239
GIMAP8	0.402126334	0.048981178
GIN1	0.177091848	0.373052958
GINS1	0.759147167	0.078983306
GINS2	0.425108654	0.205465654
GINS4	0.118164095	0.562498279
GIP	-0.1027704	0.425384977
GIPC1	-0.35923407	0.075465366
GIPC2	-0.228066926	0.184841778
GIPC3	0.061601445	0.847248255
GIPR	0.005874748	0.973903933
GIT2	0.199270043	0.121234364
GJA1	-1.070293295	0.106805963
GJA10	-0.106541178	0.49163682
GJA3	-0.024711981	0.817473461
GJA4	0.320494339	0.108546904
GJA5	-0.110017024	0.520908636
GJA8	-0.157692867	0.119298106
GJB1	-0.479789825	0.054009246
GJB2	0.447917896	0.090678601
GJB3	-0.283574119	0.03810472
GJB4	-0.169128771	0.148703181
GJB5	-0.159778476	0.34166678
GJC2	-0.532630128	0.011718351
GJD2	-0.691073501	7.88E-05
GJD3	0.22012775	0.305343847
GJE1	-0.112911061	0.23210837
GK	0.508389939	0.031768004
GK2	0.04876263	0.707354681
GKAP1	-0.203962304	0.40452116
GKN1	0.002548302	0.97776514
GKN2	-0.010320494	0.931727556
GLA	0.428363053	0.165999884
GLB1L	0.395792504	0.065420304
GLCE	-0.14103501	0.557745885
GLDC	1.09392362	0.047203268
GLDN	-0.031854519	0.967441815
GLE1	0.431724058	0.003603178
GLI1	-0.047882847	0.833439613
GLI2	0.0633923	0.907503103

GLI3	0.389688569	0.368538531
GLI4	-0.012853299	0.960585602
GLIPR1	-0.350507695	0.724508351
GLIPR1L1	-0.064074939	0.630540343
GLIPR2	0.602892443	0.278161431
GLIS1	-0.514995465	0.131673102
GLIS2	0.279486588	0.281639201
GLMN	0.436474613	0.065878376
GLMP	0.379301979	0.173831365
GLO1	-0.091243018	0.762174089
GLOD4	0.407334839	0.033401935
GLP1R	-0.010899679	0.954084441
GLP2R	-0.353035416	0.215608625
GLRA2	-0.196024447	0.569471762
GLRA3	-1.397182307	0.025650687
GLRB	-0.890224898	0.035918142
GLRX	0.093144401	0.723362559
GLRX2	-0.170524791	0.499335429
GLRX5	-0.105829304	0.508190857
GLS	-0.920713011	0.006058653
GLS2	-1.545428008	0.000391899
GLT1D1	-1.488246009	0.007966232
GLT6D1	-0.114204598	0.23210837
GLT8D2	-0.401068309	0.203901234
GLTP	-0.224213183	0.42819277
GLUD1	-0.606537396	0.035747902
GLUD2	-0.581342611	0.040927029
GLUL	0.508352391	0.386477298
GLYAT	0.033839845	0.752135041
GLYATL2	0.254143061	0.576779548
GLYCTK	0.193601096	0.156706486
GLYR1	-0.313564002	0.081442916
GM2A	-0.035304161	0.884394993
GMCL1	0.105049584	0.57448557
GMDS	0.02867869	0.906729
GMEB1	0.326140475	0.114470693
GMEB2	0.093878102	0.591128693
GMFB	-0.051118496	0.847725163
GMFG	1.256967779	0.018595078
GMIP	0.51751989	0.120299523
GML	-0.046869225	0.728307088
GMNN	0.855665382	0.001931311
GMPPA	0.350034748	0.16728951
GMPPB	0.17226206	0.341916447
GMPR	0.578050285	0.053470148
GMPR2	0.102457472	0.651014997
GMPS	0.18032369	0.448441679
GNA11	-0.49572941	0.063729998

GNA12	0.645487621	0.006497385
GNA13	0.45566101	0.020624238
GNA14	-0.346074466	0.334004274
GNA15	0.31478537	0.202580776
GNAI1	-0.629990514	0.024436271
GNAI2	0.094798655	0.670274351
GNAI3	0.494013714	0.184841778
GNAO1	-1.183442713	0.253968739
GNAQ	-0.340425224	0.141254251
GNAT1	0.02537291	0.893831905
GNAT2	-0.138967005	0.225594593
GNAT3	-0.191364674	0.068186904
GNB1	-0.215307115	0.08031761
GNB1L	-0.112925645	0.492595587
GNB2	0.449996732	0.01622654
GNB3	0.009721027	0.966982882
GNB4	1.318361603	0.001275687
GNB5	-0.250690449	0.320594067
GNG11	0.075005315	0.829989834
GNG12	0.737113316	0.304115063
GNG13	-0.12123047	0.600699957
GNG3	-2.857226044	0.003461043
GNG5	0.404696458	0.200618747
GNG5P2	0.686999046	0.111086676
GNG7	-0.135483179	0.892541412
GNG8	-0.143927159	0.343725271
GNGT1	-0.153742617	0.083993053
GNGT2	-0.014046236	0.910283756
GNL1	0.058119412	0.795866349
GNL2	0.77811451	0.009946858
GNL3	0.72436513	0.012222983
GNLY	0.158144352	0.46925675
GNMT	0.380279554	0.044027708
GNPAT	0.604545133	0.00893861
GNPDA1	0.278462883	0.134628395
GNPDA2	0.205880036	0.497981199
GNPNAT1	0.02975831	0.941983489
GNPTAB	-0.323554546	0.200428396
GNPTG	0.04926007	0.833863145
GNRH1	0.375945487	0.156935118
GNRH2	0.049799812	0.702025588
GNRHR	-0.096105693	0.350428516
GNS	0.522678467	0.033687405
GOLGA1	0.58160582	0.005391307
GOLGA2	0.167004844	0.523015082
GOLGA3	0.243144672	0.288380428
GOLGA4	0.255754959	0.305716021
GOLGA5	0.013752981	0.958571031

GOLGA6L9	0.513178676	0.111085479
GOLGA7	-0.352440327	0.152189121
GOLGA8H	0.380096527	0.184777407
GOLIM4	0.417490622	0.170784931
GOLM1	0.655566475	0.000653805
GOLPH3	0.05901151	0.779217884
GOLPH3L	0.350252207	0.05199819
GOLT1A	-0.143247618	0.349284814
GOLT1B	0.027680664	0.924928069
GON4L	0.600639835	0.000511553
GON7	0.072044198	0.62217349
GOPC	0.229645978	0.234572666
GORAB	0.439757454	0.006902788
GORASP1	0.176045353	0.325895121
GORASP2	-0.065276249	0.792979636
GOSR1	0.267548603	0.035197777
GOSR2	0.288545209	0.076949066
GOT1	-2.157976894	9.92E-05
GOT1L1	-0.134615226	0.158304741
GOT2	-0.921495287	0.003278541
GP1BA	0.10360538	0.580193623
GP2	-0.030840571	0.782002897
GP5	0.010160059	0.951100629
GP9	-0.043745639	0.752870608
GPA33	-0.057163151	0.677996486
GPAA1	0.015374943	0.944001602
GPALPP1	0.017096647	0.942141247
GPAM	-0.776799516	0.063240636
GPANK1	0.118561043	0.293632899
GPAT2	0.012041786	0.927780014
GPAT3	-0.23655452	0.135013087
GPAT4	0.62245899	0.000167994
GPATCH1	0.318929087	0.023029329
GPATCH11	0.47749279	0.005964724
GPATCH2	0.489862652	0.019185909
GPATCH2L	0.126492752	0.729055026
GPATCH3	0.299322177	0.162310231
GPATCH4	0.177862653	0.463201043
GPBP1	0.306143229	0.23287476
GPBP1L1	-0.078761067	0.657371825
GPC1	0.05322889	0.885622833
GPC2	0.319517595	0.163607707
GPC4	0.704578188	0.2583263
GPC5	-0.876658082	0.297642305
GPC6	0.525559148	0.520908636
GPCPD1	1.30240701	7.88E-05
GPD1	0.049631037	0.869597022
GPD1L	-0.355007737	0.145961015

GPHA2	0.123181783	0.394342731
GPHB5	-0.127769787	0.123278599
GPI	0.284108868	0.098350432
GPIHBP1	-0.644538131	0.020496695
GPKOW	0.101094747	0.597842196
GPLD1	-0.42262356	0.142277196
GPM6B	0.307771097	0.645711398
GPN1	0.444712408	0.232643514
GPNMB	1.168210114	0.014650406
GPR101	0.023709454	0.835312849
GPR119	-0.156919201	0.174548225
GPR12	-0.529200142	0.24372963
GPR132	0.0622183	0.796968624
GPR135	0.257375847	0.40452116
GPR137B	0.480521454	0.175372054
GPR139	0.300847475	0.237359755
GPR141	0.159598145	0.162801961
GPR142	-0.012488306	0.948704827
GPR143	-0.430221501	0.001392079
GPR146	0.125092017	0.597100313
GPR148	-0.101613963	0.469757823
GPR15	-0.133732352	0.159223076
GPR150	-0.19117871	0.159109939
GPR151	-0.039779615	0.726447094
GPR152	-0.167385977	0.304273853
GPR153	0.085606037	0.723581067
GPR156	0.080496949	0.65000111
GPR157	-0.028957464	0.846369748
GPR158	0.273680922	0.714722654
GPR160	-0.041630983	0.704780487
GPR162	-0.551995863	0.34866536
GPR171	0.004986421	0.968064507
GPR173	0.837200518	0.000186736
GPR174	0.003421123	0.986230241
GPR176	-0.357829079	0.305518308
GPR179	-0.230429759	0.299633127
GPR18	-0.049393814	0.601513992
GPR180	0.016866225	0.956931468
GPR182	-0.056870152	0.590635292
GPR183	1.222608578	0.037049711
GPR19	0.224392065	0.503991656
GPR20	0.032206182	0.871201192
GPR21	-0.295696836	0.298993003
GPR22	-1.349218363	0.025528022
GPR25	-0.136894817	0.348378671
GPR26	-0.842151678	0.009991617
GPR27	-0.824115348	0.014697052
GPR3	0.058676837	0.860002363

GPR31	-0.010292235	0.919974005
GPR35	0.164477857	0.237564766
GPR37	-1.119093152	0.215378909
GPR37L1	-0.073776942	0.930110116
GPR39	-0.112687733	0.78856472
GPR4	0.362057798	0.251731458
GPR42	0.115805391	0.401032649
GPR45	-0.088815016	0.516550683
GPR50	-0.103448719	0.34166678
GPR52	0.096745916	0.562599025
GPR55	0.007732527	0.957700639
GPR6	-0.30108901	0.037188291
GPR61	-0.555644122	0.014933685
GPR62	-0.11153114	0.385117466
GPR65	1.512831819	0.001044988
GPR68	0.016695605	0.924928069
GPR75	0.267842565	0.274320551
GPR78	-0.149148158	0.17152569
GPR82	0.465073219	0.037046054
GPR83	-0.852784295	0.068203075
GPR84	0.339281568	0.083570457
GPR87	-0.132844564	0.23960216
GPR88	-0.284149989	0.150029586
GPRC5A	-0.442466748	0.422962991
GPRC5B	-0.268586592	0.750617451
GPRC5C	0.176208356	0.117781718
GPRC5D	-0.186536469	0.138593482
GPRC6A	-0.003574272	0.97681392
GPRIN1	-0.224260979	0.363733058
GPRIN2	-0.282409802	0.067292748
GPRIN3	0.377933232	0.30699339
GPS1	-0.289504891	0.044369981
GPS2	0.160883894	0.375141671
GPSM2	0.570921277	0.286021893
GPSM3	0.759799179	0.002950583
GPT	-0.27651923	0.105842098
GPT2	-0.239788273	0.272643265
GPX1	0.773384014	0.033322986
GPX3	0.016037164	0.971953247
GPX5	-0.098413454	0.178134407
GPX6	-0.01617086	0.878322956
GPX7	0.750953194	0.049245411
GRAMD1A	0.508316261	0.037319472
GRAMD1B	-0.87687238	0.174004226
GRAMD1C	0.018191241	0.981452955
GRAMD4	0.29747395	0.220045427
GRAP	0.243210017	0.114260365
GRAP2	0.180440152	0.252826729

GRB10	0.378109894	0.41174686
GRB14	0.025892801	0.96051513
GRB2	0.118303805	0.55266491
GRB7	-0.06718099	0.540939089
GREB1	-0.055346934	0.89921021
GREM2	-0.452416647	0.003564988
GRHL1	0.152537921	0.344235354
GRHL2	-0.024283984	0.832314164
GRHL3	-0.024390832	0.925006412
GRHPR	-0.144081889	0.432364083
GRIA1	0.446526108	0.46525882
GRIA2	-0.062394565	0.957700639
GRIA3	0.16296341	0.760373199
GRID2	1.16672511	0.106919187
GRIK1	-0.217237458	0.724307904
GRIK2	-0.157050262	0.860002363
GRIK3	1.799217323	0.001784726
GRIK4	0.572532983	0.06856857
GRIK5	0.423553428	0.3184098
GRIN1	-3.451925161	0.00148582
GRIN2B	-1.844868833	0.033445756
GRIN2C	-1.42194095	0.000689186
GRIN2D	0.240916984	0.247896619
GRIN3A	-0.564584121	0.081109342
GRIPAP1	-0.13379515	0.665587965
GRK1	-0.114918402	0.335758076
GRK2	-0.365833543	0.172991578
GRK3	-0.535879377	0.340516155
GRK4	-0.02393447	0.936740169
GRK5	-0.116035841	0.668792253
GRK6	-0.558655648	0.005365281
GRK7	0.15424624	0.234531755
GRM2	-0.88343193	0.013851792
GRM3	-2.439715465	0.002884046
GRM4	-0.222624842	0.072390938
GRM6	-0.373855031	0.048424982
GRM7	-0.784875431	0.012552229
GRN	0.750802925	0.090828268
GRP	-0.022283692	0.895006181
GRPEL1	0.21344362	0.369866211
GRPEL2	0.290669909	0.347223044
GRPR	-0.044015581	0.953036745
GRSF1	0.033554309	0.869808289
GRTP1	-0.210421872	0.166695267
GRWD1	0.088691419	0.811594347
GSC	0.305483319	0.163552869
GSC2	-0.171677852	0.278481075
GSDMA	-0.00235949	0.990583302

GSDMC	0.092297711	0.347293069
GSDME	0.629053356	0.008254144
GSE1	-0.115039369	0.713113471
GSK3A	-0.508722731	0.007543692
GSK3B	-0.062040446	0.728879752
GSKIP	-0.268191401	0.305065484
GSN	0.065935866	0.884920673
GSPT1	0.012999869	0.945980127
GSPT2	0.053046995	0.82445066
GSR	0.132020318	0.439914357
GSS	0.245925101	0.323217852
GSTA1	-0.115086579	0.508340807
GSTA4	0.039932362	0.869597022
GSTA5	-0.09666568	0.55924953
GSTM1	-0.112642579	0.453656837
GSTM2	0.646272733	0.028452842
GSTM3	-0.637831731	0.010803655
GSTM4	0.134234892	0.650417176
GSTM5	-1.559409504	0.009689545
GSTO1	-0.180518547	0.510565481
GSTO2	-0.398752681	0.015093776
GSTP1	-0.163256375	0.556314926
GSTT1	-0.760874475	0.067292748
GSTT2	-0.236189597	0.572166691
GSTZ1	0.174018653	0.151903986
GSX1	0.167497697	0.456166285
GSX2	0.313084593	0.17779306
GTDC1	-0.371496888	0.089872783
GTF2A1L	0.829197272	0.046047778
GTF2B	0.201047085	0.101947989
GTF2E1	0.177537057	0.290650106
GTF2E2	0.200556879	0.330630871
GTF2F1	0.185926573	0.228531369
GTF2H2	0.775854805	0.078394754
GTF2H3	0.459623156	0.049376562
GTF2H5	-0.165223877	0.376450387
GTF2I	0.981078106	0.000948193
GTF2IRD1	0.606973307	0.048142336
GTF2IRD2	1.200613083	0.000556242
GTF3A	0.189267428	0.182130477
GTF3C1	0.168514477	0.389435147
GTF3C2	0.484956571	0.024397625
GTF3C3	0.315238022	0.178405462
GTF3C4	0.289093739	0.337711262
GTF3C6	0.302246453	0.201748507
GTPBP1	0.176049705	0.299213796
GTPBP10	0.304753482	0.052310127
GTPBP2	0.60044945	0.009786616

GTPBP3	0.080659957	0.715944482
GTPBP4	-0.253190549	0.317354392
GTPBP6	0.205439294	0.273065695
GTPBP8	0.193211314	0.45454339
GTSF1	-0.034865974	0.726883729
GTSF1L	-0.18148286	0.049276408
GUCA1A	-0.203484852	0.053575397
GUCA1B	0.176581412	0.259979076
GUCA1C	-0.055971912	0.530886986
GUCA2A	0.053399532	0.749573888
GUCA2B	-0.140868129	0.114071721
GUCD1	0.316808586	0.15015411
GUCY1A1	-0.377864851	0.546625793
GUCY1B1	-0.995141248	0.145411601
GUCY2C	-0.158036151	0.149717662
GUCY2D	-0.161506325	0.20573308
GUCY2F	-0.127136972	0.236050436
GUF1	0.104719814	0.760378549
GUSB	1.123023608	0.002244587
GXYLT2	-0.151373009	0.811789132
GYG1	-0.013012004	0.968725515
GYPA	-0.107148089	0.34504044
GYPB	-0.037775199	0.739400011
GYS1	0.106505233	0.795995458
GYS2	0.052931663	0.652822208
GZF1	0.199059715	0.222661425
GZMA	0.215798361	0.250700001
GZMB	0.017087446	0.866370176
GZMH	-0.219211593	0.196209899
GZMK	0.156044725	0.263241701
GZMM	0.074751309	0.767223379
H6PD	0.558873278	0.040293711
HAAO	-0.089607833	0.65039546
HABP2	-0.128802145	0.21659255
HABP4	-1.062369373	0.003270886
HACD1	-0.502744611	0.125990883
HACD2	-0.245684117	0.377806357
HACD3	-1.162069757	0.01050896
HACD4	0.052655804	0.847502786
HACE1	-0.160695087	0.613850833
HACL1	-0.104700758	0.596311373
HADHA	0.015356392	0.947624019
HADHB	-0.163370921	0.317519922
HAGH	-0.969341647	0.00055525
HAGHL	-0.191657293	0.46166905
HAL	0.002866496	0.98200225
HAMP	0.932630364	0.080062376
HAND1	-0.00188926	0.990417736

HAND2	0.075992916	0.7527455
HAO1	-0.097307617	0.321988625
HAP1	0.867672155	0.020530152
HAPLN1	-0.148906481	0.802533362
HAPLN2	-1.368378547	0.011653303
HAPLN3	0.119798678	0.729872694
HAPLN4	-1.458644175	0.001620311
HARBI1	0.032409224	0.82820301
HAS1	-0.129805051	0.291211053
HAS2	2.040325782	0.000627774
HAS3	-0.026741858	0.910685398
HASPIN	0.546640406	0.105478834
HAT1	0.715001225	0.009260952
HAUS2	0.081528624	0.75726135
HAUS3	0.227733872	0.211634607
HAUS6	0.461713676	0.11849221
HAVCR1	0.062718013	0.486107177
HAVCR2	0.968616727	0.006942795
HAX1	0.036464151	0.835312849
HBA1	-0.046673124	0.931748888
HBB	-0.192694646	0.222935337
HBD	0.028673593	0.765669964
HBE1	0.167377498	0.371103788
HBEGF	-0.011501473	0.982765512
HBG1	-0.121462763	0.424124501
HBP1	0.406659193	0.016088268
HBQ1	-0.148861848	0.486018117
HBZ	-0.134264813	0.095978963
HCAR2	-0.036913269	0.821868934
HCCS	-0.393583034	0.34935078
HCFC1	0.059742238	0.75582128
HCFC1R1	-0.593206183	0.016106148
HCFC2	0.363624472	0.04776679
HCLS1	1.423735643	0.00899166
HCN1	-2.600459637	0.013999648
HCN2	-2.097442875	0.001655438
HCN3	0.034276442	0.878160468
HCN4	-0.164920298	0.494314628
HCRT	-0.179703118	0.198827786
HCRTR1	0.070657309	0.571895321
HCRTR2	-0.014123722	0.901377776
HCST	0.411875925	0.162233439
HDAC1	1.022915671	0.003420022
HDAC10	-0.049705624	0.828325392
HDAC11	-0.808709703	0.002557694
HDAC3	0.484171088	0.006607673
HDAC4	0.218354287	0.240957104
HDAC5	-0.740014034	0.023286399

HDAC6	0.363851869	0.017230109
HDAC7	0.417326786	0.104998976
HDAC8	0.305326135	0.039616406
HDAC9	0.05504415	0.868668407
HDC	-0.053397939	0.776650846
HDDC2	-0.571425602	0.046756571
HDDC3	-0.097640511	0.55712095
HDGFL2	0.458140857	0.14269909
HDGFL3	-0.834084674	0.002267206
HDHD2	0.345209557	0.065307895
HDHD3	0.214558046	0.41174686
HDHD5	0.392511547	0.093938626
HEATR3	0.515696426	0.005714159
HEATR6	0.279115076	0.166957481
HEBP1	-0.160813365	0.574451759
HEBP2	0.196569828	0.568553857
HECA	-0.238347773	0.173390271
HECTD1	-0.00754023	0.973903933
HECTD2	0.002967529	0.992886227
HECTD3	0.369040125	0.103390817
HECTD4	-0.373586007	0.182057768
HECW1	-2.134826714	0.001392079
HECW2	-1.250443864	0.006329299
HELB	0.304904311	0.140577811
HELLS	1.044653005	0.027015966
HELQ	0.445403981	0.011978751
HEMK1	0.139649295	0.256956799
HENMT1	-0.392015072	0.094419727
HEPACAM	0.296379678	0.666171783
HEPACAM2	-0.070648972	0.584951716
HEPH	-0.45645195	0.351077713
HERC1	-0.673218833	0.028118418
HERC2	-0.077318294	0.712525829
HERC5	1.08772425	0.009134728
HERC6	0.698195674	0.063422605
HERPUD1	0.074678121	0.833439613
HERPUD2	0.349017256	0.063240636
HES1	0.734610562	0.065307895
HES6	0.017063537	0.960926694
HESX1	0.117673008	0.240723714
HEXA	0.789825669	0.004750798
HEXB	0.953709286	0.005704957
HEXD	-0.051834761	0.775328942
HEXIM1	-0.057077958	0.762526857
HEXIM2	0.028905475	0.823214676
HEY1	1.634368012	0.000987762
HEY2	0.583833983	0.274320551
HEYL	0.448064071	0.181953357

HFE	0.322733607	0.152801898
HGD	0.027512065	0.847063203
HGF	0.687252763	0.127744707
HGFAC	-0.079572269	0.646942576
HGH1	0.094764621	0.475052564
HGS	-0.217481051	0.393143635
HHAT	0.098084097	0.564973097
HHATL	-1.854296501	0.003140901
HHEX	0.256924431	0.170127874
HHIP	-2.560911666	0.000269441
HHIPL1	-0.606350319	0.019127748
HHLA2	-0.138697207	0.062772093
HIBADH	0.283020027	0.162538859
HIBCH	0.637231241	0.008046009
HIC2	0.335422053	0.098905266
HID1	-1.429915961	0.00117745
HIF1AN	-0.427198061	0.049245411
HIF3A	0.154002157	0.778517241
HIGD1B	0.021555754	0.927302731
HIGD1C	0.202466718	0.115969619
HIGD2A	-0.130345449	0.642440544
HILPDA	2.061361401	0.00170082
HINFP	-0.018873286	0.913796501
HINT1	-0.221754357	0.129525902
HINT2	0.046130254	0.787690301
HINT3	-0.694420568	0.029502776
HIP1R	-0.137780182	0.790313334
HIPK1	0.07659676	0.709670769
HIPK3	0.144714684	0.372273957
HIPK4	-0.018895651	0.903309166
HIRA	-0.029807617	0.923043057
HIRIP3	0.231461527	0.187526036
HIVEP1	-0.208814093	0.326633542
HIVEP2	-1.042513021	0.004981702
HIVEP3	-0.053935027	0.915271601
HJURP	0.61078579	0.152650935
HJV	-0.053922967	0.581336771
HK1	-0.508810662	0.031641787
HK3	0.164580468	0.274434723
HKDC1	0.006094292	0.990215424
HLA-A	0.635675037	0.034098511
HLA-DMA	1.157829978	0.004256114
HLA-DOA	0.598980971	0.050739896
HLA-DOB	0.278000386	0.173820085
HLA-DPB1	1.359800099	0.004828369
HLA-DQA1	0.251624362	0.5556347
HLA-DQA2	0.668177121	0.061246294
HLA-DQB1	-0.218827809	0.232654739

HLA-DQB2	0.324317933	0.034963918
HLA-DRA	1.924125237	0.03139257
HLA-DRB1	0.555309084	0.166635128
HLA-DRB5	0.251349691	0.845746964
HLA-E	0.711077999	0.017141009
HLA-G	0.71223678	0.001056288
HLF	-2.035976184	0.021312972
HLTF	0.251591844	0.301376959
HLX	0.439549036	0.021293717
HM13	0.597803632	0.014623959
HMBOX1	0.471289868	0.044274241
HMBS	0.243615749	0.155226851
HMCES	-0.225514749	0.240438022
HMCN1	0.203496846	0.761514684
HMG20A	0.007270617	0.971953247
HMG20B	0.269783316	0.327004897
HMGA1	-0.165941186	0.732792678
HMGA2	-0.116396062	0.868709798
HMGB1	0.792052362	0.0104977
HMGB2	1.237697879	0.00117745
HMGB3	-0.006629591	0.986045302
HMGB4	-0.087047652	0.285713688
HMGCLL1	-0.44964026	0.043065801
HMGCR	-0.164649331	0.604369251
HMGCS2	-0.07657016	0.398558553
HMGN1	0.52648164	0.06107631
HMGN5	0.03472344	0.91394031
HMHB1	0.023575354	0.903309166
HMMR	0.641022768	0.074604609
HMOX1	1.50820955	0.007879685
HMSD	-0.043682069	0.891478922
HMX1	0.093568921	0.682704301
HMX3	-0.16301926	0.401711068
HNF1A	0.036948944	0.79266965
HNF1B	0.08941228	0.412099167
HNF4G	0.631791817	0.057828053
HNMT	1.001405034	0.001699001
HNRNPA0	0.510714857	0.007782903
HNRNPA1	1.128390301	0.000962015
HNRNPA2B1	1.079689618	0.000386743
HNRNPA3	0.0593413	0.802201313
HNRNPAB	0.805750211	0.019561103
HNRNPC	0.419451676	0.080969794
HNRNPD	0.522175257	0.005980982
HNRNPH1	0.920898746	0.000311644
HNRNPH3	0.093086916	0.71233889
HNRNPLL	0.361787614	0.102811615
HNRNPM	0.213353349	0.237564766

HNRNPU	0.344531932	0.050414179
HNRNPUL1	0.325018462	0.111143759
HOGA1	-0.103390536	0.499086044
HOMER1	-1.342070897	0.008304461
HOMER2	-0.299793113	0.530178547
HOMEZ	0.162902235	0.322226755
HOOK1	-1.408011369	0.013611171
HOOK2	0.333394826	0.304274139
HOOK3	-0.028093864	0.886030695
HORMAD1	-0.126743648	0.203901234
HORMAD2	0.01715226	0.885791283
HOXA1	0.215648697	0.281246061
HOXA11	-0.069845408	0.662339862
HOXA13	-0.160980845	0.324365155
HOXA2	1.022103684	0.085992211
HOXA3	-0.111657896	0.609448404
HOXA4	0.078555709	0.695139082
HOXA5	0.512965597	0.261973662
HOXA6	0.371492099	0.125786347
HOXA9	0.069387106	0.636740808
HOXB1	-0.238955087	0.048776856
HOXB13	0.01169352	0.936059563
HOXB2	0.062565819	0.895640673
HOXB3	0.379712459	0.271110825
HOXB4	0.075529289	0.830717269
HOXB5	0.108915765	0.547632535
HOXB6	0.097610048	0.642922453
HOXB7	-0.108817136	0.469757823
HOXB8	-0.237714324	0.164313763
HOXB9	-0.268009243	0.053213615
HOXC10	0.191056958	0.25309049
HOXC11	-0.05270489	0.754207571
HOXC12	-0.050731559	0.787690301
HOXC13	-0.098663909	0.304700588
HOXC8	0.008282481	0.976695706
HOXC9	0.121735965	0.678115572
HOXD1	-0.294700714	0.063414818
HOXD10	1.300719272	0.088477646
HOXD11	0.611639665	0.170172919
HOXD12	-0.124196872	0.50452383
HOXD13	0.479194175	0.210681389
HOXD3	0.698665596	0.064814953
HOXD4	0.584834408	0.055161851
HOXD8	-0.064347192	0.651419625
HOXD9	0.295749459	0.25665853
HP	0.787018727	0.319103744
HP1BP3	0.705252843	0.005624542
HPCA	-0.831474049	0.005557192

HPCAL1	-0.261844801	0.264142793
HPCAL4	-1.622377181	0.003132413
HPDL	-0.068152377	0.593861868
HPF1	0.121494128	0.613052178
HPGD	-0.119046311	0.159223076
HPGDS	0.836615111	0.046792528
HPN	-0.27885524	0.123229751
HPRT1	-1.037506716	0.011647351
HPS1	-0.219091776	0.328241944
HPS3	1.795838571	0.017725216
HPS4	0.310376106	0.081390523
HPS5	0.366786051	0.100094889
HPS6	-0.162759334	0.173820085
HPX	0.062544989	0.62734035
HR	-0.401443977	0.194263418
HRAS	-0.203884828	0.22962459
HRC	0.092233716	0.616826714
HRG	-0.168484022	0.088286985
HRH3	-1.320087201	0.00530771
HRH4	-0.039065971	0.644122748
HRK	-0.060432516	0.783568677
HS1BP3	0.120632311	0.347223044
HS2ST1	0.465530128	0.077910368
HS3ST1	-0.058734773	0.846369748
HS3ST2	-0.452588144	0.164919812
HS3ST3A1	-0.048030281	0.930110116
HS3ST3B1	1.006717939	0.114270905
HS3ST4	-0.57978652	0.028571915
HS3ST5	-0.950437373	0.007640845
HS3ST6	-0.011956637	0.946174023
HS6ST1	0.086418618	0.789069634
HS6ST3	-1.939888821	0.0240514
HSBP1	0.301467287	0.125782871
HSCB	0.559140914	0.00235031
HSD11B1	-0.500258717	0.088105804
HSD11B2	0.198458952	0.321620139
HSD17B1	-0.121706553	0.657224321
HSD17B10	0.250160896	0.288549272
HSD17B11	0.457303227	0.158572001
HSD17B12	-0.051217402	0.721523148
HSD17B13	0.078106067	0.607426166
HSD17B14	0.073897232	0.845786531
HSD17B2	-0.115672471	0.348378671
HSD17B3	0.138517088	0.723856561
HSD17B6	-0.10297732	0.664585067
HSD17B7	0.418697738	0.087777021
HSD17B8	0.532717422	0.118038214
HSD3B1	-0.121647132	0.519213736

HSDL1	0.114301871	0.745502004
HSDL2	-0.326794245	0.1654732
HSF1	0.098802583	0.573790588
HSF2	-0.015645552	0.953362739
HSF2BP	0.395305332	0.07215175
HSF4	0.221830559	0.447828731
HSFX3	0.070030886	0.597300987
HSH2D	0.258633849	0.242326524
HSP90AB1	-0.245061698	0.307528419
HSP90B1	0.57011717	0.068765997
HSPA12A	-1.094204868	0.002398463
HSPA13	0.112979314	0.706057776
HSPA14	0.232830271	0.362105633
HSPA2	-1.503890701	0.045937888
HSPA4	-0.300520085	0.123511899
HSPA4L	-0.248856544	0.574475876
HSPA5	0.834986606	0.022445836
HSPA6	0.10868497	0.719732739
HSPA8	0.034745695	0.832304273
HSPA9	0.073195589	0.674472706
HSPB1	0.116756059	0.794766233
HSPB11	0.568317037	0.015599671
HSPB3	-0.413928252	0.011147349
HSPB6	-0.338512333	0.631245596
HSPB7	-0.265670238	0.335921879
HSPB9	-0.064671165	0.552725651
HSPBAP1	0.661450492	0.000685624
HSPBP1	-0.198055826	0.259053051
HSPD1	0.215175167	0.207134781
HSPH1	-0.512081035	0.142403894
HTATSF1	0.065668706	0.798544503
HTN1	-0.172981374	0.222845679
HTR1A	-0.664374583	0.002506853
HTR1B	-0.994082581	9.92E-05
HTR1D	-0.120494408	0.732977979
HTR1E	-0.930551798	0.008695727
HTR1F	-0.034451838	0.895198958
HTR2A	-2.184602789	0.023372257
HTR2B	-0.030091318	0.791653723
HTR3B	-0.843307041	0.003677817
HTR3C	-0.056971084	0.609626099
HTR4	-1.063828307	0.006177206
HTR5A	-0.849434549	0.002462149
HTR6	-0.3270463	0.060370505
HTR7	-0.181249142	0.086231823
HTRA1	0.092788644	0.846092035
HTRA2	0.02057086	0.924928069
HTRA3	0.159285535	0.267252885

HTRA4	0.183468331	0.091907448
HTT	-0.076397446	0.668746633
HUNK	0.213264571	0.654500215
HUS1	0.989035184	0.014503761
HUS1B	0.092015209	0.451027975
HUWE1	-0.152379838	0.221643074
HVCN1	0.144629957	0.428998236
HYAL2	-0.268328375	0.052773912
HYAL4	0.020051396	0.868550554
HYLS1	-0.152837709	0.437430306
HYOU1	-0.079409644	0.725073947
IAPP	-0.096807499	0.32264268
IARS2	-0.003584764	0.991232488
IBSP	2.616155909	0.011916889
ICA1	-0.586976864	0.110954577
ICAM1	0.718601147	0.301306841
ICAM3	0.450713459	0.047346166
ICAM5	-1.184568454	0.000627774
ICE2	0.389028955	0.156404863
ICMT	0.145488797	0.771020533
ICOS	-0.007568268	0.951794618
ICOSLG	-0.071671733	0.871201192
ID1	0.789129944	0.162373296
ID2	0.608615768	0.019807497
ID3	1.437327371	0.019441839
ID4	1.503493705	0.001899052
IDE	0.179346939	0.455359683
IDH1	0.941362676	0.000962015
IDH2	-0.50077608	0.029241897
IDH3A	-0.601686141	0.013196253
IDH3B	0.018462262	0.912053357
IDH3G	-0.216976526	0.131493027
IDI1	-0.796290953	0.000962664
IDI2	-0.173691123	0.274280631
IDO1	0.085529602	0.422962991
IDO2	-0.012385092	0.915486061
IDS	-1.438568648	0.001618895
IDUA	0.003744166	0.991447462
IER2	0.786336477	0.033687405
IER3	-0.070199855	0.928138525
IER5	0.438931703	0.220033149
IFFO2	-0.127165652	0.759096578
IFI16	1.447847957	0.003450027
IFI27	0.141717239	0.270464825
IFI27L2	-0.003631014	0.989859482
IFI30	2.34825966	0.00051205
IFI35	0.762503518	0.000856447
IFI44	2.50333481	3.35E-05

IFI44L	1.970988043	0.002074766
IFI6	0.688417799	0.117781718
IFIH1	0.876399028	0.004631703
IFIT2	0.514860206	0.043883036
IFIT5	0.055853452	0.847725163
IFITM1	-0.236627153	0.66061491
IFITM3	0.239381992	0.515468211
IFNA1	-0.132606434	0.174004226
IFNA10	-0.080763785	0.495812106
IFNA13	-0.208202874	0.190466912
IFNA14	-0.092875006	0.528960979
IFNA16	0.098727219	0.431793469
IFNA17	0.014184369	0.899807262
IFNA2	-0.27729888	0.034381814
IFNA21	-0.20680501	0.066745252
IFNA4	-0.171084416	0.168755601
IFNA6	-0.340826856	0.045937888
IFNA7	-0.019181998	0.878160468
IFNA8	-0.11378019	0.412449482
IFNAR1	0.234211048	0.297887786
IFNAR2	0.867845647	4.99E-05
IFNB1	-0.218851046	0.098505926
IFNE	-0.58486676	0.101058694
IFNG	-0.232933171	0.025742204
IFNGR1	0.553206295	0.057833697
IFNGR2	0.516066572	0.070736561
IFNK	-0.103572399	0.305540207
IFNL1	-0.026465473	0.84046247
IFNL2	-0.348968372	0.159869042
IFNL3	-0.051499961	0.774213189
IFNLR1	0.191369137	0.234531755
IFNW1	-0.085285535	0.34166678
IFRD1	0.708725316	0.002824951
IFT122	0.454156465	0.049321227
IFT140	0.796990431	0.000690887
IFT172	0.498177045	0.164919812
IFT22	0.15335002	0.56984988
IFT43	0.368177702	0.091870105
IFT46	0.281499721	0.155010611
IFT52	0.406235432	0.080062274
IFT57	0.154846423	0.441471891
IFT74	-0.136546145	0.668563803
IFT81	1.108447969	0.00073842
IFT88	0.329162985	0.247750029
IGBP1	-0.029584496	0.905042772
IGDCC3	0.144698274	0.539918168
IGDCC4	0.963624269	0.03217744
IGF1R	-0.408448389	0.06587285

IGF2BP1	-0.767094345	0.205207938
IGF2BP2	1.008162971	0.288316044
IGF2BP3	0.593977559	0.316598965
IGF2R	0.229889218	0.593292976
IGFBP1	0.059818855	0.746042719
IGFBP2	1.382177923	0.075609453
IGFBP4	0.603130024	0.416336033
IGFBP5	2.103090102	0.00447907
IGFBP6	-0.13261923	0.628834081
IGFBP7	-0.223269409	0.694628679
IGFL1	-0.005395563	0.977685858
IGFL2	0.024263832	0.897331052
IGFLR1	0.481690734	0.023487605
IGFN1	-0.344379844	0.127404013
IGHMBP2	0.146928802	0.425384977
IGLL1	-0.089941687	0.688563241
IGSF1	-0.052889333	0.725113118
IGSF10	0.049209796	0.7879778
IGSF11	0.615177041	0.173820085
IGSF21	-0.848015092	0.198206657
IGSF22	-0.269157345	0.080148455
IGSF3	0.758055449	0.109707043
IGSF6	0.863198029	0.013735004
IGSF8	-1.042699001	0.001027007
IGSF9	0.40849576	0.205095162
IGSF9B	0.512120335	0.445996476
IHH	-0.045282954	0.822749558
IK	0.192850832	0.18647246
IKBIP	0.644597709	0.202135975
IKBKE	0.245396701	0.437904076
IKZF1	0.273710325	0.246846601
IKZF4	0.336156737	0.07653211
IKZF5	-0.641567274	0.042468209
IL10	0.202263964	0.355183166
IL10RB	1.211891182	0.000123454
IL11	-0.683760718	0.044369981
IL12A	0.03090024	0.800623929
IL12B	-0.018027944	0.885440762
IL12RB1	-0.097961616	0.396258542
IL13	0.14850135	0.107507557
IL13RA1	0.316496114	0.518543609
IL13RA2	1.03316866	0.056100837
IL15	-0.166728754	0.136468749
IL17A	-0.150982449	0.123287453
IL17B	-0.035120118	0.795510023
IL17C	-0.095885899	0.460151242
IL17D	-0.096086941	0.729007438
IL17F	-0.074759143	0.46925675

IL17RA	0.351602697	0.34166678
IL17RB	0.243679769	0.734044406
IL17RC	0.435100654	0.05379235
IL17RD	0.413770789	0.352268887
IL17RE	0.152380233	0.404357997
IL17REL	0.113353711	0.547333024
IL18	1.172192993	0.004500154
IL18BP	1.145386031	0.000246503
IL18R1	0.012575797	0.948191138
IL18RAP	-0.05637516	0.574851886
IL19	-0.113414008	0.324819162
IL1A	-0.444516965	0.08782594
IL1B	-0.147944694	0.7644347
IL1F10	-0.082743634	0.485880843
IL1R1	0.29996829	0.632855398
IL1RAP	1.009252767	0.034422879
IL1RAPL1	-1.173061199	0.034242576
IL1RAPL2	-0.2813689	0.093135402
IL1RL1	0.015755699	0.913622296
IL1RL2	-0.116819711	0.297642305
IL1RN	-0.013318171	0.939257006
IL2	0.046371231	0.569159752
IL20	-0.071030134	0.507078763
IL20RA	0.113048301	0.669970192
IL21R	0.178413186	0.309990191
IL22	-0.223807268	0.061798257
IL22RA1	0.178572184	0.177541524
IL22RA2	-0.09541093	0.383350246
IL23A	-0.097747213	0.456272891
IL23R	-0.041528282	0.626293871
IL24	0.006069019	0.96051513
IL25	0.020421965	0.878130183
IL26	-0.268975043	0.235490774
IL27	-0.123230969	0.346427729
IL27RA	0.124871384	0.587548898
IL2RA	0.352084842	0.231625077
IL2RB	-0.001966489	0.993130668
IL2RG	0.565175372	0.008552888
IL3	0.032335024	0.753725884
IL32	0.178539794	0.491150917
IL33	0.363107012	0.138747607
IL34	-0.629263925	0.109707043
IL36A	0.071837187	0.45779214
IL36B	-0.030755384	0.736206294
IL36G	-0.023090277	0.890489751
IL36RN	-0.076937209	0.485672942
IL37	0.007834307	0.944819609
IL3RA	0.070360837	0.677252505

IL4	-0.139210067	0.161329673
IL4R	0.335214823	0.392266258
IL5	-0.026251085	0.796879518
IL5RA	0.00526161	0.982119627
IL6	-0.083435487	0.892641383
IL6R	0.412444126	0.08785171
IL6ST	-0.264094128	0.241875864
IL7	0.147479417	0.311332683
IL7R	0.26542723	0.172134152
IL9	-0.04045624	0.630476968
IL9R	-0.005928303	0.964075003
ILDR2	0.725084079	0.173820085
ILF2	0.305554951	0.286021893
ILF3	0.82201956	0.001249683
ILKAP	0.362004998	0.058992523
ILRUN	-0.662429866	0.000783213
ILVBL	0.179182832	0.313101113
IMMP1L	0.301990728	0.088639485
IMMP2L	0.565816925	0.050873245
IMMT	0.100300259	0.631524871
IMP3	-0.01625813	0.947707242
IMP4	0.024579338	0.872052146
IMPA2	0.025361338	0.941866997
IMPACT	0.551388825	0.144036533
IMPDH1	0.234159232	0.445996476
IMPG1	-0.062720047	0.419236188
IMPG2	0.157938952	0.144002414
INA	-3.045329091	0.006329299
INAFM1	0.660939388	0.122755046
INAFM2	0.49710772	0.244940659
INAVA	0.192399925	0.265590842
INF2	-0.454771616	0.132455364
ING2	0.128544686	0.404656768
ING3	0.665962516	0.016325061
ING4	0.190027673	0.23805142
INHA	-0.098096662	0.388614766
INHBA	-0.781019811	0.226735959
INHBB	0.898850632	0.001454069
INHBC	0.155908193	0.180150463
INHBE	-0.01805338	0.890957888
INIP	-0.20716703	0.245231031
INKA1	-0.039441276	0.81156652
INKA2	-0.200070754	0.536369113
INO80	-0.049321667	0.762174089
INO80D	0.563939081	0.003876591
INO80E	0.231257201	0.457728097
INPP4B	0.102244074	0.745502004
INPP5A	-1.124131232	0.001910109

INPP5B	0.293655252	0.049926726
INPP5E	0.352682219	0.062347184
INPP5F	-1.095040135	0.038710038
INPP5J	-0.42909362	0.005652763
INPPL1	0.989137113	0.006496089
INSIG1	-0.132976452	0.604513223
INSIG2	0.605764918	0.107462271
INSL4	-0.09126884	0.382977678
INSL5	0.041628284	0.727094424
INSL6	-0.115975283	0.229593976
INSM1	0.31956645	0.473162998
INSM2	-0.162321783	0.056771705
INSR	0.336488122	0.24686843
INSRR	0.073267276	0.826479462
INTS10	0.55754636	0.000962015
INTS12	0.399981065	0.086120987
INTS13	0.423141269	0.208052611
INTS3	0.549670209	0.009889747
INTS4	0.677860822	0.003378761
INTS6	0.21630295	0.247896619
INTS6L	0.06608706	0.751272631
INTS7	0.693923179	0.038812628
INTS8	0.65565922	0.005712247
INTU	0.197569801	0.460676772
IP6K1	-0.22728908	0.229823961
IPMK	0.219276097	0.498546305
IPO11	0.210915429	0.151685169
IPO13	-0.701637641	0.008623353
IPO5	-0.11254055	0.553475251
IPO7	-0.084605002	0.727460808
IPO8	0.520567899	0.013504955
IPO9	0.79614714	0.002062218
IPP	0.451426435	0.021385313
IQCA1	-1.104556621	0.008448974
IQCD	0.083371652	0.593677708
IQCE	0.333623215	0.173065741
IQCF1	0.120716889	0.222935337
IQCF2	-0.032890928	0.745309804
IQCG	0.960443449	0.002995508
IQCK	0.212572834	0.390712327
IQCM	-0.018795757	0.847725163
IQGAP1	0.318507602	0.482798306
IQGAP2	1.601094084	0.000204607
IQGAP3	0.708850346	0.079175322
IQSEC3	-0.614565142	0.035137901
IQUB	0.651288604	0.158572001
IRAK1	0.867049276	0.006888096
IRAK2	0.050223072	0.904256812

IRAK3	0.555706362	0.243067107
IREB2	0.246297192	0.307039391
IRF1	0.978628203	0.014954496
IRF2	0.785252738	4.99E-05
IRF2BP1	-0.096870576	0.661668618
IRF2BPL	0.296545888	0.133828328
IRF5	0.237776073	0.136663136
IRF6	-0.023793629	0.886463258
IRF7	0.324889855	0.057125769
IRF8	1.174830067	0.013043918
IRGC	0.12113953	0.45779214
IRS1	-0.455443636	0.161642263
IRS2	0.207123802	0.689785691
IRS4	-0.142600296	0.152774879
IRX1	0.328592427	0.341131985
IRX2	0.037240885	0.882269228
IRX3	-0.082535474	0.792979636
IRX4	-0.159112044	0.318130405
IRX5	0.085990485	0.693365203
IRX6	0.062023939	0.566497648
ISCA2	0.003531209	0.986404569
ISCU	-0.319767747	0.078529646
ISG15	0.566084585	0.116764075
ISG20	0.252388838	0.124611161
ISG20L2	0.308917908	0.044164454
ISL1	-0.037496542	0.767345752
ISL2	-0.085412424	0.56239321
ISLR	0.143327666	0.47547126
ISM2	0.048019752	0.773412832
ISOC1	-0.023743181	0.935244061
IST1	0.438686977	0.004082156
ISX	0.005796389	0.963336619
ISYNA1	-0.143681585	0.732026934
ITFG1	-0.341722876	0.097969584
ITFG2	0.359580353	0.139307762
ITGA1	0.812024014	0.027431564
ITGA10	0.198156021	0.36814093
ITGA11	-0.293064087	0.752135041
ITGA2	0.388070147	0.595349657
ITGA3	0.205778707	0.818389128
ITGA4	0.342466857	0.494709345
ITGA5	0.582239622	0.371706173
ITGA6	0.411903901	0.179104238
ITGA8	-0.368195823	0.134234469
ITGA9	-0.361312432	0.394267961
ITGAE	0.222526471	0.080901275
ITGAL	1.133184043	0.002552577
ITGAV	0.596611403	0.057048491

ITGAX	1.379825318	0.011384613
ITGB1	0.140667664	0.780799053
ITGB1BP1	0.012995717	0.957567761
ITGB1BP2	0.07708866	0.481859178
ITGB2	0.667735613	0.002977765
ITGB3	0.6715049	0.325895121
ITGB5	0.554798406	0.228956433
ITGB6	-0.054310503	0.500185066
ITGB7	-0.113462656	0.377625058
ITGB8	1.390395408	0.016287042
ITGBL1	0.290229054	0.640741619
ITIH2	0.080272405	0.757155169
ITIH3	-0.106735121	0.321226681
ITIH4	0.48104516	0.146945011
ITIH6	0.095922023	0.291220441
ITK	-0.016030975	0.924797924
ITLN1	-0.010289257	0.935244061
ITLN2	-0.087283691	0.459874564
ITM2A	-0.164419755	0.764470728
ITM2B	-0.046438274	0.854617428
ITM2C	-0.169668283	0.597300987
ITPA	0.794859734	0.003133494
ITPK1	-0.851840304	0.024864763
ITPKA	-0.966008037	0.006880123
ITPKB	0.607013091	0.293719062
ITPR2	0.978804817	0.01318187
ITPR3	0.749670044	0.054969202
ITPRIP	-0.006045584	0.98586845
ITSN1	-0.679385413	0.014250032
ITSN2	0.150455665	0.492496846
IVD	-0.017218304	0.95239805
IVL	-0.111374567	0.344075125
IVNS1ABP	0.878189823	0.001340553
IWS1	0.290913985	0.038520441
IZUMO1	-0.173471832	0.440090257
IZUMO1R	-0.011124586	0.93379029
IZUMO2	-0.249281356	0.045382318
IZUMO3	0.031693298	0.775048487
IZUMO4	-0.110726119	0.653101302
JADE1	0.227595068	0.443082319
JADE2	-0.379664147	0.086901978
JAG1	1.56100182	0.008098465
JAG2	-0.358236691	0.080384052
JAGN1	-0.03105892	0.904012019
JAK1	0.268647057	0.069194222
JAK2	0.134842091	0.663942355
JAK3	0.224891716	0.374930374
JAKMIP2	0.589050218	0.281678876

JAKMIP3	-1.237748306	0.00566737
JAM2	0.813422589	0.016041174
JAM3	0.204133705	0.549312728
JARID2	-0.000458352	0.998322071
JAZF1	0.258659156	0.198244126
JCHAIN	-0.022620273	0.892887186
JDP2	-0.428023081	0.028452842
JHY	0.06799773	0.821856037
JKAMP	0.038795877	0.876312032
JMJD4	0.302013548	0.048048058
JMJD6	0.022299207	0.919320077
JMJD7-PLA2G4B	0.159020459	0.49153673
JMY	-0.238349823	0.340381872
JOSD1	-0.257922168	0.200198733
JOSD2	0.075528994	0.730298676
JPH1	-0.431322326	0.447783257
JPH2	-0.095027425	0.720550051
JPH3	-2.224253668	0.001597225
JPT1	0.29713669	0.270624699
JPT2	0.43660469	0.037423847
JRK	0.088274723	0.506593831
JRKL	0.206302355	0.240239771
JSRP1	-0.135735652	0.397133712
JUN	1.073893849	0.004504846
JUNB	0.874233122	0.138658133
JUP	-0.813442524	0.028141354
KAAG1	-0.137589208	0.545344317
KANK4	-0.115781951	0.569712378
KANSL1	0.214158324	0.351991898
KANSL2	0.480703335	0.001795266
KAT2B	0.368349387	0.449235883
KAT5	0.216604165	0.196209899
KAT6B	-0.183118377	0.530361631
KAT7	0.507143502	0.013749517
KATNA1	0.456825928	0.020015342
KATNAL1	-0.155697372	0.500373093
KATNAL2	0.531442625	0.077798541
KATNB1	-0.440443566	0.011632897
KATNBL1	0.10368655	0.622964251
KAZALD1	-0.155769779	0.180912739
KAZN	0.308022089	0.315762096
KBTBD11-OT1	-0.115282539	0.396566491
KBTBD12	-0.034521133	0.877443564
KBTBD2	0.641958773	0.015894451
KBTBD3	0.192501686	0.458286417
KBTBD4	0.000870129	0.995953997
KBTBD6	-0.086233511	0.78413419
KBTBD7	0.02003035	0.947457916

KBTBD8	-0.171851906	0.150096828
KCMF1	-0.028129278	0.896420697
KCNA1	-1.540957564	0.004933097
KCNA10	-0.020850829	0.878285161
KCNA2	-1.083670544	0.149340701
KCNA4	-0.735774171	0.152406037
KCNA5	-0.2138443	0.241986251
KCNA7	-0.086827766	0.375950158
KCNAB1	-1.249812228	0.002419881
KCNAB2	-1.083572365	0.006336447
KCNAB3	0.337269747	0.376402549
KCNB1	-1.416500987	0.013851792
KCNB2	-0.899629414	0.007673642
KCNC3	-0.302175576	0.329369557
KCNC4	-0.379976382	0.08477333
KCND1	0.192590557	0.522242033
KCND2	0.001840339	0.998667636
KCND3	0.104605095	0.900673665
KCNE1	0.15068764	0.549902962
KCNE2	0.115175418	0.478963272
KCNE3	0.27070375	0.107375492
KCNE4	0.658815728	0.100754919
KCNE5	0.013971971	0.934380733
KCNF1	-0.175930401	0.613374066
KCNG1	-0.290589446	0.041553053
KCNG2	-0.104164297	0.647712311
KCNG3	-0.234356907	0.295546502
KCNG4	0.028602012	0.83276352
KCNH1	-1.867593434	0.011492225
KCNH2	0.296765118	0.345154663
KCNH3	-1.984699457	0.00062852
KCNH4	-0.657376022	0.014320852
KCNH5	-1.069813741	0.02383959
KCNH6	-0.100323893	0.402775257
KCNH7	-1.294618401	0.108546904
KCNH8	-0.931217421	0.176560573
KCNIP2	-1.109903607	0.01449345
KCNIP3	-0.812545375	0.228531369
KCNIP4	-1.70671114	0.012824601
KCNJ1	-0.12103783	0.161329673
KCNJ10	-0.203065245	0.870363001
KCNJ11	-0.234064111	0.120473196
KCNJ12	-0.25379959	0.013337633
KCNJ13	-0.095494814	0.548646117
KCNJ15	-0.191682047	0.122755046
KCNJ16	0.505929973	0.556759506
KCNJ2	-0.320940633	0.346852567
KCNJ3	-2.118934416	0.00295173

KCNJ4	-1.054675094	0.00860036
KCNJ5	0.111435388	0.414217978
KCNJ6	-1.024361451	0.046333619
KCNJ8	0.311443165	0.201909681
KCNJ9	-1.511668516	0.009225069
KCNK1	-0.777745869	0.093807437
KCNK10	-0.25549639	0.25896041
KCNK12	-0.524920364	0.022434883
KCNK13	-0.205479554	0.347223044
KCNK15	-0.08657789	0.584436904
KCNK18	-0.002602115	0.989421806
KCNK3	-0.090253463	0.652500938
KCNK4	-0.287919261	0.090602006
KCNK5	0.192111557	0.680038384
KCNK6	0.027435861	0.943265544
KCNK7	-0.122437356	0.397432695
KCNK9	-0.368136344	0.221676849
KCNMB1	0.833977569	0.002378329
KCNMB2	-0.043368722	0.885622833
KCNMB3	0.738852822	0.002062218
KCNMB4	-0.731877863	0.063240636
KCNN1	-1.408434434	0.003512268
KCNN2	-0.077461031	0.829199138
KCNN3	0.234031988	0.739670021
KCNQ1	0.295525717	0.096676129
KCNQ2	-0.605405894	0.291869973
KCNQ3	-1.406474086	0.020889014
KCNRG	0.129957491	0.662533067
KCNS1	-0.840369389	0.003880684
KCNS2	-1.273476116	0.00859216
KCNS3	0.0249444	0.912859018
KCNT2	0.115644925	0.863600504
KCNV1	-0.931411441	0.015093776
KCNV2	-0.069241496	0.618720598
KCTD1	-0.881143906	0.007346219
KCTD10	0.009735168	0.969317249
KCTD11	0.405939897	0.107462271
KCTD13	0.039259148	0.922591378
KCTD15	0.199804207	0.440194588
KCTD17	-0.268965971	0.277330696
KCTD18	0.161303012	0.4771806
KCTD20	0.369360485	0.316299461
KCTD21	0.018401838	0.935238087
KCTD3	0.58181214	0.018969349
KCTD4	-0.359972342	0.135123268
KCTD5	0.703276416	0.006729601
KCTD6	0.247641236	0.329369557
KCTD8	-0.307324664	0.558261496

KCTD9	0.066731287	0.847248255
KDELR1	0.32598786	0.45676302
KDELR2	0.322315535	0.254297669
KDELR3	-0.220609226	0.657371825
KDF1	-0.117160966	0.281752955
KDM2B	0.187081632	0.103450925
KDM3A	0.681678757	0.001192251
KDM3B	0.146964071	0.439914357
KDM4A	0.318373828	0.096280585
KDM4C	-0.093033844	0.612895871
KDM4D	0.095095411	0.518566194
KDM5B	-0.064109825	0.860992973
KDM5C	0.388383441	0.054474658
KDM6A	0.166785739	0.475052564
KDM6B	-0.438496218	0.055487058
KDR	0.691076915	0.172596132
KDSR	-0.015136036	0.963152726
KEAP1	0.247472855	0.232643514
KEL	-0.347370315	0.248798732
KERA	-0.114070991	0.261495614
KHDRBS1	0.406604117	0.018595078
KHDRBS2	-2.543100383	0.003306163
KHDRBS3	0.043361676	0.941168506
KHK	-0.216868894	0.209848876
KHSRP	0.233697947	0.124389428
KIAA0319L	0.093940623	0.537033283
KIAA1109	-0.210816298	0.429982193
KIAA1143	0.17183234	0.36357116
KIAA1191	-0.157544389	0.491946753
KIAA1217	-0.3329938	0.558118043
KIAA1328	0.091119812	0.628748167
KIAA1549L	-1.784443617	0.001577058
KIAA1614	0.234872801	0.295354807
KIAA2013	0.20334276	0.356467623
KIF11	0.727909947	0.165340557
KIF12	-0.410535604	0.003988714
KIF13A	-0.091501614	0.731357875
KIF13B	0.073368711	0.876312032
KIF15	1.063754749	0.039772717
KIF16B	0.069769425	0.835499613
KIF17	-0.339410218	0.056705846
KIF18A	0.654221829	0.156456644
KIF19	-0.249836275	0.219384406
KIF1C	-0.731312977	0.014320852
KIF20A	0.536495147	0.347139339
KIF20B	0.362944321	0.322614895
KIF24	0.585471038	0.071190524
KIF25	-0.121205167	0.201701899

KIF27	0.703840052	0.013916639
KIF2B	-0.069459608	0.665904933
KIF2C	0.788329148	0.086231823
KIF3A	-0.645378177	0.114014918
KIF3B	-0.140141929	0.532562355
KIF3C	-1.220005686	0.004415334
KIF4A	1.2693073	0.002895035
KIF5A	-3.130104166	0.006091959
KIF5B	-0.897625825	0.001368876
KIF6	-0.365454909	0.244617058
KIF7	0.27553524	0.143525519
KIFC2	-0.689310863	0.014668297
KIFC3	0.165825311	0.579730314
KIN	0.261889684	0.219116418
KIR2DL1	-0.190890168	0.183638914
KIR2DL4	0.086369306	0.475430754
KIR2DS4	-0.074367378	0.689387141
KIR3DL2	-0.201157684	0.329369557
KIRREL1	0.788961906	0.252127612
KIRREL2	-0.015117593	0.901235662
KIRREL3	-1.40935605	0.000165953
KISS1	-0.520549087	0.158572001
KISS1R	-0.243583495	0.161329673
KIT	-0.278783368	0.742197286
KITLG	-0.306630229	0.522852867
KIZ	0.646201588	0.054144496
KL	-0.070844658	0.723803704
KLB	-0.138639489	0.096193714
KLC1	-0.280454905	0.242750377
KLC3	-0.170619584	0.166149474
KLC4	-0.104002643	0.615470416
KLF1	-0.080514647	0.515173789
KLF10	0.549876084	0.038777871
KLF13	-0.238305249	0.203783861
KLF14	-0.111648338	0.373011317
KLF15	0.434831409	0.071387892
KLF16	-0.276127157	0.187177307
KLF17	-0.094174222	0.374930374
KLF2	0.162896502	0.774697951
KLF3	0.452895312	0.039819061
KLF4	0.085328492	0.751272631
KLF5	-0.120426283	0.519310408
KLF6	0.747514516	0.123801057
KLF7	0.062600656	0.802283726
KLF8	-0.191304644	0.497981199
KLF9	0.246613067	0.418912486
KLHDC1	-0.250747377	0.601426968
KLHDC10	0.182140023	0.325077423

KLHDC2	-0.110415579	0.596519233
KLHDC4	0.270536229	0.134776399
KLHDC7A	-0.223858596	0.024792405
KLHDC7B	-0.114836649	0.529207785
KLHDC8A	2.745193891	0.001933896
KLHDC8B	0.213264918	0.161834484
KLHDC9	-0.001939012	0.994477384
KLHL10	0.061977284	0.621978154
KLHL11	-0.141740692	0.430305006
KLHL13	0.503426184	0.010444426
KLHL17	-0.100596988	0.595623098
KLHL18	-0.083670364	0.625457691
KLHL2	-0.576076168	0.114071721
KLHL20	0.345894426	0.15015411
KLHL24	-0.559293164	0.075609453
KLHL25	0.638279956	0.001528753
KLHL26	-0.593288944	0.032911836
KLHL28	0.146382709	0.491010445
KLHL3	-0.477821986	0.05852348
KLHL30	-0.081400001	0.560024501
KLHL32	-1.568611993	0.013430848
KLHL34	-0.004008056	0.98286235
KLHL36	-0.094333523	0.681406422
KLHL38	-0.385041425	0.027565915
KLHL4	0.27086157	0.704117016
KLHL40	0.025451466	0.830365605
KLHL41	0.044717773	0.742956394
KLHL42	0.407064827	0.056771705
KLHL5	0.310765589	0.18647246
KLHL6	0.589141151	0.063600421
KLHL8	0.056748791	0.82445066
KLK1	-0.085192759	0.504166617
KLK10	0.007100893	0.959954076
KLK11	-0.11562859	0.522991757
KLK12	0.015668174	0.906128982
KLK13	-0.148814806	0.2583263
KLK15	-0.380914653	0.019482261
KLK2	0.039086736	0.703745425
KLK4	-0.094127724	0.380462395
KLK5	-0.004918016	0.981296494
KLK6	-1.682970442	0.016032017
KLK7	-0.727790965	0.056125624
KLK8	-0.050857138	0.700541866
KLKB1	0.520366272	0.102956472
KLRB1	0.083033737	0.487427325
KLRC1	0.711934542	0.232643514
KLRC3	0.958324277	0.312339407
KLRD1	0.103089975	0.5208979

KLRG1	0.199647386	0.308958227
KLRG2	0.012151489	0.94953487
KMO	0.247265855	0.271679271
KMT2D	0.1540623	0.315669042
KMT2E	0.326934942	0.162591674
KMT5A	0.300276081	0.034478937
KMT5B	0.381948193	0.049245411
KMT5C	0.16693347	0.417523719
KNDC1	-0.954601072	0.016836281
KNL1	1.055237732	0.038372241
KNOP1	0.429753744	0.061048788
KNSTRN	0.157523472	0.591664385
KNTC1	1.685991725	0.000927703
KPNA3	-0.41664322	0.243791553
KPNA4	0.123168223	0.549686574
KPNA5	0.04455183	0.900965117
KPNA6	-0.181815576	0.321226681
KPNA7	0.006825832	0.958915787
KPNB1	0.381720454	0.025948334
KPTN	0.096744989	0.604501355
KRAS	-0.095142483	0.688306212
KRBA2	0.747464928	0.014092274
KRBOX1	0.192231765	0.498017986
KRBOX4	0.540344873	0.004188961
KRCC1	0.457947026	0.063337246
KREMEN1	-0.160120101	0.368464473
KREMEN2	0.093191203	0.496096401
KRI1	0.725061561	0.002398463
KRIT1	0.891717281	0.00152992
KRR1	0.393425919	0.03424149
KRT1	-0.044965385	0.707973091
KRT10	-0.021955809	0.87676402
KRT12	-0.130076096	0.22124855
KRT13	-0.044604413	0.697649656
KRT14	-0.091057674	0.534272534
KRT15	-0.066610185	0.572166691
KRT19	-0.570128056	0.181734674
KRT2	0.085862298	0.448540855
KRT20	0.034532195	0.730363414
KRT222	-1.664667135	0.008380832
KRT23	-0.099623116	0.438999174
KRT24	-0.165984912	0.104811761
KRT25	0.0758699	0.435321191
KRT27	-0.1764769	0.094391685
KRT28	-0.067692401	0.404656768
KRT3	-0.084950601	0.419711874
KRT31	-0.188161199	0.356467623
KRT32	0.122463612	0.463233053

KRT35	-0.11435172	0.395610608
KRT36	-0.067713674	0.658552671
KRT37	0.047212652	0.843477662
KRT38	-0.34487412	0.048776856
KRT39	-0.164123405	0.144248668
KRT4	-0.014792609	0.901052359
KRT40	0.045964325	0.609287902
KRT5	0.264992897	0.127006537
KRT7	-0.391406611	0.39064296
KRT71	-0.264950885	0.049245411
KRT72	-0.375960055	0.033854322
KRT73	-0.141982988	0.311332683
KRT74	-0.185065529	0.195793241
KRT75	-0.466498108	0.118947126
KRT77	-0.102290711	0.552725651
KRT80	-0.340163978	0.300767997
KRT81	-0.083025551	0.719619039
KRT82	-0.30429316	0.090325721
KRT83	-0.319280564	0.073077233
KRT84	0.063819536	0.601656239
KRT85	0.097304052	0.59692935
KRT9	-0.085544338	0.548219322
KRTAP10-1	-0.200258858	0.253589723
KRTAP10-10	0.476212951	0.033236429
KRTAP10-11	0.064843162	0.615329823
KRTAP10-12	0.166152117	0.354981237
KRTAP10-2	-0.136143546	0.37965508
KRTAP10-3	-0.024756722	0.919404887
KRTAP10-4	-0.088441111	0.522405582
KRTAP10-5	0.021133704	0.94254959
KRTAP10-6	-0.021924403	0.91422344
KRTAP10-8	-0.039930477	0.82820301
KRTAP10-9	0.017239201	0.946750418
KRTAP11-1	0.050659271	0.791192055
KRTAP12-1	-0.050392542	0.605801522
KRTAP12-2	0.040691666	0.825865081
KRTAP12-3	-0.018725029	0.910283756
KRTAP12-4	-0.134234805	0.416890596
KRTAP1-3	-0.077746079	0.523892352
KRTAP13-1	0.03892334	0.773333319
KRTAP13-2	0.142538807	0.453509269
KRTAP13-3	-0.254532106	0.053517482
KRTAP13-4	-0.220699611	0.026331477
KRTAP1-4	-0.134669953	0.300808949
KRTAP1-5	0.044754558	0.874708742
KRTAP15-1	-0.116813402	0.350573936
KRTAP17-1	-0.015472784	0.932999857
KRTAP19-1	-0.129674985	0.271319859

KRTAP19-2	-0.742068944	0.029686684
KRTAP19-3	0.03632333	0.740118086
KRTAP19-4	-0.01669187	0.894463827
KRTAP19-5	-0.242757197	0.077022752
KRTAP19-6	-0.120073132	0.22497975
KRTAP19-7	-0.229068572	0.064067744
KRTAP20-1	0.088748461	0.628748167
KRTAP20-2	0.014555111	0.908595685
KRTAP21-1	-0.049220707	0.746806892
KRTAP21-2	-0.197726586	0.106370667
KRTAP22-1	-0.118569153	0.126986296
KRTAP23-1	-0.051644603	0.714773068
KRTAP2-4	-0.16685726	0.194534446
KRTAP26-1	-0.048534384	0.595088385
KRTAP29-1	0.213359965	0.044310474
KRTAP3-1	-0.122831141	0.318009803
KRTAP3-2	0.011210814	0.966065344
KRTAP3-3	-0.134148436	0.177287889
KRTAP4-12	-0.278116751	0.020502048
KRTAP4-16	0.227562097	0.34178924
KRTAP4-2	-0.341180973	0.057751463
KRTAP4-4	-0.139277221	0.155883617
KRTAP4-5	-0.090760703	0.323347141
KRTAP4-8	-0.339799049	0.08893089
KRTAP4-9	-0.241751591	0.048435768
KRTAP5-2	-0.233280109	0.448606398
KRTAP5-8	0.074181476	0.7879778
KRTAP5-9	0.148652379	0.291211053
KRTAP6-1	-0.053579787	0.583914788
KRTAP6-2	0.092897576	0.651557072
KRTAP6-3	-0.075620946	0.743546189
KRTAP8-1	-0.11132911	0.313353465
KRTAP9-2	-0.035317785	0.830359479
KRTAP9-3	-0.138022044	0.300621064
KRTAP9-4	0.150294705	0.472294521
KRTAP9-6	-0.094869105	0.388315947
KRTAP9-7	-0.222201451	0.11395872
KRTCAP3	0.100343055	0.475725358
KRTDAP	-0.019546149	0.878384488
KSR2	-1.859639578	0.001560884
KTN1	0.01940275	0.941091514
KY	-0.673551571	0.00148582
KYAT1	-0.118924075	0.581009554
L1CAM	-1.524638448	0.019547982
L2HGDH	-0.021079726	0.927780014
L3HYPDH	0.323306702	0.23852602
L3MBTL1	0.142389009	0.294573719
L3MBTL2	-0.05347554	0.832105499

L3MBTL3	0.043159144	0.861347321
L3MBTL4	0.618240081	0.061006569
LACC1	-0.742197657	0.054716207
LACRT	-0.068510041	0.513684677
LACTB	0.218597553	0.533233651
LACTB2	0.130500816	0.631524871
LACTBL1	0.127160814	0.387859918
LAD1	-0.375962863	0.077418713
LAG3	0.088752111	0.616852572
LAGE3	-0.105911658	0.55712095
LAIR1	0.793543199	0.005717101
LAIR2	0.175533375	0.318895907
LALBA	-0.069141084	0.545645355
LAMA2	1.238545514	0.070374278
LAMA3	-0.226227964	0.254025679
LAMA5	0.433338824	0.324631071
LAMB1	1.025099141	0.295585953
LAMB2	1.014349837	0.018844974
LAMB4	0.655374626	0.024710164
LAMC2	-0.227738692	0.43381273
LAMC3	0.648576938	0.027465518
LAMP1	0.107581113	0.729474502
LAMP2	-0.38500737	0.174946769
LAMP3	0.013550572	0.915817139
LAMP5	-0.375278616	0.683448753
LAMTOR2	0.255992482	0.053637776
LAMTOR4	0.344107969	0.097591568
LAMTOR5	0.009787301	0.953362739
LANCL1	-1.066672663	0.002413407
LANCL2	0.568023005	0.388658118
LANCL3	0.017818424	0.961264056
LAP3	1.094451213	0.00010541
LAPTM4A	0.586251916	0.006776741
LAPTM4B	0.206200929	0.646187802
LAPTM5	1.819232201	0.037049711
LARGE1	-0.558971462	0.035688634
LARGE2	0.102118719	0.528869066
LARP1	-0.160463233	0.3009126
LARP1B	-0.372987758	0.054615236
LARP4	0.225555233	0.370192134
LARP6	-1.162823925	0.000814384
LARP7	0.692968953	0.005714159
LARS2	-0.019831454	0.937288264
LAS1L	0.204341297	0.324345973
LAT	0.395797041	0.169563691
LAT2	0.307392517	0.240239771
LATS1	0.115761908	0.520756654
LATS2	-0.039826801	0.938713447

LAX1	-0.188580642	0.050414179
LBH	-0.004362063	0.990996918
LBHD1	0.138916707	0.591000178
LBP	-0.057514748	0.603497372
LBR	0.921520542	0.003568303
LBX1	-0.188838938	0.110072691
LBX2	-0.025279048	0.880469311
LCA5	-0.046678437	0.84451194
LCA5L	0.228110371	0.276465372
LCAT	1.195744161	0.003614713
LCE1B	-0.121574822	0.203905906
LCE1C	0.049272586	0.767133752
LCE1E	-0.046827882	0.738998117
LCE1F	0.043811207	0.7240164
LCE2A	-0.161398216	0.30783194
LCE2B	0.045711952	0.780805655
LCE2C	-0.145366137	0.346281317
LCE2D	0.140154751	0.414394125
LCE3A	0.25449766	0.290650106
LCE3B	-0.32151192	0.138677265
LCE3C	0.194946134	0.179527726
LCE3D	-0.070896275	0.662127679
LCE3E	-0.147731915	0.257972212
LCE4A	0.476131954	0.014474793
LCE5A	-0.133220319	0.160515731
LCMT1	-0.334634711	0.047832669
LCMT2	0.061622957	0.737301832
LCN1	-0.101560296	0.578325381
LCN10	0.116795921	0.274986782
LCN12	-0.298582188	0.056457719
LCN15	-0.154522928	0.173390717
LCN2	-0.117263727	0.562498279
LCN9	-0.191859656	0.09181658
LCP1	0.628161266	0.073808099
LCP2	1.280970076	0.039393837
LCT	-0.174091637	0.073893969
LCTL	0.098061775	0.784610747
LDAH	0.516076628	0.057492253
LDB1	-0.587882372	0.012222626
LDHAL6A	-0.040255292	0.749326279
LDHAL6B	-0.235240511	0.033465893
LDHB	-0.474524237	0.047714879
LDHC	-0.146223046	0.220916047
LDHD	-0.422095107	0.111284652
LDLR	-0.159263234	0.726649288
LDLRAD3	1.433897206	0.00015288
LDLRAP1	-0.017971412	0.958982209
LDOC1	-0.942397694	0.001541164

LEAP2	0.295751298	0.053622329
LECT2	0.010758592	0.920298284
LEFTY1	0.041442481	0.845275235
LEFTY2	0.015068314	0.917640056
LEKR1	0.066071697	0.479580596
LEMD3	-0.111156147	0.738527319
LENEP	-0.03831307	0.792979636
LENG1	0.086121402	0.553475251
LENG8	0.526574796	0.238060849
LENG9	-0.070615698	0.684286098
LEO1	-0.160768531	0.452102972
LEP	-0.047544175	0.636823004
LEPR	-0.011349711	0.964075003
LEPROTL1	0.171440122	0.205685941
LETM1	0.046103573	0.774991389
LETMD1	0.212015052	0.29981591
LEXM	-0.058997801	0.663494227
LGALS13	-0.370444574	0.009294303
LGALS2	0.060553374	0.681406422
LGALS3BP	0.725374273	0.091233528
LGALS4	0.097085529	0.505656412
LGALS7	-0.298196184	0.036926773
LGALS8	-0.040206669	0.855304945
LGALS9	0.365468616	0.127380124
LGALS9B	0.673763975	0.005461476
LGALSL	-0.288524654	0.47768508
LGI1	-1.4614273	0.153482869
LGI2	0.225266176	0.388574866
LGI3	-2.383184675	0.001072162
LGI4	-0.859405899	0.13951092
LGMN	0.152034941	0.670750072
LGR4	-0.189121742	0.587390246
LGR5	-0.834990107	0.250526607
LGR6	0.453734615	0.455880519
LGSN	-0.008774434	0.929374994
LHB	0.036348056	0.875594575
LHCGR	0.042260254	0.821666052
LHFPL1	-0.053805999	0.757437178
LHFPL2	0.768830604	0.00183367
LHFPL3	0.877933155	0.157537563
LHFPL4	-0.106292992	0.780791174
LHFPL5	-0.236515324	0.09801245
LHFPL6	0.350568167	0.271147368
LHX1	-0.095781135	0.493638298
LHX2	-1.638596594	0.000985144
LHX3	-0.446577405	0.016780753
LHX4	-0.047295171	0.574314698
LHX5	0.049857629	0.794214198

LHX6	-0.354455212	0.024933462
LHX8	-0.026859921	0.864990421
LHX9	0.075134955	0.653307912
LIAS	0.176816083	0.341052238
LIF	-0.031837258	0.973430461
LIG1	0.618775395	0.060370505
LIG3	0.662020955	0.009448032
LIG4	-0.12331395	0.484924063
LILRA1	0.111089499	0.576779548
LILRA3	0.096662069	0.522414348
LILRA4	-0.219121845	0.2583263
LILRA5	-0.095077506	0.448441679
LILRB1	0.579222538	0.077798541
LILRB2	-0.093921524	0.707337139
LILRB4	0.285285094	0.28073347
LIMD1	0.87231095	0.011584437
LIMD2	-0.052655433	0.850311401
LIMK1	-0.111716028	0.711091662
LIMS2	-0.313856856	0.014096806
LIN28A	-0.185122359	0.062814672
LIN28B	-0.078201637	0.700746349
LIN52	0.113928223	0.552995453
LIN54	-0.084812418	0.576990313
LIN7A	0.031721373	0.942141247
LIN7B	-0.270125744	0.073103353
LIN7C	-0.087288481	0.752294052
LIN9	0.373433592	0.030210499
LINGO1	-0.448387748	0.163941079
LINGO2	-1.256257701	0.008431157
LINGO4	-0.053041814	0.67915756
LINS1	0.172525152	0.5208979
LIPC	-0.14638421	0.172049116
LIPH	-0.623934479	0.259652165
LIPI	-0.111934617	0.219344445
LIPK	-0.123317728	0.248675501
LITAF	0.303706026	0.378064286
LIX1	-0.057192114	0.939044278
LLCFC1	-0.155642908	0.188234188
LLGL1	-0.345330314	0.076969804
LLGL2	-0.00932937	0.96051513
LLPH	0.089099532	0.547868374
LMAN1	0.441425664	0.173390271
LMAN1L	-0.137616476	0.279849035
LMBR1	0.167599075	0.468154973
LMBR1L	0.360000637	0.02150143
LMBRD1	-0.134527753	0.704639673
LMBRD2	-0.534216633	0.163941079
LMCD1	-0.073025318	0.901235662

LMF2	0.134223756	0.680107403
LMNA	0.369890372	0.287977302
LMNB1	1.675236311	0.013628562
LMNTD2	0.086172413	0.562784931
LMO2	0.635399972	0.014623959
LMO4	-0.349991032	0.127500859
LMO7	-1.219394119	0.00659632
LMOD1	-0.08163238	0.89372279
LMOD2	0.112408493	0.252127612
LMTK2	-0.648925207	0.093938626
LMX1A	-0.032018319	0.735607376
LMX1B	-0.049569301	0.817473461
LNPEP	0.012467616	0.942587841
LNPK	0.121965904	0.62734035
LNX1	-0.480229919	0.1627352
LNX2	0.0281716	0.939257006
LONP1	0.321100643	0.098905266
LONP2	-0.0027421	0.990215424
LONRF1	0.680077049	0.028723695
LONRF2	-0.068637758	0.937340175
LONRF3	0.195280116	0.512902228
LOX	0.109407319	0.91707785
LOXHD1	-0.136010699	0.187190382
LOXL1	0.006861038	0.994960679
LOXL2	0.096166663	0.737435487
LOXL3	0.839223461	0.054772419
LOXL4	-0.122425287	0.429130133
LPA	-0.228834307	0.041131638
LPAR1	-1.035909565	0.081891813
LPAR2	0.322338616	0.201140539
LPAR3	-0.763158791	4.99E-05
LPAR4	0.196427629	0.571936389
LPAR5	0.582970704	0.083000608
LPAR6	0.560413667	0.009605955
LPCAT1	0.784213721	0.003865067
LPCAT2	-0.414884632	0.50326232
LPCAT3	0.519068616	0.01310888
LPCAT4	-0.925888408	0.027018172
LPIN2	0.444559618	0.049245411
LPL	1.596758713	0.003699961
LPO	-0.000484228	0.99800589
LPP	0.411164071	0.232643514
LRATD1	0.416704141	0.390778383
LRATD2	0.61150123	0.179198971
LRCH1	0.339931002	0.144671907
LRCH2	0.125197297	0.688976798
LRCH3	0.537663126	0.01107981
LRFN1	-0.300112879	0.156456644

LRFN2	-0.850711242	0.02965295
LRFN3	-0.221249207	0.203243282
LRFN4	0.206723746	0.209189667
LRFN5	-1.1373627	0.048357166
LRG1	0.050162083	0.748761184
LRGUK	1.429674376	0.004734877
LRIF1	0.29346681	0.271249661
LRIG1	0.506622962	0.144154077
LRIG2	0.736528215	0.005445074
LRIG3	0.207034455	0.729015453
LRIT1	-0.047632552	0.711938307
LRIT3	0.024354472	0.839668933
LRP1	0.687569495	0.003814444
LRP10	0.269221306	0.541275947
LRP11	-0.534998651	0.009605955
LRP12	-0.315617023	0.488604083
LRP2	-1.267244543	0.016106148
LRP3	-0.65608192	0.004334512
LRP5	0.24517649	0.555557328
LRP6	0.451895579	0.158572001
LRP8	-0.254085091	0.347139339
LRPAP1	0.031979699	0.871409385
LRPPRC	0.020130599	0.923050547
LRRC1	0.253656033	0.568613411
LRRC10	0.022013649	0.865610161
LRRC14	0.232571492	0.186594828
LRRC17	-0.133070216	0.798159671
LRRC19	-0.04481416	0.599302239
LRRC2	-0.455980672	0.259768536
LRRC20	-0.307060904	0.030107464
LRRC24	-0.135599408	0.296187084
LRRC25	0.330739279	0.117675287
LRRC26	-0.250956	0.115071693
LRRC28	-0.209311162	0.309608278
LRRC3	-0.419112342	0.011584437
LRRC31	-0.102087938	0.361878441
LRRC32	0.036959049	0.949249391
LRRC36	0.129444489	0.430198927
LRRC37B	0.640076356	0.046493806
LRRC38	-0.051987591	0.740466016
LRRC3B	-0.243350713	0.459874564
LRRC4	-0.260450902	0.506700199
LRRC40	-0.0328374	0.909061463
LRRC41	-0.039592114	0.827153531
LRRC42	0.191961736	0.491771018
LRRC45	0.345351073	0.019013819
LRRC46	0.367803699	0.294809012
LRRC47	0.062908315	0.803713486

LRRC52	0.025099564	0.821302228
LRRC53	-0.071075505	0.555003869
LRRC55	0.380354359	0.286021893
LRRC56	-0.169478234	0.188569462
LRRC57	0.200395838	0.268131236
LRRC59	0.401712566	0.271523767
LRRC6	-0.528286682	0.275707921
LRRC63	-0.210655781	0.278761589
LRRC7	-2.775202201	0.006623459
LRRC71	0.193749768	0.106201611
LRRC74A	-0.173058472	0.108333194
LRRC75B	0.325244802	0.028937394
LRRC8A	-0.23355068	0.258210179
LRRC8C	-0.035657763	0.852937413
LRRC8E	-0.104832166	0.639871932
LRRC9	0.094470599	0.822749558
LRRCC1	0.668961909	0.029502776
LRRD1	0.087816552	0.288509325
LRRFIP2	0.123499938	0.519338375
LRRK1	-0.14108421	0.778517241
LRRN1	0.74437527	0.104664313
LRRN2	-0.213918364	0.542419383
LRRN4	0.055618475	0.835312849
LRRTM1	-0.588815701	0.335906467
LRRTM3	-0.511985488	0.467076714
LRSAM1	0.244039088	0.173093618
LRTM1	-0.089128199	0.531969425
LRTOMT	-0.003543537	0.985975899
LRWD1	0.226896855	0.120156439
LSAMP	0.098441716	0.912618675
LSG1	0.575226375	0.005263345
LSM1	0.255468113	0.390444458
LSM10	-0.021209809	0.904792329
LSM11	-0.43935675	0.007734895
LSM12	0.236466534	0.149717662
LSM14B	0.133403717	0.243747753
LSM2	0.16507678	0.148693764
LSM3	0.163142821	0.546130841
LSM4	-0.003769469	0.989238507
LSM6	0.578687301	0.010673905
LSM7	0.147598217	0.589248097
LSM8	1.096559096	0.000447314
LSMEM1	0.35661168	0.059333582
LSMEM2	0.0504669	0.802667241
LSP1	0.242942405	0.21213973
LSR	-0.223460615	0.463668515
LSS	-0.212850379	0.510592076
LST1	0.427593789	0.014504857

LTA4H	0.261107991	0.363884132
LTB	0.0364007	0.837711214
LTB4R2	0.166033423	0.57259631
LTBP1	0.607786992	0.376723408
LTBP2	-0.148663874	0.790780478
LTBR	0.038893439	0.931287352
LTC4S	0.101253588	0.616944401
LTF	1.234361731	0.058853838
LTK	-0.172335298	0.171602494
LTN1	0.720784153	0.001192251
LTO1	-0.15107382	0.342374585
LTV1	0.361488958	0.229191542
LUC7L	0.810406156	0.002234491
LUC7L3	0.413443251	0.230896732
LUM	2.152305598	0.047567602
LURAP1L	-0.128775155	0.443916328
LUZP1	-0.368513499	0.093938626
LUZP4	-0.139021921	0.120390608
LVRN	0.070348089	0.596936607
LXN	0.121376399	0.657224321
LY6D	-0.053295912	0.583881916
LY6E	-0.286756692	0.315162033
LY6G5C	0.181353842	0.128184123
LY6G6D	0.098026766	0.28073347
LY6H	-0.508635427	0.018591189
LY86	1.16721675	0.040133496
LY96	0.571295682	0.082058452
LYAR	0.373189984	0.289399543
LYG1	0.552814078	0.012462671
LYG2	-0.133953115	0.295585953
LYL1	-0.206078145	0.240165425
LYN	0.701443462	0.110417153
LYPD1	0.942813465	0.093612208
LYPD2	0.079966806	0.712803392
LYPD3	0.063177081	0.693189389
LYPD5	-0.247588554	0.43487483
LYPD6B	-0.30200969	0.018909145
LYPLA1	0.961532152	0.032467866
LYPLA2	-0.077961243	0.655084643
LYPLAL1	0.581108284	0.008853854
LYRM1	-0.249087761	0.089876978
LYRM7	-0.154047838	0.56699906
LYSMD1	0.345119059	0.060533332
LYSMD2	-0.219643603	0.366016704
LYSMD4	-0.238294206	0.101437619
LYST	1.042356381	0.004734877
LYVE1	-0.312229224	0.532033706
LYZ	1.512905531	0.021842275

LYZL1	0.038062545	0.775232596
LYZL4	-0.138384638	0.301050499
LYZL6	-0.067581755	0.588301474
LZIC	0.204133854	0.373254452
LZTFL1	-0.013889523	0.963013757
LZTR1	0.195295093	0.255876294
LZTS1	0.008461478	0.989859482
LZTS2	-0.139331191	0.52082598
LZTS3	-0.707200948	0.037650934
M6PR	0.24993332	0.149621879
MAB21L1	0.13008212	0.507840804
MAB21L2	0.031942684	0.846680627
MAB21L3	-0.028533401	0.864990421
MAB21L4	-0.311026352	0.08103115
MACC1	-0.060056125	0.590337798
MACF1	0.124856843	0.557745885
MACROD1	0.189304197	0.107546114
MACROD2	-0.299940931	0.198781728
MAD1L1	0.343165219	0.043765298
MAD2L1	0.523419423	0.137758641
MAD2L1BP	-0.052616079	0.715729673
MAD2L2	0.495470547	0.048690547
MADCAM1	-0.132264412	0.436573225
MADD	-0.281474847	0.448614769
MAEL	0.144291562	0.543983376
MAF1	-0.272064342	0.111086676
MAFB	1.09110076	0.009676841
MAFF	0.293700158	0.255773292
MAFG	0.244841075	0.334004274
MAFK	-0.052588384	0.865012559
MAG	-2.288188397	0.005855705
MAGEA1	-0.010649505	0.944819609
MAGEA12	0.295358493	0.012129056
MAGEA6	-0.216713974	0.164586565
MAGEA9	0.040838288	0.767223379
MAGEB1	-0.200648644	0.053039043
MAGEB10	0.02295975	0.845746964
MAGEB18	-0.030048861	0.811792514
MAGEB2	0.026195573	0.862505915
MAGEB6	-0.154238363	0.196518218
MAGEC1	-0.232052724	0.058831789
MAGEC3	-0.15101249	0.196667281
MAGED2	0.029402554	0.931727556
MAGEE1	-0.665929302	0.033803615
MAGEE2	-0.120743155	0.383645488
MAGEF1	0.335500588	0.090149025
MAGEH1	-0.347120989	0.400453703
MAGEL2	-0.09263999	0.510426591

MAGI2	-0.256423161	0.298495618
MAGI3	-0.288616319	0.282546762
MAGIX	-0.102105839	0.331393811
MAGOH	0.484642188	0.013834874
MAGOHB	0.526854245	0.007294744
MAGT1	0.578441471	0.018182486
MAIP1	0.090916093	0.62738062
MAK16	0.369297401	0.063678818
MAL	-2.621226659	0.007734895
MAL2	-1.576869236	0.011530566
MALL	0.352557889	0.054564328
MALSU1	0.501893908	0.017090165
MALT1	-0.081803841	0.869514375
MAMDC2	-1.032948048	0.149011237
MAMDC4	0.53325871	0.007301526
MAML1	0.190645094	0.235861278
MAML2	1.592391364	0.00126644
MAML3	0.126286862	0.703788367
MAMLD1	0.369438782	0.24630937
MAN1A1	0.217249532	0.669807649
MAN1A2	-0.751893719	0.005620831
MAN1C1	0.743264894	0.107059158
MAN2A1	-0.76823155	0.033812808
MAN2A2	-0.424002031	0.132445969
MAN2B2	-0.222076701	0.406207444
MANBA	0.43500426	0.110821099
MANBAL	-0.269652856	0.278325567
MANEA	0.14900402	0.670013503
MANEAL	-0.008551727	0.985975899
MANF	0.602347016	0.013652873
MANSC1	-0.638773212	0.055487058
MAOA	-0.546133626	0.19092138
MAOB	0.499279555	0.405122436
MAP1A	-1.706058117	0.000978538
MAP1B	-0.361922219	0.192925508
MAP1LC3A	-0.703848815	0.00525333
MAP1LC3B	-0.079514158	0.632999192
MAP1LC3C	0.064598125	0.727142188
MAP1S	-0.29561297	0.217900828
MAP2	-0.058906152	0.956339036
MAP2K1	-0.511839018	0.147608175
MAP2K2	-0.079712014	0.712015348
MAP2K3	0.115340075	0.760468095
MAP2K4	-0.751727599	0.002899844
MAP2K6	0.16895712	0.654458918
MAP2K7	0.073463325	0.621056923
MAP3K10	-1.135823328	0.001597225
MAP3K11	-0.152879755	0.429997313

MAP3K13	0.07780721	0.749116117
MAP3K14	0.695936112	0.0092521
MAP3K15	-0.006589742	0.960926694
MAP3K19	0.28992871	0.315477007
MAP3K2	0.375401051	0.130313404
MAP3K20	0.563683445	0.213829425
MAP3K21	-0.84720598	0.000590251
MAP3K4	0.689512099	0.028932489
MAP3K5	0.044593147	0.931588813
MAP3K6	0.793022987	0.044464051
MAP3K7	0.296470084	0.023587466
MAP3K7CL	0.008732091	0.981263887
MAP3K8	0.668110455	0.093055812
MAP3K9	-1.098932068	0.000275651
MAP4	-0.1382536	0.566957609
MAP4K2	-0.524293532	0.00525333
MAP4K3	0.175889875	0.263089873
MAP4K4	-0.042114358	0.8957464
MAP4K5	-0.010506846	0.980883521
MAP6	-0.705366843	0.134840884
MAP6D1	-0.652875941	0.02848287
MAP7D1	-0.60633975	0.025589823
MAPK1	-0.054511431	0.842676174
MAPK10	-0.010077493	0.986045302
MAPK11	-0.414145433	0.032640805
MAPK12	-0.050729669	0.817338701
MAPK13	-0.194770166	0.456985798
MAPK14	0.060499152	0.736392111
MAPK15	-0.106595688	0.448267172
MAPK1IP1L	0.497329198	0.095757
MAPK6	-0.025017245	0.924838339
MAPK7	0.333223954	0.141254251
MAPK8	-0.228940144	0.458988629
MAPK8IP1	-0.741915499	0.036980829
MAPK8IP2	-1.713448979	0.001723718
MAPK8IP3	-0.604869804	0.113680124
MAPK9	-0.639959458	0.032425399
MAPKAPK2	0.337404391	0.243454284
MAPKAPK5	0.345668269	0.014096806
MAPRE1	0.360138572	0.064313369
MAPRE2	-0.635541295	0.016766817
MAPRE3	-0.435317975	0.196134898
MAPT	-0.525913222	0.490985728
MARCKS	1.064518018	0.000522307
MARCKSL1	-0.300099174	0.308064811
MARCO	0.115805797	0.404901065
MARF1	-0.320038253	0.160250505
MARK1	0.111368672	0.769049389

MARK4	-0.427150027	0.063240636
MARS2	0.200118773	0.258361037
MARVELD1	-0.015567142	0.975563879
MARVELD2	-0.21873343	0.173820085
MARVELD3	0.316987827	0.404567109
MAS1	-0.762367676	0.016930837
MASP1	0.534525543	0.124119423
MASP2	0.012859729	0.930658808
MAST1	-1.189237602	0.019088091
MAST2	0.190760465	0.292291784
MAT1A	-0.197223244	0.053517482
MAT2A	0.406883939	0.073415686
MAT2B	0.069283815	0.580848502
MATK	-0.884367442	0.000317125
MATN1	-0.051923693	0.775328942
MATN2	0.620749563	0.030011024
MATN3	0.164909042	0.419384428
MATN4	0.151508599	0.278845375
MAU2	0.424908736	0.043883128
MAVS	0.588392996	0.013652873
MAX	0.135217149	0.39532134
MB	-0.016259845	0.903601471
MB21D2	-0.190207567	0.521819382
MBD1	0.305977835	0.288905381
MBD2	0.394500083	0.035722703
MBD3L1	-0.026839032	0.78856472
MBD3L2	-0.053913669	0.659535419
MBD3L3	0.199844108	0.104500218
MBD3L4	0.211092232	0.069711301
MBD3L5	0.230492342	0.098615853
MBD4	0.494478236	0.015845112
MBD5	0.016992707	0.941983489
MBD6	0.805136002	0.00531946
MBIP	0.316010863	0.228531369
MBLAC2	0.061948864	0.837825198
MBNL1	0.254094954	0.120149895
MBNL2	-1.073785008	0.001788518
MBNL3	0.287103996	0.350262491
MBOAT2	0.378101324	0.288380428
MBOAT7	-0.945627542	0.000760638
MBTD1	0.521941501	0.023487605
MBTPS1	0.126223743	0.589670176
MBTPS2	0.194747025	0.610025615
MC1R	0.222982527	0.161821595
MC2R	-0.134084118	0.200225172
MC3R	-0.086357854	0.653942911
MC4R	-0.139509125	0.123229751
MC5R	0.075277707	0.670415029

MCAM	0.49297689	0.176570132
MCAT	-0.313461784	0.327149371
MCCC1	0.119602953	0.604369251
MCCC2	-0.003181931	0.992886227
MCEE	0.281083696	0.067503327
MCEMP1	-0.164116964	0.334714691
MCF2L2	-2.316157003	0.002438673
MCFD2	-0.008212564	0.981610177
MCHR1	0.248599719	0.455635494
MCL1	0.73170361	0.035197777
MCM10	0.609272809	0.222480605
MCM2	0.576074216	0.103444762
MCM3	1.248818232	0.001234389
MCM3AP	0.520765109	0.006576658
MCM4	0.963414158	0.005161235
MCM5	0.828794917	0.023377036
MCM6	0.871294996	0.020589821
MCM7	1.42741333	0.006902788
MCM8	1.043908483	0.020640868
MCM9	0.811155419	0.001121126
MCMBP	0.131433257	0.642354533
MCMDC2	0.003686009	0.980883521
MCOLN3	0.092073217	0.537956374
MCPH1	0.416753814	0.004922271
MCRIP1	-0.121368651	0.572051855
MCRIP2	-0.249782846	0.111085479
MCRS1	-0.181397612	0.223870308
MCTP1	-1.019622223	0.101704242
MCTP2	-0.06421956	0.844156784
MCTS1	0.055623462	0.740548264
MCU	-0.139573086	0.668207944
MCUB	1.350489879	0.004136181
MDFI	0.68633982	0.011530566
MDFIC	0.295852623	0.29545465
MDFIC2	-0.146561864	0.071387892
MDGA1	0.101145473	0.792979636
MDH1B	0.155216468	0.510240413
MDH2	0.044260632	0.788380786
MDK	0.467895232	0.513684677
MDM1	0.701111548	0.001581707
MDM2	0.357529261	0.449083281
MDM4	0.767281249	0.002015573
MDN1	-0.019949459	0.92118823
ME1	-2.301719735	0.000783213
ME2	0.140076303	0.637692473
MEA1	-0.305721893	0.152142681
MEAF6	0.002777259	0.984641673
MEAK7	-0.135084966	0.508181018

MECP2	0.226322908	0.038343532
MED1	0.383853406	0.324848474
MED10	0.152424552	0.347223044
MED11	-0.092647169	0.691079429
MED12	0.734972802	0.001029192
MED12L	0.156892115	0.738784898
MED13	0.447449306	0.070959226
MED13L	0.340954904	0.097216431
MED14	0.61210574	0.0087262
MED15	0.077548582	0.668355407
MED16	-0.109053824	0.552362529
MED17	0.425497396	0.044369981
MED18	-0.0365468	0.879784058
MED19	0.185240135	0.064669501
MED21	-0.147942735	0.368805325
MED22	0.220658368	0.210242433
MED23	0.180809471	0.257298754
MED24	0.179592719	0.207301042
MED25	-0.261580036	0.071356407
MED26	0.279854973	0.194885839
MED28	0.258747886	0.07015884
MED30	-0.040396435	0.79077225
MED31	0.06697483	0.635420059
MED4	0.251407933	0.170608267
MED6	0.107090572	0.518566194
MED7	0.161832311	0.174037106
MED8	-0.029937577	0.888347785
MED9	0.112254847	0.550809668
MEDAG	-0.485002716	0.371858821
MEF2A	0.126464984	0.58596993
MEF2D	-0.599795517	0.038777871
MEFV	-0.179182369	0.179584139
MEGF10	-0.91004501	0.188962339
MEGF11	0.331191735	0.541275947
MEGF8	0.177055347	0.485880843
MEI1	0.029364133	0.801763828
MEIS1	0.778774144	0.056396814
MEIS2	0.169753686	0.698036563
MEIS3	-0.345319944	0.206906615
MELK	0.740799933	0.179104238
MELTF	0.151736244	0.597800651
MEMO1	-0.087219706	0.547261362
MEOX1	-0.054719685	0.682323633
MEOX2	2.594201176	0.005162429
MEP1A	-0.345154301	0.07296748
MEP1B	-0.078504664	0.361269943
MEPE	-0.272768778	0.042395564
MERTK	1.01310953	0.090466736

MESD	0.384525182	0.006587985
MESP1	0.137544977	0.348555749
MEST	0.399908878	0.587626544
METAP1	0.122035549	0.669900902
METAP1D	0.676913743	0.003613651
METAP2	0.27032148	0.077279311
METRN	0.797459159	0.008749587
METRNL	0.550179102	0.218988848
METTL1	0.503425043	0.060787905
METTL11B	-0.038487509	0.664017397
METTL14	0.402790987	0.024618793
METTL15	0.225451034	0.250766162
METTL18	0.075493043	0.726883729
METTL21A	0.23689431	0.247372481
METTL22	0.713473189	0.00611638
METTL25	0.112026222	0.680307433
METTL26	-0.327313113	0.103117374
METTL27	-0.127946365	0.608979538
METTL2A	-0.14194017	0.600040873
METTL3	1.046752172	0.000352221
METTL5	0.191155276	0.259387781
METTL6	0.020014637	0.924838339
METTL7A	0.72488666	0.342122558
METTL7B	2.22984904	0.009260952
METTL8	0.172315299	0.53406091
METTL9	-0.140398984	0.692255382
MEX3B	-0.11467178	0.556314926
MEX3C	1.189395027	0.000763434
MEX3D	0.221943942	0.425370558
MFAP1	-0.095001357	0.669970192
MFAP2	-0.254062399	0.53132491
MFAP3	-0.01091283	0.981452955
MFAP3L	-0.14563888	0.628750571
MFAP5	-0.645128412	0.126239007
MFF	0.199910573	0.328697438
MFHAS1	0.262792932	0.094473358
MFN1	0.295406454	0.111086676
MFN2	-0.507805997	0.008503385
MFNG	0.45990168	0.041049838
MFSD1	0.152994249	0.344238136
MFSD12	0.25516271	0.401707582
MFSD13A	-0.316443814	0.138969461
MFSD14A	-0.159584466	0.3391134
MFSD14B	0.041367809	0.931276695
MFSD2B	-0.365724322	0.003361178
MFSD3	-0.286612917	0.091598211
MFSD4A	-1.426961333	0.024933462
MFSD4B	0.418406821	0.156600389

MFSD6	-0.946931457	0.016899346
MFSD6L	0.014355423	0.931777778
MFSD9	0.2342988	0.271505754
MGAM	0.132139599	0.511122664
MGAM2	0.038484704	0.843216557
MGAT2	0.218102766	0.558239715
MGAT4A	0.556425651	0.220939356
MGAT4D	-0.096670812	0.369103156
MGAT5	-0.113886817	0.673382618
MGAT5B	-1.595055802	0.002977765
MGLL	-0.487493702	0.085992211
MGME1	0.917891938	0.003979493
MGMT	-0.197207867	0.299633127
MGST1	-0.286624265	0.215378909
MGST2	0.214922628	0.25431304
MGST3	0.089255344	0.563019723
MIA2	-0.056983379	0.576345689
MIB1	-0.212569296	0.394695316
MIB2	-0.071929482	0.78917966
MICAL1	0.185604869	0.628563123
MICAL2	-1.896319866	4.88E-05
MICALL1	0.118210342	0.776400425
MICALL2	0.435354294	0.184534881
MICB	-0.09846898	0.706483988
MICOS13	-0.087234489	0.795166233
MICU1	-0.810696085	0.001443843
MICU2	0.029360105	0.906358055
MICU3	-0.718095038	0.013851792
MID1IP1	-0.307261725	0.38339972
MID2	-0.297465335	0.28207718
MIEF2	0.028628834	0.894416316
MIEN1	-0.465804831	0.005391307
MIER1	0.246004252	0.113283394
MIER3	0.550646419	0.045552482
MIF	0.11722027	0.758858286
MIGA1	-0.508632877	0.035530704
MIGA2	0.143430182	0.329463009
MIIP	0.639016684	0.000515458
MINDY1	0.417232314	0.229933371
MINDY3	-0.53767825	0.074642043
MINPP1	-0.047272341	0.863600504
MIOS	0.738247711	0.001380808
MIOX	-0.028583227	0.849863963
MIP	-0.057158658	0.656247396
MIPEP	0.158540358	0.373822015
MIS12	0.181956916	0.633441438
MIS18A	0.421349571	0.061914256
MIS18BP1	0.58658598	0.006080612

MISP	-0.093631137	0.437000037
MITD1	0.170371252	0.270340758
MITF	0.194041258	0.34209717
MIXL1	-0.043122895	0.755392658
MKI67	1.476070749	0.0442251
MKKS	-0.163034131	0.582804387
MKNK2	-0.189903444	0.530163682
MKRN1	0.143760985	0.420941497
MKRN2	-0.246289198	0.2793462
MKRN2OS	-0.180036287	0.141664338
MKRN3	0.704445921	0.085395644
MKS1	0.285900948	0.09845529
MKX	-0.437426995	0.074346764
MLANA	-0.14492245	0.177906786
MLC1	0.067398957	0.941091514
MLEC	0.505258798	0.072637354
MLF2	-0.414627186	0.006589628
MLH1	0.65898722	0.00042363
MLIP	-1.025290333	0.033864145
MLKL	0.541143515	0.082388609
MLLT1	-0.016142559	0.96394377
MLLT10	0.270202791	0.396982147
MLLT11	-1.136398315	0.024296673
MLLT3	-0.720323986	0.013945317
MLLT6	-0.128265478	0.74315299
MLN	-0.143006682	0.396261246
MLNR	0.059220523	0.650417176
MLPH	-0.465707379	0.103580058
MLST8	-0.334270365	0.12248967
MLX	0.093678408	0.629200991
MLXIP	0.66075352	0.016325061
MLXIPL	-0.552984283	0.044128583
MMAA	0.307636153	0.033678519
MMAB	-0.392477297	0.034402433
MMACHC	-0.164701396	0.225699149
MMADHC	-0.208209615	0.540803856
MMD	-0.376401813	0.481823479
MMD2	0.066974727	0.746528402
MME	0.051717554	0.911273041
MMEL1	0.167353576	0.537893051
MMGT1	0.383108465	0.139280151
MMP1	-0.162629644	0.462789052
MMP10	0.060431471	0.513233372
MMP11	0.103195418	0.449807208
MMP12	-0.052373951	0.915607882
MMP13	0.429328374	0.529438374
MMP14	1.967248549	0.012489096
MMP15	-0.129676958	0.780791174

MMP16	0.834473569	0.098985444
MMP17	-0.400626267	0.153950852
MMP19	0.382054623	0.351533159
MMP2	1.439541541	0.112001268
MMP20	-0.023097884	0.879526111
MMP21	-0.038930408	0.731357875
MMP24	-0.5584043	0.128542375
MMP24OS	-0.475253367	0.108773057
MMP25	-0.027022922	0.805642311
MMP26	-0.239010566	0.019807497
MMP27	0.051565623	0.599302239
MMP28	-0.07117526	0.746400777
MMP3	-0.16149796	0.679418222
MMP7	0.285559398	0.267530519
MMP8	0.041710734	0.709670769
MMP9	1.587645251	0.026115806
MMRN1	0.121318587	0.556759506
MMRN2	-0.140085444	0.350828503
MMS19	-0.193993817	0.30488121
MMS22L	0.482267779	0.039219877
MMUT	0.052199119	0.794902982
MN1	-0.844035403	0.027925047
MNAT1	-0.033756827	0.868709798
MND1	0.80133194	0.079742987
MNDA	0.867725254	0.011164715
MNS1	1.104477274	0.002856643
MNT	0.011784437	0.974469226
MNX1	0.028262295	0.833863145
MOAP1	-0.959390586	0.021163769
MOB1A	0.463025052	0.09769566
MOB3B	-0.24677892	0.685080047
MOB3C	0.353503592	0.013945317
MOCOS	-0.028305115	0.917640056
MOCS2	-0.083912992	0.767618297
MOCS3	0.002922405	0.994668339
MOG	-2.011219625	0.017720052
MOGAT1	-0.076365262	0.422314338
MOGAT2	-0.038277241	0.718234946
MOGAT3	-0.002553384	0.993053028
MOGS	0.145947466	0.591868721
MOK	0.400018905	0.115683548
MON1A	-0.232677975	0.076356381
MON1B	-0.017576775	0.952956034
MORC1	0.041970111	0.708923274
MORC2	0.811204582	0.001577058
MORC3	0.529453514	0.009852914
MORC4	0.523337348	0.297642305
MORF4L1	-0.103558429	0.564973097

MORN1	0.385828106	0.01673506
MORN2	0.196651325	0.347416854
MORN3	0.358885689	0.096054031
MORN4	-0.495784132	0.059444546
MORN5	0.206156862	0.164580353
MOS	-0.071307441	0.595349657
MOSMO	-0.203794683	0.241986251
MOSPD1	0.089194359	0.870307862
MOSPD2	0.026606667	0.932391251
MOSPD3	0.085700825	0.749875433
MOV10	1.039978422	0.006663931
MOXD1	0.745434221	0.331307117
MPC1	0.197710462	0.5208979
MPDU1	0.078765363	0.624020772
MPG	-0.054153751	0.761907185
MPHOSPH10	0.067704601	0.687883599
MPHOSPH6	-0.173442291	0.356472461
MPHOSPH8	0.127377045	0.665904933
MPHOSPH9	0.376320273	0.051261944
MPI	-0.439016363	0.013277259
MPIG6B	-0.055437571	0.709670769
MPL	-0.201150661	0.253589723
MPLKIP	0.436317995	0.025598194
MPND	-0.231991693	0.181734674
MPO	-0.068315077	0.601656239
MPP2	-0.446853296	0.016268029
MPP3	-0.047603791	0.870307862
MPP4	-0.351088798	0.118872519
MPP5	-0.214719876	0.362028252
MPP6	-0.563583823	0.168721983
MPP7	-0.839342072	0.003133494
MPPED1	-1.703982986	0.002015573
MPPED2	0.023955386	0.965491836
MPRIP	-0.268210163	0.12248967
MPV17L	0.357604123	0.444953362
MPV17L2	-0.018492742	0.935244061
MPZ	0.283903121	0.118207209
MPZL1	0.401813346	0.119624812
MPZL2	0.327030019	0.11193036
MPZL3	0.150280949	0.241451972
MR1	0.661528179	0.017910438
MRAP	0.104165728	0.350437351
MRAP2	-0.511888803	0.084833849
MRC2	1.772180771	0.017818426
MRE11	0.474843104	0.007026076
MREG	0.393992263	0.103147355
MRFAP1	0.001461236	0.993549673
MRFAP1L1	0.176644679	0.536369113

MRGBP	0.145976196	0.549312728
MRGPRD	-0.005545825	0.982287269
MRGPRX1	-0.107733034	0.491946753
MRGPRX2	0.007778062	0.960782206
MRGPRX3	-0.023874253	0.875321102
MRGPRX4	-0.124431083	0.293521592
MRM1	0.213323933	0.382183645
MRM2	0.452789173	0.039866889
MRM3	-0.04161944	0.872992957
MRNIP	0.348175159	0.061495759
MRO	-0.559395258	0.421486967
MROH2A	-0.066854798	0.425384977
MROH2B	-0.163527828	0.169499731
MROH6	0.138305994	0.218851069
MROH8	0.102222013	0.661783029
MROH9	-0.019666503	0.863457672
MRPL1	-0.078490903	0.582941588
MRPL11	0.46695847	0.001392079
MRPL13	0.216251055	0.247709332
MRPL14	0.206003424	0.31754923
MRPL15	0.0221439	0.94671625
MRPL16	0.096566742	0.620741982
MRPL17	0.089009941	0.780791174
MRPL18	0.057814835	0.784105431
MRPL19	-0.085600075	0.668259657
MRPL2	-0.179140517	0.262151746
MRPL21	0.067865401	0.69189988
MRPL22	0.026040066	0.846369748
MRPL23	-0.162597668	0.144002691
MRPL24	0.342942709	0.030218755
MRPL27	0.310762418	0.026485864
MRPL28	-0.036699708	0.845168366
MRPL3	0.365628948	0.144197979
MRPL32	0.338770317	0.044164454
MRPL33	0.03790238	0.840155689
MRPL34	-0.396682249	0.014948728
MRPL35	-0.085823282	0.72777771
MRPL36	0.447732731	0.029057814
MRPL37	0.063894944	0.802746793
MRPL38	0.072992519	0.636452006
MRPL39	0.240661187	0.245147205
MRPL4	-0.085433917	0.742197286
MRPL40	0.104814652	0.682117201
MRPL41	-0.967293369	0.00064817
MRPL43	-0.220950554	0.407647032
MRPL44	0.088923722	0.697525606
MRPL46	0.436276593	0.034900685
MRPL47	0.101871416	0.57381297

MRPL48	-0.135109688	0.382458915
MRPL50	0.136459266	0.649271943
MRPL51	0.102691445	0.685940819
MRPL52	0.234515558	0.254059985
MRPL54	-0.178999363	0.338352185
MRPL55	0.214220701	0.259993881
MRPL57	-0.203493315	0.32527098
MRPL58	0.049169086	0.893402675
MRPL9	0.045455816	0.845379815
MRPS10	0.018388847	0.956825161
MRPS11	0.043369107	0.857392501
MRPS12	-0.29126099	0.181118964
MRPS15	-0.256227418	0.134404551
MRPS16	-0.060128579	0.861347321
MRPS17	0.170086294	0.537165089
MRPS18A	0.522876895	0.027091279
MRPS18B	-0.218596208	0.078394754
MRPS18C	0.242539651	0.046119664
MRPS22	-0.063657544	0.676429122
MRPS23	0.095512708	0.631657215
MRPS25	0.588244657	0.03791286
MRPS26	0.072922181	0.746769428
MRPS27	0.084300285	0.737031972
MRPS28	0.849873636	0.001359112
MRPS30	-0.364087366	0.056755453
MRPS31	0.093587894	0.653644729
MRPS33	0.483162306	0.0084556
MRPS34	0.020241875	0.937826236
MRPS35	-0.225822549	0.298479913
MRPS36	-0.049103995	0.838917532
MRPS7	0.207991037	0.25302897
MRPS9	0.099505484	0.563836061
MRRF	0.290919113	0.2371933
MRS2	0.384226959	0.115203724
MRTFA	2.26E-05	0.999891414
MRTO4	0.33777344	0.254727402
MS4A1	0.104348663	0.403165014
MS4A10	-0.075547164	0.583914788
MS4A12	-0.084923665	0.521837798
MS4A14	1.771882575	0.000962015
MS4A18	-0.050355226	0.665904933
MS4A2	-0.064354796	0.541275947
MS4A3	-0.000239341	0.998128955
MS4A4E	0.57474343	0.1038076
MS4A6A	1.425289762	0.004981702
MS4A6E	-0.067411241	0.506293122
MS4A8	-0.000812991	0.995560506
MSANTD2	0.473801125	0.009267208

MSC	-0.460622296	0.197549066
MSH2	0.03067711	0.917640056
MSH4	0.02750191	0.903309166
MSH5	1.681843174	0.00032518
MSI1	0.897623213	0.019354786
MSI2	0.762203993	0.020517538
MSL1	0.305330579	0.182130477
MSL2	0.248955139	0.161281614
MSL3	-0.059009878	0.753310146
MSLN	0.050458581	0.733411121
MSMO1	-1.198756875	0.01413174
MSN	0.619734598	0.201492147
MSR1	0.636387718	0.005391307
MSRA	0.704044758	0.053815504
MSRB1	-0.039173172	0.881600562
MSRB2	-0.176358982	0.517107433
MST1	0.920924559	0.006663931
MST1R	-0.086270847	0.467466505
MSTN	0.685938651	0.21860477
MSX1	0.313223207	0.040863834
MSX2	-0.086870662	0.668259657
MT1A	-0.679675224	0.251616112
MT1B	-0.265158493	0.740118086
MT1E	0.362163318	0.772614814
MT1G	-1.578316921	0.027370254
MT1X	0.863138702	0.059243634
MT2A	0.153647748	0.685220872
MT4	0.504432074	0.010201971
MTA1	0.615188682	0.013504955
MTA3	-0.089727042	0.658552671
MTAP	-0.01969	0.971953702
MTBP	0.919279622	0.000826086
MTCH1	-0.537629519	0.011942164
MTCH2	0.013593461	0.976695706
MTCL1	-0.21590866	0.487776294
MT-CO1	-0.359430589	0.217755292
MT-CO2	-0.16095136	0.069377141
MT-CO3	-0.179603636	0.02169531
MT-CYB	-0.178776167	0.038675257
MTDH	0.126333139	0.510174916
MTERF1	0.500964886	0.019659172
MTERF2	0.668997108	0.007845807
MTERF3	0.459992517	0.00553036
MTF1	0.265836732	0.152836452
MTF2	0.565126975	0.011531342
MTFMT	0.037558329	0.923498499
MTFP1	0.333846028	0.151626426
MTFR1	0.271824409	0.251373836

MTG2	0.527528531	0.002977765
MTHFD1	0.365596871	0.066907653
MTHFD1L	0.235969216	0.379504989
MTHFD2	0.539550109	0.193669061
MTHFR	0.149490997	0.187203129
MTIF3	0.041835901	0.832259949
MTLN	-0.034909527	0.707337139
MTM1	0.058960593	0.84669214
MTMR10	-0.263403624	0.558876753
MTMR3	0.286450699	0.163194123
MTMR4	-0.123332734	0.523034082
MTMR6	-0.29970345	0.17447459
MTMR8	-0.151334157	0.222119034
MTMR9	-0.015919984	0.96045146
MT-ND1	-0.299261209	0.048587206
MT-ND2	-0.126444015	0.240869834
MT-ND3	-0.379339424	0.119624812
MT-ND4	-0.249035388	0.27519833
MT-ND5	-0.092932017	0.196258018
MT-ND6	0.239873668	0.604126128
MTNR1A	-0.056833179	0.737435487
MTNR1B	-0.132201252	0.339439747
MTO1	0.398519875	0.03849907
MTOR	0.177998393	0.320453101
MTPAP	-0.146584692	0.615329823
MTR	0.507492457	0.007327295
MTREX	0.394132264	0.042219069
MTRF1	0.439718179	0.003806486
MTRF1L	0.165414703	0.556759506
MTRR	0.048342969	0.736206294
MTSS2	0.172666013	0.761779554
MTURN	-1.468290964	0.016084161
MTUS1	-0.246978659	0.493223215
MTX1	0.070397479	0.806150668
MUC13	-0.06727969	0.491062174
MUC15	-0.1502485	0.099259087
MUC17	-0.135018281	0.197460117
MUC7	-0.075757018	0.391833864
MUCL1	-0.059563164	0.624611101
MUCL3	-0.003056528	0.987724044
MUL1	0.197068603	0.45270404
MUSTN1	0.63552814	0.035047058
MVB12A	0.493028712	0.004305912
MVD	-0.398186909	0.026485864
MVK	-0.386600217	0.041354836
MX2	0.72445315	0.012733817
MXD1	0.28193677	0.128184123
MXD3	0.568667669	0.062868967

MXD4	0.109876242	0.609719783
MXI1	-0.469159744	0.150454182
MXRA5	1.064721447	0.172583942
MXRA7	-0.242189332	0.546766193
MYBBP1A	0.012558975	0.969472548
MYBL2	0.347964637	0.453795581
MYBPC2	-0.021925445	0.911188953
MYBPC3	-0.029695834	0.787364885
MYBPH	0.507605925	0.031179777
MYC	0.461496651	0.364416562
MYCBP2	-0.532905732	0.15119423
MYCBPAP	0.210360102	0.273075708
MYCN	0.223649675	0.69189988
MYCT1	0.129967835	0.780791174
MYDGF	0.462919138	0.22888704
MYEF2	0.656230183	0.037188291
MYEOV	-0.026387483	0.898425329
MYF5	-0.002967184	0.981957792
MYF6	-0.095976653	0.336404722
MYH1	-0.229660008	0.080062331
MYH13	0.038831557	0.741269852
MYH3	0.263770338	0.112446941
MYH6	-0.072780503	0.614816249
MYH8	-0.049677811	0.694869454
MYH9	0.163615034	0.810477716
MYL1	-0.11170516	0.398421989
MYL12A	0.206726723	0.70248201
MYL2	0.004329651	0.97681392
MYL3	-0.241445375	0.032576429
MYL4	-0.116758563	0.564532083
MYL5	0.057923405	0.822572635
MYL6B	0.179737494	0.521819382
MYL7	-0.037723691	0.806069995
MYL9	-0.311833901	0.739647832
MYLIP	0.217305723	0.631894147
MYLK	-0.66271021	0.099429122
MYLK2	0.029671175	0.815620046
MYLK3	-0.110740977	0.496517196
MYLPF	-0.268204178	0.052010099
MYO10	0.112605883	0.655671159
MYO15A	-0.029697186	0.921556503
MYO18A	-0.371691926	0.130707586
MYO18B	-0.054735373	0.675605501
MYO1D	-1.503085732	0.01743244
MYO1E	0.15287445	0.784482378
MYO1F	1.292663729	0.003020661
MYO3A	0.011402356	0.948191138
MYO5A	-0.969701578	0.014988328

MYO5C	0.127729098	0.779159502
MYO6	-0.25400045	0.405603417
MYO7A	0.810464026	0.004375723
MYO9A	-0.191999814	0.379419649
MYO9B	0.977881974	0.002509722
MYOC	0.023141495	0.853715871
MYOD1	-0.333507595	0.011141587
MYOF	0.410802577	0.670732222
MYOG	-0.221170336	0.03718731
MYOM1	-0.250882783	0.232654739
MYOM2	-0.319180927	0.174391138
MYOM3	-0.074071462	0.436059122
MYORG	-1.150179622	0.001954005
MYOT	-0.138598618	0.599302239
MYOZ1	0.107271123	0.533045121
MYOZ2	-0.417794937	0.228630078
MYPOP	-0.216079836	0.179527591
MYRF	-1.076785075	0.039918203
MYRIP	-1.08635461	0.012370013
MYT1	1.025951461	0.001312969
MZB1	0.106965412	0.473341158
MZF1	0.351347758	0.215246072
MZT2A	0.066509568	0.672303356
MZT2B	-0.227735049	0.21688337
N4BP1	-0.233359418	0.34117108
N4BP2	1.45682632	4.88E-05
N4BP2L1	0.063205961	0.841948776
NAA10	0.457492834	0.009605955
NAA11	-0.025752787	0.815620046
NAA15	0.346109783	0.192422457
NAA20	0.52266076	0.017231121
NAA25	0.561933853	0.006497556
NAA30	-0.086172546	0.686333481
NAA35	0.379681405	0.043883128
NAA38	-0.610771597	0.009403536
NAA40	0.580478838	0.005161235
NAA50	0.314273296	0.101117251
NAA80	0.086281479	0.548219322
NAAA	0.189842835	0.468439363
NAALAD2	-0.090872212	0.870418435
NAALADL1	-0.118349017	0.455635494
NAALADL2	0.377738768	0.079742987
NAB1	0.188385275	0.191008249
NAB2	0.306472754	0.176247091
NABP2	-0.102938654	0.461156719
NACA2	-0.185986635	0.335380845
NACC1	-0.786480574	0.002840781
NACC2	-0.044090137	0.897484194

NADSYN1	0.237751829	0.240142314
NAF1	0.128870224	0.563322948
NAGA	0.376397069	0.14118057
NAGK	0.08368651	0.729473097
NAGPA	-0.841132846	0.002565396
NAGS	-0.19105884	0.169782421
NAIF1	-0.084781042	0.514971301
NAIP	0.830001018	0.074419366
NALCN	-1.483438153	0.053502686
NAMPT	2.303114694	0.00044063
NANOS1	0.109780945	0.547868374
NANOS3	-0.250149632	0.201277247
NANP	0.176814744	0.394488685
NANS	0.025624321	0.930110116
NAP1L1	0.1589788	0.366900124
NAP1L2	-1.856742588	0.021330379
NAP1L3	-1.55994407	0.012000691
NAP1L4	0.284415048	0.168721983
NAP1L5	-1.287044318	0.004947879
NAPA	-0.820873623	0.013635119
NAPB	-1.15712829	0.120156439
NAPG	-0.498513129	0.051240229
NAPRT	0.042267951	0.837528581
NAPSA	-0.128359557	0.262254936
NARF	-0.015743108	0.940956702
NARS2	0.112963965	0.606594523
NASP	0.94752168	0.003480618
NAT10	0.31670189	0.040695726
NAT14	-0.105973083	0.60686531
NAT16	0.016673903	0.923498499
NAT2	-0.017543611	0.886782715
NAT8	0.150703744	0.273829296
NAT8L	-0.88750966	0.086250039
NAT9	0.284332143	0.17392419
NAV1	0.049131642	0.885029016
NAV3	-1.250108396	0.026520722
NAXD	-0.100764987	0.677252505
NAXE	-0.162039467	0.520406633
NBAS	0.175141446	0.26224889
NBEA	-0.817259665	0.149717662
NBN	0.242320877	0.110456596
NBPF3	0.307248885	0.159863387
NCAM2	-0.425434607	0.696913847
NCAN	0.346875218	0.714925665
NCAPD2	0.830211693	0.017835001
NCAPD3	0.999612417	0.000162776
NCAPG	1.660240025	0.030103304
NCAPG2	1.212437306	0.00186252

NCAPH	0.794212995	0.095677807
NCAPH2	-0.034488653	0.896651938
NCBP1	0.603329909	0.003519155
NCBP2	0.629881225	0.003568303
NCCRP1	0.089053609	0.623455279
NCDN	-0.827455044	0.036838113
NCF1	-0.02656418	0.907973851
NCF2	0.687333201	0.01827676
NCF4	0.680486507	0.071002153
NCK1	0.893567433	0.000511553
NCK2	0.154904875	0.563836061
NCKAP1	-0.257769965	0.384889755
NCKAP1L	1.693050317	0.003132413
NCKIPSD	-0.525117077	0.040245209
NCL	0.315821748	0.03424149
NCLN	0.137886482	0.652822208
NCMAP	-0.085889015	0.566497648
NCOA1	0.105378594	0.792979636
NCOA2	0.126756132	0.647135908
NCOA6	0.40801141	0.030129121
NCOR1	0.167017731	0.36727798
NCOR2	0.382189326	0.158857317
NCR1	-0.308151971	0.0092521
NCR2	-0.062328579	0.737836453
NCS1	-2.081718705	0.00044063
NCSTN	0.249562825	0.29981591
NDC1	0.622109405	0.044112796
NDC80	0.887788003	0.044164454
NDFIP1	-0.249712051	0.293632899
NDN	-0.225939763	0.335921879
NDNF	0.414775839	0.242578315
NDP	-0.069318174	0.885029016
NDRG2	-0.20647679	0.800675224
NDRG3	-1.105620852	0.011584437
NDST2	-0.214390565	0.152360788
NDST4	-0.033753357	0.846906695
NDUFA1	0.116507172	0.696319433
NDUFA10	-0.10202129	0.631657215
NDUFA12	-0.007486964	0.956825161
NDUFA13	0.266945514	0.316898088
NDUFA3	-0.114902321	0.499086044
NDUFA4	0.211502576	0.582804387
NDUFA4L2	0.312348688	0.188154722
NDUFA5	0.189393038	0.396977946
NDUFA6	-0.119180856	0.415378611
NDUFA7	-0.065982497	0.691932545
NDUFA8	-0.047589001	0.758628178
NDUFA9	-0.348549113	0.049134091

NDUFAB1	-0.334252168	0.175534976
NDUFAF2	-0.163174656	0.307687889
NDUFAF3	-0.255842869	0.072620438
NDUFAF4	0.090169389	0.596519233
NDUFAF5	0.07146446	0.654458918
NDUFAF6	0.429406829	0.006880123
NDUFAF7	-0.058654823	0.692469824
NDUFB1	-0.097091905	0.663793359
NDUFB10	0.156847348	0.388913223
NDUFB11	-0.05760592	0.746856269
NDUFB2	-0.013827364	0.941983489
NDUFB3	-0.366672067	0.023266695
NDUFB5	-0.345632595	0.143520741
NDUFB6	-0.187367245	0.488387975
NDUFB7	-0.221155158	0.313065913
NDUFC1	-0.281495651	0.61937456
NDUFS4	0.304093685	0.115683548
NDUFS5	-0.071759256	0.656698376
NDUFS6	0.567278428	0.001541164
NDUFS7	-0.173218653	0.229933371
NDUFS8	-0.145282574	0.359730415
NDUFV2	0.109784366	0.446507026
NDUFV3	0.040278831	0.82863186
NEBL	-0.827592805	0.074642043
NECAB1	-3.038551072	0.001121126
NECAB2	-0.854819411	0.113991261
NECAB3	-0.44211009	0.009605955
NECAP1	-0.254105788	0.532865057
NECTIN1	-0.638068608	0.001456266
NECTIN4	-0.076981868	0.531569134
NEDD1	1.102355692	0.007519601
NEDD4	0.58333921	0.193355898
NEDD8	-0.29264419	0.12071573
NEDD9	0.189665925	0.621717543
NEFH	-1.640317552	0.00305093
NEFL	-4.350784482	0.00117745
NEFM	-3.959620049	0.000612048
NEGR1	-2.16375082	0.002062218
NEIL1	-0.056633039	0.924763137
NEIL2	0.165895629	0.278760312
NEIL3	0.458701025	0.250357815
NEK11	0.546935191	0.072687194
NEK3	0.121546866	0.675082673
NEK4	0.428231539	0.033877755
NEK5	0.333537856	0.325721307
NEK7	-0.430052202	0.281639201
NEK8	0.217258147	0.20444268
NELFA	0.305706231	0.034369324

NEMF	-0.155534264	0.375141671
NEMP2	0.546972023	0.033469262
NENF	-0.115462629	0.553754223
NEO1	-0.804356324	0.023259186
NEPRO	0.287108241	0.114056713
NES	3.041023392	0.00015288
NETO1	-1.934212329	0.03718731
NETO2	-0.549970182	0.491242591
NEU1	0.152889685	0.458913885
NEU2	-0.067246396	0.689918805
NEU3	-0.125656139	0.410772979
NEURL1	-1.29306701	0.001768875
NEURL2	0.104198534	0.656698376
NEUROD1	0.102038658	0.69476983
NEUROD2	-1.208804755	0.016812375
NEUROD4	0.184072891	0.239336125
NEUROD6	-1.280629442	0.015101119
NEUROG1	-0.061640678	0.653672898
NEUROG2	-0.09962726	0.402527494
NEUROG3	-0.078878446	0.679193729
NEXN	-0.181145512	0.806150668
NF2	-0.918507755	0.001544618
NFAM1	0.082934188	0.736206294
NFAT5	-0.201509679	0.291653221
NFATC1	0.369005904	0.114290385
NFATC2	0.691684354	0.096749968
NFATC3	-0.097081947	0.543228323
NFE2L1	-0.214953152	0.232229466
NFE2L3	-0.357221118	0.487841801
NFIA	0.60717495	0.203243282
NFIX	-0.080321013	0.845167694
NFKB1	0.461488414	0.052529273
NFKB2	0.126652359	0.682117201
NFKBID	0.18998099	0.440385522
NFKBIL1	0.33704935	0.056043915
NFKBIZ	0.970489196	0.065470534
NFRKB	0.628861391	0.00390644
NFU1	0.166212376	0.347139339
NFX1	0.129892112	0.481196182
NFXL1	0.524583953	0.021490743
NFYB	-0.198941562	0.457077302
NGB	-0.334963964	0.040433822
NGDN	0.033784526	0.840369145
NGF	-0.420091976	0.153482869
NHEJ1	0.108145858	0.674019191
NHLH1	0.147809783	0.561776185
NHLH2	0.001686136	0.991171509
NHLRC1	-0.316795364	0.086144429

NHLRC2	0.271494873	0.281492733
NHLRC4	0.004573247	0.980108718
NHP2	-0.218113917	0.159585663
NHS	-0.361172536	0.363424132
NIBAN1	0.812532777	0.233002726
NIBAN2	0.260990789	0.702025588
NIBAN3	-0.016489501	0.909624464
NICN1	-0.132235226	0.569679712
NID1	1.847029433	0.001192251
NID2	0.694125391	0.411087048
NIF3L1	0.050452152	0.661709156
NIFK	0.63800329	0.04800644
NIM1K	0.531529783	0.019105374
NIN	0.219753579	0.466112968
NINJ1	0.432754186	0.042609029
NINJ2	-0.383611108	0.10827865
NINL	0.478504712	0.229158738
NIPA1	-1.037388185	0.001391257
NIPAL1	-0.016654978	0.91445925
NIPAL2	-0.840402465	0.0795112
NIPAL3	-1.373039152	0.000879799
NIPBL	0.273198141	0.166880626
NIPSNAP1	-0.680217538	0.017898594
NIPSNAP2	0.396484545	0.16993895
NIPSNAP3A	-0.09601163	0.643603569
NISCH	-0.271460589	0.422978779
NIT2	0.688830325	0.000484554
NKAIN1	-0.410774874	0.239273188
NKAIN3	-0.428747165	0.359128631
NKAIN4	0.558441637	0.11456237
NKAP	-0.068247459	0.646187802
NKAPD1	0.58412601	0.000592196
NKD1	0.587050203	0.239336125
NKD2	0.001454879	0.995181038
NKG7	0.176868785	0.208683647
NKIRAS1	-0.138263544	0.64686504
NKIRAS2	0.267654449	0.507144787
NKPD1	0.185952443	0.049245411
NKTR	0.671745711	0.002670906
NKX1-1	-0.183234554	0.404095154
NKX1-2	-0.042146726	0.819044807
NKX2-1	0.062343374	0.716141513
NKX2-2	0.754880045	0.090602006
NKX2-3	-0.088674427	0.468354165
NKX2-4	-0.171823381	0.149257703
NKX2-8	-0.103179501	0.433486087
NKX3-1	0.020438776	0.919743362
NKX3-2	-0.14329331	0.467466505

NKX6-1	-0.006449602	0.981452955
NKX6-2	-1.414865989	0.016817157
NKX6-3	-0.124919193	0.444694243
NLGN1	0.8479453	0.087673335
NLGN2	-0.221969533	0.499195822
NLGN4X	0.239051433	0.243747753
NLK	-1.167233564	0.009481506
NLN	0.079135087	0.759850228
NLRC3	0.216245569	0.183211794
NLRC4	0.102646812	0.336280026
NLRC5	0.882376162	0.009605955
NLRP1	-0.184464832	0.682117201
NLRP10	-0.06216749	0.631366022
NLRP11	-0.070254569	0.503416338
NLRP12	0.26545497	0.169315489
NLRP13	-0.146189408	0.212467368
NLRP14	-0.060412774	0.577387987
NLRP2	-0.241641146	0.056328293
NLRP3	0.63272112	0.090889195
NLRP4	0.054532251	0.642247046
NLRP5	-0.115425153	0.353410067
NLRP6	0.143726127	0.311487033
NLRP8	-0.015187466	0.905209049
NLRP9	-0.021753162	0.807436219
NLRX1	0.225802701	0.079836405
NMB	0.844157273	0.005202403
NMBR	-0.011954314	0.927976981
NMD3	0.350819903	0.057924219
NME3	-0.118439411	0.503836731
NME4	0.134835007	0.374930374
NME5	-0.19432832	0.51552441
NME6	0.001226024	0.995560506
NME9	-0.069308447	0.716141513
NMI	0.886714015	0.000511553
NMNAT1	0.112199029	0.490985728
NMNAT2	-2.116236115	0.011015862
NMRAL1	-0.000485683	0.998128955
NMRK2	-0.065699798	0.655976143
NMT1	0.061577982	0.752408133
NMT2	-0.65958492	0.008424217
NMU	-0.011374365	0.959775794
NMUR1	0.020874877	0.86599572
NMUR2	0.096310028	0.694260995
NNAT	-1.016536205	0.215582556
NNMT	0.995042532	0.061048788
NNT	-0.28629697	0.135264343
NOA1	0.124733132	0.71179902
NOB1	0.324676535	0.263668704

NOC2L	0.357742877	0.100754919
NOC3L	0.142931514	0.690801599
NOC4L	0.200900179	0.280909659
NOD1	0.926789248	0.006691815
NOD2	0.336876654	0.10691656
NODAL	0.160958094	0.574020138
NOG	0.046161742	0.893241153
NOL10	0.400698385	0.160303172
NOL11	0.712191114	0.060353236
NOL12	0.222062093	0.687005925
NOL4	0.031777042	0.968512656
NOL6	-0.286574007	0.314100734
NOL7	0.158643767	0.320067283
NOL9	0.127913312	0.584436904
NOLC1	-0.343838053	0.121791104
NOMO1	-0.115026346	0.651747521
NOMO2	-0.182941764	0.423060214
NOP10	0.169653139	0.675605501
NOP14	0.828675504	0.001625823
NOP53	-0.232064338	0.568155097
NOP58	0.457173875	0.052182985
NOP9	0.438188304	0.028359001
NOS1AP	0.059076877	0.884067303
NOS2	-0.021429272	0.958789823
NOS3	0.121266912	0.407647032
NOSIP	-0.050950457	0.872447647
NOTCH1	1.143032961	0.001620462
NOTCH2	0.593120604	0.004136181
NOTCH2NLA	1.253466786	0.000964515
NOTO	-0.173558648	0.263089873
NOTUM	-0.307355504	0.058622294
NOVA1	1.850843062	0.001613537
NOVA2	-0.335907068	0.298590553
NOX1	-0.16862607	0.16763097
NOX3	-0.129925295	0.252986183
NOX4	0.059190179	0.896359141
NOXA1	-0.211179482	0.386559646
NOXO1	0.052624735	0.692614306
NPAP1	-0.316298914	0.109707043
NPAS1	-0.418382179	0.007786557
NPAS2	-0.340157554	0.344781274
NPAS4	-0.645366043	0.192300701
NPAT	0.546610837	0.015541204
NPB	-0.256284097	0.056433133
NPBWR1	-0.218071832	0.113431835
NPBWR2	-0.158657075	0.278481075
NPC1	-0.207885167	0.546130841
NPC1L1	-0.047854546	0.696778149

NPC2	0.58105116	0.170127874
NPDC1	-0.187188978	0.549686574
NPEPPS	0.034322028	0.860613559
NPFF	0.703586386	0.000393501
NPFFR1	-0.456578738	0.08658043
NPHP1	0.730693394	0.004300012
NPHP4	0.173732479	0.268931287
NPHS1	-0.154520435	0.203503156
NPHS2	-0.119493285	0.242361174
NPIPA1	0.345599095	0.280909659
NPIPB2	0.131605128	0.723535226
NPIPB4	0.372619636	0.190798322
NPIPB5	0.34535621	0.274024801
NPL	1.163871993	0.002150168
NPLOC4	0.075560571	0.765796187
NPM1	0.392547842	0.036291465
NPM2	-1.025583645	0.001776331
NPM3	-0.227901715	0.203243282
NPPA	0.220736842	0.278333668
NPPB	-0.737685816	0.139569652
NPPC	-0.317449514	0.06041
NPR1	0.212565854	0.047047799
NPR2	0.144017908	0.559687792
NPRL2	0.091375306	0.473415288
NPSR1	0.450499868	0.514120611
NPTN	-0.921769106	0.023487605
NPTX1	-1.878528637	0.013999648
NPTX2	-0.674236404	0.223181704
NPTXR	-1.396699349	0.01574763
NPVF	-0.15136732	0.15015411
NPY	-0.235304572	0.54721235
NPY1R	-1.18666201	0.026485864
NPY2R	-0.273160621	0.279849035
NPY4R	-0.064146193	0.674740646
NPY5R	-0.37887595	0.049583192
NQO1	-1.016597353	0.065789373
NQO2	-0.156667964	0.665904933
NR0B1	0.105249448	0.645552779
NR0B2	-0.025728133	0.826584593
NR1D1	-0.548398711	0.389435147
NR1D2	-0.368701432	0.149711207
NR1H2	-0.48838547	0.059425494
NR1H3	0.276329785	0.194654605
NR1H4	-0.05394408	0.510790042
NR1I2	-0.018568536	0.911492452
NR1I3	-0.168347847	0.216682538
NR2C1	0.268556042	0.06223633
NR2C2	-0.040218405	0.854655022

NR2C2AP	0.275872628	0.178134407
NR2E1	-0.67021348	0.177946788
NR2E3	-0.120721677	0.350941185
NR2F1	0.515254538	0.030426619
NR2F6	0.019436292	0.95912527
NR3C2	-0.306836921	0.512915077
NR4A1	-0.251397667	0.582804387
NR4A2	0.223676345	0.693064053
NR4A3	0.40613788	0.413149269
NR5A1	0.014363944	0.910283756
NR5A2	0.362500844	0.037714032
NR6A1	0.400574069	0.067031014
NRAP	-0.067974954	0.589570549
NRARP	0.559642115	0.039866043
NRAS	0.430534446	0.251726604
NRBF2	-0.154173294	0.53810105
NRBP1	0.303979327	0.093194553
NRBP2	-0.161641625	0.659275683
NRCAM	0.909510893	0.178134407
NRDC	0.203518472	0.130638342
NRDE2	0.18180542	0.229609917
NRG1	-0.769076702	0.015880694
NRG2	0.179943443	0.300293113
NRG4	-0.590840207	0.004334512
NRIP1	0.363538533	0.124368264
NRIP2	-0.214963586	0.166190394
NRIP3	-0.909063864	0.012301856
NRK	-0.404490804	0.098998399
NRL	0.028206344	0.860002363
NRN1	-0.071592327	0.927976981
NRN1L	-0.103674478	0.787602992
NRP1	0.840919449	0.084089735
NRP2	0.593927544	0.30453461
NRROS	0.061543357	0.752870608
NRSN1	-1.035687698	0.045172498
NRSN2	-0.629870885	0.04367646
NRTN	0.021649432	0.921612349
NRXN2	-0.17566818	0.804383366
NRXN3	-1.996594918	0.004996766
NSA2	0.024058901	0.918053575
NSD1	0.45925243	0.019064046
NSD2	0.502720614	0.022236915
NSD3	0.237697567	0.277672163
NSFL1C	-0.045134596	0.779159502
NSG1	-0.63390942	0.010529028
NSG2	-0.343487815	0.116075189
NSL1	0.280340901	0.20573308
NSMAF	0.569760303	0.00041574

NSMCE1	0.147727741	0.678115572
NSMCE2	0.307615121	0.06009886
NSMCE3	0.051650116	0.779783251
NSMCE4A	0.144926505	0.476374594
NSRP1	0.57378284	0.003624066
NSUN2	0.562296081	0.006729601
NSUN3	0.190896133	0.117781718
NSUN4	-0.65330506	0.008524053
NSUN5	0.625956019	0.035192281
NSUN6	0.386294179	0.232358675
NSUN7	0.773384124	0.011064725
NT5C1A	-0.425666404	0.010335286
NT5C2	-0.355302367	0.105478834
NT5DC1	-1.32773968	0.000269441
NT5DC2	0.246299505	0.631104635
NT5E	0.468425931	0.526031456
NT5M	-0.2006717	0.329369557
NTAN1	-0.100817941	0.773333319
NTF4	0.018705853	0.939257006
NTHL1	-0.056456885	0.679418222
NTM	-0.569768674	0.027465518
NTMT1	-0.010860645	0.952685664
NTN1	1.163608708	0.002267206
NTN3	0.191793167	0.352886599
NTN4	-1.127295747	0.050885901
NTN5	-0.131974155	0.25585945
NTNG1	-0.645804881	0.12343853
NTNG2	-0.782051828	0.007465195
NTPCR	0.028307815	0.876958562
NTRK1	0.045849515	0.781253308
NTRK2	-0.617251276	0.582904019
NTS	0.063340563	0.729015453
NTSR1	0.021618055	0.908823411
NTSR2	-2.000962389	0.002071678
NUAK1	-1.930342746	0.000126605
NUAK2	0.045623751	0.94493417
NUBP1	0.508643174	0.035777524
NUBP2	-0.200090897	0.348945443
NUCB1	-0.158686309	0.428740734
NUCB2	-0.035596394	0.869597022
NUCKS1	0.360369615	0.1595089
NUDC	-0.172352385	0.202529193
NUDCD1	0.305504543	0.104517426
NUDCD2	0.23406913	0.083262704
NUDCD3	0.308076551	0.156456644
NUDT1	0.449815587	0.052310127
NUDT10	0.161165517	0.403911084
NUDT11	0.43357691	0.345154663

NUDT12	0.223405013	0.243747753
NUDT13	0.201000879	0.138575486
NUDT14	-0.449064215	0.043019254
NUDT15	-0.133818268	0.604126128
NUDT16	-0.349560883	0.196209899
NUDT16L1	0.095821773	0.563322948
NUDT17	0.117397471	0.536762845
NUDT18	-0.157789523	0.210412136
NUDT2	-0.181992871	0.342766618
NUDT21	0.121352036	0.532979116
NUDT22	0.182820018	0.307586975
NUDT4	0.227067702	0.065878376
NUDT5	0.422832489	0.169850016
NUDT6	0.243544182	0.096296467
NUDT8	-0.213457705	0.173820085
NUDT9	0.251086377	0.060259419
NUF2	0.905329543	0.04776679
NUFIP1	0.171029277	0.425370558
NUFIP2	0.538760634	0.005678092
NUMA1	0.187331554	0.368362316
NUMBL	0.053296466	0.90145729
NUP107	0.771843836	0.008428763
NUP133	0.171152328	0.373969966
NUP153	0.432951192	0.012142567
NUP155	0.542130369	0.026485864
NUP210	0.854060158	0.011982868
NUP210L	-0.075066482	0.525948523
NUP214	0.701396095	0.00183367
NUP35	0.478078157	0.013430848
NUP37	0.525302186	0.016899346
NUP42	0.55160201	0.023029329
NUP43	0.848384558	0.007315104
NUP50	0.203911554	0.168038754
NUP54	0.304443116	0.187177307
NUP58	0.505552858	0.005081616
NUP62CL	0.066414122	0.695473068
NUP85	0.894680288	0.003140901
NUP88	0.632245261	0.033762116
NUP93	-0.177579761	0.350518891
NUP98	0.406676886	0.046335904
NUS1	0.408349205	0.06587296
NUSAP1	1.582707033	0.011372074
NUTF2	-0.477925764	0.087441348
NUTM1	-0.081381373	0.484357668
NUTM2D	-0.045094436	0.85557224
NUTM2G	0.034900084	0.876652194
NVL	0.760310673	0.005081616
NWD2	-0.981626164	0.00400499

NXF3	-0.041108775	0.775684815
NXNL1	-0.229088574	0.127380124
NXNL2	0.092189242	0.566904258
NXPE1	-0.08735672	0.425384977
NXPE2	0.056768008	0.764477133
NXPH1	0.194200487	0.714693885
NXPH2	-0.183438327	0.095422899
NXPH3	-0.160688741	0.471442677
NXPH4	0.020290374	0.956242346
NXT1	0.167476966	0.346647327
NXT2	0.261111307	0.12071573
NYAP1	-0.314767164	0.113877532
NYAP2	-1.018725433	0.023851097
NYX	0.068583714	0.535196549
OAF	-0.703498669	0.00839662
OARD1	1.151850901	0.002827106
OAS1	1.505156699	0.000879799
OAS2	1.110509212	0.007779294
OAS3	1.011868403	0.003602867
OASL	0.132221441	0.516248742
OAT	-0.621271821	0.010254353
OAZ1	-0.529451535	0.012280746
OAZ2	-0.101520253	0.609626099
OBP2A	-0.429901434	0.047541162
OBP2B	-0.285445213	0.208984508
OCA2	-0.7607991	0.003709792
OCEL1	-0.317008557	0.105121233
OCIAD1	-0.081700414	0.520204191
OCIAD2	-0.19062913	0.669302517
OCM2	-0.107113476	0.416515509
OCRL	-0.16460868	0.418746359
ODAM	-0.110870522	0.222134524
ODC1	0.860580504	0.08230465
ODF1	-0.116000696	0.359143728
ODF3	-0.017637266	0.911150229
ODF3L1	-0.039275731	0.712803392
ODF3L2	0.033280812	0.845275235
ODF4	-0.192091035	0.120199333
ODR4	0.357382969	0.057492253
OFD1	1.001338556	0.000164034
OGA	-0.451417279	0.038350121
OGDH	-0.153437469	0.375141671
OGDHL	-1.6174616	0.002068027
OGFOD1	0.098659308	0.785869731
OGFOD2	0.198516098	0.165367965
OGFR	0.222517973	0.478877114
OGFRL1	0.054546039	0.913868035
OGG1	0.343174894	0.034224831

OGN	0.114567835	0.839402086
OGT	0.305678439	0.13284779
OIP5	0.142572427	0.50452383
OIT3	0.063072573	0.506781957
OLA1	-0.121548295	0.592465991
OLAH	-0.343501597	0.162701983
OLFM1	-1.129552679	0.046770639
OLFM2	-0.18265791	0.765319466
OLFM4	-0.172929297	0.039682292
OLFML1	0.122740997	0.685402367
OLFML2A	0.867100078	0.186501165
OLFML2B	1.330846589	0.041285832
OLFML3	1.170001459	0.010003082
OLIG1	0.56421034	0.478877114
OLIG2	1.032118865	0.197576177
OLIG3	-0.129955562	0.325077423
OLR1	0.753859944	0.246939699
OMA1	0.269602119	0.340627648
OMD	-1.002290851	0.020063686
OMG	-1.228519093	0.152998579
OMP	0.173234518	0.369866211
ONECUT1	0.309143252	0.107507557
ONECUT2	0.037768105	0.839163948
ONECUT3	-0.065472517	0.646684785
OOSP2	-0.211745248	0.024278628
OOSP4B	-0.055593433	0.563322948
OPA1	-0.261607096	0.181766132
OPA3	0.021052332	0.932102269
OPCML	-1.328889276	0.049610879
OPHN1	-0.06343246	0.915607882
OPLAH	-0.020483431	0.915446555
OPN1LW	0.282514623	0.101783522
OPN1MW	-0.027246623	0.871201192
OPN1SW	-0.05458231	0.707562098
OPN3	-0.47147421	0.279171824
OPRD1	-0.054861006	0.793511017
OPRK1	-0.301394215	0.096642913
OPRPN	-0.023707702	0.76702076
OPTC	0.03581523	0.840425315
OR10A2	-0.251402315	0.114994599
OR10A3	-0.213480076	0.059593665
OR10A4	-0.04641578	0.700015813
OR10AD1	0.018893845	0.920850924
OR10AG1	0.071785769	0.461017246
OR10D3	-0.210012964	0.438285007
OR10G2	-0.195259606	0.090451248
OR10G4	-0.202916264	0.213829425
OR10G7	-0.02536652	0.918837703

OR10G8	0.321472298	0.053470148
OR10G9	0.121159755	0.521854861
OR10H1	-0.097000881	0.251511329
OR10H2	0.119011884	0.489388373
OR10H3	-0.027635252	0.901235662
OR10H4	-0.401021272	0.037605529
OR10H5	-0.118267363	0.252482619
OR10J1	-0.262374491	0.008235508
OR10J5	0.297018132	0.074642043
OR10K1	-0.046635525	0.654152393
OR10Q1	-0.097291621	0.506700199
OR10R2	-0.118820207	0.459874564
OR10S1	-0.037713879	0.843863404
OR10T2	-0.060658142	0.685111699
OR10V1	-0.158605889	0.091598211
OR10W1	-0.123238132	0.116075189
OR10X1	-0.07688814	0.6062359
OR10Z1	-0.171012481	0.114153909
OR11A1	-0.055257307	0.643013996
OR11G2	-0.139249565	0.146945011
OR11H2	-0.054102522	0.64590421
OR11H4	-0.018573773	0.835493839
OR11H6	-0.072308463	0.371269109
OR11L1	-0.124015371	0.228009818
OR12D2	-0.116864654	0.159828897
OR12D3	-0.184224498	0.051261644
OR13A1	-0.255119064	0.050183209
OR13C2	-0.119172487	0.199371809
OR13C3	-0.139608205	0.159732415
OR13C5	0.012522788	0.91752091
OR13C8	-0.017057272	0.911273041
OR13C9	-0.203852021	0.078368292
OR13D1	-0.169050772	0.234572666
OR13F1	-0.099604218	0.153046427
OR13G1	-0.199163528	0.027032356
OR13H1	-0.044248603	0.650417176
OR13J1	-0.122242633	0.414997627
OR14C36	-0.029130792	0.843863404
OR14I1	-1.291856195	0.038044824
OR14K1	-0.064171479	0.487533227
OR1A1	0.093071372	0.516380268
OR1A2	-0.027604021	0.879784058
OR1C1	-0.089310933	0.395192157
OR1D2	-0.144377939	0.187177307
OR1E1	-0.182489867	0.084919791
OR1E2	-0.068307417	0.800675224
OR1F1	-0.09697325	0.620741982
OR1G1	-0.038596207	0.756811437

OR1I1	-0.135425575	0.243804426
OR1J1	-0.048179439	0.703745425
OR1J2	0.16283261	0.368464473
OR1J4	-0.186828799	0.111027902
OR1K1	-0.089647645	0.368538531
OR1L1	-0.104108973	0.316898088
OR1L3	0.002759323	0.981263887
OR1L6	-0.162578339	0.052529273
OR1L8	-0.085152394	0.535001127
OR1N1	-0.070746647	0.62413692
OR1N2	0.03352565	0.760420863
OR1Q1	-0.152649522	0.074346764
OR1S1	-0.006401208	0.971953247
OR1S2	0.125100901	0.393103618
OR2A12	0.085598814	0.358491591
OR2A14	-0.078544586	0.586733255
OR2A2	-0.040007201	0.81109553
OR2A25	0.038461024	0.734048692
OR2A4	-0.084567346	0.432216082
OR2A42	0.152248013	0.530178547
OR2A5	-0.187096187	0.183642284
OR2AE1	0.145774943	0.345154663
OR2AG1	-0.020123047	0.896420697
OR2AG2	-0.128394865	0.181118964
OR2AJ1	-0.1873552	0.19124811
OR2AK2	-0.54875784	0.21860477
OR2AT4	-0.064008327	0.600309691
OR2B11	-0.188329579	0.21010167
OR2B2	-0.053184694	0.576779548
OR2B3	-0.136285044	0.093334006
OR2C1	0.542445393	0.006185744
OR2C3	-0.034935438	0.727460808
OR2D2	-0.060260471	0.725355865
OR2D3	-0.124441567	0.12573363
OR2F1	0.163016263	0.160253514
OR2F2	-0.302044414	0.01944497
OR2G3	-0.05724753	0.710688939
OR2G6	-0.046353603	0.798918737
OR2H2	-0.107763829	0.438504687
OR2J1	-0.023909461	0.805642311
OR2J2	0.238329251	0.276108367
OR2J3	-0.116154899	0.365990547
OR2K2	-0.113068898	0.477276276
OR2L2	-0.37875974	0.257269688
OR2L3	0.02143994	0.931727556
OR2M2	0.05386409	0.726774471
OR2M3	-0.512058938	0.014338374
OR2M5	-0.560354941	0.006580315

OR2M7	-0.283437033	0.052779362
OR2S2	0.048834742	0.769889235
OR2T10	-0.235085441	0.133087063
OR2T12	0.150832501	0.674472706
OR2T2	0.535706788	0.042339582
OR2T27	-0.227715819	0.138969461
OR2T29	0.029372175	0.795726924
OR2T3	-0.339804263	0.06168001
OR2T33	-0.050223197	0.521703775
OR2T34	-0.153734209	0.254522401
OR2T4	-0.088301119	0.549355204
OR2T5	-0.07139337	0.488979219
OR2V2	0.071802311	0.671669419
OR2Y1	-0.038778814	0.731977162
OR2Z1	-0.291109574	0.06041
OR3A1	-0.137601453	0.401578625
OR3A2	-0.108318016	0.433740858
OR3A3	0.088631736	0.648779833
OR4A16	-0.006156992	0.952200432
OR4A47	-0.103847954	0.17266309
OR4C11	-0.064979372	0.518566194
OR4C16	-0.157382536	0.222477877
OR4C3	-0.145117006	0.069184909
OR4C46	-0.020827637	0.896915217
OR4C5	-0.09493578	0.459797666
OR4C6	-0.020898993	0.892641383
OR4D1	-0.077299024	0.324924741
OR4D10	-0.140168631	0.376151151
OR4D11	-0.108171294	0.586721542
OR4D2	-0.022376462	0.858782911
OR4D5	-0.135136542	0.291933969
OR4D6	-0.258456328	0.016753896
OR4D9	-0.135836561	0.228531369
OR4F21	-0.173979407	0.112548547
OR4F29	-0.050025902	0.712803392
OR4F4	-0.123399277	0.3184098
OR4K14	0.075127635	0.617468085
OR4K15	-0.137284751	0.149621879
OR4K17	0.026772134	0.7879778
OR4K2	-0.019392432	0.911631292
OR4K5	-0.060113077	0.538529424
OR4L1	-0.140980355	0.101437619
OR4M1	0.100747613	0.401740484
OR4N2	-0.167561471	0.396240729
OR4N4	0.135124979	0.538250904
OR4N5	0.048135483	0.780791174
OR4P4	-0.105752629	0.239115209
OR4S1	-0.015673793	0.881269954

OR4X1	-0.096049053	0.57259631
OR51A2	-0.100667853	0.669970192
OR51A4	-0.099835295	0.468100941
OR51A7	-0.152677807	0.196976608
OR51B2	-0.267041083	0.041553053
OR51B4	-0.048026613	0.744402699
OR51B6	-0.332387054	0.02900795
OR51D1	-0.042991108	0.713113471
OR51E1	0.302130003	0.078652069
OR51E2	0.037686074	0.821868934
OR51F1	-0.11137193	0.345619607
OR51F2	-0.067506001	0.448614769
OR51G2	-0.145993159	0.250512665
OR51I2	0.002156281	0.986045302
OR51L1	-0.086414411	0.252482619
OR51M1	-0.046176198	0.68290411
OR51Q1	-0.044679942	0.623458448
OR51S1	-0.377681159	0.038207018
OR51T1	-0.050805644	0.704853688
OR51V1	-0.021077338	0.914032449
OR52A1	-0.104194834	0.248606764
OR52A5	-0.045993869	0.736206294
OR52B2	-0.130520237	0.167782611
OR52B4	-0.102893401	0.21047169
OR52B6	-0.218873925	0.146973713
OR52D1	0.01456816	0.902639273
OR52E2	-0.012882617	0.932999857
OR52E4	-0.102208064	0.348322976
OR52E6	-0.061714907	0.635554181
OR52E8	-0.251076633	0.162701983
OR52H1	0.062566745	0.621217076
OR52I1	-0.07975675	0.520756654
OR52I2	-0.169089479	0.026338533
OR52J3	0.057120895	0.703788367
OR52K1	-0.052433828	0.721049202
OR52K2	0.073976302	0.678115572
OR52L1	0.100408775	0.326932275
OR52M1	0.126990749	0.21891269
OR52N1	-0.184543875	0.115181658
OR52N2	0.102442835	0.508646271
OR52N4	-0.076283621	0.436059122
OR52N5	-0.040431411	0.841759022
OR52W1	0.049098223	0.731357875
OR56A3	0.032482782	0.78555418
OR56A4	-0.011869586	0.91733085
OR56B1	-0.103503701	0.351918897
OR56B4	-0.000608493	0.996803176
OR5A1	-0.202443914	0.042178398

OR5A2	0.025122749	0.91915956
OR5AK2	-0.070665257	0.5208979
OR5AN1	-0.03537381	0.781418662
OR5AP2	-0.135150642	0.288325171
OR5AS1	0.008993315	0.932814988
OR5AU1	-0.052937739	0.641952156
OR5B12	-0.125848038	0.145754821
OR5D13	-0.159861433	0.081096724
OR5D14	-0.288010936	0.029094312
OR5D18	0.007314084	0.948055091
OR5H14	-0.136095838	0.223374357
OR5H15	-0.058934498	0.679559867
OR5I1	-0.019191277	0.927797131
OR5K1	-0.059022558	0.507728992
OR5K2	-0.006342464	0.960903847
OR5L2	0.041677492	0.861402957
OR5M1	0.290127851	0.09259476
OR5M10	-0.089732036	0.412521054
OR5M11	0.009992916	0.936643326
OR5M3	0.00512711	0.968725515
OR5M8	-0.141437045	0.102956472
OR5M9	0.014889619	0.90466277
OR5P2	-0.242596383	0.135123268
OR5P3	-0.111772935	0.306404394
OR5T2	-0.133672239	0.172795607
OR5T3	-0.102514957	0.339356809
OR6A2	-0.187964923	0.160250505
OR6B1	-0.003636841	0.981633356
OR6B2	-0.194557062	0.081898324
OR6C1	-0.059907819	0.43381273
OR6C3	-0.255849844	0.058132615
OR6C4	-0.361249495	0.077074221
OR6C65	-0.100699962	0.356472461
OR6C74	-0.029161971	0.789854304
OR6C76	-0.184581617	0.233586232
OR6F1	0.019744582	0.853715871
OR6J1	-0.012147933	0.93451723
OR6K2	0.019689484	0.849142525
OR6K3	-0.101313085	0.303115542
OR6K6	-0.149524321	0.117715258
OR6M1	-0.064170522	0.552503918
OR6N1	-0.140667888	0.336976049
OR6Q1	-0.091639234	0.603899327
OR6S1	0.083650737	0.556897693
OR6T1	-0.030334054	0.821001661
OR6V1	-0.106317663	0.619223958
OR6X1	-0.017199316	0.853715871
OR6Y1	-0.196926691	0.094029063

OR7A10	-0.124325619	0.478963272
OR7A17	-0.113543633	0.369309195
OR7A5	-0.349308684	0.015797802
OR7C1	0.301484683	0.161281614
OR7C2	-0.111990991	0.335797568
OR7D4	-0.099440589	0.593944428
OR7E24	-0.042894628	0.668794145
OR7G1	-0.162266033	0.281300157
OR7G2	-0.014361356	0.941168506
OR7G3	-0.161636916	0.153904658
OR8A1	-0.042620751	0.752594737
OR8B12	-0.061377847	0.709670769
OR8B3	-0.201575844	0.054772419
OR8B4	-0.127794339	0.243223519
OR8B8	-0.275863547	0.263197659
OR8D1	-0.131992152	0.127380124
OR8D2	-0.106666028	0.392266258
OR8D4	-0.085895438	0.27643327
OR8G1	-0.258198377	0.01673506
OR8H1	-0.052423055	0.615384214
OR8H2	-0.223079144	0.086855832
OR8H3	-0.578936317	0.001776331
OR8I2	-0.068835733	0.645011955
OR8K1	-0.08329005	0.3184098
OR8K3	-0.087908709	0.535132197
OR8K5	-0.123784912	0.313216887
OR8S1	-0.124588132	0.30041866
OR8U1	0.012490746	0.894416316
OR9A2	-0.377365898	0.159585663
OR9A4	0.10485004	0.350979442
OR9G1	-0.209107171	0.047896467
OR9K2	-0.053724639	0.548610755
ORAI3	0.217917758	0.455826784
ORC1	0.40076898	0.160853052
ORC3	0.057757271	0.754493224
ORC5	0.056203657	0.733454552
ORC6	0.290783731	0.350077294
ORM1	-0.058301528	0.518566194
ORMDL1	0.579324411	0.001341398
ORMDL2	0.194458598	0.5562846
ORMDL3	0.251661282	0.324497184
OS9	0.413220534	0.03601003
OSBP	0.047739788	0.7240164
OSBP2	-0.652967574	0.019973008
OSBPL10	0.197107191	0.459765932
OSBPL11	0.460332538	0.132445969
OSBPL2	-0.280490658	0.073592779
OSBPL3	1.214793868	0.011477141

OSBPL5	0.043129105	0.86984044
OSBPL7	0.423458062	0.067269183
OSBPL8	-0.078292745	0.821763617
OSBPL9	0.273765586	0.144197979
OSCAR	0.176006945	0.28224197
OSCP1	0.442980641	0.073454421
OSER1	0.676942197	0.001038868
OSGEP	0.451508093	0.024933462
OSGEPL1	0.220617304	0.182772588
OSGIN1	0.079835787	0.719619039
OSM	0.236000629	0.28630244
OSMR	1.285101887	0.084079729
OSR1	0.047168738	0.927976981
OSR2	0.139811528	0.490425951
OSTC	0.750436857	0.137352678
OSTF1	-0.557452967	0.133082976
OSTM1	0.3151558	0.190867027
OSTN	-0.123719978	0.168506214
OTC	-0.086149084	0.314545334
OTOA	-0.020917284	0.904151702
OTOF	-0.13751935	0.168681476
OTOG	0.033861426	0.764477133
OTOGL	-0.329975442	0.173410801
OTOP1	-0.213467878	0.080680741
OTOP2	-0.000492995	0.997942116
OTOP3	0.012398053	0.911631292
OTOR	0.016301682	0.870665906
OTOS	-0.159341512	0.377625058
OTP	-0.060123211	0.639207056
OTUB1	-0.561658391	0.004565843
OTUB2	-0.275365487	0.123801057
OTUD5	-0.156097481	0.37018066
OTUD6A	-0.186845627	0.196976608
OTUD6B	0.511549657	0.001526804
OTUD7A	-1.07924163	0.005746243
OTUD7B	0.043331585	0.874708742
OTULIN	0.603593152	0.00064817
OTULINL	0.524695341	0.091166577
OTX2	-0.177005235	0.062381284
OVCA2	0.143140491	0.564293285
OVCH1	-0.12887535	0.343725271
OVOL1	0.098302519	0.479498653
OVOL2	-0.067080343	0.766469255
OVOS2	-0.015599588	0.956825161
OXCT1	-0.619242922	0.160922732
OXER1	0.03111836	0.853363564
OXGR1	-0.172372901	0.172744121
OXNAD1	0.155402039	0.418573863

OXSM	0.150908408	0.501120842
OXSR1	-0.101079262	0.706057776
OXT	-0.255800383	0.34866536
P2RX1	-0.056548252	0.665904933
P2RX2	-0.089918528	0.488387975
P2RX3	-0.038296483	0.773333319
P2RX6	0.004763458	0.986498278
P2RY1	1.068278187	0.063175636
P2RY10	-0.042988711	0.701881584
P2RY12	-0.082293823	0.938370209
P2RY13	0.355342706	0.512007635
P2RY2	-0.003908381	0.98286235
P2RY4	0.114746023	0.375141671
P2RY6	0.029649292	0.855304945
P2RY8	-0.12033016	0.703601492
P3H2	-0.027255116	0.965711172
P3H4	0.002918932	0.993945285
P4HA1	0.624550382	0.251313843
P4HA2	0.027629721	0.941459946
P4HTM	0.159004681	0.639207144
PA2G4	0.125096894	0.543937856
PAAF1	0.233130602	0.242859456
PABPC1	0.651640568	0.026941797
PABPC1L2A	-0.591686006	0.026567115
PABPC3	-0.108258517	0.418146701
PABPC5	0.235471252	0.504202244
PACRG	0.432955564	0.091518365
PACRGL	0.452343617	0.042906172
PACS1	-0.261668599	0.033803615
PACSIN2	0.116769253	0.55712095
PADI1	-0.103936583	0.304645963
PADI2	-0.579997675	0.473735539
PADI3	-0.008024618	0.954575083
PADI4	-0.038859773	0.723856561
PADI6	-0.077185467	0.553524494
PAEP	-0.064430205	0.59150484
PAF1	0.208840662	0.131106757
PAFAH1B1	-0.389821806	0.057833697
PAFAH1B3	0.018196472	0.953362739
PAFAH2	0.460564009	0.010491654
PAG1	1.586055856	0.00010541
PAGE1	0.078350307	0.681406422
PAGE2	0.095176925	0.567137468
PAGE4	0.046516111	0.647225297
PAGE5	-0.22417743	0.373254452
PAGR1	0.525496307	0.350428516
PAH	-0.293574361	0.020347649
PAIP1	-0.029726257	0.878160468

PAIP2	-0.288946503	0.062489218
PAK1IP1	0.240552327	0.455635494
PAK2	0.313461852	0.350518891
PAK3	-2.014902038	0.004545328
PAK5	-1.435105695	0.032576429
PALB2	0.323334885	0.037997415
PALD1	0.92732679	0.006445148
PALM	-1.571396672	0.000739219
PALMD	0.345569613	0.385767435
PAM	0.270179094	0.530361631
PAN2	1.066616133	0.00010541
PANK1	-0.15935139	0.401711068
PANK3	-0.205956113	0.458993187
PANK4	0.101528918	0.602663244
PANX1	0.13589556	0.736206294
PANX3	-0.122522062	0.331088818
PAOX	0.257926884	0.252127612
PAPLN	0.484720217	0.238371212
PAPOLA	0.47000641	0.042885162
PAPOLB	0.446591324	0.059715312
PAPOLG	-0.003291977	0.989859482
PAPPA2	0.223940741	0.48497136
PAPSS1	-0.069682314	0.713485047
PAPSS2	-0.162613885	0.751177651
PAQR3	0.047275765	0.843863404
PAQR4	-0.267883447	0.512709781
PAQR5	-0.364428902	0.078344901
PAQR6	-0.442429713	0.477248291
PAQR7	-0.086079061	0.665904933
PAQR8	-0.237736936	0.589701197
PAQR9	-0.143833435	0.340627648
PARD3	0.033902708	0.907973851
PARD3B	0.675405796	0.02629232
PARD6A	-0.367536799	0.19297093
PARD6G	0.050918787	0.791876479
PARG	-0.008269839	0.978932565
PARK7	0.197859237	0.324270264
PARL	0.290673634	0.094473358
PARM1	-1.166478996	0.185411978
PARN	0.386871943	0.040781945
PARP1	0.222030697	0.203901234
PARP10	0.403050005	0.034988055
PARP11	0.720522093	0.008396018
PARP12	1.086986533	0.004563267
PARP14	2.447826295	4.99E-05
PARP16	0.177529733	0.334308509
PARP2	0.088204225	0.770931382
PARP3	0.410782253	0.268371276

PARP4	0.806645658	0.01946524
PARP6	0.769635936	0.007879685
PARPBP	0.441907807	0.063950041
PARS2	0.182255255	0.37348365
PARVA	-0.353554039	0.371744841
PARVB	0.084762326	0.631524871
PATE1	-0.173347516	0.040927029
PATE2	-0.052384705	0.724307904
PATJ	0.136026242	0.445981785
PATL1	0.675801772	0.000689186
PATZ1	0.232423459	0.41174686
PAWR	-0.395688447	0.393000761
PAX1	-0.04937978	0.630540343
PAX2	-0.010359296	0.944819609
PAX3	0.493386133	0.235415993
PAX4	0.067404897	0.612041862
PAX5	-0.027021126	0.856424995
PAX7	-0.110124888	0.389722288
PAX8	-0.251519499	0.316598965
PAX9	-0.105079017	0.211996041
PAXBP1	1.520483367	7.88E-05
PAXIP1	0.982033183	0.00044063
PAXX	0.282614107	0.277225602
PBDC1	0.843020543	0.007845807
PBK	1.359737829	0.030943115
PBLD	-0.249879868	0.164919812
PBOV1	-0.083396907	0.300029437
PBRM1	0.596010925	0.015103305
PBX1	0.15494603	0.699160214
PBX3	0.5596814	0.074609415
PBX4	0.101333612	0.44673969
PBXIP1	0.123807012	0.695139082
PCARE	0.018738124	0.886463258
PCBD1	-0.537448768	0.036915423
PCBD2	0.945347786	0.007396766
PCBP1	-0.150982047	0.529217606
PCBP3	-0.131023163	0.699013138
PCBP4	-0.397322496	0.216569464
PCCA	-0.127047547	0.71174887
PCCB	-0.138284355	0.453656837
PCDH1	-0.187305038	0.646942576
PCDH10	-0.762045469	0.08782594
PCDH11X	-0.419793962	0.46525882
PCDH11Y	-0.514469081	0.259482856
PCDH12	0.3629722	0.060011766
PCDH15	0.53123687	0.591009056
PCDH18	1.692738207	0.001249683
PCDH7	-1.35797113	0.042998566

PCDH8	-1.276514345	0.011984713
PCDH9	-0.736872544	0.379563994
PCDHA10	0.162949679	0.409654189
PCDHB10	2.35791317	4.88E-05
PCDHB11	1.235144321	0.000367982
PCDHB12	2.249699586	7.88E-05
PCDHB13	1.625728981	0.001746286
PCDHB14	1.810383237	0.000888995
PCDHB16	1.705342691	0.000511553
PCDHB2	1.295332889	0.014785217
PCDHB3	1.814752299	0.000543359
PCDHB4	0.737773414	0.152580703
PCDHB5	1.192409358	0.059715312
PCDHB7	1.922014551	0.001117596
PCDHB8	1.90950655	0.001454069
PCDHB9	1.952810555	0.000173709
PCDHGA10	0.93317593	0.005492418
PCED1A	0.646955502	0.028452842
PCED1B	0.26995776	0.369589911
PCF11	0.498700915	0.017056623
PCGF1	0.353216266	0.012140035
PCGF2	0.244424568	0.380167689
PCGF3	0.460448949	0.043461433
PCID2	0.888368472	0.012621848
PCIF1	0.056999063	0.831972223
PCK1	-0.136431405	0.152324857
PCK2	-0.048529959	0.764899333
PCLAF	0.501026383	0.037049711
PCM1	0.350518247	0.15119423
PCMT1	-0.400460568	0.096350469
PCMTD1	0.096265926	0.654458918
PCMTD2	-0.025749563	0.938914308
PCNA	0.925755328	0.046902664
PCNP	-0.016866397	0.92516359
PCNT	0.166017176	0.634228974
PCNX2	-0.150138548	0.670750072
PCNX3	0.392786346	0.032576429
PCNX4	0.574865744	0.009571203
PCOLCE	1.096107537	0.04295608
PCOLCE2	0.176640823	0.646560301
PCP4	-0.211531916	0.340381872
PCSK1	-0.727331447	0.301559457
PCSK1N	-1.206969809	0.021984143
PCSK2	-1.591075833	0.076256414
PCSK4	-0.11809305	0.427259491
PCSK6	-0.05091904	0.828914871
PCSK7	0.540239624	0.009612941
PCSK9	-0.082406323	0.58596993

PCYOX1	-0.426069104	0.032618429
PCYOX1L	0.187939499	0.155522477
PCYT1A	0.068072217	0.700479507
PCYT1B	-0.344153923	0.494972086
PDAP1	0.492825677	0.047720399
PDC	-0.197370962	0.108471441
PDCD1	-0.043805306	0.746533995
PDCD10	0.025928463	0.870363001
PDCD1LG2	0.294277254	0.677199137
PDCD2	0.443384523	0.05796386
PDCD4	-0.284005727	0.448441679
PDCD5	0.345923997	0.057823762
PDCD6IP	0.212402447	0.291211053
PDCD7	-0.169209941	0.322585459
PDCL	0.374568791	0.074033075
PDCL2	-0.042432909	0.700015813
PDCL3	-0.47381544	0.042219069
PDE11A	-0.278238157	0.015608607
PDE12	-0.002759712	0.993549673
PDE1B	-1.35421307	0.019659172
PDE3A	0.835894061	0.121673155
PDE3B	-0.42843944	0.252826729
PDE5A	0.027606041	0.96051513
PDE6A	0.001870655	0.990288492
PDE6B	-0.68901976	0.073415686
PDE6C	-0.040173368	0.65000111
PDE6D	-0.41465543	0.043368392
PDE6G	0.046259435	0.749203661
PDE6H	-0.059631647	0.57428374
PDE7A	0.396647663	0.111086676
PDE7B	-0.539719693	0.257142059
PDE8A	-0.074179265	0.845746964
PDE8B	0.214717394	0.764424424
PDE9A	1.236011627	0.001838748
PDGFA	0.396611512	0.34209717
PDGFB	0.089672103	0.808408648
PDGFD	0.723314152	0.149630608
PDGFRA	1.070422952	0.279918882
PDGFRB	0.309114752	0.596951562
PDGFRL	0.507887088	0.243747753
PDHA2	0.041651062	0.674472706
PDHB	-0.251550502	0.158848943
PDHX	-0.369517944	0.036372166
PDIA3	0.347334784	0.052762388
PDIA4	1.424579929	0.003568303
PDIA5	0.494292109	0.431369548
PDIA6	0.56451155	0.058070792
PDILT	-0.017927276	0.882434721

PDK1	0.305668071	0.512415786
PDK2	-0.260232984	0.132445969
PDK4	-0.460121693	0.543983376
PDLIM1	0.12815981	0.883613174
PDLIM3	1.135718402	0.037871129
PDLIM4	0.502362584	0.391636567
PDLIM7	0.165997761	0.810929114
PDP2	-0.051310679	0.831972223
PDPK1	-0.471148508	0.028360435
PDPR	0.676072075	0.02215249
PDRG1	0.022782034	0.884920673
PDS5B	-0.328305951	0.302467936
PDSS1	-0.021522762	0.917640056
PDSS2	0.207260542	0.385784865
PDX1	-0.295926829	0.058757095
PDXDC1	0.263252498	0.388701508
PDXK	-0.345818107	0.196029149
PDZD11	-0.10224911	0.527496385
PDZD2	0.084580607	0.91752091
PDZD4	-1.356324825	0.006589628
PDZD8	-0.529727191	0.02215249
PDZK1IP1	0.164067862	0.177287889
PEA15	-0.471614841	0.034284429
PEAK1	0.300878132	0.157113007
PEAK3	0.152927464	0.39408223
PEAR1	-0.064959205	0.853507252
PEBP1	-0.826613265	0.011141587
PECR	0.158123007	0.429982193
PELI1	1.163619071	0.006580315
PELI2	0.276053036	0.259568649
PELI3	-0.629451149	0.006881353
PEMT	-0.136244838	0.602524331
PEPD	3.57E-05	0.999891414
PER1	0.527594124	0.226576654
PER2	0.036245547	0.931287352
PER3	-0.839988417	0.030804706
PERP	-0.340912437	0.281752955
PEX1	0.546171934	0.009312966
PEX10	0.21730779	0.06799859
PEX11A	0.023199264	0.915607882
PEX11G	0.249875487	0.145193906
PEX12	0.212715162	0.460686163
PEX13	0.161896054	0.207437993
PEX14	-0.093882029	0.490985728
PEX16	-0.187287826	0.193589679
PEX19	-0.105582684	0.490024874
PEX3	-0.189044283	0.297935458
PEX5L	-2.940969173	0.000375895

PEX6	0.325553641	0.115242277
PEX7	0.088174082	0.647135908
PF4	-0.027585653	0.908526057
PF4V1	0.299654694	0.10746596
PFAS	0.520269355	0.014948728
PFDN1	-0.27012665	0.073449285
PFDN2	0.021465481	0.924838339
PFDN4	-0.285701895	0.193488137
PFDN5	0.313595639	0.054969202
PFKFB1	-0.000883458	0.996189598
PFKFB2	0.02876755	0.941168506
PFKFB3	1.118747299	0.001716415
PFN1	0.353190252	0.307083533
PFN2	-0.531654425	0.051200198
PFN4	0.230188605	0.062577359
PGA3	0.168448837	0.384578677
PGA4	0.049902905	0.733411121
PGA5	0.164037816	0.407396554
PGAM1	-0.613951333	0.012242575
PGAM2	0.3519008	0.218712417
PGAP1	1.247627187	0.005020283
PGAP3	0.016559935	0.947582101
PGBD4	-0.096695338	0.601588011
PGC	0.058279665	0.540515276
PGD	-0.528919235	0.21860477
PGF	0.143819656	0.65039546
PGGHG	1.00888004	0.012554743
PGGT1B	0.869998655	0.001754124
PGK2	-0.032049657	0.774355077
PGLS	0.493649733	0.093015324
PGLYRP1	-0.070480002	0.59670211
PGLYRP2	-0.12379274	0.323217852
PGLYRP3	-0.355103884	0.018595078
PGLYRP4	-0.232629965	0.025747567
PGM1	-0.039296879	0.89848339
PGM2	0.611132344	0.076346416
PGM2L1	0.065116266	0.934842248
PGM5	0.050504198	0.757117728
PGR	-0.241547252	0.036685772
PGRMC1	-0.283519044	0.267906543
PGRMC2	0.00568207	0.985087783
PGS1	0.573844107	0.009194554
PHACTR3	-1.306392674	0.038913473
PHAX	-0.268313935	0.288468461
PHB	0.016565467	0.952200432
PHB2	0.028347176	0.910153865
PHC2	0.83091788	0.007676985
PHC3	0.235032665	0.254025679

PHEX	0.122744818	0.366900124
PHF10	0.14235001	0.541658624
PHF12	0.51029366	0.014747057
PHF13	0.201589209	0.253171949
PHF2	0.550381825	0.015808134
PHF20	-0.06304091	0.654458918
PHF20L1	0.355991759	0.014503761
PHF23	-0.054695107	0.84655506
PHF3	0.190881055	0.311482291
PHF5A	0.280801696	0.216114937
PHF6	0.136695408	0.650427205
PHF7	0.097983951	0.441340055
PHF8	0.693696897	0.001795266
PHGDH	-0.519351355	0.136957726
PHKA1	0.71057563	0.023266695
PHKA2	0.511969767	0.039667929
PHLDA1	0.849914222	0.009946858
PHLDA2	-0.125108635	0.800296892
PHLDA3	-0.232625025	0.503333093
PHLPP1	0.25453887	0.696471369
PHOX2A	-0.1454633	0.122238554
PHOX2B	-0.279708603	0.127006537
PHTF1	0.299804851	0.247370201
PHYH	-0.273356144	0.140180911
PHYHIPL	-0.026972808	0.981263887
PHYKPL	1.094481861	0.000331368
PI15	-0.333834428	0.693565527
PI3	0.657705571	0.358047105
PI4K2A	-0.762420049	0.007772609
PI4K2B	0.865208523	0.001599756
PI4KA	-0.967228152	0.02637821
PI4KB	0.358524896	0.058546766
PIANP	-1.077103077	0.008424217
PIAS1	-0.162809986	0.347139339
PIAS2	0.361895104	0.086231823
PIAS3	0.623692678	0.027764208
PIAS4	-0.084969178	0.637682435
PIBF1	0.504563983	0.028452842
PICALM	-0.261832308	0.174941557
PICK1	-0.582106953	0.01599502
PID1	0.222240359	0.549686574
PIDD1	0.227759644	0.328445379
PIEZO2	-0.600645454	0.217367864
PIF1	0.097552887	0.534214966
PIFO	0.897274174	0.057570857
PIGA	0.041684359	0.886144927
PIGC	0.381091904	0.038110947
PIGF	0.509279913	0.074669671

PIGH	-0.007189675	0.967049328
PIGK	-0.212807675	0.304645963
PIGL	1.061295682	0.003170495
PIGM	0.852099444	0.008009103
PIGN	0.574097039	0.105525119
PIGO	0.133843385	0.536650656
PIGP	0.12487219	0.522907503
PIGR	-0.014259972	0.908290896
PIGS	-0.109385869	0.650417176
PIGU	0.200892475	0.405064917
PIGW	0.206642008	0.149585962
PIGY	0.608635432	0.012967984
PIGZ	-0.259445134	0.604176491
PIK3AP1	1.029964529	0.006735106
PIK3C2B	-0.423822936	0.270838727
PIK3C2G	-0.162407678	0.101744443
PIK3C3	0.075846562	0.599695213
PIK3CA	0.024464606	0.938370209
PIK3CB	-0.310389762	0.193328904
PIK3CD	-0.232436037	0.402692904
PIK3CG	0.667541281	0.030532161
PIK3IP1	-0.022979899	0.942826832
PIK3R1	0.280228406	0.583914788
PIK3R2	-0.328150026	0.147040544
PIK3R3	-0.081565978	0.794592949
PIK3R4	0.287782587	0.220256792
PIKFYVE	0.289761212	0.076338257
PILRA	0.719678081	0.033469262
PIM1	0.540252353	0.130405822
PIM2	-0.206826435	0.407481979
PIM3	0.078491902	0.732026934
PIMREG	0.323295617	0.253119665
PINK1	-0.809817947	0.019825246
PINX1	0.126899153	0.510592076
PIP	-0.014038679	0.919158283
PIP4K2A	-1.57668613	0.006974901
PIP4K2B	-0.300791023	0.175443269
PIP4K2C	-0.645003542	0.012096199
PIP4P1	-0.028156393	0.878677857
PIP4P2	-0.262889481	0.211354254
PIP5K1B	-0.482475635	0.020517273
PIP5K1C	-0.808720643	0.009445151
PIP5KL1	-0.018588712	0.865027086
PIPOX	0.67740017	0.240239771
PITHD1	-0.470420876	0.023741155
PITPNC1	-0.079806535	0.90078881
PITPNM1	-0.719228366	0.006880123
PITPNM3	-1.813072586	0.000998715

PITX1	0.035718826	0.844156784
PITX3	-0.084657771	0.592526061
PIWIL1	-0.123312335	0.227931177
PIWIL2	0.0177147	0.909624464
PIWIL4	0.31171905	0.194551228
PJA1	-0.126443836	0.652822208
PJA2	-0.059252487	0.835493839
PKD1L1	-0.011116651	0.927976981
PKD1L3	-0.10797623	0.494972086
PKD2	0.522644448	0.045549829
PKD2L1	-0.442256554	0.01825647
PKDREJ	0.0239629	0.816422
PKHD1	0.019546614	0.842627222
PKHD1L1	-0.068862553	0.307178611
PKIA	-0.295072282	0.450619269
PKIG	0.227699581	0.281773457
PKMYT1	-0.216748573	0.259652165
PKN1	0.589059579	0.013851792
PKN2	0.60919396	0.000782716
PKN3	0.373105963	0.17915333
PKNOX1	0.22841506	0.192245975
PKNOX2	-0.418855371	0.224437799
PKP1	-0.195312336	0.179104238
PKP3	-0.130814318	0.307083533
PKP4	-1.005740005	0.016817157
PLA1A	-0.157528621	0.214181519
PLA2G10	0.110742753	0.55480355
PLA2G12A	0.085552151	0.860643453
PLA2G12B	-0.02304482	0.856424995
PLA2G15	-0.046192457	0.900082717
PLA2G1B	0.072905981	0.568327491
PLA2G2A	0.464536011	0.34567199
PLA2G2D	0.042169549	0.720000862
PLA2G2E	-0.086975711	0.373969966
PLA2G2F	-0.179149924	0.116756442
PLA2G3	-0.073630245	0.580309198
PLA2G4A	1.20140221	0.002015573
PLA2G4D	-0.104555747	0.320386929
PLA2G4E	-0.013243144	0.911188953
PLA2G4F	0.028483361	0.910283756
PLA2G5	0.749060972	0.024855338
PLA2G6	-0.014501489	0.96148544
PLA2G7	0.172025151	0.712803392
PLA2R1	-0.165275629	0.845767698
PLAAT1	0.439902472	0.261702587
PLAAT2	-0.104978754	0.439914357
PLAAT3	-1.003691899	0.012462671
PLAAT4	0.612106372	0.079148143

PLAAT5	-0.515611463	0.011653303
PLAC1	-0.018285419	0.853715871
PLAC8	0.789711704	0.02736428
PLAC9	-0.143152098	0.250506267
PLAG1	-0.370746315	0.174661502
PLAGL1	-0.507370564	0.322585459
PLAGL2	0.203919564	0.44476973
PLAT	0.802307379	0.165819901
PLAU	0.463126448	0.58264073
PLAUR	0.644504724	0.353410067
PLBD1	1.074125818	0.012043476
PLBD2	-0.350248886	0.137352678
PLCB1	-1.703636971	0.043501035
PLCB3	0.644930568	0.151406244
PLCD3	0.065433337	0.852189704
PLCG1	0.770759628	0.003951862
PLCG2	-0.048658252	0.712015348
PLCH2	-0.994442653	0.000391368
PLCL1	-1.40502926	0.026892747
PLCL2	-0.255260422	0.148833608
PLCXD1	0.29320702	0.277210723
PLCZ1	-0.064556618	0.576430021
PLD2	0.546605768	0.046796831
PLD4	0.171331512	0.65734195
PLD5	0.131511221	0.814718655
PLD6	0.056597287	0.85906445
PLEC	-0.180355358	0.598057051
PLEK	1.339697818	0.011499351
PLEK2	0.149260459	0.705247948
PLEKHA3	-0.13006081	0.504393985
PLEKHA4	1.472692622	0.003568303
PLEKHA6	-1.311135217	0.013630302
PLEKHA7	0.567437128	0.041960568
PLEKHB1	-0.709636093	0.147608175
PLEKHF1	-0.04711072	0.877135519
PLEKHF2	0.526652849	0.014906622
PLEKHG2	1.013050759	0.020819153
PLEKHG4B	-0.007240072	0.99037514
PLEKHG5	-0.428009839	0.058607587
PLEKHG7	-0.11001751	0.265986967
PLEKHH2	1.225394973	0.042537375
PLEKHH3	0.240670388	0.214566503
PLEKHJ1	0.337961263	0.116254621
PLEKHN1	-0.049935498	0.779159502
PLEKHO1	0.122199884	0.485298126
PLEKHO2	0.408782717	0.162895283
PLET1	0.055366154	0.570760866
PLG	-0.00517901	0.964586299

PLGLB1	0.164668942	0.682620106
PLGRKT	0.223443035	0.206867165
PLIN1	-0.065392659	0.537165089
PLIN3	-0.245513299	0.658719643
PLK1	1.047766522	0.045779104
PLK2	-1.059795523	0.122300037
PLK3	0.554217708	0.210066653
PLK4	0.621807886	0.181766132
PLLP	-1.456005036	0.062796258
PLN	0.021069704	0.939870806
PLOD1	0.962693917	0.049926726
PLOD2	0.683746547	0.252826729
PLOD3	0.665250766	0.060011766
PLP2	0.83013325	0.161031781
PLPBP	-0.364186484	0.063240636
PLPP1	0.069748072	0.853607455
PLPP2	-0.20346306	0.607107446
PLPP3	-0.023534839	0.967871917
PLPP6	-0.134807782	0.563322948
PLPP7	-0.177599296	0.305720857
PLPPR1	-0.427410023	0.552362529
PLPPR2	-0.021488254	0.953362739
PLPPR3	-1.285310953	0.000873762
PLPPR4	-0.488936824	0.533309644
PLPPR5	-0.273744697	0.682485682
PLRG1	0.130961948	0.521472311
PLS1	-0.41291064	0.034333794
PLS3	0.006948559	0.993855956
PLSCR1	1.391910051	0.000391899
PLTP	0.920763847	0.028516008
PLXDC2	0.288286464	0.621460507
PLXNA1	-0.053261418	0.904207344
PLXNA2	-0.047932903	0.92218993
PLXNA4	0.249704957	0.691358344
PLXNB3	0.055033299	0.910153865
PLXNC1	0.592973857	0.094591376
PLXND1	0.456617801	0.342425377
PM20D1	0.028978019	0.82820301
PMAIP1	0.006600644	0.991447462
PMCH	-0.059246442	0.690273648
PMEL	-0.226802271	0.021546998
PMEPA1	0.419940409	0.33385262
PML	0.37459465	0.329732103
PMM1	-0.253352062	0.145896036
PMM2	1.095128133	0.005391307
PMP2	1.753588499	0.094793909
PMP22	0.192513685	0.700452575
PMPCA	0.261428497	0.149721131

PMS1	0.558158323	0.00860036
PMVK	0.042400934	0.784105431
PNISR	0.664237177	0.008867317
PNKD	-0.24798612	0.065948546
PNKP	0.377039303	0.149257703
PNLDC1	-0.122834093	0.788380786
PNMA1	-0.065112356	0.893241153
PNMA3	-1.245404224	0.000962174
PNMA8A	-0.762922038	0.025549423
PNMA8B	-0.147909877	0.368362316
PNMT	-0.359077739	0.000910583
PNN	0.386627874	0.079148143
PNO1	0.407601004	0.258448956
PNOC	0.026900462	0.927976981
PNP	0.183578577	0.47590485
PNPLA1	-0.193064591	0.231113261
PNPLA3	-0.256776052	0.104795544
PNPLA4	-0.338459358	0.311681206
PNPLA5	-0.211605272	0.077524965
PNPLA6	-0.166953329	0.480832489
PNPLA8	0.260150818	0.198631193
PNPT1	0.433691436	0.021517263
PNRC1	0.067722832	0.779467068
PODNL1	0.397374898	0.203901234
POF1B	0.021319202	0.795142945
POFUT2	0.720572596	0.078966846
POGK	0.318088183	0.093764507
POGLUT2	-0.023607798	0.95239805
POGLUT3	0.881153138	0.051261944
POLA1	1.16804305	0.001056288
POLA2	0.066855258	0.824830805
POLB	-0.097273767	0.56239321
POLD3	0.401814324	0.023168931
POLD4	0.080973955	0.802533362
POLDIP2	-0.092428095	0.679450268
POLDIP3	0.202424327	0.399038792
POLE	0.671252815	0.003149269
POLE2	0.363978829	0.161843682
POLE4	-0.0447984	0.844156784
POLG	0.288468174	0.079148143
POLG2	0.848425928	0.001056288
POLH	0.562424214	0.238058584
POLI	0.404291564	0.041652485
POLK	0.603583743	0.000969074
POLM	0.581095174	0.01322427
POLQ	0.982773944	0.029057743
POLR1A	0.401921889	0.043883128
POLR1B	0.179519072	0.250483343

POLR1C	0.259976011	0.124246057
POLR1D	0.004300595	0.986404569
POLR1E	0.270367022	0.487962412
POLR2B	0.454900481	0.05383815
POLR2C	-0.356655528	0.046068116
POLR2D	-0.093026968	0.72777771
POLR2E	-0.579366509	0.004219294
POLR2F	-0.072334071	0.817881105
POLR2G	0.131581532	0.540855396
POLR2H	0.368355361	0.038372241
POLR2I	-0.408845961	0.020976102
POLR2J	1.10093011	0.001734611
POLR2J2	0.508253125	0.163567822
POLR2K	-0.177975358	0.429592789
POLR2L	0.006480753	0.986839591
POLR3A	-0.434640886	0.067411593
POLR3D	0.479083677	0.067026366
POLR3F	-0.004542663	0.991271114
POLR3G	0.149110214	0.719125255
POLR3GL	0.121729178	0.507426717
POLR3K	0.054166083	0.71316687
POM121L12	-0.26266611	0.019880663
POMGNT1	-0.03612373	0.84174162
POMGNT2	0.135762335	0.56984988
POMK	-0.011281127	0.97286117
POMP	-0.361959716	0.138969461
POMT1	0.338074973	0.037565035
POMT2	0.609073843	0.001620462
POMZP3	0.405509268	0.105888103
PON1	0.018835253	0.901777672
PON3	-0.047724196	0.752183858
POP4	0.250488864	0.5635774
POP5	0.273315262	0.050632794
POP7	-0.041133598	0.808256838
POPDC2	0.400347609	0.031197747
POPDC3	-0.75092469	0.001392079
POR	0.05757486	0.730542382
PORCN	-0.461439863	0.17485396
POSTN	1.351328798	0.397966282
POTED	-0.248614684	0.167782611
POTEE	-0.073698973	0.569159752
POTEI	-0.091577439	0.638072743
POU2AF1	-0.052004146	0.61217971
POU3F1	-0.294903637	0.052182985
POU3F2	1.020046322	0.004240643
POU3F3	0.542151087	0.220026026
POU3F4	0.263766926	0.239581231
POU4F1	0.113117128	0.704734502

POU4F2	0.030219926	0.768157782
POU4F3	-0.388957358	0.149717662
POU5F1	0.134612935	0.473614471
POU5F1B	-0.008545667	0.953957295
POU5F2	0.149922662	0.203109583
POU6F2	-0.920227787	0.015093776
PPA1	-0.762846319	0.00553036
PPA2	0.095854347	0.600651212
PPARG	-0.298743972	0.06553881
PPARGC1A	-0.091206057	0.857467846
PPARGC1B	0.016389565	0.941720118
PPAT	0.222431966	0.39123259
PPBP	0.091365137	0.822013734
PPCDC	0.118075918	0.453084845
PPCS	0.06155012	0.801083515
PPDPF	-0.18674899	0.465864696
PPDPFL	0.080878848	0.608009487
PPEF1	-0.220591764	0.396788234
PPEF2	-0.133304337	0.088481151
PPFIA2	-1.72301082	0.013906315
PPFIA3	-0.992593319	0.003417666
PPFIA4	-0.604768981	0.161329673
PPFIBP1	-0.202673735	0.638095427
PPHLN1	0.100160621	0.551909744
PPIA	0.534460601	0.044221317
PPIB	0.427283223	0.237400644
PPIC	0.638592434	0.107224994
PPID	0.142394868	0.596429187
PPIE	0.583060862	0.009312966
PPIF	-0.57609323	0.070835627
PPIG	-0.063572919	0.649648348
PPIH	0.384011041	0.004830214
PPIL1	-0.083899022	0.74874029
PPIL2	0.478736594	0.027679757
PPIL3	0.43034741	0.039899513
PPIL6	0.5940013	0.073903736
PPIP5K1	-0.89893598	0.020877788
PPIP5K2	0.259023196	0.163567822
PPL	-0.19639712	0.510276224
PPM1A	-0.131249704	0.569712378
PPM1D	0.241833171	0.396542579
PPM1E	-0.158355356	0.493707483
PPM1F	0.030467048	0.862447143
PPM1H	-1.594394923	9.92E-05
PPM1J	-0.161929844	0.318895907
PPM1K	-0.781245529	0.072379084
PPM1L	-0.471710286	0.035996134
PPM1M	0.367961275	0.05199819

PPME1	-0.636317239	0.079675036
PPP1CA	0.292730325	0.173506036
PPP1CB	0.634599115	0.001544618
PPP1CC	0.507812647	0.023364365
PPP1R10	0.087885229	0.640818885
PPP1R11	-0.18225815	0.12375102
PPP1R12A	0.023819517	0.857392501
PPP1R12B	0.011077595	0.979439064
PPP1R12C	-0.509537592	0.085490651
PPP1R13B	-0.869997091	0.009991617
PPP1R14A	-1.679584429	0.002031166
PPP1R14C	-0.150750989	0.622639969
PPP1R14D	-0.033816661	0.803713486
PPP1R15A	0.158086927	0.739429288
PPP1R15B	0.284800321	0.18088109
PPP1R16A	0.002669837	0.987724044
PPP1R16B	-2.635160006	0.000352221
PPP1R17	-0.150446131	0.329854426
PPP1R1A	-1.380982895	0.015961582
PPP1R1B	0.352402137	0.267863134
PPP1R26	-0.082238682	0.770808116
PPP1R35	0.184090077	0.49846128
PPP1R36	-0.018871376	0.882926634
PPP1R37	-0.36970641	0.012979298
PPP1R3A	0.007408782	0.954701525
PPP1R3C	-1.217509804	0.013856599
PPP1R3D	0.007347351	0.986196876
PPP1R7	-0.585666704	0.001956896
PPP1R9B	-0.558819587	0.052182985
PPP2CA	0.023173761	0.881608479
PPP2R1A	-0.856777247	0.001131282
PPP2R1B	0.175775247	0.372787239
PPP2R2B	0.129470658	0.847502786
PPP2R2D	-0.270166552	0.155683121
PPP2R3A	-0.426160074	0.321988625
PPP2R3B	0.382943461	0.084275462
PPP2R5A	-0.251304943	0.332875404
PPP2R5C	0.075991665	0.647059839
PPP2R5D	-0.435126239	0.039349851
PPP2R5E	0.138992244	0.51415788
PPP3CA	-0.880750493	0.053470148
PPP3R2	-0.186973494	0.295075692
PPP4R2	0.029406726	0.904012019
PPP4R3A	0.395463918	0.05804252
PPP4R4	-1.600990962	0.008894029
PPP5C	0.239500498	0.308004845
PPP6R1	0.222685947	0.164011584
PPP6R2	-0.190980377	0.409249316

PPP6R3	0.484874198	0.005831979
PPT2	0.358490869	0.059593665
PPTC7	0.396219548	0.043406974
PPWD1	0.441679845	0.032856063
PPY	-0.166210713	0.281519398
PRAC1	-0.045334914	0.667815566
PRAC2	-0.020762795	0.833439613
PRADC1	0.133248288	0.506185012
PRAF2	0.087081212	0.799883829
PRAM1	0.412867878	0.052792887
PRAME	0.343049187	0.512168114
PRAMEF27	-0.070377513	0.799171455
PRAMEF6	-0.25520953	0.214885024
PRAP1	-0.051715778	0.826802803
PRB1	-1.298066403	0.003174993
PRB3	-0.261548032	0.012186973
PRB4	-0.111703187	0.472228468
PRC1	1.182719627	0.017881224
PRCP	0.368029906	0.043666436
PRDM1	0.677865154	0.045365523
PRDM10	0.388570929	0.11032819
PRDM11	-0.273160706	0.218311247
PRDM12	-0.147073743	0.209247743
PRDM13	0.00256285	0.98447865
PRDM14	-0.028455823	0.821951585
PRDM15	0.162183575	0.291764429
PRDM16	-0.605366975	0.145541661
PRDM2	-0.33592502	0.104998976
PRDM4	-0.034446552	0.831972223
PRDM5	0.571331221	0.008169001
PRDM8	-0.7462898	0.06218343
PRDM9	-0.1295032	0.277203467
PRDX2	-0.58940164	0.007553782
PRDX3	-0.698464253	0.02365566
PRDX4	0.313773584	0.293451908
PRDX5	-0.236362202	0.225020885
PRDX6	-0.241981141	0.4489729
PREB	0.202519928	0.245965752
PRELID1	0.115932074	0.711938307
PRELID2	-0.229358202	0.167560462
PRELP	-0.381955024	0.38617935
PREP	-0.086496335	0.765578799
PREX1	0.521359167	0.27986152
PRF1	0.357644673	0.205129605
PRG2	1.502696153	0.001597225
PRG3	0.012734922	0.942091567
PRG4	-0.014877611	0.901377776
PRH1	-0.007634792	0.958915787

PRICKLE2	-0.519731258	0.217434794
PRICKLE3	-1.092851056	0.046525227
PRIM1	0.678632465	0.048115911
PRIM2	0.414278477	0.122585946
PRIMA1	0.070288458	0.867945633
PRIMPOL	0.98912572	0.00010541
PRKAA1	0.442735559	0.040193549
PRKAA2	-0.510516495	0.156642513
PRKAB1	0.354237139	0.072637354
PRKAB2	0.067102806	0.844156784
PRKACA	-0.262623509	0.324631071
PRKACB	-0.77448248	0.056858433
PRKACG	-0.214659674	0.11600824
PRKAG2	-0.363813766	0.152650935
PRKAG3	0.010136656	0.935244061
PRKAR1A	-0.130045589	0.571895321
PRKAR1B	-0.200423725	0.349146637
PRKAR2A	0.384131848	0.207995426
PRKAR2B	-1.308430368	0.02004471
PRKCE	-0.244716692	0.446254987
PRKCG	-1.68426734	0.007280477
PRKCI	-0.493626481	0.077786153
PRKCQ	-0.714210434	0.004162689
PRKD2	0.727459586	0.040433822
PRKD3	0.885018526	0.010749576
PRKG2	-0.152207939	0.737031972
PRKRIP1	0.841084437	0.006195115
PRKX	0.812907852	0.081206737
PRL	0.016731223	0.845289305
PRLH	-0.119137082	0.367881966
PRLHR	0.005775236	0.980092136
PRLR	-0.067165489	0.571821766
PRM1	-0.035217772	0.830717269
PRM2	0.261238374	0.115235849
PRMT2	0.393718334	0.034018658
PRMT3	0.822006216	0.002376995
PRMT5	0.079248193	0.804551372
PRMT6	-0.111606769	0.589126878
PRMT7	0.450162646	0.068740397
PRMT8	-1.353289338	0.001620311
PRMT9	0.402962564	0.065878376
PRND	-0.088505698	0.500451938
PRNP	-1.113047936	0.002175978
PROB1	0.156069701	0.356581679
PROC	0.024659715	0.855492277
PROCA1	0.177741162	0.281433263
PRODH	-0.848023363	0.078078298
PRODH2	-0.105510296	0.218183183

PROK1	-0.161469613	0.327004897
PROKR1	-0.059187562	0.666720521
PROM1	0.147617649	0.780791174
PROP1	0.059448749	0.697649656
PROS1	1.107797285	0.010200751
PROSER1	0.257060413	0.327370792
PROSER2	-0.294393117	0.073454421
PROSER3	0.552652215	0.024933462
PROX1	0.758101212	0.106511131
PRPF18	0.287992702	0.250131367
PRPF19	-0.21908328	0.195245143
PRPF31	0.270500892	0.351918897
PRPF38A	0.367719427	0.143145541
PRPF39	0.333722045	0.075884656
PRPF4	0.526962253	0.081092156
PRPF40A	0.394436739	0.060370505
PRPF4B	0.777985255	0.000447744
PRPF8	-0.116994572	0.47880118
PRPH	-0.0231592	0.885002588
PRPH2	-0.154290667	0.166149474
PRPS1	-0.034049559	0.941168506
PRPS1L1	-0.007046098	0.966203371
PRPSAP1	0.588430101	0.018535636
PRPSAP2	0.095661681	0.601588011
PRR11	0.693109044	0.224437799
PRR13	0.473580614	0.005110635
PRR14	0.394605102	0.124886222
PRR14L	-0.108747347	0.609073355
PRR15	-0.193953101	0.483366908
PRR15L	-0.165178674	0.259077956
PRR16	-0.305001556	0.223903108
PRR18	-1.174664895	0.001392079
PRR20A	-0.16271845	0.097598309
PRR27	-0.004770896	0.967752584
PRR29	0.169389476	0.466288438
PRR30	-0.00043525	0.997942116
PRR35	-0.200014224	0.211730961
PRR4	0.261814574	0.141918597
PRR5-ARHGAP8	0.043012092	0.775328942
PRR7	0.176853994	0.094240425
PRRC1	0.57670149	0.072462197
PRRC2C	0.162371027	0.239454723
PRRG1	-0.890795015	0.005445074
PRRG2	-0.248143241	0.082384816
PRRG3	-0.554090754	0.041553053
PRRG4	0.293581347	0.233586232
PRRT1	-0.48140936	0.061198429
PRRT3	-0.429834835	0.048688614

PRRX1	1.234244181	0.002150691
PRRX2	-0.454580163	0.008423911
PRSS1	-0.292966571	0.051653571
PRSS12	-0.504630531	0.055187008
PRSS16	-0.271284636	0.053470148
PRSS21	-0.115791314	0.389435147
PRSS22	-0.03538731	0.805141045
PRSS23	-0.072932089	0.909043385
PRSS27	0.129242919	0.338481031
PRSS33	-0.034605569	0.791787659
PRSS36	0.016789489	0.881105892
PRSS38	-0.097362294	0.4590178
PRSS45P	-0.179392883	0.082720016
PRSS48	-0.035317238	0.811706762
PRSS50	0.215825269	0.130444585
PRSS51	0.118869332	0.447631076
PRSS57	-0.064062195	0.723362559
PRSS58	-0.00792322	0.950574955
PRSS8	-0.045612237	0.821302228
PRTFDC1	0.373903795	0.330630871
PRTN3	-0.153481852	0.380462395
PRX	-0.106593371	0.350295763
PRXL2C	0.421524513	0.214885024
PRY	0.009095459	0.932068382
PSAP	-0.137178964	0.525315493
PSAT1	-0.441028973	0.275673333
PSD	-1.21059215	0.001401204
PSD2	-1.063572502	0.142381638
PSD4	0.117577079	0.527496385
PSEN1	-0.47043329	0.040433822
PSEN2	0.077277334	0.566497648
PSENEN	0.358284374	0.111431621
PSG1	-0.057557662	0.682485682
PSG11	-0.018789594	0.922182479
PSG3	-0.263990234	0.052182985
PSG4	-0.064187545	0.753117676
PSG5	-0.056446571	0.718751008
PSIP1	-0.069734955	0.851455746
PSKH1	0.293221549	0.226693178
PSKH2	-0.298164285	0.070786463
PSMA1	0.374834816	0.084913957
PSMA2	0.436442847	0.090828268
PSMA3	0.349285304	0.022050211
PSMA4	0.136592723	0.522847527
PSMA5	0.178685862	0.430567574
PSMA6	0.269980003	0.089683623
PSMA7	0.112803451	0.582941588
PSMA8	-0.057690877	0.530273258

PSMB1	-0.236165951	0.20690805
PSMB10	0.269346566	0.296863641
PSMB2	0.153550312	0.468354165
PSMB4	0.326436691	0.041444988
PSMB6	-0.155460691	0.669970192
PSMB7	-0.129738078	0.468354165
PSMB8	0.935102306	0.005023435
PSMB9	0.830532868	0.00055525
PSMC1	-0.010040315	0.960903847
PSMC2	0.19549718	0.293871366
PSMC3	-0.107712777	0.508340807
PSMC4	0.04483602	0.864502447
PSMC6	0.272295063	0.121027454
PSMD1	-0.060536935	0.782208437
PSMD10	-0.227344207	0.194570323
PSMD11	0.36170745	0.17152569
PSMD12	-0.040889517	0.888106292
PSMD13	0.221197496	0.234889728
PSMD14	-0.01492677	0.958982209
PSMD2	-0.04314807	0.872447647
PSMD3	0.179110793	0.194885839
PSMD4	0.236364365	0.123801057
PSMD6	0.131917505	0.431685736
PSMD7	-0.48443372	0.022175525
PSMD8	-0.434627132	0.071218501
PSME1	0.067684095	0.792979636
PSME2	0.633597163	0.020411052
PSME4	0.689442482	0.056874602
PSMF1	-0.14711411	0.366597455
PSMG1	-0.238570476	0.382195809
PSMG2	0.103723912	0.681406422
PSMG3	0.44873304	0.032425399
PSMG4	0.414709116	0.001776331
PSORS1C1	0.34068017	0.204441277
PSORS1C2	0.204180908	0.203901234
PSPH	0.703259906	0.008448974
PSRC1	0.386444643	0.401249386
PSTK	-0.273403858	0.052115212
PSTPIP1	0.018226858	0.899700466
PSTPIP2	-0.217365533	0.646942576
PTBP1	0.830133696	0.041889564
PTBP2	0.245974499	0.382513211
PTBP3	0.186563978	0.553475251
PTCD2	0.158158587	0.500918577
PTCD3	0.316969709	0.096985686
PTCH2	0.026576384	0.946788797
PTCHD1	0.246506029	0.653672898
PTDSS1	-0.001368065	0.997942116

PTDSS2	-0.111884416	0.398866706
PTEN	0.076343683	0.749350052
PTF1A	-0.289505397	0.004064451
PTGDR	0.001931307	0.992886227
PTGDR2	-0.017332854	0.901052359
PTGDS	-1.78238071	0.028631206
PTGER1	-0.201043424	0.238517657
PTGER4	0.630373543	0.110354212
PTGES	-0.118049821	0.591000178
PTGES2	-0.093622187	0.604513223
PTGES3	0.406977764	0.004002042
PTGFR	-0.05805358	0.726307053
PTGFRN	1.717918219	0.01119529
PTGIR	0.034431389	0.910685398
PTGIS	-0.332409196	0.471442677
PTGS1	0.94483282	0.019993679
PTGS2	0.118162281	0.857443332
PTH	-0.060257777	0.566497648
PTH1R	-0.183401713	0.412315933
PTH2	-0.231688297	0.217414475
PTH2R	-0.052876463	0.912618675
PTHLH	-0.125780178	0.788340109
PTK2	-0.241710277	0.201140539
PTK2B	-0.636933804	0.247249456
PTK6	-0.125258246	0.582941588
PTMS	0.149380715	0.56757765
PTN	1.07381219	0.074266856
PTP4A1	-0.02921546	0.920298284
PTP4A2	-0.146432809	0.506781957
PTP4A3	1.468695254	0.000389052
PTPA	-0.436918425	0.009134728
PTPDC1	-0.245108846	0.449083281
PTPMT1	-0.065599467	0.735925036
PTPN1	0.328883563	0.259768536
PTPN12	0.960175818	0.003741174
PTPN13	0.681503774	0.010744892
PTPN14	-0.239601673	0.563322948
PTPN18	0.438881305	0.080954866
PTPN21	0.23745102	0.534409435
PTPN22	0.249146747	0.144002691
PTPN23	0.042966008	0.847725163
PTPN3	-0.623948105	0.159943598
PTPN4	-0.652971814	0.074332346
PTPN6	0.649844329	0.012301856
PTPN7	0.270078697	0.096068807
PTPN9	0.054272237	0.893583861
PTPRC	1.204964984	0.01308988
PTPRCAP	0.100322804	0.560024501

PTPRD	-1.708831788	0.021148735
PTPRE	0.53231737	0.093970275
PTPRF	-0.346120457	0.094233344
PTPRG	0.345014565	0.163567822
PTPRH	-0.159819918	0.242784226
PTPRJ	0.185780636	0.502354128
PTPRN	-1.705148509	0.008737673
PTPRO	-0.018743901	0.97405834
PTPRR	-1.163868363	0.005161235
PTPRS	-0.046998161	0.905092701
PTPRZ1	2.566236489	0.002757016
PTRH1	0.022144755	0.88985577
PTRHD1	-0.071460429	0.616944401
PTS	0.340632504	0.176007134
PTTG1	0.070124799	0.675122539
PTTG2	0.216038783	0.23805142
PTX3	1.388220567	0.094442086
PUF60	0.208387907	0.253718431
PUM1	0.33100298	0.039200246
PUM2	0.293925078	0.08331423
PUM3	0.369085449	0.204477428
PURB	0.237470513	0.239227004
PUS1	0.084381792	0.576288716
PUS3	0.091403305	0.646887867
PUS7	0.460789222	0.137948025
PUSL1	0.069769985	0.723856561
PVALB	-0.439359262	0.008424217
PVRIG	0.012849189	0.965067493
PWP1	0.199616053	0.230229256
PWP2	0.295532905	0.245965752
PWWP2B	-0.717498423	0.006974901
PWWP3B	-0.675214867	0.009412305
PXDC1	0.696884239	0.051272131
PXDNL	0.362589142	0.247587959
PXMP2	-0.004079376	0.991179488
PXMP4	0.0706538	0.74315299
PXT1	-0.151050982	0.078394754
PXYLP1	0.742234302	0.004842068
PYCARD	0.319637538	0.302277667
PYCR1	0.093067684	0.770808116
PYCR2	0.392649174	0.01197672
PYCR3	0.142203042	0.473389208
PYDC1	-0.141434802	0.266750083
PYGL	1.740927001	0.002565396
PYGM	-1.214218515	0.010949353
PYGO1	0.164502288	0.627758643
PYROXD1	-0.092911681	0.723956289
PYY	-0.283285863	0.147757533

PZP	0.009632974	0.944326644
QDPR	-1.430941242	0.001910109
QKI	0.549346766	0.136223183
QPCT	-0.525572529	0.072637354
QPCTL	-0.103387329	0.789957096
QPRT	-0.081861101	0.788249312
QRFP	0.341541071	0.048776856
QRFPR	-0.055158173	0.527536022
QRICH1	0.033779419	0.809020028
QRICH2	0.345372235	0.27289831
QRSL1	0.671188944	0.030568757
QSOX1	-0.444293988	0.307475995
QSOX2	0.707793199	0.001056288
QTRT2	0.334624927	0.08399916
R3HCC1	-0.173440156	0.338323678
R3HDM1	-1.179064902	0.003247194
R3HDM2	-0.192139282	0.503964238
R3HDM4	-0.096183284	0.704169763
R3HDML	-0.068008157	0.527763948
RAB10	0.331142322	0.007315104
RAB11A	-0.071035429	0.478333461
RAB11B	0.029772045	0.848699395
RAB11FIP1	-0.377725499	0.077000956
RAB11FIP2	-0.786783071	0.016945972
RAB11FIP3	0.289320009	0.2965381
RAB11FIP4	-2.104959974	0.004594929
RAB13	0.444110316	0.004412189
RAB14	-0.195943652	0.229609917
RAB15	-0.934621989	0.010927461
RAB17	-0.081882481	0.401477205
RAB1A	0.226026613	0.136468749
RAB1B	-0.004724027	0.986045302
RAB20	0.50538325	0.066664283
RAB21	-0.094350523	0.547525935
RAB22A	-0.052491866	0.792932535
RAB23	0.425639219	0.220825577
RAB25	-0.024409245	0.899858876
RAB26	-0.688691027	0.041262891
RAB27A	0.181388496	0.462224028
RAB27B	-0.735943724	0.329369557
RAB28	0.639176918	0.000266541
RAB2A	0.150414124	0.390444458
RAB2B	0.183237886	0.308169445
RAB30	-0.417059095	0.084556199
RAB31	0.417787894	0.179965296
RAB32	-0.138467835	0.655757308
RAB33A	-0.323493816	0.099266337
RAB33B	0.278748387	0.304700588

RAB34	0.135264798	0.718341166
RAB35	0.040676388	0.817903256
RAB36	0.146321178	0.626774792
RAB38	0.069516723	0.825240544
RAB39A	0.909193753	0.054474658
RAB39B	-0.090147448	0.796879518
RAB3A	-1.366422229	0.007465195
RAB3B	-1.121591499	0.024618793
RAB3C	-0.969352932	0.064015775
RAB3IL1	0.160910551	0.462455166
RAB3IP	0.710952035	0.008580383
RAB40A	0.07872458	0.635260154
RAB40B	-1.680333939	0.00051205
RAB42	0.221785931	0.363847333
RAB4A	0.059662845	0.872447647
RAB5A	0.042322691	0.847502786
RAB5C	0.492539168	0.189491289
RAB6A	-0.305129403	0.285713688
RAB6B	-0.988403534	0.137352678
RAB8B	0.620267041	0.006675668
RAB9A	0.363954835	0.12597831
RAB9B	-0.124126967	0.536160749
RABAC1	-0.332123084	0.174059618
RABEP1	-0.441303792	0.069570474
RABEP2	-0.013888259	0.953024606
RABEPK	0.239283635	0.163309963
RABGAP1	-0.093256676	0.632964128
RABGAP1L	0.076781627	0.674828435
RABGEF1	0.179889071	0.431619733
RABGGTA	-0.08179084	0.708923274
RABGGTB	0.443492809	0.023665741
RABIF	-0.160437566	0.270674947
RABL2A	0.30858006	0.166501994
RABL3	-0.243440695	0.194021622
RAC1	0.213933505	0.131026245
RAC2	0.0985038	0.743546189
RAC3	-0.456782084	0.035137901
RACGAP1	0.497016351	0.013530596
RACK1	0.662021285	0.001392079
RAD1	-0.000309421	0.998986205
RAD17	0.506174552	0.065693801
RAD18	-0.337394674	0.445186396
RAD23A	0.078907792	0.690273648
RAD23B	0.151773952	0.331100069
RAD51	0.065923468	0.862276842
RAD51AP2	-0.146772332	0.063306681
RAD51B	0.108072758	0.396171568
RAD51C	-0.125185152	0.696698187

RAD52	0.572863717	0.00126644
RAD54B	0.593541159	0.055158612
RAD54L	0.690213472	0.041692864
RAD9A	0.617799474	0.007233797
RAD9B	0.14614035	0.203901234
RADX	0.569176715	0.253119665
RAE1	-0.046270045	0.799328416
RAET1E	-0.244535396	0.022050211
RAET1L	-0.072093308	0.549686574
RAG1	0.052899815	0.554271524
RALA	0.507735951	0.003876591
RALB	-0.382409272	0.079163191
RALBP1	-0.280080143	0.196209899
RALGAPA1	-0.030561369	0.934380733
RALGAPB	0.084985637	0.542314727
RALGDS	-0.044333941	0.919802284
RALGPS2	0.080679824	0.884920673
RALY	0.238316499	0.190374885
RAMAC	0.257202248	0.344975317
RAMP1	0.586717283	0.082281418
RAMP2	0.24011626	0.490877089
RAMP3	0.150161796	0.420536191
RAN	0.323892621	0.083689501
RANBP10	0.235981138	0.25431304
RANBP17	-0.377879115	0.481954142
RANBP2	-0.084219976	0.736640271
RANBP6	-0.439823342	0.055452669
RANBP9	-0.219868896	0.334971664
RANGAP1	-0.828236814	0.000392073
RAP1A	0.081653744	0.690273648
RAP1B	0.666146345	0.025034029
RAP1GAP2	-1.593010619	0.007161481
RAP2A	0.004822372	0.993666428
RAP2B	0.536984098	0.016278382
RAP2C	0.370523685	0.080694474
RAPGEF4	-1.845805145	0.051665185
RAPGEF5	-1.907998953	0.00275489
RAPGEFL1	-0.841063183	0.034855955
RAPH1	-0.064829265	0.851301109
RAPSN	0.116856962	0.402775257
RARA	0.689653012	0.006974423
RARB	-0.813120639	0.041226578
RARRES1	0.136038574	0.559010743
RARRES2	0.450956823	0.16925862
RARS2	0.401677303	0.106926699
RASA1	0.102949227	0.580193623
RASA2	0.44895809	0.08144142
RASA3	0.332682194	0.321226681

RASA4	0.567684006	0.235819875
RASA4B	0.88874749	0.053470148
RASD1	0.078031753	0.927976981
RASD2	-0.949934002	0.025293987
RASEF	0.234366531	0.433770317
RASGEF1A	-0.750556416	0.011829886
RASGEF1B	0.379915167	0.217502534
RASGEF1C	0.069901468	0.815126481
RASGRF1	-2.168054158	0.000635034
RASGRF2	-2.54264949	0.000969074
RASGRP1	-0.463466382	0.207311857
RASGRP2	0.044765753	0.837559998
RASGRP3	0.129064772	0.767294518
RASGRP4	0.195671682	0.318895907
RASL10A	-0.337730287	0.138417553
RASL10B	-0.98079028	0.001234825
RASL11A	-0.042128881	0.774227942
RASL11B	0.051429776	0.936643326
RASL12	0.19013798	0.516380268
RASSF1	0.04813893	0.811305444
RASSF2	0.68122553	0.340627648
RASSF3	0.279413071	0.313353465
RASSF4	0.911185581	0.011740569
RASSF6	0.016479512	0.86678759
RASSF7	0.055396739	0.717407272
RASSF9	-0.091222982	0.845586637
RAVER2	0.208747109	0.599418528
RAX	-0.10457629	0.447239377
RAX2	-0.218185727	0.131501559
RB1	0.738911895	0.008556158
RB1CC1	-1.164164699	0.00062386
RBBP4	0.461396017	0.009571203
RBBP6	0.488348038	0.010292284
RBBP7	0.862986073	4.88E-05
RBBP8	0.820070867	0.017969795
RBBP8NL	-0.06667553	0.665904933
RBBP9	0.13979396	0.765830743
RBCK1	0.579868506	0.00699121
RBFA	0.349128449	0.063747636
RBKS	-0.105659419	0.416336033
RBL1	1.080558195	0.001871944
RBL2	0.270017476	0.149630608
RBM10	0.484853072	0.006471398
RBM11	-0.443174465	0.057102086
RBM12	0.463044634	0.008749587
RBM12B	0.854156436	0.001099778
RBM15	1.021190241	0.000643038
RBM15B	0.049650807	0.803047625

RBM19	0.429948068	0.031787603
RBM22	0.490647267	0.009312966
RBM24	-0.601195629	0.040927029
RBM25	0.075910281	0.692586142
RBM26	-0.043749556	0.871201192
RBM3	1.077072737	5.06E-05
RBM33	0.699217307	0.016287545
RBM34	0.311619938	0.215764173
RBM38	0.531105088	0.008460059
RBM4	0.438389248	0.01467734
RBM41	0.630399266	0.013280653
RBM42	0.117639799	0.619107892
RBM43	0.415468225	0.00872419
RBM46	0.014886071	0.918837703
RBM47	0.561766873	0.009451151
RBM48	0.274976876	0.179337637
RBM4B	0.689592893	0.002280221
RBM5	0.717144855	0.00300851
RBM6	0.765013386	0.003979493
RBM7	0.529534302	0.145336887
RBMS1	0.604755397	0.121743152
RBMS3	-0.104698991	0.79818783
RBMX	0.539342592	0.047536049
RBMX2	0.104876382	0.480832489
RBMY1A1	-0.091198697	0.371135496
RBMY1D	-0.111804586	0.378881173
RBMY1E	-0.136613921	0.267549543
RBMY1F	-0.111309724	0.277618337
RBP1	0.474620193	0.350428516
RBP2	-0.01913951	0.881608479
RBP3	0.004549718	0.971953247
RBP4	-1.6083878	0.002341058
RBP5	0.098970838	0.566497648
RBP7	-0.799907974	0.003075463
RBPJ	0.117147026	0.608177639
RBPJL	-0.028950044	0.888379819
RBSN	0.056442402	0.782749113
RBX1	-0.038542207	0.861019362
RC3H1	0.156865914	0.275812696
RCAN1	0.762208587	0.038372241
RCBTB1	-0.062289167	0.86089558
RCC1L	0.441414533	0.022393652
RCC2	1.066913793	0.001834847
RCHY1	0.103072032	0.680511492
RCL1	0.131932776	0.360423208
RCN1	0.757018186	0.048380481
RCN2	0.120715818	0.65640162
RCN3	0.410127096	0.435455714

RCOR1	0.684291375	0.029360999
RCOR2	0.433464575	0.313708077
RCSD1	0.204168713	0.324819162
RCVRN	-0.267450097	0.282032148
RD3	0.130433787	0.414462192
RDH10	0.821587776	0.032813584
RDH11	0.013397011	0.959590568
RDH12	-0.017264152	0.902522899
RDH16	-0.002485927	0.990388651
RDH8	-0.071703581	0.574759224
RDX	0.825193428	0.014166764
REC114	-0.003125117	0.981263887
RECK	-0.335524783	0.369103156
RECQL	0.918256906	0.008943538
RECQL4	-0.003339884	0.992505943
REEP2	-0.602612994	0.039397705
REEP3	0.361005424	0.368362316
REEP4	0.544140196	0.066664283
REEP5	-0.58705071	0.028452842
REEP6	-0.881857879	0.005492418
REG1A	0.022436757	0.845746964
REG1B	-0.19486168	0.24630937
REG3A	-0.064272301	0.57993217
REL	0.534179053	0.130031819
RELB	0.224722694	0.435455714
RELCH	-0.009606457	0.964639376
RELL2	-0.84018455	0.002310018
RELN	-0.319344246	0.51415788
RELT	0.314277125	0.109530663
REM1	0.021164079	0.95239805
REM2	-0.394417924	0.003196079
REN	-0.066179457	0.454235198
RENBP	0.652802615	0.037997415
REPS1	-0.199771397	0.507144787
REPS2	-2.244828597	0.009851752
RERE	-0.328606274	0.127006537
RERG	-0.743954345	0.019789186
RERGL	-0.306421832	0.010371018
RESF1	0.464262728	0.001053722
REST	0.552974416	0.099639765
RET	-0.170902183	0.405273771
RETN	0.042463085	0.802283726
RETNLB	0.039713151	0.726378259
RETREG2	-0.229296555	0.123246343
RETSAT	-0.124889955	0.568950838
REV1	0.384911251	0.011311993
REV3L	0.82892748	0.014785217
REX1BD	-0.049831423	0.826798333

REXO1	-0.329741035	0.109530663
RFC1	0.529850953	0.006623459
RFC2	1.019400128	0.004003629
RFC3	1.005339128	0.006329299
RFC4	0.740662919	0.011942164
RFC5	0.64373665	0.00261964
RFK	-0.312374855	0.121322442
RFLNB	0.228909246	0.5972046
RFPL1	0.043415053	0.846369748
RFPL4A	0.032187514	0.748431761
RFPL4AL1	-0.037652711	0.840666604
RFT1	0.724930856	0.018807195
RFTN1	-0.117684252	0.745153139
RFTN2	0.288595908	0.668259657
RFWD3	0.950390088	0.015719444
RFX1	-0.040976681	0.782002897
RFX2	0.719838675	0.059344288
RFX3	0.594774284	0.155321388
RFX4	1.35434203	0.013851792
RFX5	0.138237243	0.504393985
RFX6	-0.103763781	0.265476116
RFX7	-0.000328376	0.999086955
RFX8	0.231067977	0.496083712
RFXANK	0.549388693	0.011056649
RFXAP	0.343690683	0.056043915
RGCC	0.679826114	0.410413003
RGL1	-0.293621577	0.267903967
RGL2	0.667399375	0.01413174
RGL4	-0.13630506	0.862269669
RGN	0.068221819	0.864567865
RGP1	-0.02883723	0.859529021
RGPD2	-0.050107025	0.820606478
RGS1	1.570081339	0.071614528
RGS10	0.443408388	0.296280307
RGS11	-0.683851642	0.220549716
RGS12	0.751923406	0.006777787
RGS13	0.076329053	0.425368046
RGS14	-0.260762965	0.019825246
RGS16	0.882733175	0.051261644
RGS17	0.461189476	0.189824623
RGS18	0.209379323	0.19285978
RGS19	0.337992216	0.257683688
RGS2	1.428450029	0.011740569
RGS20	-0.41875542	0.014650406
RGS22	0.712579194	0.043049402
RGS3	-0.014888875	0.928511726
RGS6	0.256586062	0.614768396
RGS7	-1.683385241	0.032589875

RGS7BP	-1.319393897	0.097030992
RGS8	-0.015334541	0.977315182
RGS9	-0.016268564	0.970066069
RHAG	-0.081727033	0.514642427
RHBDD3	-0.009705925	0.96045146
RHBDF2	0.594311265	0.196627447
RHBDL1	-0.561029601	0.007044968
RHBDL2	-0.543293904	0.007833485
RHBDL3	-0.306002695	0.220134489
RHBG	0.024074485	0.861019362
RHCE	0.061155709	0.735607376
RHCG	-0.345895555	0.018550439
RHD	-0.250296601	0.109350636
RHEB	0.170320173	0.347223044
RHEBL1	-0.101468897	0.432345774
RHEX	-0.085649326	0.291211053
RHO	-0.071605073	0.755392658
RHOA	-0.040575074	0.852908451
RHOB	0.331755243	0.217900828
RHOBTB3	0.127701449	0.710300146
RHOC	0.272537184	0.159863387
RHOD	-0.324096902	0.327370792
RHOF	-0.292495413	0.093764507
RHOG	-0.213600466	0.317452401
RHOH	-0.170495221	0.058622294
RHOJ	1.009665189	0.074372269
RHOQ	0.653899662	0.002017803
RHOT2	0.194383459	0.562498279
RHOU	0.074252824	0.90145729
RHOV	-0.026799056	0.846092035
RHOXF1	-0.061488306	0.595889777
RHOXF2B	-0.021122436	0.896420697
RHPN1	0.387941273	0.208984508
RHPN2	0.468324009	0.161642263
RIBC2	0.22986095	0.437430306
RIC8A	-0.183529739	0.363731178
RIC8B	0.469348982	0.020961619
RICTOR	-0.072394605	0.821001661
RIDA	-0.122185797	0.764982968
RILP	-0.465361791	0.010454593
RILPL2	0.208523471	0.367711309
RIMBP2	-1.427397071	0.006614098
RIMKLA	-0.639220239	0.23670507
RIMS1	-2.721952743	0.001009748
RIMS2	-2.703163572	0.000644185
RIMS3	-1.941251353	0.001072162
RIN3	0.320718953	0.234063981
RIOK1	0.577546284	0.007327295

RIOK2	0.182625532	0.356472461
RIOK3	0.324685602	0.102503489
RIOX1	-0.404596284	0.081613897
RIPK1	0.876283743	0.003196079
RIPK2	0.671725774	0.025937403
RIPK4	-0.123163294	0.583914788
RIPPLY3	-0.125005829	0.216114937
RIT2	-0.007965738	0.99037514
RITA1	-0.221160562	0.189557746
RLBP1	-0.05218631	0.925179867
RLF	-0.115852255	0.698224223
RLN1	-0.219226757	0.152605297
RLN2	-0.02485305	0.82820301
RLN3	0.03368413	0.81669995
RMC1	0.26994693	0.131425384
RMDN1	0.601956231	0.007315104
RMDN2	0.138433256	0.53183769
RMDN3	-0.122260457	0.597734018
RMI1	0.293658816	0.23807847
RMI2	0.190582823	0.241986251
RMND1	-0.167188394	0.607426166
RMND5A	-0.300212387	0.178947433
RMND5B	0.058416888	0.726545287
RNASE1	-1.198091172	0.014073797
RNASE2	2.121210618	0.002383866
RNASE3	0.25265137	0.301306841
RNASE6	1.383515848	0.010349778
RNASE7	0.086708138	0.516248742
RNASE8	-0.175139429	0.061498739
RNASE9	-0.043149221	0.635955798
RNASEH1	0.164678458	0.257553945
RNASEH2A	0.559903436	0.080074179
RNASEH2B	0.052912565	0.84046247
RNASEH2C	-0.033000685	0.863539486
RNASEL	0.168054165	0.357808768
RNASET2	-0.302832626	0.278290025
RND1	-0.493774226	0.116771295
RND2	1.116928264	0.010345901
RNF10	0.061597466	0.600991795
RNF103	-0.378255501	0.024305591
RNF11	-0.78505993	0.002707241
RNF111	-0.174735923	0.127380124
RNF112	-0.344179535	0.174250255
RNF113B	-0.180226954	0.122957034
RNF122	0.806248859	0.040293711
RNF123	-0.167864628	0.3184098
RNF125	-0.160000866	0.59150484
RNF128	-0.64647525	0.051933883

RNF13	-0.027585907	0.94768647
RNF130	0.209960959	0.484924063
RNF133	0.024487609	0.852189704
RNF135	0.465279364	0.116419877
RNF138	0.401222308	0.025596149
RNF139	-0.064040762	0.769734898
RNF14	-0.046400681	0.792479756
RNF141	-0.239206593	0.273422182
RNF144A	0.181433286	0.752202506
RNF144B	0.449890014	0.142277196
RNF148	-0.010064873	0.975644295
RNF149	0.452335126	0.098350432
RNF152	-0.262782777	0.481392472
RNF166	0.370206277	0.228524446
RNF167	-0.239521786	0.122247398
RNF168	0.14749744	0.562310445
RNF17	-0.110521316	0.129528093
RNF175	-0.540099223	0.102956472
RNF181	0.060750389	0.822572635
RNF183	-0.019199823	0.869766914
RNF185	0.237337224	0.59797176
RNF186	0.015608064	0.903996278
RNF187	-0.283477507	0.16732834
RNF19A	0.654201353	0.017399266
RNF19B	0.133286518	0.61217971
RNF2	0.377715971	0.12375132
RNF20	0.104926844	0.65039546
RNF207	-0.669565096	0.014379774
RNF212B	-0.111854988	0.157594067
RNF214	0.337914381	0.055158612
RNF215	-0.078454365	0.668259657
RNF216	0.513061827	0.013460729
RNF217	-0.21270872	0.477248291
RNF220	-0.673075258	0.006663931
RNF24	0.273288138	0.175487696
RNF25	-0.084090317	0.583881916
RNF26	-0.091649815	0.776457433
RNF31	1.117281153	0.000814384
RNF34	0.089901941	0.604795111
RNF38	-0.114647747	0.553100776
RNF44	0.050828731	0.856635804
RNF6	-0.213024959	0.326832679
RNFT1	0.621525727	0.031618823
RNGTT	-0.055146618	0.795121827
RNH1	-0.409679821	0.061668141
RNLS	-0.708425423	0.002149243
RNMT	0.093251054	0.752870608
RNPC3	0.178791149	0.699363253

RNPEP	0.377903769	0.175622233
RNPEPL1	-0.161294699	0.407481979
RNPS1	-0.040112796	0.854389834
RO60	0.612117816	0.014092274
ROBO3	0.037789098	0.830717269
ROBO4	0.477981558	0.041005164
ROCK1	0.229864489	0.271505754
ROCK2	-0.13005879	0.616944401
ROM1	0.276316782	0.263632626
ROMO1	-0.004379932	0.979485412
ROPN1	-0.165147818	0.188494462
ROPN1L	0.267987043	0.62738062
ROR1	-0.009494702	0.99128072
ROR2	0.193650295	0.478877114
RORA	0.297569151	0.44673969
RORB	-1.333112573	0.113241854
RORC	-0.063887479	0.658535487
ROS1	-0.718546449	0.02150143
RP1	0.012771032	0.899317206
RP1L1	-0.145009677	0.242884418
RP2	1.175032712	0.004056506
RP9	0.759908808	0.016287042
RPA1	0.51737378	0.009571203
RPA2	0.568497462	0.000613602
RPA3	0.327181675	0.080680741
RPA4	0.101599918	0.463668515
RPAP1	0.242232161	0.304273853
RPAP2	-0.195821968	0.415320372
RPAP3	0.244320351	0.163552869
RPE	0.365502989	0.141452995
RPE65	0.785795239	0.045697721
RPF1	0.504109119	0.019702345
RPF2	0.357275061	0.316979901
RPGR	0.900747523	0.001232424
RPGRIP1	-0.136219055	0.211820413
RPIA	0.454307204	0.023949051
RPL10	0.335355086	0.05199819
RPL10A	-0.112314503	0.643603569
RPL10L	-0.006413352	0.959862565
RPL11	0.224819091	0.291312962
RPL12	0.046625885	0.879932781
RPL13A	0.315246652	0.093938626
RPL15	-0.054840802	0.779164783
RPL17	-0.091759017	0.695913541
RPL18	-0.107018848	0.628748167
RPL21	0.217996654	0.142041718
RPL22	-2.613639147	0.000885479
RPL22L1	0.22270817	0.389834925

RPL23	0.485532952	0.050089771
RPL23A	0.338118079	0.250699139
RPL24	-0.007784235	0.967768864
RPL26	-0.074280175	0.632430202
RPL26L1	0.432687261	0.023869263
RPL27A	0.304023469	0.188234188
RPL28	0.098825207	0.607107446
RPL29	0.112153149	0.705433075
RPL3	0.051955027	0.816408453
RPL30	0.232490931	0.28073347
RPL32	0.203722803	0.310474997
RPL34	0.012080359	0.959740431
RPL35	-0.042901425	0.869441328
RPL35A	0.008491397	0.967748239
RPL36	-0.69032304	0.008098465
RPL36AL	-0.186709216	0.291653221
RPL37	-0.345410831	0.243417181
RPL37A	0.141469632	0.494966878
RPL38	-0.172113032	0.227175047
RPL39	0.084139551	0.700121086
RPL39L	0.075112958	0.866703923
RPL3L	-0.19807589	0.159943598
RPL4	-0.158622332	0.475052564
RPL41	-0.289120898	0.118163756
RPL7	-0.164531852	0.056582965
RPL7L1	0.270683293	0.127500859
RPL8	0.144838422	0.513249713
RPL9	0.39239012	0.041960568
RPLP0	0.380195031	0.073188902
RPLP1	0.263514218	0.488751358
RPLP2	0.085044261	0.587548898
RPN1	0.285852808	0.183211794
RPN2	0.920956267	0.003449103
RPP25	-0.10649278	0.574209412
RPP25L	-0.096806644	0.521819382
RPP38	0.04741682	0.725355865
RPP40	0.321542152	0.214566503
RPRD1A	0.097460155	0.629974353
RPRD1B	0.353878204	0.041552435
RPRD2	0.042841143	0.811018604
RPRM	-0.193844879	0.127118786
RPRML	-0.203582204	0.371124935
RPS11	-0.096491535	0.658430807
RPS12	0.149526415	0.422625176
RPS13	0.13567848	0.66988891
RPS15	-0.60070255	0.012317094
RPS15A	-0.341042649	0.409052598
RPS16	0.178215918	0.536880917

RPS17	-0.951580927	0.001053722
RPS19	0.48007091	0.2529387
RPS19BP1	-0.178548919	0.209830984
RPS2	0.136825693	0.704780487
RPS20	0.116009073	0.625222652
RPS21	-0.37706163	0.141930369
RPS23	-0.400537132	0.120199333
RPS25	0.908255709	0.003789735
RPS26	0.101999213	0.711938307
RPS27	-0.44920268	0.076127964
RPS27A	-0.27303446	0.160883971
RPS27L	0.362031387	0.227849141
RPS28	-0.204204115	0.518220879
RPS29	-0.014220093	0.957332066
RPS3	0.21167584	0.629366653
RPS3A	0.527972335	0.008930363
RPS4X	0.206904728	0.272446904
RPS4Y1	0.480297275	0.207045825
RPS5	-0.043171414	0.923050547
RPS6	0.034149797	0.905260794
RPS6KA1	0.09587816	0.710630056
RPS6KA3	0.472716029	0.023487605
RPS6KA5	-0.828524508	0.046770639
RPS6KA6	-0.676548162	0.006188209
RPS6KB1	0.516755876	0.0104977
RPS6KC1	-0.447830094	0.022878349
RPS6KL1	-0.627459552	0.024362454
RPS7	0.22582655	0.173093618
RPS8	0.144850246	0.539346863
RPS9	0.094234426	0.6693843
RPSA	0.485133388	0.007092374
RPUSD1	-0.132368794	0.68009459
RPUSD2	0.082215486	0.689908567
RPUSD3	-0.161612212	0.469613201
RPUSD4	0.065644307	0.631200083
RRAGA	-0.237685821	0.130613815
RRAGB	0.316181522	0.082556089
RRAGC	0.205128359	0.263839378
RRAGD	0.418263363	0.329463009
RRAS	-0.6495944	0.225745688
RRH	0.161619573	0.221643074
RRM1	0.35759485	0.166149474
RRM2	0.321979033	0.415378611
RRN3	-0.013824418	0.963152726
RRNAD1	0.513183506	0.002341058
RRP1	0.055385206	0.815673957
RRP12	-0.270941311	0.136957726
RRP15	0.363419723	0.093135402

RRP7A	0.140934482	0.615329823
RRP8	0.157548827	0.301050499
RRP9	0.137316113	0.409869127
RRS1	0.260185177	0.420543567
RS1	-0.031644767	0.740924048
RSAD1	0.3524868	0.045663086
RSAD2	0.91454202	0.005293384
RSBN1	0.166238315	0.507426717
RSBN1L	0.317212409	0.181118964
RSF1	-0.034097932	0.879255634
RSL1D1	0.104464437	0.719087761
RSL24D1	-0.024178425	0.923498499
RSPH1	0.501856312	0.338352185
RSPH10B	-0.083691519	0.560024501
RSPH14	-0.135380448	0.189498076
RSPH3	0.022263899	0.945087192
RSPH6A	0.184754315	0.086333407
RSPH9	-0.247689223	0.035090341
RSPO2	-0.771282761	0.021330379
RSPO3	-0.545471124	0.008460059
RSPRY1	0.659235146	0.073454181
RSRC1	0.221766951	0.100200988
RSRP1	0.7954973	0.008222173
RSU1	0.013063935	0.978582066
RTBDN	-0.472614244	0.000902126
RTCB	0.094561527	0.6712973
RTF1	-0.455552802	0.10214248
RTF2	0.026718213	0.868894234
RTKN	0.059853892	0.910717577
RTKN2	0.474468536	0.271505754
RTL3	-0.301110499	0.01673506
RTL4	-0.311465144	0.01990602
RTL6	-0.012057123	0.963634773
RTL9	0.032748343	0.789310457
RTN1	-1.394075094	0.005020283
RTN2	-0.730494974	0.005391307
RTN3	-0.752103028	0.056733504
RTN4	-0.724464399	0.000223407
RTN4IP1	0.069824001	0.779159502
RTN4R	-0.330009729	0.054969202
RTN4RL1	-1.135898431	0.007623782
RTN4RL2	-0.725310244	0.009105074
RTP1	-0.157131878	0.220134489
RTP2	-0.042419887	0.605512659
RTP3	-0.077356461	0.509043396
RTP4	0.630232506	0.001392079
RTP5	0.07077024	0.855799407
RTRAF	0.027093859	0.936643326

RTTN	0.846932732	0.022766171
RUBCNL	1.398350218	0.008424217
RUFY2	-0.587698729	0.03296851
RUNDC1	-0.257796167	0.06755688
RUNDC3B	-0.772772171	0.010505691
RUNX2	0.332207111	0.610213815
RUVBL2	-0.043191015	0.883869703
RWDD2A	-0.173085124	0.606390156
RWDD2B	-0.188029183	0.543983376
RWDD4	0.652566249	0.011440642
RXFP1	-1.397885282	0.005610423
RXFP2	-0.119331914	0.082754206
RXFP3	-0.270240038	0.076360903
RXFP4	-0.137479137	0.445186396
RXRA	0.331556339	0.139277883
RXRG	-0.829957093	0.008092314
RXYLT1	0.150748348	0.500185066
RYBP	-0.128607152	0.731977162
RYR1	-0.04398687	0.918837703
RYR2	-2.885944933	0.001834847
S100A1	-0.938293777	0.001776331
S100A10	0.989850642	0.027957226
S100A11	0.5574767	0.138100719
S100A12	-0.038694077	0.810771254
S100A14	0.001027218	0.993317406
S100A16	0.251139634	0.578328949
S100A2	0.273590959	0.045979268
S100A3	0.138577678	0.469599376
S100A5	-0.073407618	0.517738935
S100A6	0.323165318	0.183400098
S100A7	-0.06419764	0.673856736
S100A7A	-0.04497886	0.743883616
S100A8	0.561790051	0.113317425
S100A9	0.710181385	0.057226797
S100B	0.827425132	0.326932275
S100G	-0.146677748	0.086496592
S100P	-0.198278481	0.098505926
S100Z	0.18230579	0.125489093
S1PR1	-0.537948213	0.462801577
S1PR2	1.019855672	0.00924652
S1PR3	0.59466894	0.031906402
S1PR4	-0.177707912	0.227110312
S1PR5	-0.774815686	0.002977765
SAA1	0.340334717	0.566717857
SAAL1	0.273275282	0.093135402
SAC3D1	0.127711576	0.305389973
SACM1L	0.164819292	0.338352185
SACS	-0.114866122	0.761697772

SAG	-0.0671138	0.590748714
SAGE1	-0.139880936	0.241263532
SALL1	1.077812823	0.026224574
SALL3	0.974579445	0.036451367
SALL4	0.282184038	0.144719236
SAMD1	0.170625082	0.461374489
SAMD10	0.250586608	0.293846746
SAMD11	-0.053543527	0.821198512
SAMD12	-1.471383358	0.00036919
SAMD14	-0.317195063	0.127006537
SAMD15	0.431306727	0.093135402
SAMD4A	0.800070668	0.005846428
SAMD5	0.07738681	0.732437478
SAMD7	-0.045816848	0.681447551
SAMD8	-0.156932922	0.515150095
SAMD9	1.809797158	0.000271506
SAMD9L	2.334826811	4.88E-05
SAMHD1	0.761310762	0.023850462
SAMM50	-0.336324684	0.161711365
SAP18	-0.164410492	0.286830799
SAP30	0.704725924	0.003607731
SAPCD2	0.070119008	0.643609296
SAR1B	0.12822564	0.380722196
SARAF	-0.184362296	0.33838734
SARDH	0.014224291	0.928511726
SARM1	0.331801215	0.111348105
SART3	0.193022916	0.139350979
SASH1	-0.443148355	0.304312885
SASH3	0.891841605	0.00199451
SASS6	0.776284512	0.010127765
SATB2	-0.631458494	0.089748678
SATL1	0.107188371	0.673702005
SAV1	0.032601153	0.90255532
SAXO1	-0.159216626	0.223714901
SAYSD1	0.331824095	0.015009402
SBDS	-0.032010257	0.915980695
SBF1	-0.396127991	0.110874003
SBF2	0.181023674	0.181285137
SBSPON	0.325356936	0.656755885
SC5D	-0.57131187	0.087888582
SCAF1	-0.436525355	0.186566312
SCAF4	0.305893449	0.040232811
SCAF8	0.015117343	0.948140379
SCAI	-1.001423966	0.004908431
SCAMP1	-0.47192044	0.037717269
SCAMP2	0.305958245	0.234889728
SCAMP3	0.337566515	0.031787603
SCAND1	-0.059894275	0.625121753

SCAP	-0.011450326	0.957353117
SCAPER	-0.201440868	0.531043314
SCARA3	0.437585448	0.277203467
SCARA5	-0.093761646	0.496399767
SCARB1	0.040832981	0.870609869
SCARB2	0.244609447	0.218459979
SCARF2	-0.111356243	0.658430807
SCCPDH	0.063842607	0.797120422
SCEL	-0.155647859	0.057102086
SCFD2	1.024927202	0.040284267
SCG2	0.425706236	0.465591931
SCG3	-1.354376606	0.293451908
SCGB1A1	0.022590085	0.862447143
SCGB1C1	-0.010715954	0.941983489
SCGB1D1	-0.141874793	0.204130967
SCGB1D2	-0.039205203	0.841759022
SCGB1D4	-0.018354688	0.898032902
SCGB2A1	0.003510089	0.976193867
SCGB2A2	-0.084097891	0.340627648
SCGB3A1	-0.119401975	0.431642249
SCGB3A2	-0.072004595	0.731401308
SCGN	0.162978849	0.746313198
SCIMP	1.108598203	0.007327295
SCLT1	0.037288305	0.902943459
SCML2	0.430164017	0.068021799
SCML4	0.007028296	0.96051513
SCN10A	0.00097639	0.994668339
SCN1A	0.609628381	0.369727841
SCN1B	-1.503704837	0.001225213
SCN2A	-1.431255402	0.08157781
SCN2B	-1.996301879	0.003607731
SCN3A	0.659568136	0.539728912
SCN3B	-1.67317343	0.05014765
SCN4A	0.05079072	0.713485047
SCN4B	-0.441343779	0.094240425
SCN8A	-1.071678475	0.06009886
SCN9A	-0.142060087	0.841183224
SCNN1G	0.380922475	0.389862618
SCO1	0.207808536	0.320347182
SCP2	0.57208382	0.004768244
SCP2D1	-0.008241583	0.941091514
SCPEP1	0.64749504	0.036778412
SCRG1	0.232942954	0.172372462
SCRIB	0.13255483	0.564532083
SCRN3	0.085175324	0.698224223
SCRT1	-1.230310023	0.000815172
SCRT2	-0.269354764	0.156379487
SCT	-0.031068713	0.825257297

SCTR	-0.304164905	0.166149474
SCUBE1	-0.163030828	0.281159119
SCUBE2	0.38621176	0.059929986
SCUBE3	-0.612809291	0.31931821
SCYL2	0.282194295	0.379871619
SCYL3	0.516889954	0.001149566
SDAD1	0.0965781	0.670085809
SDC2	-0.183961153	0.735925036
SDC4	-2.035500405	0.001954005
SDCBP2	-0.014470622	0.953304329
SDCCAG8	0.221234603	0.395686368
SDE2	0.321130266	0.173390271
SDF2	0.236208416	0.355317656
SDF2L1	0.045635617	0.918837703
SDHAF1	0.037573151	0.854103995
SDHAF2	0.243195611	0.172093322
SDHAF3	0.502908669	0.029741798
SDHAF4	0.295286544	0.100754919
SDHB	-0.068112081	0.695912218
SDHD	0.196105731	0.078955881
SDK1	0.212345523	0.510565481
SDR16C5	-0.269814327	0.076256414
SDR42E1	-0.043535716	0.742071029
SDR9C7	-0.14573681	0.390626215
SDS	-0.265713485	0.242699986
SDSL	0.282620822	0.16184892
SEC11A	0.934053841	0.003568303
SEC11C	0.285741983	0.435309414
SEC14L4	0.11497316	0.473389208
SEC14L6	0.15305114	0.515958709
SEC16B	0.189958412	0.390778383
SEC22A	0.298471974	0.05383815
SEC22B	0.189862716	0.482342112
SEC22C	0.066658326	0.594436667
SEC23A	-0.264718255	0.301839697
SEC23B	0.339758116	0.138969461
SEC23IP	-0.052057909	0.851362495
SEC24B	0.085220643	0.654934037
SEC24D	0.341175226	0.537165089
SEC31A	0.458302215	0.021290241
SEC31B	0.002227472	0.995730111
SEC61A1	0.873419939	0.027593953
SEC61B	0.456457537	0.200766737
SEC61G	1.67633034	0.036591578
SEC62	-0.406114053	0.060109318
SEC63	0.291687982	0.134776399
SECISBP2	0.543291907	0.013587776
SECTM1	0.4114312	0.226735959

SEH1L	-0.356449275	0.188151106
SEL1L	-0.02843371	0.920838877
SEL1L3	-0.013246278	0.989997727
SELE	-0.029213301	0.845809763
SELENOF	0.012100446	0.956557169
SELENOH	0.237250999	0.269249476
SELENOI	-0.161975432	0.617046773
SELENOK	-0.189456498	0.188151106
SELENOM	-0.690885086	0.001056288
SELENON	0.766372941	0.027370254
SELENOO	-0.015639786	0.953657114
SELENOS	0.305820003	0.16500399
SELENOT	-0.160128839	0.409857307
SELENOV	0.12906531	0.47831484
SELENOW	-1.140513223	0.001038868
SEM1	0.704900932	0.000613602
SEMA3A	-0.283723693	0.737023599
SEMA3B	-0.493320338	0.356227857
SEMA3C	-0.8559224	0.285032637
SEMA3D	-0.25416562	0.597800651
SEMA3F	-0.139134167	0.628067677
SEMA3G	-0.285618816	0.016845974
SEMA4A	-0.53794415	0.028516222
SEMA4B	-0.090606311	0.629181947
SEMA4C	0.167429488	0.563836061
SEMA4F	-0.870732168	0.003270886
SEMA4G	-0.321114246	0.156254002
SEMA5A	1.639217213	0.000393501
SEMA6A	0.740472324	0.070790493
SEMA6B	-0.937023424	0.057699866
SEMA7A	-1.202007025	0.003626754
SEMG1	-0.016350598	0.89481295
SEMG2	-0.124730323	0.473389208
SENP2	-0.277972942	0.070097516
SENP5	0.011356006	0.966247194
SENP6	0.161689171	0.345902213
SENP7	0.908318924	0.001005364
SEPHS1	0.165753505	0.537165089
SEPHS2	-0.053860227	0.843863404
SEPSECS	0.750965456	0.001392079
SEPTIN1	-0.00339895	0.98286235
SEPTIN10	0.613184848	0.05613251
SEPTIN11	-0.434563884	0.117175706
SEPTIN12	-0.081300893	0.553281412
SEPTIN14	-0.164598126	0.136247412
SEPTIN6	-0.070806987	0.787690301
SEPTIN7	0.548241812	0.139745462
SEPTIN8	-0.283647364	0.359128631

SERAC1	0.091010504	0.780287176
SERF1A	0.021808569	0.907751217
SERGEF	-0.051317312	0.843477662
SERHL2	-0.068731571	0.600991795
SERINC3	-0.665213776	0.021414705
SERP1	0.136021404	0.405162479
SERPINA1	0.594906252	0.022434883
SERPINA10	-0.218283331	0.061498739
SERPINA12	-0.00841759	0.950396272
SERPINA3	0.313092716	0.103580058
SERPINA4	-0.145678337	0.194183662
SERPINA5	-0.067536745	0.462356333
SERPINA6	0.068976375	0.606165035
SERPINA7	0.055010121	0.577168524
SERPINA9	-0.57053415	0.167822896
SERPINB1	0.272915469	0.353410067
SERPINB10	-0.126398645	0.11710374
SERPINB11	-0.120855271	0.171793831
SERPINB12	-0.164549618	0.055634097
SERPINB13	-0.182448896	0.019622827
SERPINB3	-0.211221981	0.124427682
SERPINB5	-0.014296721	0.888106292
SERPINB7	-0.604651768	0.187031577
SERPINB9	-0.056838372	0.913028318
SERPIND1	-0.194724321	0.298598061
SERPINE1	0.958119579	0.476733294
SERPINE2	-0.578590176	0.160246628
SERPINF1	0.090497894	0.93583597
SERPING1	1.368729538	0.020640868
SERPINH1	1.074478593	0.147353898
SERPINI1	-2.605350184	0.013851792
SERPINI2	1.192842628	0.013618101
SERTAD1	-0.083599976	0.816408453
SERTAD3	0.110537864	0.584951716
SERTAD4	-0.636070424	0.003261714
SERTM1	-1.000795344	0.016082718
SESN1	-0.170074468	0.443670516
SESN2	0.101250991	0.802576875
SESN3	-0.215329424	0.589239821
SESTD1	-0.254702726	0.476374594
SETBP1	0.711072801	0.024278564
SETD1B	0.056289864	0.855799407
SETD2	0.339562551	0.019689342
SETD3	-0.503339402	0.010124983
SETD4	0.343709742	0.006185744
SETD7	0.044839914	0.860472196
SETD9	-0.002256877	0.99128072
SETDB2	-0.111044029	0.649190814

SETMAR	0.416547789	0.047203268
SETX	0.491687469	0.003603178
SEZ6	0.105059095	0.871201192
SEZ6L	-0.597345083	0.503473628
SF1	0.609165755	0.002733133
SF3A1	0.059212174	0.747102301
SF3A3	0.708147204	0.004565843
SF3B1	0.29352215	0.054474658
SF3B3	0.446263937	0.098337798
SF3B4	0.35482649	0.334108571
SF3B5	0.244819458	0.380147927
SF3B6	0.775334729	0.020937754
SFI1	0.262514362	0.150068851
SFMBT1	0.525196267	0.005965654
SFN	0.038456335	0.855935923
SFR1	-0.122263	0.568985288
SFRP1	-0.806981204	0.107375492
SFRP2	0.0714315	0.947248727
SFRP4	0.952657229	0.025834777
SFRP5	-0.235193805	0.120199333
SFT2D1	0.15268284	0.626696492
SFT2D2	0.792488277	0.016739189
SFTA2	0.041396924	0.726285678
SFTA3	0.078244373	0.310496927
SFTPB	-0.117089301	0.281715857
SFTPD	-0.048045759	0.731357875
SFXN1	0.190947767	0.225667568
SFXN2	0.435846104	0.024037243
SFXN3	-0.30502414	0.235197653
SFXN4	-0.117751203	0.62772996
SFXN5	-0.32962933	0.317017888
SGCA	0.13893818	0.45271852
SGCB	0.645253362	0.066518126
SGCD	-0.696416369	0.026567115
SGCZ	-0.275739281	0.322585459
SGF29	0.391218965	0.15119423
SGIP1	-0.734657839	0.164294375
SGK2	-0.297675972	0.251929013
SGMS1	-0.747295853	0.042421405
SGMS2	-0.025322955	0.948903933
SGO2	0.291761393	0.09769566
SGPL1	0.122749462	0.761697772
SGPP1	0.027891125	0.941168506
SGPP2	-0.509893753	0.105918599
SGSH	0.510896499	0.065639456
SGSM2	-0.18096615	0.606700784
SGSM3	-0.266433428	0.100093789
SGTA	0.212821829	0.277210723

SGTB	-1.178792848	0.002621142
SH2B1	0.326327751	0.245528041
SH2B2	0.394447116	0.051200198
SH2B3	-0.006928133	0.982318638
SH2D1A	-0.00478587	0.968926234
SH2D1B	-0.316723124	0.008448974
SH2D3A	-0.039130748	0.772269568
SH2D3C	-0.246492018	0.260870097
SH2D4B	-0.156933751	0.201748507
SH2D6	0.164616039	0.234240052
SH2D7	-0.171769812	0.311487033
SH3BGR	0.608374019	0.035400588
SH3BGRL	0.069881643	0.712803392
SH3BGRL2	-1.396783457	0.00501668
SH3BGRL3	-0.081100731	0.828000658
SH3BP1	-1.461023106	0.001056288
SH3BP2	0.621114097	0.025920214
SH3BP4	-0.296855408	0.443186348
SH3BP5	-0.795467925	0.00126644
SH3BP5L	-0.116322988	0.587262655
SH3D21	0.1804406	0.731977162
SH3GL2	-1.85621429	0.015991691
SH3GL3	-2.304402821	0.000903692
SH3GLB2	-0.469131065	0.127500859
SH3KBP1	-0.027982625	0.920850924
SH3PXD2B	1.279438384	0.004149839
SH3RF1	-0.25022963	0.335608162
SH3RF2	-1.071779062	0.053467733
SH3TC1	0.221069759	0.327635999
SH3YL1	0.410339647	0.011984713
SHANK1	-2.509983104	0.000879799
SHANK2	-0.357178567	0.044310474
SHARPIN	0.108265299	0.472294521
SHBG	0.022449372	0.860472196
SHC1	0.4959463	0.481196182
SHC3	-1.341610272	0.017336888
SHC4	0.137998531	0.756015246
SHCBP1	0.521479657	0.144125938
SHCBP1L	-0.025682823	0.79266965
SHD	0.363364666	0.523371606
SHF	-0.133268075	0.402631506
SHFL	0.236000031	0.441249384
SHH	-0.074375994	0.697417494
SHISA4	-0.394313361	0.193603939
SHISA5	0.524257809	0.048227867
SHISA6	-0.405532451	0.525774605
SHISAL2A	0.071021595	0.653942911
SHKBP1	0.190232427	0.653255633

SHLD2	0.519497029	0.188151106
SHOC1	0.036431391	0.64013899
SHOC2	-0.703021813	0.005780236
SHOX	-0.073341736	0.430567574
SHOX2	1.178176131	0.029475105
SHQ1	0.044706228	0.802305314
SHROOM2	-0.051843586	0.827153531
SHROOM3	-0.025730892	0.95917592
SHROOM4	-0.079338051	0.751540124
SI	-0.027001524	0.744872906
SIAE	-0.228342278	0.239588827
SIAH2	0.1094313	0.567948214
SIAH3	-0.502468014	0.018227997
SIDT1	-0.787953756	0.01574763
SIDT2	-0.508901559	0.135653207
SIGLEC1	0.601959908	0.05679073
SIGLEC10	1.249741513	0.022459642
SIGLEC11	0.180467927	0.269096401
SIGLEC12	-0.036279535	0.808256838
SIGLEC15	-0.082577079	0.635198978
SIGLEC5	0.000615199	0.99830973
SIGLEC6	-0.26659022	0.022635497
SIGLEC7	0.266814861	0.152927916
SIGLEC8	0.545908738	0.213262526
SIGLEC9	0.704960431	0.033319952
SIGLECL1	0.025350522	0.862505915
SIGMAR1	-0.554035653	0.203243282
SIK1	0.494922786	0.277225602
SIK2	-0.301733777	0.09868809
SIK3	-0.189065041	0.638604363
SIM1	-0.255242471	0.034355668
SIM2	0.552885155	0.02736428
SIMC1	0.012403677	0.94671625
SIN3B	0.155001989	0.66490125
SINHCAF	-0.346138401	0.072419586
SIPA1	0.357591497	0.112320276
SIPA1L1	-0.03914457	0.936643326
SIPA1L2	0.976692788	0.00872419
SIRPB1	0.289467445	0.074269608
SIRPD	0.099856079	0.46925675
SIRPG	-0.118312352	0.423445426
SIRT1	0.001309054	0.996803176
SIRT3	0.206236702	0.302520363
SIRT4	0.036418742	0.736392111
SIRT5	-0.455992258	0.082058452
SIRT6	0.006831488	0.979996214
SIVA1	0.184563328	0.756211506
SIX1	0.140517912	0.614816249

SIX2	-0.000220233	0.998986205
SIX3	-0.111025281	0.451421649
SIX4	-0.161696833	0.583914788
SIX5	0.058951663	0.776438413
SIX6	0.113668484	0.7527455
SKA3	0.780721914	0.132066167
SKAP1	0.005454518	0.965294214
SKI	0.39265028	0.11600824
SKIDA1	-0.005533711	0.981876206
SKIL	0.218501664	0.251718718
SKP1	0.355321482	0.067155561
SKP2	0.525523159	0.198182188
SLA2	0.115621119	0.471673292
SLAMF1	-0.17591752	0.133828328
SLAMF6	0.133945216	0.298658542
SLAMF7	-0.256384739	0.595404685
SLAMF8	0.903124488	0.011068079
SLAMF9	0.020432955	0.912618675
SLBP	0.238267851	0.336009555
SLC10A1	-0.050764437	0.670737227
SLC10A2	0.019622578	0.901235662
SLC10A3	-0.303447982	0.181766132
SLC10A4	0.581072707	0.117748464
SLC10A5	-0.216269046	0.420941497
SLC10A6	0.191270755	0.111086676
SLC11A1	2.04361194	0.002329559
SLC11A2	0.425856851	0.021546998
SLC12A3	0.039540233	0.778517241
SLC12A6	-0.046995762	0.880038975
SLC12A7	0.588743489	0.0097843
SLC13A1	-0.034190246	0.72399071
SLC13A4	-0.133231342	0.735051879
SLC13A5	-0.900924911	0.298120652
SLC15A1	-0.194330258	0.148833608
SLC15A2	0.463680756	0.48715344
SLC15A3	0.628283762	0.004981702
SLC15A4	0.582392606	0.010173577
SLC16A1	0.190955775	0.409271249
SLC16A10	0.137519929	0.695795543
SLC16A11	-0.034536143	0.822013734
SLC16A12	-0.12682559	0.608118412
SLC16A13	0.07889665	0.615344143
SLC16A14	-0.738007505	0.030129121
SLC16A2	-0.108363971	0.779159502
SLC16A4	0.609970288	0.209686107
SLC16A5	0.118879827	0.237192365
SLC16A7	-0.290926921	0.267530519
SLC16A8	-0.16437364	0.09845529

SLC16A9	-0.004118429	0.992886227
SLC17A2	-0.147698134	0.121554917
SLC17A3	0.087692126	0.252819548
SLC17A4	-0.092239955	0.249098781
SLC17A5	-0.002729043	0.992886227
SLC17A6	-0.536815198	0.044567499
SLC17A7	-3.538624184	0.003274587
SLC17A8	-0.23274855	0.093612208
SLC17A9	-0.027452093	0.923616918
SLC18A2	-0.138035362	0.152144847
SLC18A3	0.013049565	0.939547287
SLC18B1	-0.00999865	0.985639169
SLC19A1	-0.080322572	0.517739911
SLC19A2	0.451629835	0.075993917
SLC19A3	0.277089634	0.301306841
SLC1A1	-0.56766812	0.318175113
SLC1A3	0.602110391	0.419236188
SLC1A4	-0.986022293	0.017706015
SLC1A6	-0.30259945	0.129133169
SLC1A7	-0.040262049	0.7527455
SLC20A1	0.248513629	0.583881916
SLC22A1	-0.113676892	0.511706372
SLC22A11	-0.043801291	0.796644258
SLC22A12	-0.144850131	0.281291951
SLC22A13	-0.093608164	0.555003869
SLC22A14	-0.16676707	0.124389428
SLC22A15	-0.781437707	0.038596937
SLC22A16	-0.057226128	0.519650217
SLC22A17	-0.563251934	0.278333668
SLC22A18	0.142171533	0.510940875
SLC22A2	-0.01850409	0.853715871
SLC22A25	-0.099602342	0.6062359
SLC22A3	0.04514989	0.845746964
SLC22A5	-0.198122934	0.396542579
SLC22A6	0.033719931	0.768066204
SLC22A7	-0.156031373	0.169004215
SLC22A8	-0.003358293	0.983832633
SLC22A9	-0.515750929	0.016780753
SLC23A1	-0.171744922	0.267906543
SLC23A2	-0.061594422	0.851610088
SLC24A1	0.332919307	0.030697987
SLC24A2	-2.517929923	0.022905522
SLC24A3	0.832931853	0.141950937
SLC24A4	-0.118851553	0.779777053
SLC24A5	-0.089153623	0.391553303
SLC25A1	-0.139034041	0.704306918
SLC25A10	-0.001885307	0.994849586
SLC25A11	-0.489046419	0.001678442

SLC25A14	-0.085674566	0.680038384
SLC25A15	0.173935962	0.546004746
SLC25A16	-0.401452658	0.051344603
SLC25A17	0.361926138	0.092158618
SLC25A2	-0.001845119	0.994849586
SLC25A20	-0.003854795	0.992328935
SLC25A23	-1.600340477	0.001072162
SLC25A24	0.277071596	0.564973097
SLC25A26	-0.21424696	0.191493024
SLC25A27	0.173460707	0.79460985
SLC25A28	-0.021272767	0.912632913
SLC25A3	-0.115057717	0.562821065
SLC25A31	-0.181197309	0.135013087
SLC25A32	0.381215418	0.153950852
SLC25A33	-0.253324609	0.547868374
SLC25A34	-0.3269071	0.165320955
SLC25A35	0.314117509	0.090942013
SLC25A37	0.682085883	0.041817707
SLC25A38	-0.052699524	0.777042628
SLC25A4	-1.016840506	0.015845112
SLC25A40	0.44448655	0.112969337
SLC25A41	-0.060909108	0.639674012
SLC25A43	0.468661424	0.182057768
SLC25A44	-0.467488038	0.054020991
SLC25A46	-0.285494737	0.067223112
SLC25A47	-0.34933159	0.059425494
SLC25A48	-0.553496287	0.047728123
SLC25A5	-0.06438645	0.736640271
SLC25A6	-0.40126099	0.098350432
SLC26A1	0.025485272	0.871201192
SLC26A2	0.393537962	0.180879322
SLC26A3	-0.022937764	0.87141719
SLC26A4	-0.381358705	0.108325357
SLC26A5	-0.09178335	0.259318019
SLC26A7	-0.145350118	0.092781756
SLC26A8	-0.299240373	0.100710754
SLC26A9	-0.278363227	0.008460059
SLC27A1	0.067148428	0.852711266
SLC27A2	-0.399900877	0.064315566
SLC27A3	0.629731065	0.042998566
SLC27A4	-0.27140374	0.106698418
SLC27A5	-0.475708441	0.002901092
SLC27A6	0.200489438	0.503449371
SLC28A1	-0.003925842	0.985187657
SLC28A2	0.050596757	0.634130013
SLC29A2	0.319423431	0.53598362
SLC29A3	0.092125255	0.839668933
SLC2A1	-0.494087355	0.117089519

SLC2A10	1.034840971	0.021290241
SLC2A12	-0.828329433	0.061700422
SLC2A13	-0.006333808	0.993945285
SLC2A14	0.998318927	0.120440236
SLC2A2	-0.026336661	0.871201192
SLC2A4	-0.117607253	0.459119552
SLC2A5	0.431296473	0.226896023
SLC2A6	-0.58935083	0.007389617
SLC2A7	0.071850467	0.478648665
SLC2A8	-0.247886506	0.035197777
SLC2A9	0.121235768	0.331915484
SLC30A1	0.02421971	0.935244061
SLC30A2	-0.057377551	0.575632215
SLC30A3	-1.016572418	0.002245834
SLC30A4	-0.429849409	0.104500218
SLC30A5	0.29656372	0.268131236
SLC30A7	0.245739222	0.354066677
SLC30A9	-0.190170925	0.459765932
SLC31A1	0.14540659	0.620075563
SLC31A2	-0.588140925	0.163293362
SLC32A1	-0.353737042	0.110537981
SLC33A1	0.47565832	0.044369981
SLC34A1	0.095881254	0.489607619
SLC35A1	-0.052952111	0.845168366
SLC35A2	0.129576056	0.469680937
SLC35A3	0.245361106	0.407219221
SLC35A4	-0.075836819	0.853875788
SLC35A5	0.176940177	0.487841801
SLC35B1	0.000546073	0.99830973
SLC35B2	0.104053099	0.78413419
SLC35B3	0.352040306	0.102381232
SLC35B4	0.475765279	0.041961227
SLC35C1	0.063258613	0.861298403
SLC35C2	-0.014718531	0.927473249
SLC35D1	0.586475666	0.080148455
SLC35D2	0.963404817	0.010927461
SLC35D3	-0.104357841	0.342189794
SLC35E1	0.197940509	0.307012392
SLC35E3	0.273531278	0.052010099
SLC35E4	-0.275096329	0.034713194
SLC35F3	-1.225994097	0.00041574
SLC35F5	0.534470237	0.024305591
SLC35F6	-0.158500481	0.453928716
SLC35G1	0.073985246	0.514190889
SLC35G2	0.310199183	0.080719531
SLC35G3	-0.136296065	0.223143157
SLC35G5	0.194621797	0.388913223
SLC36A1	-0.180151494	0.332875404

SLC36A4	0.392901016	0.02140583
SLC37A1	-0.232317068	0.486990502
SLC37A2	0.441853466	0.025115037
SLC37A3	0.102297156	0.697525606
SLC38A1	-1.094418949	0.131139525
SLC38A11	0.149054018	0.776484731
SLC38A3	0.331778655	0.574759224
SLC38A4	-0.156836553	0.129383683
SLC38A5	0.856210671	0.005610423
SLC38A6	0.499313466	0.013311097
SLC38A7	-0.348009324	0.101738623
SLC38A9	0.146573734	0.391984317
SLC39A1	0.341332776	0.064680316
SLC39A2	-0.00114091	0.993903659
SLC39A3	-0.072771222	0.733411121
SLC39A4	-0.149135434	0.401984916
SLC39A5	0.021638134	0.862505915
SLC39A6	-0.026145982	0.96148544
SLC39A9	-0.017034565	0.946720667
SLC3A1	-0.197626032	0.052433755
SLC40A1	0.647537387	0.010398332
SLC41A1	-0.13030194	0.745484819
SLC41A2	-0.232211452	0.528168359
SLC41A3	-0.313620053	0.068367319
SLC43A1	0.300800299	0.072267723
SLC43A2	0.278574837	0.365150025
SLC44A1	-0.10302301	0.881098535
SLC44A2	-0.214075209	0.402740226
SLC44A4	-0.125673783	0.203901234
SLC44A5	0.708064093	0.250506267
SLC45A1	-0.339070302	0.06041
SLC45A2	0.132879128	0.289558045
SLC45A3	-0.441259432	0.123229751
SLC46A1	0.112545947	0.719087761
SLC46A2	-0.132682596	0.2168835
SLC46A3	0.473892739	0.149513034
SLC47A1	0.01968615	0.95239805
SLC47A2	0.992947397	0.023949051
SLC48A1	-0.835683403	0.014012467
SLC49A3	0.241815373	0.235131833
SLC49A4	-0.146715431	0.655935013
SLC4A1	-0.296304191	0.013239936
SLC4A11	0.421903137	0.339412764
SLC4A1AP	-0.195937996	0.231113261
SLC4A3	-0.009690745	0.984794134
SLC4A5	-0.684333766	0.000635455
SLC4A7	0.265540885	0.549835967
SLC51A	-0.164910413	0.327635999

SLC51B	0.012519455	0.93721758
SLC52A2	0.158421536	0.399038792
SLC52A3	-0.136189282	0.501409704
SLC5A1	0.009299294	0.931727556
SLC5A10	-0.133420483	0.154053453
SLC5A11	-1.386191617	0.012583552
SLC5A12	-0.127664121	0.372787239
SLC5A2	-0.34907612	0.007425085
SLC5A3	0.185566921	0.49846128
SLC5A4	0.048368553	0.749327914
SLC5A5	-0.12820355	0.243875421
SLC5A7	-0.054540607	0.623980543
SLC5A8	-0.242141787	0.031489177
SLC66A1	0.002892988	0.991101527
SLC66A2	-0.059004633	0.795891666
SLC66A3	0.687678947	0.100493531
SLC6A1	-0.545347471	0.601588011
SLC6A11	-0.71454234	0.383128678
SLC6A12	-0.36689589	0.077145205
SLC6A13	-0.072620303	0.749573888
SLC6A14	0.072157955	0.372787239
SLC6A15	-1.426892952	0.015970768
SLC6A16	0.182102043	0.503761344
SLC6A17	-2.683369895	0.002419881
SLC6A18	-0.128438835	0.323792532
SLC6A19	-0.196217528	0.200551216
SLC6A20	0.059179245	0.780486192
SLC6A3	-0.190919136	0.124819142
SLC6A4	-0.099223164	0.274554303
SLC6A5	-0.0817884	0.463668515
SLC6A6	-0.201373993	0.646560301
SLC6A7	-1.657996397	0.005081616
SLC6A8	-0.098893814	0.746879258
SLC6A9	0.651075655	0.159337592
SLC7A1	-0.376872555	0.257553312
SLC7A10	-0.699085729	0.14496164
SLC7A11	-0.445217667	0.201343599
SLC7A13	-0.11359304	0.31754923
SLC7A2	-0.340070433	0.513684677
SLC7A3	-0.107414096	0.689387141
SLC7A4	-0.177202999	0.168755601
SLC7A6OS	0.31340613	0.085483631
SLC7A9	0.079097247	0.543574831
SLC8A1	-0.430322463	0.167613921
SLC8A3	-0.313502673	0.405754532
SLC8B1	0.438008042	0.009183894
SLC9A1	0.016191349	0.959862565
SLC9A2	-0.149226873	0.250939062

SLC9A3	0.032084412	0.816084385
SLC9A3R1	-1.011509491	0.011867165
SLC9A5	-0.299626626	0.239584751
SLC9A9	0.681456138	0.08399916
SLC9B1	-0.253671822	0.425184154
SLC9C1	-0.217609217	0.042976977
SLC9C2	0.369540878	0.316898088
SLCO1A2	-1.636812281	0.004305912
SLCO1B1	0.123962016	0.480653171
SLCO1B3	-0.074775966	0.504434311
SLCO2A1	0.350938125	0.200551216
SLCO2B1	0.813237365	0.114071721
SLCO3A1	-0.885313557	0.065878376
SLCO4A1	-0.359962816	0.131374634
SLCO4C1	-0.514774524	0.028347752
SLCO5A1	0.117536363	0.721502294
SLCO6A1	-0.001414072	0.991232488
SLF1	0.152305093	0.616944401
SLF2	0.222746272	0.364268819
SLFN12	0.131192573	0.758441099
SLFN12L	-0.011401839	0.938588678
SLFN13	0.452185472	0.158534697
SLFN5	0.710555363	0.086538792
SLFNL1	-0.195723771	0.234695411
SLIT1	-0.562091455	0.263326427
SLIT2	-1.574855045	0.002326657
SLIT3	-1.061266464	0.038709224
SLITRK1	-1.341282206	0.152254709
SLITRK2	-0.032803306	0.96148544
SLITRK3	0.122962181	0.846242672
SLITRK5	-0.491118321	0.291256785
SLITRK6	-0.263041092	0.033651918
SLK	-0.441220184	0.036441711
SLMAP	-0.142753363	0.334512578
SLN	1.171374397	0.066664283
SLPI	0.89831495	0.141930369
SLTM	0.313993485	0.070220293
SLU7	-0.398789452	0.07653211
SLURP1	-0.072759486	0.600343054
SLX4	0.195368199	0.0941179
SMAD1	0.425174768	0.068186904
SMAD2	0.350080401	0.019820304
SMAD4	0.277137176	0.168755601
SMAD5	0.460268153	0.063240636
SMAD7	-0.103239007	0.791633255
SMAGP	0.698302013	0.000331368
SMAP2	-0.209496057	0.562420326
SMARCA1	0.085483057	0.695912218

SMARCA2	-0.286859912	0.391833864
SMARCA5	0.111214653	0.654062386
SMARCAL1	0.357042268	0.049245411
SMARCB1	0.121767253	0.520931342
SMARCD1	0.444420446	0.031618823
SMARCD3	0.215445829	0.361795688
SMARCE1	0.516968196	0.011740569
SMC1A	0.709995146	0.015826873
SMC1B	0.005023616	0.981610177
SMC3	-0.087383376	0.789310457
SMC4	1.652134438	0.002566687
SMC5	1.245512714	0.001745335
SMCO1	-0.171808717	0.077022752
SMCO2	0.049733749	0.658552671
SMCO3	0.065984086	0.597443209
SMCO4	0.090639889	0.335921879
SMCP	0.08607703	0.471442677
SMCR8	0.266495905	0.181962299
SMDT1	-0.570302198	0.03700233
SMG1	0.391236658	0.012866514
SMG5	0.566098627	0.012204489
SMG7	0.314805926	0.015826873
SMG8	-0.16234659	0.305375238
SMG9	0.358126595	0.142116362
SMIM1	-0.084678744	0.410558742
SMIM11A	0.123324435	0.515432943
SMIM13	-0.390989824	0.159229048
SMIM14	0.026364159	0.899536861
SMIM15	0.578085955	0.005239256
SMIM19	0.041329772	0.884394993
SMIM2	-0.142952482	0.18665382
SMIM21	-0.088829009	0.434340515
SMIM24	-0.25171414	0.227717638
SMIM26	0.25747459	0.319706075
SMIM29	-0.067757666	0.653942911
SMIM3	0.671154457	0.029665053
SMIM34A	-0.096044045	0.449235883
SMIM4	0.337637279	0.227889709
SMIM7	0.469819613	0.001980282
SMIM8	0.413523037	0.179519907
SMKR1	-0.012463111	0.957381217
SMN1	0.446692774	0.007998856
SMNDC1	-0.168970712	0.516729658
SMO	0.942698094	0.011984713
SMOC2	0.144922209	0.481412872
SMOX	0.728735373	0.054240566
SMPD1	-0.69025044	0.025145888
SMPD2	0.389962629	0.056043915

SMPD3	-0.793062024	0.001541164
SMPDL3A	0.557302726	0.054014736
SMPDL3B	-0.056961935	0.581110545
SMR3A	-0.01659522	0.935244061
SMR3B	-0.021430161	0.882524574
SMS	0.01250179	0.981452955
SMUG1	0.215795273	0.371103788
SMURF1	0.261375461	0.209059496
SMYD1	-0.065100207	0.560642078
SMYD2	-0.143788225	0.709670769
SMYD5	0.021874537	0.911053286
SNAI2	0.803658004	0.412532067
SNAP23	0.386923248	0.222845679
SNAP25	-2.765740154	0.019500011
SNAP29	-0.384922915	0.039536351
SNAP47	-0.15870963	0.245299006
SNAPC1	0.526396337	0.126004782
SNAPC2	0.332103166	0.168721983
SNAPC3	-0.151928176	0.465188581
SNAPC4	0.144050154	0.462515109
SNAPC5	0.073840806	0.731136744
SNAPIN	0.200474626	0.362632921
SNCA	-1.59274342	0.010857404
SNCAIP	0.78941159	0.115870812
SNCB	-1.205285604	0.015388688
SNED1	-0.101410678	0.819641909
SNF8	0.19923884	0.185469519
SNIP1	0.332254886	0.054716207
SNN	-0.208528494	0.663890076
SNORC	-0.245831294	0.049821649
SNPH	-1.598818024	0.003206188
SNRK	0.249392794	0.115683548
SNRNP200	0.661755181	0.000311823
SNRNP25	-0.24289038	0.116756442
SNRNP27	-0.086448213	0.731357875
SNRNP35	0.152547196	0.23210837
SNRNP40	0.474408201	0.028347752
SNRNP48	0.550467794	0.014349686
SNRNP70	0.253991964	0.597100313
SNRPA	0.634663449	0.029037894
SNRPA1	0.360401271	0.04843526
SNRPB2	0.424532077	0.076351364
SNRPD1	0.149504518	0.6062359
SNRPD2	-0.174143812	0.506397029
SNRPD3	0.115689497	0.714486809
SNRPE	0.334496854	0.025846165
SNRPF	0.343036601	0.110426436
SNRPG	0.001508996	0.992505943

SNRPN	-1.171708828	0.044164454
SNTA1	-1.253569344	0.009546257
SNTB1	0.991780207	0.001620462
SNTG1	0.174730133	0.849399089
SNTG2	-0.170694925	0.253069122
SNTN	-0.153155908	0.073916903
SNU13	-0.227050437	0.148131173
SNUPN	-0.180248542	0.368114726
SNW1	-0.106030688	0.540894311
SNX11	0.032667505	0.862505915
SNX12	0.154670889	0.355969763
SNX13	0.610342829	0.003347402
SNX14	0.157426211	0.20690805
SNX16	0.299874425	0.11654049
SNX18	0.112452733	0.695574594
SNX19	-0.079039787	0.738527319
SNX2	-0.228522521	0.497470334
SNX20	0.337894492	0.058038312
SNX21	0.129875172	0.524326744
SNX22	-0.11474218	0.728011946
SNX24	0.170087268	0.313892119
SNX25	0.065154253	0.861682022
SNX27	-0.053622365	0.8481789
SNX3	0.486593654	0.008867317
SNX31	0.126876342	0.375010931
SNX32	-0.828875128	0.044859681
SNX33	0.544761175	0.067798973
SNX4	0.096771106	0.75649984
SNX5	0.438519631	0.009571203
SNX6	-0.217929132	0.419000593
SNX7	1.074540015	0.003569991
SNX8	0.041928566	0.865755743
SNX9	-0.386921746	0.261749631
SOAT1	0.941405691	0.006242017
SOAT2	0.085419917	0.45999477
SOBP	-0.160429532	0.699974153
SOCS1	0.190073839	0.3807326
SOCS2	0.232395776	0.566497648
SOCS3	1.404994769	0.047875946
SOCS4	0.246805039	0.344680288
SOCS5	-0.14999935	0.519850005
SOCS6	0.529871336	0.08658043
SOD1	-0.456375179	0.019659172
SOD3	-0.024713605	0.962306545
SOGA1	0.867860129	0.000962015
SON	0.753954217	0.003970808
SORBS1	0.449878008	0.435871216
SORBS2	-0.949928528	0.024432601

SORBS3	0.374232486	0.05367882
SORCS2	-0.997315824	0.024296673
SORCS3	0.811121875	0.209059496
SORL1	0.173725342	0.817881105
SOS1	0.219236359	0.243868912
SOST	-0.133303282	0.43704344
SOSTDC1	-0.251622788	0.223870308
SOWAHA	-0.648959152	0.016533545
SOWAHC	0.048676851	0.816408453
SOX1	0.068789677	0.760060564
SOX10	-0.413581291	0.435124766
SOX11	0.370996193	0.439383607
SOX12	0.200033846	0.299204961
SOX13	0.72491979	0.016372941
SOX14	0.053040762	0.686976208
SOX15	-0.178427866	0.274093403
SOX17	-0.100050744	0.385875334
SOX18	0.039102757	0.863152687
SOX2	1.400767014	0.026104691
SOX21	0.14112972	0.726469436
SOX3	0.046389999	0.91394031
SOX30	-0.154015951	0.3273107
SOX4	1.349068592	0.038574812
SOX5	0.309354667	0.324497184
SOX7	-0.209295425	0.151803036
SOX8	0.786192799	0.210412136
SOX9	0.9747586	0.039280377
SP1	0.918614855	0.000275651
SP100	0.911782007	0.038372241
SP110	1.346392697	0.000375895
SP3	0.784261477	0.000106517
SP4	0.664926881	0.011530566
SP5	0.268296628	0.022847707
SP7	0.173859455	0.240279812
SP8	0.251610988	0.625457691
SP9	0.062367478	0.688306212
SPA17	0.713280349	0.013757489
SPACA1	-0.052232514	0.601588011
SPACA3	-0.020897815	0.870363001
SPACA4	-0.225975079	0.151596061
SPACA5	-0.219601512	0.156921607
SPACA7	-0.137277208	0.11814796
SPACA9	0.443151785	0.007237767
SPAG1	0.694058716	0.00868089
SPAG11B	-0.140044255	0.483754557
SPAG17	0.448362288	0.202186223
SPAG4	-0.002995844	0.992505943
SPAG5	0.946627576	0.019550799

SPAG7	0.017467417	0.941983489
SPAG8	0.599451615	0.241364393
SPAM1	-0.358960313	0.017413144
SPANXA1	-0.48450358	0.014096806
SPANXC	-0.141909902	0.247399342
SPANXD	-0.287767289	0.123511899
SPARC	1.171458342	0.01413174
SPART	-0.150505088	0.502848118
SPAST	0.024765655	0.951240846
SPATA12	0.145631178	0.268371276
SPATA16	-0.135229097	0.115986257
SPATA17	0.801078494	0.017625882
SPATA18	-0.099337574	0.844747122
SPATA19	0.102784554	0.519914544
SPATA21	0.109270418	0.327370792
SPATA25	0.426319554	0.033434046
SPATA2L	-0.176753246	0.300109754
SPATA3	0.067425965	0.583914788
SPATA31A6	-0.096236767	0.483366908
SPATA31A7	-0.070604919	0.68290411
SPATA31D1	-0.242766559	0.086946952
SPATA31D3	-0.135801338	0.505280983
SPATA31E1	0.106938951	0.320594067
SPATA32	-0.009591463	0.93340442
SPATA4	-0.108585222	0.435871216
SPATA45	0.040459742	0.720933419
SPATA46	-0.027954637	0.861019362
SPATA48	-0.096169281	0.245605733
SPATA5	0.710070348	0.011930446
SPATA5L1	0.341733584	0.155127393
SPATA6	1.442171578	0.009663141
SPATA7	0.097792275	0.687005925
SPATA9	0.05459172	0.557596975
SPATC1	-0.065217983	0.736257563
SPATS1	-0.257528181	0.063219078
SPATS2	-0.001620157	0.996803176
SPC24	-0.002088053	0.995953997
SPC25	0.747496913	0.029502776
SPCS1	0.005510896	0.979840621
SPCS2	0.246897304	0.29859358
SPCS3	0.218292721	0.449235883
SPDEF	-0.130233786	0.298266643
SPDL1	0.467291248	0.20948538
SPDYA	1.781464925	0.000382747
SPDYC	-0.013350495	0.931383194
SPDYE1	-0.058167651	0.779159502
SPDYE3	0.05782694	0.693077328
SPECC1L	0.1078944	0.58021102

SPEF1	0.118496963	0.575794132
SPEF2	-0.078489979	0.795121827
SPEG	0.069589602	0.853715871
SPEM2	-0.070710442	0.591045589
SPEN	0.337896936	0.142677345
SPG11	0.477240208	0.009619098
SPG21	0.508699185	0.014849844
SPG7	0.359877548	0.054475095
SPHK1	0.275167688	0.285384169
SPHK2	-0.047828979	0.827654674
SPIB	-0.139954539	0.272107889
SPIC	-0.199653833	0.057699866
SPICE1	1.263265919	0.000106517
SPIN1	-0.399280423	0.081095673
SPIN2A	0.059390502	0.750617451
SPINDOC	0.018301743	0.926926533
SPINK13	0.029119207	0.716100726
SPINK14	-0.1540634	0.087866706
SPINK2	-0.031763452	0.857321112
SPINK4	-0.018646717	0.908376724
SPINK6	-0.037031831	0.70534963
SPINK7	-0.203399694	0.011661388
SPINT1	-0.098477426	0.65988307
SPINT4	-0.019507891	0.871201192
SPIRE1	-0.076105797	0.770808116
SPNS1	0.296395172	0.20573308
SPNS3	0.052837923	0.753117676
SPO11	-0.093970226	0.206867165
SPOCD1	0.597106909	0.347223044
SPOCK2	-0.24840262	0.64778447
SPON1	-0.145244749	0.878160468
SPOPL	0.11080532	0.473389208
SPOUT1	0.66300532	0.007391046
SPP2	-0.001736412	0.988588476
SPPL2A	0.336729267	0.184887584
SPPL2C	-0.147788631	0.33838734
SPR	0.137858354	0.549214914
SPRED1	0.50417924	0.162324805
SPRED2	0.37839252	0.340299987
SPRED3	0.176884967	0.417445896
SPRR1A	-0.020808246	0.885213989
SPRR1B	-0.045780531	0.730363414
SPRR2A	-0.124631586	0.66689123
SPRR2B	0.131905327	0.239336125
SPRR2D	-0.029094081	0.79460985
SPRR4	-0.206266784	0.140901274
SPRY2	0.84265589	0.160935633
SPRY3	0.228425778	0.377310626

SPRY4	1.663725151	0.015826873
SPRYD3	-1.211288514	0.001544789
SPRYD4	0.282213719	0.27770568
SPRYD7	-0.440009143	0.053492038
SPSB1	0.261799266	0.269592402
SPSB2	-0.087451921	0.345154663
SPSB3	0.02576072	0.917640056
SPSB4	0.391987247	0.170355392
SPTA1	-0.004608156	0.963013757
SPTBN1	-0.331587562	0.214706104
SPTBN2	-1.46889718	0.004305912
SPTBN4	-0.786611877	0.063340634
SPTBN5	-0.020295152	0.893241153
SPTLC2	-0.378627955	0.187076144
SPTLC3	0.040061622	0.941459946
SPTSSA	0.756003757	0.00097933
SPTSSB	-0.242137787	0.58497945
SPTY2D1	0.120033326	0.592780583
SPX	-0.459436862	0.049926726
SPZ1	-0.124254155	0.159828897
SQLE	-0.065781117	0.831104935
SQOR	0.902070062	0.013504955
SQSTM1	0.051523971	0.800109679
SRARP	-0.508087632	0.004058403
SRBD1	0.842996381	0.001454069
SRC	-0.234038793	0.269999177
SRCAP	0.565416537	0.006825681
SRCIN1	-1.27889504	0.003435521
SRD5A1	0.61122294	0.044023577
SRD5A2	-0.182187628	0.108090014
SRD5A3	-0.585078112	0.044369981
SREBF2	-0.809976113	0.006386181
SREK1	0.825664758	0.000968689
SREK1IP1	0.113666329	0.471442677
SRF	-0.149431969	0.577204384
SRFBP1	0.595663184	0.003777152
SRGAP1	1.448711795	0.000595082
SRGAP3	0.959838553	0.005145286
SRGN	0.582956359	0.453795581
SRI	0.411969961	0.07634901
SRM	-0.245151893	0.52308883
SRMS	-0.229596921	0.10084482
SRP14	0.08415288	0.592797021
SRP54	0.327158051	0.08103115
SRP68	-0.056757189	0.852908451
SRP72	0.13121413	0.629254753
SRPK1	-0.050524142	0.823214676
SRPK2	-0.363558018	0.317786978

SRPK3	-0.003282274	0.980027337
SRPRA	0.239174719	0.364387092
SRPRB	0.287278233	0.138969461
SRPX	1.100751744	0.212233703
SRPX2	1.141293711	0.231101199
SRRM1	0.121032928	0.573387796
SRRM2	0.380636111	0.110456596
SRRM4	-1.728658261	0.001422408
SRSF11	0.799642073	0.002172874
SRSF12	0.301427201	0.271519387
SRSF2	0.625416702	0.000613602
SRSF4	0.716995317	0.001454069
SRSF5	0.298974349	0.182130477
SRSF8	-0.048981688	0.845746964
SRSF9	0.881615272	4.99E-05
SRXN1	-0.373413962	0.128558957
SRY	-0.041255096	0.711612584
SS18L1	-0.124046863	0.618630164
SS18L2	0.16583716	0.334342007
SSB	0.156288027	0.361947699
SSBP2	0.351899117	0.105121233
SSBP3	-0.263434946	0.40680463
SSBP4	0.115732461	0.762190129
SSC4D	-0.021399337	0.888728833
SSH1	0.267086895	0.485880843
SSH2	0.160449832	0.648779833
SSH3	-0.349287609	0.084312483
SSMEM1	-0.117190654	0.110059166
SSNA1	-0.082378076	0.59772495
SSPN	-0.076944276	0.475371044
SSR1	0.151299347	0.519665265
SSR3	0.155458115	0.667053498
SSR4	0.229632967	0.291869479
SSRP1	0.799500275	0.003140901
SST	-1.935870691	0.012809431
SSTR1	-0.558912772	0.165752439
SSTR2	-0.738261873	0.07904689
SSTR4	-0.176997157	0.234706512
SSTR5	-0.206639783	0.145358656
SSU72	-0.045015677	0.801708776
SSUH2	0.160441033	0.346371233
SSX1	-0.124418452	0.376317376
SSX3	-0.157953377	0.108120642
SSX4	-0.100152205	0.293451908
SSX5	-0.232264761	0.057059545
SSX7	-0.037852567	0.766400946
ST13	0.204125351	0.194885839
ST14	0.521981316	0.090598385

ST18	-1.441681518	0.01044807
ST3GAL1	-0.308366217	0.109565374
ST3GAL2	0.02199163	0.931284293
ST3GAL3	0.233232792	0.130844614
ST3GAL4	0.096693169	0.664720648
ST3GAL5	0.783938439	0.075408885
ST3GAL6	0.250394512	0.183879085
ST6GAL1	0.460699795	0.337687984
ST6GALNAC1	-0.363069393	0.012288804
ST6GALNAC2	0.588243739	0.086333407
ST6GALNAC3	-0.633504482	0.011829886
ST6GALNAC4	0.056664822	0.752633615
ST6GALNAC5	-1.377825947	0.008098465
ST6GALNAC6	-0.88778957	0.004564614
ST7	0.501610435	0.108325357
ST7L	0.196460634	0.247750029
ST8SIA1	0.447306956	0.141921844
ST8SIA3	-2.298736177	0.023815176
ST8SIA4	0.696793534	0.020517273
ST8SIA5	-0.76314965	0.240533047
ST8SIA6	-0.155982501	0.372787239
STAB1	1.950128572	0.003977596
STAB2	-0.063915367	0.417929756
STAC	-0.095629783	0.890955285
STAC2	-0.250315127	0.668142275
STAC3	0.50504675	0.002565396
STAG1	0.69448224	0.004992742
STAG3	0.225482639	0.065878376
STAM	-0.789798526	0.006188209
STAM2	0.314504501	0.144270603
STAMBP	0.223118918	0.209830984
STAMBPL1	-0.796072425	0.023806817
STAP1	0.057427227	0.59692935
STAP2	-0.015167575	0.941983489
STAR	-0.255260262	0.107375492
STARD10	-0.501169395	0.017141009
STARD13	0.325384473	0.116731387
STARD3	0.452185325	0.010505622
STARD3NL	0.352198124	0.095937561
STARD4	-0.472791893	0.248798732
STARD5	-0.203808923	0.31490984
STARD6	-0.069352055	0.490425951
STARD7	-0.123527766	0.523499736
STAT1	1.024685082	0.006497556
STAT2	0.834399028	0.014160616
STAT4	-1.430651128	0.00231787
STAT5B	0.198955785	0.221394959
STAU1	0.02135663	0.906358055

STC1	0.200080114	0.831104935
STC2	-0.063210515	0.935415912
STEAP1	0.152586453	0.481954142
STEAP3	1.967885231	0.027165261
STIL	0.568237322	0.190829597
STIM1	0.142136133	0.503333093
STIP1	0.177895592	0.391553303
STK10	0.858562821	0.01845413
STK11IP	0.166445059	0.234483133
STK17A	1.499974628	0.007553782
STK17B	0.574267841	0.022188125
STK24	-0.124032865	0.537165089
STK25	-0.07021392	0.569712378
STK26	-0.482484111	0.088481151
STK31	-0.067325822	0.581403145
STK32A	1.956997625	2.32E-05
STK32B	0.053542354	0.889851708
STK32C	-0.062077882	0.605851261
STK33	1.522018288	0.002363145
STK35	0.099176451	0.47867356
STK38L	-0.024750396	0.926133015
STK39	-0.408843561	0.064510865
STK4	0.374983314	0.002943203
STKLD1	-0.208755231	0.100661194
STMN3	-0.646133231	0.059949188
STMN4	-1.403843374	0.003490517
STMND1	0.073602613	0.676901408
STN1	-0.08832101	0.736392111
STOM	-0.063588033	0.912632913
STOML2	-0.301660548	0.327800169
STON2	0.04503868	0.928188661
STPG1	-0.471785554	0.022100665
STPG2	0.010868175	0.946415853
STPG3	-0.240449501	0.082058452
STPG4	-0.133651628	0.480832489
STRA8	-0.049775427	0.631446775
STRADA	0.414265429	0.038372241
STRADB	0.024922629	0.904256812
STRAP	-0.143181837	0.574475876
STRC	-0.368526662	0.035197777
STRIP1	0.155078695	0.341121661
STRN	-0.301587261	0.240723714
STRN4	-0.042193112	0.872052146
STS	-0.823521908	0.033272095
STT3B	0.348716116	0.156566897
STUB1	-0.94917639	0.00044063
STUM	-0.121108401	0.642247046
STX10	0.219688291	0.333221325

STX11	0.177317335	0.564973097
STX12	-0.001161602	0.993666428
STX17	0.172802552	0.283439618
STX18	0.209412839	0.119341573
STX1A	-1.392390088	0.000511553
STX1B	-2.241982603	0.005726112
STX2	0.411454462	0.046139083
STX4	0.113006445	0.68280633
STX6	0.050478094	0.719619039
STX7	-0.210755047	0.339600809
STX8	-5.91E-05	0.999827462
STXBP1	-1.728981956	0.004064451
STXBP2	0.264419137	0.303934395
STXBP3	0.235835393	0.271558363
STXBP4	0.885390801	0.00297406
STXBP5	-1.084955384	0.017441341
STXBP5L	-2.61126345	0.003202538
STXBP6	-1.117769513	0.000475064
STYK1	-0.656522392	0.011393746
STYX	0.247407816	0.589126878
STYXL1	0.155327823	0.48715344
SUB1	0.576840842	0.006613337
SUCLA2	-0.422407978	0.076360903
SUCLG1	-0.240979365	0.183176919
SUCNR1	-0.244862258	0.094233344
SUCO	0.385081984	0.142159077
SUDS3	0.264861319	0.168681476
SUFU	-0.084580399	0.72777771
SUGP1	0.265474742	0.197253085
SUGT1	-0.204091997	0.321891972
SULF2	1.013966828	0.139569652
SULT1A1	-0.288724649	0.059425494
SULT1B1	-0.11843714	0.731984105
SULT1C2	0.110693204	0.539746
SULT1C4	0.766066413	0.109221693
SULT1E1	-0.210982428	0.373930983
SULT2A1	-0.153952191	0.215113125
SULT2B1	-0.265995807	0.050270949
SULT6B1	-0.199198101	0.087123107
SUMF1	0.318682962	0.033882818
SUMO1	0.170458461	0.375950158
SUMO3	-0.120901114	0.521898562
SUN2	-0.072217765	0.75582128
SUN5	0.036165343	0.781711754
SUOX	0.45934791	0.049245411
SUPT16H	0.081557331	0.730363414
SUPT3H	-0.934414594	0.005445074
SUPT4H1	-0.147588857	0.446115446

SUPT5H	-0.036445481	0.884159274
SUPT7L	0.300795251	0.091890711
SUPV3L1	-0.485892046	0.082058452
SURF1	-0.000657723	0.998128955
SURF2	0.301493991	0.041961227
SURF4	0.42672508	0.203783861
SURF6	0.169278376	0.351536825
SUSD1	0.091399049	0.75726135
SUSD2	0.894520761	0.033633563
SUSD3	0.127475052	0.510240413
SUSD4	-0.194971243	0.752202506
SUSD6	0.817320773	0.018775605
SUV39H1	-0.049432937	0.802283726
SUZ12	0.600505913	0.015361754
SV2B	-2.602095342	0.007837981
SV2C	-0.403354083	0.348378671
SVBP	-0.078106767	0.773333319
SVIL	-0.018882677	0.98286235
SVIP	-0.373673363	0.011608304
SVOP	-1.323316282	0.009480447
SWAP70	0.057631054	0.882695839
SWSAP1	0.058020937	0.663881113
SWT1	0.053656443	0.735754893
SYAP1	0.074594273	0.793328446
SYCE2	0.426749289	0.079742987
SYCP1	-0.148994939	0.0409604
SYCP2	0.384359313	0.334714691
SYDE1	0.22781032	0.55266491
SYMPK	0.134219665	0.665053766
SYN1	-2.771097935	0.010259208
SYN3	-1.64498821	0.008749587
SYNC	-0.320845136	0.42819277
SYNDIG1	-0.372801604	0.30787282
SYNE1	-0.876897153	0.024101505
SYNE2	1.43071997	0.001507636
SYNE3	0.292798758	0.104292401
SYNGR1	-0.55568753	0.018435704
SYNGR2	-0.481083693	0.320148985
SYNGR3	-0.977584944	0.006091959
SYNGR4	-0.286538321	0.056043915
SYNJ1	-0.594930434	0.170990838
SYNJ2	-1.813929097	0.001607242
SYNM	0.046768705	0.907209684
SYNPR	-0.594762889	0.012809986
SYNRG	0.303802735	0.073149775
SYPL1	0.283880872	0.247896619
SYT1	-2.178120085	0.003660561
SYT10	-0.318059229	0.036591578

SYT12	-0.062313502	0.650328231
SYT15	-0.937320927	0.011867165
SYT16	-1.077415344	0.006890676
SYT17	0.794939822	0.0613912
SYT3	-0.787673628	0.044768969
SYT4	-2.355492568	0.014251391
SYT5	-1.379910344	0.010555103
SYT8	0.087876459	0.295937093
SYT9	-0.826689143	0.004811183
SYTL2	-0.532563214	0.162538859
SZRD1	0.052128519	0.807115118
SZT2	0.596239122	0.008975543
TAAR1	0.013622597	0.896454017
TAAR2	0.069461575	0.379281394
TAAR5	0.131401696	0.348023905
TAAR6	-0.024042286	0.819233636
TAAR8	-0.048859387	0.605799318
TAAR9	-0.083324236	0.390611771
TAB1	-0.116573797	0.601985624
TAB2	-0.064264455	0.791865875
TAB3	-0.175534484	0.327635999
TAC1	-0.313350801	0.202186223
TAC3	0.150817054	0.620025088
TAC4	-0.161937819	0.399340316
TACC1	-0.386051742	0.141985137
TACC2	0.11278562	0.65000111
TACC3	0.585350287	0.077450452
TACO1	0.232556703	0.179104238
TACR1	0.197462749	0.558549984
TACR2	-0.072602197	0.512639536
TACR3	-0.199687022	0.193669061
TACSTD2	-0.171702628	0.29545465
TADA1	-0.011634052	0.958909167
TAF1	0.010268769	0.951704316
TAF10	-0.145363465	0.542356935
TAF11	0.437704256	0.056755453
TAF12	0.468205141	0.013375961
TAF13	-0.178506816	0.621265546
TAF15	0.481729835	0.017090165
TAF1A	0.44895441	0.004828369
TAF1B	0.652907974	0.021385313
TAF1C	0.750461127	0.006185744
TAF1D	-0.50157768	0.016124829
TAF1L	0.0151581	0.924571982
TAF2	0.310254315	0.190374885
TAF3	-0.012626706	0.963152726
TAF4	0.355474259	0.060490531
TAF5	0.006714702	0.98042003

TAF5L	0.232555524	0.295585953
TAF6	0.231363381	0.30734069
TAF8	0.528135028	0.005161235
TAF9	0.297084543	0.089504455
TAF9B	-0.007228268	0.987724044
TAFA1	-1.056866736	0.010638687
TAFA2	-0.594495478	0.019571398
TAFA3	0.390052852	0.315747165
TAFA4	-0.113937723	0.409284168
TAFA5	-0.421669298	0.127744707
TAGAP	0.890266732	0.015877342
TAGLN2	0.413494047	0.406099532
TAL1	-0.051220718	0.847725163
TAL2	-0.058135457	0.692469824
TALDO1	-0.126471711	0.576597914
TAMM41	0.329755912	0.113639888
TANGO2	0.005984265	0.981721885
TANK	0.608555647	0.002172874
TAOK1	0.009147768	0.970717025
TAOK2	0.07837639	0.787365837
TAOK3	0.623596874	0.032879525
TAP1	1.008956355	0.008352909
TAP2	0.538933854	0.007543692
TAPBP	0.194487322	0.429798231
TAPBPL	0.70863427	0.001454069
TAPT1	-0.160397383	0.601985624
TARBP1	-0.037352418	0.916630472
TARBP2	0.149759942	0.503836731
TARDBP	0.483057484	0.018713776
TAS1R1	0.134114838	0.387527537
TAS1R2	-0.030273401	0.80938792
TAS1R3	-0.158762113	0.150559807
TAS2R1	-0.12101657	0.114177498
TAS2R10	0.263701167	0.409675613
TAS2R13	0.248082835	0.135997589
TAS2R14	0.267772028	0.460686163
TAS2R16	-0.094305984	0.318764205
TAS2R19	0.413297957	0.16989969
TAS2R20	0.33143831	0.042774935
TAS2R3	0.568717768	0.038766047
TAS2R30	0.374467596	0.305389973
TAS2R31	1.196910185	0.000744793
TAS2R38	-0.055686436	0.537165089
TAS2R39	-0.058223652	0.568950838
TAS2R4	0.804311483	0.001530337
TAS2R40	-0.232179965	0.079742987
TAS2R41	0.085610345	0.606390156
TAS2R42	-0.062854777	0.390253816

TAS2R43	0.128452441	0.305948766
TAS2R46	0.404217429	0.110456596
TAS2R5	0.622800757	0.032425399
TAS2R50	0.15856843	0.436517988
TAS2R60	-0.048215439	0.701598088
TAS2R7	-0.148284731	0.132445969
TAS2R8	-0.064500618	0.464564895
TAS2R9	-0.052197832	0.816084385
TASP1	0.35606813	0.013416891
TAT	0.377262274	0.512982653
TAX1BP1	-0.090992958	0.485111162
TAZ	0.160055774	0.517388907
TBATA	0.06112197	0.582945807
TBC1D10B	0.138290357	0.425097598
TBC1D13	-0.278740179	0.035918142
TBC1D14	0.223631929	0.329463009
TBC1D16	0.351116653	0.180879322
TBC1D17	-0.362411977	0.198727899
TBC1D19	0.082916833	0.731474241
TBC1D21	-0.133702639	0.203901234
TBC1D22A	0.141504315	0.58596993
TBC1D22B	0.225482528	0.160515731
TBC1D25	0.048120214	0.814709933
TBC1D28	-0.183455511	0.181224257
TBC1D31	-0.028379801	0.874708742
TBC1D32	0.56833206	0.035197777
TBC1D7	0.227174374	0.339412764
TBC1D8B	-0.184324365	0.55298525
TBC1D9B	0.061932052	0.750419071
TBCA	0.29901502	0.077798541
TBCD	0.179116574	0.340627648
TBCEL	0.056340209	0.83517312
TBK1	0.315912065	0.096531295
TBL1X	0.276212164	0.27519833
TBL1XR1	0.285379939	0.109575774
TBL1Y	-0.064382899	0.736206294
TBL2	0.497364894	0.06380888
TBP	0.175223394	0.347674054
TBPL2	-0.033994199	0.70495706
TBR1	-1.680115917	0.001454069
TBRG1	-0.248507021	0.204256557
TBRG4	0.226121583	0.28895102
TBX1	-0.079310644	0.556360059
TBX10	-0.178871799	0.296839898
TBX18	-0.295005649	0.378481287
TBX19	0.139492374	0.512915077
TBX2	0.548299619	0.060370505
TBX20	-0.181074999	0.188962339

TBX21	-0.108187451	0.616944401
TBX3	0.149932215	0.544529109
TBX4	0.087732938	0.492496846
TBX5	0.563428498	0.405122436
TBX6	0.090570259	0.603778013
TBXA2R	0.221077443	0.29859358
TBXT	0.298617219	0.559486406
TCAIM	-0.142191654	0.671138397
TCAP	-0.18024058	0.329369557
TCEA1	0.430561981	0.008064299
TCEA2	-0.319733099	0.015278861
TCEA3	1.046626161	0.017720052
TCEAL1	0.036676065	0.861348324
TCEAL2	-0.464083016	0.159223076
TCEAL3	-0.2486253	0.317017888
TCEAL4	0.018215095	0.923498499
TCEAL7	-0.802432097	0.036191403
TCEAL8	-0.069157365	0.728905441
TCEAL9	0.558295652	0.155016527
TCEANC2	0.379869734	0.012784279
TCERG1	0.163142966	0.340516155
TCERG1L	-0.711696667	0.010079794
TCF15	-0.034291664	0.828704205
TCF21	0.037922278	0.845168366
TCF23	-0.203820292	0.183680045
TCF7L1	0.946311407	0.007242844
TCFL5	-0.541826803	0.089488133
TCHP	0.474358117	0.015543214
TCIM	1.106677539	0.028932489
TCIRG1	0.670809513	0.06848807
TCL1B	0.043207332	0.755616949
TCN1	-0.004135877	0.974469226
TCN2	0.469585145	0.119590948
TCP1	-0.145824747	0.6429669
TCP10L	-0.144945044	0.653009152
TCP10L2	0.046191565	0.663494227
TCP11L1	-0.329546966	0.067318223
TCP11L2	-0.155646945	0.557596975
TCTA	-0.236663645	0.125506675
TCTE1	0.316281452	0.078981808
TCTN1	0.991053312	0.000511553
TCTN3	-0.179619474	0.587701111
TDG	0.334326047	0.293583349
TDGF1	-0.04127811	0.778703276
TDO2	0.493572103	0.305176366
TDP1	0.053499136	0.841954421
TDP2	-0.073301512	0.670757694
TDRD1	-0.124112662	0.228262486

TDRD3	-0.109970777	0.600699957
TDRD7	0.315494061	0.097337244
TDRD9	-0.242553567	0.085789536
TDRP	0.519516024	0.006580315
TEAD1	0.603756325	0.099639765
TEAD3	0.461021864	0.377488879
TEAD4	-0.041698807	0.921484201
TEC	-0.241730514	0.065878376
TECPR1	0.247291424	0.284248254
TECRL	-0.067209073	0.444756716
TECTA	0.122910189	0.479498653
TECTB	0.197926469	0.404357997
TEDC2	0.184796476	0.290250443
TEDDM1	-0.232671064	0.042243736
TEF	-0.362769871	0.035648781
TEFM	0.415353734	0.032216345
TEK	-0.636296386	0.03184771
TEKT1	0.309098819	0.406355565
TEKT2	0.667988972	0.203050772
TEKT3	0.040371343	0.81839364
TEKT4	-0.04362691	0.798614335
TEKT5	0.030856907	0.799968711
TELO2	0.06644066	0.740003676
TENM3	-1.599657367	0.002142284
TENM4	0.398653189	0.325895121
TENT4A	0.538179357	0.025792332
TENT5A	1.400800825	0.002150168
TENT5B	0.165740161	0.505928126
TENT5C	0.12882301	0.490269353
TEP1	1.167566655	0.000389052
TEPP	-0.076244757	0.485880843
TEPSIN	0.043726256	0.806535926
TERB2	-0.082226971	0.385170796
TERF1	0.000942034	0.9973031
TERF2	-0.025316928	0.882117081
TERF2IP	-0.619920156	0.0240514
TERT	-0.044064665	0.794214198
TES	-0.518105648	0.471442677
TESK1	-0.223081113	0.227110312
TESK2	-0.361038561	0.317915289
TESMIN	-0.115188891	0.189348765
TET1	0.430468977	0.227157722
TET3	0.366464245	0.033469262
TEX10	0.676079147	0.041552435
TEX101	-0.256234776	0.022457395
TEX11	-0.075889705	0.506700199
TEX13A	-0.040514968	0.752183858
TEX13B	-0.074952519	0.589701197

TEX14	0.090141283	0.698905873
TEX15	0.004188754	0.990996918
TEX19	-0.090972013	0.56239321
TEX2	-0.887231435	0.003717794
TEX26	0.410290908	0.059786399
TEX261	0.306169081	0.064735077
TEX264	0.170267537	0.372780807
TEX29	-0.39540042	0.06969506
TEX30	0.323030748	0.074282574
TEX33	-0.261765478	0.041708728
TEX35	0.330106081	0.03718731
TEX37	-0.240551697	0.042178398
TEX44	-0.051292989	0.747350354
TEX45	-0.150829723	0.234475496
TEX47	0.010320884	0.934236436
TEX51	-0.103109323	0.257269688
TEX55	-0.135591176	0.084833849
TEX9	1.00738064	0.002957531
TF	-1.459558547	0.085460485
TFAM	0.267096016	0.179313418
TFAP2A	0.335490227	0.169782421
TFAP2B	0.480035661	0.328283112
TFAP2C	-0.548262294	0.189557746
TFAP2D	-0.145982003	0.139706069
TFAP2E	-0.229027048	0.169794477
TFAP4	0.115628477	0.625507497
TFB1M	0.404301312	0.055086894
TFB2M	0.047064713	0.858985019
TFCP2L1	1.355686919	0.043403256
TFDP1	0.100614394	0.649045963
TFDP3	-0.36112662	0.023991462
TFE3	-0.094846779	0.671295451
TFEB	0.021592967	0.920603476
TFEC	0.2972223	0.047896467
TFF1	-0.19747043	0.061509092
TFF2	-0.126653255	0.162029152
TFF3	0.183706916	0.31754923
TFG	0.035671504	0.883425636
TFIP11	0.070757708	0.668563803
TFPI2	-0.28585572	0.520268919
TFPT	-1.114478425	0.001620311
TFR2	0.018877773	0.940935752
TFRC	0.47162267	0.173946935
TG	0.090019094	0.382797194
TGDS	0.445402148	0.065408457
TGFA	-0.240251322	0.28073347
TGFB1	0.493440647	0.406099532
TGFB1I1	0.56912639	0.488224575

TGFB3	-0.06003207	0.912659903
TGFBI	0.775559978	0.385767435
TGFBR1	1.030850964	0.009312966
TGFBR3	0.047215607	0.941984856
TGFBRAP1	0.164272323	0.272646915
TGIF1	0.60852718	0.017266937
TGIF2LX	-0.084203521	0.346281317
TGIF2LY	-0.096989463	0.710778616
TGM1	-0.255878083	0.080901275
TGM2	-0.903091345	0.036119739
TGM3	-0.011730086	0.924928069
TGM4	-0.103507846	0.270261389
TGM5	-0.030229874	0.878677857
TGM6	-0.049543004	0.77132529
TGM7	-0.054655931	0.625457691
TGOLN2	0.19869991	0.42128601
TGS1	0.288593966	0.158088917
TH	-0.037260625	0.761697772
THAP1	0.057131991	0.726208146
THAP10	-0.205786727	0.397987844
THAP11	0.031953684	0.897050949
THAP12	0.202404979	0.203243282
THAP2	0.27025086	0.093804428
THAP3	0.251971	0.211874026
THAP4	-0.282542092	0.087410295
THAP6	0.376628983	0.061048788
THAP7	0.078965472	0.67159631
THAP8	0.116133832	0.562821065
THAP9	0.352354069	0.08431809
THBD	0.083248258	0.809182602
THBS1	-0.155044257	0.91733085
THBS2	0.062806172	0.927323396
THBS4	1.231149224	0.024414512
THEG	-0.056838696	0.602016891
THEM4	0.090578718	0.864288922
THEM5	-0.258196448	0.031528115
THEM6	-0.133989038	0.314300073
THEMIS2	0.705246618	0.143372073
THG1L	0.375786708	0.113921677
THNSL1	-0.127478834	0.650417176
THOC3	0.05391212	0.835499613
THOC6	0.483165258	0.04439483
THOC7	-0.287847378	0.073008417
THOP1	0.08204178	0.728900852
THPO	-0.105746045	0.6062359
THRSP	-0.004388682	0.976861091
THSD4	-0.738772293	0.003741174
THUMPD1	0.089726313	0.663942355

THUMPD3	0.354762125	0.140563462
THY1	-0.407140166	0.240200614
THYN1	0.076711086	0.756216671
TIA1	0.866109262	0.002679628
TIAM1	-0.426386498	0.21213973
TICAM1	-0.262338886	0.185743937
TICAM2	0.097257644	0.695912218
TICRR	0.851555172	0.073507284
TIE1	-0.205849403	0.325895121
TIFA	0.010519711	0.956911263
TIFAB	-0.165834818	0.193669061
TIGAR	-0.116395456	0.747730826
TIGD1	0.859856203	0.00044063
TIGD2	0.425119805	0.099639765
TIGD3	0.035633162	0.69480838
TIGD4	0.347668483	0.058987158
TIGD5	0.131480799	0.406099532
TIGD7	0.460215357	0.011313988
TIGIT	-0.165777642	0.103795913
TIMD4	0.075866265	0.479580596
TIMELESS	1.179742962	0.009319251
TIMM10	-0.159493489	0.254576415
TIMM10B	-0.411821078	0.211353461
TIMM17A	-0.069319229	0.690144407
TIMM21	0.282948319	0.175622233
TIMM22	-0.126492289	0.751047947
TIMM29	0.071105516	0.637981361
TIMM50	-0.013844583	0.969065635
TIMM8A	0.217337124	0.481196182
TIMM9	0.33256671	0.06554543
TIMMDC1	0.010656493	0.972663977
TIMP1	1.960025924	0.009605955
TIMP2	0.588912091	0.044369981
TIMP3	0.150602027	0.665904933
TIMP4	2.712271644	0.00057149
TINAG	-0.083849049	0.392870911
TINCR	0.030052041	0.858244318
TIPIN	0.401102036	0.02383959
TIPRL	0.257084375	0.368571166
TJP1	-0.598505475	0.061486368
TJP2	-0.069122627	0.868550554
TK2	0.061948535	0.707562098
TKFC	0.414097271	0.042998566
TKT	-0.088217235	0.773493792
TKTL1	0.284459444	0.727460808
TKTL2	-0.216616958	0.152174013
TLCD3A	0.427946446	0.133924264
TLCD3B	-1.141791034	0.001944812

TLDC2	0.016918892	0.891101631
TLE1	0.457327307	0.107462271
TLE2	0.512981552	0.188151106
TLE3	0.375815234	0.052695296
TLE4	-0.501996115	0.024305591
TLE5	-0.49978127	0.048048058
TLK1	0.438410346	0.027490144
TLK2	0.369039468	0.036926773
TLL1	-0.152215033	0.623230316
TLL2	-0.38151437	0.015346199
TLN1	0.211320299	0.625084775
TLN2	-0.542460958	0.078529646
TLR1	0.593883781	0.028490862
TLR10	0.285123295	0.23852602
TLR2	0.966571199	0.023168931
TLR3	0.772047205	0.015655094
TLR4	-0.381144779	0.393204579
TLR5	0.793808677	0.016287042
TLR6	0.394103595	0.033039576
TLR7	0.843615283	0.039963264
TLR8	0.333517465	0.0362885
TLX1	-0.003740815	0.983410163
TLX2	-0.113764842	0.326841159
TLX3	-0.211067434	0.145025939
TM2D1	0.263926954	0.091598211
TM2D2	0.109790181	0.746595096
TM2D3	0.017301805	0.948270602
TM4SF1	-0.175859532	0.796566753
TM4SF18	0.26608112	0.279849035
TM4SF20	-0.122244881	0.162286208
TM4SF4	-0.094087981	0.368662343
TM4SF5	-0.088844307	0.49846128
TM6SF1	0.479578583	0.29495708
TM7SF2	-0.237888673	0.193417506
TM7SF3	0.159897872	0.601985624
TM9SF2	0.032979443	0.908578479
TM9SF3	-0.42385184	0.145025939
TMA16	0.442471134	0.035553998
TMA7	-0.112067766	0.553384688
TMBIM1	-0.038030744	0.90380542
TMBIM4	0.572263277	0.012629466
TMC1	0.103237379	0.51277895
TMC2	-0.164766534	0.171793831
TMC3	0.004685577	0.971953247
TMC7	-0.56040439	0.278333668
TMCC2	-0.536510822	0.036294358
TMCC3	-0.092524472	0.762740359
TMCO3	0.038273207	0.913028318

TMCO4	0.23364549	0.345154663
TMCO5A	-0.033483711	0.659463792
TMED1	0.055162477	0.845106603
TMED10	0.458004322	0.060370505
TMED2	0.238813192	0.286747095
TMED3	-0.115003143	0.668917615
TMED4	0.499030509	0.01044807
TMED6	0.001007115	0.995560506
TMED8	-0.501363635	0.059425494
TMED9	0.371470313	0.279849035
TMEFF1	-0.703003042	0.046632821
TMEFF2	-1.084574392	0.046493806
TMEM101	0.164298402	0.277203467
TMEM102	0.132397003	0.496608658
TMEM104	0.364444823	0.1901552
TMEM106B	0.477891386	0.008614407
TMEM107	0.460144338	0.028631206
TMEM108	0.098657433	0.782749113
TMEM109	-0.067398289	0.823177958
TMEM114	0.266755989	0.072841553
TMEM115	0.204392973	0.262254936
TMEM116	0.328086097	0.220045427
TMEM117	0.155116854	0.519310408
TMEM119	0.14466736	0.673087029
TMEM121	-0.133703744	0.350979442
TMEM121B	-0.715972514	0.012910317
TMEM123	0.210336266	0.258106699
TMEM125	0.00157528	0.993666428
TMEM126A	-0.162891551	0.466288438
TMEM127	-0.01466424	0.949150237
TMEM128	0.619206077	0.036794892
TMEM132A	-0.005154171	0.989136356
TMEM132D	-1.309814722	0.001041012
TMEM132E	0.166382803	0.596943625
TMEM134	-0.014384941	0.935244061
TMEM135	0.084833547	0.676327171
TMEM138	0.241554504	0.34166678
TMEM139	0.064643641	0.658374119
TMEM140	0.611135823	0.088557311
TMEM143	-0.375872975	0.009786616
TMEM144	-0.913864015	0.20573308
TMEM145	-0.52341978	0.3273107
TMEM147	0.098885514	0.739736703
TMEM14A	-0.442984476	0.068196972
TMEM14B	-0.117667236	0.596519233
TMEM150B	0.260684084	0.202380032
TMEM150C	-0.99444349	0.056472512
TMEM151A	-1.498168264	0.000313636

TMEM151B	-0.670354859	0.000815172
TMEM154	0.197324752	0.358825148
TMEM156	-0.092089897	0.826785392
TMEM158	0.880569614	0.111109571
TMEM160	-1.0427242	4.99E-05
TMEM161A	0.187291394	0.552352587
TMEM161B	0.085430206	0.64590421
TMEM163	0.20068862	0.707337139
TMEM164	0.478016932	0.018943464
TMEM165	-0.167600866	0.620023788
TMEM167A	0.095006695	0.546766193
TMEM167B	0.344604672	0.01413174
TMEM168	0.807511351	0.006195115
TMEM169	0.210342262	0.42896696
TMEM17	0.040757068	0.744791797
TMEM170A	0.290606523	0.312373019
TMEM171	-0.477697474	0.177287889
TMEM174	-0.047886584	0.599416085
TMEM175	-0.044927464	0.862447143
TMEM176A	0.277613987	0.412147265
TMEM178A	-0.680067137	0.113044747
TMEM179	-0.440345783	0.039959279
TMEM179B	0.305004483	0.430953947
TMEM18	0.327787736	0.014002435
TMEM182	0.130546509	0.18666232
TMEM183A	0.42530704	0.005947029
TMEM184C	-0.126646084	0.731977162
TMEM186	0.17396312	0.305375238
TMEM187	-0.086775185	0.652822208
TMEM190	-0.025440585	0.84159081
TMEM196	-0.526974217	0.233702991
TMEM198	0.112685942	0.500185066
TMEM199	0.058852145	0.705327879
TMEM200A	-0.305540603	0.075884325
TMEM203	0.31504438	0.048768087
TMEM204	0.548778461	0.069126309
TMEM205	-0.060204144	0.843825329
TMEM207	-0.089905428	0.406035977
TMEM208	0.303944042	0.083132498
TMEM209	0.595663527	0.044164454
TMEM210	0.065654742	0.663881113
TMEM211	-0.335234663	0.021385313
TMEM212	-0.031468654	0.824830805
TMEM213	-0.111131692	0.289558045
TMEM215	0.071106637	0.440193934
TMEM216	0.038190627	0.843090108
TMEM219	0.252313806	0.227110312
TMEM220	0.32249704	0.234889728

TMEM223	-0.130188091	0.29545465
TMEM229B	0.433353866	0.017588539
TMEM237	0.277348839	0.253702641
TMEM239	-0.226977425	0.220212658
TMEM241	-0.391207296	0.076338257
TMEM243	0.459685532	0.042969502
TMEM245	-0.488705537	0.062044321
TMEM248	0.144524738	0.451027975
TMEM25	-0.495454698	0.127493472
TMEM250	-0.233502063	0.094098876
TMEM251	0.348344257	0.032988136
TMEM252	-0.059158616	0.670737227
TMEM253	0.040203886	0.731136744
TMEM255A	2.554737861	3.51E-05
TMEM255B	0.588785069	0.007821977
TMEM258	-0.02899168	0.875151351
TMEM259	0.30444329	0.115969619
TMEM26	-0.011129307	0.938370209
TMEM260	-0.104798349	0.618336681
TMEM263	0.795408943	0.017791812
TMEM266	-0.222761396	0.112146564
TMEM267	0.302162872	0.151947457
TMEM268	-0.84633843	0.013504955
TMEM270	-0.164949756	0.22489608
TMEM273	0.015580297	0.946627587
TMEM30A	0.060406957	0.749326279
TMEM30B	-0.151643891	0.506700199
TMEM31	0.034764256	0.86089558
TMEM33	0.543062428	0.003075463
TMEM35A	-0.868644627	0.070990975
TMEM38A	-0.477557266	0.336020775
TMEM38B	0.075690111	0.863055486
TMEM39A	0.94085075	0.003979493
TMEM39B	0.160211632	0.197968577
TMEM40	-0.34236414	0.2491227
TMEM41A	0.351166129	0.015346199
TMEM42	0.211136182	0.231403456
TMEM44	-0.042105591	0.893241153
TMEM45A	1.331566349	0.016780753
TMEM45B	0.009579694	0.966778229
TMEM47	-0.944406478	0.043005694
TMEM50A	-0.069782406	0.794214198
TMEM52	-0.117535296	0.234119708
TMEM52B	-0.157712922	0.202710133
TMEM53	0.073651773	0.700015813
TMEM54	0.369341964	0.111023214
TMEM59	0.078432107	0.615096809
TMEM59L	-1.076220062	0.048659492

TMEM60	0.248775269	0.185998916
TMEM61	0.114307044	0.240239771
TMEM62	-0.061750749	0.838282366
TMEM63B	-0.20302125	0.227494389
TMEM64	-0.047061903	0.843863404
TMEM65	-0.295037655	0.199940891
TMEM67	0.804657657	0.004751113
TMEM68	0.098236097	0.620047289
TMEM69	0.147596169	0.566476311
TMEM71	0.816146798	0.116972907
TMEM72	-0.079642003	0.693517481
TMEM74	0.035080216	0.862276842
TMEM74B	-0.075299443	0.79400762
TMEM79	0.235376452	0.109119511
TMEM80	0.33738691	0.116419877
TMEM81	0.222938279	0.352491367
TMEM87A	0.467243807	0.049245411
TMEM87B	0.463441426	0.115071693
TMEM89	-0.317593725	0.135342825
TMEM9	-0.046660488	0.790615582
TMEM92	-0.176398954	0.374228363
TMEM94	-0.120308121	0.716721669
TMEM95	-0.013035186	0.932999857
TMEM97	0.317801082	0.323763042
TMEM98	-0.911576299	0.039856062
TMEM9B	-0.168898623	0.506810291
TMF1	0.51691708	0.038331746
TMIE	-0.326230676	0.08893089
TMIGD1	-0.087636372	0.248138036
TMIGD2	-0.118137936	0.552725651
TMLHE	0.375808501	0.116756442
TMOD2	-1.077459321	0.093677832
TMOD3	0.099759272	0.737435487
TMPRSS11A	0.004743091	0.971953247
TMPRSS11B	-0.106408131	0.246080462
TMPRSS11D	-0.130297235	0.156600389
TMPRSS11E	-0.166728688	0.044164454
TMPRSS11F	0.029230815	0.740558635
TMPRSS12	-0.186038707	0.036741407
TMPRSS15	-0.041817277	0.644122748
TMPRSS4	-0.156979502	0.324819162
TMPRSS5	0.656006973	0.052479337
TMPRSS6	-0.225625744	0.06039945
TMPRSS9	-0.076373009	0.657224321
TMSB10	-0.081170629	0.879932781
TMSB15A	-0.138847722	0.678115572
TMSB15B	0.133816761	0.34329054
TMSB4X	0.508839132	0.090466736

TMSB4Y	0.519554546	0.207666044
TMTC2	-0.086299679	0.887269342
TMTC3	-0.114762284	0.769800562
TMTC4	-0.189540361	0.658038853
TMUB2	0.121747213	0.505656412
TMX1	0.961856512	0.004285981
TMX3	0.496549523	0.079742987
TMX4	-0.047730216	0.901235662
TNC	3.405904684	0.001041012
TNF	-0.026847942	0.917640056
TNFAIP1	-0.477110522	0.044567499
TNFAIP2	1.015776987	0.035553998
TNFAIP3	1.454880611	0.019234826
TNFAIP6	1.584948914	0.009106737
TNFAIP8	0.135198567	0.4053602
TNFAIP8L1	-0.185370904	0.334991453
TNFAIP8L3	-0.239335144	0.247049805
TNFRSF10A	0.053357091	0.746533995
TNFRSF10B	0.139074349	0.798603379
TNFRSF10C	0.134593183	0.514980897
TNFRSF10D	-1.004296138	0.195632868
TNFRSF11A	0.258327184	0.396240729
TNFRSF11B	0.142852171	0.845746964
TNFRSF12A	1.227921236	0.18666232
TNFRSF13B	0.282151763	0.033687405
TNFRSF13C	0.221352424	0.281492733
TNFRSF14	0.551467234	0.051442184
TNFRSF17	-0.058548752	0.608407823
TNFRSF18	0.105592718	0.510796592
TNFRSF19	1.326079555	0.00515055
TNFRSF1B	0.952909327	0.035197806
TNFRSF21	0.727581146	0.078312945
TNFRSF8	-0.094570816	0.397372097
TNFRSF9	-0.12848054	0.452673547
TNFSF10	0.866116171	0.019659172
TNFSF11	0.067423889	0.647135908
TNFSF12-TNFSF13	-0.272973579	0.341341981
TNFSF13B	0.615424421	0.011982868
TNFSF15	0.043488937	0.723824018
TNFSF18	-0.287989328	0.037997415
TNFSF4	0.043708394	0.821868934
TNFSF8	0.33367467	0.252127612
TNFSF9	-0.145611873	0.276915624
TNIK	0.350966079	0.471056924
TNIP2	0.426373757	0.034988055
TNK2	-0.021593906	0.959590568
TNKS	0.512218365	0.02119146
TNKS1BP1	0.718297072	0.043883128

TNKS2	-0.176925529	0.365289647
TNMD	0.082130784	0.712015348
TNNC1	-0.240892165	0.136250515
TNNC2	-0.220525054	0.104915574
TNNI1	-0.245063588	0.050515468
TNNT1	-0.159978766	0.279385306
TNNT2	-0.758507495	0.001454069
TNNT3	-0.005897647	0.968767026
TNP1	-0.197628554	0.10216072
TNP2	-0.109019362	0.288509325
TNPO1	0.557031074	0.027290512
TNPO2	-0.286775802	0.175391196
TNR	-0.229590591	0.86984044
TNRC6A	0.379153402	0.079673072
TNS1	0.433422529	0.209039712
TNS3	0.124484024	0.741478857
TNS4	0.009360497	0.936417439
TOGARAM1	-0.027099122	0.938370209
TOGARAM2	0.325103927	0.372787239
TOLLIP	-0.657596855	0.0104977
TOM1L1	0.07323455	0.927976981
TOMM20	-0.353449957	0.024734881
TOMM20L	-0.045996595	0.651747521
TOMM34	-0.632295921	0.135123268
TOMM40L	-0.0335529	0.877135519
TOMM7	-0.19215769	0.380367986
TOMM70	-0.149633067	0.308257437
TONSL	0.038133249	0.858696459
TOP1	0.249611951	0.164064396
TOP1MT	0.007010511	0.963152726
TOP2A	2.260383378	0.015500364
TOP2B	-0.133164325	0.604855555
TOP3A	0.437460674	0.020678144
TOP3B	0.32447074	0.077809769
TOPAZ1	-0.07735063	0.316480913
TOPBP1	0.860741947	0.00197588
TOR1A	-0.296658246	0.490425951
TOR1AIP2	-0.110524868	0.228009818
TOR2A	0.01780344	0.926890103
TOR3A	0.201409975	0.123229751
TOR4A	-0.30045348	0.265986967
TOX	-0.324564279	0.616478815
TOX2	-0.482578397	0.030307345
TP53	1.090091823	0.041202469
TP53I13	0.023798701	0.906262768
TP53I3	0.202508184	0.703268477
TP53INP1	0.846379096	0.079742987
TP53INP2	-0.876118041	0.087126301

TP53RK	0.017722433	0.941168506
TP53TG3	0.078598149	0.623230316
TP53TG5	-0.731369142	0.002552577
TP63	0.158001762	0.575906314
TP73	0.57459713	0.123801057
TPBG	-0.352671433	0.2150697
TPCN2	0.446656695	0.092265919
TPD52L1	-1.146629027	0.010751175
TPD52L2	-0.100780016	0.70248201
TPD52L3	-0.243932712	0.083132498
TPGS1	-0.358397258	0.008264854
TPGS2	-0.474391708	0.058517391
TPH1	-0.13923564	0.793328446
TPH2	-0.067643105	0.490269353
TPK1	-0.151030938	0.505816776
TPM1	-0.293398326	0.642440544
TPM2	0.33656728	0.674337869
TPO	-0.321935288	0.011982868
TPP2	0.406113516	0.038690557
TPPP	-2.384539953	0.006139844
TPPP2	-0.32939469	0.014785217
TPPP3	-0.156305796	0.853715871
TPR	0.629562618	0.001939871
TPRA1	0.245633709	0.117106423
TPRG1	0.721820708	0.000910583
TPRG1L	-1.536420769	0.001359112
TPRKB	0.252041036	0.247249456
TPRX1	-0.081126346	0.645797508
TPSAB1	-0.130849244	0.550364514
TPSG1	-0.105168466	0.466288438
TPST1	1.083367519	0.004362877
TPST2	-0.273986144	0.257676696
TPT1	0.268261953	0.234051376
TPTEP2-CSNK1E	0.11077144	0.511186861
TPX2	1.654325481	0.026115806
TRA2A	0.974947855	0.000352221
TRABD	0.301454361	0.165650312
TRADD	0.091811074	0.748619279
TRAF1	0.549097039	0.069483034
TRAF2	0.185872883	0.270286323
TRAF3	-0.309338796	0.093764507
TRAF3IP1	-0.033599344	0.861678768
TRAF3IP3	0.257247017	0.093356271
TRAF4	0.75335811	0.01710508
TRAF5	0.293936121	0.513684677
TRAF6	0.341417705	0.172458541
TRAF7	0.08664069	0.774118227
TRAIP	0.100947297	0.59150484

TRAK2	-0.114138934	0.581329553
TRAM1	0.679534896	0.022107213
TRAM1L1	-0.3170656	0.101504175
TRAP1	-0.075835169	0.696930692
TRAPPC10	-0.051822213	0.796166695
TRAPPC11	0.376305851	0.021168934
TRAPPC12	0.316976605	0.134390273
TRAPPC13	0.177197727	0.484357668
TRAPPC2L	-0.343331567	0.016426385
TRAPPC4	-0.000684436	0.99800589
TRAPPC6A	-0.104307612	0.566856555
TRAPPC8	0.247821245	0.262617666
TRARG1	-0.059518996	0.608118412
TRAT1	-0.005797179	0.958982209
TRDMT1	0.341537354	0.157140014
TRDN	-0.161871882	0.139113867
TREH	-0.071121003	0.452308799
TREM1	1.250972592	0.036907089
TREM2	1.069668315	0.038599609
TREML1	0.718942796	0.014096806
TREML2	-0.205466726	0.139376315
TREML4	-0.244284321	0.071584801
TRERF1	0.10894731	0.743883616
TREX1	0.045417555	0.791787659
TREX2	0.172792434	0.481196182
TRH	0.103129578	0.523095969
TRHR	-0.145948128	0.188690734
TRIAP1	0.143799735	0.667792293
TRIB1	0.350576495	0.242326524
TRIB2	1.858345547	0.004322736
TRIB3	0.121519735	0.592253696
TRIM10	-0.063501593	0.516550683
TRIM11	0.099564468	0.55530936
TRIM14	0.788623101	0.001169859
TRIM15	-0.20415182	0.243520829
TRIM16	0.000549212	0.998811869
TRIM2	-0.58729574	0.168721983
TRIM21	0.864889875	0.012397403
TRIM22	1.093692163	0.022796687
TRIM23	-0.41012217	0.09362029
TRIM24	0.930204919	0.00893861
TRIM25	1.16262853	0.000515458
TRIM29	-0.105513871	0.257376116
TRIM3	-0.139333434	0.618336681
TRIM31	0.080794533	0.313708077
TRIM32	0.064163052	0.855799407
TRIM34	0.22645757	0.277053669
TRIM35	0.038133254	0.844747122

TRIM36	0.119408594	0.655038746
TRIM37	-0.503220943	0.099427445
TRIM40	-0.031321845	0.838068964
TRIM41	-0.046529284	0.819106755
TRIM42	-0.033639165	0.753075946
TRIM43	-0.201347543	0.1153414
TRIM44	-0.442146787	0.029864398
TRIM45	0.349641035	0.169563691
TRIM47	1.066110297	0.005293384
TRIM48	0.209475705	0.201909681
TRIM5	0.362391448	0.308994733
TRIM50	-0.055105796	0.532562355
TRIM51	0.342574472	0.053996926
TRIM52	0.907860285	0.00044063
TRIM54	-0.312719934	0.054570871
TRIM55	-0.111343934	0.324631071
TRIM56	0.800809729	0.01347706
TRIM58	-0.719967876	0.007043312
TRIM59	-0.556053067	0.000627774
TRIM62	-0.100642455	0.745484819
TRIM63	-0.108282188	0.498407599
TRIM64B	0.126909401	0.596519233
TRIM67	-0.142578063	0.553100776
TRIM68	0.050100731	0.779043568
TRIM69	0.40528986	0.053351649
TRIM7	-0.034434752	0.746879258
TRIM73	0.391807971	0.458803777
TRIM74	0.542945397	0.125453352
TRIM8	-0.663319166	0.007026076
TRIM9	0.72490553	0.296839898
TRIML1	-0.088074817	0.382797194
TRIML2	0.064263969	0.623080098
TRIO	0.794119662	0.002267206
TRIP11	0.015034708	0.94671625
TRIP12	0.108128875	0.398558553
TRIP13	0.375642722	0.146354967
TRIP4	0.011678076	0.971953247
TRIP6	1.012084288	0.038529708
TRIT1	0.685304685	0.00097933
TRMO	0.466276852	0.006729601
TRMT1	0.210664486	0.304273853
TRMT10A	0.14064551	0.516550683
TRMT10B	0.737529436	0.000569843
TRMT10C	0.033384209	0.864502447
TRMT112	0.05944062	0.760637284
TRMT12	0.178144827	0.545964266
TRMT13	0.69617713	0.000888995
TRMT1L	0.219295629	0.327635999

TRMT2A	0.083816743	0.730609436
TRMT2B	0.401695403	0.12248967
TRMT44	0.17412257	0.195235967
TRMT5	0.531859608	0.019579731
TRMT6	0.274296756	0.166957481
TRMT61A	-0.067079845	0.719087761
TRMT61B	0.623537014	0.006623459
TRMT9B	0.21869305	0.613196297
TRMU	0.161182371	0.487841801
TRNT1	0.362940903	0.069184909
TROAP	0.432513884	0.118578456
TRPA1	-0.175526908	0.05383815
TRPC4	-0.241853846	0.687598582
TRPC5	-0.863931951	0.003157254
TRPC6	0.06157685	0.698320209
TRPC7	-0.180123498	0.123588719
TRPM2	-0.318341059	0.548410959
TRPM4	0.055192568	0.905111767
TRPM5	0.096600675	0.55483914
TRPM6	-0.153683425	0.192559557
TRPM7	0.365883966	0.062277687
TRPM8	0.438464803	0.117781718
TRPS1	0.600939769	0.000739219
TRPV1	0.238577469	0.350573936
TRPV2	0.066902516	0.772614814
TRPV4	-0.007442242	0.96051513
TRPV5	-0.317030008	0.008548221
TRPV6	-0.115275376	0.240533047
TRUB1	-0.507387527	0.051665185
TRUB2	-0.364156525	0.090944468
TSACC	-0.130103346	0.184229981
TSBP1	-0.054684522	0.570177706
TSC1	-0.046535489	0.861678768
TSC2	0.096125788	0.648765251
TSC22D1	-0.293617443	0.31574383
TSC22D2	-0.308210561	0.172220439
TSC22D3	0.257530774	0.176288675
TSC22D4	0.575738201	0.063030599
TSEN2	0.385879663	0.079338783
TSEN34	-0.191485836	0.295888621
TSFM	0.22014764	0.102143397
TSG101	-0.101035949	0.663753666
TSGA10	-0.122445245	0.618720598
TSGA10IP	-0.203978298	0.134077507
TSGA13	0.32126204	0.068196972
TSHB	0.030497312	0.75582128
TSHR	0.212990887	0.461034207
TSHZ1	0.18950185	0.523486054

TSHZ3	0.063630463	0.833863145
TSKS	-0.100049542	0.46525882
TSLP	-0.167799234	0.258448956
TSN	-0.097797788	0.665506283
TSNARE1	-0.124875967	0.471442677
TSNAXIP1	0.365238302	0.169800243
TSPAN1	-0.107089744	0.326518226
TSPAN10	-0.116440206	0.518818023
TSPAN12	0.525860024	0.072687194
TSPAN13	-0.4078424	0.309608278
TSPAN14	-0.231166834	0.529800532
TSPAN15	-0.220468193	0.576107076
TSPAN16	-0.100844963	0.327635999
TSPAN17	-0.108376932	0.684766999
TSPAN2	-0.655837466	0.106511131
TSPAN3	0.382190426	0.134840884
TSPAN31	0.336983768	0.18480698
TSPAN32	0.093323688	0.6794731
TSPAN33	0.293688818	0.458171833
TSPAN5	-0.588652173	0.018595078
TSPAN6	1.395387322	0.00183367
TSPAN7	-0.645945784	0.332155942
TSPAN8	-0.188903903	0.058315151
TSPAN9	0.224686189	0.551477865
TSPEAR	-0.119041819	0.259078223
TSPOAP1	-1.129806395	0.024305591
TSPY1	0.03074863	0.828704205
TSPY4	0.015013213	0.905209049
TSPYL2	-0.954542876	0.031528115
TSPYL4	-1.060798128	0.006438851
TSPYL5	-1.008108135	0.01622654
TSPYL6	-0.062580676	0.752870608
TSR1	0.339997434	0.173390717
TSR2	-0.534945306	0.025762855
TSR3	0.13841575	0.577063344
TSSC4	0.210198349	0.240255823
TSSK1B	-0.016091936	0.912859018
TSSK2	-0.097112948	0.563322948
TSSK3	0.547538083	0.139706069
TST	-0.215641407	0.542074826
TSTD2	0.663049638	0.007821977
TTBK2	-1.005538554	0.004300012
TTC1	-0.403092342	0.010960238
TTC12	0.396431512	0.184340094
TTC13	0.264766821	0.15015411
TTC16	-0.117686864	0.368464473
TTC17	0.599625978	0.004751113
TTC21B	0.560482008	0.01157297

TTC22	-0.113102958	0.515908172
TTC23L	0.013882382	0.942141247
TTC26	1.149975936	0.000783213
TTC27	0.263172812	0.234804462
TTC29	-0.000795394	0.996231571
TTC3	-0.101024716	0.640752919
TTC30A	0.145441266	0.623455279
TTC30B	0.351043113	0.102152834
TTC37	0.059763922	0.749350052
TTC38	0.554179003	0.076142408
TTC39B	0.182935937	0.307329861
TTC5	-0.085441735	0.653255633
TTC8	0.346155979	0.121696908
TTC9B	-0.934841411	0.008131149
TTC9C	0.359737917	0.173820085
TTF1	0.585938324	0.034355668
TTF2	0.552123722	0.013188958
TTI1	0.594794958	0.002183267
TTI2	0.061689721	0.752870608
TTK	0.697836798	0.098691561
TTL	-0.179451951	0.499195822
TTLL1	0.026380463	0.875122493
TTLL12	-0.502832017	0.018595078
TTLL13P	0.01005865	0.968064507
TTLL2	-0.203144679	0.056043915
TTLL3	0.304542964	0.121602925
TTLL4	0.942451908	0.000199223
TTLL6	0.192488179	0.731169603
TTLL7	-0.880060886	0.05100357
TTLL8	-0.019532098	0.847248255
TTLL9	1.086189603	0.016780753
TTPA	-0.245335654	0.616478815
TTPAL	0.067496367	0.772614814
TTR	-0.042542345	0.727094424
TTYH1	0.078292726	0.905495936
TTYH2	-0.557855961	0.316770683
TTYH3	0.553114742	0.084833849
TUB	-0.668370522	0.146945011
TUBA1C	0.063825294	0.709670769
TUBA3C	-0.325471763	0.2400032
TUBA3E	-0.133600565	0.318130405
TUBA4A	-0.236050228	0.062814672
TUBA8	-0.417551053	0.035113882
TUBAL3	-0.134890131	0.198182188
TUBB1	-0.185829915	0.067331974
TUBB2A	-0.532311452	0.12535762
TUBB4A	-2.309311472	0.057216668
TUBB4B	0.216791314	0.260960873

TUBB6	0.014553239	0.98447865
TUBD1	0.242428841	0.159585663
TUBE1	0.618555717	0.025920214
TUBG2	-0.508186442	0.165779238
TUBGCP3	0.612689236	0.013448084
TUBGCP4	-0.445645538	0.200198733
TUBGCP5	0.047438854	0.833320907
TUBGCP6	0.206096905	0.541941622
TUFT1	-0.50541164	0.197953923
TULP1	0.015612072	0.901235662
TULP2	-0.054629562	0.56276506
TULP3	0.116590226	0.678361974
TULP4	-1.301641705	7.88E-05
TUSC2	-0.186631666	0.291730861
TUSC3	-1.461101491	0.013034457
TUT1	0.199796341	0.462455166
TVP23B	0.181018238	0.229188829
TWF2	-0.210747498	0.456272891
TWIST1	0.156142493	0.596519233
TWNK	0.093711201	0.725826977
TWSG1	0.606482684	0.147237299
TXK	-0.084831287	0.512058508
TXLNA	0.846600545	0.006974901
TXLNB	1.433012641	0.001022378
TXLNG	-0.056045188	0.810771254
TXN	-0.217658925	0.566497648
TXN2	0.103657574	0.747273148
TXNDC11	0.46469772	0.083433085
TXNDC12	-0.002735844	0.993945285
TXNDC15	0.598112237	0.003832546
TXNDC17	0.359142873	0.218994449
TXNDC8	-0.163623564	0.133894177
TXNDC9	-0.035867194	0.871978886
TXNIP	1.277258709	0.005755113
TXNL4A	0.024336742	0.898425329
TXNRD2	-0.183927074	0.088731979
TYK2	0.773770976	0.004934247
TYMS	0.802308984	0.27243937
TYR	-0.017321487	0.867384023
TYRO3	-0.954090965	0.001203469
TYROBP	1.591665505	0.044170471
TYRP1	-0.240858528	0.113666836
TYSND1	0.002656593	0.986979433
TYW1	0.220998152	0.327016527
TYW3	-0.063929326	0.755992211
U2AF2	0.281594061	0.305375238
UACA	0.805753186	0.131501559
UAP1	0.171239664	0.679193729

UAP1L1	0.306950207	0.398445034
UBA2	0.320418545	0.071166412
UBA3	0.054426076	0.819044807
UBA5	0.47213968	0.013851792
UBA6	0.361218458	0.117781718
UBA7	1.619994575	0.000243076
UBAC1	0.360443769	0.033434046
UBALD1	-0.354408903	0.205446848
UBALD2	0.790574214	0.082556089
UBAP1L	-0.026448842	0.925006412
UBAP2	-0.455725615	0.041513123
UBASH3A	-0.104990729	0.322277035
UBASH3B	0.140450445	0.817589262
UBB	0.161729441	0.473175092
UBC	0.903317559	0.017336888
UBD	-0.367515503	0.504558025
UBE2A	0.478571645	0.113776379
UBE2C	0.893351478	0.022215458
UBE2D1	-0.285211462	0.354260306
UBE2D2	0.041866376	0.844347734
UBE2D3	0.255828194	0.08424441
UBE2D4	0.168786683	0.46525882
UBE2E1	0.45059333	0.030011024
UBE2E2	-0.319959664	0.279646787
UBE2F	0.186539676	0.23564844
UBE2G1	-0.306458982	0.161974398
UBE2G2	0.80716987	0.00168267
UBE2H	0.422941966	0.08362629
UBE2I	0.347116953	0.086022326
UBE2J1	0.041818128	0.904207344
UBE2K	-0.399941701	0.035378133
UBE2L6	0.578018702	0.008517827
UBE2M	-0.431615702	0.106926699
UBE2N	0.327793714	0.077079168
UBE2O	-0.19977099	0.329413397
UBE2Q2	0.252398695	0.37636203
UBE2R2	-0.190870399	0.39100251
UBE2T	0.473760759	0.412449482
UBE2U	-0.043995829	0.662139666
UBE2V1	0.032063283	0.804558867
UBE2V2	-0.137383174	0.352415623
UBE2W	-0.224374061	0.217801569
UBE3A	-0.21904551	0.317277549
UBE3B	0.02216579	0.871656435
UBE3D	-0.084242094	0.663942355
UBE4A	-0.075335143	0.670750072
UBFD1	0.257146846	0.220256792
UBL3	-0.024616359	0.963194792

UBL4A	-0.392664749	0.006580315
UBL4B	0.072528567	0.856723311
UBL5	-0.19451833	0.198206657
UBL7	-0.614486675	0.029922706
UBLCP1	-0.393773736	0.093377863
UBN1	0.383645341	0.04367646
UBOX5	0.170831102	0.309662927
UBP1	0.064574064	0.631524871
UBQLN1	-0.362687697	0.033392091
UBQLN2	-0.973273512	0.000511553
UBQLN3	-0.013765434	0.924763137
UBQLN4	-0.487300389	0.059333582
UBQLNL	0.766165927	0.021385313
UBR2	0.046523479	0.764778035
UBR3	-0.16622719	0.510565481
UBR4	0.204156591	0.142065598
UBR7	0.239501868	0.309255617
UBTD1	-0.208049953	0.300327655
UBTD2	0.89651983	0.009178714
UBXN1	0.210693003	0.076949066
UBXN10	-0.235955002	0.320594067
UBXN11	0.453878475	0.094448461
UBXN2A	0.388445998	0.174902958
UBXN4	0.296017145	0.008900957
UBXN7	0.093280861	0.650065932
UBXN8	0.42854753	0.028314249
UCHL1	-0.996500727	0.011147349
UCHL3	-0.710192961	0.020108663
UCK2	0.246442342	0.334898175
UCKL1	0.489616693	0.024405624
UCMA	0.112392584	0.407322211
UCN	0.341343793	0.075375848
UCN2	0.122478212	0.653672898
UCN3	-0.303579926	0.014933685
UCP1	-0.131542654	0.072245427
UCP2	0.414116932	0.105046429
UCP3	0.183477473	0.246846601
UFC1	-0.342291929	0.035192281
UFL1	0.221390957	0.193375679
UFM1	0.214419789	0.431369548
UFSP2	0.389958	0.137948025
UGCG	0.308513729	0.643997527
UGDH	0.607452295	0.202580776
UGP2	0.055411694	0.841759022
UGT1A9	-0.172227903	0.023701352
UGT2B10	-0.123934889	0.296863641
UGT2B11	-0.066546873	0.577204384
UGT2B15	-0.09798429	0.240723714

UGT2B17	-0.225545163	0.059227366
UGT2B4	-0.055315295	0.576299851
UGT2B7	-0.059430213	0.558876753
UGT3A1	0.035148969	0.734926031
UGT3A2	-0.050906119	0.677199137
UGT8	-1.277957272	0.203569816
UHMK1	-0.361003204	0.081390523
UHRF1BP1	0.605762222	0.006663931
UIMC1	-0.93775613	0.18647246
ULBP1	-0.133401082	0.751015759
ULBP2	-0.120727492	0.64718882
ULBP3	-0.453965726	0.347293069
ULK1	-0.451172526	0.007761866
ULK2	-0.718019118	0.013855945
ULK4	0.477148787	0.02575868
UMOD	0.145754485	0.317101004
UMODL1	-0.124521633	0.249578491
UNC119	-0.095580783	0.639693527
UNC45B	0.028650557	0.787164211
UNC50	0.477230821	0.003409275
UNC5B	-0.115894155	0.833863145
UNC5C	-1.223066832	0.006417812
UNC5CL	0.287060323	0.091068169
UNC5D	-1.225470403	0.030924492
UNC79	-1.36687079	0.06828476
UNC93A	-0.06501635	0.566497648
UNC93B1	0.147147288	0.511285816
UNCX	-0.129653592	0.404656768
UNG	0.100435918	0.697558282
UPB1	-0.107784415	0.238517657
UPF1	-0.01296606	0.956825161
UPF2	0.02587849	0.934842248
UPF3A	0.579198646	0.051261644
UPF3B	-0.036039006	0.833863145
UPK1A	-0.089455219	0.501492531
UPK1B	-0.099388429	0.455626029
UPK2	-0.146716293	0.340299987
UPK3A	-0.179732648	0.18454753
UPP1	0.359755342	0.298658542
UQCC1	0.165941971	0.574537564
UQCC2	0.287944404	0.209059496
UQCC3	-0.108731774	0.593292976
UQCR10	-0.73180465	0.006691815
UQCR11	0.190559685	0.457590223
UQCRC1	-0.329280249	0.065856573
UQCRC2	-0.329267134	0.057341251
UQCRFS1	-0.094109226	0.839170012
UQCRH	0.205540337	0.209830984

UQCRQ	0.173585635	0.064594628
URB1	0.936118342	0.004064451
URI1	0.103477594	0.823597248
UROS	-0.532513069	0.003041398
USB1	-0.058963798	0.782419818
USE1	0.355912888	0.035047058
USF1	0.511757836	0.004334512
USF2	-0.006913164	0.984417114
USH1C	-0.100144341	0.372796875
USH1G	0.001022241	0.995181038
USHBP1	-0.008048522	0.963152726
USO1	0.1151055	0.55101437
USP10	0.062241692	0.839170012
USP11	-0.596573004	0.024485612
USP12	-0.250346776	0.283748511
USP13	0.227932288	0.254613036
USP14	-0.307177005	0.227472354
USP17L2	-0.226049611	0.166903888
USP17L23	-0.27612931	0.026757026
USP17L5	-0.53850502	0.201016628
USP18	1.318749271	0.001009748
USP2	-0.127645023	0.739277771
USP20	-0.095194382	0.577680191
USP25	-0.247809722	0.319022762
USP26	-0.04972257	0.577168524
USP28	0.171668215	0.420259847
USP29	-0.118388283	0.334004274
USP30	-0.27843377	0.21369076
USP31	-0.545886819	0.045979268
USP32	0.04591362	0.853607455
USP33	0.004190501	0.98640958
USP34	0.074683098	0.712625147
USP36	0.221583782	0.424543725
USP37	0.362753556	0.05199819
USP38	0.051949151	0.854751842
USP4	0.360582794	0.026631425
USP40	0.582237543	0.013830022
USP43	0.575542504	0.258448956
USP44	-0.249779558	0.025834777
USP46	-0.621329133	0.035809715
USP47	0.109687945	0.567374527
USP48	0.217140684	0.325538652
USP49	0.007885471	0.968767026
USP50	0.023156168	0.872447647
USP51	-0.083876023	0.584436904
USP54	-0.224257464	0.684047451
USP6	-0.094691665	0.53121255
USP6NL	0.270356825	0.156600389

USP7	0.099502244	0.48715344
USPL1	0.371022909	0.134390273
UST	1.153972853	0.002824951
UTF1	-0.130982477	0.132468097
UTP11	0.310392937	0.134776399
UTP14A	-0.027145757	0.926926533
UTP15	0.63322369	0.041652485
UTP20	0.767207105	0.007346219
UTP23	-0.000441972	0.998667636
UTP25	-0.178060268	0.293521592
UTP3	-0.026583507	0.896420697
UTP4	0.060485486	0.862276842
UTP6	0.761664124	0.001175955
UTS2	-0.080875438	0.36134822
UTS2B	-0.2088575	0.084711293
UTS2R	-0.428686187	0.044616248
UVSSA	0.469021111	0.019662203
UXT	0.076902917	0.737832072
VAC14	0.112923994	0.61296361
VAMP1	-1.032258857	0.004965795
VAMP3	-0.424292654	0.23852602
VAMP4	-0.040883064	0.937340175
VAMP5	0.288582656	0.352268887
VAMP7	-0.008819164	0.977625155
VAPA	0.003440779	0.986230241
VAPB	-0.315751778	0.041829023
VASH1	0.986413483	0.000389052
VASN	-0.24979855	0.50539062
VASP	0.163632723	0.776786373
VAT1L	-0.495005597	0.575839088
VAV1	0.929452898	0.007821977
VAX1	-0.114681501	0.752269641
VAX2	0.516861136	0.074241257
VBP1	-0.129025168	0.556261656
VCAM1	1.346180509	0.018844974
VCAN	1.620483387	0.010927461
VCL	0.020413667	0.98388747
VCP	0.007961184	0.98200225
VCPIP1	-0.055674718	0.832518473
VCPKMT	-0.15523531	0.578589007
VCX2	-0.10587119	0.253356951
VCX3A	0.103428483	0.514051025
VCY	0.052277992	0.668746633
VDAC1	0.070683739	0.736257563
VDAC3	0.014650309	0.941984856
VEGFC	-0.227323213	0.46925675
VENTX	0.189279957	0.452537757
VEPH1	-0.215349369	0.547138402

VEZT	-0.254722458	0.073113834
VGF	-0.472372478	0.351918897
VGLL1	-0.115445654	0.272282766
VGLL2	0.080178048	0.729007438
VGLL3	-0.655207004	0.299604752
VHL	0.401153091	0.015989621
VHLL	-0.113887857	0.306813719
VIL1	-0.089744512	0.375950158
VILL	-0.149453224	0.309377298
VIM	1.446843345	0.004919031
VIP	-0.389408602	0.062573611
VIPAS39	0.263964835	0.243047488
VIPR2	0.295385734	0.609173141
VIRMA	0.04093172	0.853607455
VIT	0.200215431	0.440419328
VKORC1	-0.074978785	0.792979636
VKORC1L1	-0.044176455	0.884394993
VLDLR	-0.945114671	0.019575959
VMO1	-0.104123379	0.376184182
VMP1	-0.190167804	0.232654739
VN1R1	0.46204716	0.119714903
VN1R2	0.185823397	0.151839878
VN1R4	-0.012711913	0.886463258
VNN1	0.059487265	0.691193069
VNN2	0.175550175	0.163412734
VOPP1	0.481830546	0.160515731
VPREB1	0.004304315	0.98146514
VPREB3	0.080727487	0.515958709
VPS11	-0.094381803	0.660708583
VPS13A	-0.156089048	0.595291621
VPS13B	0.553920124	0.002165273
VPS13D	0.050029223	0.840907532
VPS16	0.053080924	0.796166695
VPS18	-0.304667616	0.288939179
VPS25	0.150377259	0.658038853
VPS26A	-0.53781789	0.005365281
VPS26B	0.123882276	0.57259631
VPS26C	0.082093787	0.666171783
VPS29	0.481731358	0.03315189
VPS33A	0.179962157	0.39532134
VPS35	-0.375410094	0.162819818
VPS35L	-0.227728004	0.141920231
VPS36	0.419007461	0.055158612
VPS37A	-0.032638021	0.871846165
VPS37B	0.373640901	0.107059158
VPS37C	0.221206422	0.123939793
VPS41	0.174667428	0.327149371
VPS4A	-0.297769047	0.226887829

VPS4B	0.202767059	0.221465287
VPS51	-0.228807407	0.197475137
VPS54	0.934326047	0.000796826
VPS72	0.062563765	0.789946329
VPS9D1	0.213908623	0.483641731
VRK1	0.468076918	0.099889742
VRK2	-0.649629353	0.017162009
VRK3	0.448046651	0.045552482
VRTN	-0.140206139	0.169782421
VSIG10	0.306503238	0.191832919
VSIG2	-0.009710565	0.947707242
VSNL1	-1.051622326	0.001225213
VSTM1	0.072925901	0.573767576
VSTM2A	-1.839436965	0.066142214
VSTM2L	-1.055056577	0.004734877
VSTM5	-0.371215882	0.029037894
VSX2	-0.303059533	0.019659172
VTA1	-0.221079685	0.576779548
VTI1A	-0.249058297	0.122356355
VTI1B	-0.296774064	0.12154063
VTN	-0.04743594	0.712803392
VWA1	-0.135328006	0.655803928
VWA2	0.006253297	0.973630919
VWA5A	0.444719303	0.138476976
VWA7	-0.390426665	0.056133238
VWC2	-0.170799044	0.634698732
VWC2L	-0.087219612	0.821001661
VWCE	0.404774749	0.108120642
VWF	0.851494028	0.081384285
VXN	-0.662239249	0.267609207
WAC	-0.169967648	0.379563994
WAPL	-0.037034535	0.832518473
WARS2	0.620640823	0.009319251
WAS	0.792513432	0.019571398
WASF1	-1.343017652	0.007043312
WASF3	-0.730102845	0.174004226
WASH6P	-0.036033048	0.845746964
WASHC2C	-0.447421066	0.0883666
WASHC3	0.194433463	0.257228425
WASHC4	0.629148514	0.003140901
WASHC5	-0.085960967	0.734048692
WASL	-0.033461837	0.918837703
WBP1	0.485328876	0.01889564
WBP11	-0.132691282	0.284624449
WBP1L	0.002948068	0.992651559
WBP2	-0.706674185	0.016616103
WBP2NL	-1.489963157	0.123801057
WBP4	-0.147104988	0.626696492

WDCP	0.485232019	0.037372626
WDFY1	0.122908034	0.501608696
WDFY2	0.417466431	0.09818426
WDFY3	0.023210063	0.924366909
WDHD1	0.605502613	0.129536206
WDPCP	0.251906547	0.116731387
WDR1	0.222342947	0.654912404
WDR11	0.110864128	0.59150484
WDR12	0.119323122	0.481196182
WDR13	0.080526179	0.697520607
WDR17	-0.870906373	0.175213777
WDR18	0.063808511	0.732232421
WDR19	0.724846019	0.002733133
WDR20	0.000784425	0.997824485
WDR24	-2.797947419	0.001290677
WDR25	0.130214223	0.376954905
WDR27	0.723083223	0.004240643
WDR3	0.77076812	0.021376611
WDR33	0.33181997	0.065219541
WDR36	0.606821819	0.030250358
WDR37	-0.8051141	0.002287614
WDR41	0.155797051	0.427120463
WDR43	0.420110022	0.054240566
WDR44	-0.526892146	0.0613912
WDR45	0.116043344	0.515432943
WDR45B	0.393552966	0.037997415
WDR48	-0.056367283	0.765319466
WDR49	1.523484678	0.002287614
WDR5	0.312010263	0.238793091
WDR53	0.487478406	0.006576658
WDR54	0.367224534	0.026701201
WDR55	0.479296141	0.049276081
WDR5B	0.794021781	0.003420534
WDR6	1.029463317	0.000331368
WDR61	0.341627201	0.096106171
WDR64	-0.001434934	0.989421806
WDR7	-0.403336992	0.164376137
WDR70	0.32820358	0.008140033
WDR72	-0.069674672	0.414473871
WDR73	0.640052301	0.003132413
WDR74	0.088518192	0.618720598
WDR75	0.364083565	0.057828053
WDR76	0.996880375	0.006496089
WDR77	0.369753947	0.06169183
WDR81	0.304457231	0.084821873
WDR82	0.00084817	0.996621431
WDR86	-0.094492607	0.573751372
WDR88	0.067681464	0.620718416

WDR90	0.746657968	0.003011379
WDR91	0.467686792	0.138304867
WDR92	-1.105404949	0.006880123
WDR93	0.144161081	0.373009992
WDTC1	-0.519229826	0.006185744
WEE1	1.779443602	0.01131857
WFDC1	-0.659913979	0.024478272
WFDC10A	-0.105707518	0.318895907
WFDC10B	-0.074221378	0.790705482
WFDC11	-0.072830129	0.54414515
WFDC12	-0.075865635	0.521837798
WFDC13	-0.10705793	0.565587548
WFDC2	-0.210341726	0.265363438
WFDC3	-0.099540512	0.512450358
WFDC5	-0.060100871	0.643533369
WFDC6	-0.059264747	0.520975945
WFDC8	0.041231901	0.656466133
WFDC9	-0.058032618	0.461109885
WFIKKN1	-0.117790006	0.516380268
WFIKKN2	-0.110323316	0.568950838
WFS1	-1.165445861	0.000287589
WHRN	0.056226076	0.892887186
WIF1	-2.163463562	0.008058515
WIPF1	0.927149221	0.011358426
WIPF2	-0.25674269	0.338892631
WIPI1	0.064038065	0.909515033
WIPI2	0.539946913	0.004322736
WLS	1.005841558	0.000627774
WNK1	-0.19242317	0.414394125
WNK2	-1.503066563	0.005947029
WNK3	0.755919102	0.018230654
WNK4	-0.333187833	0.42505501
WNT1	-0.144980829	0.317786978
WNT10A	0.032828405	0.842367499
WNT10B	-0.571459436	0.001544789
WNT11	-0.127290689	0.507927681
WNT16	0.354216832	0.52275846
WNT2	-0.109601536	0.396542579
WNT2B	-0.189318711	0.553100776
WNT3	-0.286765803	0.284611629
WNT3A	0.068051123	0.674391113
WNT5A	0.410851255	0.459874564
WNT5B	-0.149602492	0.611795648
WNT6	-0.061123709	0.653942911
WNT7A	-0.087236467	0.6712973
WNT7B	-0.267357925	0.159828897
WNT8A	-0.011769089	0.930110116
WNT8B	-0.130390355	0.318175113

WNT9A	-0.188443293	0.114995133
WNT9B	-0.296343766	0.027935576
WRAP53	0.261909487	0.052571981
WRAP73	0.283884742	0.033620037
WRN	0.715905465	0.003613651
WRNIP1	0.053019601	0.782230826
WSB1	0.995359541	0.009323954
WSB2	-1.00619786	0.000457836
WSCD2	-0.470695425	0.05355385
WTAP	0.586206054	0.015663749
WWC1	-0.331811435	0.187190382
WWC2	0.019672079	0.956825161
WWC3	-0.080108662	0.745870016
WWP1	0.047253247	0.895198958
WWP2	0.135448842	0.500451938
WWTR1	0.664673057	0.059191332
XAB2	-0.141248914	0.448762755
XAF1	1.615889739	0.004149839
XAGE2	-0.184168818	0.029094312
XAGE3	-0.219467129	0.133924264
XAGE5	-0.198296759	0.115985915
XBP1	0.445284848	0.147608175
XCL1	0.06541749	0.724287924
XCR1	-0.087877337	0.635955798
XDH	0.022358103	0.855799407
XIAP	0.462029666	0.014510731
XIRP1	-0.318751277	0.316898088
XK	-0.422322607	0.034227184
XKR3	-0.060179731	0.560642078
XKR4	-0.884824689	0.136352325
XKR5	-0.023350877	0.837559998
XKR6	0.15469952	0.62641977
XKR8	-0.124170852	0.535923979
XKRX	0.098322427	0.489634549
XPC	0.13390309	0.509363127
XPNPEP1	0.002227287	0.993666428
XPNPEP2	0.239957446	0.149621879
XPNPEP3	0.147562833	0.372787239
XPO4	0.184558482	0.340713923
XPO5	0.552742472	0.009287172
XPO7	0.290033653	0.139868898
XPOT	0.411297875	0.103147355
XPR1	0.524413657	0.011530566
XRCC1	0.250068013	0.141921844
XRCC3	0.209888505	0.1627352
XRCC4	0.490142567	0.037871129
XRCC5	-0.008611591	0.970066069
XRCC6	-0.058902553	0.807436219

XRN1	0.522881275	0.005091231
XRN2	0.585077626	0.023410247
XXYLT1	0.0836313	0.727094424
XYLB	0.387697417	0.100881592
XYLT1	0.029405511	0.957556819
YAE1	0.860313369	0.006185744
YBX1	0.749435857	0.012264957
YBX2	0.119212353	0.628063601
YBX3	1.218371251	0.000851026
YEATS2	0.482617788	0.029423198
YEATS4	0.53912301	0.068213095
YES1	0.29328488	0.456362542
YIPF2	-0.026618557	0.942091567
YIPF3	0.036910114	0.888379819
YIPF4	0.150392582	0.41820216
YIPF6	0.126711679	0.648771023
YIPF7	0.156332678	0.168306375
YJEFN3	-0.165412709	0.642440544
YJU2	0.365122542	0.042824008
YKT6	0.816048604	0.000962015
YME1L1	-0.262766769	0.320805847
YPEL1	-0.132206942	0.581700499
YPEL3	-0.680799879	0.081321467
YPEL4	-0.464420406	0.052715555
YRDC	0.187130715	0.485938514
YTHDC1	0.278979077	0.235805814
YTHDC2	0.411470355	0.01414208
YTHDF3	0.145616839	0.409052598
YWHAB	-0.221168042	0.177165745
YWHAE	0.175628053	0.338225296
YWHAG	-1.010296536	0.010544328
YWHAH	-0.356495691	0.283835422
YWHAQ	-0.173012385	0.278913814
YWHAZ	-0.583703912	0.001056288
YY1	0.239446252	0.168110036
YY1AP1	0.54255013	0.019659172
ZACN	-0.118998185	0.333526976
ZADH2	-0.057798978	0.849500029
ZAN	0.024923463	0.816408453
ZAP70	-0.118718243	0.214621434
ZAR1	0.151961027	0.24372963
ZAR1L	-0.065451969	0.444579569
ZBED2	-0.03342349	0.785071735
ZBED3	0.121568889	0.699556828
ZBED5	0.210029359	0.406099532
ZBED6	0.868371914	0.004981702
ZBED6CL	0.001218266	0.995181038
ZBED8	0.461165602	0.037857472

ZBED9	-0.214294829	0.21448759
ZBP1	-0.058845314	0.658719643
ZBTB10	0.572984641	0.017184737
ZBTB11	-0.02484149	0.910966557
ZBTB16	0.060199694	0.922718328
ZBTB17	0.162821092	0.389435147
ZBTB2	0.642143243	0.012312641
ZBTB21	0.277741238	0.18279964
ZBTB25	0.529873499	0.043037935
ZBTB26	0.193115214	0.503583473
ZBTB3	-0.039337825	0.864502447
ZBTB32	0.101590106	0.485880843
ZBTB34	0.090721971	0.556950582
ZBTB41	-0.037672247	0.878631732
ZBTB42	0.292038093	0.537033283
ZBTB43	0.137798653	0.384578677
ZBTB44	-0.257574994	0.371378038
ZBTB45	-0.214913555	0.326841159
ZBTB46	-0.178069219	0.534296745
ZBTB47	-0.211764468	0.320347182
ZBTB48	0.395795045	0.027960552
ZBTB49	0.341607667	0.028452842
ZBTB5	0.251432409	0.227844734
ZBTB6	0.118159374	0.725919768
ZBTB7A	-0.518507329	0.117781718
ZBTB8OS	0.345077845	0.03718731
ZBTB9	0.517160992	0.075352976
ZC2HC1A	0.045929252	0.904792329
ZC2HC1C	-0.059780789	0.611063981
ZC3H10	0.143184887	0.228478318
ZC3H11A	0.57027695	0.001454069
ZC3H12A	0.002203404	0.994849586
ZC3H12D	-0.17676423	0.327179869
ZC3H13	-0.517932897	0.02100609
ZC3H14	0.173502289	0.289876417
ZC3H15	0.045581892	0.753752527
ZC3H18	0.205477197	0.289646583
ZC3H3	0.192908283	0.373969966
ZC3H6	-0.224708389	0.588301474
ZC3H7A	0.636805368	0.001301336
ZC3H7B	-0.828929615	0.000962015
ZC3H8	0.160117178	0.523015082
ZC3HAV1	1.290665068	0.000164034
ZC3HAV1L	0.740557714	0.063760341
ZC3HC1	0.554241452	0.009912086
ZCCHC10	0.172397247	0.483366908
ZCCHC12	-0.494447321	0.082058452
ZCCHC13	-0.072281647	0.505816776

ZCCHC14	0.113618594	0.582804387
ZCCHC17	0.001317957	0.995953997
ZCCHC24	-0.127231836	0.776400425
ZCCHC8	0.854741112	0.000855276
ZCCHC9	0.700989779	0.000569843
ZCRB1	-0.403882296	0.066664283
ZCWPW1	0.359451794	0.052523699
ZDBF2	-0.8247036	0.116232759
ZDHHC11	-0.33398592	0.274294555
ZDHHC12	-0.03338745	0.914495458
ZDHHC13	-0.230296777	0.323792532
ZDHHC14	0.133921593	0.670187191
ZDHHC15	1.445442527	0.002062218
ZDHHC16	-0.036583092	0.905209049
ZDHHC17	0.128308383	0.577576584
ZDHHC18	0.177049884	0.347223044
ZDHHC2	-0.385460398	0.356472461
ZDHHC20	-0.684781703	0.01197672
ZDHHC21	-0.236279683	0.472122894
ZDHHC22	-0.420716463	0.350067341
ZDHHC23	0.140462143	0.629566427
ZDHHC24	0.079190374	0.754543337
ZDHHC3	0.023735057	0.91394031
ZDHHC5	-0.094000679	0.70495706
ZDHHC6	0.331609917	0.034988055
ZDHHC8	-0.119400307	0.544520373
ZDHHC9	-0.220630994	0.549312728
ZEB1	0.836880821	0.00183367
ZEB2	0.051488778	0.901052359
ZFAND1	-0.256536515	0.327370792
ZFAND2A	0.243054485	0.25431304
ZFAND2B	0.018297687	0.904256812
ZFAND3	0.571863244	0.016817157
ZFAND4	0.144922111	0.552352587
ZFAT	0.006475702	0.971953247
ZFC3H1	0.450295389	0.022050211
ZFHX3	0.60209415	0.019310611
ZFHX4	0.749676664	0.073507284
ZFP1	-0.043321496	0.907973851
ZFP2	0.353157297	0.042346382
ZFP28	-0.153613482	0.584478839
ZFP3	0.245876275	0.457590223
ZFP36L2	1.969717946	0.000269441
ZFP37	0.425319566	0.07435448
ZFP41	0.232050475	0.125453352
ZFP42	-0.107951141	0.295585953
ZFP64	0.164258617	0.243238732
ZFP69	0.342795323	0.230284914

ZFP69B	0.072002582	0.830359479
ZFP82	0.210298084	0.387859918
ZFPM1	-0.130359249	0.529207785
ZFPM2	-0.062115126	0.6712973
ZFR	-0.021294076	0.911351241
ZFX	0.220673847	0.218311247
ZFY	0.33365353	0.266622752
ZFYVE1	0.101009557	0.680593066
ZFYVE16	0.212637402	0.5972046
ZFYVE26	0.518709855	0.004059517
ZFYVE27	-0.356640616	0.019659172
ZFYVE28	-0.31120181	0.105539538
ZG16B	-0.185300287	0.431910829
ZGPAT	0.376750732	0.15887854
ZGRF1	0.446139809	0.130844614
ZHX2	0.30789152	0.409564724
ZHX3	0.407672911	0.11456237
ZIC1	0.617808459	0.096296467
ZIC2	-0.337683188	0.382513211
ZIC3	0.124666102	0.311113182
ZIC5	-0.128290886	0.686976208
ZIK1	0.067309148	0.690801599
ZIM2	-0.883827121	0.14755208
ZIM3	-0.167118841	0.15962126
ZKSCAN1	0.194504627	0.320347182
ZKSCAN4	0.166132226	0.257587266
ZKSCAN5	0.460816385	0.013182136
ZKSCAN7	0.225616437	0.360043599
ZMAT2	0.195093431	0.121145569
ZMAT3	-0.305250398	0.529207785
ZMAT4	-0.842582325	0.024235737
ZMAT5	0.007891477	0.981452955
ZMIZ1	-0.030719918	0.929501569
ZMYM1	0.625628629	0.011890471
ZMYM2	-0.060421913	0.734958082
ZMYM3	0.102609742	0.544236806
ZMYM4	0.468513747	0.008414497
ZMYM6	-0.025554756	0.847248255
ZMYND10	0.252066585	0.25431304
ZMYND11	-0.543535654	0.016997895
ZMYND12	0.066693286	0.739670021
ZMYND19	0.264345287	0.050284897
ZMYND8	0.173616972	0.329369557
ZNF10	0.40182597	0.059547219
ZNF100	0.863764664	0.006139844
ZNF101	0.388409177	0.021385313
ZNF106	-0.636241676	0.029686684
ZNF107	1.115003403	0.00227739

ZNF114	0.446744232	0.058992523
ZNF117	1.400881909	0.000511553
ZNF132	0.556255204	0.047389638
ZNF133	0.029952115	0.868709798
ZNF134	0.394413158	0.133087063
ZNF135	0.173611045	0.486107177
ZNF136	0.45226266	0.011313988
ZNF14	0.06015673	0.775232596
ZNF141	0.906981739	0.003951862
ZNF142	0.412267731	0.058479765
ZNF143	0.247192406	0.176006693
ZNF148	0.04329291	0.837559998
ZNF154	0.010758033	0.981452955
ZNF157	-0.039928753	0.859770224
ZNF160	0.580868341	0.009032828
ZNF169	0.124316581	0.456272891
ZNF17	0.342236448	0.150717605
ZNF174	0.139524179	0.409735831
ZNF180	0.186820619	0.367065883
ZNF184	-0.017921288	0.951240846
ZNF189	-0.108219206	0.651014997
ZNF19	0.263296955	0.058755115
ZNF197	0.317133823	0.076022756
ZNF2	0.143216775	0.394423915
ZNF200	0.254457327	0.203243282
ZNF202	0.213255509	0.108681693
ZNF205	0.26375382	0.0883666
ZNF207	0.545785026	0.002068027
ZNF208	-0.223055103	0.648779833
ZNF212	0.678676004	0.005737837
ZNF214	0.166760643	0.375141671
ZNF215	-0.345177937	0.073507284
ZNF217	1.01166323	0.00282173
ZNF22	0.464116803	0.107974874
ZNF221	0.4364108	0.064765698
ZNF222	-0.145903545	0.380010094
ZNF223	0.332911374	0.210806938
ZNF224	0.265508805	0.234483133
ZNF225	0.515880054	0.010139305
ZNF226	0.479192713	0.082736051
ZNF227	0.659297193	0.000739219
ZNF232	0.235095516	0.281678876
ZNF233	-0.123744102	0.520268919
ZNF235	0.85142171	0.004676856
ZNF236	0.28315654	0.20012355
ZNF239	0.1275447	0.549214914
ZNF24	0.167734838	0.635749683
ZNF248	-0.139965677	0.651980684

ZNF25	-0.689101362	0.136317858
ZNF250	0.136426524	0.396343062
ZNF253	0.306742467	0.348320692
ZNF254	0.439656191	0.110071198
ZNF256	0.213810197	0.365836266
ZNF257	-0.121678978	0.733059533
ZNF260	0.374709868	0.316349868
ZNF263	0.096110756	0.664232577
ZNF266	1.008963995	0.000961198
ZNF267	0.299652274	0.177165745
ZNF268	0.401144771	0.11511463
ZNF273	-0.215972958	0.290358098
ZNF274	0.160132897	0.452102972
ZNF277	0.06989225	0.734926031
ZNF280A	-0.000339169	0.99800589
ZNF280B	-0.075189406	0.780766159
ZNF280C	0.643734544	0.025808904
ZNF280D	0.05418652	0.855799407
ZNF281	-0.180346133	0.548787288
ZNF282	0.605915467	0.006974901
ZNF283	0.597257156	0.011757324
ZNF286A	0.3466376	0.353442814
ZNF287	0.29631841	0.192059218
ZNF296	-0.11602897	0.57259631
ZNF3	0.624492512	0.004777671
ZNF302	0.050206381	0.902244732
ZNF304	0.135698173	0.522821115
ZNF322	0.173592863	0.560024501
ZNF324	-0.099135288	0.575480713
ZNF324B	-0.21517585	0.244737991
ZNF326	0.266511175	0.283314784
ZNF329	0.121286942	0.519650217
ZNF330	0.302360873	0.090417782
ZNF331	0.662510656	0.06581677
ZNF333	0.497469473	0.006185744
ZNF334	0.921080302	0.006652649
ZNF335	0.06692968	0.725355865
ZNF337	0.477038092	0.021714154
ZNF34	0.656601943	0.018305386
ZNF341	0.017585168	0.932046426
ZNF343	0.213097425	0.513693168
ZNF346	0.178502499	0.350077294
ZNF35	0.652503069	0.006974901
ZNF350	0.205957953	0.286021893
ZNF354A	0.443190652	0.029925492
ZNF354B	0.260904083	0.020496695
ZNF354C	0.791397994	0.007840793
ZNF358	-0.758843661	0.001678442

ZNF362	0.201374573	0.3425752
ZNF365	-0.477368814	0.009312966
ZNF366	0.007860475	0.975084351
ZNF382	-0.51779401	0.137352678
ZNF383	0.522415009	0.023504034
ZNF384	0.508396407	0.003384695
ZNF385A	-0.105444789	0.845289305
ZNF385B	-1.237897956	0.021385313
ZNF385D	-0.161838371	0.149721131
ZNF391	0.065510619	0.745484819
ZNF394	0.551458393	0.004625182
ZNF395	0.24743564	0.447321521
ZNF396	0.048243923	0.739736703
ZNF397	0.362213312	0.12421724
ZNF398	0.336468292	0.061006569
ZNF407	0.07828514	0.708608221
ZNF408	-0.119852305	0.342374585
ZNF41	0.223199602	0.203783861
ZNF410	0.127324541	0.597842196
ZNF416	-0.013564935	0.960926694
ZNF417	0.688302327	0.027236942
ZNF420	-0.043235545	0.908526057
ZNF423	-0.304424547	0.361019878
ZNF425	0.292944136	0.250506267
ZNF426	0.739351449	0.001485471
ZNF428	0.54774439	0.023168931
ZNF429	0.482511958	0.19680363
ZNF430	0.330431015	0.081772108
ZNF431	1.008877511	0.000387503
ZNF432	0.623284621	0.023949051
ZNF439	0.605507158	0.004412189
ZNF440	0.621085666	0.014596147
ZNF442	0.091625673	0.439613622
ZNF444	-0.174535061	0.350866255
ZNF445	0.440273335	0.016325061
ZNF446	0.210113467	0.143899893
ZNF449	0.698386155	0.005755113
ZNF460	0.364521263	0.063351752
ZNF461	0.23065733	0.277053669
ZNF462	1.149519138	0.000393501
ZNF467	-0.23480989	0.217503543
ZNF470	0.184122123	0.493225752
ZNF471	0.102636145	0.620075563
ZNF474	0.734182269	0.059750851
ZNF48	0.005695625	0.974469226
ZNF480	0.460410984	0.126004782
ZNF485	0.148325112	0.451027975
ZNF490	0.076526265	0.673702005

ZNF491	0.210476413	0.351536825
ZNF493	0.863882335	0.008614407
ZNF496	0.289689959	0.099126975
ZNF502	1.035083868	0.003133494
ZNF503	0.061066372	0.742188902
ZNF510	0.242978028	0.331569694
ZNF511	-0.259888861	0.06143058
ZNF513	0.533839681	0.011477141
ZNF514	0.284140836	0.132468097
ZNF517	0.109347275	0.477434776
ZNF521	0.785552446	0.021385313
ZNF524	-0.191403268	0.210990029
ZNF528	0.278252508	0.190374885
ZNF532	0.790146128	0.001917421
ZNF543	0.116500825	0.629200991
ZNF544	0.313026886	0.405907035
ZNF546	0.359218183	0.012552229
ZNF547	0.365418015	0.040927029
ZNF549	0.289954611	0.077279311
ZNF550	0.244487679	0.072620438
ZNF551	0.575348016	0.003733005
ZNF552	-0.003381965	0.98042003
ZNF555	0.44469878	0.093631895
ZNF556	0.100429819	0.538033419
ZNF557	0.378707406	0.196209899
ZNF558	1.189145138	0.004322736
ZNF560	0.257204989	0.560024501
ZNF565	-0.099720448	0.576345689
ZNF567	0.413599391	0.14496164
ZNF569	0.107365835	0.596519233
ZNF57	0.067997326	0.568950838
ZNF570	0.090667328	0.710390583
ZNF571	0.377976204	0.035047058
ZNF572	0.298361841	0.05796386
ZNF573	0.117399334	0.392870911
ZNF574	-0.149189913	0.291829223
ZNF575	-0.013598055	0.927776777
ZNF576	0.031051514	0.875962569
ZNF577	0.645760629	0.019659172
ZNF578	0.162314534	0.476541458
ZNF579	-0.183781775	0.3423464
ZNF582	0.426237805	0.10943235
ZNF583	-0.156980928	0.24685101
ZNF584	0.301534813	0.274357141
ZNF585A	0.11284957	0.606203239
ZNF585B	0.188058113	0.460575483
ZNF586	0.175773734	0.582696087
ZNF589	0.528056542	0.019775221

ZNF593	-0.068649495	0.698320209
ZNF596	0.314635496	0.058479765
ZNF597	0.118317837	0.566875679
ZNF600	0.370800178	0.066665076
ZNF607	0.519100982	0.023847803
ZNF608	1.02320559	0.009991617
ZNF609	-0.054801844	0.711061769
ZNF614	0.259814054	0.376402549
ZNF616	0.415932228	0.026987419
ZNF619	0.15504903	0.2304955
ZNF620	0.340466196	0.136027186
ZNF621	1.017147015	0.000204607
ZNF622	-0.209076026	0.340516155
ZNF624	-0.035727105	0.804558867
ZNF627	0.301377933	0.084560537
ZNF630	-0.184597841	0.308958479
ZNF639	0.372348111	0.07634901
ZNF641	0.242567102	0.197687255
ZNF644	0.28372847	0.150454182
ZNF646	0.41957647	0.012228465
ZNF653	-0.166976237	0.335797568
ZNF654	0.117399769	0.63571801
ZNF660	0.817157709	0.015223102
ZNF664	0.235271224	0.22231702
ZNF665	0.268689743	0.149621879
ZNF668	0.03135293	0.872692977
ZNF669	0.338718088	0.130707646
ZNF671	0.315619894	0.087441348
ZNF672	0.289654227	0.147149625
ZNF675	0.23665788	0.161711365
ZNF676	-0.101981028	0.823896006
ZNF677	-0.01512081	0.959575538
ZNF679	-0.074700653	0.559782053
ZNF680	0.551949982	0.142881619
ZNF681	0.485400278	0.348455731
ZNF682	0.354696182	0.05199819
ZNF684	0.136840067	0.516550683
ZNF687	0.172116914	0.344494174
ZNF688	0.166560718	0.307475995
ZNF689	0.307509414	0.217502534
ZNF691	0.712659905	0.000879799
ZNF696	0.218896318	0.151109549
ZNF699	-0.083701063	0.866396155
ZNF7	0.089541683	0.541406687
ZNF70	0.659584978	0.009991617
ZNF700	0.439662348	0.147889899
ZNF703	-0.323627946	0.149184941
ZNF705A	-0.037833001	0.75582128

ZNF705E	-0.168425301	0.481859178
ZNF705G	-0.234389594	0.079519714
ZNF707	0.022479023	0.892887186
ZNF709	-0.03959786	0.857392501
ZNF71	-0.002581031	0.993549673
ZNF710	0.001433779	0.994477384
ZNF711	0.750280334	0.038596937
ZNF74	0.003332241	0.986045302
ZNF740	0.437186982	0.010398332
ZNF746	0.262919949	0.282289556
ZNF747	-0.027138152	0.906641603
ZNF750	-0.126912264	0.218311247
ZNF75D	0.19152724	0.283716255
ZNF76	0.614520555	0.022930456
ZNF764	0.201548382	0.189562427
ZNF768	0.273884098	0.277225602
ZNF77	0.207961137	0.187203129
ZNF770	-0.125307486	0.760373199
ZNF772	0.047866217	0.84194477
ZNF773	0.334357594	0.220586083
ZNF774	0.072830472	0.836438676
ZNF775	0.391178611	0.069838947
ZNF777	-0.161813053	0.250506267
ZNF780A	0.132924035	0.63571801
ZNF781	0.684641108	0.041663268
ZNF782	0.424968113	0.039819061
ZNF783	1.453111646	0.000927703
ZNF784	-0.095994538	0.674828435
ZNF785	0.516747697	0.00711757
ZNF787	-0.289839939	0.233795457
ZNF789	0.534050927	0.010822959
ZNF79	0.599038125	0.01505056
ZNF790	0.730521118	0.003709792
ZNF791	0.281466656	0.136009863
ZNF792	0.49918206	0.010348041
ZNF8	0.1986162	0.255106721
ZNF80	0.039121399	0.794902982
ZNF800	0.801338585	0.000690887
ZNF804A	-0.019027434	0.965060729
ZNF804B	-0.723005991	0.003951862
ZNF808	0.120383827	0.651747521
ZNF81	0.31580382	0.15015411
ZNF813	0.210585472	0.455880519
ZNF827	0.469948449	0.086231823
ZNF83	0.669797859	0.057216668
ZNF830	0.177467564	0.287234393
ZNF84	0.762538106	0.022582499
ZNF843	-0.118641113	0.506700199

ZNF844	0.675797347	0.016780753
ZNF862	0.485353249	0.002062218
ZNF875	0.563296571	0.013203199
ZNF879	0.127464003	0.572051855
ZNF91	-0.254410563	0.487860245
ZNF92	0.513431792	0.010454593
ZNF93	0.655389239	0.164294375
ZNFX1	0.360068671	0.10935091
ZNHIT1	0.398920329	0.059020938
ZNHIT3	0.001917275	0.992920546
ZNHIT6	0.265555069	0.211051989
ZNRD2	-0.599092542	0.010555103
ZNRF1	-0.342494787	0.084229601
ZNRF2	1.431069009	0.004240643
ZNRF3	-0.772443459	0.023487605
ZNRF4	-0.043458046	0.798159671
ZP2	-0.002582336	0.981721885
ZP3	0.009324128	0.958982209
ZP4	-0.046656857	0.746817551
ZPBP	-0.026860609	0.747075649
ZPBP2	-0.137218221	0.157061337
ZPLD1	-0.479576972	0.234051376
ZPR1	0.122426499	0.511971209
ZRANB1	-0.671790408	0.01930396
ZRANB2	0.357626595	0.023675041
ZRANB3	0.065914306	0.779238574
ZRSR2	0.310790692	0.180150463
ZSCAN1	0.157082913	0.478755294
ZSCAN10	-0.063714391	0.48715344
ZSCAN12	0.318530647	0.041553053
ZSCAN16	0.36699268	0.079825037
ZSCAN18	-0.01760523	0.932999857
ZSCAN2	0.167067548	0.262270598
ZSCAN20	0.107449133	0.416702311
ZSCAN21	0.655865274	0.011141587
ZSCAN22	0.022172531	0.925298218
ZSCAN25	0.425058038	0.023108209
ZSCAN29	0.306097346	0.216070315
ZSCAN32	0.115970979	0.46040277
ZSCAN4	-0.096240507	0.305389973
ZSCAN5A	0.063381936	0.645864133
ZSCAN5C	-0.008513192	0.957816002
ZSWIM1	0.13777397	0.546625793
ZSWIM2	-0.104107373	0.209291851
ZSWIM8	0.275912826	0.360294372
ZSWIM9	-0.098765938	0.520931342
ZUP1	0.123381492	0.484732467
ZW10	0.419591758	0.030011024

ZWINT	0.438305369	0.12962083
ZXDA	0.237739433	0.543983376
ZXDB	0.678199697	0.089117643
ZXDC	0.610975532	0.006776741
ZYG11A	0.046708569	0.733786115
ZYX	0.247374262	0.618576683
ZZEF1	0.284051809	0.07970029
ZZZ3	0.171316405	0.335608162
