# Supplementary figures and images for: LYZ Gene as a Novel Therapeutic Target and Diagnostic Biomarker in Glioblastoma: Insights from Multi-Omics Analysis and Functional Validation
Source: Biology (Basel). 2025 Dec 19;15(1):9. doi: 10.3390/biology15010009 (PMC12784846; doi:10.3390/biology15010009)

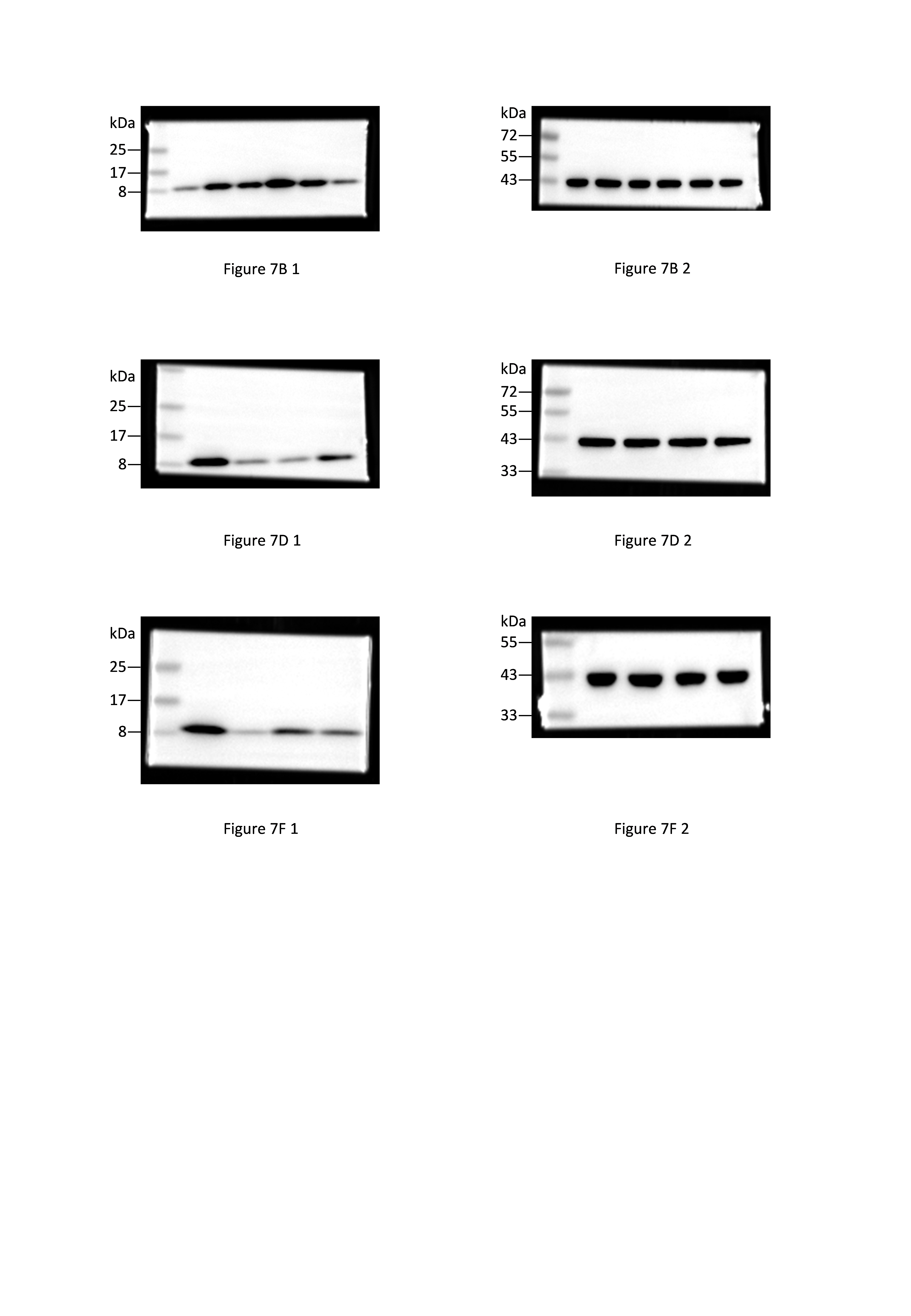

Supplement: Supplementary file 1 [file biology-15-00009-s001.zip › Supplementary Files/Figure S2.tif]
